# Desymmetrization of *meso*-Pyrrolidines via Oxoammonium-Catalyzed Enantioselective Hydride Transfer

**DOI:** 10.1021/jacs.5c20639

**Published:** 2026-02-26

**Authors:** Jonas Rein, Bartosz Górski, Ayça M. Keskin, Minh Hoang Le, Song Lin

**Affiliations:** Department of Chemistry and Chemical Biology, 5922 Cornell University, Ithaca, New York 14853, United States

## Abstract

We report the oxidative desymmetrization of urea-protected pyrrolidines via site-selective hydride transfer from enantiotopic C–H bonds. The optimal oxoammonium–peptide conjugate catalyst provided over 90% *ee* across all tested pyrrolidines, providing products that can readily undergo subsequent N-deprotection and other derivatization reactions to form medicinally relevant compounds. We isolated key on-cycle catalytic intermediates, which allowed us to elucidate both the mechanism of catalytic activation and the origin of stereochemical induction in detail. In particular, a stereochemical model for asymmetric induction emerged from analyzing a covalent catalyst–substrate adduct, which served as an isolable analog of the enantiodetermining transition state. In this model, a tight hydrogen bond between the urea protecting group and the peptide directs the asymmetric hydride transfer.

## Graphical abstract

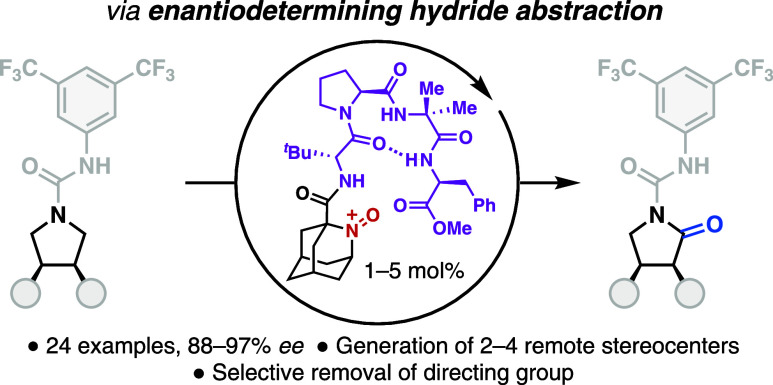

## Introduction

Pyrrolidine is a ubiquitous motif in active pharmaceutical ingredients and is the third most prevalent heterocycle among pharmaceuticals approved by the US FDA between 2013–2023.^1^ As such, efficient and selective methods for their synthesis and structural diversification are in high demand.^1–4^ The direct activation of C–H bonds is an attractive strategy to rapidly access substituted pyrrolidines from readily available starting materials. In this regard, the oxidation of α-C–H bonds in pyrrolidine and related aliphatic N-heterocycles have been extensively investigated^5^ using metal catalysis,^6–10^ photocatalysis,^11–13^ electrosynthesis,^14,15^ biocatalysis,^16,17^ organocatalysis,^18^ and hydride transfer catalysis.^19–22^ Nevertheless, enantioselective variants of these transformations remain rare. Achieving this objective requires either direct enantioselective activation of a C–H bond or nonselective C–H activation followed by asymmetric functionalization of the resultant prochiral intermediate. These mechanistically distinct paradigms provide access to divergent substitution patterns (Figure 1A): nonselective α-C–H activation followed by enantioselective functionalization is limited to generating a singular stereocenter at the α-position,^22–26^ whereas enantioselective activation (i.e., desymmetrization or kinetic resolution) could generate two or more stereocenters at remote positions, offering access to complex stereochemical arrays including chiral polycyclic architectures. Despite the prevalence of 3,4-disubstituted pyrrolidines in biologically active compounds including drug candidates (Figure 1B),^27–33^ their desymmetrizing C–H functionalization remains underdeveloped, likely because the challenge of differentiating enantiotopic C–H bonds is compounded with inherent difficulties of achieving chemo- and site-selective C–H activation.

**Figure 1:**
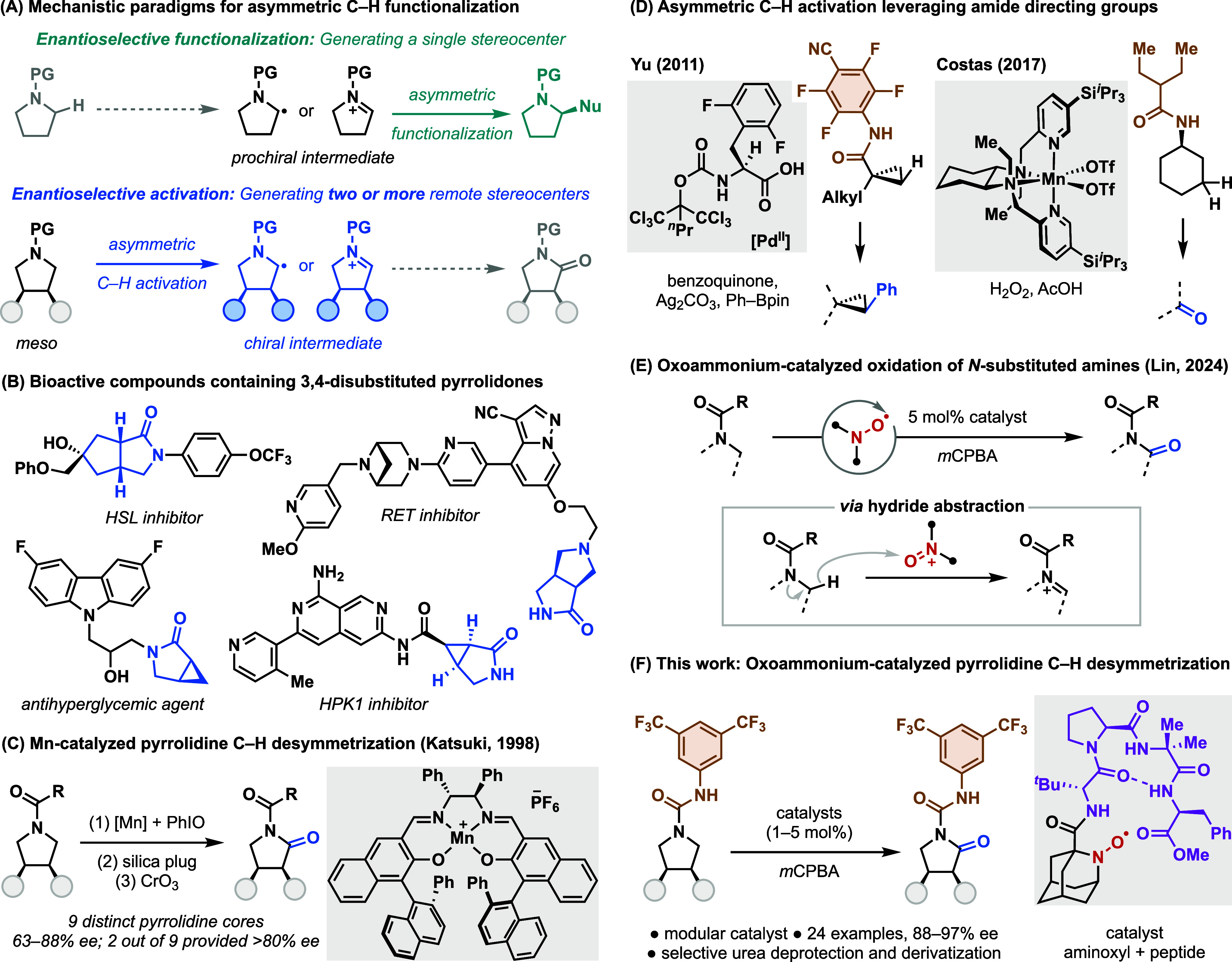
Background and introduction.

A rare precedent for the desymmetrization of N-substituted *meso*-amines to chiral amides was introduced by Katsuki and coworkers, who employed a Mn­(salen)-based hydrogen-atom abstraction (HAA) catalyst to effect C–H hydroxylation, which was followed by Jones oxidation to furnish amide products (Figure 1C).^34^ The method provided products in generally moderate yield and enantioselectivity. Recently, Costas and Sigman disclosed an Mn-catalyzed oxidative desymmetrization and alkoxylation of sulfonamide-protected piperidines via an analogous radical-based mechanism.^35^ Beyond these pioneering contributions, desymmetrizing C­(sp^3^)–H functionalization has been documented in a limited number of reports in other contexts, often employing elegant directing group strategies to strengthen and rigidify catalyst–substrate binding. For example, Yu and coworkers showed that a perfluorinated amide could direct the enantioselective arylation of a cyclopropane C–H bond via palladium-catalyzed C–H activation (Figure 1D, left).^23,36,37^ More recently, Costas and coworkers used a similar strategy in the asymmetric β-C–H oxidation of cyclohexanes through manganese-oxo-mediated HAA (Figure 1D, right).^38,39^ Additionally, a rare example of an organocatalytic C–H desymmetrization was reported by Phipps and coworkers, who leveraged a chiral quinuclidine catalyst to achieve epimerization of *meso*-diols via HAA.^40^

Recently, we developed the α-C–H oxidation of N-substituted amines including sulfonamides, amides, and carbamates, catalyzed by oxoammonium ions generated from aminoxyl radicals (Figure 1E).^19^ This reaction proceeds via a hydride transfer from the substrate to the catalyst, delivering an iminium intermediate that is further transformed into a lactam product.^19,41^ We envisioned that this catalytic mechanism could be rendered enantioselective using appropriate chiral aminoxyl catalysts, achieving the desired desymmetrization of *meso*-pyrrolidines. Traditional approaches to enantioselective aminoxyl catalysis rely on embedding chirality directly into the catalyst core with sterically demanding substituents.^42–44^ These catalysts lack structural tunability and show limited activity toward C–H activation due to the steric hindrance around the aminoxyl group. To address these limitations, we recently developed a modular platform in which a simple, achiral aminoxyl core is conjugated to a small peptide via amide coupling.^45^ This strategy substantially simplifies catalyst synthesis, granting access to diverse catalyst libraries for rapid reaction optimization. We initially applied it to the desymmetrization of *meso*-diols, for which the peptide framework was shown to deliver high enantioselectivity through noncovalent substrate recognition.

In this work, we report the development of an oxidative desymmetrization of pyrrolidines via enantiodetermining hydride abstraction catalyzed by modular aminoxyl–peptide conjugates. High enantioselectivity across a wide range of substrates is achieved using a readily deprotected urea directing group, which engages the catalyst through a strong hydrogen bonding interaction. A stereochemical model is proposed wherein enantioinduction is governed by stabilizing noncovalent interactions, which was informed by the isolation and conformational characterization of a covalent on-cycle catalyst–substrate adduct that mimics the transition state.

## Results and Discussion

### Reaction Design and Development

To extend the scope of oxoammonium–peptide catalysis from alcohol oxidation to the desymmetrization of *meso*-pyrrolidines, we recognized the need to optimize both the aminoxyl core and the peptide scaffold to attain high reactivity and stereocontrol. We previously found that removing bulky substituents near the aminoxyl unit extends reactivity beyond alcohol oxidation to the functionalization of more challenging substrates.^19,20,41^ We thus chose AzcH as an unhindered catalyst core, which was shown effective in the racemic α-C–H oxidation of N-substituted amines. For the peptide, we built on our optimal sequence in previous studies on diol oxidation^45,46^ but modified it to create a shorter, trimeric peptide (H-*d*Tle-Pro-Aib-OMe) that retains key noncovalent recognition features while offering easier synthesis and greater conformational flexibility than the previously used pentamers.

Using Cbz-protected 6,6-dimethyl-3-azabicyclo[3.1.0]­hexane **1** as the model substrate, we found that 1 mol % catalyst **P1** delivered lactam **2** in 95% yield with 35% *ee* using *meta*-chloroperoxybenzoic acid (*m*CPBA) as a stoichiometric oxidant (Figure 2A). In our previous work on diol oxidation, we uncovered critical roles that noncovalent interactions such as hydrogen bonding play in enforcing strong substrate–catalyst association. Thus, we focused our initial efforts on identifying an optimal N-protecting group that may provide such binding interactions. An ideal protecting group would direct the desired oxidation in a fashion that is agnostic to the remote substituents on the pyrrolidine to allow for a broad substrate scope, while also allowing for easy deprotection to reveal the lactam after the reaction. Initial screening revealed that modest enantioinduction could be achieved with numerous protecting groups (including commonly used Boc and Cbz) with *ee* ranging from 21–47% (Figure 2B). Notably, we found that arylurea-derived substrates (**4-Ar**) experienced a dramatic boost in enantioselectivity, with 3,5-bis­(trifluoromethyl)­phenyl urea showing the highest 82% *ee*. This group also enabled various product derivatizations including facile N-deprotection (vide infra).

**Figure 2:**
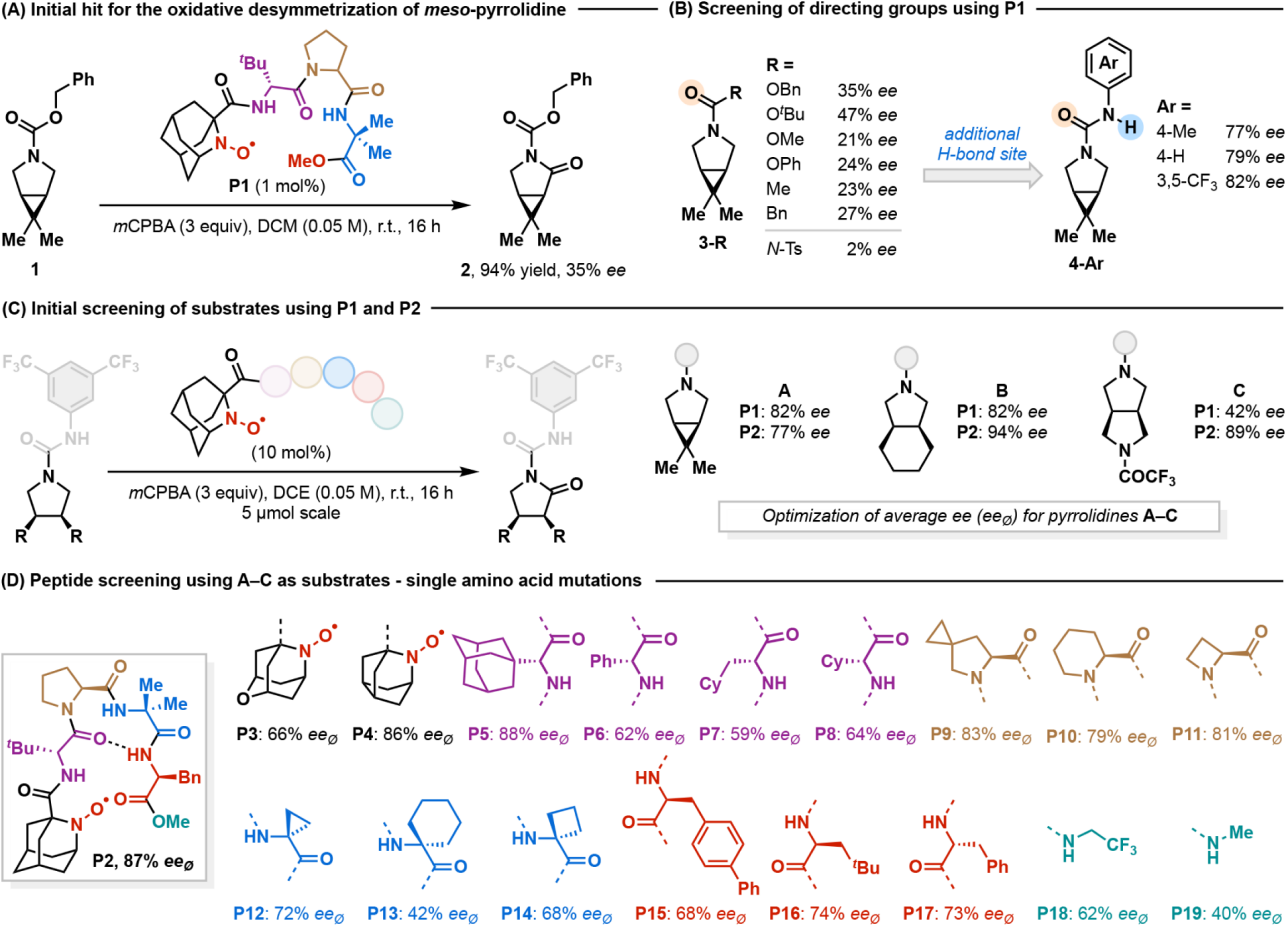
Reaction discovery and optimization. (A) Initial reaction discovery. (B) Discovery of the urea directing group. (C) Reaction optimization through peptide screening. The average %*ee* reported is based on substrates **A**, **B**, and **C**. (D) Screening of aminoxyl-peptide catalysts. **P5** provided a 1% higher average *ee* than **P2**, but was not selected for all further experiments due to the high cost of 1-adamantyl-glycine derivatives.

We then further optimized the catalyst peptide structure using a generality-oriented optimization strategy via microscale (5 μmol) high throughput experimentation,^45,47^ screening a panel of substrates against a group of 19 catalysts with the goal to obtain a catalyst capable of providing high enantioselectivity across substrates with varying structures (Figure 2C). We initially employed three pyrrolidines with different bicyclic ring scaffolds (**A**: [3.1.0], **B**: [4.3.0], and **C**: [3.3.0]). The initial trimeric peptide **P1** provided an average *ee* of 67%. Extending the peptide by an additional phenylalanine residue (**P2**, AzcH-*d*Tle-Pro-Aib-Phe-OMe) significantly boosted the average *ee* to 87%. From this catalyst, single residue mutations were carried out to systematically vary the sterics, bond angles, and dihedrals at each amino acid (Figure 2D). Nevertheless, we found that **P2** remained one of the most general and selective among all 19 catalysts surveyed. Indeed, this tetrameric amino acid sequence has been widely used in small peptide catalysis with other types of catalytic residues, representing a privileged scaffold owing to its propensity to form a rigid β-turn secondary structure as a well-defined environment for stereoinduction.^48^ We note that we also screened these catalysts for several cyclic amines with larger ring sizes, including a morpholine and two bridged bicyclic [3.2.1] azepanes. While high enantioselectivities were attained (69–85% *ee*), we were unable to identify conditions that provided satisfactory yields for these as well as piperidine- and azetidine-type substrates (see SI Section 3.5 for all unsuccessful substrates attempted).

### Condition Optimization and Scope

We further optimized the reaction conditions on a 0.1 mmol scale using compound **A** as the model substrate and found that exposure to air and moisture was tolerated. Diluting the reaction and adding a catalytic amount of HNTf_2_ increased the selectivity, allowing us to achieve 84% isolated yield with 92% *ee* (see SI Section 3.4). The catalytic strong acid promotes the disproportionation of the aminoxyl radical, an off-cycle resting state of the active oxoammonium catalyst.^41^ Under these optimal conditions, we explored the scope of the transformation (Figure 3A) and found that it exhibits excellent generality for a suite of skeletally diverse pyrrolidines, providing an average of 94% *ee* across the entire scope with the highest reaching 97% *ee*. We found that 1 mol % of **P2** was sufficient to desymmetrize pharmaceutically relevant [3.2.0] and [3.1.0] azabicycles (**5**–**10**),^31,33^ while an increased catalyst loading of 5 mol % provided satisfactory results for all remaining less reactive substrates. Excellent enantioselectivities were obtained for a panel of analogs of substrate **A** with electronically distinct arylurea groups (**6-R**). The reaction tolerated bicyclic skeletons with varying ring sizes, including a bispyrrolidine (**12**) as well as tetrahydrofuran- (**14**), and dioxane- (**16**) fused pyrrolidines. Notably, in product **15** an array of four contiguous stereocenters were established though a single process of enantioselective hydride transfer. In addition, various functional groups including acetals (**19**), esters (**8**, **9**, **17**, **20**), unactivated (**14**, **16**) and benzylic ethers (**21**), amides (**12**), and geminal dihalides (**18**, **22**) were found to be compatible. Beyond azabicycles, monocyclic pyrrolidine **17** was also synthesized with excellent yield and enantioselectivity.

**Figure 3:**
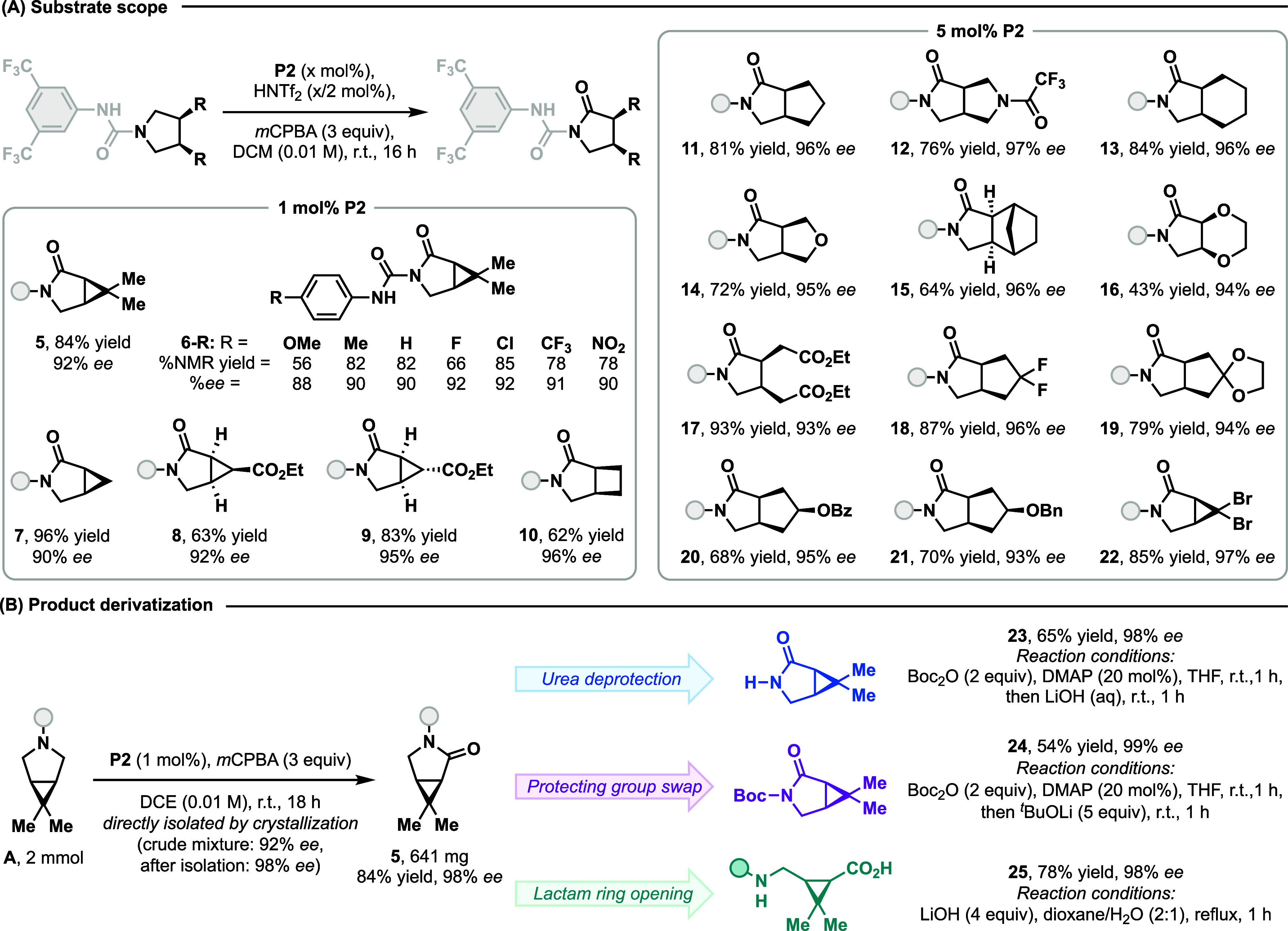
Scope of *meso*-pyrrolidines and selective derivatization of the chiral lactam. (A) All yields refer to isolated yields at the 0.1 mmol scale unless specified otherwise. %*ee* was determined by chiral HPLC after isolation. Absolute stereochemistry for **16** was established with an X-ray crystal structure and for **5** based on diastereomeric NMR of intermediate **P2·sub**; all other products were assigned based on analogy. (B) Reactions did not require agitation owing to the small scale. Reagents: DMAP = 4-(Dimethylamino)­pyridine, Boc_2_O = Di-*tert*-butyl dicarbonate.

To further enhance synthetic utility, we scaled up the reaction 20-fold and obtained product **5** via direct crystallization in 84% yield with an increased *ee* of 98%. Because ureas are unconventional N-protecting groups, we established conditions to remove them for derivatization of the product (Figure 3B). Preactivation of the N–H moiety with Boc_2_O and DMAP followed by direct treatment with LiOH yielded the unprotected lactam (**23**) in good yield. Additionally, the N-Boc urea intermediate could also undergo alcoholysis with lithium *tert*-butoxide, providing Boc-protected lactam **24** in 54% yield. Finally, direct hydrolysis of **5** with LiOH resulted in ring-opening of the pyrrolidinone, leading to an enantioenriched analog of gamma-aminobutyric acid (GABA) inhibitors (**25**).^49^ Thus, this highly enantioselective desymmetrization protocol facilitates the synthesis of valuable chiral pyrrolidones—both as common pharmacophores themselves^1,3^ and as new precursors to other medicinally relevant building blocks.^50–52^

### Mechanistic Hypothesis

Finally, we carried out additional investigations to elucidate the origin of enantioselectivity in the desymmetrization of substrate **A** (Figure 4). The general mechanism of oxoammonium-catalyzed C–H oxidation, including the mechanism of catalytic turnover, was established through detailed studies of a related reaction system in our previous work.^19,41^ The reaction mechanism starts with an initial hydride transfer from **A** to oxoammonium **P2** ^**+**^ followed by an in-cage trapping of the resulting iminium ion (**27**) by hydroxylamine **P2H**, furnishing a catalyst–substrate adduct (**P2·sub**) (Figure 4E). This adduct undergoes N-oxidation by *m*CPBA (**28**) and then collapses to form lactam product **5** and **P2H** via Cope-type elimination. **P2H** was further transformed to **P2** ^**+**^ via oxidation and disproportionation to close the cycle.^41^ The feasibility of this proposed mechanism in the present system was interrogated through a series of stoichiometric experiments as well as isolation and characterization of catalytic intermediates (Figure 4A).

**Figure 4:**
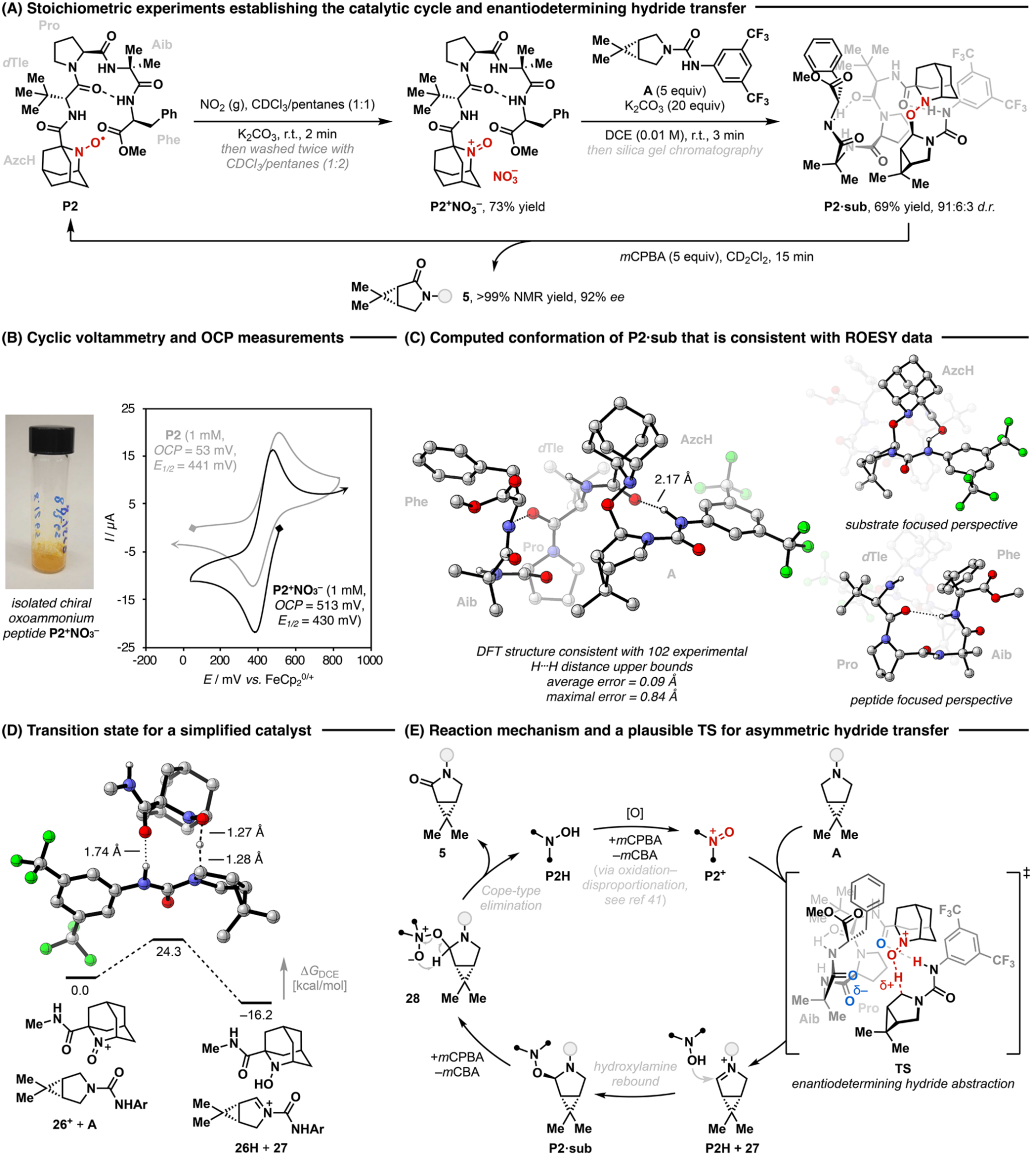
Mechanistic studies and stereoelectronic model for enantioinduction. (A) Stoichiometric experiments characterizing isolable intermediates in the mechanistic cycle. (B) Cyclic voltammetry and open circuit measurements were performed in 0.1 M tetrabutylammonium hexafluorophosphate in MeCN electrolyte using a glassy carbon working electrode with a 3.2 mm diameter, a platinum wire counter electrode, and an Ag/AgNO_3_ (0.01 M in MeCN) reference electrode and then referenced against the ferrocene/ferrocenium couple. (C) Initial structures were obtained from a CREST^55^ based conformational search followed by geometry optimization at the AimNet2 ^56^ level of theory using Rowan.^57^ The structure was consistent with manual dihedral optimization based on the NOE map and featured a β-turn conformation.^53^ The structure was then multifurcated by systematic variation of bond dihedrals and geometry optimization in DFT at the M062*X*/6-31G­(d,p)^54^ level of theory and candidate was reevaluated and scored based on the average error in H···H when compared to the experimental upper bounds established by ROESY NMR. (D) Lowest energy hydride transfer transition state found at the M062*X*/6-311G­(d,p)/SMD­(DCE)//M062*X*/6-31G­(d,p)/SMD­(DCE) level of theory;^54^ 9 distinct transition state structures were localized. (E) Proposed reaction mechanism and stereochemical model for asymmetric induction. The blue O atoms labeled with δ^–^ in the TS are from the Pro and Aib residues that are proposed to stabilize the developing positive charge on the substrate.

### Isolation and Characterization of Catalytic Intermediates

Peptide-appended oxoammonium salt **P2** ^**+**^ **NO**_**3**_^–^ was successfully isolated as a bench-stable, bright yellow solid through oxidation of the corresponding aminoxyl **P2** with nitrogen dioxide. The oxidation state and speciation of **P2** ^**+**^ **NO**_**3**_^–^ were confirmed through electroanalysis (Figure 4B). Open circuit potential (OCP) measurements were used to determine the equilibrium potential at which no current flows in an electrochemical cell, while cyclic voltammetry provided the thermodynamic half-wave potential *E*_1/2_ for the reversible redox couples. The ratio of oxoammonium to aminoxyl radical in the analyte was then calculated using the Nernst equation (OCP = *E*_1/2_ – 59.2 log­([aminoxyl]/[oxoammonium]) mV). Thus, the **P2** ^**+**^ **NO**_**3**_^–^ sample we obtained (OCP = 513 mV; *E*_1/2_ = 430 mV) contains 96% of the oxoammonium salt. The low concentration of paramagnetic aminoxyl radical also permitted characterization by NMR. As expected for a highly electron-deficient oxoammonium ion, the protons on the azaadamantyl fragment of **P2** ^**+**^ were up to 2.3 ppm more deshielded than in its lower-oxidation-state alkoxylamine form (**P2·sub**).

We then performed stoichiometric experiments to study each step in the proposed catalytic cycle. Exposure of **P2** ^**+**^ **NO**_**3**_^–^ to five equivalents of substrate **A** allowed for the isolation of the hydroxylamine-substrate adduct **P2·sub** in 69% yield, consisting of three diastereomers in a 91:6:3 *d.r.* To assign the stereochemistry, **P2·sub** was then oxidized using *m*CPBA, providing **5** in >99% NMR yield with 92% *ee* (96:4 *e.r.*). Accordingly, the first two diastereomers were converted to the major enantiomeric product, showing that they resulted from the desired pathway with **P2H** added to either face of the chiral iminium. The third diastereomer, on the other hand, was formed via the minor pathway leading to the opposite enantiomeric product. The matching enantioselectivity obtained in the stoichiometric and catalytic conditions indicated that the isolable oxoammonium **P2** ^**+**^ **NO**_**3**_^–^ is indeed the active hydride abstractor during catalysis, and that this step is enantiodetermining. CV and NMR analysis of the reaction mixture after oxidation of adduct **P2·sub** with *m*CPBA also confirmed the formation of free catalyst as aminoxyl radical **P2**, further supporting the role of **P2·sub** as a catalytic intermediate.

### Conformational Analysis of Transition State Analogue

After confirming the enantiodetermining step, we conducted both experimental and computational investigations to gain insights into the molecular interactions that selectively stabilize the major transition state (TS). We opted against the traditional computational approach to establishing TS models, as the vast conformational space of the peptide catalyst impedes exhaustive conformational sampling of hydride abstraction transition states for accurate quantitative analysis. Rather, we recognized that adduct **P2·sub** may provide an experimental entry point to help understand catalyst–substrate interactions that may be present in the preceding hydride transfer TS.

To assign the solution structure of **P2·sub**, detailed 2D NMR experiments were performed in tandem with computational modeling to identify conformations consistent with rotating-frame Overhauser enhancement spectroscopy (ROESY) (Figure 4C).^53^ We generated an ensemble of computational conformers and evaluated them against 102 experimental upper pairwise distance bounds for H···H distances within **P2·sub** obtained from quantitative nuclear Overhauser effect (NOE) analysis (see Figure 4C caption and SI for details). This yielded a density functional theory (DFT) optimized structure with <0.1 Å average deviation from the experimental data. This solution conformation of **P2·sub** features a H-bond between the urea N–H and the carbonyl adjacent to the aminoxyl core (2.17 Å), as well as a β-turn induced by the Pro consistent with literature precedent for similar amino acid sequences.^53^ The urea N–H and the bulky *d*Tle residue lock the β-turn in a conformation where the carbonyl groups of the Pro and Aib residue face the pyrrolidine substrate.

We then performed computational TS analysis for the hydride transfer from **A** to a simplified catalyst **26** ^**+**^ at the M062*X*/6-311G­(d,p)/SMD­(DCE)//M062*X*/6-31G­(d,p)/SMD­(DCE) level of DFT (Figure 4D).^54^ The lowest free-energy TS resembles the computed structure of **P2·sub** regarding connectivity and catalyst–substrate orientation (TS_model_ (^1^N–C···H···O=^2^N^+^)/**P2·sub** (^1^N–C–O–^2^N): distances: O–C 2.48 Å/1.43 Å; C–^2^N 3.00 Å/2.34 Å; O^2^–N: 1.26 Å/1.43 Å; dihedral: ^1^N–C–O–^2^N 20°/58°; angle: C–O–^2^N 102°/109°). A tight H-bond (1.74 Å) between the N–H of the urea protecting group and the carbonyl of the catalyst constrains the orientation of the oxoammonium core with respect to the pyrrolidine. Notably, the increasing positive charge in the substrate during hydride transfer further strengthens this stabilizing H-bond relative to uncharged **P2·sub**. This similarity in catalyst–substrate trajectory and orientation (see comparison in SI, Figure S8) suggests that the noncovalent interactions in the major TS should resemble those in the hydroxylamine-substrate adduct **P2·sub**. As informed by the structure of **P2·sub**, we postulate that the peptide β-turn in the full catalyst orients the carbonyl groups of the Pro and Aib residues toward the substrate to further stabilize the developing positive charge during hydride abstraction, while minimizing steric clashes between the substrate and the peptide side chains (see TS in Figure 4E). This hypothesis is consistent with the high enantioselectivity observed using the truncated peptide **P1** (AzcH-*d*Tle-Pro-Aib-OMe) featuring both Pro and Aib. Together, these noncovalent interactions enable differentiation between the enantiotopic α-C–H bonds.

## Conclusion

In conclusion, we report an oxidative desymmetrization of pyrrolidines via oxoammonium–peptide catalysis. The work extends the scope of asymmetric hydride transfer catalysis beyond alcohol oxidation, showcasing its potential as an effective approach for asymmetric C–H functionalizations alongside traditional transition-metal-catalyzed and radical-based methods. This reaction also constitutes a rare example of organocatalytic asymmetric C–H activation, and to the best of our knowledge the first example of an asymmetric C–H hydride abstraction where an intermolecular^58–60^ C–H activation step is enantiodetermining. Owing to its distinct mechanism from homolytic, anionic, or metal-based activation pathways, we anticipate that oxoammonium catalysis will find broad and complementary applications in enantioselective organic synthesis.

## Supplementary Material



## Notes

Experimental section; procedures for all results described above (catalyst optimization; catalyst and substrate synthesis; substrate scope; mechanistic studies); characterization data; computational data; X-ray and NMR spectra (PDF)
