# Supplementary material for: Desymmetrization of meso-Pyrrolidines via Oxoammonium-Catalyzed Enantioselective Hydride Transfer
Source: J Am Chem Soc. 2026 Feb 26;148(9):9650–8. doi: 10.1021/jacs.5c20639 (PMC12983322; doi:10.1021/jacs.5c20639)
Supplement: Supplementary file 1 [file ja5c20639_si_001.pdf]

Supporting Information for

Desymmetrization of Meso-Pyrrolidines *via* Oxoammonium-Catalyzed Enantioselective Hydride Transfer

Jonas Rein[1‡], Bartosz Górski[1‡], Ayça M. Keskin[1], Minh Hoang Le[1], Song Lin[1]*

[1]Department of Chemistry and Chemical Biology, Cornell University, Ithaca, New York 14853, United States.

‡J. R. and B. G. contributed equally.

*Correspondence to: songlin@cornell.edu

Contents

1. General Information: 3

2. Catalyst Synthesis 5

3. Reaction Optimization: 6

3.1. General Procedure for Microscale Screening 6

3.2. Initial Protecting Group Optimization 7

3.3. Catalyst Optimization and Characterization 8

3.4. Reaction Optimization at 0.1 mmol Scale 10

3.5. Non-Pyrrolidine *meso*-Heterocylces 11

4. Substrate Scope 12

5. Substrate Preparation: 26

6. Product Derivatization 44

7. Mechanistic Study: 46

7.1. Isolation and Characterization of Oxoammonium $P2^+NO_3^-$ 46

7.2. Preparation, Characterization, and Reactivity of P2·sub 50

7.3. ROESY analysis of P2·sub 55

7.4. Transition State Analysis for a Model System 61

7.4.1. Cartesian Coordinates 62

8. HPLC Traces 72

9. Crystal Structure 107

10. NMR Spectra 109

11. References: 267

1. General Information:

Commercial reagents were purchased from Sigma Aldrich, Alfa Aesar, Acros, Chem-Impex, TCI, AK Scientific, Oakwood, and Ambeed. All reactions were run with HPLC grade solvents without further purification and are set up under ambient conditions without protection against air or moisture unless otherwise specified.

Preparative scale reactions (>0.05 mmol) were monitored by thin layer chromatography (TLC) [EMD Millipore silica gel 60 F254 precoated plates (0.25 mm thickness)], crude ultra-high performance liquid chromatography-mass spectrometry (UPLC/MS), crude gas chromatography–mass spectrometry (GC-MS), or proton nuclear magnetic resonance spectroscopy (^{1}H NMR). TLC visualization was performed under a UV lamp or $KMnO_4$ stain developed with heat. Solvent evaporation was conducted by rotary evaporation at the appropriate temperature and pressure. The oxidized urea products are stable during UPLC analysis with H_2O/MeCN (0.1% formic acid buffer); typical Boc-fragmentation patterns are observed on electrospray ionization (ESI). The 3,5-bis(trifluoromethyl)phenyl urea moiety was found to be an excellent chromophore with a maximum absorption at approximately 254 nm, and a suitable mass spectrometry handle for both positive and negative mode ESI. 4,4′-di-*tert*-butylbiphenyl (0.25 equiv) may be used as an internal standard during the reaction without any changes to yield or selectivity for room temperature reactions. Flash chromatography was performed using silica gel 60 (230-400 mesh) from SiliCycle or with an automated Biotage® Isolera™ One flash purification system equipped with a Biotage® Sfär Silica D Duo 60 μm (10 g, 25 g, 50 g, 100 g) column. All reported yields reflect spectroscopically (^{1}H-NMR) pure material unless otherwise stated.

Proton nuclear magnetic resonance (^{1}H NMR) spectra and carbon nuclear magnetic resonance (^{13}C NMR) spectra were recorded on a Bruker AVIII HD 500 MHz spectrometer equipped with a 500 MHz Bruker AVIII HD with BBO Prodigy cryoprobe, a 400 MHz Bruker AVIII HD with BBFO probe, or a 600 MHz Varian INOVA. Chemical shifts for protons are reported in parts per million (ppm) downfield from tetramethyl silane and are referenced to residual protium in the NMR solvent ($CDCl_3$ = δ 7.26, CD_2Cl_2 = δ 5.32, MeCN-d_3 = δ 1.94, Methanol-d_4 = δ 3.31, benzene-d_6 = δ 7.16, acetone-d_6 = δ 2.05, DMSO-d_6 = δ 2.50). Chemical shifts for carbons are reported in parts per million downfield from tetramethyl silane and are referenced to the carbon resonances of the solvent ($CDCl_3$ = δ 77.16, CD_2Cl_2 = δ 53.84, MeCN-d_3 = δ 1.32, methanol-d_4 = δ 49.00, benzene-d_6 = δ 128.06, acetone-d_6 = δ 29.84, DMSO-d_6 = δ 39.52). Data are represented as follows: chemical shift (ppm), multiplicity (br. s = broad singlet, s = singlet, d = doublet, t = triplet, q = quartet, dd = doublet of doublets, hept = heptet, m = multiplet), coupling constants in Hertz (Hz), integration.

Gas chromatography with flame ionization detector (GC-FID) was performed on an Agilent 8860 GC system equipped with a HP-5 (30 m × 0.32 mm I.D. × 0.25 μm) column using helium as the carrier gas. Gas chromatography–mass spectrometry (GC-MS) was performed on an Agilent 8860 GC system equipped with a HP-5 MS UI (30 m × 0.25 mm I.D. × 0.25 μm) coupled to an Agilent 5977 GC/MSD using helium as the carrier gas.

Reverse phase liquid chromatography was carried out using an Agilent 1290 Infinity II LC system equipped with a multisampler, a DAD detector, an Agilent Single Quad LC/MSD iQ, and a Poroshell 120, EC-C18, 2.1 × 50 mm, 2.7 μm column. The HPLC was operating at 28 °C with a

1.0 mL/min flow rate of a binary eluent gradient of A or B (A = aqueous mobile phase (HPLC grade water with 0.1% formic acid), B = organic mobile phase (HPLC grade MeCN or MeOH with 0.1% formic acid)). Injection volumes were adjusted based on dilution of samples and ranged between 0.1–5.0 μL. High-resolution mass spectrometry (HRMS) was conducted on a Thermo Fisher Scientific Exactive series DART mass spectrometer. Enantiomeric excess was determined using either an Agilent 1290 Infinity II SFC equipped with a DAD detector and an Agilent Single Quad LC/MSD iQ, a Shimadzu HPLC system, or an Agilent 6890N Network GC system with the methods and columns indicated for each compound in section 10.

All electroanalytical studies were conducted with the BASi Epsilon without any protection from air or moisture. Measurements were performed in 0.1 M $TBAPF_6$ in MeCN. The working electrode is a 3.2 mm diameter glassy carbon working electrode and was polished with 1.0, 0.3 and 0.05 μm aluminum oxide and then sonicated in distilled water and acetone, then air dried before each scan. An $Ag/AgNO_3$ (0.01 M in MeCN (0.1 M $TBAPF_6$)) reference electrode is used. Before and after every batch of experiments, the potential is referenced to the ferrocenium/ferrocene redox couple ($Fc^{+/0}$). A platinum wire that is sonicated in acetone and then burnt for 10 seconds with a butane torch is used as a counter electrode. Cyclic voltammograms were acquired with a scan rate of 100 mV/s with a positive or negative scan direction.

2. Catalyst Synthesis

Synthesis of peptides is accomplished utilizing solution-phase HATU couplings. The synthesis of **P2** is provided as a representative example.

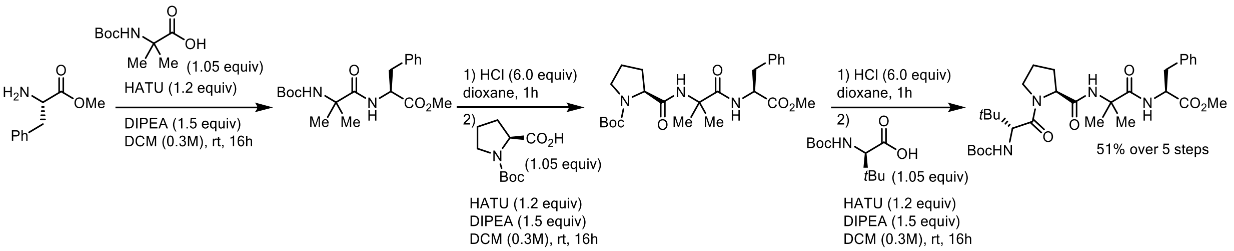

Coupling: To a suspension of **Boc-Aib-OH** (5.00 g, 24.6 mmol, 1 equiv), **H-Phe-OMe•HCl** (5.57 g, 25.8 mmol, 1.05 equiv), and HATU (11.20 g, 29.5 mmol, 1.2 equiv) in DCM (82 mL) was added DIPEA (10.7 mL, 61.5 mmol, 2.5 equiv). The reaction was allowed to stir at room temperature for 2 h prior to transferring to a separatory funnel, washing with saturated $NaHCO_3$ (100 mL), 10% citric acid (100 mL), and saturated $NaHCO_3$ (100 mL). The organic layer was dried over Na_2SO_4 and concentrated to yield crude **Boc-Pip-Aib-OMe**, which was used in the next step without further purification.

Deprotection: Boc-Aib-Phe-OMe (8.97 g, 24.6 mmol, 1.0 equiv) was placed in a round-bottom flask and 4 M HCl in dioxane was added (37 mL, 148 mmol, 6.0 equiv). The mixture was stirred for 1 h at room temperature, and then dioxane was evaporated on the rotary evaporator. The crude **H-Aib-Phe-OMe•HCl** product was dried *in vacuo* and used in the next step without further purification.

Coupling: To a suspension of crude **H-Aib-Phe-OMe•HCl** (24.6 mmol, 1.0 equiv), **Boc-Pro-OH** (5.54 g, 25.8 mmol, 1.05 equiv), and HATU (11.20 g, 29.5 mmol, 1.2 equiv) in DCM (82 mL) was added DIPEA (10.7 mL, 61.5 mmol, 2.5 equiv). The reaction was allowed to stir at room temperature for 2 h prior to transferring to a separatory funnel, washing with saturated $NaHCO_3$ (100 mL), 10% citric acid (100 mL), and saturated $NaHCO_3$ (100 mL). The organic layer was dried over Na_2SO_4 and concentrated to yield crude **Boc-Pro-Pip-Aib-OMe**, which was used in the next step without further purification.

Deprotection: Crude **Boc-Pro-Aib-Phe-OMe** (assumed 24.6 mmol, 1.0 equiv) was placed in a round-bottom flask and 4 M HCl in dioxane was added (37 mL, 148 mmol, 6.0 equiv). The mixture was stirred for 1 h at room temperature, and then dioxane was evaporated on the rotary evaporator. The crude product was dried *in vacuo* and used in the next step without further purification.

Coupling: To a suspension of crude **H-Pro-Aib-Phe-OMe•HCl** (24.6 mmol, 1 equiv), **Boc-*d*Tle-OH** (5.97 g, 25.8 mmol, 1.05 equiv), and HATU (11.20 g, 29.5 mmol, 1.2 equiv) in DCM (82 mL) was added DIPEA (10.7 mL, 61.5 mmol, 2.5 equiv). The reaction was allowed to stir at room temperature for 2 h prior to transferring to a separatory funnel, washing with saturated $NaHCO_3$ (100 mL), 10% citric acid (100 mL), and saturated $NaHCO_3$ (100 mL). The organic layer was dried over Na_2SO_4, concentrated and purified by column chromatography using ethyl acetate

in hexanes from 0% to 100% to obtain **Boc-*d*Tle-Pro-Aib-Phe-OMe** as a white solid (7.20 g, 12.5 mmol, 51% yield over 5 steps). Purity of the peptide was assessed by UPLC.

Deprotection: Crude **Boc-*d*Tle-Pro-Aib-Phe-OMe** (1.15 g, 2.0 mmol, 1.0 equiv) was placed in a round-bottom flask and 4 M HCl in dioxane was added (3 mL, 148 mmol, 6.0 equiv). The mixture was stirred for 1 h in room temperature, and then dioxane was evaporated on the rotary evaporator. The crude product was dried *in vacuo* and promptly used in the next step without further purification.

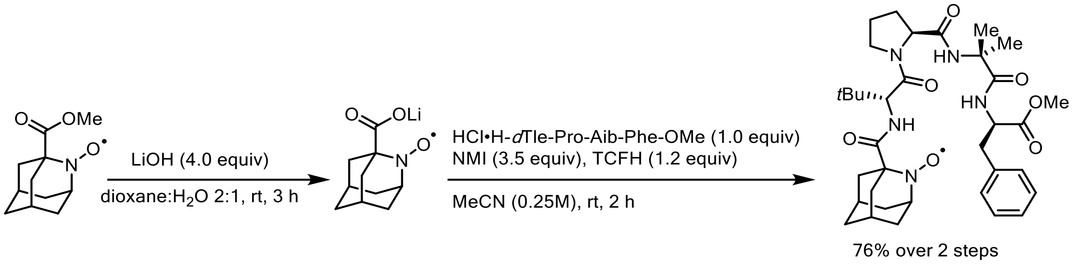

To a solution of **Azc-OMe** (105 mg, 0.5 mmol, 1.0 equiv) in dioxane (900 μL) was added a solution of LiOH•H_2O (83.9 mg, 2.0 mmol, 4.0 equiv) in H_2O (600 μL). The heterogeneous red mixture was stirred vigorously for 3 h before the addition of trimethylamine hydrochloride (139 mg, 1.45 mmol, 2.9 equiv). Solvent was evaporated on the rotary evaporator (bath temperature 30 °C). The oil was dried azeotropically via repeated concentration from acetonitrile and dried *in vacuo* to yield lithium salt AzcH-OLi as an orange solid that was used in the next step without further purification.

The round-bottom flask was charged with crude **AzcH-OLi** (0.5 mmol, 1 equiv), **H-*d*Tle-Pro-Aib-Phe-OMe** (256 mg, 0.5 mmol, 1.0 equiv), and *N*-methylimidazole (144 mg, 1.75 mmol, 3.5 equiv). MeCN (2 mL) was added, followed by TCFH (168 mg, 0.6 mmol, 1.2 equiv). Mixture was stirred for 2 h and then transferred to separatory funnel. H_2O was added (20 mL), and the mixture was extracted with DCM (3 × 20 mL). Combined organic phases were dried over Na_2SO_4 and concentrated on the rotary evaporator. Crude product was purified by column chromatography using a gradient of EtOAc in DCM from 0% to 100% to obtain **P2** as an off-red solid (249 mg, 0.38 mmol, 76% yield). HRMS (DART/Orbitrap) calculated for $[M+H]^+$ ($C_{35}H_{52}N_5O_7$) m/z 654.3861; found m/z 654.3840. Catalysts were isolated as a mixture of the hydroxylamine and aminoxyl form.

Catalysts can also be purified using reverse-phase HPLC (preparative C18 column) using a MeCN:H_2O mobile phase, however we observed significantly lower yields using that approach.

3. Reaction Optimization:

3.1. General Procedure for Microscale Screening

1) The corresponding **substrate** was dissolved in DCE to obtain 0.1 M stock solution.
2) The corresponding **catalyst** was dissolved in DCE to obtain a 0.05M stock solution.

3) ***m*CPBA** was dissolved in DCE to obtain a 0.375M stock solution. Then, the solution was filtered through a plug of anhydrous $MgSO_4$.
4) 1 mL glass vials with stir bars were placed in 24- or 96-position reaction block.
5) Vials were charged consecutively with stock solutions of **substrate** (50 µL, 5 µmol, 1.0 equiv), **catalyst** (10 µL, 0.5 µmol, 0.1 equiv), and ***m*CPBA** (40 µL, 15 µmol, 3.0 equiv).
6) Reaction block was sealed, and the reaction mixtures were stirred for 16 h at room temperature.
7) After that time, the reaction mixtures were diluted with *i*PrOH and transferred to GC vials for UPLC or SFC analysis to determine the enantiomeric excess.

3.2. Initial Protecting Group Optimization

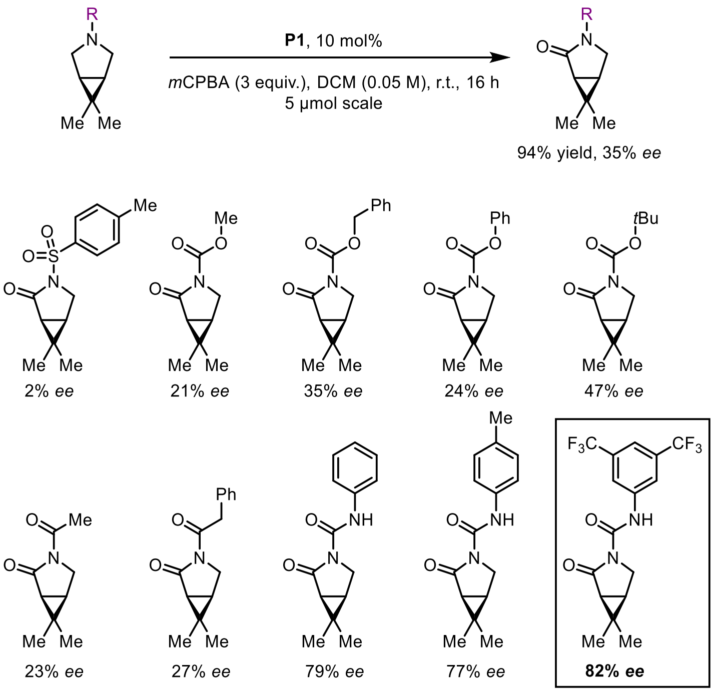

During the initial screening of protecting groups using high-throughput experimentation, only the enantiomeric excess of the reaction was analyzed. Initial protecting group screening revealed that ureas are an especially privileged protecting group, providing significantly higher *%ee* than amides, sulfonamides and carbamates tested. We selected 3,5-difluoromethylphenyl urea as a model starting material for further studies.

3.3. Catalyst Optimization and Characterization

All catalyst screening reactions were performed according to the general procedure outlined in section 3.1 on 5 µmol scale.

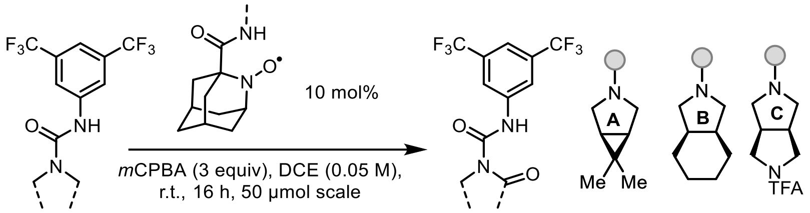

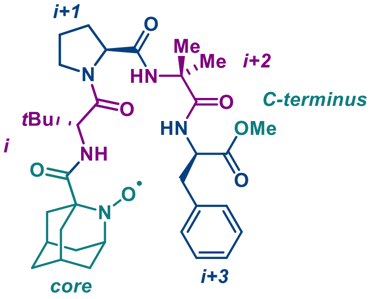

	core	*i*	*i+1*	*i+2*	*i+3*	*C-terminus*	%*ee* A	B	C	average
**P1**	AzcH	d Tle	Pro	Aib	-	OMe	76	82	42	67
**P2**	AzcH	d Tle	Pro	Aib	Phe	OMe	77	94	89	87
**P3**	O-Azc	d Tle	Pro	Aib	Phe	OMe	53	68	78	66
**P4**	Nor	d Tle	Pro	Aib	Phe	OMe	74	91	92	86
**P5**	AzcH	d Adam	Pro	Aib	Phe	OMe	83	93	89	88
**P6**	AzcH	d Phg	Pro	Aib	Phe	OMe	15	83	88	62
**P7**	AzcH	d Cha	Pro	Aib	Phe	OMe	48	57	71	59
**P8**	AzcH	d Chg	Pro	Aib	Phe	OMe	28	86	78	64
**P9**	AzcH	d Tle	Pro-Cyp	Aib	Phe	OMe	82	85	82	83
**P10**	AzcH	d Tle	Pip	Aib	Phe	OMe	89	88	59	79
**P11**	AzcH	d Tle	Azt	Aib	Phe	OMe	81	88	73	81
**P12**	AzcH	d Tle	Pro	Acpc	Phe	OMe	88	68	59	72
**P13**	AzcH	d Tle	Pro	Achc	Phe	OMe	36	40	49	42
**P14**	AzcH	d Tle	Pro	Acbc	Phe	OMe	78	62	64	68
**P15**	AzcH	d Tle	Pro	Aib	Bip	OMe	71	76	56	68
**P16**	AzcH	d Tle	Pro	Aib	γ-Me-Leu	OMe	66	86	70	74
**P17**	AzcH	d Tle	Pro	Aib	d Phe	OMe	73	81	66	73
**P18**	AzcH	d Tle	Pro	Aib	Phe	$NHCH_2CF_3$	73	54	60	62
**P19**	AzcH	d Tle	Pro	Aib	Phe	NHMe	44	30	46	40

Catalyst synthesis was performed following the procedures from section “2. Catalyst Synthesis”. The structure of the catalyst was confirmed by HRMS (DART/Orbitrap).

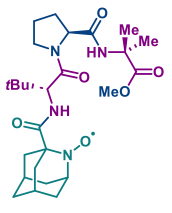

P1
$[M+2H]^+ = [C_{26}H_{43}N_4O_6]^+$
Requires: 507.3172
Found: 507.3163

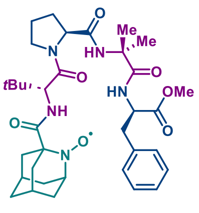

P2
$[M+2H]^+ = [C_{35}H_{52}N_5O_7]^+$
Requires: 654.3856
Found: 654.3840

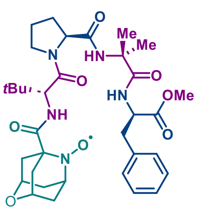

P3
$[M+2H]^+ = [C_{34}H_{50}N_5O_8]^+$
Requires: 656.3648
Found: 656.3630

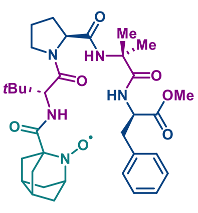

P4
$[M+2H]^+ = [C_{34}H_{50}N_5O_7]^+$
Requires: 640.3699
Found: 640.3692

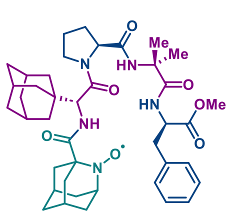

P5
$[M+2H]^+ = [C_{41}H_{58}N_5O_7]^+$
Requires: 732.4325
Found: 732.4302

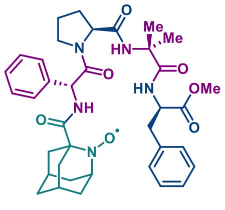

P6
$[M+2H]^+ = [C_{37}H_{48}N_5O_7]^+$
Requires: 674.3543
Found: 674.3544

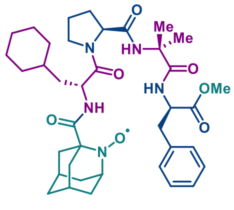

P7
$[M+2H]^+ = [C_{38}H_{56}N_5O_7]^+$
Requires: 694.4169
Found: 694.4167

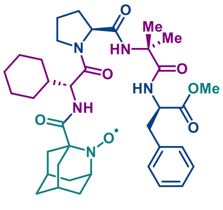

P8
$[M+2H]^+ = [C_{37}H_{54}N_5O_7]^+$
Requires: 680.4012
Found: 680.4000

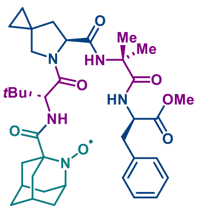

P9
$[M+2H]^+ = [C_{37}H_{54}N_5O_7]^+$
Requires: 680.4012
Found: 668.4000

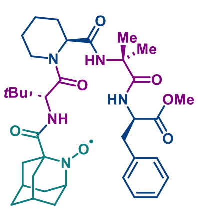

P10
$[M+2H]^+ = [C_{36}H_{54}N_5O_7]^+$
Requires: 668.4012
Found: 668.3989

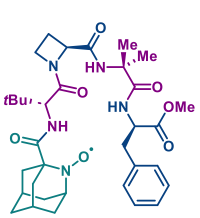

P11
$[M+2H]^+ = [C_{34}H_{50}N_5O_7]^+$
Requires: 640.3699
Found: 640.3687

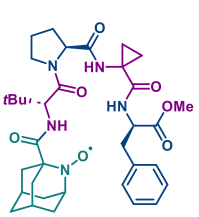

P12
$[M+2H]^+ = [C_{35}H_{50}N_5O_7]^+$
Requires: 652.3699
Found: 652.3690

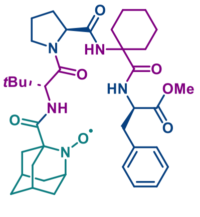

P13
$[M+2H]^+ = [C_{38}H_{56}N_5O_7]^+$
Requires: 694.4169
Found: 694.4159

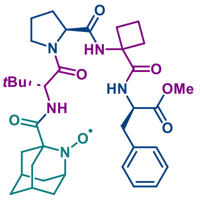

P14
$[M+2H]^+ = [C_{36}H_{52}N_5O_7]^+$
Requires: 666.3856
Found: 666.3848

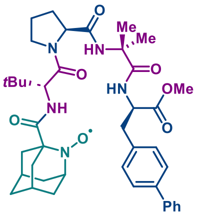

P15
$[M+2H]^+ = [C_{41}H_{56}N_5O_7]^+$
Requires: 730.4169
Found: 730.4153

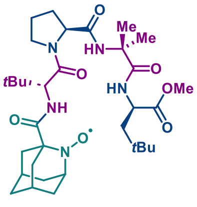

P16
$[M+2H]^+ = [C_{33}H_{56}N_5O_7]^+$
Requires: 634.4169
Found: 634.4146

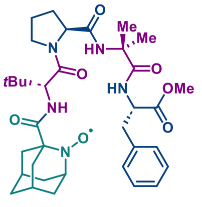

P17
$[M+2H]^+ = [C_{35}H_{52}N_5O_7]^+$
Requires: 654.3856
Found: 654.3838

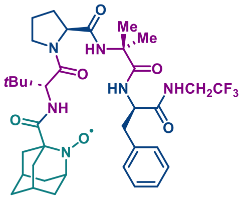

P18
$[M+2H]^+ = [C_{36}H_{52}F_3N_6O_6]^+$
Requires: 721.3889
Found: 721.3875

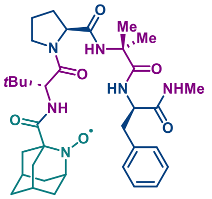

P19
$[M+2H]^+ = [C_{35}H_{53}N_6O_6]^+$
Requires: 653.4016
Found: 653.4001

3.4. Reaction Optimization at 0.1 mmol Scale

solvent	*oxidant*	*temp.*	*catalyst loading*	*additive*	*yield*	*%ee*
DCE (0.05 M)	*m*CPBA	r.t.	1 mol%	-	88	85
DCM (0.05 M)	*m*CPBA	r.t.	1 mol%	-	90	79
$CHCl_3$ (0.05 M)	*m*CPBA	r.t.	1 mol%	-	87	45
$CDCl_3$ (0.05 M)	*m*CPBA	r.t.	1 mol%	-	88	42
$PhCF_3$ (0.05 M)	*m*CPBA	r.t.	1 mol%	-	90	77
PhH (0.05 M)	*m*CPBA	r.t.	1 mol%	-	83	30
PhF (0.05 M)	*m*CPBA	r.t.	1 mol%	-	91	51
MeCN (0.05 M)	*m*CPBA	r.t.	1 mol%	-	89	53
EtOAc (0.05 M)	*m*CPBA	r.t.	1 mol%	-	61	35
DCE (0.05 M)	MMPP	r.t.	1 mol%	-	4	53
DCE (0.05 M)	*m*CPBA	r.t.	1 mol%	$HNTf_2$ (0.5 mol%)	87	85
DCE (0.05 M)	*m*CPBA	r.t.	1 mol%	TFA (1.0 eq.)	92	81
DCE (0.05 M)	*m*CPBA	r.t.	1 mol%	$NaSbF_6$ (20 mol%)	88	85
DCE (0.05 M)	*m*CPBA	r.t.	1 mol%	$NaSbF_6$ (100 mol%)	66	73
DCE (0.05 M)	*m*CPBA	r.t.	0.1 mol%	-	54	10
DCE (0.025 M)	*m*CPBA	r.t.	2 mol%	-	88	88
DCE (0.025 M)	*m*CPBA	r.t.	1 mol%	$HNTf_2$ (0.5 mol%)	88	89
DCE (0.025 M)	*m*CPBA	r.t.	2 mol%	$HNTf_2$ (1 mol%)	84	89
DCE (0.05 M)	*m*CPBA	40 °C	1 mol%	-	87	82
DCE (0.05 M)	*m*CPBA	0 °C, 1h	1 mol%	-	17	80
DCE (0.1 M)	*m*CPBA	r.t.	1 mol%	-	90	80
DCE (0.05 M)	*m*CPBA	r.t.	1 mol%	-	89	85
DCE (0.025 M)	*m*CPBA	r.t.	1 mol%	-	90	87
DCE (0.0125 M)	*m*CPBA	r.t.	1 mol%	-	94	89
DCE (0.01 M)	*m*CPBA	r.t.	1 mol%	-	90	90
DCE (0.005 M)	*m*CPBA	r.t.	1 mol%	-	81	91
DCE (0.01 M)	*m*CPBA	5 °C	1 mol%	-	56	90
DCE (0.01 M)	*m*CPBA	5 °C	1 mol%	$HNTf_2$ (0.5 mol%)	79	92
DCE (0.01 M)	*m*CPBA	–25 °C	1 mol%	-	46	89
**DCE (0.01 M)**	***m*CPBA**	**r.t.**	**1 mol%**	**$HNTf_2$ (0.5 mol%)**	**89**	**92**

3.5. Non-Pyrrolidine *meso*-Heterocylces

During optimization, we also attempted the desymmetrization of a morpholine (**E**), and two bridged bicyclic [3.2.1] azepanes (**D**, **F**). The substrates were screened against various catalysts and conditions. The reactions were monitored by UPLC-MS at 254 nm, where the 3,5-bis(trifluroromethyl)phenyl-urea has a strong characteristic absorption, allowing for accurate estimation of amount based on the area percentage (LCAP) of a given peak. The %ee of the crude reaction samples was determined by chiral HPLC, with ESI-MS areas of the molecular ion peak correlating well with the UV trace (supporting the purity of the peaks used for %ee determination[1]). The substrates showed sluggish conversion to the desired lactams under standard reaction conditions, delivering >60% recovered starting material under most reaction conditions. With more forcing conditions (higher concentration with higher *m*CPBA and catalyst loadings, or higher temperatures), the conversion could be increased. However, this resulted in messy reaction profiles with multiple side products. Despite the high crude enantioselectivities (not validated by isolation) for the oxidized products, optimization of yield proofed challenging with yields remaining below 20–30%. Azetidine (**G**), piperidine (**H**, **I**, **J**) and piperazine (**K**) proved to be even more challenging substrates which did not provide quantifiable oxidation products. This prompted us to fully focus our optimization efforts and scope studies, on pyrrolidines within which we rapidly identified remarkably general conditions with high yields and enantioselectivities all pyrrolidines tested.

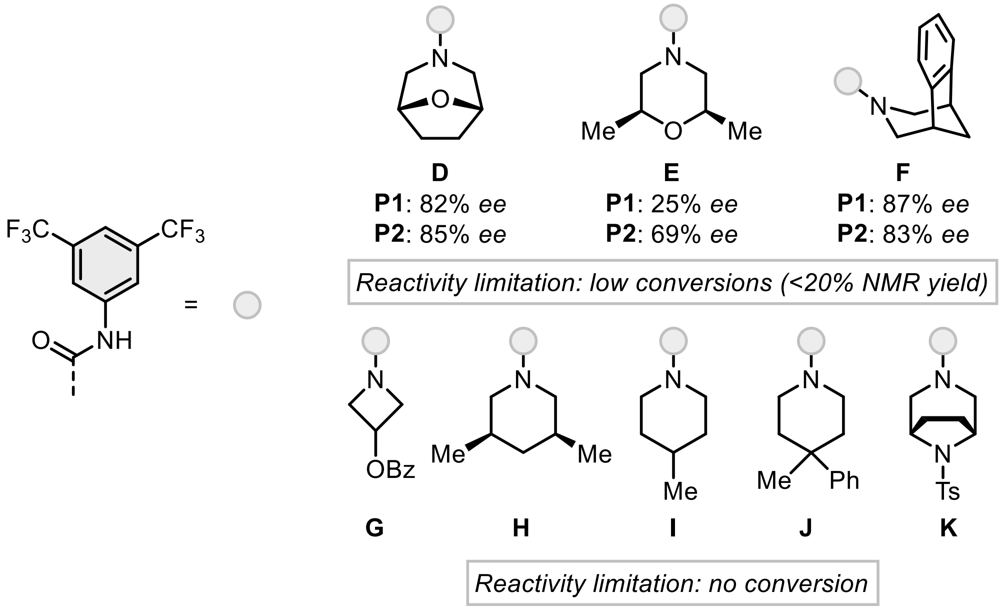

4. Substrate Scope

GP-A: The substrate (0.1 mmol, 1 equiv), **catalyst (P2)** (0.65 mg, 0.001 mmol, 1 mol%), and 10 mL of DCE were added to a 20-mL vial charged with a magnetic stir bar. $HNTf_2$ (0.14 mg, 0.5 mol%, 20 µL of a 0.025 M stock solution in DCE) followed by *m*CPBA (67.2 mg, 0.3 mmol, 3 equiv, 77% purity) were then added to the vial. The solution was stirred for 18 h. 5 mL of saturated Na_2SO_3 solution was added and stirred for 20 min. The mixture was diluted with 50 mL of EtOAc and washed with 50 mL of saturated Na_2SO_3 solution, 50 mL saturated sodium bicarbonate solution, and brine (50 mL), dried over anhydrous $MgSO_4$, and concentrated. The crude product was purified by flash silica chromatography gradient.

GP-B: The substrate (0.1 mmol, 1 equiv), **catalyst (P2)** (3.3 mg, 0.005 mmol, 5 mol%), and 10 mL of DCE were added to a 20-mL vial charged with a magnetic stir bar. $HNTf_2$ (0.70 mg, 2.5 mol%, 100 uL of a 0.025 M stock solution in DCE) followed by *m*CPBA (67.2 mg, 0.3 mmol, 3 equiv, 77% purity) were then added to the vial. The solution was stirred for 18 h. 5 mL of saturated Na_2SO_3 solution was added and stirred for 20 min. The mixture was diluted with 50 mL of EtOAc and washed with 50 mL of saturated Na_2SO_3 solution, 50 mL saturated sodium bicarbonate solution, and brine (50 mL), dried over anhydrous $MgSO_4$, and concentrated. The crude product was purified by flash silica chromatography gradient.

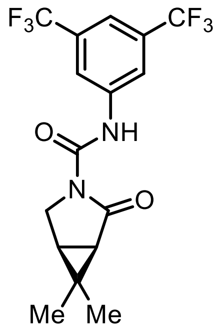

(1*R*,5*S*)-*N*-(3,5-bis(trifluoromethyl)phenyl)-6,6-dimethyl-2-oxo-3-azabicyclo[3.1.0]hexane-3-carboxamide (5) Prepared from (1*r*,5*s*)-*N*-(3,5-bis(trifluoromethyl)phenyl)-6,6-dimethyl-3-azabicyclo[3.1.0]hexane-3-carboxamide (36.6 mg, 0.1 mmol, 1.0 equiv) following GP-A. The concentrated crude product was then loaded onto a SiO_2 column and eluted with a gradient from 0% hexanes/DCM to 100% hexanes/DCM. The product was obtained as a white solid (31.9 mg, 0.084 mmol, 84% yield). $[\alpha]_D^{20}$ −46 (*c* 1.0, $CHCl_3$). ^1H NMR (500 MHz, $CDCl_3$) δ 10.91 (s, 1H), 8.02 (s, 2H), 7.57 (s, 1H), 3.97 (dd, *J* = 12.2, 6.7 Hz, 1H), 3.84 (dt, *J* = 12.2, 1.4 Hz, 1H), 2.05 (dd, *J* = 6.3, 1.7 Hz, 1H), 1.87 (t, *J* = 6.5 Hz, 1H), 1.22 (s, 3H), 1.14 (s, 3H). ^{13}C NMR (126 MHz, $CDCl_3$) δ 175.5, 150.1, 139.2, 132.5 (q, *J* = 33.4 Hz), 123.3 (q, *J* = 272.8 Hz), 119.8 – 119.6 (m), 117.4 – 117.2 (m), 44.7, 35.0, 26.0, 24.5, 14.2. ^{19}F NMR (470 MHz, $CDCl_3$) δ -63.05. HRMS (DART/Orbitrap) calculated for $[M+H]^+$ ($C_{16}H_{15}F_6N_2O_2$) m/z 381.1032; found m/z 381.1024.

Large scale preparation:

(1*r*,5*s*)-*N*-(3,5-bis(trifluoromethyl)phenyl)-6,6-dimethyl-3-azabicyclo[3.1.0]hexane-3-carboxamide (733 mg, 2.0 mmol, 1 equiv), **catalyst (P2)** (13.1 mg, 0.02 mmol, 1 mol%), and 200 mL of DCE were added to a 20-mL vial charged with a magnetic stir bar. $HNTf_2$ (0.70 mg, 0.25 mol%, 200 uL of a 0.025M stock solution in DCE) followed by *m*CPBA (1.34 g, 6.0 mmol, 3

equiv, 77% purity) were then added to the vial. The solution was stirred for 18 h. 200 mL of saturated Na_2SO_3 solution was added and stirred for 20 min. The mixture was diluted with 200 mL of EtOAc and the phases were separated. The organic phase was washed with 200 mL of saturated sodium bicarbonate solution, 200 mL of brine, dried over anhydrous $MgSO_4$, and concentrated. The crude product was crystalized from DCM/hexanes mixture. The product was obtained as off-white thin needles (641 mg, 1.69 mmol, 84% yield, 98 *%ee*).

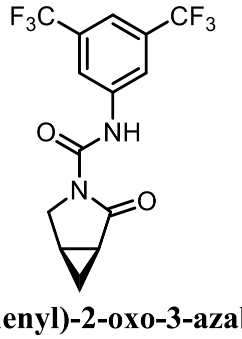

(1*S*,5*R*)-*N*-(3,5-bis(trifluoromethyl)phenyl)-2-oxo-3-azabicyclo[3.1.0]hexane-3-carboxamide (7) Prepared from (1r,5s)-N-(3,5-bis(trifluoromethyl)phenyl)-3-azabicyclo[3.1.0]hexane-3-carboxamide (33.8 mg, 0.1 mmol, 1.0 equiv) following GP-A. The concentrated crude product was then loaded onto a SiO_2 column and eluted with a gradient from 0% hexanes/DCM to 100% hexanes/DCM. The product was obtained as a white solid (33.9 mg, 0.096 mmol, 96% yield). $[\alpha]_D^{20}$ −19 (*c* 1.0, $CHCl_3$). ^1H NMR (500 MHz, $CDCl_3$) δ 10.80 (s, 1H), 8.00 (s, 2H), 7.57 (s, 1H), 4.01 – 3.92 (m, 2H), 2.23 – 2.17 (m, 1H), 2.12 – 2.06 (m, 1H), 1.37 (ddd, *J* = 8.9, 7.6, 5.1 Hz, 1H), 0.89 – 0.83 (m, 1H). ^{13}C NMR (126 MHz, $CDCl_3$) δ 177.3, 150.6, 139.2, 132.5 (q, *J* = 33.5 Hz), 123.2 (q, *J* = 272.7 Hz), 119.8 – 119.6 (m), 117.5 – 117.2 (m), 47.7, 22.4, 13.6, 12.2. ^{19}F NMR (470 MHz, $CDCl_3$) δ -63.06. HRMS (DART/Orbitrap) calculated for $[M+H]^+$ ($C_{14}H_{11}F_6N_2O_2$) m/z 353.0719; found m/z 353.0711.

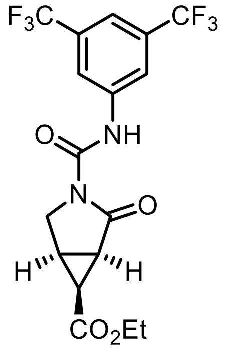

ethyl (1*R*,5*S*,6*S*)-3-((3,5-bis(trifluoromethyl)phenyl)carbamoyl)-2-oxo-3-azabicyclo[3.1.0]hexane-6-carboxylate (8) Prepared from ethyl (1*R*,5*S*,6*s*)-3-((3,5-bis(trifluoromethyl)phenyl)carbamoyl)-3-azabicyclo[3.1.0]hexane-6-carboxylate (41.0 mg, 0.1 mmol, 1.0 equiv) following GP-A. The concentrated crude product was then loaded onto a SiO_2 column and eluted with a gradient from 0% hexanes/DCM to 100% hexanes/DCM, then DCM/AcOEt 0% to DCM/AcOEt 20%. The product was obtained as a white solid (26.9 mg, 0.063 mmol, 63% yield). $[\alpha]_D^{20}$ −50 (*c* 1.0, $CHCl_3$). ^1H NMR (500 MHz, $CDCl_3$) δ 10.63 (s, 1H), 7.99 (s, 2H), 7.59 (s, 1H), 4.20 (q, *J* = 7.1 Hz, 2H), 4.11 – 4.01 (m, 2H), 2.72 – 2.69 (m, 1H), 2.57 – 2.53 (m, 1H), 1.98 – 1.96 (m, 1H), 1.30 (t, *J* = 7.1 Hz, 3H). ^{13}C NMR (126 MHz, $CDCl_3$) δ 174.2, 169.5, 149.9, 138.8, 132.6 (q, *J* = 33.5 Hz), 123.2 (q, *J* = 272.8 Hz), 119.9 – 119.7 (m), 117.7 –

117.6 (m), 62.0, 47.5, 30.5, 26.7, 20.1, 14.3. ^{19}F NMR (470 MHz, $CDCl_3$) δ -63.06. HRMS (DART/Orbitrap) calculated for $[M+H]^+$ ($C_{17}H_{15}F_6N_2O_4$) m/z 425.0931; found m/z 425.0935.

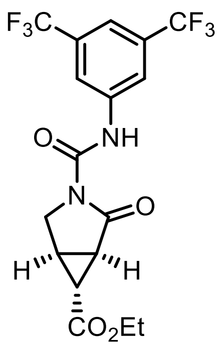

ethyl (1*R*,5*S*,6*R*)-3-((3,5-bis(trifluoromethyl)phenyl)carbamoyl)-2-oxo-3-azabicyclo[3.1.0]hexane-6-carboxylate (9) Prepared from ethyl (1*R*,5*S*,6*r*)-3-((3,5-bis(trifluoromethyl)phenyl)carbamoyl)-3-azabicyclo[3.1.0]hexane-6-carboxylate (41.0 mg, 0.1 mmol, 1.0 equiv) following GP-A. The concentrated crude product was then loaded onto a SiO_2 column and eluted with a gradient from 0% hexanes/DCM to 100% hexanes/DCM, then DCM/AcOEt 0% to DCM/AcOEt 20%. The product was obtained as a white solid (26.9 mg, 0.083 mmol, 83% yield). $[\alpha]_D^{20}$ −21 (*c* 1.0, $CHCl_3$). ^{1}H NMR (500 MHz, $CDCl_3$) δ 10.75 (s, 1H), 8.04 (s, 2H), 7.57 (s, 1H), 4.20 – 4.11 (m, 3H), 4.09 – 4.05 (m, 1H), 2.67 (ddd, *J* = 8.3, 6.4, 1.5 Hz, 1H), 2.49 – 2.43 (m, 1H), 2.30 (t, *J* = 8.4 Hz, 1H), 1.24 (t, *J* = 7.1 Hz, 3H). ^{13}C NMR (126 MHz, $CDCl_3$) δ 173.1, 168.1, 149.7, 139.2, 132.4 (q, *J* = 33.4 Hz), 123.3 (q, *J* = 272.8 Hz), 119.7 (q, *J* = 3.9 Hz), 117.3 (hept, *J* = 3.2 Hz), 61.8, 44.8, 29.5, 25.0, 20.0, 14.2. ^{19}F NMR (470 MHz, $CDCl_3$) δ -63.05. HRMS (DART/Orbitrap) calculated for $[M+H]^+$ ($C_{17}H_{15}F_6N_2O_4$) m/z 425.0931; found m/z 425.0934.

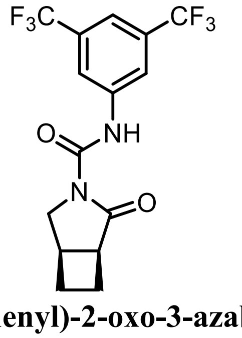

(1*S*,5*R*)-N-(3,5-bis(trifluoromethyl)phenyl)-2-oxo-3-azabicyclo[3.2.0]heptane-3-carboxamide (10) Prepared from (1*R*,5*S*)-N-(3,5-bis(trifluoromethyl)phenyl)-3-azabicyclo[3.2.0]heptane-3-carboxamide (35.2 mg, 0.1 mmol, 1.0 equiv) following GP-A. The concentrated crude product was then loaded onto a SiO_2 column and eluted with a gradient from 0% hexanes/DCM to 100% hexanes/DCM. The product was obtained as a white solid (22.6 mg, 0.062 mmol, 62% yield). $[\alpha]_D^{20}$ +64 (*c* 1.0, $CHCl_3$). ^{1}H NMR (500 MHz, $CDCl_3$) δ 11.17 (s, 1H), 8.06 (s, 2H), 7.60 (s, 1H), 3.96 (dd, *J* = 11.6, 7.2 Hz, 1H), 3.90 (d, *J* = 11.7 Hz, 1H), 3.37 – 3.31 (m, 1H), 3.06 (p, *J* = 7.4 Hz, 1H), 2.68 – 2.59 (m, 1H), 2.51 – 2.41 (m, 1H), 2.23 – 2.16 (m, 1H), 2.13 – 2.03 (m, 1H). ^{13}C NMR (126 MHz, $CDCl_3$) δ 180.9, 150.8, 139.2, 132.5 (q, *J* = 33.5 Hz), 123.3 (q, *J* = 272.8 Hz), 119.8 (q, *J* = 3.9 Hz), 117.5 – 117.3 (m), 52.5, 43.8, 28.5, 26.4, 24.4. ^{19}F NMR (470 MHz, $CDCl_3$) δ -63.04. HRMS (DART/Orbitrap) calculated for $[M+H]^+$ ($C_{15}H_{13}F_6N_2O_2$) m/z 367.0876; found m/z 367.0882.

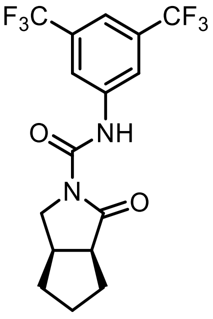

(3a*R*,6a*S*)-*N*-(3,5-bis(trifluoromethyl)phenyl)-1-oxohexahydrocyclopenta[c]pyrrole-2(1*H*)-carboxamide (11) Prepared from (3a*R*,6a*S*)-*N*-(3,5-bis(trifluoromethyl)phenyl)hexahydrocyclopenta[*c*]pyrrole-2(1*H*)-carboxamide (36.6 mg, 0.1 mmol, 1.0 equiv) following GP-B using 5 mol% of the catalyst and 2.5 mol% of $HNTf_2$. The concentrated crude product was then loaded onto a SiO_2 column and eluted with a gradient from 0% hexanes/DCM to 100% hexanes/DCM. The product was obtained as a white solid (30.9 mg, 0.081 mmol, 81% yield). $[\alpha]_D^{20}$ +62 (*c* 1.0, $CHCl_3$). ^1H NMR (500 MHz, $CDCl_3$) δ 11.09 (s, 1H), 8.03 (s, 2H), 7.58 (s, 1H), 4.06 (dd, *J* = 11.6, 8.6 Hz, 1H), 3.67 (dd, *J* = 11.6, 2.9 Hz, 1H), 3.22 (td, *J* = 9.3, 3.3 Hz, 1H), 2.82 – 2.73 (m, 1H), 2.12 – 1.94 (m, 3H), 1.78 – 1.68 (m, 1H), 1.66 – 1.52 (m, 3H). ^{13}C NMR (126 MHz, $CDCl_3$) δ 180.8, 150.3, 139.2, 132.5 (q, *J* = 33.5 Hz), 123.2 (q, *J* = 272.8 Hz), 119.8 (q, *J* = 2.8 Hz), 117.5 – 117.3 (m), 51.3, 49.7, 34.4, 33.5, 30.7, 25.7. ^{19}F NMR (470 MHz, $CDCl_3$) δ -63.05. HRMS (DART/Orbitrap) calculated for $[M+H]^+$ ($C_{16}H_{15}F_6N_2O_2$) m/z 381.1032; found m/z 381.1027.

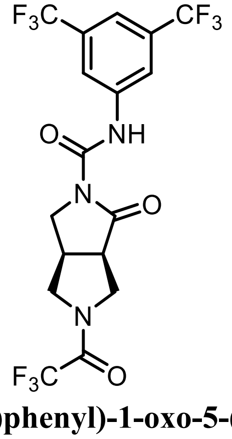

(3a*S*,6a*R*)-*N*-(3,5-bis(trifluoromethyl)phenyl)-1-oxo-5-(2,2,2-trifluoroacetyl)hexahydropyrrolo[3,4-*c*]pyrrole-2(1*H*)-carboxamide (12) Prepared from (3a*R*,6a*S*)-*N*-(3,5-bis(trifluoromethyl)phenyl)-5-(2,2,2-trifluoroacetyl)hexahydropyrrolo[3,4-c]pyrrole-2(1*H*)-carboxamide (46.3 mg, 0.1 mmol, 1.0 equiv) following GP-B using 5 mol% of the catalyst and 2.5 mol% of $HNTf_2$. The concentrated crude product was then loaded onto a SiO_2 column and eluted with a gradient from 0% hexanes/DCM to 100% hexanes/DCM, then DCM/AcOEt 0% to DCM/AcOEt 20%. The product was obtained as a white solid (36.3 mg, 0.076 mmol, 76% yield). $[\alpha]_D^{20}$ −22 (*c* 1.0, $CHCl_3$). ^1H NMR (500 MHz, $CDCl_3$, rotamers) ^1H NMR (500 MHz, $CDCl_3$) δ 10.72 (s, 0.5H), 10.69 (s, 0.5H), 8.02 (s, 2H), 7.62 (s, 1H), 4.29 (d, *J* = 11.8 Hz, 0.5H), 4.23 – 4.12 (m, 1.5H), 4.10 – 4.04 (m, 1H), 4.00 (dd, *J* = 11.9, 1.4 Hz, 0.5H), 3.97 – 3.88 (m, 1.5H), 3.55 (td, *J* = 8.0, 1.7 Hz, 0.5H), 3.53 – 3.42 (m, 1.5H), 3.31 – 3.23 (m, 0.5H), 3.20 – 3.11 (m, 0.5H). ^{13}C NMR (126 MHz, $CDCl_3$,

rotamers) δ 176.1, 175.9, 155.5 (q, J = 37.7 Hz), 155.4 (q, J = 37.6 Hz), 149.6, 149.5, 138.6, 132.7 (q, J = 33.6 Hz), 123.1 (q, J = 273.2 Hz), 112.0, 118.2 – 117.2 (m), 116.1 (q, J = 286.4 Hz), 52.2, 50.7 (q, J = 3.2 Hz), 49.5, 49.0, 48.6, 48.0 (q, J = 3.9 Hz), 47.8, 46.6, 34.5, 31.5. ^{19}F NMR (470 MHz, $CDCl_3$, rotamers) δ -63.06, -72.35, -72.36. HRMS (DART/Orbitrap) calculated for $[M+H]^+$ ($C_{17}H_{13}F_9N_3O_3$) m/z 478.0808; found m/z 478.0804.

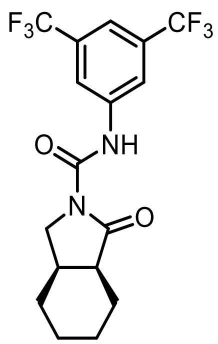

(3a*R*,7a*S*)-*N*-(3,5-bis(trifluoromethyl)phenyl)-1-oxooctahydro-2*H*-isoindole-2-carboxamide (13) Prepared from (3a*R*,7a*S*)-*N*-(3,5-bis(trifluoromethyl)phenyl)octahydro-2*H*-isoindole-2-carboxamide (38.0 mg, 0.1 mmol, 1.0 equiv) following GP-B using 5 mol% of the catalyst and 2.5 mol% of $HNTf_2$. The concentrated crude product was then loaded onto a SiO_2 column and eluted with a gradient from 0% hexanes/DCM to 100% hexanes/DCM. The product was obtained as a white solid (33.2 mg, 0.084 mmol, 84% yield). $[\alpha]_D^{20}$ +4 (*c* 1.0, $CHCl_3$). ^{1}H NMR (500 MHz, $CDCl_3$) δ 10.98 (s, 1H), 8.04 (s, 2H), 7.58 (s, 1H), 3.75 (dd, J = 10.9, 5.7 Hz, 1H), 3.68 (dd, J = 11.0, 2.0 Hz, 1H), 2.82 (td, J = 6.5, 3.6 Hz, 1H), 2.46 – 2.38 (m, 1H), 2.16 – 2.08 (m, 1H), 1.87 – 1.79 (m, 1H), 1.70 – 1.56 (m, 3H), 1.33 – 1.14 (m, 3H). ^{13}C NMR (126 MHz, $CDCl_3$) δ 179.1, 150.8, 139.3, 132.5 (q, J = 33.5 Hz), 123.3 (q, J = 272.7 Hz), 119.7 (q, J = 3.2 Hz), 117.4 – 117.1 (m), 49.6, 44.5, 31.3, 27.9, 23.5, 23.0, 22.8. ^{19}F NMR (470 MHz, $CDCl_3$) δ -63.07. HRMS (DART/Orbitrap) calculated for $[M+H]^+$ ($C_{17}H_{17}F_6N_2O_2$) m/z 395.1189; found m/z 395.1184.

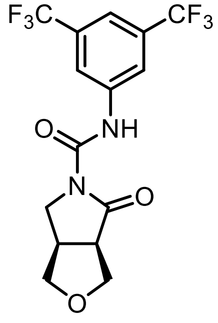

(3a*R*,6a*R*)-*N*-(3,5-bis(trifluoromethyl)phenyl)-4-oxotetrahydro-1*H*-furo[3,4-*c*]pyrrole-5(3*H*)-carboxamide (14) Prepared from (3a*R*,6a*S*)-*N*-(3,5-bis(trifluoromethyl)phenyl)tetrahydro-1*H*-furo[3,4-*c*]pyrrole-5(3*H*)-carboxamide (36.8 mg, 0.1 mmol, 1.0 equiv) following GP-B using 5 mol% of the catalyst and 2.5 mol% of $HNTf_2$. The concentrated crude product was then loaded onto a SiO_2 column and eluted with a gradient from 0% hexanes/DCM to 100% hexanes/DCM, then DCM/AcOEt 0% to DCM/ACOEt 20%. The product was obtained as a white solid (27.6 mg, 0.072 mmol, 72% yield). $[\alpha]_D^{20}$ +40 (*c* 1.0, $CHCl_3$). ^{1}H NMR (500 MHz, $CDCl_3$) δ 10.92 (s, 1H), 8.02 (s, 2H), 7.60 (s, 1H), 4.31 (dd, J = 9.5, 2.0 Hz, 1H), 4.15 (dd, J = 11.7, 8.8 Hz, 1H), 3.94 – 3.78 (m, 4H), 3.46 (ddd, J = 9.3, 7.4, 2.0 Hz, 1H), 3.06 (tdt, J = 9.3, 6.5, 3.4 Hz, 1H). ^{13}C NMR

(126 MHz, $CDCl_3$) δ 178.0, 149.7, 138.9, 132.6 (q, *J* = 33.5 Hz), 123.2 (q, *J* = 272.9 Hz), 119.9 – 119.7 (m), 117.8 – 117.5 (m), 75.7, 71.6, 51.0, 50.6, 33.6. ^{19}F NMR (470 MHz, $CDCl_3$) δ -63.04. HRMS (DART/Orbitrap) calculated for $[M+H]^+$ ($C_{15}H_{17}F_6N_2O_2$) m/z 383.0825; found m/z 383.0821.

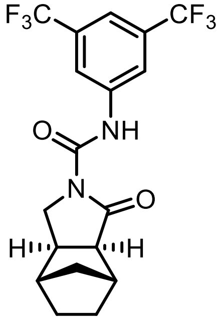

(3a*R*,4*S*,7*R*,7a*S*)-*N*-(3,5-bis(trifluoromethyl)phenyl)-1-oxooctahydro-2*H*-4,7-methanoisoindole-2-carboxamide (15) Prepared from (3a*R*,4*R*,7*S*,7a*S*)-*N*-(3,5-bis(trifluoromethyl)phenyl)octahydro-2*H*-4,7-methanoisoindole-2-carboxamide (39.2 mg, 0.1 mmol, 1.0 equiv) following GP-B using 5 mol% of the catalyst and 2.5 mol% of $HNTf_2$. The concentrated crude product was then loaded onto a SiO_2 column and eluted with a gradient from 0% hexanes/DCM to 100% hexanes/DCM. The product was obtained as a white solid (26.1 mg, 0.064 mmol, 64% yield). $[\alpha]_D^{20}$ +47 (*c* 1.0, $CHCl_3$). ^1H NMR (500 MHz, $CDCl_3$) δ 11.10 (s, 1H), 8.03 (s, 2H), 7.58 (s, 1H), 4.08 (dd, *J* = 11.8, 9.9 Hz, 1H), 3.51 (dd, *J* = 11.8, 3.6 Hz, 1H), 2.73 (d, *J* = 8.6 Hz, 1H), 2.68 (d, *J* = 4.2 Hz, 1H), 2.32 – 2.26 (m, 2H), 1.71 – 1.55 (m, 2H), 1.39 – 1.24 (m, 4H). ^{13}C NMR (126 MHz, $CDCl_3$) δ 179.3, 150.1, 139.2, 132.5 (q, *J* = 33.5 Hz), 123.2 (q, *J* = 272.8 Hz), 119.9 – 119.6 (m), 117.5 – 117.2 (m), 53.6, 51.3, 43.4, 41.1, 35.9, 33.5, 28.3, 27.7. ^{19}F NMR (470 MHz, $CDCl_3$) δ -63.05. HRMS (DART/Orbitrap) calculated for $[M+H]^+$ ($C_{18}H_{17}F_6N_2O_2$) m/z 407.1189; found m/z 407.1182.

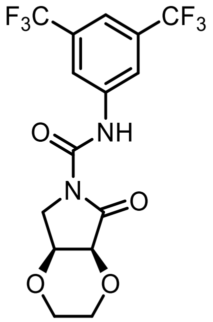

(4a*S*,7a*S*)-*N*-(3,5-bis(trifluoromethyl)phenyl)-5-oxohexahydro-6*H*-[1,4]dioxino[2,3-*c*]pyrrole-6-carboxamide (16) Prepared from (4a*R*,7a*S*)-*N*-(3,5-bis(trifluoromethyl)phenyl)hexahydro-6*H*-[1,4]dioxino[2,3-*c*]pyrrole-6-carboxamide (38.4 mg, 0.1 mmol, 1.0 equiv) following GP-B. The concentrated crude product was then loaded onto a SiO_2 column and eluted with a gradient from 0% hexanes/DCM to 100% hexanes/DCM, then DCM/AcOEt 0% to DCM/AcOEt 20%. The product was obtained as a white solid (17.0 mg, 0.043 mmol, 43% yield). $[\alpha]_D^{20}$ +25 (*c* 1.0, $CHCl_3$). ^1H NMR (500 MHz, $CDCl_3$) δ 10.66 (s, 1H), 8.04 (s, 2H), 7.62 (s, 1H), 4.52 (d, *J* = 4.0 Hz, 1H), 4.34 (t, *J* = 3.8 Hz, 1H), 4.04 (d, *J* = 12.6 Hz, 1H), 3.80 – 3.72 (m, 5H). ^{13}C NMR (126 MHz, $CDCl_3$) δ 173.5, 149.8, 138.8, 132.6 (q, *J* = 33.6 Hz), 124.3 (d, *J* = 273.0 Hz), 112.0 – 119.8 (m), 117.9 – 117.7 (m), 75.1, 67.4, 64.9, 63.1, 48.2. ^{19}F

NMR (470 MHz, $CDCl_3$) δ -63.04. HRMS (DART/Orbitrap) calculated for $[M+H]^+$ ($C_{15}H_{13}F_6N_2O_4$) m/z 399.0774; found m/z 399.0779.

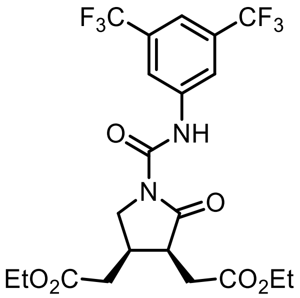

diethyl 2,2'-((3*S*,4*R*)-1-((3,5-bis(trifluoromethyl)phenyl)carbamoyl)-2-oxopyrrolidine-3,4-diyl)diacetate (17) Prepared from diethyl 2,2'-((3*R*,4*S*)-1-((3,5-bis(trifluoromethyl)phenyl)carbamoyl)pyrrolidine-3,4-diyl)diacetate (49.8 mg, 0.1 mmol, 1.0 equiv) following GP-B using 5 mol% of the catalyst and 2.5 mol% of $HNTf_2$. The concentrated crude product was then loaded onto a SiO_2 column and eluted with a gradient from 0% hexanes/DCM to 100% hexanes/DCM, then DCM/AcOEt 0% to DCM/AcOEt 20%. The product was obtained as a white solid (47.4 mg, 0.093 mmol, 93% yield). $[\alpha]_D^{20}$ +36 (*c* 1.0, $CHCl_3$). ^{1}H NMR (500 MHz, $CDCl_3$) δ 10.77 (s, 1H), 8.01 (s, 2H), 7.59 (s, 1H), 4.21 (qd, *J* = 7.2, 1.9 Hz, 2H), 4.15 (qd, *J* = 7.2, 0.9 Hz, 2H), 3.97 (dd, *J* = 11.7, 6.7 Hz, 1H), 3.85 (dd, *J* = 11.7, 2.5 Hz, 1H), 3.44 (td, *J* = 8.4, 5.6 Hz, 1H), 3.12 – 3.03 (m, 1H), 2.82 (dd, *J* = 17.1, 5.6 Hz, 1H), 2.54 (dd, *J* = 17.1, 8.5 Hz, 1H), 2.45 (dd, *J* = 16.4, 5.3 Hz, 1H), 2.31 (dd, *J* = 16.4, 9.2 Hz, 1H), 1.30 (t, *J* = 7.1 Hz, 3H), 1.26 (t, *J* = 7.1 Hz, 3H). ^{13}C NMR (126 MHz, $CDCl_3$) δ 177.2, 171.2, 171.1, 149.9, 139.0, 132.6 (q, *J* = 33.5 Hz), 123.2 (q, *J* = 272.8 Hz), 119.8 – 119.6 (m), 117.6 – 117.4 (m), 61.5, 61.4, 49.1, 44.1, 33.7, 30.7, 30.3, 14.3, 14.2. ^{19}F NMR (470 MHz, $CDCl_3$) δ -63.08. HRMS (DART/Orbitrap) calculated for $[M+H]^+$ ($C_{21}H_{23}F_6N_2O_6$) m/z 513.1455; found m/z 513.1450.

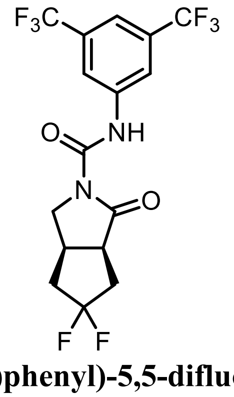

(3a*R*,6a*S*)-*N*-(3,5-bis(trifluoromethyl)phenyl)-5,5-difluoro-1-oxohexahydrocyclopenta[c]pyrrole-2(1*H*)-carboxamide (18) Prepared from (3a*R*,6a*S*)-*N*-(3,5-bis(trifluoromethyl)phenyl)-5,5-difluorohexahydrocyclopenta[*c*]pyrrole-2(1*H*)-carboxamide (40.2 mg, 0.1 mmol, 1.0 equiv) following GP-B using 5 mol% of the catalyst and 2.5 mol% of $HNTf_2$. The concentrated crude product was then loaded onto a SiO_2 column and eluted with a gradient from 0% hexanes/DCM to 100% hexanes/DCM. The product was obtained as a white solid (36.1 mg, 0.087 mmol, 87% yield). $[\alpha]_D^{20}$ +9 (*c* 1.0, $CHCl_3$). ^{1}H NMR (500 MHz, $CDCl_3$) δ 10.91 (s, 1H), 8.03 (s, 2H), 7.61 (s, 1H), 4.10 (dd, *J* = 11.8, 8.3 Hz, 1H), 3.85 (dd, *J* = 11.8, 2.3 Hz, 1H), 3.38 (td, *J* = 9.7, 3.2 Hz, 1H), 3.10 – 3.00 (m, 1H), 2.62 – 2.44 (m, 3H), 2.13 – 2.01 (m, 1H). ^{13}C NMR (126 MHz, $CDCl_3$) δ 178.0, 149.9, 138.9, 132.6 (q,

J = 33.5 Hz), 130.6 (dd, J = 251.7, 247.9 Hz), 123.2 (q, J = 272.9 Hz), 120.0 – 119.8 (m), 117.9 – 117.5 (m), 50.4, 45.9 (dd, J = 5.2, 2.9 Hz), 41.2 (t, J = 24.8 Hz), 37.8 (t, J = 26.3 Hz), 30.1 (dd, J = 4.6, 3.0 Hz). ^{19}F NMR (470 MHz, $CDCl_3$) δ -63.06, -95.66 (dtt, J = 233.7, 17.6, 12.8 Hz), -97.97 (dp, J = 233.7, 11.4 Hz). HRMS (DART/Orbitrap) calculated for $[M+H]^+$ ($C_{16}H_{13}F_8N_2O_2$) m/z 417.0844; found m/z 417.0836.

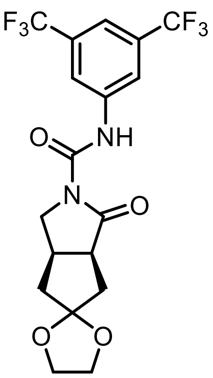

(3a*R*,6a*S*)-*N*-(3,5-bis(trifluoromethyl)phenyl)-1-oxotetrahydro-1*H*-spiro[cyclopenta[*c*]pyrrole-5,2'-[1,3]dioxolane]-2(3*H*)-carboxamide (19) Prepared from (3a*R*,6a*S*)-*N*-(3,5-bis(trifluoromethyl)phenyl)tetrahydro-1*H*-spiro[cyclopenta[*c*]pyrrole-5,2'-[1,3]dioxolane]-2(3*H*)-carboxamide (42.4 mg, 0.1 mmol, 1.0 equiv) following GP-B. The concentrated crude product was then loaded onto a SiO_2 column and eluted with a gradient from 0% hexanes/DCM to 100% hexanes/DCM, then DCM/AcOEt 0% to DCM/AcOEt 20%. The product was obtained as a white solid (34.5 mg, 0.079 mmol, 79% yield). $[\alpha]_D^{20}$ +21 (c 1.0, $CHCl_3$). ^{1}H NMR (500 MHz, $CDCl_3$) δ 11.08 (s, 1H), 8.05 (s, 2H), 7.58 (s, 1H), 4.09 (dd, J = 11.4, 9.0 Hz, 1H), 3.94 – 3.87 (m, 4H), 3.80 (dd, J = 11.5, 2.9 Hz, 1H), 3.27 (td, J = 9.6, 2.8 Hz, 1H), 2.97 – 2.87 (m, 1H), 2.27 – 2.14 (m, 3H), 1.76 (ddd, J = 13.7, 4.3, 1.7 Hz, 1H). ^{13}C NMR (126 MHz, $CDCl_3$) δ 179.7, 150.3, 139.2, 132.5 (q, J = 33.5 Hz), 123.3 (q, J = 272.8 Hz), 119.8 – 119.6 (m), 117.4 – 117.2 (m), 116.4, 65.2, 64.8, 51.6, 46.7, 42.1, 38.4, 29.7. ^{19}F NMR (470 MHz, $CDCl_3$) δ -63.05. HRMS (DART/Orbitrap) calculated for $[M+H]^+$ ($C_{18}H_{17}F_6N_2O_4$) m/z 439.1087; found m/z 439.1091.

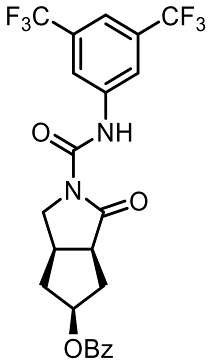

(3a*R*,5*S*,6a*S*)-2-((3,5-bis(trifluoromethyl)phenyl)carbamoyl)-1-oxooctahydrocyclopenta[*c*]pyrrol-5-yl benzoate (20) Prepared from (3a*R*,5*r*,6a*S*)-2-((3,5-bis(trifluoromethyl)phenyl)carbamoyl)octahydrocyclopenta[c]pyrrol-5-yl benzoate (48.6 mg, 0.1 mmol, 1.0 equiv) following GP-B. The concentrated crude product was then loaded onto a SiO_2 column and eluted with a gradient from 0% hexanes/DCM to 100% hexanes/DCM, then

DCM/AcOEt 0% to DCM/AcOEt 20%. The product was obtained as a colorless oil (34.2 mg, 0.068 mmol, 68% yield). $[\alpha]_D^{20}$ +16 (*c* 1.0, $CHCl_3$). ^{1}H NMR (500 MHz, $CDCl_3$, rotamers) δ 10.88 (s, 0.6H), 8.03 – 8.00 (m, 0.8H), 7.85 (s, 1.2H), 7.80 – 7.76 (m, 1.2H), 7.59 (s, 0.6H), 7.60 – 7.52 (m, 0.4H), 7.47 – 7.42 (m, 0.4H), 7.26 – 7.22 (m, 0.6H), 7.12 – 7.08 (m, 1.2H), 5.57 (tt, *J* = 5.0, 1.9 Hz, 0.4H), 5.50 – 5.47 (m, 0.6H), 4.55 (s, 0.4H), 4.20 (dd, *J* = 11.6, 9.2 Hz, 0.6H), 3.91 (dd, *J* = 11.6, 2.6 Hz, 0.6H), 3.69 (dd, *J* = 15.4, 9.0 Hz, 0.4H), 3.41 – 3.35 (m, 0.6H), 3.29 (ddd, *J* = 11.3, 8.2, 3.9 Hz, 0.4H), 3.23 (dd, *J* = 15.4, 5.7 Hz, 0.4H), 3.03 (qt, *J* = 9.5, 2.3 Hz, 0.6H), 2.82 – 2.74 (m, 0.4H), 2.66 (d, *J* = 14.7 Hz, 0.6H), 2.38 (ddd, *J* = 14.6, 10.0, 4.4 Hz, 0.6H), 2.29 – 2.16 (m, 1H), 2.14 – 2.02 (m, 1.4H), 1.92 (d, *J* = 14.8 Hz, 0.4H). ^{13}C NMR (126 MHz, $CDCl_3$, rotamers) δ 179.9, 166.1, 165.6, 150.1, 139.0, 133.3, 133.1, 132.4 (q, *J* = 33.5 Hz), 130.2, 129.8, 129.7, 129.3, 128.7, 128.4, 123.2 (q, *J* = 272.8 Hz), 119.9 – 119.7 (m), 117.4 – 117.2 (m), 87.6, 78.1, 63.8, 52.6, 48.6, 45.8, 41.1, 40.6, 38.67, 37.5, 34.5, 31.0. ^{19}F NMR (470 MHz, $CDCl_3$) δ -63.04. HRMS (DART/Orbitrap) calculated for $[M+H]^+$ ($C_{23}H_{19}F_6N_2O_4$) m/z 501.1244; found m/z 501.1250.

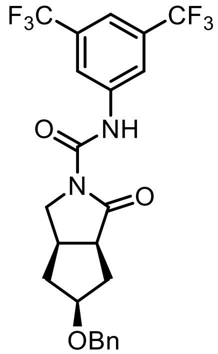

(3a*R*,5*S*,6a*S*)-5-(benzyloxy)-*N*-(3,5-bis(trifluoromethyl)phenyl)-1-oxohexahydrocyclopenta[*c*]pyrrole-2(1*H*)-carboxamide (21) Prepared from (3a*R*,5*r*,6a*S*)-5-(benzyloxy)-*N*-(3,5-bis(trifluoromethyl)phenyl)hexahydrocyclopenta[*c*]pyrrole-2(1*H*)-carboxamide (47.2 mg, 0.1 mmol, 1.0 equiv) following GP-B. The concentrated crude product was then loaded onto a SiO_2 column and eluted with a gradient from 0% hexanes/DCM to 100% hexanes/DCM, then DCM/AcOEt 0% to DCM/AcOEt 20%. The product was obtained as a colorless oil (34.1 mg, 0.070 mmol, 70% yield). $[\alpha]_D^{20}$ +36 (*c* 1.0, $CHCl_3$). ^{1}H NMR (500 MHz, $CDCl_3$) δ 10.83 (s, 1H), 7.86 (s, 2H), 7.56 (s, 1H), 7.17 – 7.13 (m, 2H), 7.05 (t, *J* = 7.7 Hz, 2H), 6.95 – 6.90 (m, 1H), 4.40 (d, *J* = 11.3 Hz, 1H), 4.33 (d, *J* = 11.3 Hz, 1H), 4.12 – 4.07 (m, 2H), 3.81 (dd, *J* = 11.4, 2.6 Hz, 1H), 3.24 (t, *J* = 9.1 Hz, 1H), 2.87 (qt, *J* = 9.5, 2.3 Hz, 1H), 2.62 (d, *J* = 14.0 Hz, 1H), 2.09 (ddd, *J* = 14.0, 9.8, 4.0 Hz, 1H), 1.99 (d, *J* = 14.3 Hz, 1H), 1.87 (ddd, *J* = 14.0, 9.1, 3.4 Hz, 1H). ^{13}C NMR (126 MHz, $CDCl_3$) δ 180.3, 150.2, 139.3, 137.9, 132.2 (q, *J* = 33.4 Hz), 128.3, 127.4, 127.1, 123.3 (q, *J* = 272.7 Hz), 119.8 – 119.6 (m), 117.1 – 116.9 (m), 79.9, 70.0, 52.6, 48.4, 41.2, 36.0, 30.7. ^{19}F NMR (470 MHz, $CDCl_3$) δ -63.02. HRMS (DART/Orbitrap) calculated for $[M+H]^+$ ($C_{23}H_{21}F_6N_2O_3$) m/z 487.1451; found m/z 487.1456.

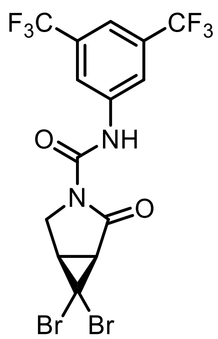

(1*S*,5*R*)-N-(3,5-bis(trifluoromethyl)phenyl)-6,6-dibromo-2-oxo-3-azabicyclo[3.1.0]hexane-3-carboxamide (22) Prepared from (1*R*,5*S*)-N-(3,5-bis(trifluoromethyl)phenyl)-6,6-dibromo-3-azabicyclo[3.1.0]hexane-3-carboxamide (49.6 mg, 0.1 mmol, 1.0 equiv) following GP-B. The concentrated crude product was then loaded onto a SiO_2 column and eluted with a gradient from 0% hexanes/DCM to 100% hexanes/DCM. The product was obtained as a colorless oil (43.4 mg, 0.085 mmol, 85% yield). $[\alpha]_D^{20}$ −18 (*c* 1.0, $CHCl_3$). ^{1}H NMR (500 MHz, $CDCl_3$) δ 10.52 (s, 1H), 8.01 (s, 2H), 7.61 (s, 1H), 4.13 (dd, *J* = 12.6, 6.5 Hz, 1H), 4.03 (d, *J* = 12.7 Hz, 1H), 3.16 (d, *J* = 7.1 Hz, 1H), 2.79 (t, *J* = 6.8 Hz, 1H). ^{13}C NMR (126 MHz, $CDCl_3$) δ 169.7, 149.2, 138.6, 132.6 (q, *J* = 33.6 Hz), 124.2 (d, *J* = 273.1 Hz), 119.9 – 119.7 (m), 117.9 – 117.7 (m), 47.2, 41.3, 30.1, 25.3. ^{19}F NMR (470 MHz, $CDCl_3$) δ -63.05. HRMS (DART/Orbitrap) calculated for $[M+H]^+$ ($C_{14}H_9Br_2F_6N_2O_2$) m/z 508.8929; found m/z 508.8935.

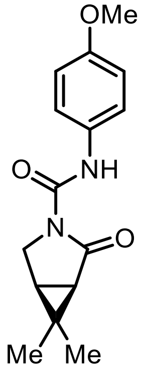

(1*R*,5*S*)-*N*-(4-methoxyphenyl)-6,6-dimethyl-2-oxo-3-azabicyclo[3.1.0]hexane-3-carboxamide (6-OMe) Prepared from (1*R*,5*S*)-*N*-(4-methoxyphenyl)-6,6-dimethyl-3-azabicyclo[3.1.0]hexane-3-carboxamide (26.0 mg, 0.1 mmol, 1.0 equiv) following GP-A. The yield was determined by ^{1}H-NMR using 4,4′-di-*tert*-butylbiphenyl (0.25 equiv) as an internal standard, providing an NMR yield of 53%. The enantiomeric excess of the crude mixture was measured to be 88% *ee*.

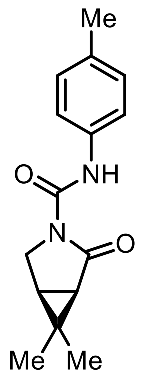

(1*R*,5*S*)-6,6-dimethyl-2-oxo-*N*-(*p*-tolyl)-3-azabicyclo[3.1.0]hexane-3-carboxamide (6-Me) Prepared from (1*R*,5*S*)-6,6-dimethyl-*N*-(*p*-tolyl)-3-azabicyclo[3.1.0]hexane-3-carboxamide (24.4 mg, 0.1 mmol, 1.0 equiv) following GP-A. The yield was determined by ^{1}H-NMR using 4,4′-di-*tert*-butylbiphenyl (0.25 equiv) as an internal standard, providing an NMR yield of 81%. The enantiomeric excess of the crude mixture was measured to be 90% *ee*.

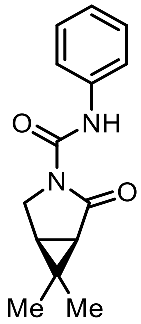

(1*R*,5*S*)-6,6-dimethyl-2-oxo-*N*-phenyl-3-azabicyclo[3.1.0]hexane-3-carboxamide (6-H) Prepared from (1*R*,5*S*)-6,6-dimethyl-*N*-phenyl-3-azabicyclo[3.1.0]hexane-3-carboxamide (23.0 mg, 0.1 mmol, 1.0 equiv) following GP-A. The yield was determined by ^{1}H-NMR using 4,4′-di-*tert*-butylbiphenyl (0.25 equiv) as an internal standard, providing an NMR yield of 81%. The enantiomeric excess of the crude mixture was measured to be 90% *ee*.

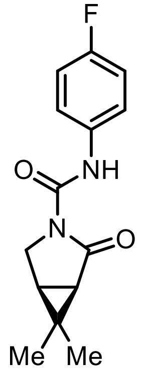

(1*R*,5*S*)-*N*-(4-fluorophenyl)-6,6-dimethyl-2-oxo-3-azabicyclo[3.1.0]hexane-3-carboxamide (6-F) Prepared from (1*R*,5*S*)-*N*-(4-fluorophenyl)-6,6-dimethyl-3-azabicyclo[3.1.0]hexane-3-carboxamide (24.8 mg, 0.1 mmol, 1.0 equiv) following GP-A. The yield was determined by ^{1}H-NMR using 4,4′-di-*tert*-butylbiphenyl (0.25 equiv) as an internal standard, providing an NMR yield of 60%. The enantiomeric excess of the crude mixture was measured to be 92% *ee*.

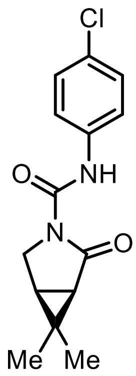

(1*R*,5*S*)-*N*-(4-chlorophenyl)-6,6-dimethyl-2-oxo-3-azabicyclo[3.1.0]hexane-3-carboxamide (6-Cl) Prepared from (1*R*,5*S*)-*N*-(4-chlorophenyl)-6,6-dimethyl-3-azabicyclo[3.1.0]hexane-3-carboxamide (26.5 mg, 0.1 mmol, 1.0 equiv) following GP-A. The yield was determined by ^{1}H-NMR using 4,4′-di-*tert*-butylbiphenyl (0.25 equiv) as an internal standard, providing an NMR yield of 85%. The enantiomeric excess of the crude mixture was measured to be 92% *ee*.

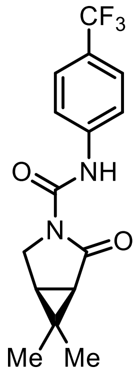

(1*R*,5*S*)-6,6-dimethyl-2-oxo-*N*-(4-(trifluoromethyl)phenyl)-3-azabicyclo[3.1.0]hexane-3-carboxamide (6-CF_3) Prepared from (1*R*,5*S*)-6,6-dimethyl-*N*-(4-(trifluoromethyl)phenyl)-3-azabicyclo[3.1.0]hexane-3-carboxamide (29.8 mg, 0.1 mmol, 1.0 equiv) following GP-A. The yield was determined by ^{1}H-NMR using 4,4′-di-*tert*-butylbiphenyl (0.25 equiv) as an internal standard, providing an NMR yield of 80%. The enantiomeric excess of the crude mixture was measured to be 92% *ee*.

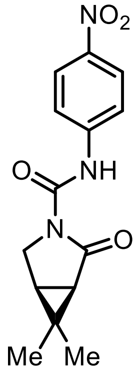

(1*R*,5*S*)-6,6-dimethyl-*N*-(4-nitrophenyl)-2-oxo-3-azabicyclo[3.1.0]hexane-3-carboxamide (6-NO_2) Prepared from (1*R*,5*S*)-6,6-dimethyl-*N*-(4-nitrophenyl)-3-azabicyclo[3.1.0]hexane-3-carboxamide (27.5 mg, 0.1 mmol, 1.0 equiv) following GP-A. The yield was determined by ^{1}H-

NMR using 4,4′-di-*tert*-butylbiphenyl (0.25 equiv) as an internal standard, providing an NMR yield of 83%. The enantiomeric excess of the crude mixture was measured to be 90% *ee*.

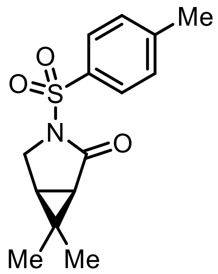

(1*R*,5*S*)-6,6-dimethyl-3-tosyl-3-azabicyclo[3.1.0]hexan-2-one (2-Ts) Prepared from (1*R*,5*S*)-6,6-dimethyl-3-tosyl-3-azabicyclo[3.1.0]hexane (26.5 mg, 0.1 mmol, 1.0 equiv) following GP-A. The yield was determined by ^{1}H-NMR using 4,4′-di-*tert*-butylbiphenyl (0.25 equiv) as an internal standard, providing an NMR yield of 7%. The enantiomeric excess of the crude mixture was measured to be 18% *ee*.

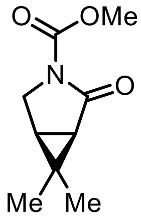

methyl (1*R*,5*S*)-6,6-dimethyl-2-oxo-3-azabicyclo[3.1.0]hexane-3-carboxylate (2-OMe) Prepared from methyl (1*R*,5*S*)-6,6-dimethyl-3-azabicyclo[3.1.0]hexane-3-carboxylate (19.6 mg, 0.1 mmol, 1.0 equiv) following GP-A. The yield was determined by ^{1}H-NMR using 4,4′-di-*tert*-butylbiphenyl (0.25 equiv) as an internal standard, providing an NMR yield of 10%. The enantiomeric excess of the crude mixture was measured to be 15% *ee*.

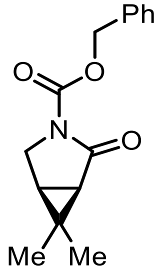

benzyl (1*R*,5*S*)-6,6-dimethyl-2-oxo-3-azabicyclo[3.1.0]hexane-3-carboxylate (2-OBn) Prepared from benzyl (1R,5S)-6,6-dimethyl-3-azabicyclo[3.1.0]hexane-3-carboxylate (24.5 mg, 0.1 mmol, 1.0 equiv) following GP-A. The yield was determined by ^{1}H-NMR using 4,4′-di-*tert*-butylbiphenyl (0.25 equiv) as an internal standard, providing an NMR yield of 46%. The enantiomeric excess of the crude mixture was measured to be 32% *ee*.

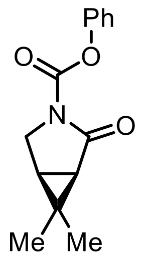

phenyl (1*R*,5*S*)-6,6-dimethyl-2-oxo-3-azabicyclo[3.1.0]hexane-3-carboxylate (2-OPh) Prepared from phenyl (1*R*,5*S*)-6,6-dimethyl-3-azabicyclo[3.1.0]hexane-3-carboxylate (23.1 mg, 0.1 mmol, 1.0 equiv) following GP-A. The yield was determined by ^{1}H-NMR using 4,4′-di-*tert*-butylbiphenyl (0.25 equiv) as an internal standard, providing an NMR yield of 8% of the product (13% *ee*).

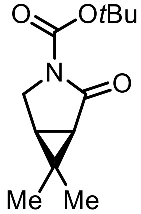

***tert*-butyl (1*R*,5*S*)-6,6-dimethyl-2-oxo-3-azabicyclo[3.1.0]hexane-3-carboxylate (2-O^{t}Bu)** Prepared from *tert*-butyl (1*R*,5*S*)-6,6-dimethyl-3-azabicyclo[3.1.0]hexane-3-carboxylate (21.1 mg, 0.1 mmol, 1.0 equiv) following GP-A. The yield was determined by ^{1}H-NMR using 4,4′-di-*tert*-butylbiphenyl (0.25 equiv) as an internal standard, providing an NMR yield of 90%. The enantiomeric excess of the crude mixture was measured to be 36% *ee*.

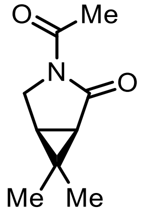

(1*R*,5*S*)-3-acetyl-6,6-dimethyl-3-azabicyclo[3.1.0]hexan-2-one (2-Me) Prepared from methyl 1-((1*R*,5*S*)-6,6-dimethyl-3-azabicyclo[3.1.0]hexan-3-yl)ethan-1-one (15.3 mg, 0.1 mmol, 1.0 equiv) following GP-A. The yield was determined by ^{1}H-NMR using 4,4′-di-*tert*-butylbiphenyl (0.25 equiv) as an internal standard, providing an NMR yield of 6%. The enantiomeric excess of the crude mixture was measured to be 33% *ee*.

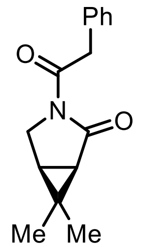

(1*R*,5*S*)-6,6-dimethyl-3-(2-phenylacetyl)-3-azabicyclo[3.1.0]hexan-2-one (2-Bn) Prepared from (1*R*,5*S*)-6,6-dimethyl-3-(2-phenylacetyl)-3-azabicyclo[3.1.0]hexan-2-one (24.3 mg, 0.1

mmol, 1.0 equiv) following GP-A. The yield was determined by ^{1}H-NMR using 4,4′-di-*tert*-butylbiphenyl (0.25 equiv) as an internal standard, providing an NMR yield of 12%. The enantiomeric excess of the crude mixture was measured to be 24% *ee*.

5. Substrate Preparation:

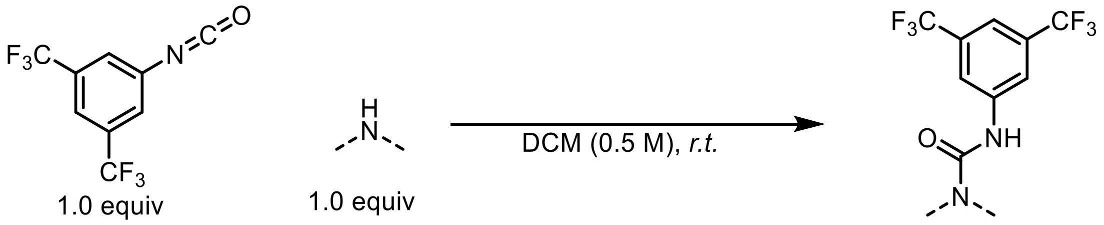

GP-C – Urea formation: The amine (1.0 equiv) was dissolved in DCM (0.5 M) in a round-bottom flask. The corresponding isocyanate (1.0 equiv) was added dropwise or portion wise and the reaction was stirred for 20 minutes. The crude product was purified by crystallization or by a column chromatography.

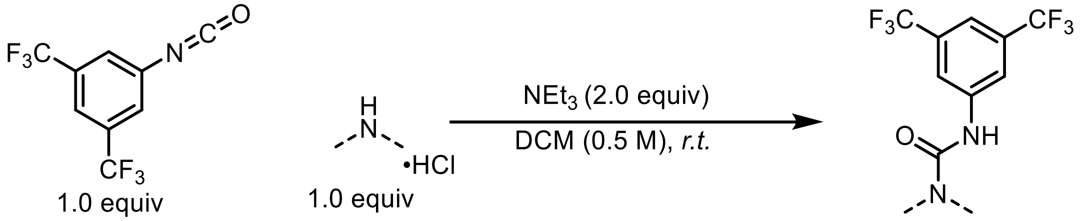

GP-D – Urea formation from HCl-amine salts: The amine (1.0 equiv) was dissolved in DCM (0.5 M) in a round-bottom flask and the triethylamine (2.0 equiv) was added. The corresponding isocyanate (1.0 equiv) was added dropwise or portion wise and the reaction was stirred for 20 minutes. The crude product was purified by a column chromatography.

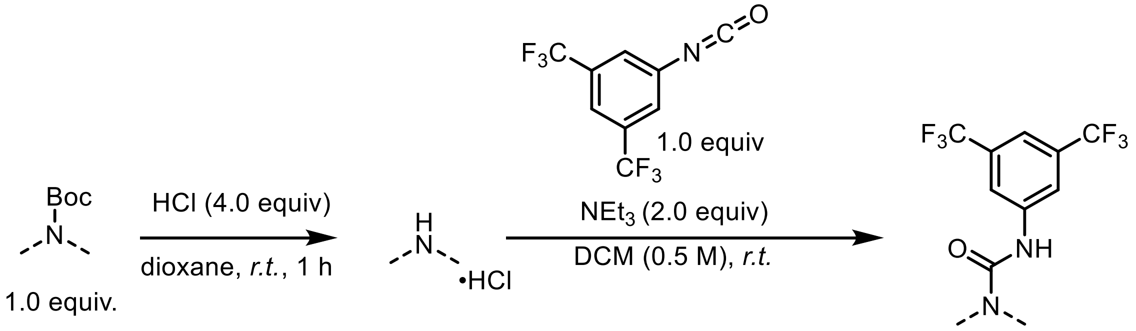

GP-E – Urea formation from Boc-protected amines: Boc-protected amine was placed in a round-bottom flask, 4 M HCl in dioxane (4.0 equiv) was added, and the mixture was stirred for 1 h. Then the solvent was evaporated, and the amine salt was used in the next step without further purification. The crude amine salt from the previous step (1.0 equiv) was placed in a round-bottom flask and then triethylamine (2.0 equiv) and DCM (0.5 M) were added. The corresponding isocyanate (1.0 equiv) was added dropwise or portion wise and the reaction was stirred for 20 minutes. The crude product was purified by column chromatography.

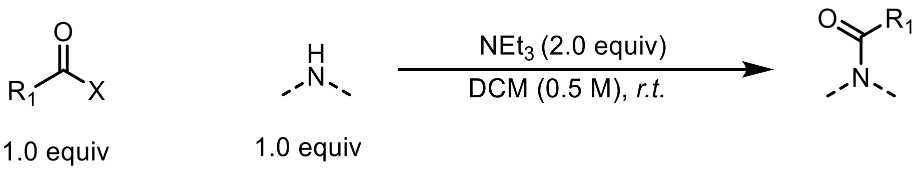

GP-F – Amine protection: The amine (1.0 equiv) was dissolved in DCM (0.5 M) in the round-bottom flask, and the triethylamine (2.0 equiv) was added. The protecting agent (1.0 equiv) was added dropwise or portion wise. After stirring for 16 hours, $NaHCO_3$ (50 mL, sat.) was added and mixture was extracted with DCM (3 × 50 mL). Combined organic phases were dried over $MgSO_4$, concentrated, and the crude product was purified by column chromatography.

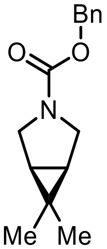

Benzyl (1*R*,5*S*)-6,6-dimethyl-3-azabicyclo[3.1.0]hexane-3-carboxylate (3-OBn) Prepared from (1*R*,5*S*)-6,6-dimethyl-3-azabicyclo[3.1.0]hexane (1.00 g, 8.99 mmol, 1.0 equiv) and benzyl chloroformate (1.53 g, 8.99 mmol, 1.0 equiv) following GP-F. The concentrated crude product was then loaded onto a SiO_2 column and eluted with a gradient from 0% hexanes/EtOAc to 100% hexanes/EtOAc. The product was obtained as a colorless oil (1.61 g, 6.56 mmol, 73% yield). ^{1}H NMR (500 MHz, $CDCl_3$) δ 7.38 – 7.27 (m, 5H), 5.16 – 5.05 (m, 2H), 3.58 – 3.48 (m, 2H), 3.40 (dd, *J* = 17.1, 11.2 Hz, 2H), 1.37 – 1.30 (m, 2H), 1.02 (s, 3H), 0.91 (s, 3H). ^{13}C NMR (126 MHz, $CDCl_3$) δ 154.3, 137.2, 128.6, 128.0, 127.8, 66.7, 46.7, 46.1, 28.0, 27.2, 26.4, 19.2, 12.5. HRMS (DART/Orbitrap) calculated for $[M+H]^+$ ($C_{15}H_{20}NO_2$) m/z 246.1489; found m/z 246.1498.

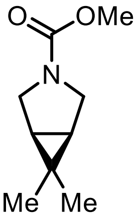

Methyl (1*R*,5*S*)-6,6-dimethyl-3-azabicyclo[3.1.0]hexane-3-carboxylate (3-OMe) Prepared from (1*R*,5*S*)-6,6-dimethyl-3-azabicyclo[3.1.0]hexane (445 mg, 4.0 mmol, 1.0 equiv) and methyl chloroformate (378 mg, 4.0 mmol, 1.0 equiv) following GP-F. The concentrated crude product was then loaded onto a SiO_2 column and eluted with a gradient from 0% hexanes/EtOAc to 100% hexanes/EtOAc. The product was obtained as a colorless oil (649 mg, 3.83 mmol, 96% yield). ^{1}H NMR (500 MHz, $CDCl_3$) δ 3.66 (s, 3H), 3.55 – 3.49 (m, 1H), 3.48 – 3.43 (m, 1H), 3.38 (d, *J* = 11.3 Hz, 1H), 3.30 (d, *J* = 11.1 Hz, 1H), 1.35 – 1.30 (m, 2H), 1.02 (s, 3H), 0.90 (s, 3H). ^{13}C NMR (126 MHz, $CDCl_3$) δ 155.0, 52.4, 46.6, 46.0, 28.0, 27.2, 26.4, 19.2, 12.5. HRMS (DART/Orbitrap) calculated for $[M+H]^+$ ($C_9H_{16}N_2O$) m/z 170.1176; found m/z 170.1184.

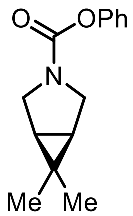

Phenyl (1*R*,5*S*)-6,6-dimethyl-3-azabicyclo[3.1.0]hexane-3-carboxylate (3-OPh) Prepared from (1*R*,5*S*)-6,6-dimethyl-3-azabicyclo[3.1.0]hexane (222 mg, 2.0 mmol, 1.0 equiv) and phenyl chloroformate (313 mg, 2.0 mmol, 1.0 equiv) following GP-F. The concentrated crude product was then loaded onto a SiO_2 column and eluted with a gradient from 0% hexanes/EtOAc to 100% hexanes/EtOAc. The product was obtained as a colorless oil (463 mg, 1.86 mmol, 93% yield). ^{1}H NMR (500 MHz, $CDCl_3$) δ 7.34 (t, *J* = 8.0 Hz, 2H), 7.17 (t, *J* = 7.5 Hz, 1H), 7.11 (d, *J* = 7.5 Hz, 2H), 3.71 (dd, *J* = 11.2, 5.0 Hz, 1H), 3.62 (dd, *J* = 11.4, 5.0 Hz, 1H), 3.56 (d, *J* = 11.1 Hz, 1H), 3.48 (d, *J* = 11.6 Hz, 1H), 1.46 – 1.37 (m, 2H), 1.07 (s, 3H), 1.01 (s, 3H). ^{13}C NMR (126 MHz, $CDCl_3$) δ 152.5, 151.5, 129.3, 125.2, 121.8, 46.9, 46.6, 28.0, 27.2, 26.4, 19.3, 12.6. HRMS (DART/Orbitrap) calculated for $[M+H]^+$ ($C_{14}H_{18}NO_2$) m/z 232.1332; found m/z 232.1342.

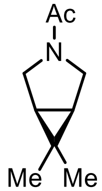

1-((1*R*,5*S*)-6,6-dimethyl-3-azabicyclo[3.1.0]hexan-3-yl)ethan-1-one (3-Me) Prepared from (1*R*,5*S*)-6,6-dimethyl-3-azabicyclo[3.1.0]hexane (222 mg, 2.0 mmol, 1.0 equiv) and acetyl chloride (157 mg, 2.0 mmol, 1.0 equiv) following GP-F. The concentrated crude product was then loaded onto a SiO_2 column and eluted with a gradient from 0% DCM/MeOH to 10% DCM/MeOH. The product was obtained as a colorless oil (273 mg, 1.78 mmol, 89% yield). ^{1}H NMR (500 MHz, $CDCl_3$) δ 3.57 (qd, *J* = 5.3, 1.9 Hz, 1H), 3.50 – 3.38 (m, 2H), 3.30 (d, *J* = 10.5 Hz, 1H), 1.92 (s, 3H), 1.39 – 1.28 (m, 2H), 0.98 (s, 3H), 0.85 (s, 3H). ^{13}C NMR (126 MHz, $CDCl_3$) δ 168.8, 47.6, 45.9, 27.8, 26.9, 26.2, 22.6, 19.2, 12.4. HRMS (DART/Orbitrap) calculated for $[M+H]^+$ ($C_9H_{16}NO$) m/z 154.1226; found m/z 154.1232.

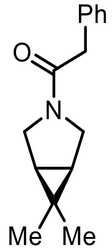

1-((1*R*,5*S*)-6,6-dimethyl-3-azabicyclo[3.1.0]hexan-3-yl)-2-phenylethan-1-one (3-Bn) Prepared from (1*R*,5*S*)-6,6-dimethyl-3-azabicyclo[3.1.0]hexane (445 mg, 4.00 mmol, 1.0 equiv) and 2-phenylacetyl chloride (309 mg, 2.0 mmol, 1.0 equiv) following GP-F. The concentrated crude product was then loaded onto a SiO_2 column and eluted with a gradient from 20% hexanes/EtOAc to 100% hexanes/EtOAc. The product was obtained as a pale-yellow oil (692 mg, 3.02 mmol, 75% yield). ^{1}H NMR (500 MHz, $CDCl_3$) δ 7.33 – 7.27 (m, 2H), 7.26 – 7.20 (m, 3H), 3.64 – 3.54 (m, 4H), 3.52 (d, *J* = 12.7 Hz, 1H), 3.38 (d, *J* = 10.5 Hz, 1H), 1.34 (dd, *J* = 3.4, 1.8 Hz, 2H), 1.00 (s,

3H), 0.78 (s, 3H). ^{13}C NMR (126 MHz, $CDCl_3$) δ 169.2, 134.7, 129.1, 128.7, 126.9, 47.0, 46.4, 42.6, 28.1, 26.8, 26.3, 19.3, 12.4. HRMS (DART/Orbitrap) calculated for $[M+H]^+$ ($C_{15}H_{20}NO$) m/z 230.1539; found m/z 230.1534.

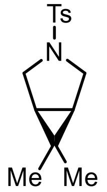

(1*R*,5*S*)-6,6-dimethyl-3-tosyl-3-azabicyclo[3.1.0]hexane (3-Ts) Prepared from (1R,5S)-6,6-dimethyl-3-azabicyclo[3.1.0]hexane (445 mg, 4.0 mmol, 1.0 equiv) and tosyl chloride (381 mg, 2.0 mmol, 1.0 equiv) following GP-F. The concentrated crude product was then loaded onto a SiO_2 column and eluted with a gradient from 0% hexanes/EtOAc to 100% hexanes/EtOAc. The product was obtained as a white solid (962 mg, 3.63 mmol, 91% yield). ^{1}H NMR (500 MHz, $CDCl_3$) δ 7.70 – 7.66 (m, 2H), 7.30 (d, *J* = 7.8 Hz, 2H), 3.34 (d, *J* = 9.5 Hz, 2H), 3.31 – 3.25 (m, 2H), 2.41 (s, 3H), 1.27 – 1.23 (m, 2H), 0.954 (s, 3H), 0.948 (s, 3H). ^{13}C NMR (126 MHz, $CDCl_3$) δ 143.4, 134.1, 129.7, 127.5, 47.8, 28.2, 26.6, 21.6, 19.9, 12.8. HRMS (DART/Orbitrap) calculated for $[M+H]^+$ ($C_{14}H_{20}NO_2S$) m/z 266.1209; found m/z 266.1220.

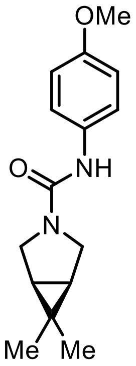

(1*R*,5*S*)-*N*-(4-methoxyphenyl)-6,6-dimethyl-3-azabicyclo[3.1.0]hexane-3-carboxamide (4-OMe) Prepared from (1*R*,5*S*)-6,6-dimethyl-3-azabicyclo[3.1.0]hexane (222 mg, 2.0 mmol, 1.0 equiv) and 4-methoxyphenyl isocyanate (298 mg, 2.0 mmol, 1.0 equiv) following GP-C. The concentrated crude product was then purified by crystallization from DCM/hexanes. The product was obtained as a white solid (345 mg, 1.33 mmol, 66% yield). ^{1}H NMR (500 MHz, $CDCl_3$) δ 7.32 – 7.25 (m, 2H), 6.81 (d, *J* = 9.0 Hz, 2H), 5.98 (s, 1H), 3.77 (s, 3H), 3.59 (dd, *J* = 9.8, 1.9 Hz, 2H), 3.43 – 3.38 (m, 2H), 1.43 (dd, *J* = 3.6, 1.8 Hz, 2H), 1.05 (s, 3H), 0.96 (s, 3H). ^{13}C NMR (126 MHz, $CDCl_3$) δ 155.7, 153.6, 132.3, 121.8, 114.2, 55.6, 46.1, 27.8, 26.3, 19.3, 12.6. HRMS (DART/Orbitrap) calculated for $[M+H]^+$ ($C_{15}H_{21}N_2O_2$) m/z 261.1598; found m/z 261.1607.

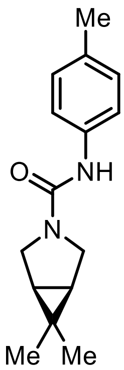

(1*R*,5*S*)-6,6-dimethyl-*N*-(*p*-tolyl)-3-azabicyclo[3.1.0]hexane-3-carboxamide (4-Me) Prepared from (1R,5S)-6,6-dimethyl-3-azabicyclo[3.1.0]hexane (222 mg, 2.0 mmol, 1.0 equiv) and *p*-tolyl isocyanate (266 mg, 2.0 mmol, 1.0 equiv) following GP-C. The concentrated crude product was then purified by crystallization from DCM/hexanes. The product was obtained as a white solid (305 mg, 1.25 mmol, 62% yield). ^{1}H NMR (500 MHz, $CDCl_3$) δ 7.28 (d, *J* = 8.5 Hz, 2H), 7.06 (d, *J* = 8.4 Hz, 2H), 6.06 (s, 1H), 3.63 – 3.55 (m, 2H), 3.44 – 3.38 (m, 2H), 2.28 (s, 3H), 1.43 (dd, *J* = 3.7, 1.7 Hz, 2H), 1.05 (s, 3H), 0.96 (s, 3H). ^{13}C NMR (126 MHz, $CDCl_3$) δ 153.4, 136.6, 132.4, 129.4, 119.7, 46.1, 27.8, 26.3, 20.9, 19.3, 12.5. HRMS (DART/Orbitrap) calculated for $[M+H]^+$ ($C_{15}H_{21}N_2O$) m/z 245.1648; found m/z 245.1660.

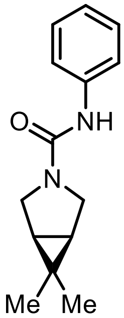

(1*R*,5*S*)-6,6-dimethyl-N-phenyl-3-azabicyclo[3.1.0]hexane-3-carboxamide (4-H) Prepared from (1*R*,5*S*)-6,6-dimethyl-3-azabicyclo[3.1.0]hexane (445 mg, 4.0 mmol, 1.0 equiv) and phenyl isocyanate (238 mg, 2.0 mmol, 1.0 equiv) following GP-C. The concentrated crude product was then purified by crystallization from DCM/hexanes. The product was obtained as a white solid (921 mg, 3.52 mmol, 88%). ^{1}H NMR (500 MHz, $CDCl_3$) δ 7.43 – 7.39 (m, 2H), 7.29 – 7.24 (m, 2H), 7.01 (tt, *J* = 7.3, 1.2 Hz, 1H), 6.15 (s, 1H), 3.62 (ddd, *J* = 10.1, 3.7, 2.0 Hz, 2H), 3.54 – 3.32 (m, 2H), 1.44 (dd, *J* = 3.7, 1.7 Hz, 2H), 1.07 (s, 3H), 0.97 (s, 3H). ^{13}C NMR (126 MHz, $CDCl_3$) δ 153.2, 139.2, 128.9, 122.9, 119.5, 46.1, 27.8, 26.3, 19.3, 12.5. HRMS (DART/Orbitrap) calculated for $[M+H]^+$ ($C_{14}H_{19}N_2O$) m/z 231.1492; found m/z 231.1488.

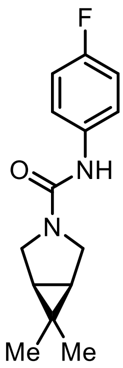

(1*R*,5*S*)-*N*-(4-fluorophenyl)-6,6-dimethyl-3-azabicyclo[3.1.0]hexane-3-carboxamide (4-F)
Prepared from (1*R*,5*S*)-6,6-dimethyl-3-azabicyclo[3.1.0]hexane (222 mg, 2.0 mmol, 1.0 equiv) and 4-fluorophenyl isocyanate (274 mg, 2.0 mmol, 1.0 equiv) following GP-C. The concentrated crude product was then purified by crystallization from DCM/hexanes. The product was obtained as a white solid (390 mg, 1.57 mmol, 79% yield). ^{1}H NMR (500 MHz, $CDCl_3$) δ 7.36 – 7.31 (m, 2H), 6.97 – 6.91 (m, 2H), 6.16 (s, 1H), 3.58 (ddd, *J* = 10.1, 3.7, 2.0 Hz, 2H), 3.51 – 3.28 (m, 2H), 1.45 – 1.38 (m, 2H), 1.05 (s, 3H), 0.94 (s, 3H). ^{13}C NMR (126 MHz, $CDCl_3$) δ 159.7, 157.8, 153.4, 135.2 (d, *J* = 2.7 Hz), 121.5 (d, *J* = 7.7 Hz), 115.4 (d, *J* = 22.5 Hz), 46.1, 27.8, 26.3, 19.3, 12.5. ^{19}F NMR (470 MHz, $CDCl_3$) δ -120.48. HRMS (DART/Orbitrap) calculated for $[M+H]^+$ ($C_{14}H_{18}FN_2O$) m/z 249.1398; found m/z 249.1401.

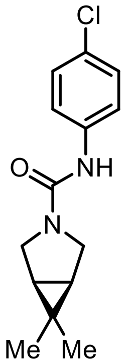

(1*R*,5*S*)-*N*-(4-chlorophenyl)-6,6-dimethyl-3-azabicyclo[3.1.0]hexane-3-carboxamide (4-Cl)
Prepared from (1*R*,5*S*)-6,6-dimethyl-3-azabicyclo[3.1.0]hexane (222 mg, 2.0 mmol, 1.0 equiv) and 4-chlorophenyl isocyanate (307 mg, 2.0 mmol, 1.0 equiv) following GP-C. The concentrated crude product was then purified by crystallization from DCM/hexanes. The product was obtained as a white solid (408 mg, 1.54 mmol, 77% yield). ^{1}H NMR (500 MHz, $CDCl_3$) δ 7.38 – 7.32 (m, 2H), 7.24 – 7.19 (m, 2H), 6.16 – 6.05 (m, 1H), 3.60 (tt, *J* = 8.4, 4.2 Hz, 2H), 3.50 – 3.30 (m, 2H), 1.45 (dd, *J* = 3.9, 1.4 Hz, 2H), 1.06 (s, 3H), 0.96 (s, 3H). ^{13}C NMR (126 MHz, $CDCl_3$) δ 153.0, 137.8, 128.9, 127.8, 120.7, 46.2, 27.8, 26.3, 19.4, 12.5. HRMS (DART/Orbitrap) calculated for $[M+H]^+$ ($C_{14}H_{18}ClN_2O$) m/z 265.1102; found m/z 265.1112.

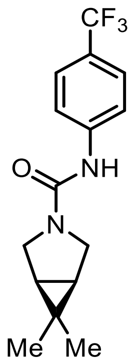

(1*R*,5*S*)-6,6-dimethyl-*N*-(4-(trifluoromethyl)phenyl)-3-azabicyclo[3.1.0]hexane-3-carboxamide (4-CF_3) Prepared from (1*R*,5*S*)-6,6-dimethyl-3-azabicyclo[3.1.0]hexane (222 mg, 2.0 mmol, 1.0 equiv) and 4-(trifluoromethyl)phenyl isocyanate (374 mg, 2.0 mmol, 1.0 equiv) following GP-C. The concentrated crude product was then purified by crystallization from DCM/hexanes. The product was obtained as a white solid (422 mg, 1.41 mmol, 71% yield). ^{1}H NMR (500 MHz, $CDCl_3$) δ 7.56 – 7.48 (m, 4H), 6.23 (s, 1H), 3.69 – 3.61 (m, 2H), 3.44 (s, 2H), 1.47 (dd, *J* = 3.9, 1.4 Hz, 2H), 1.07 (s, 3H), 0.97 (s, 3H). ^{13}C NMR (126 MHz, $CDCl_3$) δ 152.6, 142.4, 126.28 (q, J = 3.8 Hz)., 118.7, 46.3, 27.8, 26.3, 19.4, 12.6. The ^{13}C peak for the CF_3-group was not observed due to poor signal to noise ratio. ^{19}F NMR (470 MHz, $CDCl_3$) δ -61.88. HRMS (DART/Orbitrap) calculated for $[M+H]^+$ ($C_{15}H_{18}F_3N_2O$) m/z 299.1366; found m/z 299.1377.

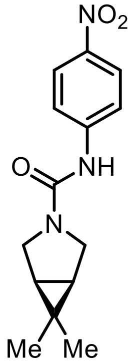

(1*R*,5*S*)-6,6-dimethyl-*N*-(4-nitrophenyl)-3-azabicyclo[3.1.0]hexane-3-carboxamide (4-NO_2) Prepared from (1*R*,5*S*)-6,6-dimethyl-3-azabicyclo[3.1.0]hexane (222 mg, 2.0 mmol, 1.0 equiv) and 4-nitrophenyl isocyanate (328 mg, 2.0 mmol, 1.0 equiv) following GP-C. The concentrated crude product was then purified by crystallization from DCM/hexanes. The product was obtained as a yellowish solid (465 mg, 1.69 mmol, 84% yield). ^{1}H NMR (500 MHz, $CDCl_3$) δ 8.18 – 8.11 (m, 2H), 7.62 – 7.55 (m, 2H), 6.52 (s, 1H), 3.69 – 3.62 (m, 2H), 3.45 (s, 2H), 1.54 – 1.44 (m, 2H), 1.07 (s, 3H), 0.96 (s, 3H). ^{13}C NMR (126 MHz, $CDCl_3$) δ 152.1, 145.4, 142.5, 125.2, 118.2, 46.4, 27.7, 26.2, 19.5, 12.5. HRMS (DART/Orbitrap) calculated for $[M+H]^+$ ($C_{14}H_{18}N_3O_3$) m/z 276.1343; found m/z 276.1354.

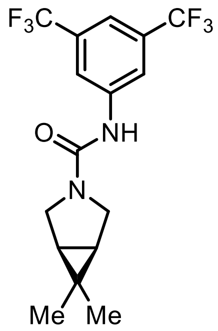

(1*R*,5*S*)-N-(3,5-bis(trifluoromethyl)phenyl)-6,6-dimethyl-3-azabicyclo[3.1.0]hexane-3-carboxamide (A) Prepared from (1*R*,5*S*)-6,6-dimethyl-3-azabicyclo[3.1.0]hexane (1.67 g, 15.0 mmol, 1.0 equiv) and 3,5-bis(trifluoromethyl)phenyl isocyanate (3.83 g, 15.0 mmol, 1.0 equiv) following GP-A. The concentrated crude product was then purified by crystallization from DCM/hexanes. The product was obtained as a white solid (4.07 g, 11.11 mmol, 74% yield). ^{1}H NMR (500 MHz, $CDCl_3$) δ 7.93 (s, 2H), 7.48 (s, 1H), 6.55 – 6.45 (m, 1H), 3.67 – 3.62 (m, 2H), 3.44 (s, 2H), 1.53 – 1.44 (m, 2H), 1.07 (s, 3H), 0.95 (s, 3H). ^{13}C NMR (126 MHz, $CDCl_3$) δ 152.4, 140.7, 132.2 (q, J = 33.5 Hz), 123.4 (q, J = 272.6 Hz), 119.2 – 118.9 (m), 116.2 – 116.0 (m), 46.3, 27.7, 26.2, 19.4, 12.5. ^{19}F NMR (470 MHz, $CDCl_3$) δ -63.05. HRMS (DART/Orbitrap) calculated for $[M+H]^+$ ($C_{16}H_{17}F_6N_2O$) m/z 367.1240; found m/z 367.1254.

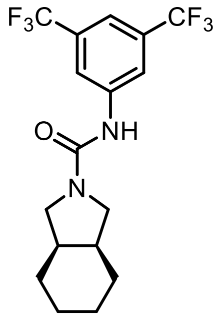

(3a*R*,7a*S*)-*N*-(3,5-bis(trifluoromethyl)phenyl)octahydro-2*H*-isoindole-2-carboxamide (B) Prepared from (3a*R*,7a*S*)-octahydro-1*H*-isoindole (250 mg, 2.0 mmol, 1.0 equiv) and 3,5-bis(trifluoromethyl)phenyl isocyanate (510 mg, 2.0 mmol, 1.0 equiv) following GP-C. The concentrated crude product was then purified by crystallization from DCM/hexanes. The product was obtained as a white solid (460 mg, 1.21 mmol, 60% yield). ^{1}H NMR (500 MHz, $CDCl_3$) δ 7.94 (s, 2H), 7.47 (s, 1H), 6.68 – 6.57 (m, 1H), 3.47 (dd, J = 9.5, 6.9 Hz, 2H), 3.42 – 3.31 (m, 2H), 2.31 (s, 2H), 1.72 – 1.60 (m, 2H), 1.57 – 1.33 (m, 6H). ^{13}C NMR (126 MHz, $CDCl_3$) δ 153.8, 140.9, 132.2 (q, J = 33.2 Hz), 123.4 (q, J = 272.7 Hz), 119.1 – 118.9 (m), 116.0 – 115.7 (m), 50.0, 37.3, 25.9, 22.8. ^{19}F NMR (470 MHz, $CDCl_3$) δ -63.04. HRMS (DART/Orbitrap) calculated for $[M+H]^+$ ($C_{17}H_{19}F_6N_2O$) m/z 381.1396; found m/z 381.1410.

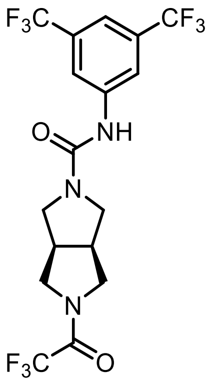

(3a*R*,6a*S*)-*N*-(3,5-bis(trifluoromethyl)phenyl)-5-(2,2,2-trifluoroacetyl)hexahydropyrrolo[3,4-*c*]pyrrole-2(1*H*)-carboxamide (C) Prepared from *tert*-butyl 5-(2,2,2-trifluoroacetyl)hexahydropyrrolo[3,4-*c*]pyrrole-2(1*H*)-carboxylate (1.54 g, 5.0 mmol, 1.0 equiv) and 3,5-bis(trifluoromethyl)phenyl isocyanate (1.28 g, 5.0 mmol, 1.0 equiv) following GP-E. The concentrated crude product was then loaded onto a SiO_2 column and eluted with a gradient from 0% hexanes/EtOAc to 100% hexanes/EtOAc and then crystalized from DCM/hexanes. The product was obtained as a white solid (2.32 g, 3.56 mmol, 71% yield over 2 steps). ^{1}H NMR (500 MHz, DMSO-*d6*) δ 8.88 (s, 1H), 8.27 (s, 2H), 7.56 (s, 1H), 3.89 (dd, *J* = 11.3, 7.3 Hz, 1H), 3.76 (dd, *J* = 12.9, 7.8 Hz, 1H), 3.68 (ddd, *J* = 10.4, 7.5, 2.4 Hz, 2H), 3.54 (dd, *J* = 11.4, 5.1 Hz, 1H), 3.45 – 3.35 (m, 3H), 3.16 – 3.04 (m, 1H), 3.03 – 2.96 (m, 1H). ^{13}C NMR (126 MHz, DMSO) δ 154.3 (q, *J* = 36.1 Hz), 153.4, 142.6, 130.4 (q, *J* = 32.5 Hz), 123.4 (q, *J* = 272.6 Hz), 118.8 – 118.3 (m), 116.1 (q, *J* = 288.0 Hz), 114.1 – 113.8 (m), 51.1, 49.9 – 49.7 (m), 49.5, 49.0, 42.0. ^{19}F NMR (470 MHz, DMSO) δ -61.78 (s, 6F), -71.33 (s, 3F).
HRMS (DART/Orbitrap) calculated for $[M+H]^+$ ($C_{17}H_{15}F_9N_3O_2$) m/z 464.1015; found m/z 464.1032.

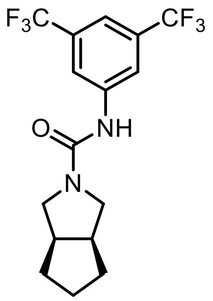

(3a*R*,6a*S*)-*N*-(3,5-bis(trifluoromethyl)phenyl)hexahydrocyclopenta[*c*]pyrrole-2(1*H*)-carboxamide (S1) Prepared from (3a*R*,6a*S*)-octahydrocyclopenta[*c*]pyrrole hydrochloride (738 mg, 5.0 mmol, 1.0 equiv) and 3,5-bis(trifluoromethyl)phenyl isocyanate (1.28 g, 5.0 mmol, 1.0 equiv) following GP-D. The concentrated crude product was then loaded onto a SiO_2 column and eluted with a gradient from 50% hexanes/DCM to 100% hexanes/DCM. The product was obtained as a white solid (1.60 g, 4.37 mmol, 87% yield). ^{1}H NMR (500 MHz, $CDCl_3$) δ 7.93 (s, 2H), 7.47 (s, 1H), 6.66 – 6.57 (m, 1H), 3.68 (dd, *J* = 10.2, 8.0 Hz, 2H), 3.26 (dd, *J* = 10.3, 3.8 Hz, 2H), 2.82 – 2.69 (m, 2H), 1.93 – 1.84 (m, 2H), 1.83 – 1.75 (m, 1H), 1.71 – 1.61 (m, 1H), 1.54 – 1.45 (m, 2H). ^{13}C NMR (126 MHz, $CDCl_3$) δ 153.2, 140.9, 132.16 (q, *J* = 33.2 Hz), 123.4 (q, *J* = 272.7 Hz), 119.2 – 118.9 (m), 116.1 – 115.8 (m), 52.3, 43.3, 32.1, 25.5. ^{19}F NMR (470 MHz, $CDCl_3$) δ -63.05. HRMS (DART/Orbitrap) calculated for $[M+H]^+$ ($C_{16}H_{17}F_6N_2O$) m/z 367.1240; found m/z 367.1254.

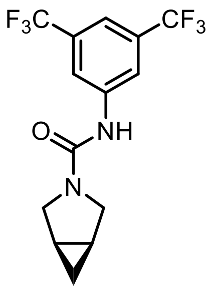

(1*R*,5*S*)-*N*-(3,5-bis(trifluoromethyl)phenyl)-3-azabicyclo[3.1.0]hexane-3-carboxamide (S2) Prepared from (1*R*,5*S*)-3-azabicyclo[3.1.0]hexane hydrochloride (478 mg, 4.0 mmol, 1.0 equiv) and 3,5-bis(trifluoromethyl)phenyl isocyanate (1.02 g, 4.0 mmol, 1.0 equiv) following GP-D. The concentrated crude product was then loaded onto a SiO_2 column and eluted with a gradient from 50% hexanes/DCM to 100% hexanes/DCM. The product was obtained as a white solid (1.09 g, 3.22 mmol, 81% yield). ^{1}H NMR (500 MHz, DMSO) δ 8.79 (s, 1H), 8.25 (s, 2H), 7.53 (s, 1H), 3.63 (d, J = 10.2 Hz, 2H), 3.41 (d, J = 10.3 Hz, 2H), 1.62 – 1.57 (m, 2H), 0.69 (td, J = 7.8, 4.7 Hz, 1H), 0.13 (q, J = 4.2 Hz, 1H). ^{13}C NMR (126 MHz, DMSO-*d6*) δ 153.9, 142.6, 130.3 (q, J = 32.5 Hz), 123.4 (q, J = 272.6 Hz), 118.6 – 118.5 (m), 113.9 – 113.8 (m), 48.1, 15.3, 9.2. ^{19}F NMR (470 MHz, DMSO) δ -61.82. HRMS (DART/Orbitrap) calculated for $[M+H]^+$ ($C_{14}H_{13}F_6N_2O$) m/z 339.0927; found m/z 339.0923.

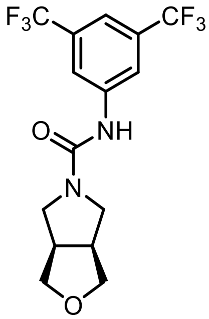

(3a*R*,6a*S*)-*N*-(3,5-bis(trifluoromethyl)phenyl)tetrahydro-1*H*-furo[3,4-*c*]pyrrole-5(3*H*)-carboxamide (S3) Prepared from (3a*R*,6a*S*)-hexahydro-1*H*-furo[3,4-*c*]pyrrole hydrochloride (898 mg, 6.0 mmol, 1.0 equiv) and 3,5-bis(trifluoromethyl)phenyl isocyanate (1.53 g, 6.0 mmol, 1.0 equiv) following GP-D. The concentrated crude product was then loaded onto a SiO_2 column and eluted with a gradient from 10% hexanes/EtOAc to 50% hexanes/EtOAc. The product was obtained as a white solid (1.30 g, 3.53 mmol, 59% yield). ^{1}H NMR (500 MHz, $CDCl_3$) δ 7.91 (s, 2H), 7.49 (s, 1H), 6.65 (s, 1H), 3.94 (dd, J = 9.1, 6.5 Hz, 2H), 3.75 (dd, J = 10.3, 7.8 Hz, 2H), 3.70 (dd, J = 9.2, 3.3 Hz, 2H), 3.44 (dd, J = 10.5, 3.3 Hz, 2H), 3.09 – 3.01 (m, 2H). ^{13}C NMR (126 MHz, $CDCl_3$) δ 153.1, 140.6, 132.2 (q, J = 33.3 Hz), 123.3 (q, J = 272.7 Hz), 119.3 – 119.1 (m), 116.3 – 116.2 (m), 73.6, 50.9, 43.7. ^{19}F NMR (470 MHz, $CDCl_3$) δ -63.04. HRMS (DART/Orbitrap) calculated for $[M+H]^+$ ($C_{15}H_{15}F_6N_2O_2$) m/z 369.1032; found m/z 369.1025.

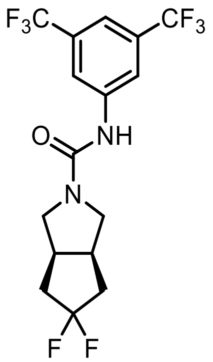

(3a*R*,6a*S*)-N-(3,5-bis(trifluoromethyl)phenyl)-5,5-difluorohexahydrocyclopenta[*c*]pyrrole-2(1*H*)-carboxamide (S4) Prepared from *tert*-butyl (3a*R*,6a*S*)-5,5-difluorohexahydrocyclopenta[*c*]pyrrole-2(1*H*)-carboxylate (989 mg, 4.0 mmol, 1.0 equiv) and 3,5-bis(trifluoromethyl)phenyl isocyanate (1.02 g, 4.0 mmol, 1.0 equiv) following GP-E. The concentrated crude product was then loaded onto a SiO_2 column and eluted with a gradient from 0% DCM/EtOAc to 30% DCM/EtOAc. The product was obtained as a white solid (1.09 g, 2.71 mmol, 68% yield. ^{1}H NMR (500 MHz, $CDCl_3$) δ 7.90 (s, 2H), 7.49 (s, 1H), 6.69 (s, 1H), 3.78 – 3.71 (m, 2H), 3.43 (dd, *J* = 10.5, 3.8 Hz, 2H), 3.01 – 2.91 (m, 2H), 2.48 – 2.38 (m, 2H), 2.12 – 2.00 (m, 2H). ^{13}C NMR (126 MHz, $CDCl_3$) δ 153.3, 140.5, 132.5 (dd, *J* = 251.3, 249.1 Hz), 132.2 (q, *J* = 33.4 Hz), 123.3 (q, *J* = 272.6 Hz), 119.4 – 119.2 (m), 116.4 – 116.3 (m), 51.2, 40.5 (dd, *J* = 25.8, 24.4 Hz), 40.0 (t, *J* = 4.0 Hz). ^{19}F NMR (470 MHz, $CDCl_3$) δ -63.07, -89.16 (dp, *J* = 232.4, 16.0 Hz), -90.05 (dddd, *J* = 232.4, 23.2, 13.2, 9.8 Hz). HRMS (DART/Orbitrap) calculated for $[M+H]^+$ ($C_{16}H_{15}F_8N_2O$) m/z 403.1051; found m/z 403.1049.

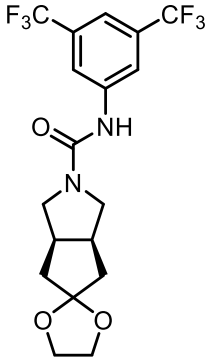

(3a*R*,6a*S*)-*N*-(3,5-bis(trifluoromethyl)phenyl)tetrahydro-1*H*-spiro[cyclopenta[*c*]pyrrole-5,2'-[1,3]dioxolane]-2(3*H*)-carboxamide (S5) Prepared from *tert*-butyl (3a*R*,6a*S*)-tetrahydro-1*H*-spiro[cyclopenta[*c*]pyrrole-5,2'-[1,3]dioxolane]-2(3*H*)-carboxylate (362 mg, 1.34 mmol, 1.0 equiv) and 3,5-bis(trifluoromethyl)phenyl isocyanate (341 g, 1.34 mmol, 1.0 equiv) following GP-E. The concentrated crude product was then loaded onto a SiO_2 column and eluted with a gradient from 0% hexanes/EtOAc to 40% hexanes/EtOAc. The product was obtained as a white solid (201 mg, 0.47 mmol, 35% yield). Compound contains traces of an unidentified impurity (^{1}H-NMR δ 2.78 (s), ^{13}C-NMR δ 38.7). ^{1}H NMR (500 MHz, $CDCl_3$) δ 7.91 (s, 2H), 7.45 (s, 1H), 6.86 (s, 1H), 3.88 (s, 4H), 3.71 – 3.65 (m, 2H), 3.42 (dd, *J* = 10.4, 3.8 Hz, 2H), 2.83 (dt, *J* = 6.2, 3.0 Hz, 2H), 2.12 (dd, *J* = 13.8, 7.8 Hz, 2H), 1.77 (dd, *J* = 14.1, 5.0 Hz, 2H). ^{13}C NMR (126 MHz, $CDCl_3$) δ 153.4, 140.9, 132.1 (q, *J* = 33.2 Hz), 123.4 (q, *J* = 272.6 Hz), 119.3 – 119.1 (m), 118.4, 116.1 – 115.8 (m), 64.8, 64.2, 51.5, 41.1, 40.6. ^{19}F NMR (470 MHz, $CDCl_3$) δ -63.09. HRMS (DART/Orbitrap) calculated for $[M+H]^+$ ($C_{18}H_{19}F_6N_2O_3$) m/z 425.1294; found m/z 425.1309.

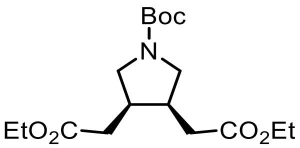

diethyl 2,2'-((3*R*,4*S*)-1-(*tert*-butoxycarbonyl)pyrrolidine-3,4-diyl)diacetate (S6)) A round-bottom flask was charged with 2,2'-((3*R*,4*S*)-1-(*tert*-butoxycarbonyl)pyrrolidine-3,4-diyl)diacetic acid (2.87 g, 10.0 mmol, 1.0 equiv), potassium carbonate (6.91 g, 50 mmol, 5.0 equiv), ethyl iodide (6.24 g, 40.0 mmol, 4.0 equiv) and DMF (20 mL). Reaction mixture was stirred for 16 h at room temperature, and then transferred to a separatory funnel. The reaction mixture was diluted with H_2O (100 mL) and extracted with DCM (3 x 50 mL), combined organic phases were dried over Na_2SO_4, concentrated, and purified by column chromatography (SiO_2, ethyl acetate in hexanes from 0% to 100%). The product was obtained as a pale-yellow oil (1.42 g, 4.1 mmol, 41%). ^{1}H NMR (500 MHz, $CDCl_3$) δ 4.17 – 4.09 (m, 4H), 3.55 – 3.46 (m, 2H), 3.18 – 3.03 (m, 2H), 2.73 – 2.65 (m, 2H), 2.42 – 2.31 (m, 2H), 2.29 – 2.17 (m, 2H), 1.46 – 1.40 (m, 9H), 1.28 – 1.22 (m, 6H). ^{13}C NMR (126 MHz, $CDCl_3$) δ 172.2, 172.1, 154.7, 79.5, 60.8, 50.3, 50.1, 37.5, 36.8, 33.3, 28.6, 14.3. HRMS (DART/Orbitrap) calculated for $[M+H]^+$ ($C_{17}H_{30}NO_6$) m/z 344.2068; found m/z 344.2062.

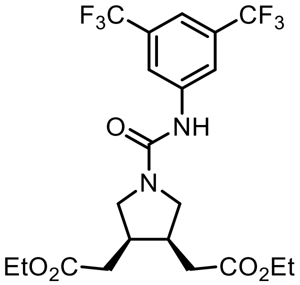

Diethyl 2,2'-((3*R*,4*S*)-1-((3,5-bis(trifluoromethyl)phenyl)carbamoyl)pyrrolidine-3,4-diyl)diacetate (S7) Prepared from diethyl 2,2'-((3*R*,4*S*)-1-(tert-butoxycarbonyl)pyrrolidine-3,4-diyl)diacetate (879 mg, 2.5 mmol, 1.0 equiv) and 3,5-bis(trifluoromethyl)phenyl isocyanate (638 mg, 2.5 mmol, 1.0 equiv) following GP-E. The concentrated crude product was then loaded onto a SiO_2 column and eluted with a gradient from 0% DCM/EtOAc to 30% DCM/EtOAc. The product was obtained as a white solid (1.10 g, 2.21 mmol, 88% yield). ^{1}H NMR (500 MHz, $CDCl_3$) δ 7.93 (s, 2H), 7.52 – 7.47 (m, 1H), 6.73 – 6.47 (m, 1H), 4.19 – 4.11 (m, 4H), 3.75 – 3.67 (m, 2H), 3.35 – 3.29 (m, 2H), 2.88 – 2.80 (m, 2H), 2.49 – 2.41 (m, 2H), 2.35 – 2.26 (m, 2H), 1.30 – 1.23 (m, 6H). ^{13}C NMR (126 MHz, $CDCl_3$) δ 171.9, 153.2, 140.6, 132.2 (q, J = 33.2 Hz), 123.3 (q, J = 272.8 Hz), 119.1, 116.2, 61.1, 50.2, 37.3, 33.1, 14.3. ^{19}F NMR (470 MHz, $CDCl_3$) δ -63.04. HRMS (DART/Orbitrap) calculated for $[M+H]^+$ ($C_{21}H_{25}F_6N_2O_5$) m/z 499.1662; found m/z 499.1653.

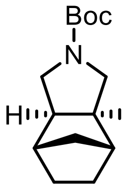

***tert*-butyl (3a*R*,4*S*,7*R*,7a*S*)-octahydro-2*H*-4,7-methanoisoindole-2-carboxylate (S8)** A round bottom flask was charged with $LiAlH_4$ (2.37 g, 62.5 mmol, 2.5 equiv) and 50 mL dry THF under nitrogen atmosphere in an ice bath. (3a*R*,4*S*,7*R*,7a*S*)-hexahydro-1*H*-4,7-methanoisoindole-1,3(2*H*)-dione (4.13 g, 25.0 mmol, 1.0 equiv) was slowly added to a vigorously stirred suspension. The reaction mixture was then refluxed for 12 h. Then the reaction vessel was placed in an ice bath and 4 mL of 9:1 THF:H_2O mixture were slowly added, followed by 4 mL of 15% NaOH aqueous solution, followed by 8 mL of H_2O. The resulting slurry was stirred for 1 h and then filtered through a pad of celite. The celite pad was then washed with DCM (3 × 30 mL), combined extracts were concentrated and the resulting amine was used in the next step without further purification. A round-bottom flask was charged with the crude amine and DCM (100 mL, 0.25 M). The reaction was placed in an ice bath and Boc_2O was added (6.55 g, 30.0 mmol, 1.2 equiv) followed by Et_3N (3.79 g, 37.5 mmol, 1.5 equiv). Then the ice bath was removed. After 1 h, imidazole (3.40 g, 50.0 mmol, 2.0 equiv) was added, and the reaction mixture was stirred for an additional 10 minutes. After that time the reaction was diluted with EtOAc (100 mL), washed with 1 M HCl (2 × 100 mL) and brine (100 mL), dried over $MgSO_4$ and concentrated. The crude product was then loaded onto a SiO_2 column and eluted with a gradient from 0% hexanes/EtOAc to 40% hexanes/EtOAc. The product was obtained as an orange oil (3.47 g, 14.62 mmol, 58% yield over 2 steps). ^1H NMR (500 MHz, $CDCl_3$) δ 3.58 – 3.49 (m, 2H), 2.98 (dd, J = 11.3, 3.4 Hz, 2H), 2.15 (ddd, J = 7.6, 3.9, 2.1 Hz, 2H), 2.06 (d, J = 1.3 Hz, 2H), 1.48 – 1.35 (m, 3H), 1.42 (s, 9H), 1.14 – 1.08 (m, 2H), 1.04 (dp, J = 10.5, 1.7 Hz, 1H). ^{13}C NMR (126 MHz, $CDCl_3$) δ 154.2, 79.0, 52.1, 46.6, 42.0, 32.6, 28.7, 28.1. HRMS (DART/Orbitrap) calculated for $[M+H]^+$ ($C_{14}H_{24}F_6NO_2$) m/z 238.1802; found m/z 238.1802.

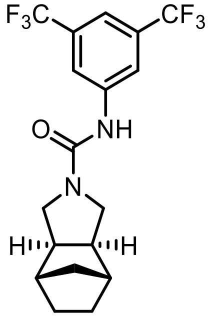

(3a*R*,4*R*,7*S*,7a*S*)-*N*-(3,5-bis(trifluoromethyl)phenyl)octahydro-2*H*-4,7-methanoisoindole-2-carboxamide (S9) Prepared from *tert*-butyl (3a*R*,4*R*,7*S*,7a*S*)-octahydro-2*H*-4,7-methanoisoindole-2-carboxylate (712 mg, 3.0 mmol, 1.0 equiv) and 3,5-bis(trifluoromethyl)phenyl isocyanate (765 mg, 3.0 mmol, 1.0 equiv) following GP-E. The concentrated crude product was then loaded onto a SiO_2 column and eluted with a gradient from 0% DCM/EtOAc to 20% DCM/EtOAc. The product was obtained as a white solid (1.03 g, 2.62 mmol, 88% yield). ^1H NMR (500 MHz, $CDCl_3$) δ 7.92 (s, 2H), 7.49 – 7.45 (m, 1H), 6.70 – 6.44 (m, 1H), 3.77 – 3.67 (m, 2H), 3.23 – 3.14 (m, 2H), 2.39 – 2.29 (m, 2H), 2.19 – 2.10 (m, 2H), 1.56 – 1.47 (m, 2H), 1.45 – 1.36 (m, 1H), 1.21 – 1.10 (m, 3H). ^{13}C NMR (126 MHz, $CDCl_3$) δ 152.7,

140.8, 132.2 (q, J = 33.1 Hz), 123.4 (q, J = 272.7 Hz), 119.1, 116.0, 52.4, 46.7, 42.3, 32.7, 28.0. ^{19}F NMR (470 MHz, $CDCl_3$) δ -63.02 – -63.08 (m). HRMS (DART/Orbitrap) calculated for $[M+H]^+$ ($C_{18}H_{19}F_6N_2O$) m/z 393.1396; found m/z 393.1390.

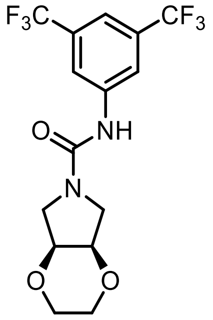

(4a*R*,7a*S*)-*N*-(3,5-bis(trifluoromethyl)phenyl)hexahydro-6*H*-[1,4]dioxino[2,3-*c*]pyrrole-6-carboxamide (S10) Prepared from *tert*-butyl (4a*R*,7a*S*)-hexahydro-6*H*-[1,4]dioxino[2,3-*c*]pyrrole-6-carboxylate (688 mg, 3.0 mmol, 1.0 equiv) and 3,5-bis(trifluoromethyl)phenyl isocyanate (765 mg, 3.0 mmol, 1.0 equiv) following GP-E. The concentrated crude product was then loaded onto a SiO_2 column and eluted with a gradient from 0% DCM/EtOAc to 40% DCM/EtOAc. The product was obtained as a white solid (1.06 g, 2.76 mmol, 92% yield). ^{1}H NMR (500 MHz, $CDCl_3$) δ 7.92 (s, 2H), 7.51 (s, 1H), 6.51 (s, 1H), 4.28 (t, J = 4.5 Hz, 2H), 3.90 – 3.83 (m, 2H), 3.74 (dd, J = 10.6, 4.6 Hz, 2H), 3.70 – 3.63 (m, 2H), 3.58 (dd, J = 10.6, 6.2 Hz, 2H). ^{13}C NMR (126 MHz, $CDCl_3$) δ 153.4, 140.5, 132.3 (q, J = 33.3 Hz), 123.3 (q, J = 272.8 Hz), 119.3 – 119.1 (m), 116.5 – 116.3 (m), 72.8, 62.6, 46.5. ^{19}F NMR (470 MHz, $CDCl_3$) δ -63.03. HRMS (DART/Orbitrap) calculated for $[M+H]^+$ ($C_{15}H_{15}F_6N_2O_3$) m/z 385.0981; found m/z 385.0975.

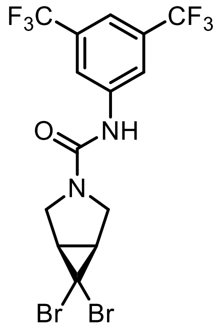

(1*R*,5*S*)-*N*-(3,5-bis(trifluoromethyl)phenyl)-6,6-dibromo-3-azabicyclo[3.1.0]hexane-3-carboxamide (S11) Prepared from *tert*-butyl (1*R*,5*S*)-6,6-dibromo-3-azabicyclo[3.1.0]hexane-3-carboxylate (853 mg, 2.5 mmol, 1.0 equiv) and 3,5-bis(trifluoromethyl)phenyl isocyanate (638 mg, 2.5 mmol, 1.0 equiv) following GP-E. The concentrated crude product was then loaded onto a SiO_2 column and eluted with a gradient from 0% DCM/EtOAc to 20% DCM/EtOAc. The product was obtained as a white solid (654 mg, 1.32 mmol, 53% yield). ^{1}H NMR (500 MHz, $CDCl_3$) δ 7.91 (s, 2H), 7.51 (s, 1H), 6.38 (s, 1H), 3.83 – 3.76 (m, 2H), 3.75 – 3.64 (m, 2H), 2.58 (dd, J = 3.7, 1.5 Hz, 2H). ^{13}C NMR (126 MHz, $CDCl_3$) δ 152.1, 140.3, 132.4 (q, J = 33.4 Hz), 123.3 (q, J = 272.7 Hz), 119.3 – 119.2 (m), 116.7 – 116.5 (m), 49.1, 35.2, 33.8. ^{19}F NMR (470 MHz, $CDCl_3$) δ -63.03. HRMS (DART/Orbitrap) calculated for $[M+H]^+$ ($C_{14}H_{11}Br_2F_6N_2O$) m/z 494.9137; found m/z 494.9130.

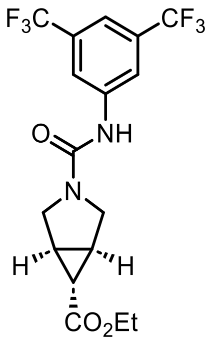

(ethyl (1*r*,5*s*,6*r*)-3-((3,5-bis(trifluoromethyl)phenyl)carbamoyl)-3-azabicyclo[3.1.0]hexane-6-carboxylate (S12) Prepared from 3-(*tert*-butyl) 6-ethyl (1*r*,5*s*,6*r*)-3-azabicyclo[3.1.0]hexane-3,6-dicarboxylate (511 mg, 2.0 mmol, 1.0 equiv) and 3,5-bis(trifluoromethyl)phenyl isocyanate (510 mg, 2.0 mmol, 1.0 equiv) following GP-E. The concentrated crude product was then loaded onto a SiO_2 column and eluted with a gradient from 0% DCM/EtOAc to 30% DCM/EtOAc. The product was obtained as a white solid (746 mg, 1.82 mmol, 91% yield). ^{1}H NMR (500 MHz, $CDCl_3$) δ 7.89 (s, 2H), 7.51 (s, 1H), 6.49 (s, 1H), 4.15 (q, *J* = 7.1 Hz, 2H), 3.77 (d, *J* = 10.1 Hz, 2H), 3.64 (ddd, *J* = 10.2, 2.5, 1.4 Hz, 2H), 2.26 – 2.21 (m, 2H), 1.57 (t, *J* = 3.2 Hz, 1H), 1.27 (t, *J* = 7.2 Hz, 3H). ^{13}C NMR (126 MHz, $CDCl_3$) δ 172.1, 153.4, 140.4, 132.3 (q, *J* = 33.3 Hz), 123.3 (q, *J* = 272.6 Hz), 119.4 – 119.2 (m), 116.6 – 116.3 (m), 61.1, 48.3, 26.2, 25.1, 14.4. ^{19}F NMR (470 MHz, $CDCl_3$) δ -63.05. HRMS (DART/Orbitrap) calculated for $[M+H]^+$ ($C_{17}H_{17}F_6N_2O_3$) m/z 411.1138; found m/z 411.1131.

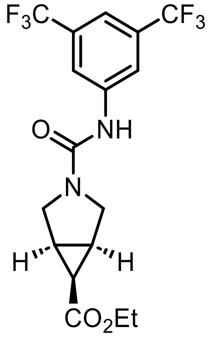

Ethyl (1*r*,5*s*,6*s*)-3-((3,5-bis(trifluoromethyl)phenyl)carbamoyl)-3-azabicyclo[3.1.0]hexane-6-carboxylate (S13) Prepared from 3-(*tert*-butyl) 6-ethyl (1*r*,5*s*,6*s*)-3-azabicyclo[3.1.0]hexane-3,6-dicarboxylate (766 mg, 3.0 mmol, 1.0 equiv) and 3,5-bis(trifluoromethyl)phenyl isocyanate (765 mg, 3.0 mmol, 1.0 equiv) following GP-E. The concentrated crude product was then loaded onto a SiO_2 column and eluted with a gradient from 0% DCM/EtOAc to 30% DCM/EtOAc. The product was obtained as a white solid (1.12 g, 2.73 mmol, 91% yield). ^{1}H NMR (500 MHz, $CDCl_3$) δ 7.91 (s, 2H), 7.48 (s, 1H), 6.44 (s, 1H), 4.10 (q, *J* = 7.1 Hz, 2H), 3.88 (d, *J* = 10.1 Hz, 2H), 3.67 (ddd, *J* = 10.2, 3.0, 1.6 Hz, 2H), 2.08 (ddd, *J* = 8.2, 3.0, 1.5 Hz, 2H), 1.88 (t, *J* = 8.2 Hz, 1H), 1.23 (t, *J* = 7.1 Hz, 3H). ^{13}C NMR (126 MHz, $CDCl_3$) δ 169.2, 152.6, 140.7, 132.2 (q, *J* = 33.3 Hz), 123.4 (q, *J* = 272.7 Hz), 119.2 – 118.9 (m), 116.2 – 116.0 (m), 61.0, 45.9, 22.6, 22.4, 14.3. ^{19}F NMR (470 MHz, $CDCl_3$) δ -63.03. HRMS (DART/Orbitrap) calculated for $[M+H]^+$ ($C_{17}H_{17}F_6N_2O_3$) m/z 411.1138; found m/z 411.1130.

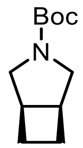

***tert*-butyl (1*R*,5*S*)-3-azabicyclo[3.2.0]heptane-3-carboxylate (S14)** Prepared based on the literature procedure[2]. 1 L measuring cylinder was charged with H_2SO_4 (4.90 g, 50.0 mmol, 1.0 equiv) and DI water (1000 mL). Then $CuSO_4$ (624 mg, 2.5 mmol, 0.05 equiv) and diallylamine (4.86 g, 50.0 mmol, 1.0 equiv) were added. The stirred reaction mixture was irradiated UV-C lamp immersed in the solution (COOSPIDER UV Light Bulb, 36 Watts UV-C Lamp) for 24 h. Then the mixture was basified with solid NaOH (20 g) and extracted with Et_2O (3 × 200 mL). The combined organic extracts were dried over Na_2SO_4 and concentrated. The crude orange oil (1.17 g) was used in the next step without further purification. A round-bottom flask was charged with the crude amine (1.17 g, assumed 12.1 mmol, 1.0 equiv) and 48.3 mL DCM (0.25 M). The reaction was placed in an ice bath and Boc_2O was added (3.16 g, 15.5 mmol, 1.2 equiv) followed by Et_3N (1.83 g, 18.1 mmol, 1.5 equiv). Then the ice bath was removed. After 1 h, imidazole (1.64 g, 24.1 mmol, 2.0 equiv) was added and the reaction mixture was stirred for an additional 10 minutes. After that time the reaction was diluted with EtOAc (100 mL), washed with 1 M HCl (2 × 100 mL) and brine (100 mL), dried over MgSO4 and evaporated. The concentrated crude product was then loaded onto a SiO_2 column and eluted with a gradient from 0% hexanes/EtOAc to 30% hexanes/EtOAc. The product was obtained as a colorless oil (1.17 g, 5.93 mmol, 12% yield over 2 steps). ^{1}H NMR (500 MHz, $CDCl_3$) δ 3.64 – 3.39 (m, 2H), 3.23 (s, 2H), 2.87 (q, J = 3.6 Hz, 2H), 2.21 – 2.13 (m, 2H), 1.77 – 1.64 (m, 2H), 1.48 (s, 9H). ^{13}C NMR (126 MHz, $CDCl_3$) δ 155.5, 79.2, 53.3, 52.9, 38.2, 37.3, 28.7, 24.7. HRMS (DART/Orbitrap) calculated for $[M+H]^+$ ($C_{11}H_{20}NO_2$) m/z 198.1489; found m/z 198.1487.

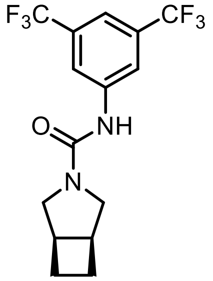

(1*R*,5*S*)-N-(3,5-bis(trifluoromethyl)phenyl)-3-azabicyclo[3.2.0]heptane-3-carboxamide (S15) Prepared from tert-butyl (1*R*,5*S*)-3-azabicyclo[3.2.0]heptane-3-carboxylate (395 mg, 2.0 mmol, 1.0 equiv) and 3,5-bis(trifluoromethyl)phenyl isocyanate (510 mg, 2.0 mmol, 1.0 equiv) following GP-E. The concentrated crude product was then loaded onto a SiO_2 column and eluted with a gradient from 0% DCM/EtOAc to 20% DCM/EtOAc. The product was obtained as a white solid (684 mg, 1.94 mmol, 97% yield). ^{1}H NMR (500 MHz, $CDCl_3$) δ 7.96 (s, 2H), 7.49 (s, 1H), 6.73 (s, 1H), 3.63 (d, J = 10.5 Hz, 2H), 3.51 – 3.42 (m, 2H), 3.09 – 3.01 (m, 2H), 2.31 – 2.24 (m, 2H), 1.82 – 1.76 (m, 2H). ^{13}C NMR (126 MHz, $CDCl_3$) δ 154.0, 140.9, 132.2 (q, J = 33.2 Hz), 123.4 (q, J = 272.7 Hz), 119.3 – 119.1 (m), 116.2 – 116.0 (m), 53.4, 37.9, 24.9. ^{19}F NMR (470 MHz, $CDCl_3$) δ -63.04. HRMS (DART/Orbitrap) calculated for $[M+H]^+$ ($C_{15}H_{15}F_6N_2O$) m/z 353.1083; found m/z 353.1077.

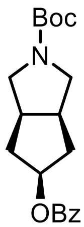

tert-butyl (3a*R*,5*r*,6a*S*)-5-(benzoyloxy)hexahydrocyclopenta[*c*]pyrrole-2(1*H*)-carboxylate (S16) tert-butyl (3a*R*,6a*S*)-5-oxohexahydrocyclopenta[*c*]pyrrole-2(1*H*)-carboxylate (2.70 g, 12.0 mmol, 1.0 equiv) was placed in a 100 mL round-bottom flask, dissolved in MeOH (30 mL, 0.4 M) and placed in an ice bath. $NaBH_4$ (545 mg, 14.4 mmol, 1.2 equiv) was added portion wise to a vigorously stirred solution. After the addition was complete, the ice bath was removed and the reaction mixture was slowly warmed up to room temperature. After 1 h, NH_4Cl (100 mL, 10% *w*/*v*) was added to the stirred solution. Most of the methanol was evaporated on the rotary evaporator, and after that the reaction mixture was transferred into the separatory funnel. The aqueous phase was extracted with DCM (3 × 100 mL). The combined organic extracts were dried over $MgSO_4$, concentrated, and dried on the high vacuum. Obtained crude alcohol was used in the next step without further purification. The crude alcohol was placed in 100 mL round-bottom flask, dissolved in DCM (24 mL, 0.5 M) and placed in an ice-bath. NEt_3 (2.43 g, 24.0 mmol, 2.0 equiv) was added, followed by dropwise addition of benzoyl chloride (2.02 g, 14.4 mmol, 1.2 equiv) to a vigorously stirred solution. The ice-bath was removed and the reaction was stirred for 1 h. Then the reaction mixture was transferred to the separatory funnel and $NaHCO_3$ (100 mL, sat.) was added. The reaction mixture was extracted with DCM (3 × 50 mL). The combined organic phases were dried over Na_2SO_4 and concentrated. The crude product was then loaded onto a SiO_2 column and eluted with a gradient from 0% hexanes/EtOAc to 40% hexanes/EtOAc. The product was obtained as a pale-yellow oil (2.53 g, 7.63 mmol, 64% yield over 2 steps, *d.r.* 20:1). ^{1}H NMR (500 MHz, $CDCl_3$) δ 7.99 (d, *J* = 7.3 Hz, 2H), 7.58 – 7.52 (m, 1H), 7.45 – 7.40 (m, 2H), 5.42 (tt, *J* = 6.5, 4.9 Hz, 1H), 3.69 – 3.53 (m, 2H), 3.47 – 3.30 (m, 2H), 2.81 – 2.68 (m, 2H), 2.39 – 2.29 (m, 2H), 1.85 – 1.74 (m, 2H), 1.44 (s, 9H). ^{13}C NMR (126 MHz, $CDCl_3$) δ 166.4, 154.7, 133.1, 130.5, 129.7, 128.5, 79.3, 77.8, 52.3, 52.1, 41.9, 41.0, 38.2, 28.7. HRMS (DART/Orbitrap) calculated for $[M+H]^+$ ($C_{19}H_{26}NO_4$) m/z 332.1856; found m/z 332.1851.

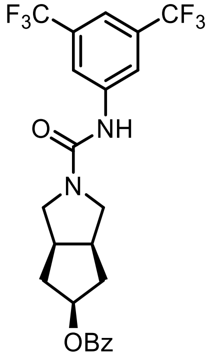

(3a*R*,5*r*,6a*S*)-2-((3,5-bis(trifluoromethyl)phenyl)carbamoyl)octahydrocyclopenta[*c*]pyrrol-5-yl benzoate (S17) Prepared from *tert*-butyl (3a*R*,5*r*,6a*S*)-5-(benzoyloxy)hexahydrocyclopenta[*c*]pyrrole-2(1*H*)-carboxylate (997 mg, 3.0 mmol, 1.0 equiv) and 3,5-bis(trifluoromethyl)phenyl isocyanate (765 mg, 3.0 mmol, 1.0 equiv) following GP-E. The concentrated crude product was then loaded onto a SiO_2 column and eluted with a gradient from

0% DCM/EtOAc to 20% DCM/EtOAc. The product was obtained as a white solid (1.08 g, 2.22 mmol, 74% yield). ^{1}H NMR (500 MHz, $CDCl_3$) δ 8.00 – 7.93 (m, 2H), 7.88 (s, 2H), 7.51 – 7.45 (m, 2H), 7.33 (t, *J* = 7.9 Hz, 2H), 6.46 (s, 1H), 5.47 (tt, *J* = 6.5, 3.9 Hz, 1H), 3.76 (dd, *J* = 10.2, 8.1 Hz, 2H), 3.57 (dd, *J* = 10.4, 3.5 Hz, 2H), 2.94 (qd, *J* = 8.1, 4.1 Hz, 2H), 2.40 (ddd, *J* = 14.3, 8.0, 6.1 Hz, 2H), 1.92 (dt, *J* = 14.7, 3.8 Hz, 2H). ^{13}C NMR (126 MHz, $CDCl_3$) δ 166.3, 153.0, 140.7, 133.3, 132.2 (q, *J* = 33.3 Hz), 130.2, 129.6, 128.6, 123.4 (q, *J* = 272.9 Hz), 119.2 – 118.0 (m), 116.2 – 116.0 (m), 77.8, 52.6, 41.9, 38.7. ^{19}F NMR (470 MHz, $CDCl_3$) δ -63.02. HRMS (DART/Orbitrap) calculated for $[M+H]^+$ ($C_{23}H_{21}F_6N_2O_3$) m/z 487.1451; found m/z 487.1441.

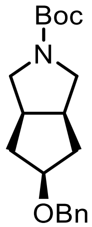

***tert*-butyl (3a*R*,5*r*,6a*S*)-5-(benzyloxy)hexahydrocyclopenta[c]pyrrole-2(1*H*)-carboxylate (S18)** *tert*-butyl (3a*R*,6a*S*)-5-oxohexahydrocyclopenta[*c*]pyrrole-2(1*H*)-carboxylate (2.70 g, 12.0 mmol, 1.0 equiv) was placed in a 100 mL round-bottom flask, dissolved in MeOH (30 mL, 0.4M) and placed in an ice bath. $NaBH_4$ (545 mg, 14.4 mmol, 1.2 equiv) was added portion wise to a vigorously stirred solution. After the addition was complete, the ice bath was removed and the reaction mixture was slowly warmed up to room temperature. After 1 h, NH_4Cl (100 mL, 10% *w*/*v*) was added to the stirred solution. Most of the MeOH was evaporated on the rotary evaporator, and the reaction mixture was transferred into a separatory funnel. The aqueous phase was extracted with DCM (3 × 100 mL). The combined organic extracts were dried over $MgSO_4$, concentrated, and dried on the high vacuum. The crude alcohol was used in the next step without further purification. The crude alcohol was placed in a 100 mL round-bottom flask under nitrogen atmosphere, dissolved in THF (24 mL, 0.5 M), and placed in an ice-bath. NaH (720 mg, 18.0 mmol, 1.5 equiv, 60% dispersion in mineral oil) was added portion wise. After 5 minutes, benzyl bromide (3.08 g, 18.0 mmol, 1.5 equiv) was added and the ice-bath was removed. After 6 h the reaction was quenched by addition of $NaHCO_3$ (100 mL, sat.) and transferred to the separatory funnel. The reaction mixture was extracted with DCM (3 × 50 mL). The combined organic extracts were dried over Na_2SO_4 and concentrated. The crude product was then loaded onto a SiO_2 column and eluted with a gradient from 0% hexanes/EtOAc to 40% hexanes/EtOAc. The product was obtained as a pale-yellow oil (2.43 g, 7.66 mmol, 64% yield over 2 steps, *d.r.* 20:1). ^{1}H NMR (500 MHz, $CDCl_3$) δ 7.36 – 7.24 (m, 5H), 4.47 (s, 1H), 4.02 (p, *J* = 6.5 Hz, 1H), 3.53 (br. s, 2H), 3.43 – 3.24 (m, 2H), 2.57 (br. s, 2H), 2.14 (br. s, 2H), 1.66 – 1.58 (m, 2H), 1.45 (s, 9H). ^{13}C NMR (126 MHz, $CDCl_3$) δ 154.7, 138.7, 128.5, 127.6, 127.6, 81.6, 79.1, 71.4, 52.2, 51.8, 41.3, 40.5, 37.7, 28.6. HRMS (DART/Orbitrap) calculated for $[M+H]^+$ ($C_{19}H_{28}NO_3$) m/z 318.2064; found m/z 318.2058.

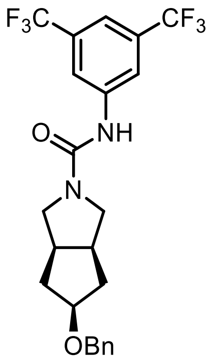

(3a*R*,5*r*,6a*S*)-5-(benzyloxy)-N-(3,5-bis(trifluoromethyl)phenyl)hexahydrocyclopenta[*c*]pyrrole-2(1*H*)-carboxamide (S19) Prepared from *tert*-butyl (3a*R*,5*r*,6a*S*)-5-(benzyloxy)hexahydrocyclopenta[*c*]pyrrole-2(1*H*)-carboxylate (952 mg, 3.0 mmol, 1.0 equiv) and 3,5-bis(trifluoromethyl)phenyl isocyanate (765 mg, 3.0 mmol, 1.0 equiv) following GP-E. The concentrated crude product was then loaded onto a SiO_2 column and eluted with a gradient from 0% DCM/EtOAc to 20% DCM/EtOAc. The product was obtained as a pale-yellow oil (1.10 g, 2.33 mmol, 78% yield). ^{1}H NMR (500 MHz, $CDCl_3$) δ 7.89 (s, 2H), 7.47 (s, 1H), 7.31 – 7.19 (m, 5H), 6.64 – 6.52 (m, 1H), 4.46 (s, 2H), 4.10 (tt, *J* = 6.4, 4.7 Hz, 1H), 3.67 (dd, *J* = 10.0, 7.9 Hz, 2H), 3.52 (dd, *J* = 10.4, 3.6 Hz, 2H), 2.80 – 2.71 (m, 2H), 2.16 (ddd, *J* = 14.0, 7.8, 6.1 Hz, 2H), 1.75 (dt, *J* = 14.2, 4.6 Hz, 2H). ^{13}C NMR (126 MHz, $CDCl_3$) δ 153.2, 140.9, 138.5, 132.1 (q, *J* = 33.4 Hz), 128.5, 127.7, 127.6, 123.4 (q, *J* = 272.7 Hz), 119.1 – 118.9 (m), 115.9 – 115.7 (m), 81.7, 71.3, 52.3, 41.7, 38.0 (d, *J* = 1.2 Hz). ^{19}F NMR (470 MHz, $CDCl_3$) δ -63.00. HRMS (DART/Orbitrap) calculated for $[M+H]^+$ ($C_{23}H_{23}F_6N_2O_2$) m/z 473.1658; found m/z 473.1649.

6. Product Derivatization

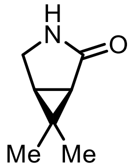

(1*R*,5*S*)-6,6-dimethyl-3-azabicyclo[3.1.0]hexan-2-one (23) (1*R*,5*S*)-*N*-(3,5-bis(trifluoromethyl)phenyl)-6,6-dimethyl-2-oxo-3-azabicyclo[3.1.0]hexane-3-carboxamide (38.0 mg, 0.1 mmol, 1.0 equiv), Boc_2O (43.7 mg, 0.2 mmol, 2.0 equiv) and DMAP (2.4 mg, 0.02 mmol, 20 mol%) were placed in a round-bottom flask under nitrogen atmosphere. Dry THF (1.0 mL) was added and the reaction mixture was stirred for 1 h at room temperature. Then LiOH (2.0 mmol, 10.0 equiv) and H_2O (0.5 mL) were added, and the reaction mixture was stirred for an additional 1 h. Then the solvent was evaporated followed by azeotropic evaporation with toluene to remove traces of water. The crude reaction mixture was purified by column chromatography (SiO_2) from 50% to 100% ethyl acetate in hexanes followed by 0% to 10% methanol in ethyl acetate as eluent. Product was obtained as a white solid (8.1 mg, 0.065 mmol, 65%). $[\alpha]_D^{20}$ −60 (*c* 0.50, $CHCl_3$). ^{1}H NMR (500 MHz, $CDCl_3$) δ 5.56 (s, 1H), 3.48 (dd, *J* = 10.7, 6.3 Hz, 1H), 3.20 (d, *J* = 10.7 Hz, 1H), 1.76 – 1.66 (m, 2H), 1.12 (s, 3H), 1.12 (s, 3H). ^{13}C NMR (126 MHz, $CDCl_3$) δ 176.3, 41.1, 32.3, 27.6, 26.0, 21.2, 13.9. HRMS (DART/Orbitrap) calculated for $[M+H]^+$ ($C_7H_{12}NO$) m/z 126.0913; found m/z 126.0915.

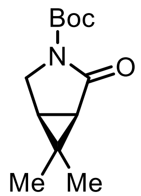

***tert*-butyl (1*R*,5*S*)-6,6-dimethyl-2-oxo-3-azabicyclo[3.1.0]hexane-3-carboxylate (24)** (1*R*,5*S*)-*N*-(3,5-bis(trifluoromethyl)phenyl)-6,6-dimethyl-2-oxo-3-azabicyclo[3.1.0]hexane-3-carboxamide (76.1 mg, 0.2 mmol, 1.0 equiv), Boc_2O (87.3 mg, 0.4 mmol, 2.0 equiv) and DMAP (4.9 mg, 0.04 mmol, 20 mol%) were placed in a round-bottom flask under nitrogen atmosphere. Dry THF (1.0 mL) was added, and the reaction mixture was stirred for 1 h at room temperature. Then ^{t}BuOLi (1.0 mmol, 5.0 equiv, 1 mL, 1.0M in THF) was added, and the reaction mixture was stirred for an additional 1 h. Then the reaction mixture was diluted with $NaHCO_3$ (50 mL) and extracted with DCM (3 × 20 mL). The combined organic phases were dried over Na_2SO_4, concentrated and purified by column chromatography (SiO_2) from 0% to 50% ethyl acetate in hexanes as eluent. The product was obtained as a white solid (24.4 mg, 0.108 mmol, 54%). $[\alpha]_D^{20}$ −62 (*c* 1.0, $CHCl_3$). ^{1}H NMR (500 MHz, $CDCl_3$) δ 3.80 (dd, *J* = 11.8, 6.6 Hz, 1H), 3.58 (dt, *J* = 11.8, 1.2 Hz, 1H), 1.87 (dd, *J* = 6.5, 1.5 Hz, 1H), 1.66 (td, *J* = 6.6, 0.9 Hz, 1H), 1.49 (s, 9H), 1.12 (s, 3H), 1.11 (s, 3H). ^{13}C NMR (126 MHz, $CDCl_3$) δ 172.2, 150.0, 82.8, 45.4, 34.5, 28.2, 26.0, 24.0, 23.1, 14.2. Data in accordance with the literature reports.[3]

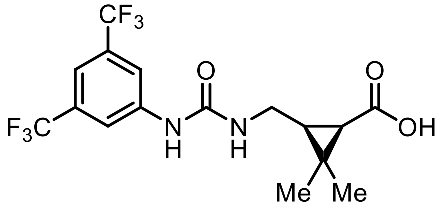

(1*R*,3*S*)-3-((3-(3,5-bis(trifluoromethyl)phenyl)ureido)methyl)-2,2-dimethylcyclopropane-1-carboxylic acid (25) (1*R*,5*S*)-*N*-(3,5-bis(trifluoromethyl)phenyl)-6,6-dimethyl-2-oxo-3-azabicyclo[3.1.0]hexane-3-carboxamide (38.0 mg, 0.1 mmol, 1.0 equiv) and LiOH•H_2O (16.8 mg, 0.4 mmol, 4.0 equiv) were placed in a round-bottom flask. Solvent was added (2 mL of dioxane and 1 mL of H_2O) and the reaction mixture was refluxed for 1 h. The reaction mixture was then transferred to a separatory funnel, diluted with aqueous HCl (50 mL, 1.0 M), and extracted with DCM (3 × 20 mL). The combined organic extracts were dried over Na_2SO_4, concentrated and purified by column chromatography (SiO_2) eluting from 0% to 100% ethyl acetate in hexanes. Obtained white solid (31.1 mg, 0.078 mmol, 78%). $[\alpha]_D^{20}$ −120 (*c* 0.50, $CHCl_3$). ^{1}H NMR (500 MHz, $CDCl_3$) δ 8.89 (s, 1H), 7.78 (s, 2H), 7.44 (s, 1H), 6.41 (s, 1H), 3.72 – 3.55 (m, 2H), 1.44 (d, *J* = 8.2 Hz, 1H), 1.36 (q, *J* = 7.5 Hz, 1H), 1.21 (s, 3H), 1.13 (s, 3H). ^{13}C NMR (126 MHz, $CDCl_3$) δ 178.9, 156.7, 140.8, 132.3 (q, *J* = 33.0 Hz), 123.3 (q, *J* = 272.7 Hz), 118.8 – 118.6 (m), 116.0 – 115.8 (m), 35.0, 34.4, 28.6, 28.3, 27.2, 14.2. ^{19}F NMR (470 MHz, $CDCl_3$) δ -63.39. HRMS (DART/Orbitrap) calculated for $[M+H]^+$ ($C_{16}H_{17}F_6N_2O_3$) m/z 399.1138; found m/z 399.1161.

7. Mechanistic Study:

The mechanistic studies focused on gaining structural insights into the mode of enantioinduction. The reaction mechanism for the racemic transformation was studied in detail in previous works.[3,4]

7.1. Isolation and Characterization of Oxoammonium $P2^+NO_3^-$

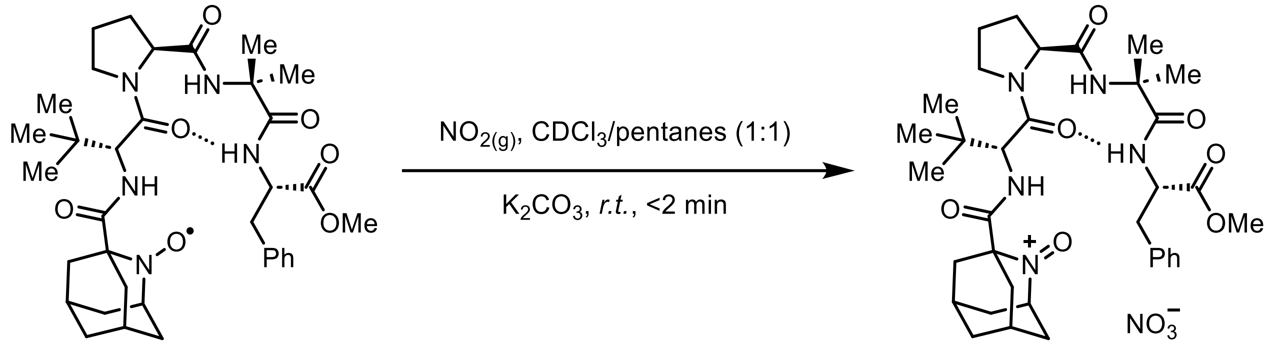

P2 **$P2^+NO_3^-$**

Procedure: *Generation of the NO_2 gas (standard operating procedures for the handling of toxic gases and concentrated nitric acid must be followed): A 150 mL Erlenmeyer flask was loaded with copper wire (approximately 10 g). 5 mL of concentrated nitric acid was added in one portion. The flask was covered loosely with aluminum foil. After 15–30 seconds, the brown NO_2 gas displaced the air in the Erlenmeyer flask, and the gas was collected in a 30 mL syringe. To remove water vapor, the needle was capped with a rubber septum, and the syringe was placed in a freezer for 5 min (to condense the water vapor; the NO_2 gas also condensed to provide a green/blue liquid). After letting the syringe come to room temperature, the gas passed through 2 cm of polypropylene tubing charged with powdered $MgSO_4$.*

In a 1-dram vial equipped with a magnetic stir bar, aminoxyl radical (**P2**, 19.6 mg, 0.03 mmol, 1 equiv) was dissolved in 1 mL of $CDCl_3$/*n*-pentane (1:1). Note: This solvent mixture ensures that aminoxyl radical **P2** and the corresponding hydroxylamine are soluble, while the oxoammonium **$P2^+NO_3^-$** has a diminished solubility. Anhydrous K_2CO_3 (41.5 mg, 0.3 mmol, 10 equiv) was added. The suspension was vigorously stirred, and NO_2 gas (30 mL, approximately 1.3 mmol) was bubbled into the solution over the course of 2 min. To collect/coagulate the orange precipitate that formed upon addition of the gas, the solution was sonicated and stirred until the orange solid coated the K_2CO_3, and the solution was colorless. The solution was decanted, and the precipitate was washed with $CDCl_3$/*n*-pentane (1:2, 3 × 1 mL). The oxoammonium salt was then collected with 1 mL of $CDCl_3$ providing a deep orange solution that was filtered through a 0.2 µm PTFE syringe filter. Note: The color of the solution is much more intense than the color of starting material solution. The solution is then concentrated to provide the oxoammonium as an orange solid (15.7 mg, 0.022 mmol, 73% yield).

The solid and its solution ($CDCl_3$ or CD_2Cl_2) were found to be stable for 7 days on the bench without a change in physical appearance or ^1H shifts. *Note*: The reaction was run 5 times with scales of 5–30 mg **$P2^+NO_3^-$**, and the yield varied between 57–82% due to challenges with washes

and filtration at sub-mL solution volumes. We found that while all ^{1}H shifts and integrals remained unchanged across different batches, the peak broadness of ^{1}H on the AzcH core varied sample by sample, due to trace radical contaminants. This is due to paramagnetic relaxation driven by rapid electron transfer with trace aminoxyl radical is well documented for related oxoammoniums.[5]

Characterization: The peptide oxoammonium was isolated as an orange solid. ^{1}H NMR (500 MHz, $CDCl_3$) δ 7.66 (d, J = 6.7 Hz, 1H), 7.62 (s, 1H), 7.28 (m, 2H), 7.23 (t, J = 7.2 Hz, 1H), 7.16 (d, J = 7.8 Hz, 1H), 7.12 (d, J = 6.7 Hz, 2H), 5.47 (br. s, 1H), 4.71 (q, J = 6.7 Hz, 1H), 4.47 (m, 2H), 3.86 – 3.78 (m, 1H), 3.67 (s, 3H), 3.61 (q, J = 8.1 Hz, 1H), 3.22 (m, 2H), 3.10 (ddt, J = 20.3, 14.0, 6.2 Hz, 2H), 3.06 (m, 1H), 3.04 – 2.82 (m, 4H), 2.77 (m, 1H), 2.65 (s, 1H), 2.52 (s, 1H), 2.44 (d, J = 13.5 Hz, 1H), 2.36 (d, J = 14.1 Hz, 1H), 2.19 – 1.91 (m, 4H), 1.49 (s, 3H), 1.47 (s, 3H), 1.03 (s, 9H). The reported number of protons was based on integral areas in an additional sample in CD_2Cl_2 and 2D HSQC. ^{13}C NMR (126 MHz, CD_2Cl_2) δ 175.2, 172.9, 172.4, 170.4, 162.4, 136.9, 129.8, 129.1, 127.5, 105.3, 98.9, 61.4, 60.0, 57.8, 54.6, 54.4, 52.9, 49.1, 38.2, 35.6, 29.5, 26.6, 25.4, 25.1. HRMS (DART/Orbitrap) calculated for $[M]^+$ ($C_{35}H_{50}N_5O_7$) m/z 652.3705; found m/z 652.3695. Due to the short relaxation times and line broadening ^{13}C signals corresponding AzcH core could not be determined for all positions.

In addition, 2D ROESY experiments were conducted to determine intramolecular upper bound distances based on the integration of ROESY crosspeaks and well-established procedures.[6] The methylene units within the proline residue were used as a distance reference (d = 1.78 Å). We found that no single conformation could provide the array of distance upper bounds observed, suggesting the presence of conformational mobility on the experimental timescale or that the analysis of the ROESY NMR is impeded by the presence residual aminoxyl radical (4% aminoxyl radical as indicated by open-circuit potential measurements).

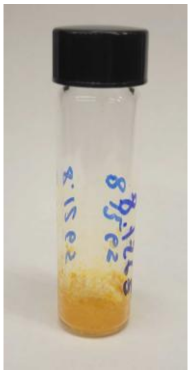

Figure S1. Oxoammonium $\mathbf{P2^{+}NO_3^{-}}$ in a 1-dram vial.

Table S1. Intramolecular experimental upper bound distances based on integration of ROESY cross peaks.

	7.38 (s, 1H)	7.28 (t, J = 7.3 Hz, 2H)	7.23 (t, J = 7.1 Hz, 1H)	7.17 (d, J = 6.9 Hz, 1H)	7.15 (d, J = 7.4 Hz, 2H)	4.67 (q, J = 6.6 Hz, 1H)	4.47 (s, 1H)	4.41 (t, J = 5.8 Hz, 1H)	3.81 (ddd, J = 11.7, 7.4, 4.4 Hz, 1H)	3.66 (s, 3H)	3.65 – 3.58 (m, 1H)	3.14 (dd, J = 13.9, 6.2 Hz, 1H)	3.06 (dd, J = 14.0, 6.6 Hz, 1H)	2.09 (q, J = 7.2 Hz, 2H)	2.05 – 1.92 (m, 2H)	1.46 (s, 3H)	1.44 (s, 4H)	1.03 (s, 9H)
1.03 (s, 9H)							2.6	3.4	3.8		2.6	4.2	4.2	4.0	3.5			
1.44 (s, 4H)	2.8			2.7	z	4.1		4.0										
1.46 (s, 3H)	2.8			2.9	3.9	4.2		3.8										
2.05 – 1.92 (m, 2H)	3.6																	3.5
2.09 (q, J = 7.2 Hz, 2H)	3.4	4.5																4.0
3.06 (dd, J = 14.0, 6.6 Hz, 1H)				2.9	2.7	2.5												4.2
3.14 (dd, J = 13.9, 6.2 Hz, 1H)				3.0	2.7	2.5												4.2
3.65 – 3.58 (m, 1H)							2.2	3.0	**1.8**									2.6
3.66 (s, 3H)						4.2												
3.81 (ddd, J = 11.7, 7.4, 4.4 Hz, 1H)	3.2					3.5	2.2				**1.8**							3.8
4.41 (t, J = 5.8 Hz, 1H)	2.3			3.4							3.0					3.8	4.0	3.4
4.47 (s, 1H)									2.2		2.2							2.6
4.67 (q, J = 6.6 Hz, 1H)				2.7	2.9				3.5	4.2		2.5	2.5			4.2	4.1	
7.15 (d, J = 7.4 Hz, 2H)						2.9						2.7	2.7			3.9	3.6	
7.17 (d, J = 6.9 Hz, 1H)	3.5					2.7		3.4				3.0	2.9			2.9	2.7	
7.23 (t, J = 7.1 Hz, 1H)																		
7.28 (t, J = 7.3 Hz, 2H)														4.5				
7.38 (s, 1H)				3.5				2.3	3.2					3.4	3.6	2.8	2.8	

distance upper bounds [Å]; the reference distance was marked in bold.

Electroanalytical Characterization: 1 mM of solutions of oxoammonium-peptide **P2$^+$NO$_3^-$** and aminoxyl radical **P2** in 0.1 M tetrabutylammonium hexafluorophosphate in MeCN, were subjected to open-circuit potential (OCP) measurements. Cyclic voltammograms of each solution were recorded starting at the open-circuit potential and scanning towards the reductive and oxidative direction in the forward scan respectively. The OCP of the **P2$^+$NO$_3^-$** solution was 513 mV vs. $Fc^{+/0}$. The oxoammonium cation provided a reversible cyclic voltammogram with $E_{p,red}$ = 477 mV, $E_{p,ox}$ = 383 mV, and $E_{1/2}$ = 430 mV vs. $Fc^{+/0}$. The OCP of the **P2** solution was 53 mV vs. $Fc^{+/0}$. The aminoxyl radical provided a reversible CV with $E_{p,ox}$ = 510 mV, $E_{p,red}$ = 371 mV, $E_{1/2}$ = 430 mV vs. $Fc^{+/0}$.

Based on the Nernst equation (here: $OCP = E_{1/2} - 59.2 \log([aminoxyl]/[oxoammonium])$ mV) the ratio of oxoammonium to aminoxyl radical in the analyte was calculated. The **P2$^+$NO$_3^-$** contained 96% oxoammonium, whereas the aminoxyl-peptide starting material **P2** contained <10^{-4} % oxoammonium. While the $E_{1/2}$ of **P2$^+$NO$_3^-$** and **P2** are not identical they show good agreement; we hypothesize that the differences in oxidation potential could be due to the presence of hydroxylamine impurities in the aminoxyl radical catalyst.

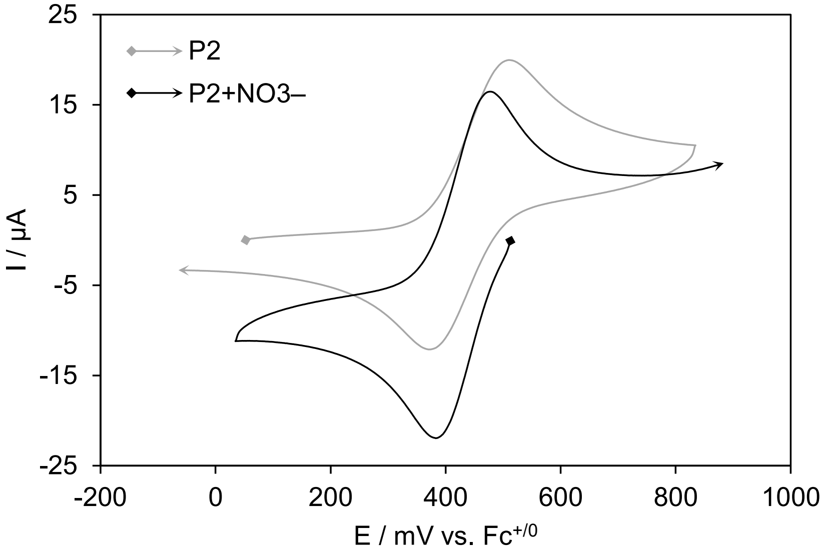

Figure S2. Cyclic voltammograms of **P2** and **P2$^+$NO$_3^-$**.

7.2. Preparation, Characterization, and Reactivity of P2·sub

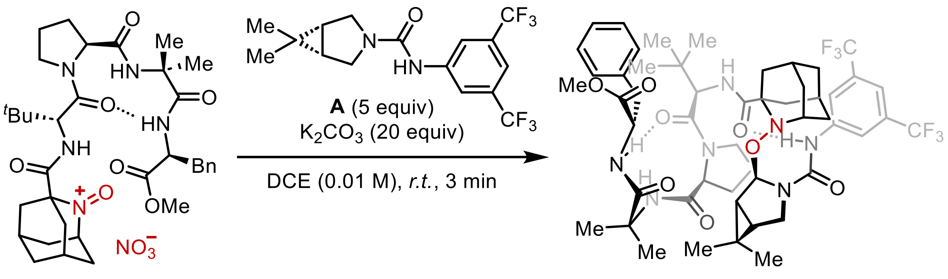

P2$^+$NO$_3^-$ **P2·sub**, 69% yield, 91:6:3 *d.r.*

Procedure: P2$^+$NO$_3^-$ (15.7 mg, 0.022 mmol, 1 equiv) was dissolved in 2 mL of DCE. **A** (42.2 mg, 0.11 mmol, 5 equiv) and potassium carbonate (60.7 mg, 0.44 mmol, 20 equiv) were dissolved in 8 mL DCE and vigorously stirred for 20 min (to make sure the potassium carbonate was a fine suspension). The oxoammonium salt solution was added dropwise over the course of 1 min. After 2 min of addition stirring the solution was diluted with 10 mL of hexanes and loaded onto a silica gel column. The product was eluted with a gradient from 0% to 50% EtOAc in hexanes. The product containing fractions as indicated by UV absorption upon TLC analysis were concentrated to provide **P2·sub** as a colorless solid (15.5 mg, 0.015 mmol, 69% yield).

Characterization: The product was obtained in 91:6:3 *d.r.* as established by quantitative ^{1}H NMR analysis by integration of the well-resolved characteristic N,O-ketal proton at 5.59 ppm, 5.51 ppm, and 5.56 ppm respectively. NMR data and a full assignment based on 2D NMR analysis (COSY, HSQC, HMBC, ROESY) is provided for the major diastereomer; proton numbers and coupling constants were determined through analysis of the 2D NMR data for overlapping peaks. ^{1}H NMR (599 MHz, CD_2Cl_2) δ 10.84 (s, 1H), 8.32 – 8.31 (m, 2H), 7.41 (s, 1H), 7.30 – 7.26 (m, 2H), 7.25 – 7.22 (m, 1H), 7.18 – 7.16 (m, 2H), 7.15 (d, J = 8.1 Hz, 1H), 6.66 (s, 1H), 6.42 (d, J = 9.5 Hz, 1H), 5.55 (s, 1H), 4.90 (d, J = 9.5 Hz, 1H), 4.74 – 4.69 (m, 1H), 4.29 (dd, J = 7.9, 3.8 k.38 (dd, J = 11.6, 4.9 Hz, 1H), 3.21 (s, 1H), 3.15 (dd, J = 13.7, 6.6 Hz, 1H), 3.03 (dd, J = 13.7, 6.5 Hz, 1H), 2.37 (d, J = 12.2 Hz, 1H), 2.22 (d, J = 13.7 Hz, 1H), 2.17 – 2.11 (m, 1H) , 2.12 – 2.06 (m, 1H) , 2.07 – 1.99 (m, 1H), 2.04 (d, J = 12.4 Hz, 1H), 1.98 – 1.93 (m, 1H), 1.91 (s, 1H), 1.86 (m, 1H), 1.82 (d, J = 12.5 Hz, 1H), 1.71 (d, J = 12.1 Hz, 1H), 1.69 (s, 2H), 1.61 (d, J = 12.4 Hz, 1H), 1.61 (d, J = 11.8 Hz, 1H), 1.46 (s, 3H), 1.43 (s, 3H), 1.40 (d, J = 7.0 Hz, 1H), 1.32 (dd, J = 7.1, 4.9 Hz, 1H), 1.27 (dd, J = 13.8 Hz, 1H), 1.05 (s, 9H), 0.95 (s, 3H), 0.82 (s, 3H). ^{13}C NMR (126 MHz, CD_2Cl_2) δ 175.21, 173.95, 172.36, 170.93, 170.76, 154.86, 142.98, 137.01, 131.93 (q, J = 32.7 Hz), 129.77, 128.77, 127.25, 124.06 (d, J = 272.5 Hz), 118.23, 114.50, 90.06, 64.63, 61.02, 57.74, 57.58, 57.11, 54.17, 52.43, 48.84, 44.77, 42.10, 38.57, 36.67, 32.63, 30.95, 30.09, 28.72, 28.70, 26.83, 26.72, 26.51, 26.48, 26.09, 25.55, 25.29, 25.21, 18.05, 14.40, 13.28. ^{19}F NMR (470 MHz, CD_2Cl_2) δ -63.37. The N,O-ketal fragments upon ionization to provide the iminium cation and hydroxylammonium cation: HRMS (DART/Orbitrap) calculated for [iminium]$^+$ ($C_{16}H_{15}N_2OF_6$) m/z 365.1083; found m/z 365.1083. HRMS (DART/Orbitrap) calculated for [hydroxylammonium]$^+$ ($C_{27}H_{53}N_5O_7$) m/z 654.3860; found m/z 654.3857.

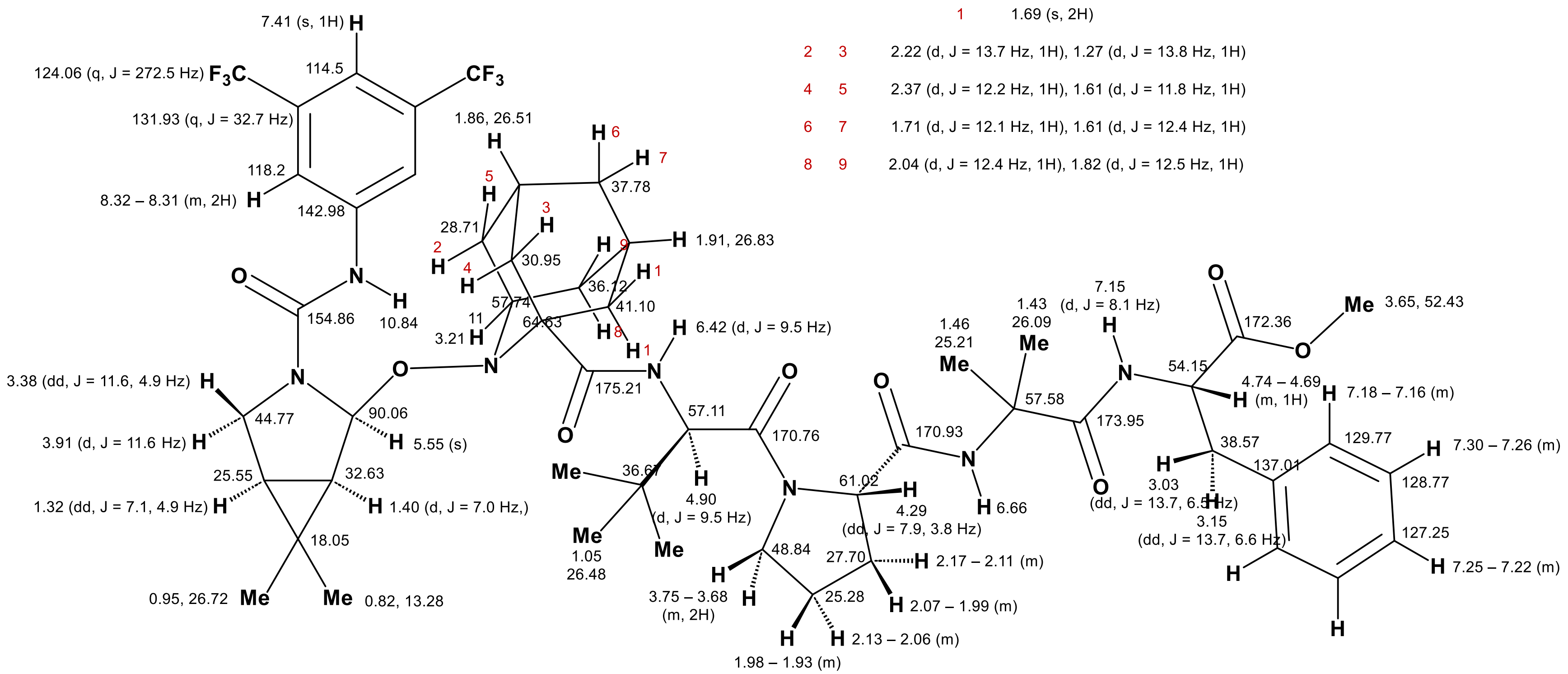

Figure S3. Fully assigned structure for the major enantiomer of **P2·sub**. Diastereotopic protons were assigned based on analysis of the ROESY spectrum (see section "7.3. ROESY analysis of P2·sub" for details).

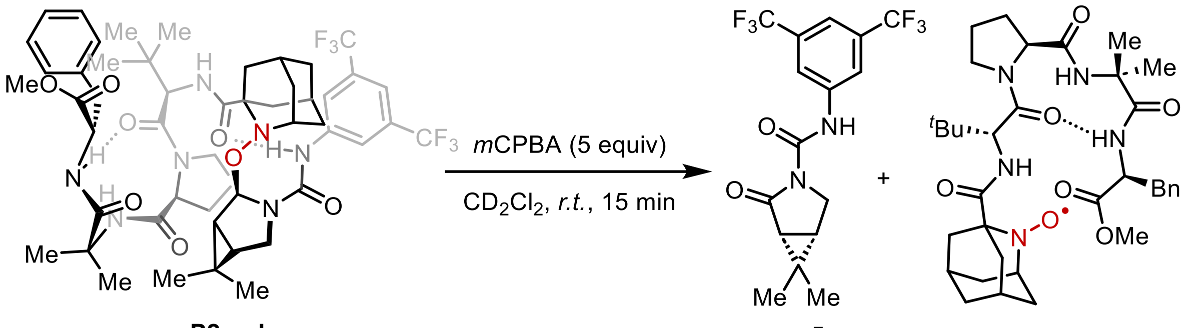

Reactivity: An NMR tube was charged with **P2·sub** (6.7 mg, 6.6 µmol, 1 equiv), 1,1,2,2-tetrachloroethane (10.0 µL, 15.9 mg, 0.0947 mmol) as an internal standard, and 0.75 mL CD_2Cl_2. The sample was locked and shimmed and a time = 0 min ^{1}H NMR spectrum was collected. *m*CPBA (7.4 mg, 0.037 mmol, 5 equiv, 77% purity) was added as a solid and the tube was vigorously shaken for 10 seconds and then loaded into the spectrometer. ^{1}H NMRs were collected with time increments indicating the reaction time after addition of the *m*CPBA. The concentrations of the product and reactant was quantified by integration between 1.136 – 1.125 ppm (Me group on the gem-dimethyl cyclopropyl moiety) and 5.581 – 5.473 ppm (N,O-ketal C–H of all **P2·sub** diastereomers). After completion of the reaction the sample was subjected to chiral HPLC analysis indicating that the **5** was formed with 92% *ee*.

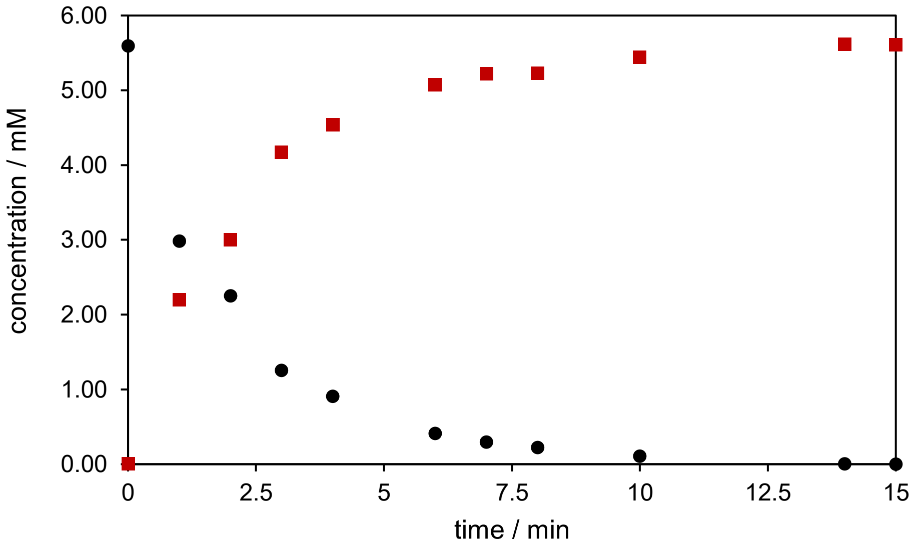

Figure S4. Concentration profile showing the consumption of P2·sub (black dots) and the formation of lactam product 5 (red squares) in >99% yield after 15 minutes.

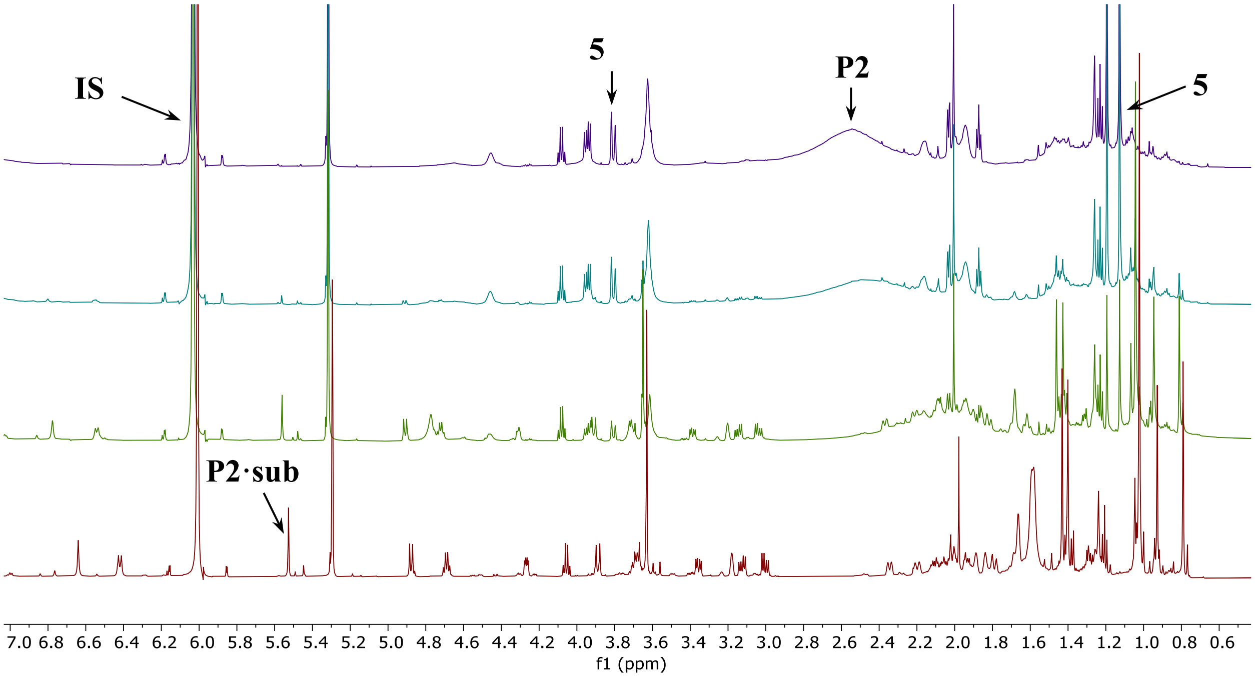

Figure S5. *In situ* NMRs at 0 min (bottom), 1 min, 6 min, and 15 min (top) after the addition of *m*CPBA. The broad peak at between 3.0 – 2.0 ppm and 1.7 – 0.7 ppm are consistent with the formation of the paramagnetic aminoxyl radical **P2** upon oxidation of **P2·sub**.

After 20 minutes 100 µL of the reaction mixture from the NMR tube were then added to 0.75 mL of 0.1 M tetrabutylammonium hexafluorophosphate in MeCN and cyclic voltammetry data was collected, verifying the presence of aminoxyl radical **P2** (*OCP* = 321 mV; $E_{1/2}$ = 429 mV). The potentials were internally calibrated through addition of ferrocene to account for the potential shift induced by the MeCN/CD_2Cl_2 solvent, compared to pure MeCN used for measuring CVs of **P2** and **P2**$^{+}$**NO**$_3^{-}$.

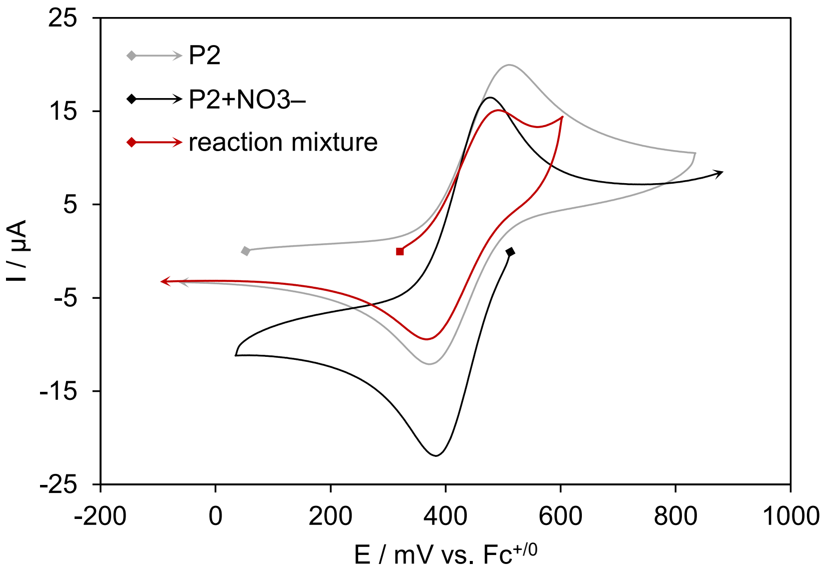

Figure S5. Cyclic voltammograms of the reaction mixture after exposure to *m*CPBA.

$NOSbF_6$ promoted oxidation: **P2** (13.1 mg, 0.02 mmol, 1 equiv), **A** (36.6 mg, 0.1 mmol, 5 equiv) and potassium carbonate (27.6 mg, 0.2 mmol, 20 equiv) were dissolved in 1 mL of DCM. In a separate vial $NOSbF_6$ (86 mg, 0.32 mmol) was dissolved in 0.5 mL of anhydrous MeCN. The oxidant solution was slowly titrated into the vigorously stirred orange **P2** solution using a 250 µL glass syringe until the solution was fully discolored (this took approximately 50 µL of the oxidant solution). The reaction was loaded onto a silica gel and eluted with a gradient from 0% hexanes to 50% EtOAc in hexanes. The product containing fractions as indicated by UV absorption upon TLC analysis were concentrated to provide **P2·sub** as a colorless solid (15.2 mg, 0.015 mmol, 75% yield) and an 82:6:12 *d.r.* The isolated product was oxidized using *m*CPBA (following the procedure described above) to provide **5** with 76% *ee*.

Relationship between P2·sub's *d.r.* and 5's *e.r.* upon oxidation: The oxidation of **P2·sub** with 91:6:3 *d.r.* provided lactam **5** with 92% *ee* (96:4 *e.r.*); **P2·sub** with 82:6:12 *d.r.* provided lactam **5** with 76% *ee* (88:12 *e.r.*). Taken together these results indicate that the first two **P2·sub** diastereomers correspond to the major enantiomer, while the third diastereomer corresponds to the minor enantiomer.

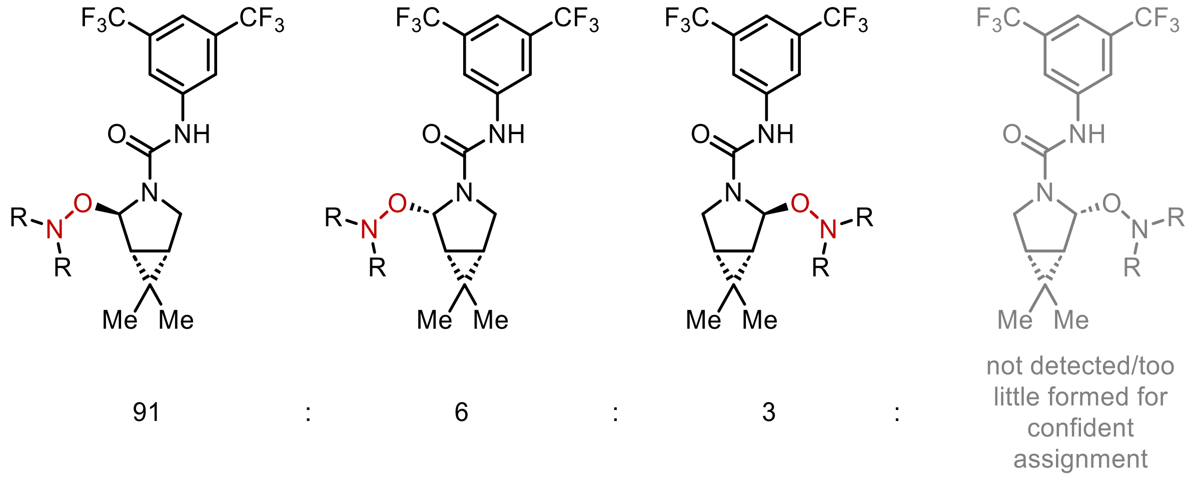

d.r. 91 : 6 : 3 :

7.3. ROESY analysis of P2·sub

Determination of experimental upper bounds for H···H distances in the solution conformation of P2·sub: 2D ROESY experiments were conducted for the determination of intramolecular upper bound distances, based on integration of ROESY crosspeaks following well-established procedures in the literature.[7] The NMR sample of **P2·sub** was prepared in CD_2Cl_2, as DCM provided similar enantioselectivity to the optimal reaction conditions in DCE under otherwise identical conditions (see section "3.4. Reaction Optimization at 0.1 mmol Scale"). The methylene units within the proline and AzcH residue were used as a distance reference (d = 1.78 Å). The intermolecular upper bound distances were tabulated for every observable cross peak and are provided in Table S4 (a fully assigned structure for **P2·sub** is provided in Figure S3). A total of 102 intramolecular H···H distances bounds were experimentally determined.

Evaluation of potential conformers: To identify the solution conformation of **P2·sub** the intramolecular experimental upper bound distances between all protons were compared to computationally generated conformers at various levels of theory. The mean and maximum deviation between the experimentally determined distance and the computed distance in each conformer were used for the evaluation, penalizing conformers with computed distances *larger* than those experimentally determined (Note: ROESY only provides upper bound distances, but not lower bound distances). The pairwise distance deviation is provided for the best conformer in Table S5. Detailed evaluation data for all conformers are available free of charge under https://github.com/JonasRein/Desymmetrization_of_Meso-Pyrrolidines in an excel file (ROESY-Analysis/Conformer Analysis - computed structure vs experimental.xlsx). Cartesian coordinates as .xyz files are available in the GitHub repository for all evaluated conformers. In addition, custom Python code (ROESY-Analysis/H-H-Distances-in-Conformer.py) that was developed to extract all intramolecular H···H distances within a molecule from .xyz files is available in the repository.

Conformer generation: Initial conformers were rapidly generated using a CREST[8] based conformational search followed by geometry optimization at the AimNet2[9] level of theory as implemented in Rowan[10]. Notably, the conformer that provided the best fit to the experimental data features a β-turn and an H-bond between the urea N–H and the AzcH carbonyl. In addition, an initial structure for further refinement was also generated by manual modification of dihedrals and angles based on the rOe map, yielding a structure featuring a β-turn and orienting the AzcH moiety, akin to that in the computationally obtained conformer. This manual analysis was performed prior to the development of the quantitative scoring and conformer search and was thus unbiased by those conformers.

The agreement between the manual and computationally obtained conformer suggested that the structure could serve as a good starting point for further granular geometry modification using density functional theory (DFT).

Table S2. Evaluation of the initial conformers prior to DFT refinement.

	Mean deviation from the upper distance bounds / Å	Maximum deviation from the upper distance bounds / Å
CREST-Rowan-conformer 1	-1.1	-10.67
CREST-Rowan-conformer 2	-0.89	-9.75

CREST-Rowan-conformer 3	-1.12	-11.6
CREST-Rowan-conformer 4	-0.65	-8.22
CREST-Rowan-conformer 5	-0.47	-6.04
CREST-Rowan-conformer 6	-0.51	-7.47
CREST-Rowan-conformer 7	-0.86	-9.35
CREST-Rowan-conformer 8	-0.95	-6.8
CREST-Rowan-conformer 9	-0.44	-6.16
CREST-Rowan-conformer 10	-1.06	-7.88
**CREST-Rowan-conformer 11**	**-0.18**	**-4.81**
CREST-Rowan-conformer 12	-0.91	-8.38
CREST-Rowan-conformer 13	-1	-8.34
CREST-Rowan-conformer 14	-0.79	-9.45

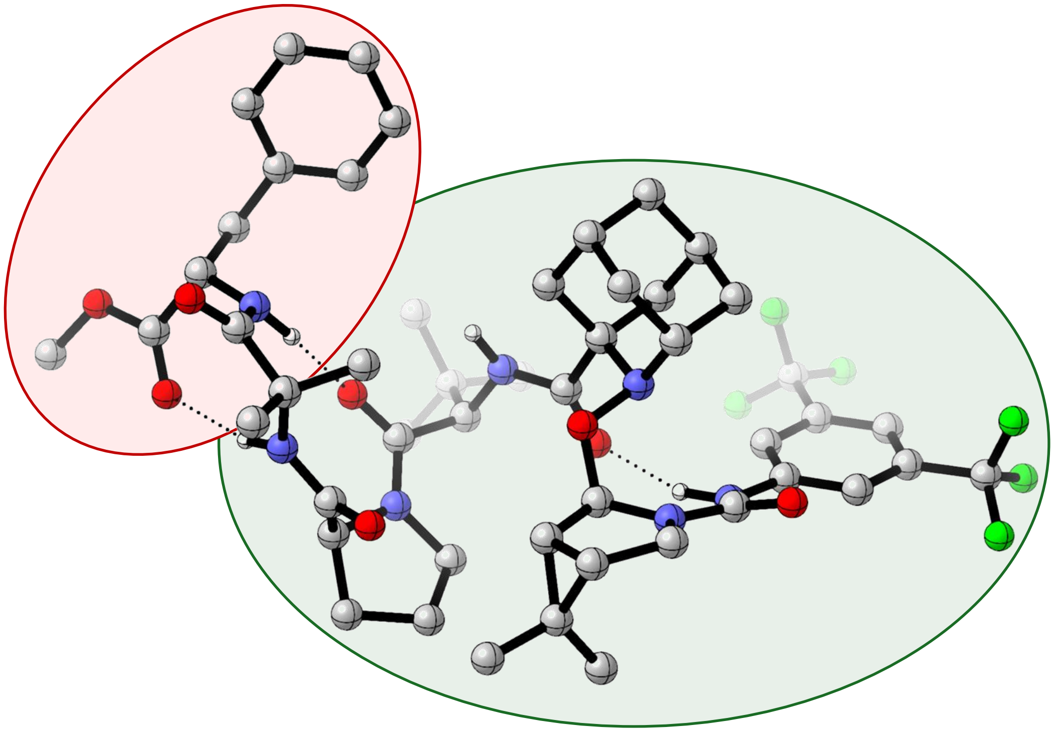

Figure S6. CREST-Rowan-conformer 11 showed excellent agreement with the experimental upper distance bounds within the Substrate- AzcH-^{d}Tle-Pro-Aib unit of **P2·sub** (green region). However, the Phe-OMe ester was engaged in an H-bond with the Aib N–H, which results in a large deviation from the experimental structure (red region). The experimental data suggests a conformation, in which the benzylic protons of the Phe unit are oriented towards the AzcH core. This and all other 3D structures were visualized using CYLview.[11]

CREST-Rowan-conformer 11 was then systematically refined by modifying dihedrals and angles focusing on the orientation of the Phe unit. The resulting conformers were preoptimized at the AimNet2 level of theory using Rowan and then subjected to geometry optimization and frequency calculation at the M062X/6-31G(d,p)[12] level of theory using Gaussian16.[13] All

conformers were verified as true minima with no imaginary frequencies. The resulting summary metrics upon conformer evaluation are provided in Table S3.

Table S3. Evaluation of the initial conformers prior to DFT refinement.

	Mean deviation from the upper distance bounds / Å	Maximum deviation from the upper distance bounds / Å
CREST-Rowan-conformer 11	-0.18	-4.81
DFT-refinement -conformer 1	-0.08	-1.11
DFT-refinement -conformer 2	-0.1	-2.74
DFT-refinement -conformer 3	-0.27	-6.51
DFT-refinement -conformer 4	-0.08	-1.11
DFT-refinement -conformer 5	-0.14	-4.08
DFT-refinement -conformer 6	-0.13	-1.47
DFT-refinement -conformer 7	-0.12	-1.42
DFT-refinement -conformer 8	-0.13	-1.84
**DFT-refinement -conformer 9**	**-0.09**	**-0.81**
DFT-refinement -conformer 10	-0.1	-1.8
DFT-refinement -conformer 11	-0.09	-0.85
DFT-refinement -conformer 12	-0.08	-1.8
DFT-refinement -conformer 13	-0.08	-1.86

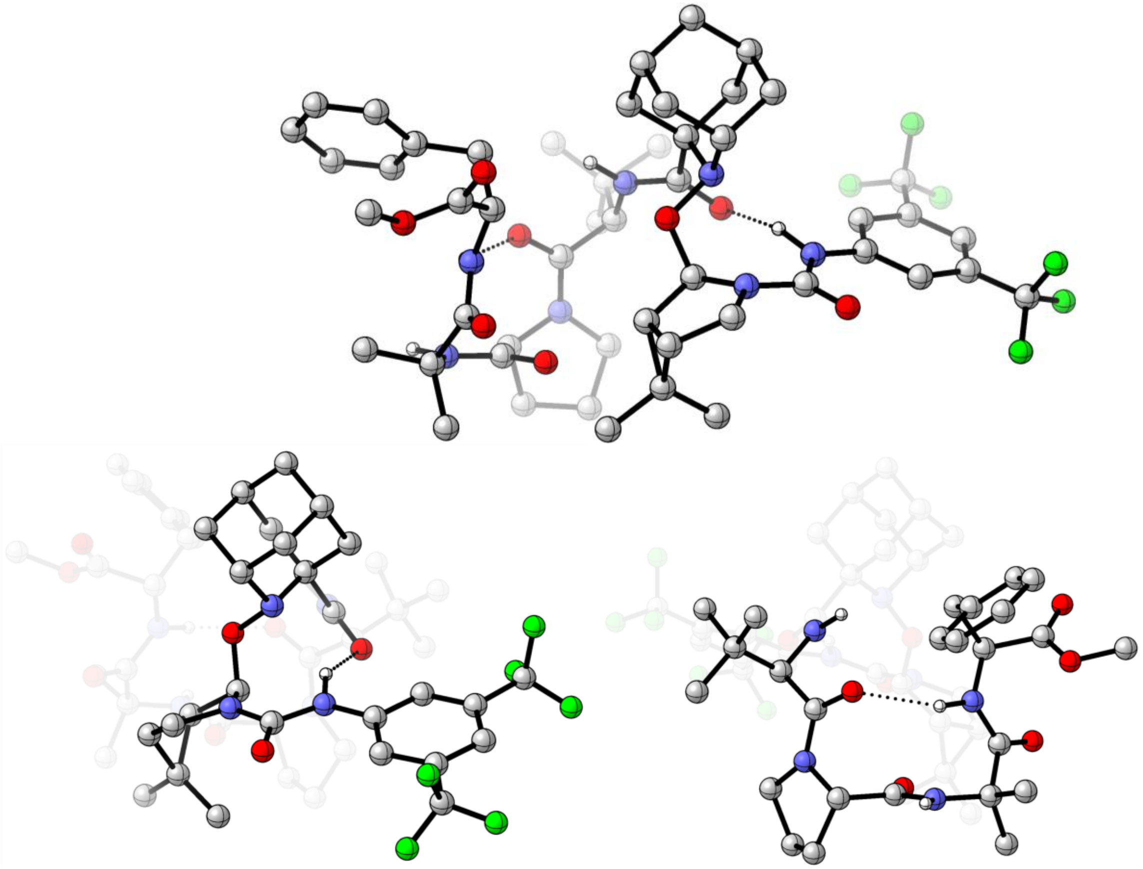

Figure S7. DFT-refinement-conformer 9 showed excellent agreement with the experimental upper distance bounds across all 102 intramolecular H···H distances evaluated (see Table S4).Fi

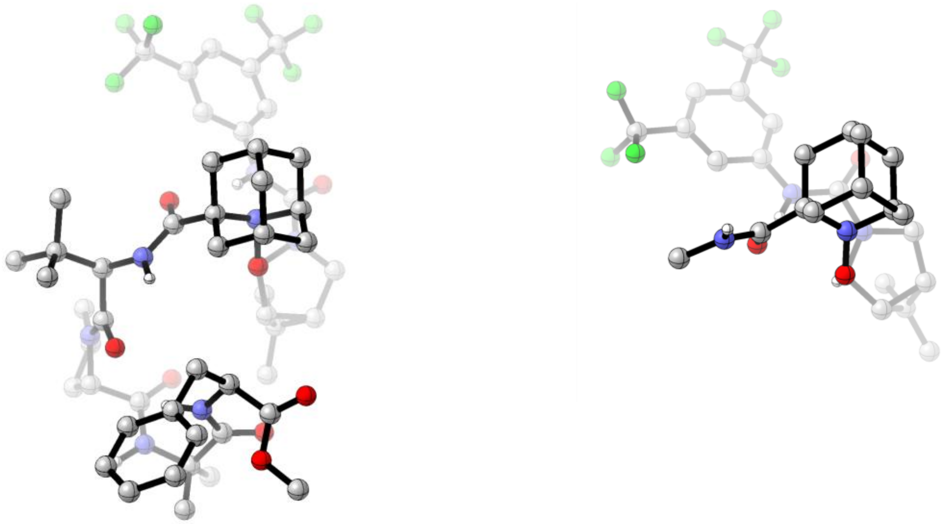

Figure S8. DFT-refinement-conformer 9 of **P2·sub** (left) and **TS-1** (right) view along the C–O bond.

	10.84	8.32 – 8.31 (m)	7.41	7.30 – 7.26 (m)	7.25 – 7.22 (m)	7.18 – 7.16 (m)	7.15	6.66	6.42 (d, J = 9.5 Hz)	5.55	4.90 (d, J = 9.5 Hz)	4.74 – 4.69 (m)	4.29 (dd, J = 7.9, 3.8 Hz)	3.91 (d, J = 11.6 Hz)	3.75 – 3.68 (m)	3.65	3.38 (dd, J = 11.6, 4.9 Hz)	3.21	3.15 (dd, J = 13.7, 6.6 Hz)	3.03 (dd, J = 13.7, 6.5 Hz)	2.37 (d, J = 12.2 Hz)	2.22 (d, J = 13.7 Hz)	2.17 – 2.11 (m)	2.13 – 2.06 (m)	2.07 – 1.99 (m)	2.04 (d, J = 12.4 Hz)	1.98 – 1.93 (m)	1.91	1.86	1.82 (d, J = 12.5 Hz)	1.71 (d, J = 12.1 Hz)	1.69 (s)	1.61 (d, J = 12.4 Hz)	1.61 (d, J = 11.8 Hz)	1.46	1.43	1.40 (d, J = 7.0 Hz)	1.32 (dd, J = 7.1, 4.9 Hz)	1.27 (d, J = 13.8 Hz)	1.05	0.95	0.82
0.82	5.3									3.1				2.9										[illegible]													4.1	3.7			2.9	
0.95										4.4		4.8			4.6								3.7	3.7											3.8		2.7	2.7				2.9
1.05		4.0				4.9			3.0	5.2	2.7		4.3		3.2				5.0	5.6																						
1.27 (d, J = 13.8 Hz)																		2.5				1.8						2.6				2.4										
1.32 (dd, J = 7.1, 4.9 Hz)										4.1				2.9			2.4																								2.7	3.7
1.40 (d, J = 7.0 Hz)										2.8				4.4	4.2		3.5				3.8	4.1																			2.7	4.1
1.43							3.4	2.8				4.7																														
1.46							3.4	3.0				4.7																													3.8	
1.61 (d, J = 12.4 Hz)									2.2												1.9							2.3	2.5	2.3												
1.61 (d, J = 11.8 Hz)									2.2												1.9							2.3	2.5	2.3												
1.69 (s)	3.4	3.8																2.3								2.6			2.4		2.3								2.4			
1.71 (d, J = 12.1 Hz)																												2.5				2.3										
1.82 (d, J = 12.5 Hz)									3.4																	1.9							2.3	2.3								
1.86																										2.5						2.4	2.5	2.5								
1.91																					2.5	2.5									2.5		2.3	2.3					2.6			
1.98 – 1.93 (m)																							2.0																			
2.04 (d, J = 12.4 Hz)	3.2	2.7																											2.5	1.9		2.6										
2.07 – 1.99 (m)													2.8																													
2.13 – 2.06 (m)																																									3.7	4.5
2.17 – 2.11 (m)								3.9					3.0														2.0														3.7	
2.22 (d, J = 13.7 Hz)																		2.4			2.6							2.5									4.1		1.8			
2.37 (d, J = 12.2 Hz)									2.5											3.3		2.6						2.5					1.9	1.9			3.8					
3.03 (dd, J = 13.7, 6.5 Hz)						2.8	3.3		3.6			2.6									3.3																			5.6		
3.15 (dd, J = 13.7, 6.6 Hz)						2.7	3.4		3.6			2.6																												5.0		
3.21	3.2									3.9							2.8					2.4										2.3							2.5			
3.38 (dd, J = 11.6, 4.9 Hz)										4.2				1.8				2.8																			3.5	2.4				
3.65						5.2																																				
3.75 – 3.68 (m)										3.3	2.3		3.8																								4.2			3.2	4.6	
3.91 (d, J = 11.6 Hz)										4.0							1.8																				4.4	2.9				2.9
4.29 (dd, J = 7.9, 3.8 Hz)								2.2							3.8								3.0		2.8															4.3		
4.74 – 4.69 (m)						3.4	3.2												2.6	2.6															4.7	4.7					4.8	
4.90 (d, J = 9.5 Hz)									3.0						2.3																									2.7		
5.55	2.0	3.8												4.0	3.3		4.2	3.9																			2.8	4.1		5.2	4.4	3.1
6.42 (d, J = 9.5 Hz)											3.0								3.6	3.6	2.5									3.4			2.2	2.2						3.0		
6.66							2.9						2.2										3.9												3.0	2.8						
7.15								2.9				3.2							3.4	3.3															3.4	3.4						
7.18 – 7.16 (m)												3.4				5.2			2.7	2.8																				4.9		
7.25 – 7.22 (m)																																										
7.30 – 7.26 (m)																																										
7.41		4.1																																								
8.32 – 8.31 (m)	2.3		4.1							3.8																2.7						3.8								4.0		
10.84		2.3								2.0								3.2								3.2						3.4										5.3

Table S4. Experimental H···H distance upper bound distances based on integration of ROESY in Å.

	10.84	8.32 – 8.31 (m)	7.41	7.30 – 7.26 (m)	7.25 – 7.22 (m)	7.18 – 7.16 (m)	7.15	6.66	6.42 (d, J = 9.5 Hz)	5.55	4.90 (d, J = 9.5 Hz)	4.74 – 4.69 (m)	4.29 (dd, J = 7.9, 3.8 Hz)	3.91 (d, J = 11.6 Hz)	3.75 – 3.68 (m)	3.65	3.38 (dd, J = 11.6, 4.9 Hz)	3.21	3.15 (dd, J = 13.7, 6.6 Hz)	3.03 (dd, J = 13.7, 6.5 Hz)	2.37 (d, J = 12.2 Hz)
0.82	0.0									0.0				0.0							
0.95										0.0		0.0			-0.2						
1.05		0.0				0.0			0.0	-0.1	0.0		0.0		0.0				0.0	0.0	
1.27 (d, J = 13.8 Hz)																		-0.1			
1.32 (dd, J = 7.1, 4.9 Hz)										-0.2				0.0			-0.1				
1.40 (d, J = 7.0 Hz)										0.0				0.0	-0.7		-0.4				-0.2
1.43							0.0	0.0				-0.1									
1.46							-0.8	-0.2				-0.5									
1.61 (d, J = 12.4 Hz)									0.0												0.0
1.61 (d, J = 11.8 Hz)									0.0												0.0
1.69 (s)	0.0	-0.1																-0.2			
1.71 (d, J = 12.1 Hz)																					
1.82 (d, J = 12.5 Hz)									-0.3												
1.86																					
1.91																					0.0
1.98 – 1.93 (m)																					
2.04 (d, J = 12.4 Hz)	0.0	0.0																			
2.07 – 1.99 (m)													0.0								
2.13 – 2.06 (m)																					
2.17 – 2.11 (m)								0.0					0.0								
2.22 (d, J = 13.7 Hz)																		-0.1			0.0
2.37 (d, J = 12.2 Hz)									0.0											0.0	
3.03 (dd, J = 13.7, 6.5 Hz)						0.0	-0.3		0.0			0.0									0.0
3.15 (dd, J = 13.7, 6.6 Hz)						0.0	0.0		0.0			0.0									
3.21	-0.2									0.0							0.0				
3.38 (dd, J = 11.6, 4.9 Hz)										0.0				0.0				0.0			
3.65						0.0															
3.75 – 3.68 (m)										-0.5	0.0		0.0								
3.91 (d, J = 11.6 Hz)										0.0							0.0				
4.29 (dd, J = 7.9, 3.8 Hz)								0.0							0.0						
4.74 – 4.69 (m)						-0.8	0.0												0.0	0.0	
4.90 (d, J = 9.5 Hz)									0.0						0.0						
5.55	0.0	0.0												0.0	-0.5		0.0	0.0			
6.42 (d, J = 9.5 Hz)											0.0								0.0	0.0	0.0
6.66							0.0						0.0								
7.15								0.0				0.0							0.0	-0.3	
7.18 – 7.16 (m)												-0.8				0.0			0.0	0.0	
7.25 – 7.22 (m)																					
7.30 – 7.26 (m)																					
7.41		-0.2																			
8.32 – 8.31 (m)	0.0		-0.2							0.0											
10.84		0.0								0.0								-0.2			

	2.22 (d, J = 13.7 Hz)	2.17 – 2.11 (m)	2.13 – 2.06 (m)	2.07 – 1.99 (m)	2.04 (d, J = 12.4 Hz)	1.98 – 1.93 (m)	1.91	1.86	1.82 (d, J = 12.5 Hz)	1.71 (d, J = 12.1 Hz)	1.69 (s)	1.61 (d, J = 12.4 Hz)	1.61 (d, J = 11.8 Hz)	1.46	1.43	1.40 (d, J = 7.0 Hz)	1.32 (dd, J = 7.1, 4.9 Hz)	1.27 (d, J = 13.8 Hz)	1.05	0.95	0.82
0.82			0.0													0.0	-0.2			0.0	
0.95		-0.8	0.0											0.0		0.0	0.0				0.0
1.05																					
1.27 (d, J = 13.8 Hz)	0.0						0.0				-0.1										
1.32 (dd, J = 7.1, 4.9 Hz)																				0.0	-0.2
1.40 (d, J = 7.0 Hz)	0.0																			0.0	0.0
1.43																					
1.46																				0.0	
1.61 (d, J = 12.4 Hz)							-0.2	0.0	-0.3												
1.61 (d, J = 11.8 Hz)							-0.2	0.0	-0.3												
1.69 (s)					0.0			-0.1		-0.3								-0.1			
1.71 (d, J = 12.1 Hz)							0.0				-0.3										
1.82 (d, J = 12.5 Hz)					0.0							-0.3	-0.3								
1.86					0.0						-0.1	0.0	0.0								
1.91	0.0									0.0		-0.2	-0.2					0.0			
1.98 – 1.93 (m)		-0.7																			
2.04 (d, J = 12.4 Hz)								0.0	0.0		0.0										
2.07 – 1.99 (m)																					
2.13 – 2.06 (m)																				0.0	0.0
2.17 – 2.11 (m)						-0.7														-0.8	
2.22 (d, J = 13.7 Hz)							0.0									0.0		0.0			
2.37 (d, J = 12.2 Hz)	0.0						0.0					0.0	0.0			-0.2					
3.03 (dd, J = 13.7, 6.5 Hz)																			0.0		
3.15 (dd, J = 13.7, 6.6 Hz)																			0.0		
3.21	-0.1										-0.2							-0.1			
3.38 (dd, J = 11.6, 4.9 Hz)																-0.4	-0.1				
3.65																					
3.75 – 3.68 (m)																-0.7			0.0	-0.2	
3.91 (d, J = 11.6 Hz)																0.0	0.0				0.0
4.29 (dd, J = 7.9, 3.8 Hz)		0.0		0.0															0.0		
4.74 – 4.69 (m)														-0.5	-0.1					0.0	
4.90 (d, J = 9.5 Hz)																			0.0		
5.55																0.0	-0.2		-0.1	0.0	0.0
6.42 (d, J = 9.5 Hz)									-0.3			0.0	0.0						0.0		
6.66		0.0												-0.2	0.0						
7.15														-0.8	0.0						
7.18 – 7.16 (m)																			0.0		
7.25 – 7.22 (m)																					
7.30 – 7.26 (m)																					
7.41																					
8.32 – 8.31 (m)					0.0						-0.1								0.0		
10.84					0.0						0.0										0.0

Table S5. Deviation between experimental data and DFT-refinement-conformer 9: (Experimental upper bound – computed distance) / Å (Note: When the computed H···H distance is shorter than the experimental distance upper bounds there is 0 Å error).

7.4. Transition State Analysis for a Model System

To understand the accessible conformations for hydride abstraction, a detailed conformational search of hydride transfer transition states between the AzcH-NHMe oxoammonium ion **26**$^+$ and substrate **A** was performed. The calculations were conducted using Gaussian16.[14] The structures were optimized at the M062X/6-31G(d,p) level of theory with SMD (dichloroethane) solvation correction, and a frequency calculation revealed a single imaginary frequency corresponding to the hydride transfer for the transition states. All ground states had no imaginary frequencies consistent with true minima. Single point energies were then calculated at the M062X/6-311G(d,p) level of theory, with SMD (dichloroethane) solvent correction and additional diffuse functions added for oxygen and nitrogen.[15] The reported energies are reported relative to **TS-1** and calculated based on the single point energy with thermal free energy correction obtained from the frequency calculation.

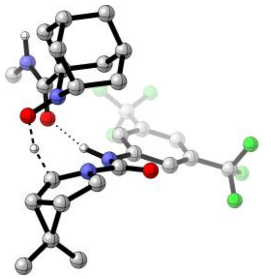

TS-1: 0.0 kcal/mol

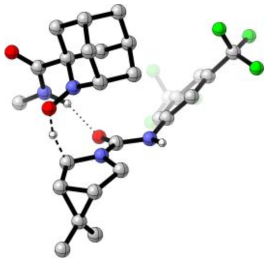

TS-2: 3.7 kcal/mol

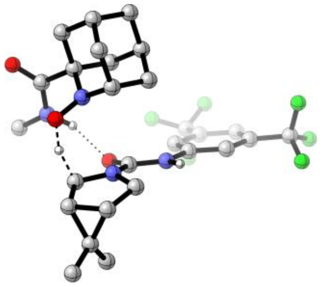

TS-3: 3.5 kcal/mol

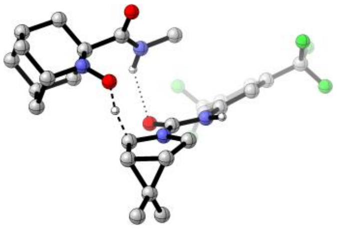

TS-4: 3.3 kcal/mol

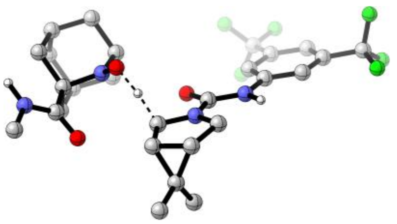

TS-5: 3.9 kcal/mol

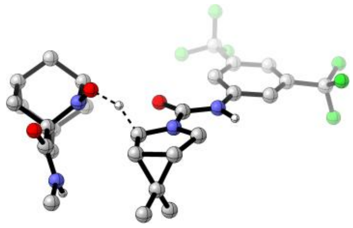

TS-6: 8.2 kcal/mol

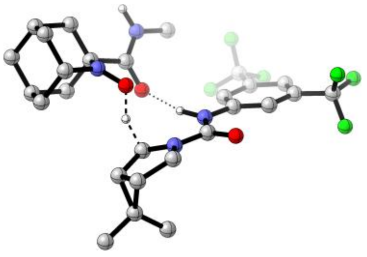

TS-7: 4.1 kcal/mol

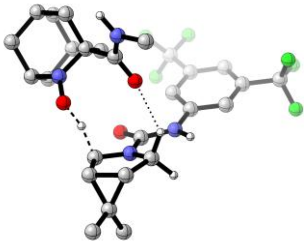

TS-8: 4.4 kcal/mol

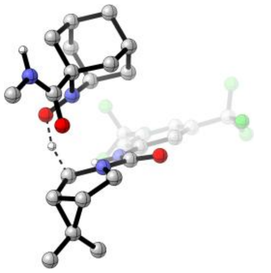

TS-9: 3.8 kcal/mol

7.4.1. Cartesian Coordinates

TS-1

1 1

C	3.629201	2.120275	-1.648029
C	3.960701	2.803090	-0.336544
C	2.374095	1.354388	-1.410409
N	1.913660	1.622758	-0.130299
C	2.859692	2.467773	0.645695
C	0.556051	1.630118	0.296781
O	0.248890	2.241165	1.303228
N	-0.288271	0.927979	-0.505404
C	-1.662534	0.754663	-0.275329
C	-2.433223	1.599916	0.526983
C	-3.794250	1.340424	0.666322
C	-4.413894	0.271289	0.032588
C	-3.629722	-0.552356	-0.769581
C	-2.271900	-0.322935	-0.929521
C	2.274026	-0.570035	2.606057
C	1.341959	-1.787270	2.692205
C	0.662342	-1.992046	1.329518
C	1.730298	-2.263372	0.251700
C	3.350109	-0.832846	1.550785
C	4.175682	-2.083543	1.909270
C	2.561281	-3.511028	0.640369
C	2.155184	-3.034605	3.067389
C	3.233189	-3.288976	2.002682
H	3.998560	0.025870	1.368204
H	2.310229	3.343529	0.998847
H	3.224532	1.930148	1.524026
H	0.138369	0.199593	-1.088725
H	-1.983218	2.442203	1.034006
H	-5.474639	0.085522	0.156986
H	-1.673451	-0.973066	-1.560952
H	1.706042	0.332968	2.358122
H	2.773957	-0.401647	3.564861
H	0.569059	-1.600813	3.442780
H	-0.010395	-2.855715	1.352037
H	0.076339	-1.110356	1.051005
C	1.078008	-2.332823	-1.132309
H	4.943396	-2.242333	1.144633
H	4.675315	-1.900103	2.865549
H	1.884647	-4.369650	0.709664
H	3.303256	-3.707325	-0.141572
H	2.626324	-2.889743	4.045759
H	1.493015	-3.904579	3.142237
H	3.809137	-4.183455	2.256785
O	3.312374	-0.863280	-0.804352

H	1.614839	1.252890	-2.189075
H	2.806100	0.150900	-1.370108
H	4.387150	1.669129	-2.278798
H	4.980033	2.828468	0.031204
N	2.690718	-1.123950	0.258791
O	0.739789	-1.301560	-1.730830
N	0.839169	-3.540994	-1.632726
H	1.134966	-4.368326	-1.134454
C	0.117089	-3.699154	-2.885847
H	0.604209	-3.123483	-3.675151
H	0.117757	-4.754896	-3.150612
H	-0.914474	-3.352367	-2.780853
C	3.592017	3.634730	-1.532900
C	2.294657	4.410579	-1.592662
H	2.406156	5.359636	-1.058125
H	2.046710	4.639667	-2.633569
H	1.442569	3.883313	-1.157906
C	4.745593	4.333289	-2.222726
H	4.523181	4.491719	-3.282583
H	4.922170	5.311289	-1.763185
H	5.667357	3.749644	-2.147826
C	-4.271805	-1.682027	-1.523520
C	-4.597625	2.221346	1.581749
F	-4.506118	1.819347	2.860671
F	-4.177162	3.494118	1.546438
F	-5.899596	2.218132	1.264489
F	-3.410732	-2.689259	-1.744125
F	-5.321641	-2.189063	-0.863399
F	-4.720513	-1.286939	-2.725674

TS-2

1 1

C	4.284830	-1.809404	-0.622716
C	3.828858	-1.738464	-2.068914
C	3.093863	-1.493634	0.199590
N	1.993038	-1.377504	-0.623959
C	2.347263	-1.419618	-2.066298
C	0.687640	-1.525979	-0.122218
O	0.484788	-1.698602	1.071693
N	-0.296145	-1.441648	-1.058412
C	-1.643443	-1.169417	-0.726102
C	-2.281567	-1.781869	0.356407
C	-3.599882	-1.445532	0.631659
C	-4.301754	-0.522628	-0.141341
C	-3.651459	0.062845	-1.217082
C	-2.330131	-0.253612	-1.519372
C	0.695448	1.918605	-1.481600
C	-0.155822	2.646291	-0.430408
C	0.092487	2.009477	0.947971
C	1.578950	2.154815	1.325113
C	2.177284	2.050438	-1.120552
C	2.587911	3.535571	-1.086599
C	1.980829	3.646177	1.327104
C	0.234766	4.131103	-0.404203
C	1.723773	4.265623	-0.049451
H	2.831427	1.468060	-1.773853
H	1.758189	-2.206567	-2.550050
H	2.123197	-0.460906	-2.544168
H	-0.041252	-1.226809	-2.015502
H	-1.757319	-2.506160	0.964686
H	-5.331204	-0.272540	0.090804
H	-1.829417	0.217270	-2.362469
H	0.423762	0.861252	-1.528995
H	0.541314	2.356436	-2.473008
H	-1.215008	2.537059	-0.684857
H	-0.501792	2.509546	1.719304
H	-0.199231	0.951579	0.934173
C	1.955543	1.520185	2.681946
H	3.650344	3.614432	-0.833722
H	2.441064	3.950328	-2.089102
H	1.390883	4.142293	2.103613
H	3.034748	3.719739	1.609671
H	0.038935	4.582787	-1.383243
H	-0.372124	4.664745	0.335803
H	2.007950	5.321893	-0.022279
O	3.473149	0.956694	0.531717
H	2.908606	-1.916888	1.189064
H	3.367229	-0.255330	0.573710
H	5.247761	-1.435174	-0.294340
H	4.446131	-1.261518	-2.821301
N	2.370558	1.512099	0.241235
O	2.526032	2.175589	3.542648
N	1.630264	0.227309	2.839174
H	1.087510	-0.271583	2.143602
C	1.951646	-0.466015	4.072623
H	1.670178	-1.513343	3.961700
H	3.022620	-0.398442	4.279853
H	1.409302	-0.036417	4.919963
C	-4.335954	1.096113	-2.066006
C	-4.302347	-2.062246	1.809537
F	-4.265237	0.789117	-3.370212
F	-5.627378	1.238551	-1.753025
F	-3.753898	2.301141	-1.926614

F	-5.529009	-2.491641	1.477960
F	-4.461404	-1.174920	2.804722
F	-3.631699	-3.105926	2.312002
C	4.252607	-3.058888	-1.495115
C	5.623653	-3.544663	-1.917608
H	6.058276	-4.187596	-1.146280
H	5.546188	-4.126563	-2.841691
H	6.306277	-2.709199	-2.095701
C	3.262344	-4.176713	-1.254521
H	3.087783	-4.720424	-2.188696
H	3.671314	-4.883851	-0.526860
H	2.294203	-3.837612	-0.879095

TS-3

1 1

C	-2.117319	0.860424	-0.033836
C	-2.579557	-0.115996	-0.916706
C	-2.995057	1.407055	0.912308
H	-1.920078	-0.551202	-1.655203
N	-0.785327	1.303416	0.008057
H	-2.639948	2.165777	1.602950
C	-4.787933	-0.007600	0.099918
C	0.175112	1.066990	-0.934204
H	-0.541310	1.847115	0.826244
H	-5.815198	-0.349278	0.155265
N	1.447206	1.551844	-0.595468
O	-0.007014	0.477583	-1.987654
C	1.752793	2.429125	0.563521
C	2.458493	1.534181	-1.539536
H	1.847624	1.830983	1.469720
H	0.945778	3.163668	0.671247
C	3.491499	2.527975	-1.147956
H	2.122403	1.418958	-2.571760
H	4.528519	2.309942	-1.375092
N	2.337916	-1.355585	-0.480255
C	1.177379	-2.136641	-0.961250
C	2.936609	-1.787455	0.811014
O	3.111482	-0.869123	-1.348047
C	0.095922	-2.151034	0.119957
C	1.711043	-3.563204	-1.210162
H	0.846963	-1.651283	-1.880708
C	1.823025	-1.794925	1.871369
C	3.450791	-3.223876	0.562923
C	3.998389	-0.748429	1.183676
C	0.678334	-2.714253	1.422280
H	-0.286361	-1.141518	0.289284
H	-0.735505	-2.765933	-0.240938
C	2.289379	-4.116089	0.099945
H	0.879180	-4.181540	-1.562075
H	2.473268	-3.532301	-1.995982
H	1.468938	-0.767714	2.011571
H	2.261657	-2.140652	2.813129
H	4.249867	-3.196023	-0.187129
H	3.862185	-3.609705	1.502235
N	5.187700	-1.203539	1.594782
O	3.717881	0.445862	1.135825
C	1.202698	-4.137962	1.185993
H	-0.095197	-2.721319	2.195546
H	2.670055	-5.127465	-0.070253
C	6.221401	-0.273804	2.014907
H	5.390811	-2.192208	1.590608
H	0.378404	-4.789125	0.874765
H	1.614972	-4.544805	2.116468
H	7.092024	-0.843747	2.335228
H	6.503828	0.385825	1.189935
H	5.865817	0.342473	2.844414
H	3.012238	0.390299	-1.409302
C	3.065794	3.987167	-1.025830
C	3.059360	3.094582	0.182772
H	3.786396	3.250517	0.970550
C	4.213498	4.972102	-1.108304
H	4.441724	5.213229	-2.151332
H	3.949909	5.902888	-0.595081
H	5.117661	4.570233	-0.642384
C	1.785121	4.501137	-1.645811
H	0.966931	3.776967	-1.633170
H	1.447609	5.394122	-1.109028
H	1.961375	4.785230	-2.687855
C	-3.906080	-0.534787	-0.829126
C	-4.308668	0.972897	0.967801
C	-4.339294	-1.623104	-1.769851
C	-5.259142	1.589543	1.955209
F	-3.602237	-2.734866	-1.595897
F	-4.183696	-1.261190	-3.052526
F	-5.621691	-1.963978	-1.601019
F	-6.059449	2.495022	1.370509
F	-6.059878	0.667274	2.508938
F	-4.618086	2.216179	2.951241

TS-4

1 1

C	-2.998258	3.049388	0.265941
C	-2.131244	3.935557	-0.614562
C	-2.184492	1.852274	0.572152

N	-0.900516	2.067091	0.117186
C	-0.796609	3.234619	-0.782431
C	0.129369	1.197929	0.477316
O	-0.072357	0.282381	1.265124
N	1.322805	1.461960	-0.125046
C	2.512906	0.739127	0.063352
C	2.753659	-0.069194	1.177216
C	3.967408	-0.745112	1.269243
C	4.952624	-0.634216	0.296960
C	4.696910	0.185981	-0.797064
C	3.494026	0.865570	-0.924262
C	-2.746041	-1.939823	0.815533
C	-4.133624	-1.990652	1.478533
C	-4.919960	-0.729289	1.092606
C	-5.096917	-0.699532	-0.441449
C	-2.913623	-1.904828	-0.722769
C	-3.692700	-3.148990	-1.190769
C	-5.850563	-1.943808	-0.921623
C	-4.894779	-3.239524	1.010215
C	-5.066893	-3.198419	-0.514892
C	-1.632537	-1.781480	-1.570475
H	0.046225	3.858476	-0.465267
H	-0.638215	2.903835	-1.813945
H	1.353842	2.158781	-0.860350
H	2.010547	-0.172956	1.956235
H	5.890614	-1.168771	0.387324
H	3.306605	1.489994	-1.793374
H	-2.189054	-1.062422	1.166546
H	-2.160218	-2.830697	1.067805
H	-4.001375	-2.016566	2.564151
H	-5.917093	-0.730332	1.544310
H	-4.404138	0.175597	1.433223
H	-5.561666	0.226730	-0.784783
H	-3.784869	-3.107305	-2.279069
H	-3.092898	-4.027235	-0.928963
H	-6.842713	-1.934339	-0.459938
H	-5.979183	-1.901056	-2.007408
H	-4.343362	-4.141476	1.299032
H	-5.875695	-3.279606	1.497047
H	-5.601134	-4.088948	-0.858426
O	-3.170218	0.387315	-1.238820
H	-2.270389	1.285542	1.502560
H	-2.731296	0.952202	-0.228432
H	-4.062229	2.920782	0.102773
H	-2.550460	4.432322	-1.481699
N	-3.746491	-0.721027	-1.024451
O	-1.716054	-1.793876	-2.791886
N	-0.465697	-1.715610	-0.910111
H	-0.442653	-1.576202	0.091562
C	0.785304	-1.648036	-1.643532
H	1.607914	-1.818665	-0.948733
H	0.915524	-0.672947	-2.126131
H	0.798305	-2.418282	-2.417342
C	4.171546	-1.652072	2.450497
C	5.744037	0.384967	-1.856622
F	3.448530	-2.778616	2.333596
F	5.450970	-2.017297	2.594925
F	3.786881	-1.066914	3.594923
F	6.379730	1.558568	-1.707081
F	5.206100	0.393739	-3.086001
F	6.675868	-0.575159	-1.830576
C	-2.502517	4.409844	0.756694
C	-3.591355	5.462084	0.809476
H	-4.137087	5.404225	1.756172
H	-3.149340	6.460712	0.732512
H	-4.305993	5.341092	-0.009100
C	-1.480811	4.548499	1.864093
H	-0.975425	5.515367	1.771850
H	-1.980832	4.524970	2.836929
H	-0.715829	3.769651	1.860203

TS-5

1 1

C	-2.539499	3.067237	-0.007219
C	-1.536688	4.118882	-0.453216
C	-1.802750	1.789059	0.064669
N	-0.464284	2.049276	-0.139436
C	-0.200944	3.421585	-0.617154
C	0.486961	1.047931	0.057478
O	0.142088	-0.074773	0.395640
N	1.772491	1.445946	-0.161741
C	2.924179	0.650354	-0.067249
C	2.923799	-0.681713	0.349087
C	4.133975	-1.373045	0.402626
C	5.339875	-0.782439	0.062542
C	5.318349	0.549763	-0.347903
C	4.135453	1.264215	-0.416875
C	-1.754275	-2.587784	0.786198
C	-3.137154	-3.061373	1.253648
C	-4.089645	-1.859068	1.255293
C	-4.219056	-1.287728	-0.170713
C	-1.862587	-2.038699	-0.637966
C	-2.397345	-3.116893	-1.603945

C	-4.748146	-2.380044	-1.127200
C	-3.670223	-4.143879	0.302677
C	-3.780393	-3.575687	-1.121170
H	-0.927470	-1.598414	-0.983468
H	0.580033	3.880527	0.000118
H	0.128411	3.398296	-1.660258
H	1.934936	2.401284	-0.455093
H	2.001768	-1.173299	0.626697
H	6.271173	-1.334875	0.114415
H	4.141054	2.301058	-0.738753
H	-1.360265	-1.807267	1.443452
H	-1.042277	-3.419551	0.776290
H	-3.064923	-3.457272	2.270900
H	-5.092149	-2.152505	1.585518
H	-3.727005	-1.066382	1.916777
C	-5.014000	0.020693	-0.106504
H	-2.452237	-2.702029	-2.616187
H	-1.690932	-3.953169	-1.612494
H	-5.737158	-2.699906	-0.779307
H	-4.845059	-1.963120	-2.136888
H	-2.995374	-5.007272	0.304908
H	-4.653058	-4.489732	0.643204
H	-4.166559	-4.341135	-1.800951
O	-2.699686	0.007802	-1.457196
H	-2.042593	0.994078	0.791523
H	-2.276547	1.025054	-0.884106
H	-3.571395	3.041821	-0.330418
H	-1.808615	4.867433	-1.188343
N	-2.859358	-0.956211	-0.654823
O	-4.554452	0.974942	0.516957
N	-6.213498	0.042252	-0.698166
H	-6.536945	-0.753779	-1.228031
C	-7.049904	1.226990	-0.623444
H	-6.585091	2.065385	-1.150033
H	-8.011441	1.002985	-1.082732
H	-7.203236	1.515071	0.419089
C	6.609862	1.194101	-0.765407
C	4.091154	-2.800598	0.871451
F	3.713355	-2.886556	2.157324
F	5.279464	-3.407091	0.764296
F	3.206479	-3.523409	0.165396
F	6.515510	2.529285	-0.821186
F	7.605747	0.895019	0.081290
F	6.998503	0.774688	-1.980636
C	-2.108176	4.200448	0.927530
C	-1.267272	3.950916	2.160072
H	-0.744951	4.872571	2.437600
H	-1.910515	3.666016	2.998016
H	-0.517251	3.167833	2.031047
C	-3.180146	5.250979	1.132149
H	-3.868858	4.947879	1.926647
H	-2.722167	6.202041	1.423142
H	-3.758305	5.415134	0.218606

TS-6

1 1

C	-2.631511	2.745398	-0.599228
C	-1.807105	1.531924	-0.430786
N	-0.484594	1.879238	-0.522282
C	-0.267612	3.274296	-0.954675
C	0.497400	0.900538	-0.326143
O	0.168258	-0.262795	-0.151833
N	1.772465	1.374923	-0.354307
C	2.950777	0.627641	-0.197170
C	2.980683	-0.768801	-0.112145
C	4.206549	-1.406381	0.038520
C	5.404949	-0.704659	0.107268
C	5.352707	0.680546	0.017231
C	4.145544	1.349320	-0.134726
C	-1.740547	-2.624296	0.847693
C	-3.057439	-2.837298	1.604471
C	-3.866751	-1.532807	1.542239
C	-4.206398	-1.184392	0.083096
C	-2.047824	-2.290694	-0.611832
C	-2.845793	-3.431602	-1.275867
C	-4.990725	-2.351555	-0.565844
C	-3.852042	-3.982986	0.961373
C	-4.157543	-3.638287	-0.504093
H	-1.152088	-2.024655	-1.174047
H	0.398046	3.779021	-0.245142
H	0.183511	3.288625	-1.950657
H	1.910886	2.374616	-0.442897
H	2.066980	-1.342741	-0.156288
H	6.350596	-1.221380	0.226969
H	4.126837	2.433430	-0.202171
H	-1.155818	-1.811984	1.289530
H	-1.131712	-3.533965	0.866904
H	-2.845686	-3.068431	2.652444
H	-4.806894	-1.630882	2.095293
H	-3.276573	-0.726408	1.994668
C	-4.998200	0.125794	-0.119961
H	-3.043330	-3.175378	-2.322185
H	-2.231572	-4.337637	-1.253924

H	-5.933152	-2.468230	-0.019341
H	-5.224699	-2.069848	-1.595905
H	-3.271930	-4.911155	1.015088
H	-4.787901	-4.143670	1.508949
H	-4.726827	-4.448932	-0.969247
O	-2.815941	-0.334204	-1.665464
H	-2.038283	0.722406	0.298317
H	-2.175987	0.677699	-1.360964
H	-3.606942	2.639833	-1.062205
N	-2.922652	-1.100768	-0.663581
O	-5.586819	0.342045	-1.170355
N	-5.013282	0.993258	0.910409
H	-4.595987	0.746356	1.795052
C	-5.860635	2.171075	0.852498
H	-6.911171	1.887324	0.737847
H	-5.736934	2.733570	1.777280
H	-5.583664	2.803848	0.005898
C	4.250214	-2.908894	0.075162
C	6.609918	1.494433	0.142317
F	4.563449	-3.422034	-1.125924
F	5.173629	-3.356578	0.938511
F	3.073527	-3.440782	0.433266
F	7.706307	0.768749	-0.107326
F	6.610894	2.535479	-0.704475
F	6.746372	2.004126	1.377641
C	-2.395649	3.914919	0.359491
C	-3.533975	4.912294	0.421153
H	-4.258434	4.631016	1.190540
H	-3.141652	5.903236	0.672711
H	-4.055926	4.984841	-0.537198
C	-1.695286	3.716623	1.685430
H	-1.287031	4.671942	2.030686
H	-2.413656	3.369822	2.435190
H	-0.875442	2.996107	1.647706
C	-1.662563	3.867147	-0.943872
H	-1.909557	4.588264	-1.714194

TS-7

1 1

C	-2.809933	2.805828	0.852338
C	-1.680011	1.877820	0.596828
N	-0.808929	2.492716	-0.279479
C	-1.389519	3.685670	-0.927073
C	0.533610	2.169165	-0.523195
O	1.242398	2.949357	-1.134977
N	0.926230	0.964151	-0.018348
C	2.240071	0.473938	0.011067
C	3.326125	1.072053	-0.632490
C	4.582985	0.476622	-0.531944
C	4.793398	-0.701170	0.169452
C	3.696607	-1.281830	0.801864
C	2.439926	-0.702416	0.745640
C	-3.561969	-1.737216	1.091138
C	-5.052849	-1.481287	0.836856
C	-5.192227	-0.224453	-0.031706
C	-4.487652	-0.464645	-1.382012
C	-2.832523	-1.984594	-0.256769
C	-3.476322	-3.174043	-0.990698
C	-5.106542	-1.661962	-2.108966
C	-5.678160	-2.684704	0.117100
C	-4.969511	-2.907950	-1.225902
C	-1.323915	-2.109341	0.024951
H	-0.657812	4.495948	-0.884072
H	-1.609728	3.467393	-1.976100
H	0.219229	0.296031	0.290445
H	3.195950	1.981398	-1.202923
H	5.776361	-1.153407	0.226311
H	1.597367	-1.157628	1.257066
H	-3.092301	-0.885904	1.594656
H	-3.406965	-2.624632	1.714579
H	-5.549958	-1.316823	1.797491
H	-6.242517	0.005359	-0.236916
H	-4.749087	0.642335	0.468905
H	-4.466651	0.433924	-2.000724
H	-2.974380	-3.333281	-1.951821
H	-3.340616	-4.065728	-0.369450
H	-6.159019	-1.432266	-2.300709
H	-4.608388	-1.809519	-3.072047
H	-5.587555	-3.581543	0.740237
H	-6.745425	-2.503863	-0.051924
H	-5.401078	-3.770473	-1.741174
O	-2.239041	0.161819	-1.112070
H	-1.238393	1.285818	1.402538
H	-2.208189	0.894850	-0.082115
H	-3.773648	2.390960	1.121759
N	-3.076660	-0.779441	-1.088197
O	-0.758339	-1.261633	0.726708
N	-0.701822	-3.183565	-0.456482
H	-1.203540	-3.844571	-1.032290
C	0.702165	-3.441116	-0.181362
H	0.896138	-3.342444	0.889337
H	1.340870	-2.739555	-0.724703
H	0.935012	-4.456833	-0.497409

C	5.730007	1.171167	-1.210455
C	3.853870	-2.600947	1.500883
F	3.634805	-3.630674	0.659763
F	5.085630	-2.765196	1.999430
F	2.984409	-2.743334	2.512814
F	6.004664	2.351271	-0.630661
F	5.455677	1.433808	-2.498414
F	6.854674	0.444724	-1.181558
C	-2.644807	3.956595	-0.123777
H	-3.502347	4.366123	-0.645158
C	-2.486664	4.217614	1.343239
C	-3.660764	4.900018	2.015506
H	-3.711955	4.627735	3.074321
H	-3.548087	5.987066	1.950068
H	-4.607932	4.626547	1.542133
C	-1.152173	4.580359	1.957147
H	-0.300372	4.079678	1.493432
H	-0.993331	5.659774	1.864869
H	-1.151604	4.333715	3.023321

TS-8

1 1

C	2.010475	-1.104582	-0.445627
C	2.227053	0.157161	-1.008142
C	3.057899	-1.739822	0.231112
C	3.470127	0.763733	-0.855916
H	1.440908	0.659183	-1.555261
N	0.778198	-1.771167	-0.481362
C	4.289418	-1.112608	0.351681
H	2.903544	-2.724615	0.663972
C	3.634661	2.133730	-1.450542
C	4.517707	0.151844	-0.182458
C	-0.348374	-1.337696	-1.113001
H	0.710851	-2.588426	0.112734
C	5.370394	-1.819843	1.119335
F	4.833611	2.665344	-1.185665
F	3.487917	2.117478	-2.784346
F	2.705964	2.984938	-0.976434
H	5.479607	0.638452	-0.077859
N	-1.505827	-2.043895	-0.779783
O	-0.401755	-0.403770	-1.899596
F	5.476365	-3.106172	0.750609
F	5.118879	-1.820411	2.439303
F	6.569209	-1.250852	0.945921
C	-1.626750	-3.078255	0.264434
C	-2.724261	-1.561031	-1.194131
H	-1.334443	-2.654261	1.229170
H	-0.979897	-3.926203	0.010254
C	-3.783026	-2.474948	-0.718200
H	-2.743379	-1.064644	-2.165451
H	-4.769769	-2.063749	-0.536572
N	-3.096454	1.517068	0.228301
C	-3.379141	2.485651	-0.838758
C	-1.986880	1.848234	1.150295
O	-3.605530	0.356687	0.188375
C	-2.097177	2.683265	-1.668035
C	-3.787295	3.795390	-0.145421
H	-4.194977	2.059380	-1.425180
C	-0.711548	2.092538	0.308500
C	-2.401703	3.161330	1.844344
C	-1.750739	0.621206	2.046142
C	-0.973257	3.174425	-0.743533
H	-1.810479	1.737753	-2.141307
H	-2.311928	3.412466	-2.455964
C	-2.649846	4.247866	0.781286
H	-3.977356	4.547339	-0.917792
H	-4.713308	3.638997	0.416700
H	-0.422887	1.153668	-0.165301
H	0.087944	2.390338	0.996891
H	-3.316094	3.006829	2.428422
H	-1.595742	3.460112	2.523009
N	-2.134807	0.702046	3.323671
O	-1.215792	-0.378640	1.569216
C	-1.378888	4.481293	-0.048309
H	-0.059615	3.324024	-1.327410
H	-2.942020	5.171218	1.289995
C	-1.936098	-0.417922	4.226165
H	-2.578698	1.539185	3.671587
H	-1.563357	5.264368	-0.792444
H	-0.564859	4.824395	0.600528
H	-2.500721	-1.290797	3.886649
H	-0.877621	-0.685501	4.272653
H	-2.279825	-0.129872	5.218347
H	-2.988127	-0.470460	-0.467880
C	-3.671824	-3.963467	-1.049461
C	-3.099441	-3.444353	0.233726
H	-3.567892	-3.719369	1.171856
C	-4.985349	-4.708150	-0.923208
H	-5.564566	-4.627110	-1.848172
H	-4.796879	-5.769673	-0.732482
H	-5.590816	-4.315291	-0.101672
C	-2.796360	-4.477151	-2.171298
H	-1.859018	-3.929233	-2.283888

H	-2.551195	-5.528387	-1.987001
H	-3.336739	-4.422668	-3.121239

TS-9

1 1

C	2.010373	0.887368	0.076261
C	2.623800	0.568055	-1.142379
C	2.755670	0.838167	1.257118
C	3.964792	0.205900	-1.143371
H	2.066947	0.609169	-2.066446
N	0.655738	1.242053	0.203135
C	4.095871	0.473273	1.218934
H	2.288682	1.090103	2.205701
C	4.637245	-0.161319	-2.437623
C	4.722051	0.150845	0.022970
C	-0.259593	1.342865	-0.804635
H	0.362289	1.437707	1.151163
C	4.849372	0.383504	2.516374
F	5.757616	0.552096	-2.629169
F	5.000901	-1.454506	-2.448265
F	3.848158	0.038084	-3.499848
H	5.769236	-0.130582	-0.002440
N	-1.586850	1.581234	-0.398638
O	-0.008257	1.223377	-1.990710
F	4.564132	-0.753378	3.173350
F	4.534369	1.392903	3.342058
F	6.174295	0.410015	2.331687
C	-2.558663	1.954850	-1.462940
C	-2.051621	1.869479	0.870507
H	-2.945237	1.056128	-1.941157
H	-2.025240	2.562723	-2.198094
C	-3.313017	2.646752	0.751086
H	-1.317529	2.156030	1.627069
H	-4.072185	2.516097	1.513107
N	-1.863799	-1.183615	0.709275
C	-0.482542	-1.721091	0.719683
C	-2.889157	-2.032779	0.041366
O	-2.223909	-0.480603	1.694367
C	-0.033853	-1.993626	-0.717419
C	-0.540203	-3.035336	1.524342
H	0.133176	-0.975719	1.225285
C	-2.413100	-2.282274	-1.400658
C	-2.920906	-3.361173	0.830821
C	-4.200162	-1.241375	0.031900
C	-1.029868	-2.950731	-1.386298
H	0.038524	-1.065292	-1.289194
H	0.965262	-2.439694	-0.676554
C	-1.523545	-3.994990	0.843050
H	0.468214	-3.460256	1.551534
H	-0.850767	-2.819559	2.552205
H	-2.377204	-1.323735	-1.930213
H	-3.154064	-2.921796	-1.891253
H	-3.279460	-3.168940	1.848611
H	-3.621680	-4.039544	0.331043
N	-5.324101	-1.904977	0.330955
O	-4.191210	-0.055830	-0.285184
C	-1.082276	-4.269873	-0.602547
H	-0.717377	-3.136707	-2.417634
H	-1.566856	-4.931083	1.407465
C	-6.607496	-1.227580	0.268853
H	-5.296461	-2.874245	0.611047
H	-0.093781	-4.742351	-0.607209
H	-1.784470	-4.963600	-1.078781
H	-6.634464	-0.391098	0.972238
H	-6.785939	-0.839048	-0.736945
H	-7.389359	-1.940639	0.524936
H	-2.386262	0.723526	1.359912
C	-3.255125	3.972741	-0.004880
C	-3.638287	2.710287	-0.721020
H	-4.660725	2.571540	-1.051438
C	-4.395966	4.912829	0.325580
H	-4.163769	5.506466	1.215488
H	-4.567403	5.603479	-0.506780
H	-5.323954	4.364365	0.509653
C	-1.950027	4.686898	-0.281317
H	-1.106729	4.015758	-0.460398
H	-2.059160	5.322194	-1.166508
H	-1.690002	5.333268	0.562851

26^+

1 1

N	-0.20488	1.093733	-0.49174
C	-1.68128	1.177238	-0.61201
C	0.362053	-0.13205	0.155586
O	0.491669	1.935091	-0.95182
C	-2.27829	1.040699	0.800158
C	-2.0872	-0.01701	-1.50654
H	-1.87459	2.143455	-1.07912
C	-0.29799	-0.24633	1.550563
C	-0.09482	-1.30064	-0.75268
C	1.878113	0.105686	0.321534
C	-1.82149	-0.28172	1.421796
H	-1.97661	1.894245	1.413468

H	-3.3644	1.075279	0.674975
C	-1.62517	-1.32141	-0.85264
H	-3.17638	0.027584	-1.59592
H	-1.6541	0.108077	-2.50316
H	0.040541	0.593655	2.162083
H	0.09229	-1.17052	1.988997
H	0.353376	-1.19271	-1.74622
H	0.286519	-2.21516	-0.28683
N	2.697795	-0.69521	-0.36695
O	2.253965	0.984581	1.084093
C	-2.24952	-1.46395	0.541992
H	-2.25503	-0.37605	2.421089
H	-1.91848	-2.16225	-1.48705
C	4.137755	-0.55964	-0.22099
H	2.333677	-1.39742	-0.99441
H	-3.34072	-1.49101	0.456779
H	-1.92597	-2.40451	1.000183
H	4.420423	-0.65695	0.82989
H	4.619895	-1.34469	-0.8008
H	4.471117	0.416665	-0.58201

4

0 1

C	-5.28797	-0.49535	-0.70232
C	-4.91357	0.968967	-0.69511
C	-4.01661	-1.31236	-0.61565
N	-2.95246	-0.31542	-0.46609
C	-3.4052	1.072219	-0.59755
C	-1.66752	-0.73723	-0.35637
O	-1.37099	-1.92926	-0.32334
N	-0.72794	0.282829	-0.28435
C	0.652864	0.144792	-0.15821
C	1.318269	-1.08272	-0.0757
C	2.706722	-1.09954	0.051505
C	3.463675	0.059413	0.10077
C	2.78541	1.275061	0.015959
C	1.408949	1.329335	-0.11173
H	-3.08629	1.66269	0.272675
H	-2.99477	1.538087	-1.50199
H	-1.06142	1.237047	-0.31908
H	0.756794	-2.00583	-0.11021
H	4.542248	0.025578	0.202903
H	0.90733	2.290178	-0.17346
H	-3.99971	-2.00252	0.235297
H	-3.86658	-1.90172	-1.52671
H	-6.07498	-0.86118	-1.35312
H	-5.42098	1.679213	-1.33902
C	3.368681	-2.44421	0.153563
C	3.592852	2.540671	0.031579
F	4.704998	-2.35491	0.189928
F	2.984916	-3.10173	1.260843
F	3.050009	-3.23299	-0.88581
F	4.293607	2.693825	-1.10498
F	4.487307	2.542384	1.032889
F	2.831318	3.633886	0.174273
C	-5.67437	0.37746	0.464526
C	-7.13975	0.754403	0.542357
H	-7.71026	-0.01591	1.07248
H	-7.2697	1.699623	1.080612
H	-7.57328	0.870341	-0.45567
C	-5.02322	0.204791	1.820613
H	-5.09716	1.134164	2.395965
H	-5.53793	-0.57769	2.388627
H	-3.96731	-0.06826	1.756687

26H

0 1

C	-2.18253	1.021216	-0.85117
C	-1.94237	-0.45494	-1.20115
C	-0.43577	-0.66336	-1.41681
C	0.307651	-0.28493	-0.12909
C	-1.41071	1.360542	0.431701
C	-1.90479	0.490062	1.591094
C	-0.16926	-1.18724	1.028045
C	-2.43033	-1.34392	-0.04562
C	-1.6736	-0.98668	1.243199
H	-1.51355	2.421812	0.676842
H	-1.84488	1.667385	-1.66741
H	-3.24972	1.213237	-0.68969
H	-2.48169	-0.71254	-2.11909
H	-0.20959	-1.70502	-1.66284
H	-0.0729	-0.04058	-2.24035
C	1.819653	-0.46784	-0.28911
H	-1.36752	0.753466	2.509023
H	-2.96981	0.68836	1.75455
H	0.059713	-2.22729	0.770085
H	0.384887	-0.93366	1.939247
H	-3.50789	-1.20332	0.105069
H	-2.26709	-2.40056	-0.29142
H	-2.01341	-1.62284	2.067428
O	0.47407	1.948189	-0.80576
H	1.244916	2.403373	-0.43775
N	0.040514	1.115601	0.273878
O	2.295007	-1.26715	-1.08861

N	2.58256	0.266405	0.548736
H	2.112774	0.858549	1.220498
C	4.020846	0.103597	0.58544
H	4.442291	0.844443	1.264513
H	4.301577	-0.89707	0.930017
H	4.444955	0.250761	-0.41119

27

1 1

C	4.997878	1.015216	-0.59965
C	5.280071	-0.48994	-0.76882
C	3.48763	1.153794	-0.52754
N	3.017576	-0.2577	-0.55574
C	3.990005	-1.1076	-0.74427
C	1.646885	-0.72793	-0.43409
O	1.433685	-1.91687	-0.45748
N	0.757672	0.276434	-0.31275
C	-0.64088	0.136644	-0.18182
C	-1.28096	-1.09939	-0.05929
C	-2.6644	-1.1242	0.077003
C	-3.42736	0.036797	0.094614
C	-2.76823	1.254242	-0.02996
C	-1.38879	1.316066	-0.16787
H	1.095453	1.232846	-0.33001
H	-0.71874	-2.02167	-0.06753
H	-4.50624	-0.00654	0.200991
H	-0.895	2.278075	-0.26772
H	3.15039	1.632047	0.397078
H	3.071254	1.674209	-1.39314
H	5.546227	1.725497	-1.20712
H	6.078688	-0.92475	-1.35488
C	-3.37467	-2.44689	0.16655
C	-3.55713	2.531918	0.042891
F	-3.95916	-2.77052	-0.99805
F	-2.54377	-3.4478	0.48297
F	-4.3453	-2.41926	1.0912
F	-4.77788	2.39151	-0.48951
F	-2.94461	3.53333	-0.60425
F	-3.72823	2.934014	1.312255
C	5.678615	0.316022	0.510087
C	7.175828	0.514746	0.607714
H	7.621161	0.710696	-0.37082
H	7.382781	1.368662	1.259929
H	7.65166	-0.36945	1.040266
C	5.009966	0.062407	1.8415
H	5.133236	0.952059	2.46688
H	3.941644	-0.15267	1.774705
H	5.494742	-0.77666	2.346956
H	3.760185	-2.16151	-0.87025

8. HPLC Traces

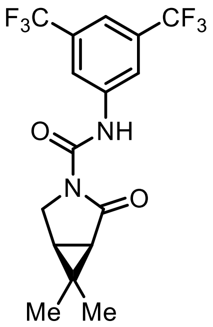

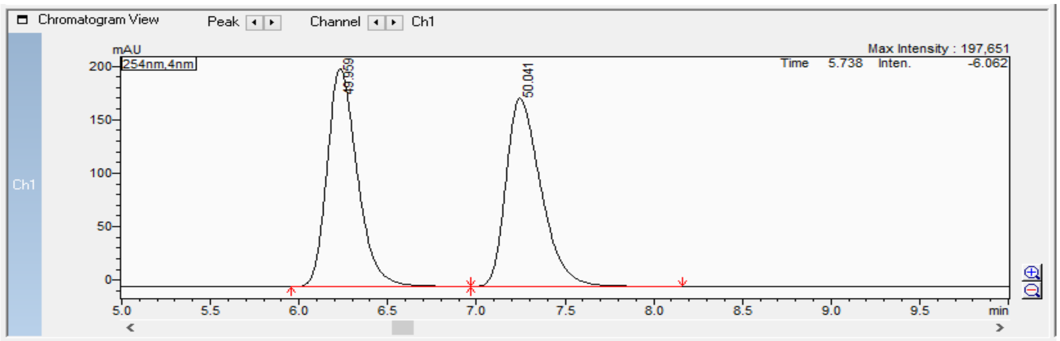

Results View - Peak Table

Peak Table | Compound | Group | Calibration Curve

Peak#	Ret. Time	Area%
1	6.233	49.959
2	7.246	50.041
Total		100.000

Extracted 190nm | Ch1 254nm

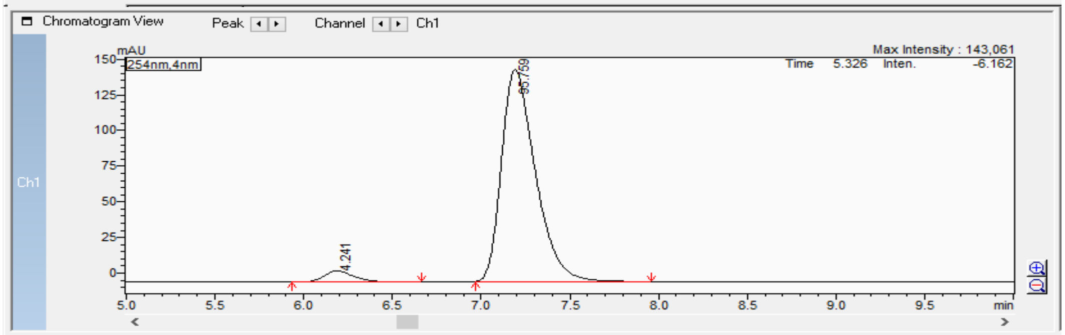

Results View - Peak Table

Peak Table | Compound | Group | Calibration Curve

Peak#	Ret. Time	Area%
1	6.190	4.241
2	7.192	95.759
Total		100.000

Extracted 190nm | Ch1 254nm

LC charts of racemic (top) and enantioenriched (bottom) compound **5**. ChiralCel® OD-H 250 x 4.6 mm ID Analytical column with a column temperature of 40 °C, flow rate 1.0 mL/min, 2% *i*PrOH in hexanes, isocratic.

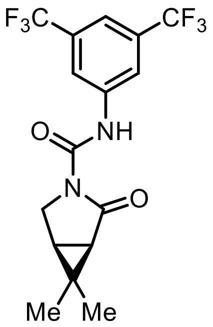

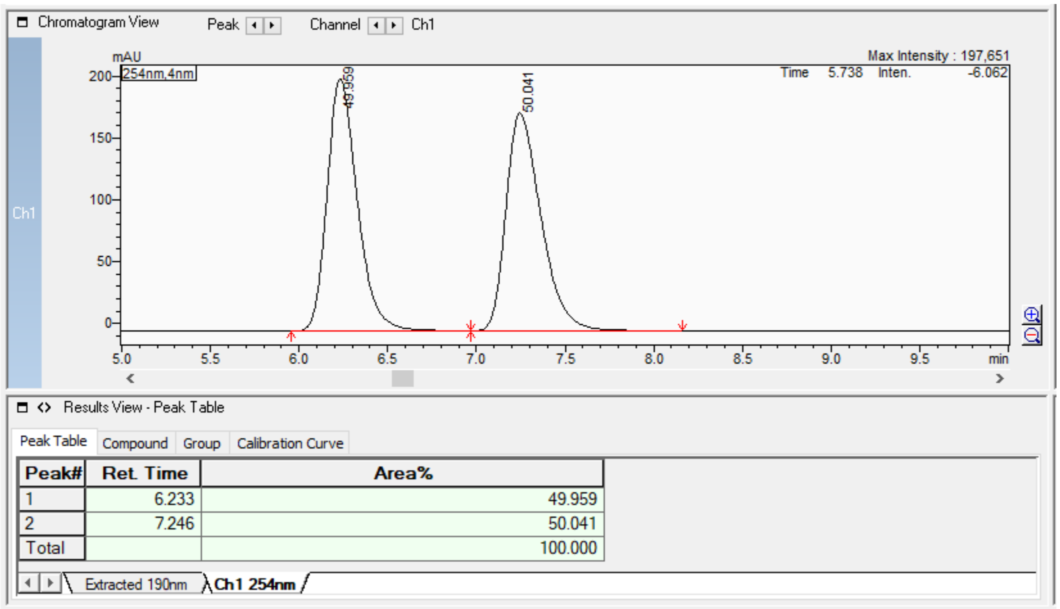

Peak#	Ret. Time	Area%
1	6.233	49.959
2	7.246	50.041
Total		100.000

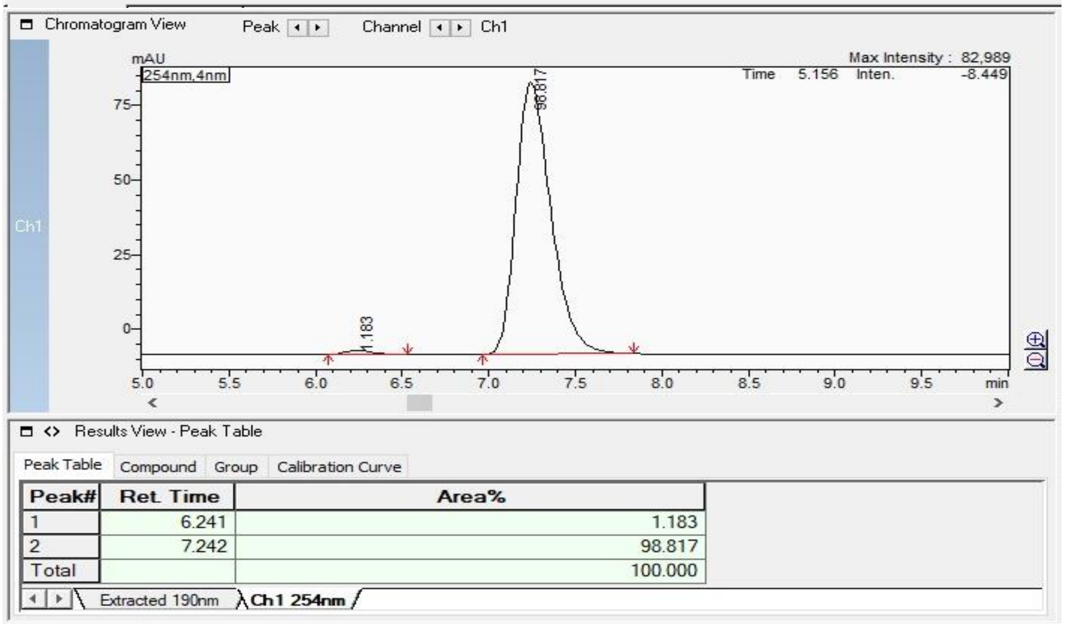

Peak#	Ret. Time	Area%
1	6.241	1.183
2	7.242	98.817
Total		100.000

LC charts of racemic (top) and enantioenriched by crystallization (bottom) compound **5**. ChiralCel® OD-H 250 x 4.6 mm ID Analytical column with a column temperature of 40 °C, flow rate 1.0 mL/min, 2% *i*PrOH in hexanes, isocratic.

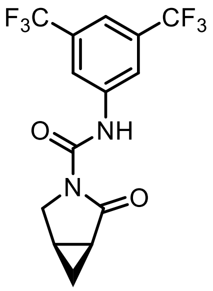

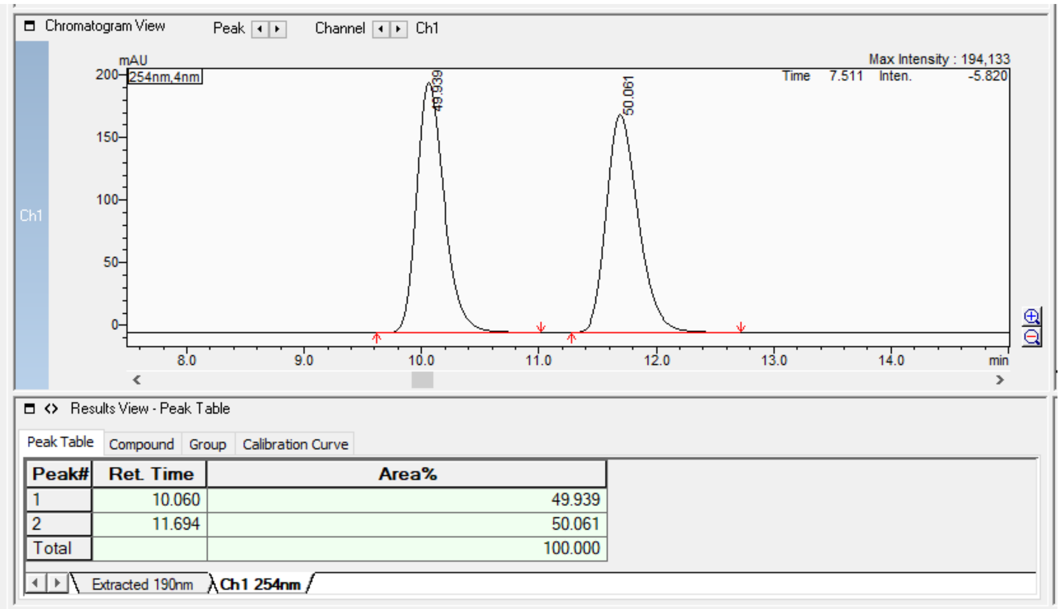

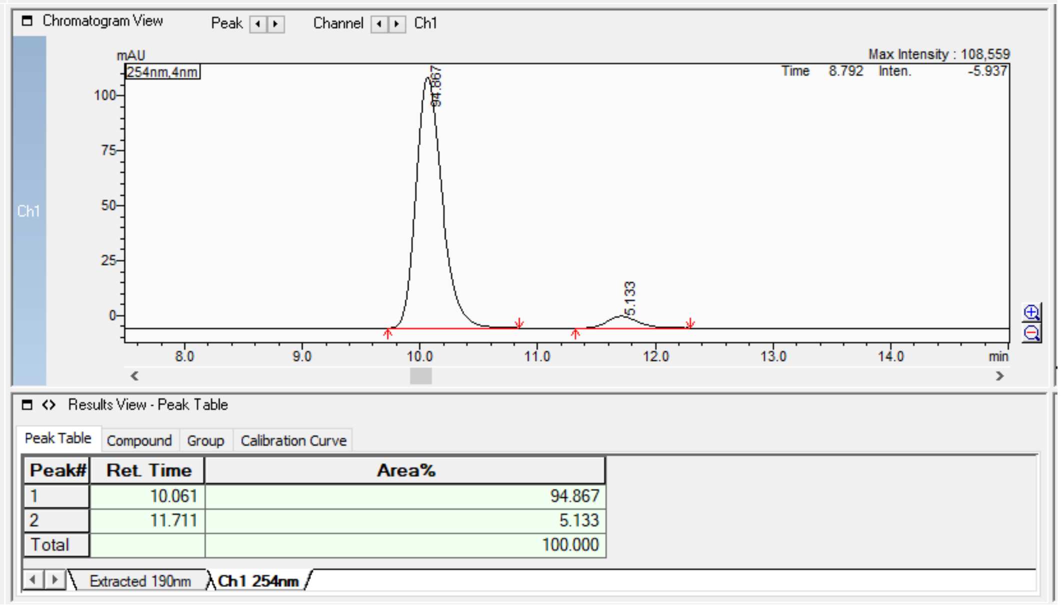

LC charts of racemic (top) and enantioenriched (bottom) compound **7**. ChiralCel® OD-H 250 x 4.6 mm ID Analytical column with a column temperature of 40 °C, flow rate 1.0 mL/min, 2% *i*PrOH in hexanes, isocratic.

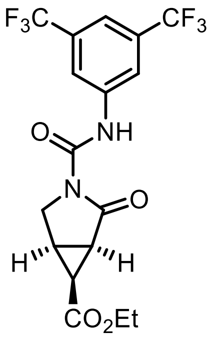

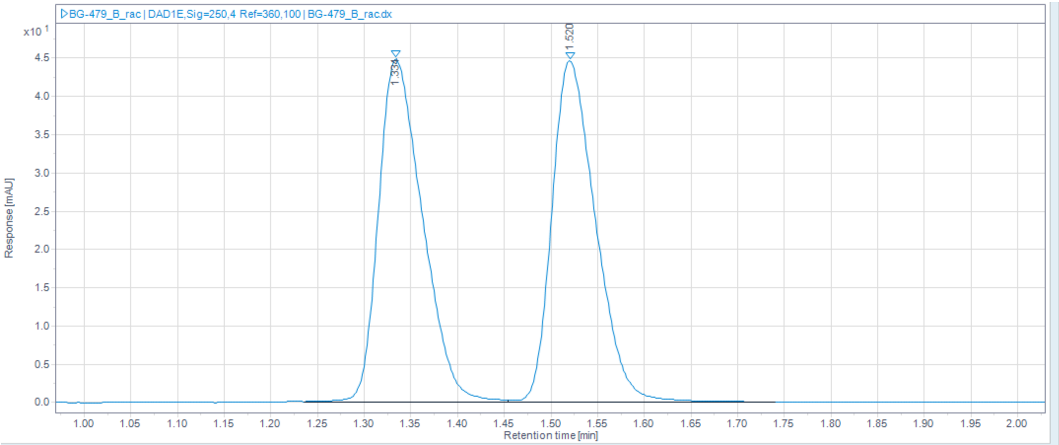

Injection Results

Peaks | Summary

#	Signal description	RT (min)	Area (mAU·s)	Area%	Height (mAU)	Height%	Amount	Concentration	Start time (min)	End time (min)
1	DAD1E,Sig=250,4 Ref=360,100	1.334	147.682	50.336	44.872	50.14			1.235	1.454
2	DAD1E,Sig=250,4 Ref=360,100	1.520	145.711	49.664	44.622	49.86			1.454	1.740

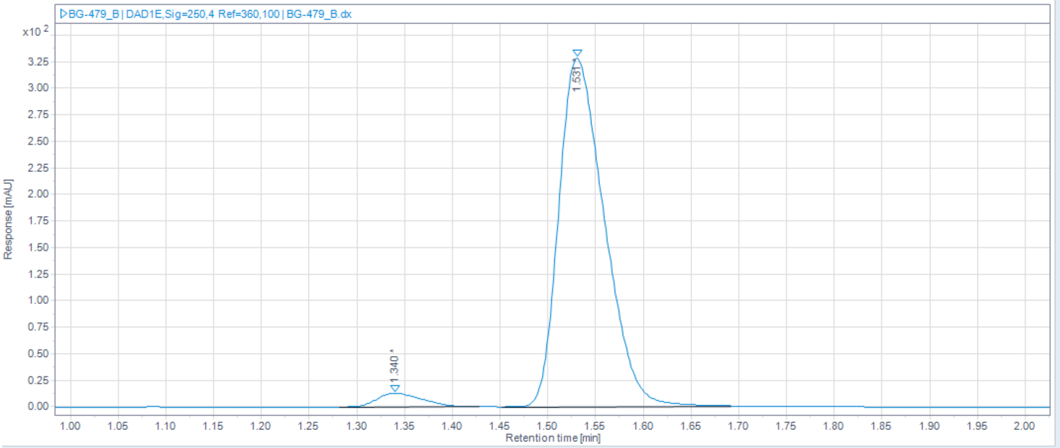

Injection Results

Peaks | Summary

#	Signal description	RT (min)	Area (mAU·s)	Area%	Height (mAU)	Height%	Amount	Concentration	Start time (min)	End time (min)
1	DAD1E,Sig=250,4 Ref=360,100	1.340	42.682	3.781	13.070	3.83			1.281	1.428
2	DAD1E,Sig=250,4 Ref=360,100	1.531	1086.172	96.219	328.304	96.17			1.453	1.691

SFC charts of racemic (top) and enantioenriched (bottom) compound **8**. Chiralpak® IB N-3 150 x 4.6 mm ID Analytical column with a column temperature of 40 °C, flow rate 2.75 mL/min, 3% MeOH in CO_2, isocratic.

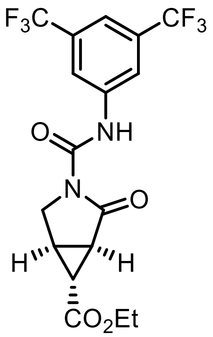

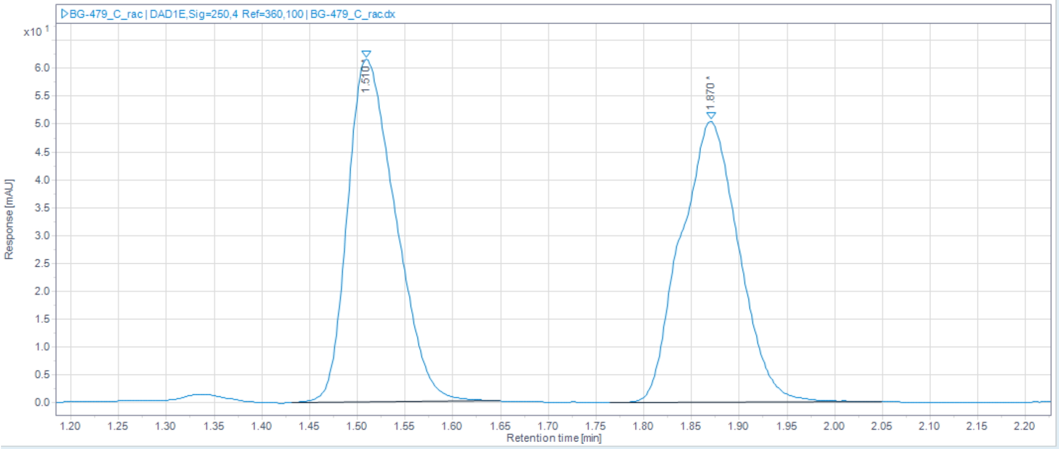

Injection Results

Peaks | Summary

#	Signal description	RT (min)	Area (mAU·s)	Area%	Height (mAU)	Height%	Amount	Concentration	Start time (min)	End time (min)
1	DAD1E,Sig=250,4 Ref=360,100	1.510	214.260	50.089	61.547	54.97			1.432	1.649
2	DAD1E,Sig=250,4 Ref=360,100	1.870	213.494	49.911	50.410	45.03			1.765	2.049

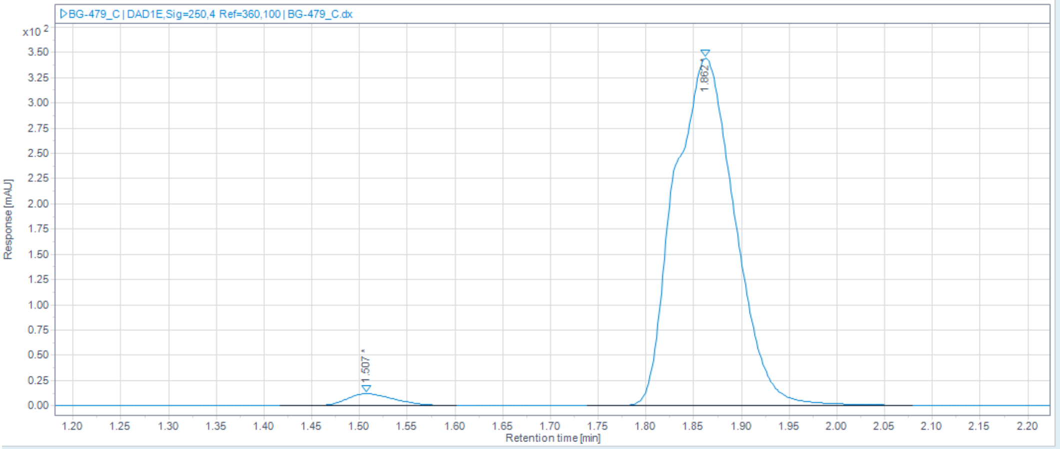

Injection Results

Peaks | Summary

#	Signal description	RT (min)	Area (mAU·s)	Area%	Height (mAU)	Height%	Amount	Concentration	Start time (min)	End time (min)
1	DAD1E,Sig=250,4 Ref=360,100	1.507	40.642	2.617	11.774	3.31			1.416	1.602
2	DAD1E,Sig=250,4 Ref=360,100	1.862	1512.419	97.383	344.335	96.69			1.739	2.079

SFC charts of racemic (top) and enantioenriched (bottom) compound **9**. Chiralpak® IB N-3 150 x 4.6 mm ID Analytical column with a column temperature of 40 °C, flow rate 2.75 mL/min, 3% *i*PrOH in CO_2, isocratic.

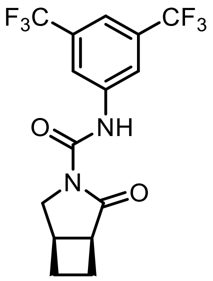

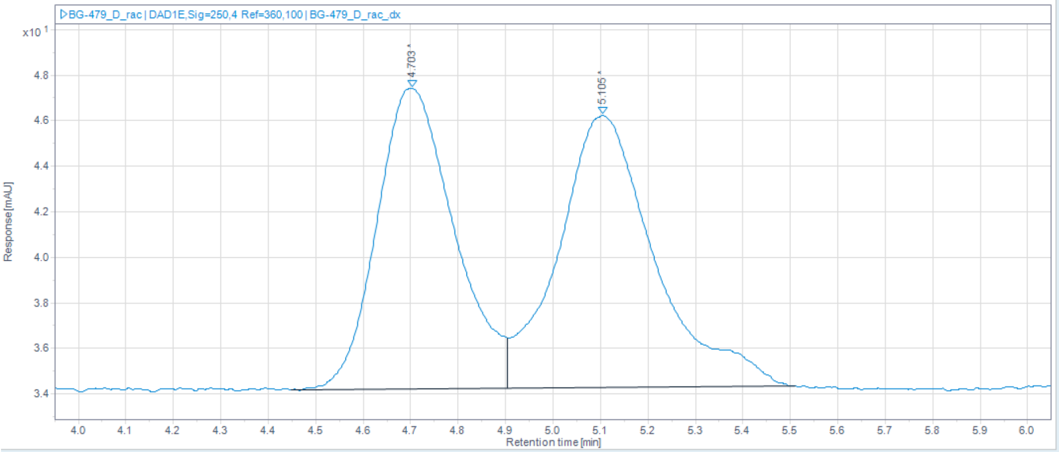

Injection Results

Peaks | Summary

#	Signal description	RT (min)	Area (mAU·s)	Area%	Height (mAU)	Height%	Amount	Concentration	Start time (min)	End time (min)
1	DAD1E,Sig=250,4 Ref=360,100	4.703	149.326	46.985	13.199	52.52			4.448	4.904
2	DAD1E,Sig=250,4 Ref=360,100	5.105	168.493	53.015	11.932	47.48			4.904	5.510

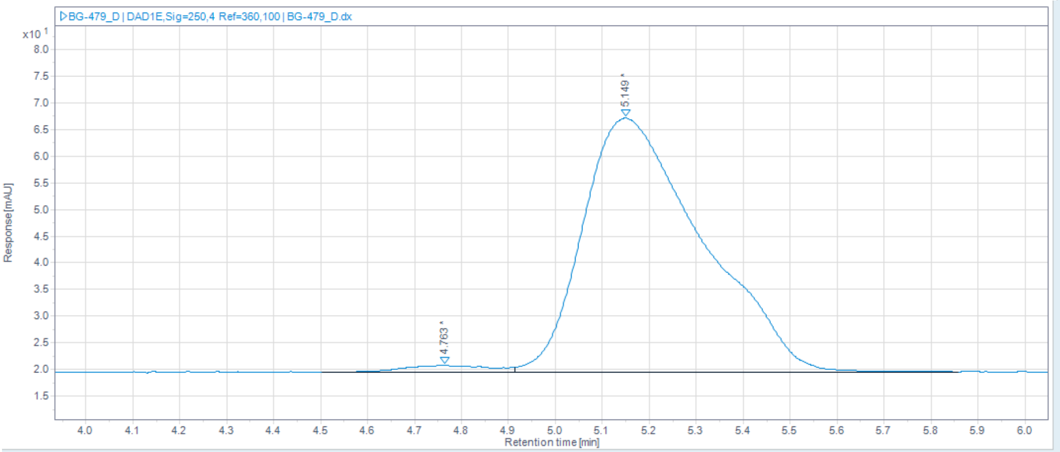

Injection Results

Peaks | Summary

#	Signal description	RT (min)	Area (mAU·s)	Area%	Height (mAU)	Height%	Amount	Concentration	Start time (min)	End time (min)
1	DAD1E,Sig=250,4 Ref=360,100	4.763	15.692	1.832	1.234	2.53			4.502	4.913
2	DAD1E,Sig=250,4 Ref=360,100	5.149	840.822	98.168	47.594	97.47			4.913	5.857

SFC charts of racemic (top) and enantioenriched (bottom) compound **10**. Chiralpak® IB N-3 150 x 4.6 mm ID Analytical column with a column temperature of 40 °C, flow rate 2.0 mL/min, CO_2, isocratic.

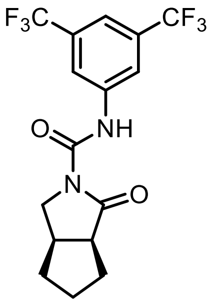

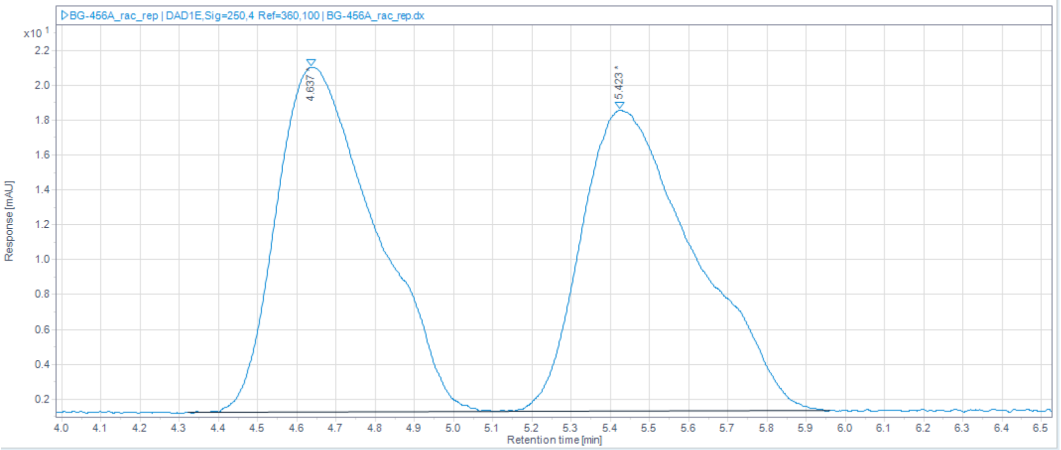

Injection Results

Peaks | Summary

#	Signal description	RT (min)	Area (mAU·s)	Area%	Height (mAU)	Height%	Amount	Concentration	Start time (min)	End time (min)
1	DAD1E,Sig=250,4 Ref=360,100	4.637	346.755	49.883	19.735	53.36			4.321	5.111
2	DAD1E,Sig=250,4 Ref=360,100	5.423	348.383	50.117	17.247	46.64			5.111	5.960

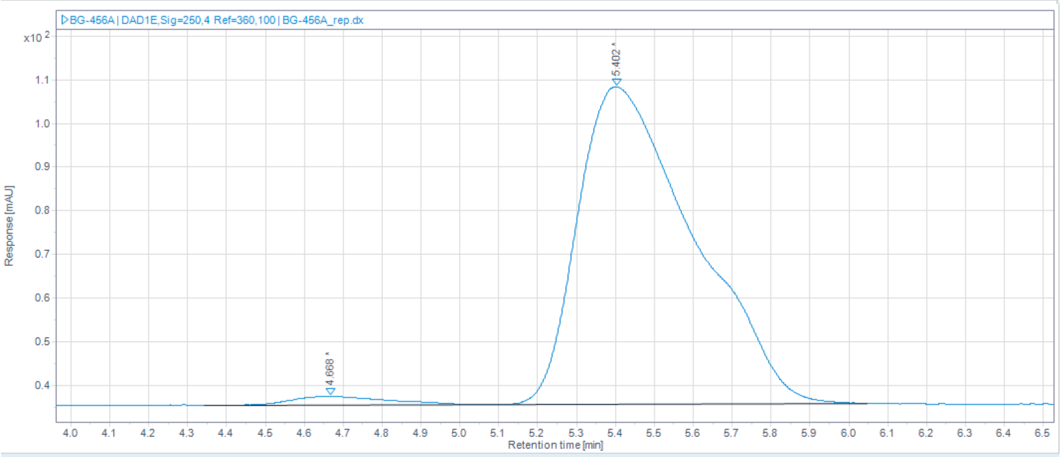

Injection Results

Peaks | Summary

#	Signal description	RT (min)	Area (mAU·s)	Area%	Height (mAU)	Height%	Amount	Concentration	Start time (min)	End time (min)
1	DAD1E,Sig=250,4 Ref=360,100	4.668	33.578	2.186	1.953	2.62			4.342	5.067
2	DAD1E,Sig=250,4 Ref=360,100	5.402	1502.733	97.814	72.700	97.38			5.067	6.048

SFC charts of racemic (top) and enantioenriched (bottom) compound **11**. Chiralpak® IB N-3 150 x 4.6 mm ID Analytical column with a column temperature of 40 °C, flow rate 2.0 mL/min, CO_2, isocratic.

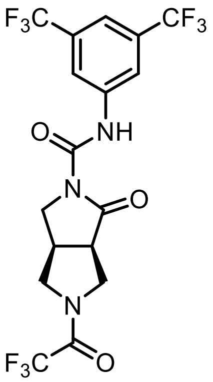

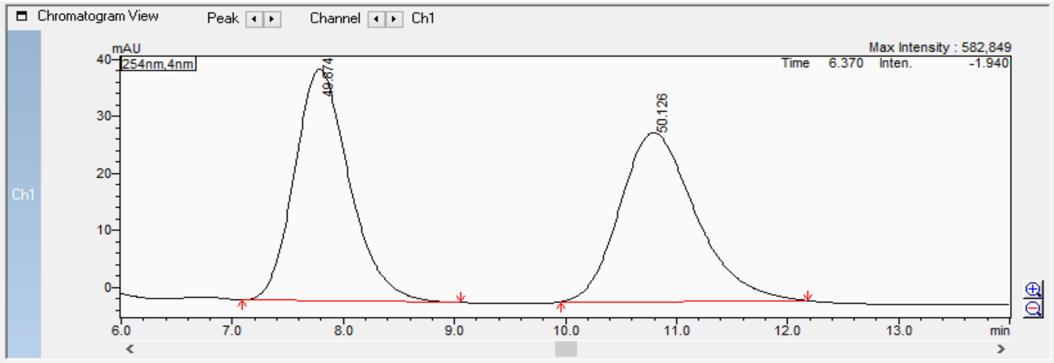

Results View - Peak Table

Peak Table | Compound | Group | Calibration Curve

Peak#	Ret. Time	Area%
1	7.784	49.874
2	10.794	50.126
Total		100.000

Extracted 190nm | Ch1 254nm

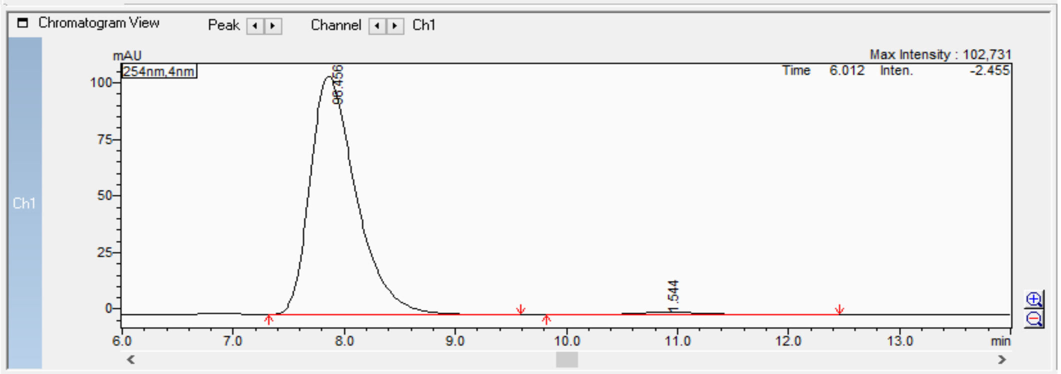

Results View - Peak Table

Peak Table | Compound | Group | Calibration Curve

Peak#	Ret. Time	Area%
1	7.859	98.456
2	10.883	1.544
Total		100.000

Extracted 190nm | Ch1 254nm

LC charts of racemic (top) and enantioenriched (bottom) compound **12**. ChiralCel® AD-H 150 x 4.6 mm ID Analytical column with a column temperature of 40 °C, flow rate 1.0 mL/min, 7.5% *i*PrOH in hexanes, isocratic.

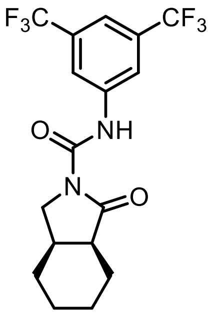

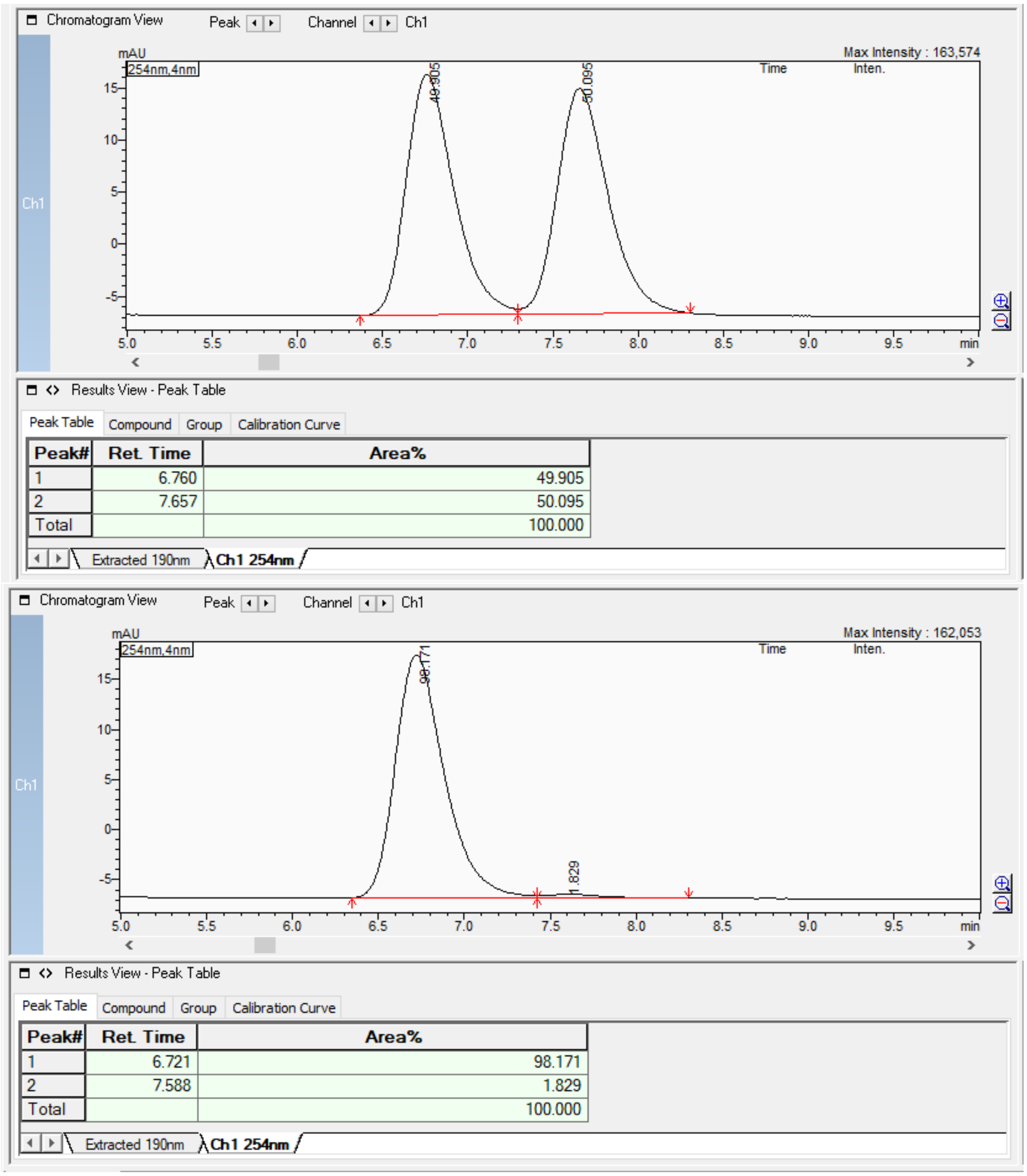

Peak#	Ret. Time	Area%
1	6.760	49.905
2	7.657	50.095
Total		100.000

Peak#	Ret. Time	Area%
1	6.721	98.171
2	7.588	1.829
Total		100.000

LC charts of racemic (top) and enantioenriched (bottom) compound **13**. ChiralCel® OD-H 250 x 4.6 mm ID Analytical column with a column temperature of 40 °C, flow rate 1.0 mL/min, hexanes, isocratic.

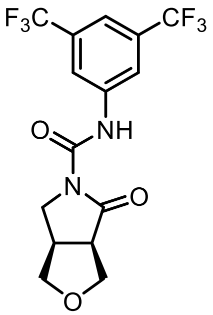

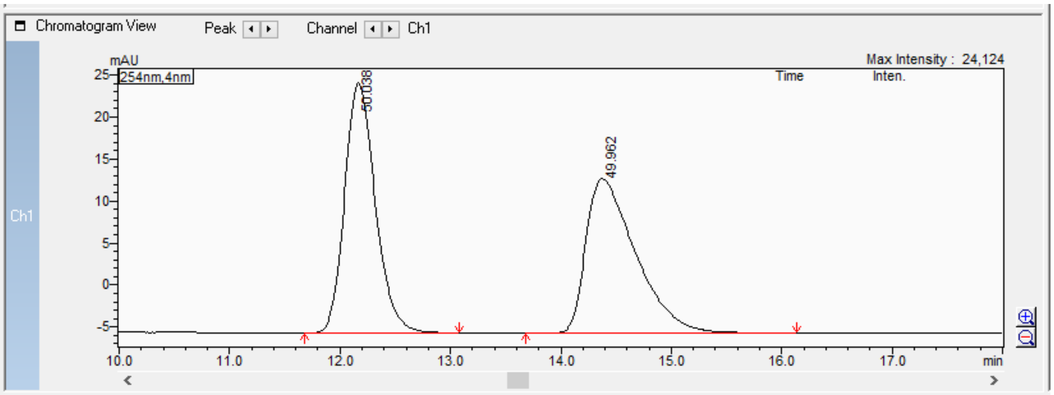

Results View - Peak Table

Peak Table | Compound | Group | Calibration Curve

Peak#	Ret. Time	Area%
1	12.168	50.038
2	14.375	49.962
Total		100.000

Extracted 190nm | Ch1 254nm

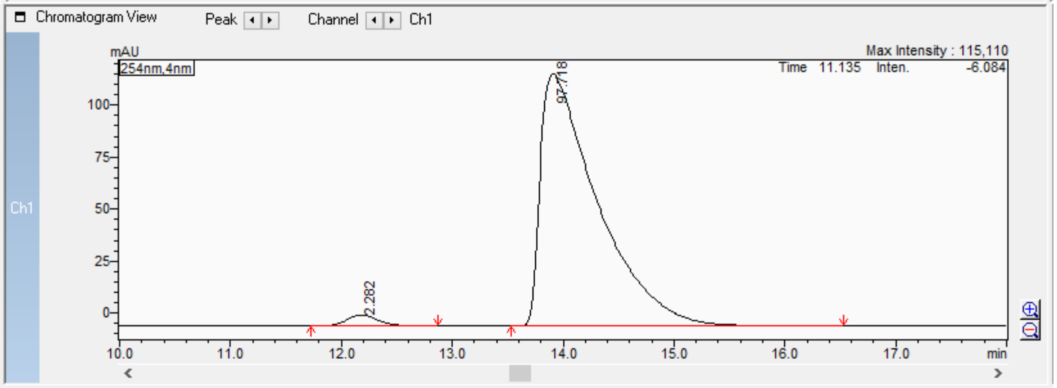

Results View - Peak Table

Peak Table | Compound | Group | Calibration Curve

Peak#	Ret. Time	Area%
1	12.175	2.282
2	13.911	97.718
Total		100.000

Extracted 190nm | Ch1 254nm

LC charts of racemic (top) and enantioenriched (bottom) compound **14**. ChiralCel® OD-H 250 x 4.6 mm ID Analytical column with a column temperature of 40 °C, flow rate 1.0 mL/min, 5% *i*PrOH in hexanes, isocratic.

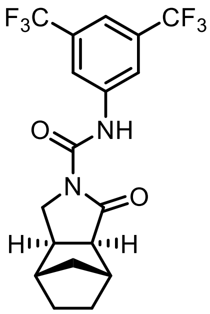

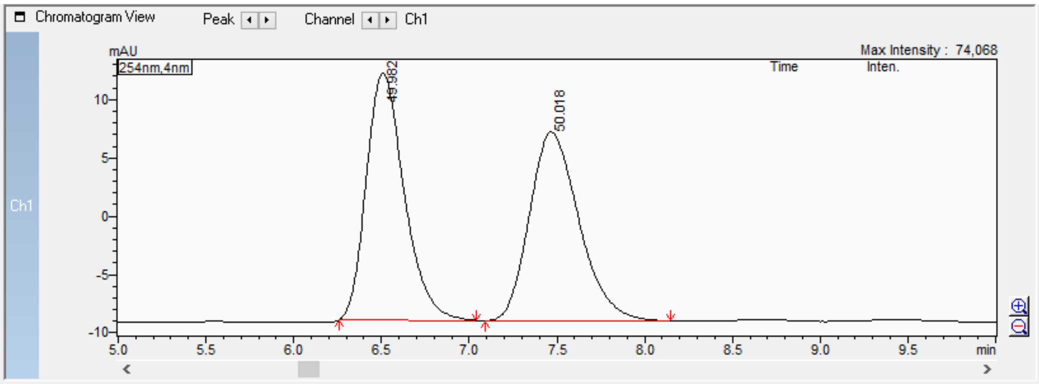

Results View - Peak Table

Peak Table | Compound | Group | Calibration Curve

Peak#	Ret. Time	Area%
1	6.509	49.982
2	7.468	50.018
Total		100.000

Extracted 190nm | Ch1 254nm

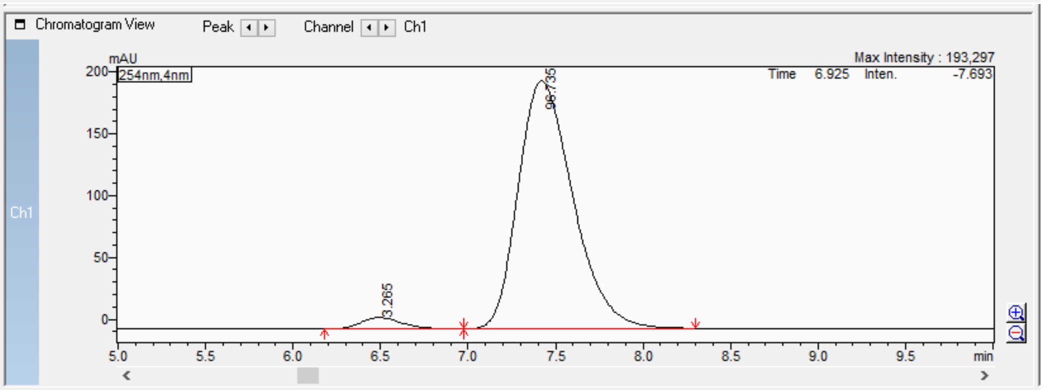

Results View - Peak Table

Peak Table | Compound | Group | Calibration Curve

Peak#	Ret. Time	Area%
1	6.492	3.265
2	7.421	96.735
Total		100.000

Extracted 190nm | Ch1 254nm

LC charts of racemic (top) and enantioenriched (bottom) compound **15**. Chiralpak® OD-H 250 x 4.6 mm ID Analytical column with a column temperature of 40 °C, flow rate 1.0 mL/min, 1% *i*PrOH in CO_2, isocratic.

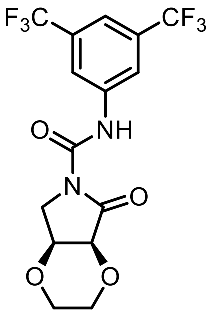

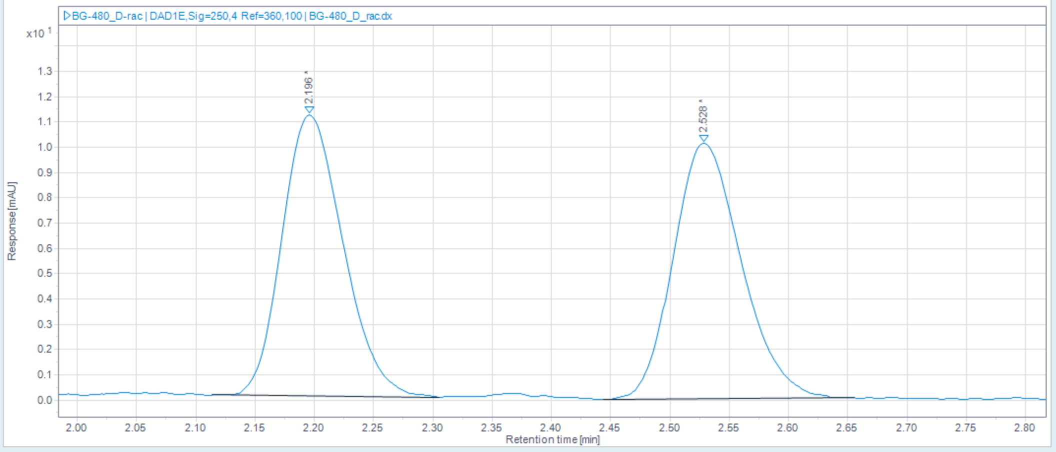

Injection Results

Peaks | Summary

#	Signal description	RT (min)	Area (mAU·s)	Area%	Height (mAU)	Height%	Amount	Concentration	Start time (min)	End time (min)
1	DAD1E,Sig=250,4 Ref=360,100	2.196	40.078	49.819	11.110	52.34			2.114	2.305
2	DAD1E,Sig=250,4 Ref=360,100	2.528	40.368	50.181	10.119	47.66			2.444	2.655

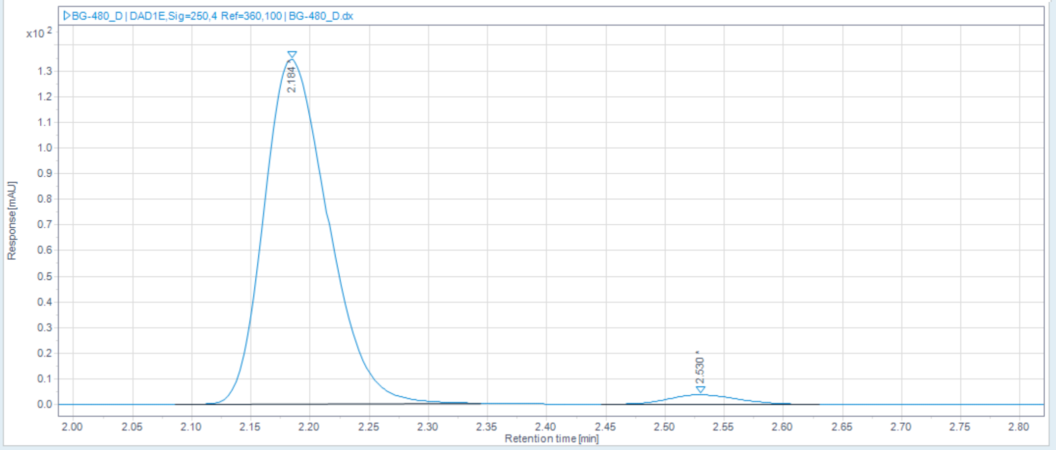

Injection Results

Peaks | Summary

#	Signal description	RT (min)	Area (mAU·s)	Area%	Height (mAU)	Height%	Amount	Concentration	Start time (min)	End time (min)
1	DAD1E,Sig=250,4 Ref=360,100	2.184	503.432	97.114	134.315	97.34			2.086	2.344
2	DAD1E,Sig=250,4 Ref=360,100	2.530	14.959	2.886	3.675	2.66			2.446	2.631

SFC charts of racemic (top) and enantioenriched (bottom) compound **16**. Chiralpak® IB N-3 150 x 4.6 mm ID Analytical column with a column temperature of 40 °C, flow rate 2.75 mL/min, 3% MeOH in CO_2, isocratic.

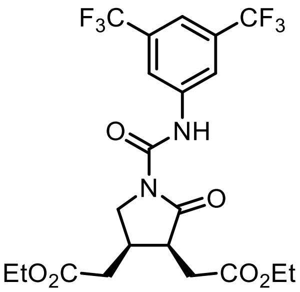

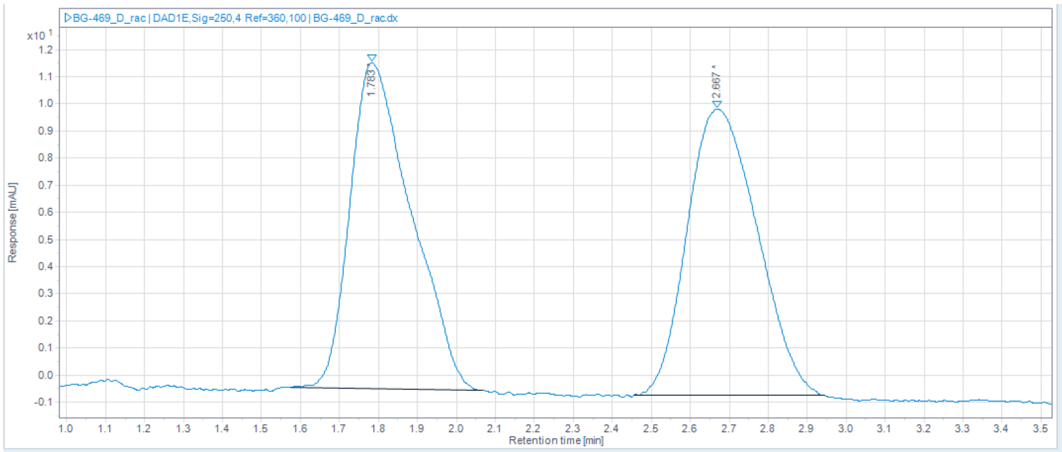

Injection Results

Peaks Summary

#	Signal description	RT (min)	Area (mAU·s)	Area%	Height (mAU)	Height%	Amount	Concentration	Start time (min)	End time (min)
1	DAD1E,Sig=250,4 Ref=360,100	1.783	130.870	49.761	12.001	53.19			1.574	2.066
2	DAD1E,Sig=250,4 Ref=360,100	2.667	132.126	50.239	10.561	46.81			2.454	2.943

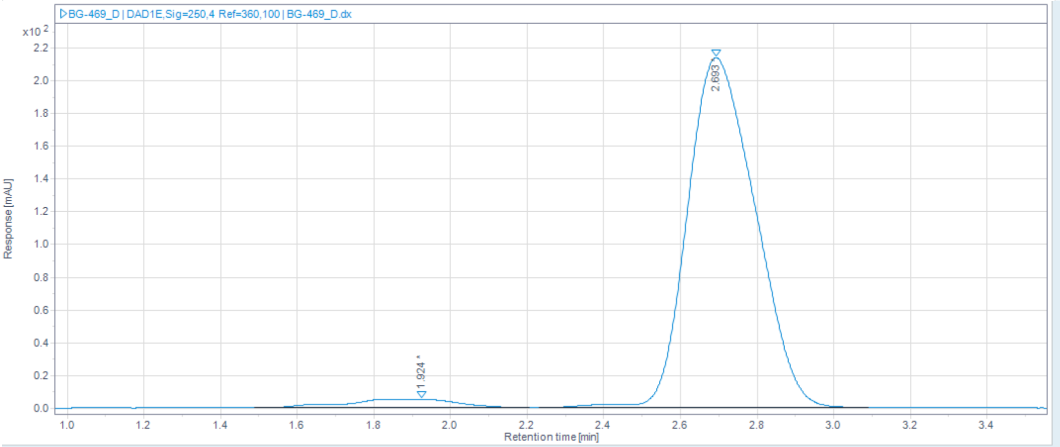

Injection Results

Peaks Summary

#	Signal description	RT (min)	Area (mAU·s)	Area%	Height (mAU)	Height%	Amount	Concentration	Start time (min)	End time (min)
1	DAD1E,Sig=250,4 Ref=360,100	1.924	107.983	3.958	5.321	2.43			1.487	2.205
2	DAD1E,Sig=250,4 Ref=360,100	2.693	2620.318	96.042	213.634	97.57			2.231	3.090

SFC charts of racemic (top) and enantioenriched (bottom) compound **17**. Chiralpak® IC-3 150 x 4.6 mm ID Analytical column with a column temperature of 40 °C, flow rate 2.75 mL/min, 2% *i*PrOH in CO_2, isocratic.

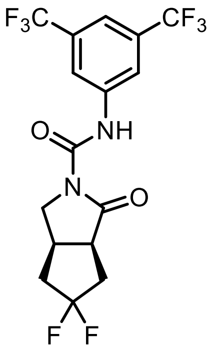

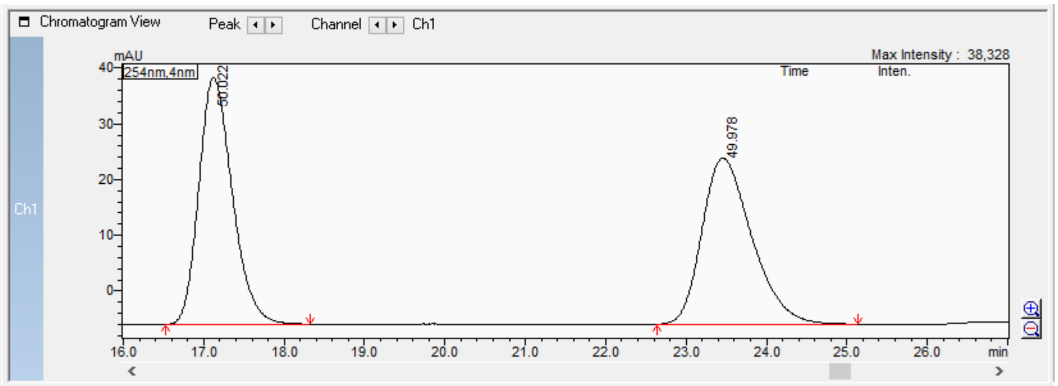

Results View - Peak Table

Peak Table | Compound | Group | Calibration Curve

Peak#	Ret. Time	Area%
1	17.124	50.022
2	23.458	49.978
Total		100.000

Extracted 190nm | Ch1 254nm

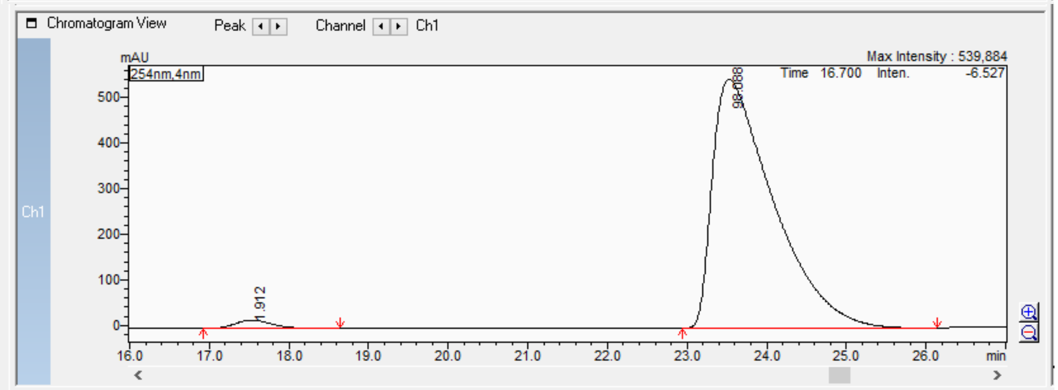

Results View - Peak Table

Peak Table | Compound | Group | Calibration Curve

Peak#	Ret. Time	Area%
1	17.532	1.912
2	23.531	98.088
Total		100.000

Extracted 190nm | Ch1 254nm

LC charts of racemic (top) and enantioenriched (bottom) compound **18**. Chiralpak® OD-H 250 x 4.6 mm ID Analytical column with a column temperature of 40 °C, flow rate 1.0 mL/min, 2% *i*PrOH in hexanes, isocratic.

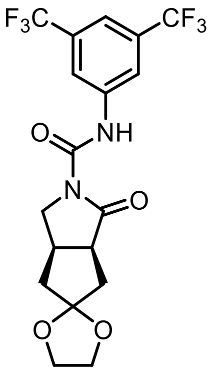

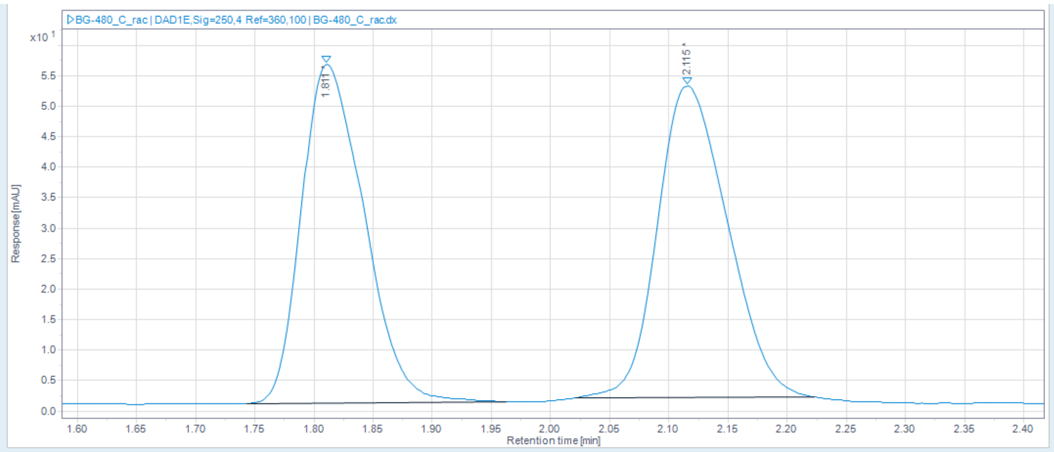

Injection Results

Peaks | Summary

#	Signal description	RT (min)	Area (mAU·s)	Area%	Height (mAU)	Height%	Amount	Concentration	Start time (min)	End time (min)
1	DAD1E,Sig=250,4 Ref=360,100	1.811	204.105	49.363	55.557	52.11			1.743	1.962
2	DAD1E,Sig=250,4 Ref=360,100	2.115	209.370	50.637	51.049	47.89			2.022	2.222

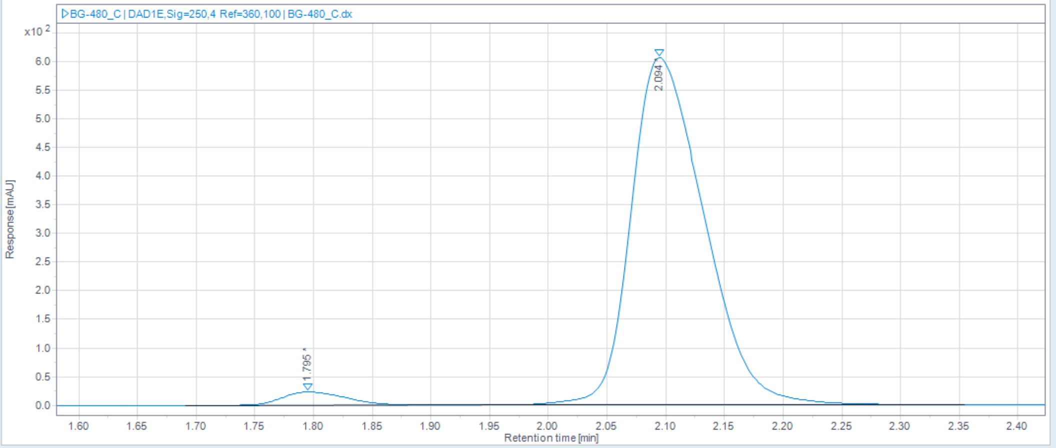

Injection Results

Peaks | Summary

#	Signal description	RT (min)	Area (mAU·s)	Area%	Height (mAU)	Height%	Amount	Concentration	Start time (min)	End time (min)
1	DAD1E,Sig=250,4 Ref=360,100	1.795	86.417	3.170	23.163	3.68			1.691	1.944
2	DAD1E,Sig=250,4 Ref=360,100	2.094	2639.423	96.830	607.104	96.32			1.944	2.354

SFC charts of racemic (top) and enantioenriched (bottom) compound **19**. Chiralpak® IB N-3 150 x 4.6 mm ID Analytical column with a column temperature of 40 °C, flow rate 2.75 mL/min, 3% MeOH in CO_2, isocratic.

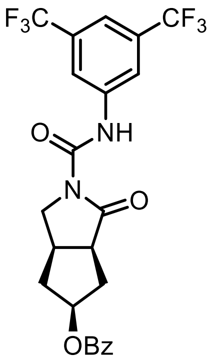

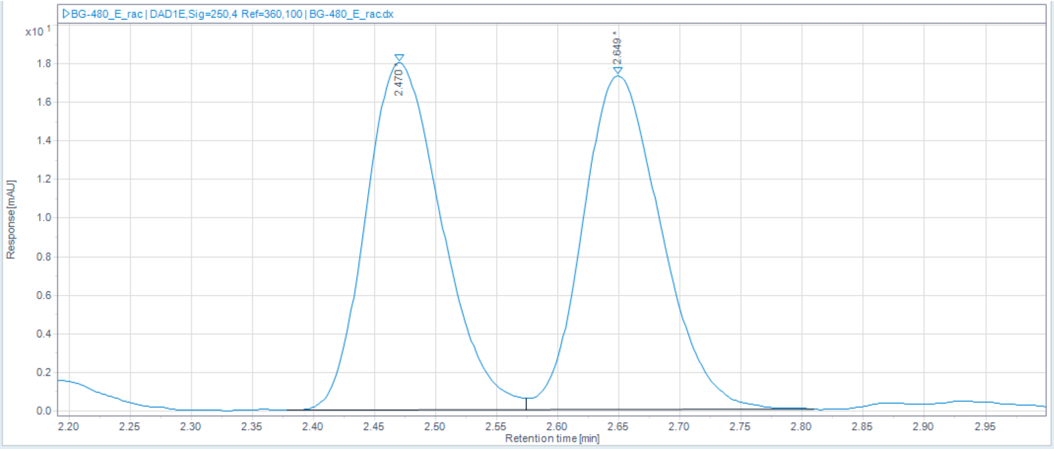

Injection Results

Peaks Summary

#	Signal description	RT (min)	Area (mAU·s)	Area%	Height (mAU)	Height%	Amount	Concentration	Start time (min)	End time (min)
1	DAD1E,Sig=250,4 Ref=360,100	2.470	77.935	50.187	18.029	50.99			2.378	2.574
2	DAD1E,Sig=250,4 Ref=360,100	2.649	77.353	49.813	17.330	49.01			2.574	2.809

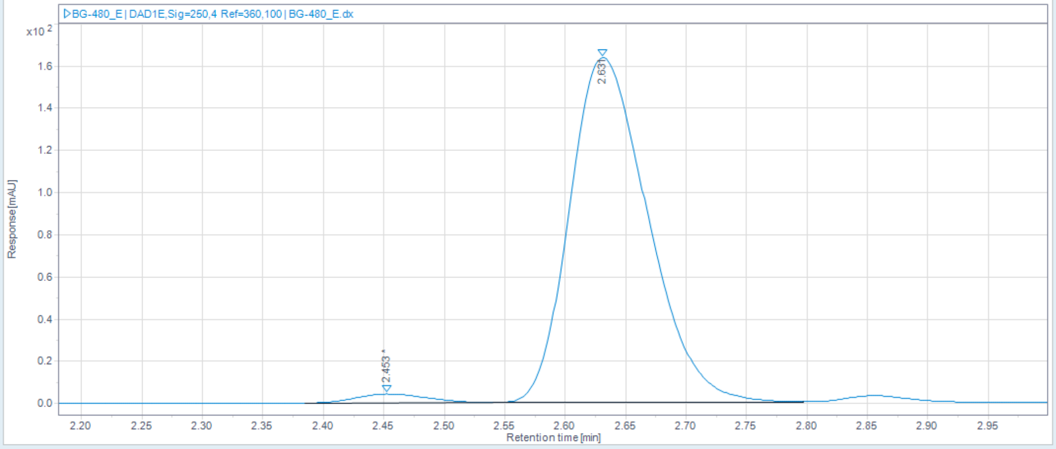

Injection Results

Peaks Summary

#	Signal description	RT (min)	Area (mAU·s)	Area%	Height (mAU)	Height%	Amount	Concentration	Start time (min)	End time (min)
1	DAD1E,Sig=250,4 Ref=360,100	2.453	17.529	2.315	4.287	2.55			2.384	2.540
2	DAD1E,Sig=250,4 Ref=360,100	2.631	739.569	97.685	163.624	97.45			2.540	2.797

SFC charts of racemic (top) and enantioenriched (bottom) compound **20**. Chiralpak® IB N-3 150 x 4.6 mm ID Analytical column with a column temperature of 40 °C, flow rate 2.75 mL/min, 3% MeOH in CO_2, isocratic.

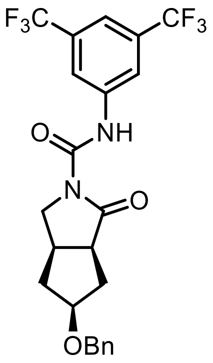

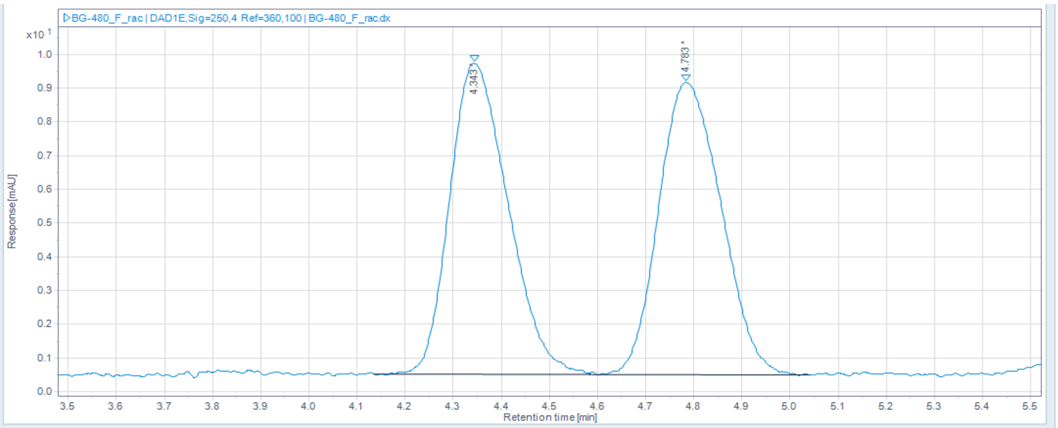

Injection Results

Peaks | Summary

#	Signal description	RT (min)	Area (mAU·s)	Area%	Height (mAU)	Height%	Amount	Concentration	Start time (min)	End time (min)
1	DAD1E,Sig=250,4 Ref=360,100	4.343	77.767	49.780	9.220	51.51			4.135	4.583
2	DAD1E,Sig=250,4 Ref=360,100	4.783	78.456	50.220	8.678	48.49			4.583	5.043

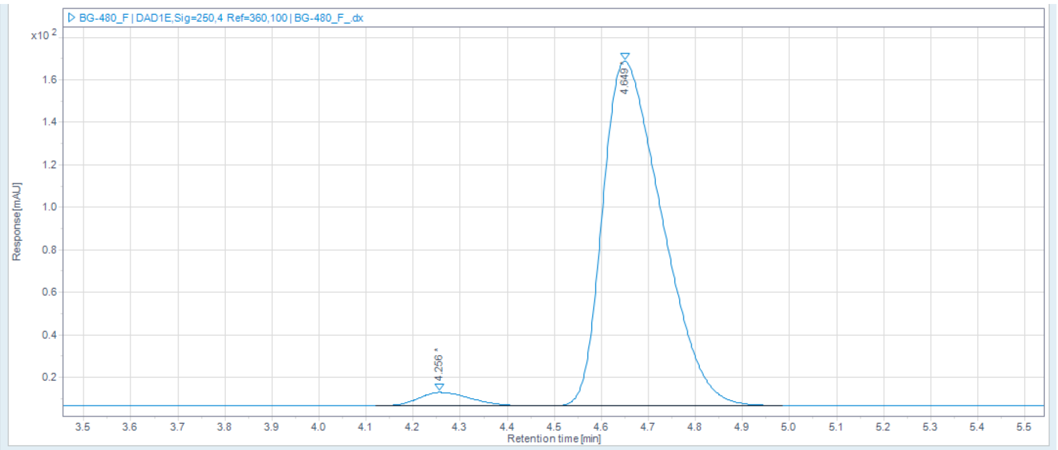

Injection Results

Peaks | Summary

#	Signal description	RT (min)	Area (mAU·s)	Area%	Height (mAU)	Height%	Amount	Concentration	Start time (min)	End time (min)
1	DAD1E,Sig=250,4 Ref=360,100	4.256	49.405	3.374	6.361	3.77			4.121	4.465
2	DAD1E,Sig=250,4 Ref=360,100	4.649	1414.706	96.626	162.206	96.23			4.465	4.985

SFC charts of racemic (top) and enantioenriched (bottom) compound **21**. Chiralpak® IB N-3 150 x 4.6 mm ID Analytical column with a column temperature of 40 °C, flow rate 2.75 mL/min, 1% MeOH in CO_2, isocratic.

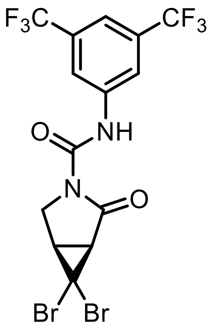

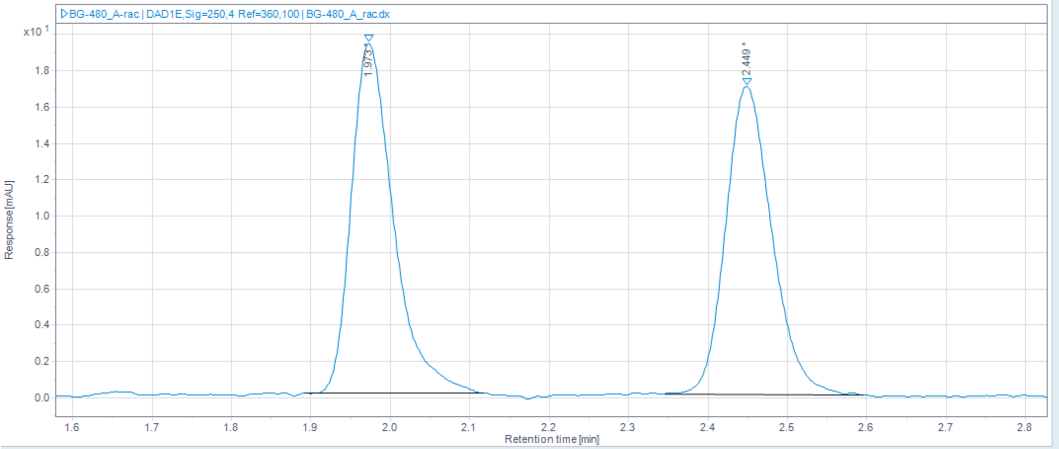

Injection Results

Peaks | Summary

#	Signal description	RT (min)	Area (mAU·s)	Area%	Height (mAU)	Height%	Amount	Concentration	Start time (min)	End time (min)
1	DAD1E,Sig=250,4 Ref=360,100	1.973	70.960	50.766	19.251	53.14			1.893	2.118
2	DAD1E,Sig=250,4 Ref=360,100	2.449	68.817	49.234	16.976	46.86			2.345	2.596

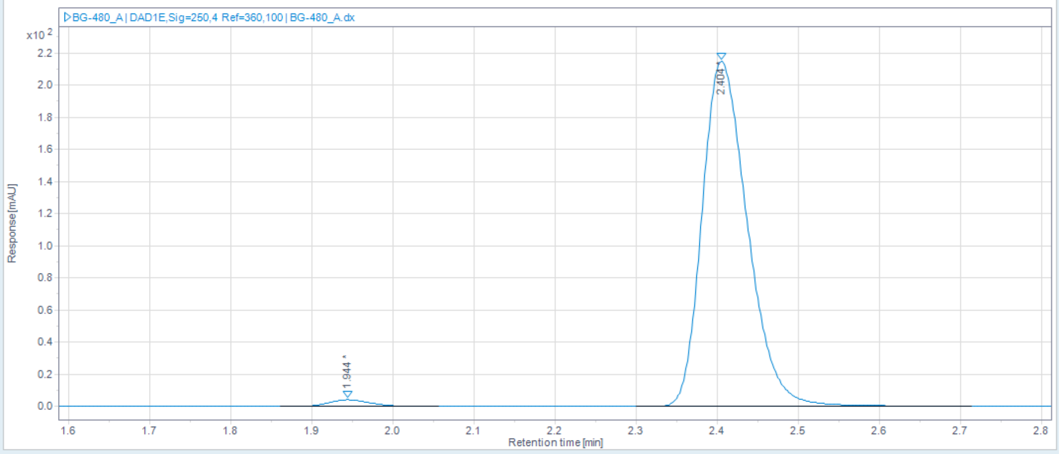

Injection Results

Peaks | Summary

#	Signal description	RT (min)	Area (mAU·s)	Area%	Height (mAU)	Height%	Amount	Concentration	Start time (min)	End time (min)
1	DAD1E,Sig=250,4 Ref=360,100	1.944	13.970	1.644	4.059	1.85			1.860	2.056
2	DAD1E,Sig=250,4 Ref=360,100	2.404	835.937	98.356	214.877	98.15			2.299	2.713

SFC charts of racemic (top) and enantioenriched (bottom) compound **22**. Chiralpak® IB N-3 150 x 4.6 mm ID Analytical column with a column temperature of 40 °C, flow rate 2.75 mL/min, 3% MeOH in CO_2, isocratic.

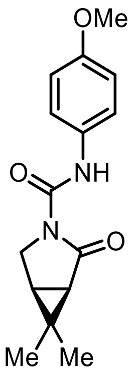

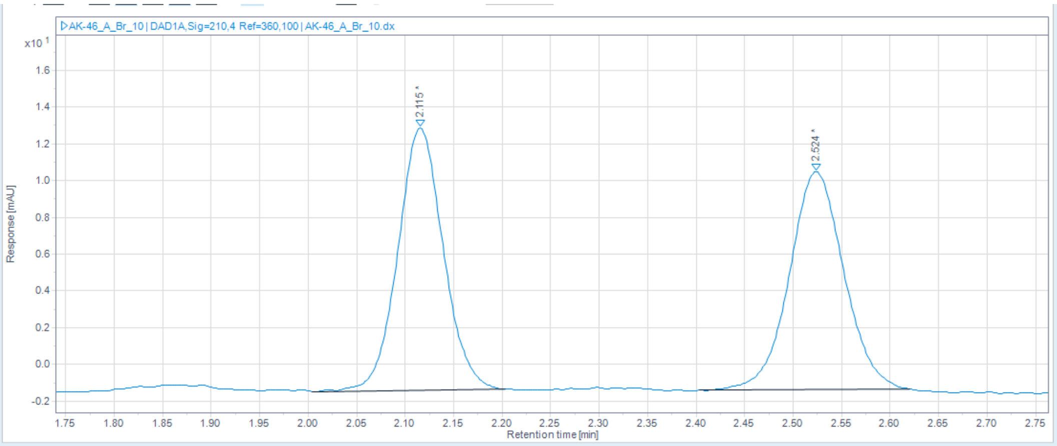

Injection Results

Peaks | Summary

#	Signal description	RT (min)	Area (mAU·s)	Area%	Height (mAU)	Height%	Amount	Concentration	Start time (min)	End time (min)
1	DAD1A,Sig=210,4 Ref=360,100	2.115	46.364	50.005	14.253	54.59			2.004	2.203
2	DAD1A,Sig=210,4 Ref=360,100	2.524	46.355	49.995	11.856	45.41			2.403	2.620

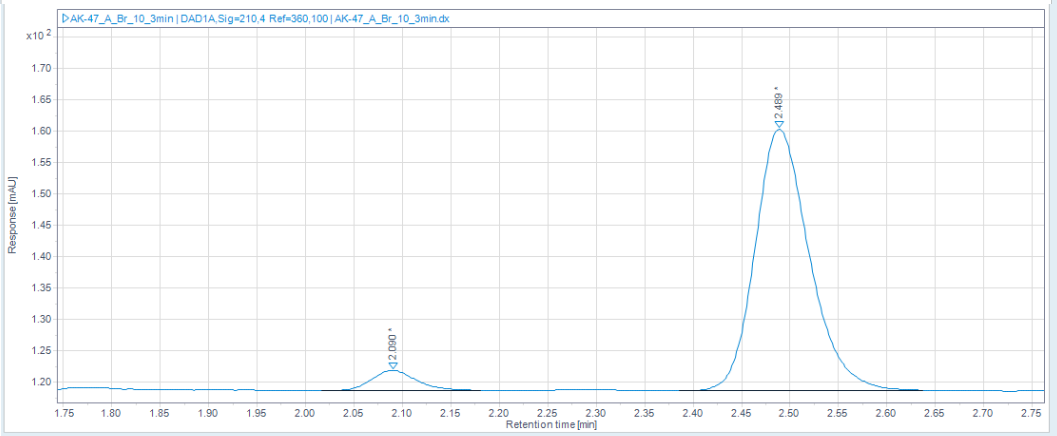

Injection Results

Peaks | Summary

#	Signal description	RT (min)	Area (mAU·s)	Area%	Height (mAU)	Height%	Amount	Concentration	Start time (min)	End time (min)
1	DAD1A,Sig=210,4 Ref=360,100	2.090	10.437	6.054	3.218	7.17			2.016	2.180
2	DAD1A,Sig=210,4 Ref=360,100	2.489	161.976	93.946	41.657	92.83			2.385	2.637

SFC charts of racemic (top) and enantioenriched (bottom) compound **6-OMe**. Chiralpak® IA-3 150 x 4.6 mm ID Analytical column with a column temperature of 40 °C, flow rate 2.75 mL/min, 10% MeOH in CO_2, isocratic.

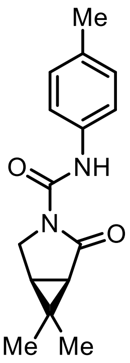

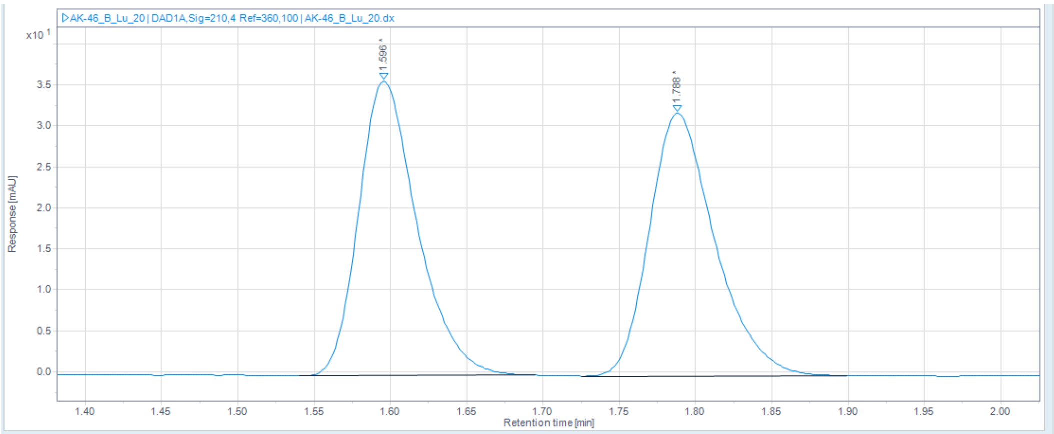

Injection Results

Peaks | Summary

#	Signal description	RT (min)	Area (mAU·s)	Area%	Height (mAU)	Height%	Amount	Concentration	Start time (min)	End time (min)
1	DAD1A,Sig=210,4 Ref=360,100	1.596	95.793	49.946	35.920	52.82			1.540	1.695
2	DAD1A,Sig=210,4 Ref=360,100	1.788	96.001	50.054	32.079	47.18			1.725	1.899

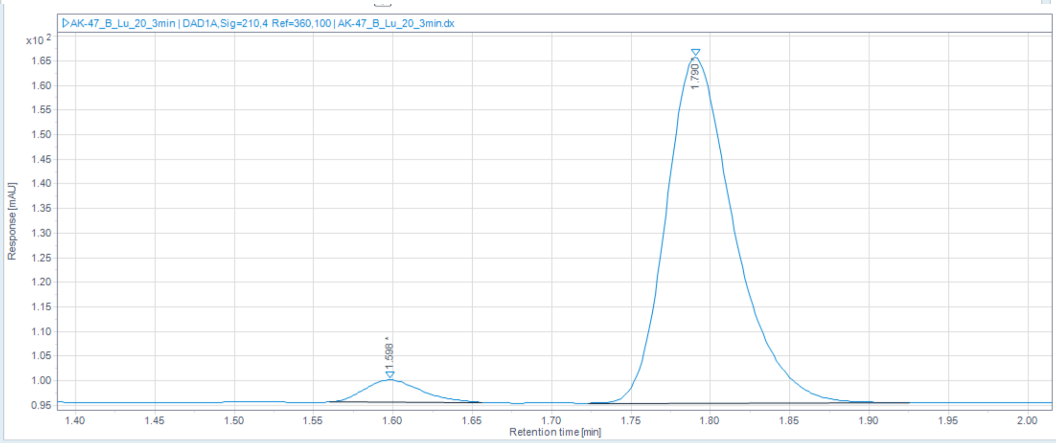

Injection Results

Peaks | Summary

#	Signal description	RT (min)	Area (mAU·s)	Area%	Height (mAU)	Height%	Amount	Concentration	Start time (min)	End time (min)
1	DAD1A,Sig=210,4 Ref=360,100	1.598	11.134	5.058	4.507	6.03			1.560	1.656
2	DAD1A,Sig=210,4 Ref=360,100	1.790	208.974	94.942	70.186	93.97			1.723	1.925

SFC charts of racemic (top) and enantioenriched (bottom) compound **6-Me**. Chiralpak® IC-3 150 x 4.6 mm ID Analytical column with a column temperature of 40 °C, flow rate 2.75 mL/min, 20% MeOH in CO_2, isocratic.

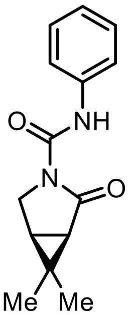

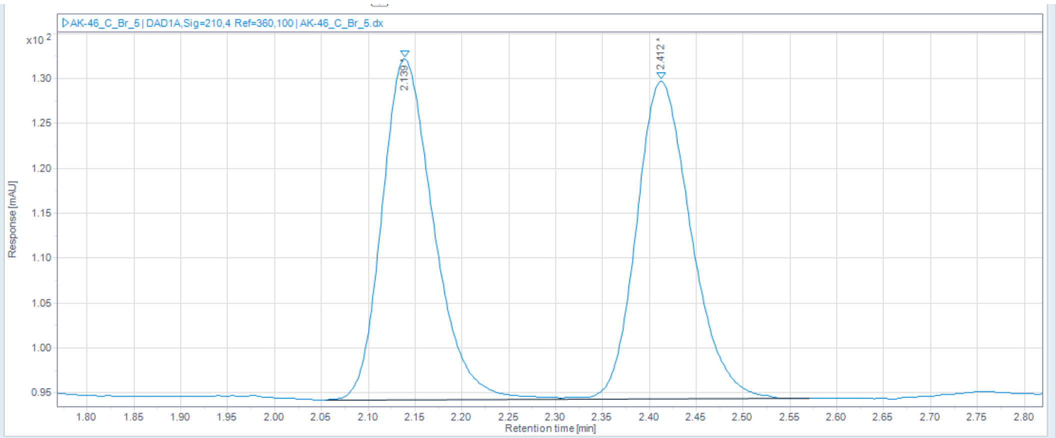

Injection Results

Peaks | Summary

#	Signal description	RT (min)	Area (mAU·s)	Area%	Height (mAU)	Height%	Amount	Concentration	Start time (min)	End time (min)
1	DAD1A,Sig=210,4 Ref=360,100	2.139	144.848	50.322	37.935	51.72			2.055	2.305
2	DAD1A,Sig=210,4 Ref=360,100	2.412	142.991	49.678	35.407	48.28			2.305	2.571

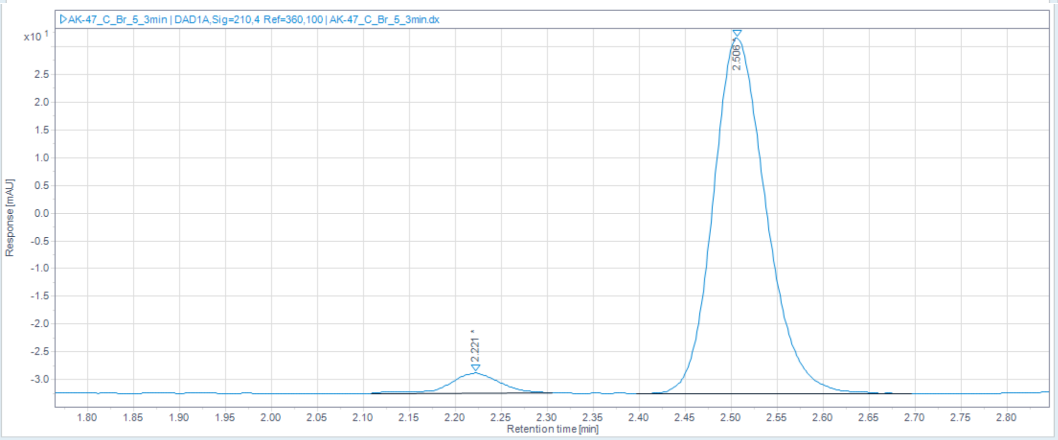

Injection Results

Peaks | Summary

#	Signal description	RT (min)	Area (mAU·s)	Area%	Height (mAU)	Height%	Amount	Concentration	Start time (min)	End time (min)
1	DAD1A,Sig=210,4 Ref=360,100	2.221	13.377	4.971	3.581	5.29			2.108	2.306
2	DAD1A,Sig=210,4 Ref=360,100	2.506	255.727	95.029	64.093	94.71			2.397	2.696

SFC charts of racemic (top) and enantioenriched (bottom) compound **6-H**. Chiralpak® IA-3 150 x 4.6 mm ID Analytical column with a column temperature of 40 °C, flow rate 2.75 mL/min, 5% MeOH in CO_2, isocratic.

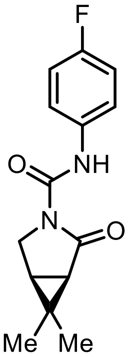

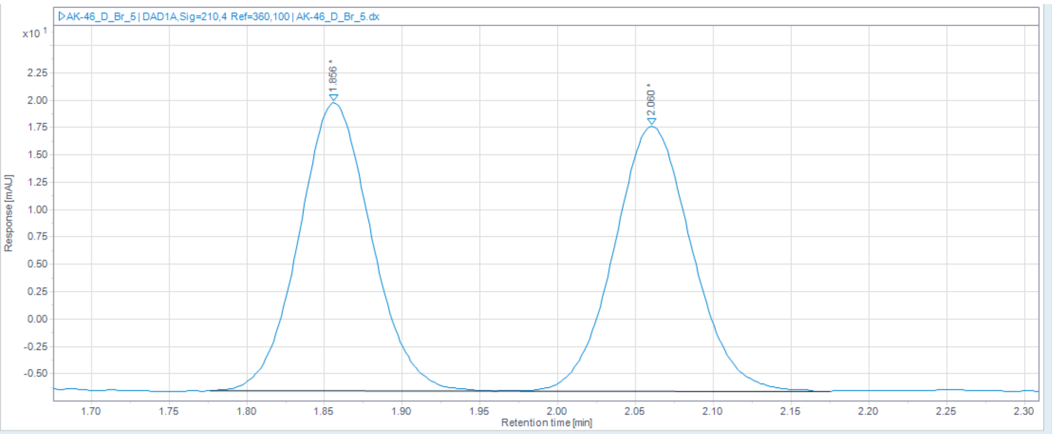

Injection Results

Peaks | Summary

#	Signal description	RT (min)	Area (mAU·s)	Area%	Height (mAU)	Height%	Amount	Concentration	Start time (min)	End time (min)
1	DAD1A,Sig=210,4 Ref=360,100	1.856	85.038	50.346	26.289	52.10			1.776	1.963
2	DAD1A,Sig=210,4 Ref=360,100	2.060	83.870	49.654	24.167	47.90			1.963	2.175

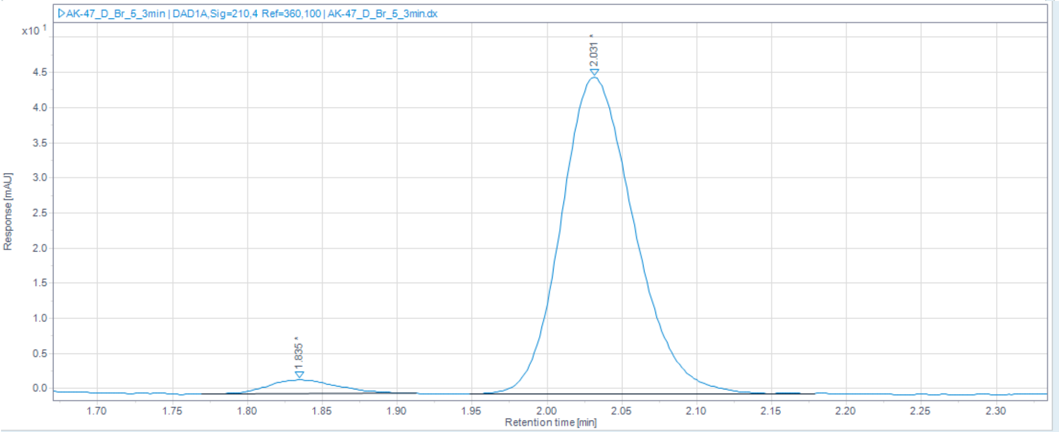

Injection Results

Peaks | Summary

#	Signal description	RT (min)	Area (mAU·s)	Area%	Height (mAU)	Height%	Amount	Concentration	Start time (min)	End time (min)
1	DAD1A,Sig=210,4 Ref=360,100	1.835	6.707	4.220	1.999	4.25			1.769	1.913
2	DAD1A,Sig=210,4 Ref=360,100	2.031	152.231	95.780	45.027	95.75			1.948	2.179

SFC charts of racemic (top) and enantioenriched (bottom) compound **6-F**. Chiralpak® IA-3 150 x 4.6 mm ID Analytical column with a column temperature of 40 °C, flow rate 2.75 mL/min, 5% MeOH in CO_2, isocratic.

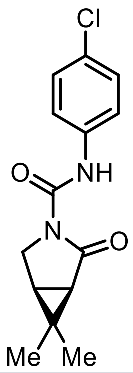

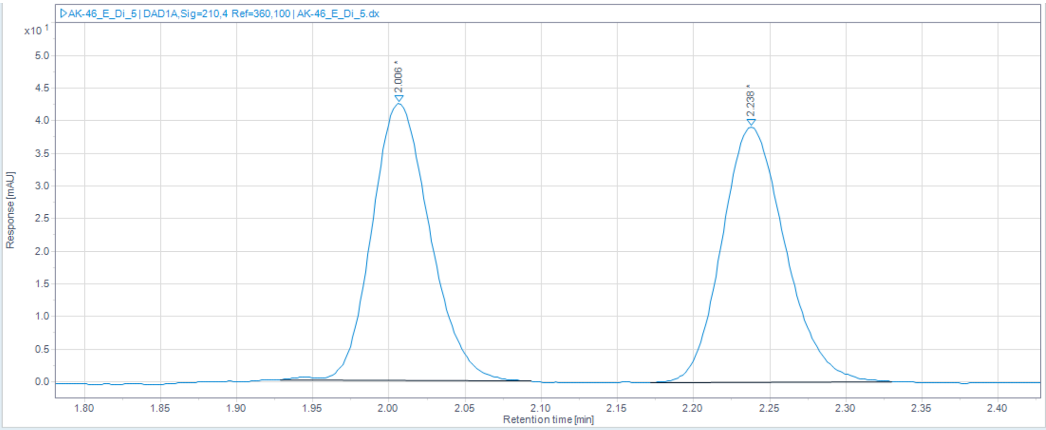

Injection Results

Peaks | Summary

#	Signal description	RT (min)	Area (mAU·s)	Area%	Height (mAU)	Height%	Amount	Concentration	Start time (min)	End time (min)
1	DAD1A,Sig=210,4 Ref=360,100	2.006	112.136	49.872	42.433	52.05			1.929	2.094
2	DAD1A,Sig=210,4 Ref=360,100	2.238	112.710	50.128	39.086	47.95			2.171	2.330

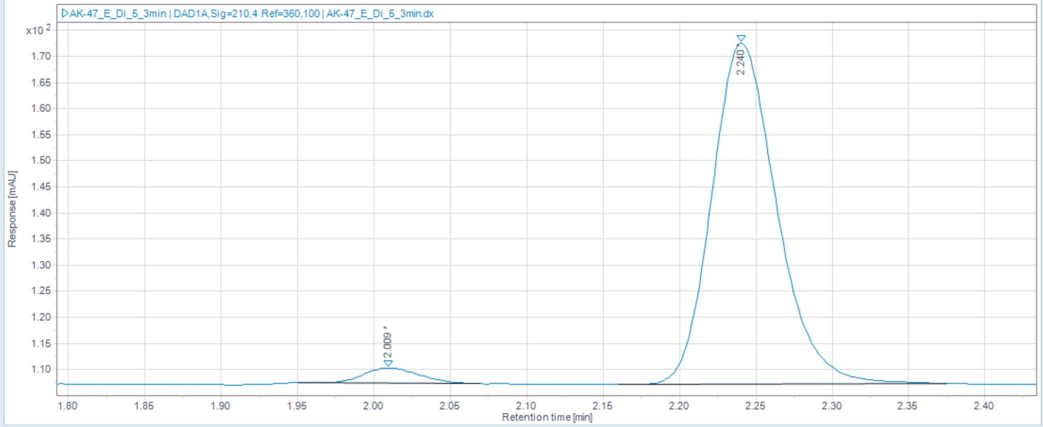

Injection Results

Peaks | Summary

#	Signal description	RT (min)	Area (mAU·s)	Area%	Height (mAU)	Height%	Amount	Concentration	Start time (min)	End time (min)
1	DAD1A,Sig=210,4 Ref=360,100	2.009	7.546	3.778	2.911	4.27			1.951	2.070
2	DAD1A,Sig=210,4 Ref=360,100	2.240	192.170	96.222	65.294	95.73			2.160	2.375

SFC charts of racemic (top) and enantioenriched (bottom) compound **6-Cl**. Chiralpak® IB N-3 150 x 4.6 mm ID Analytical column with a column temperature of 40 °C, flow rate 2.75 mL/min, 5% MeOH in CO_2, isocratic

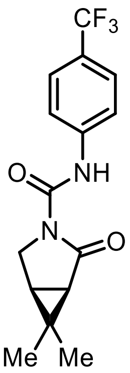

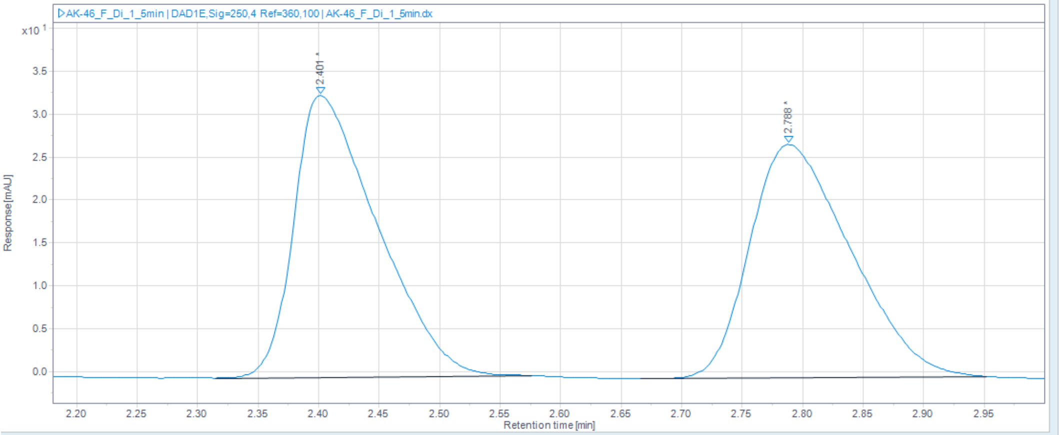

Injection Results

Peaks | Summary

#	Signal description	RT (min)	Area (mAU·s)	Area%	Height (mAU)	Height%	Amount	Concentration	Start time (min)	End time (min)
1	DAD1E,Sig=250,4 Ref=360,100	2.401	157.587	50.087	32.805	54.70			2.315	2.576
2	DAD1E,Sig=250,4 Ref=360,100	2.788	157.040	49.913	27.166	45.30			2.666	2.952

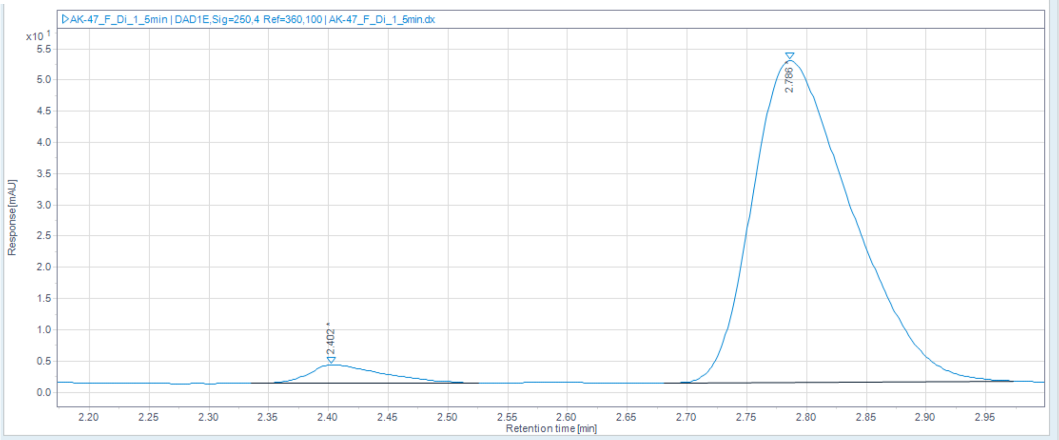

Injection Results

Peaks | Summary

#	Signal description	RT (min)	Area (mAU·s)	Area%	Height (mAU)	Height%	Amount	Concentration	Start time (min)	End time (min)
1	DAD1E,Sig=250,4 Ref=360,100	2.402	12.649	4.084	2.956	5.42			2.335	2.526
2	DAD1E,Sig=250,4 Ref=360,100	2.786	297.086	95.916	51.552	94.58			2.681	2.973

SFC charts of racemic (top) and enantioenriched (bottom) compound **6-CF3**. Chiralpak® IB N-3 150 x 4.6 mm ID Analytical column with a column temperature of 40 °C, flow rate 2.75 mL/min, 1% MeOH in CO_2, isocratic.

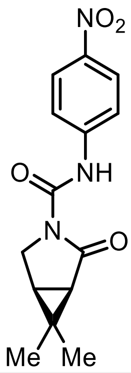

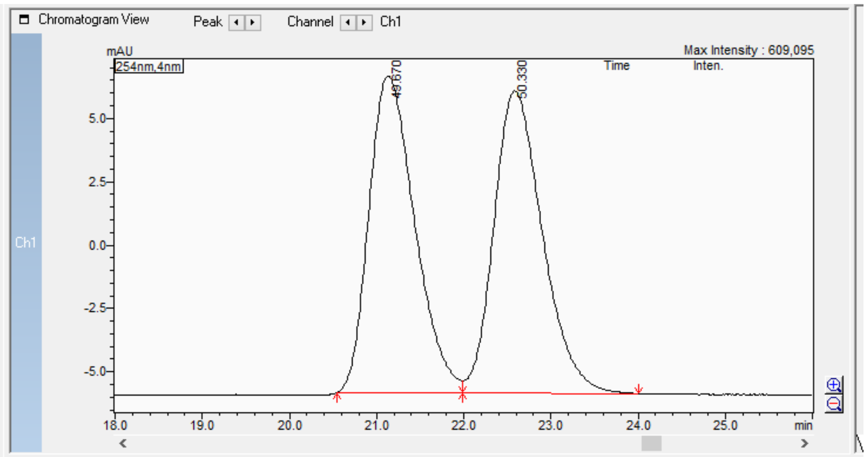

Results View - Peak Table

Peak Table | Compound | Group | Calibration Curve

Peak#	Ret. Time	Area%
1	21.131	49.670
2	22.583	50.330
Total		100.000

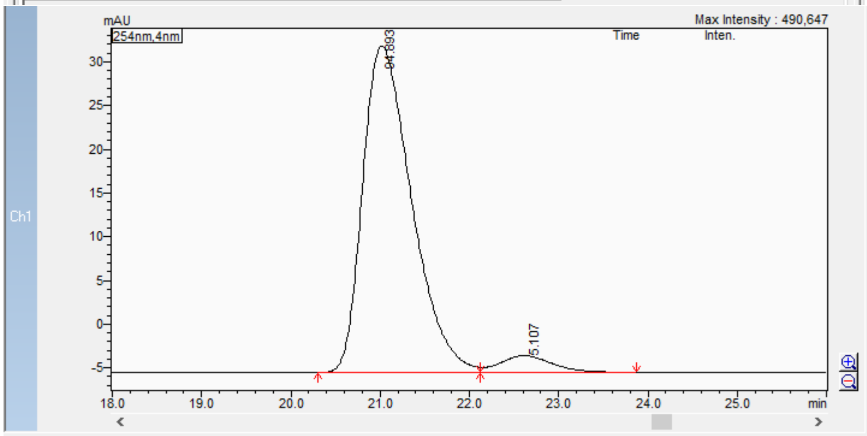

Results View - Peak Table

Peak Table | Compound | Group | Calibration Curve

Peak#	Ret. Time	Area%
1	21.022	94.893
2	22.628	5.107
Total		100.000

LC charts of racemic (top) and enantioenriched (bottom) compound **6-NO$_2$**. ChiralCel® OD-H 250 x 4.6 mm ID Analytical column with a column temperature of 40 °C, flow rate 1.0 mL/min, 5% *i*PrOH in hexanes, isocratic.

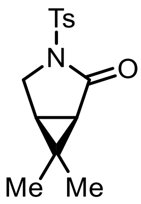

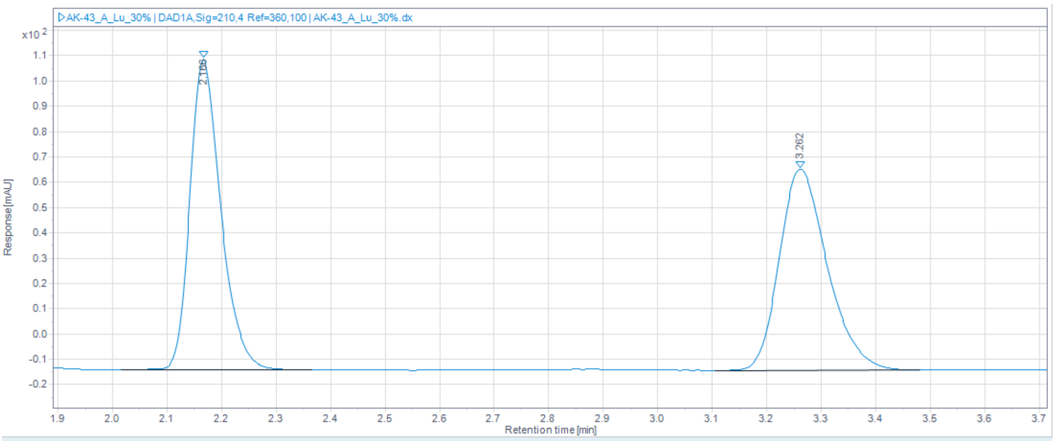

Injection Results

Peaks | Summary

#	Signal description	RT (min)	Area (mAU·s)	Area%	Height (mAU)	Height%	Amount	Concentration	Start time (min)	End time (min)
1	DAD1A,Sig=210,4 Ref=360,100	2.166	486.474	49.965	122.831	60.69			2.017	2.366
2	DAD1A,Sig=210,4 Ref=360,100	3.262	487.160	50.035	79.557	39.31			3.105	3.481

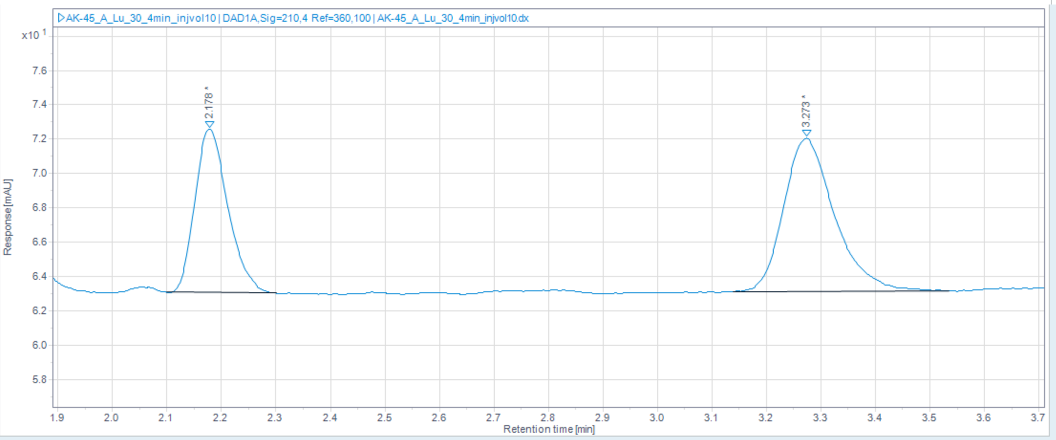

Injection Results

Peaks | Summary

#	Signal description	RT (min)	Area (mAU·s)	Area%	Height (mAU)	Height%	Amount	Concentration	Start time (min)	End time (min)
1	DAD1A,Sig=210,4 Ref=360,100	2.178	40.095	41.019	9.537	51.68			2.099	2.301
2	DAD1A,Sig=210,4 Ref=360,100	3.273	57.652	58.981	8.918	48.32			3.138	3.536

SFC charts of racemic (top) and enantioenriched (bottom) compound **2-Ts**. Chiralpak® IC-3 150 x 4.6 mm ID Analytical column with a column temperature of 40 °C, flow rate 2.75 mL/min, 30% MeOH in CO_2, isocratic.

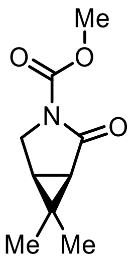

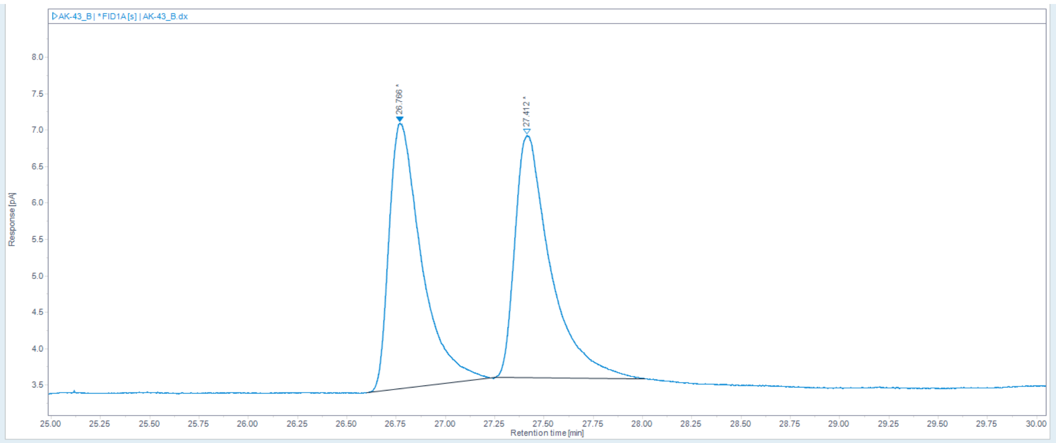

Injection Results

Peaks | Summary

#	Name	Signal description	RT (min)	Area (pA·s)	Area%	Height (pA)	Height%	Amount	Concentration	Start time (min)	End time (min)
1		* FID1A [s]	26.766	41.540	49.977	3.648	52.36			26.599	27.255
2		* FID1A [s]	27.412	41.578	50.023	3.319	47.64			27.255	28.011

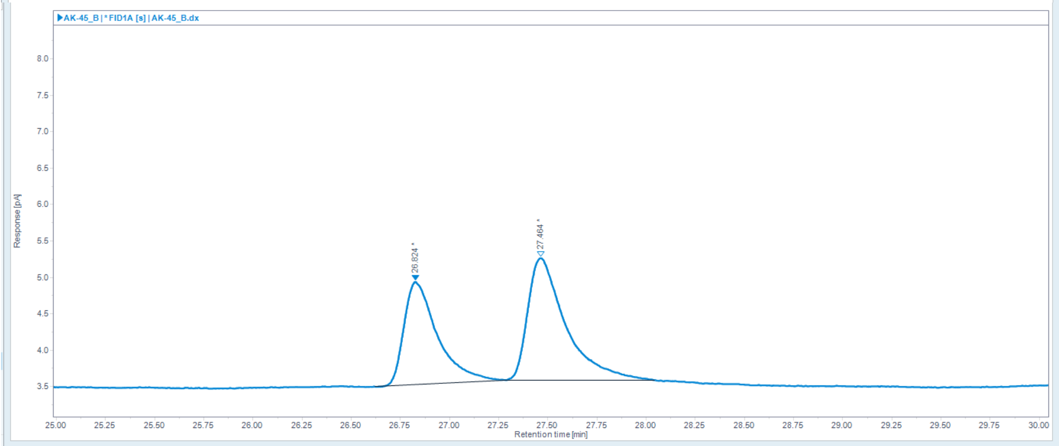

Injection Results

Peaks | Summary

#	Name	Signal description	RT (min)	Area (pA·s)	Area%	Height (pA)	Height%	Amount	Concentration	Start time (min)	End time (min)
1		* FID1A [s]	26.824	16.593	42.508	1.415	45.78			26.618	27.271
2		* FID1A [s]	27.464	22.443	57.492	1.676	54.22			27.271	28.044

GC charts of racemic (top) and enantioenriched (bottom) compound **2-OMe**. J&W CP-ChiraSil-DEX CB GC column, 50 m x 0.25 mm x 0.25 µm, 1.6 mL/min He, 140 °C isocratic.

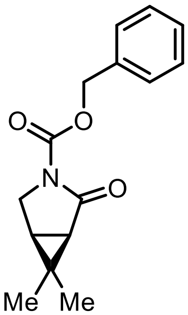

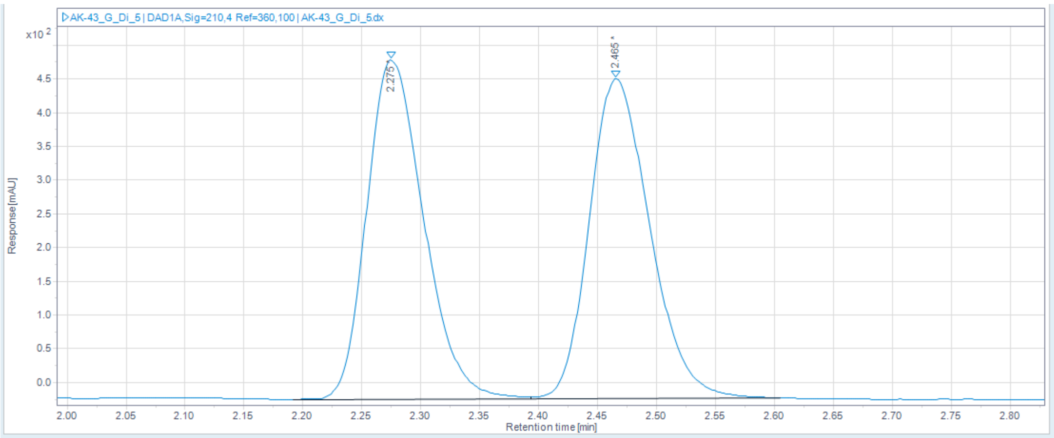

Injection Results

Peaks | Summary

#	Signal description	RT (min)	Area (mAU·s)	Area%	Height (mAU)	Height%	Amount	Concentration	Start time (min)	End time (min)
1	DAD1A,Sig=210,4 Ref=360,100	2.275	1688.765	49.931	502.935	51.49			2.191	2.394
2	DAD1A,Sig=210,4 Ref=360,100	2.465	1693.403	50.069	473.860	48.51			2.394	2.605

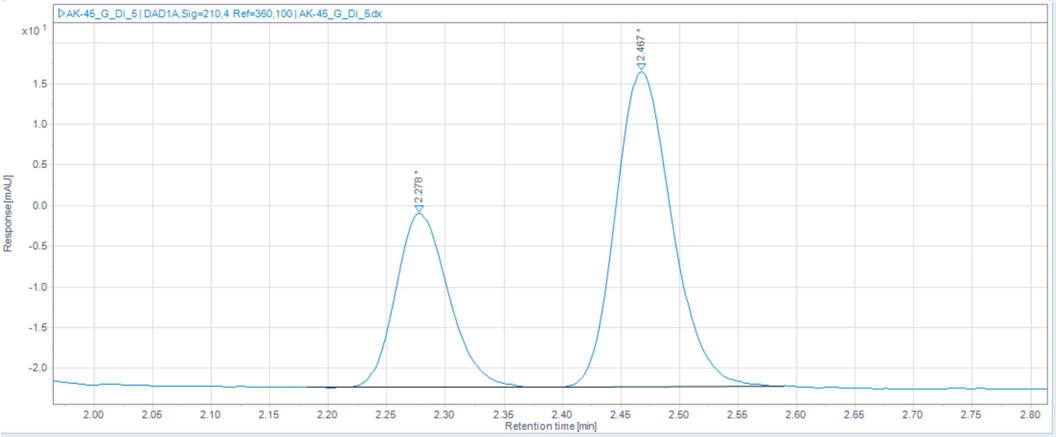

Injection Results

Peaks | Summary

#	Signal description	RT (min)	Area (mAU·s)	Area%	Height (mAU)	Height%	Amount	Concentration	Start time (min)	End time (min)
1	DAD1A,Sig=210,4 Ref=360,100	2.278	66.785	34.048	21.346	35.51			2.182	2.396
2	DAD1A,Sig=210,4 Ref=360,100	2.467	129.362	65.952	38.770	64.49			2.396	2.590

SFC charts of racemic (top) and enantioenriched (bottom) compound **2-OBn**. Chiralpak® IB N-3 150 x 4.6 mm ID Analytical column with a column temperature of 40 °C, flow rate 2.75 mL/min, 5% MeOH in CO_2, isocratic.

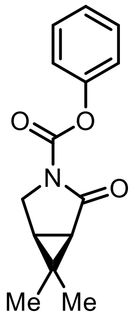

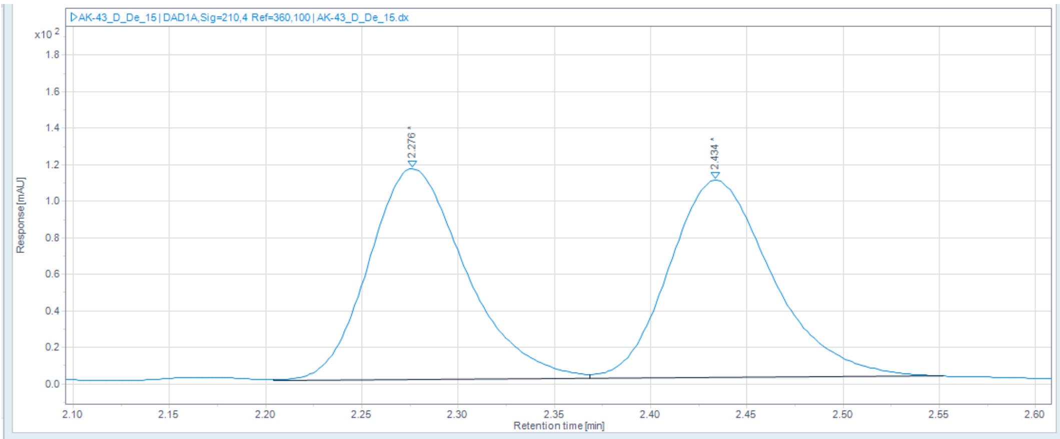

Injection Results

Peaks | Summary

#	Signal description	RT (min)	Area (mAU·s)	Area%	Height (mAU)	Height%	Amount	Concentration	Start time (min)	End time (min)
1	DAD1A,Sig=210,4 Ref=360,100	2.276	404.619	50.063	115.388	51.67			2.204	2.368
2	DAD1A,Sig=210,4 Ref=360,100	2.434	403.594	49.937	107.930	48.33			2.368	2.552

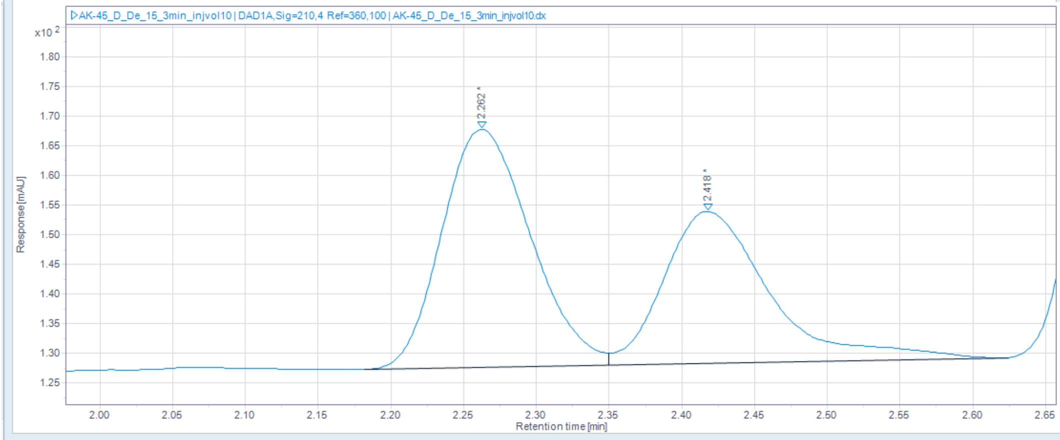

Injection Results

Peaks | Summary

#	Signal description	RT (min)	Area (mAU·s)	Area%	Height (mAU)	Height%	Amount	Concentration	Start time (min)	End time (min)
1	DAD1A,Sig=210,4 Ref=360,100	2.262	170.248	56.739	40.140	61.03			2.182	2.350
2	DAD1A,Sig=210,4 Ref=360,100	2.418	129.809	43.261	25.626	38.97			2.350	2.624

SFC charts of racemic (top) and enantioenriched (bottom) compound **2-OPh**. Chiralpak® IG-3 150 x 4.6 mm ID Analytical column with a column temperature of 40 °C, flow rate 2.75 mL/min, 15% MeOH in CO_2, isocratic.

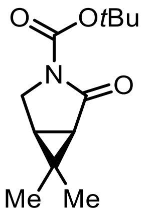

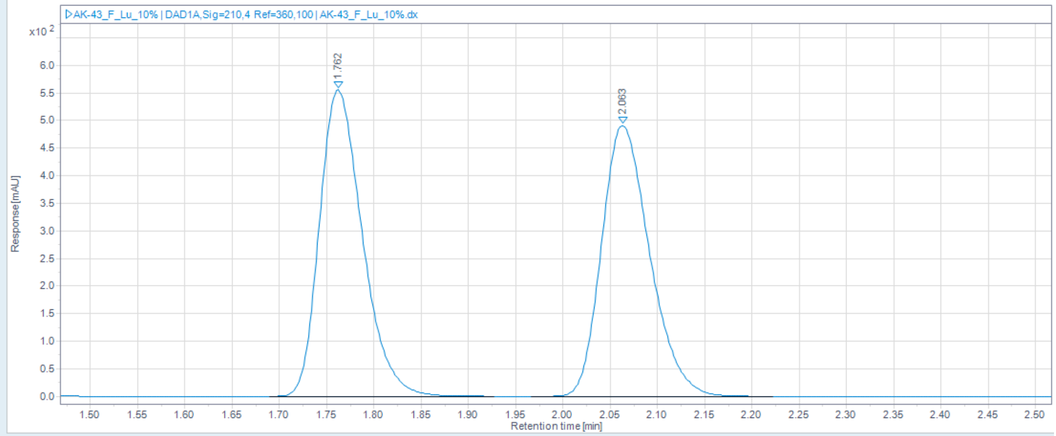

Injection Results

Peaks | Summary

#	Signal description	RT (min)	Area (mAU·s)	Area%	Height (mAU)	Height%	Amount	Concentration	Start time (min)	End time (min)
1	DAD1A,Sig=210,4 Ref=360,100	1.762	1754.651	50.233	555.941	53.08			1.689	1.927
2	DAD1A,Sig=210,4 Ref=360,100	2.063	1738.344	49.767	491.338	46.92			1.967	2.222

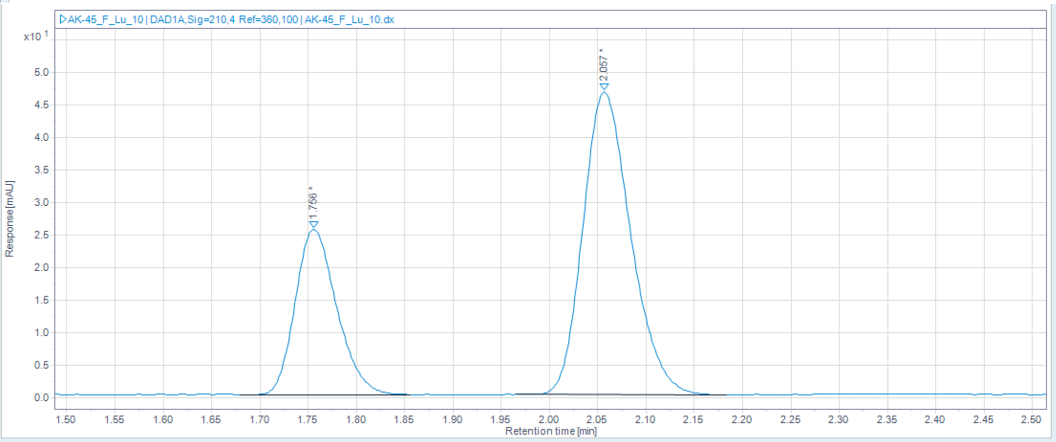

Injection Results

Peaks | Summary

#	Signal description	RT (min)	Area (mAU·s)	Area%	Height (mAU)	Height%	Amount	Concentration	Start time (min)	End time (min)
1	DAD1A,Sig=210,4 Ref=360,100	1.756	76.045	32.106	25.431	35.32			1.680	1.856
2	DAD1A,Sig=210,4 Ref=360,100	2.057	160.809	67.894	46.572	64.68			1.965	2.183

SFC charts of racemic (top) and enantioenriched (bottom) compound **2-O^{t}Bu** from oxidation of 3-O^{t}Bu. Chiralpak® IC-3 150 x 4.6 mm ID Analytical column with a column temperature of 40 °C, flow rate 2.75 mL/min, 10% MeOH in CO_2, isocratic.

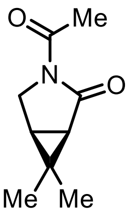

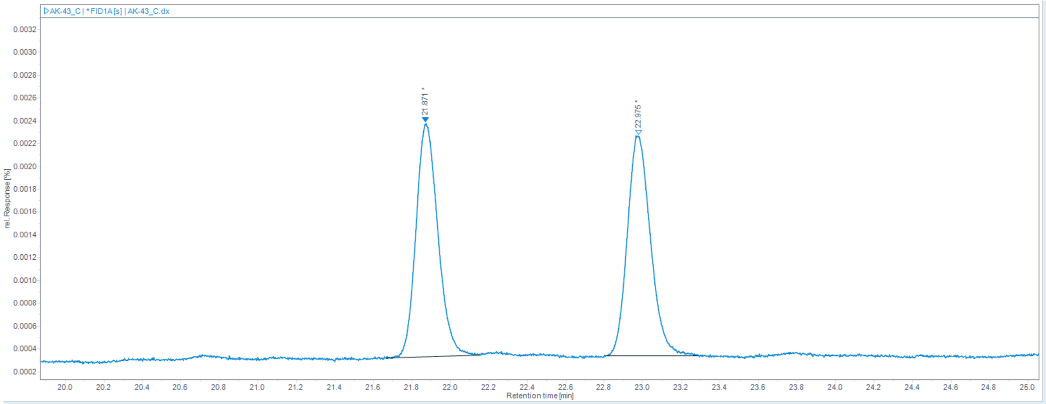

Injection Results

Peaks | Summary

#	Name	Signal description	RT (min)	Area (pA·s)	Area%	Height (pA)	Height%	Amount	Concentration	Start time (min)	End time (min)
1		* FID1A [s]	21.871	8.886	49.961	1.126	51.48			21.664	22.156
2		* FID1A [s]	22.975	8.900	50.039	1.061	48.52			22.804	23.285

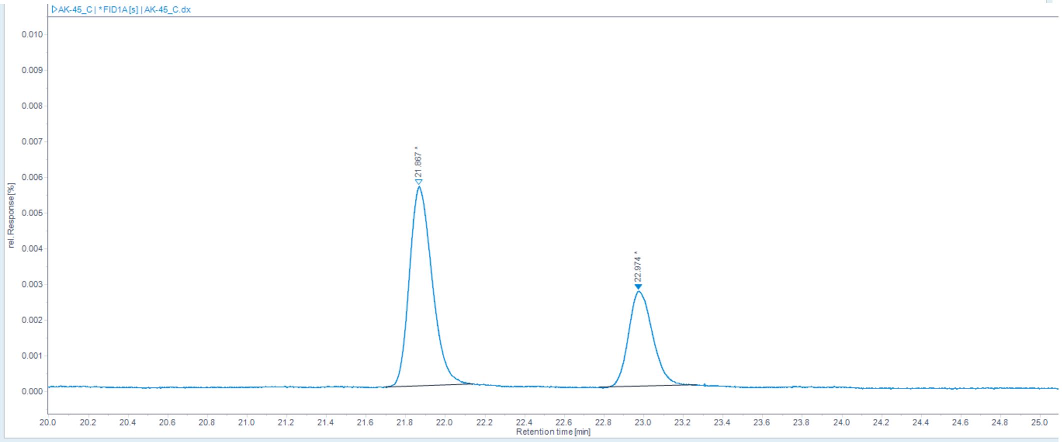

Injection Results

Peaks | Summary

#	Name	Signal description	RT (min)	Area (pA·s)	Area%	Height (pA)	Height%	Amount	Concentration	Start time (min)	End time (min)
1		* FID1A [s]	21.867	17.689	66.442	2.230	67.70			21.695	22.139
2		* FID1A [s]	22.974	8.934	33.558	1.064	32.30			22.775	23.267

GC charts of racemic (top) and enantioenriched (bottom) compound **2-Me**. J&W CP-ChiraSil-DEX CB GC column, 50 m x 0.25 mm x 0.25 µm, 1.6 mL/min He, 130 °C isocratic.

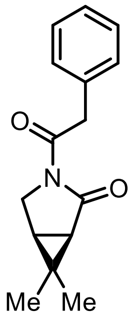

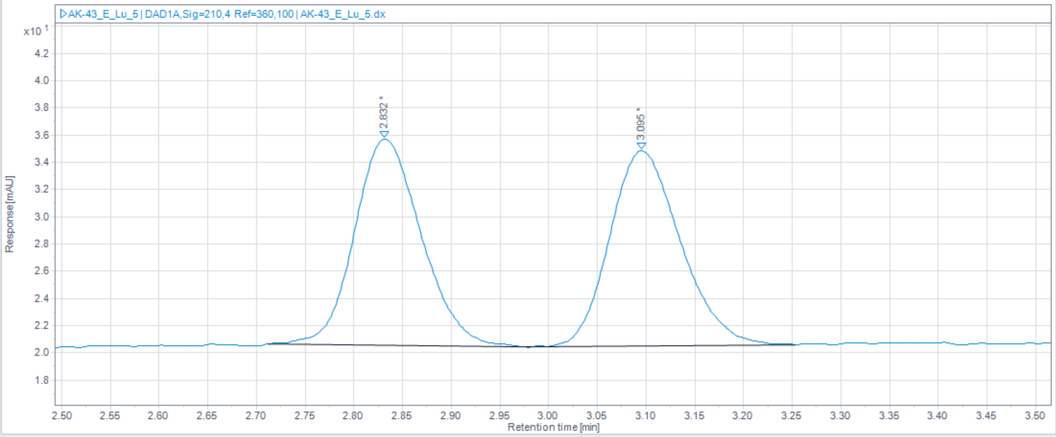

Injection Results

Peaks | Summary

#	Signal description	RT (min)	Area (mAU·s)	Area%	Height (mAU)	Height%	Amount	Concentration	Start time (min)	End time (min)
1	DAD1A,Sig=210,4 Ref=360,100	2.832	72.317	49.984	15.148	51.36			2.712	2.975
2	DAD1A,Sig=210,4 Ref=360,100	3.095	72.364	50.016	14.346	48.64			2.975	3.252

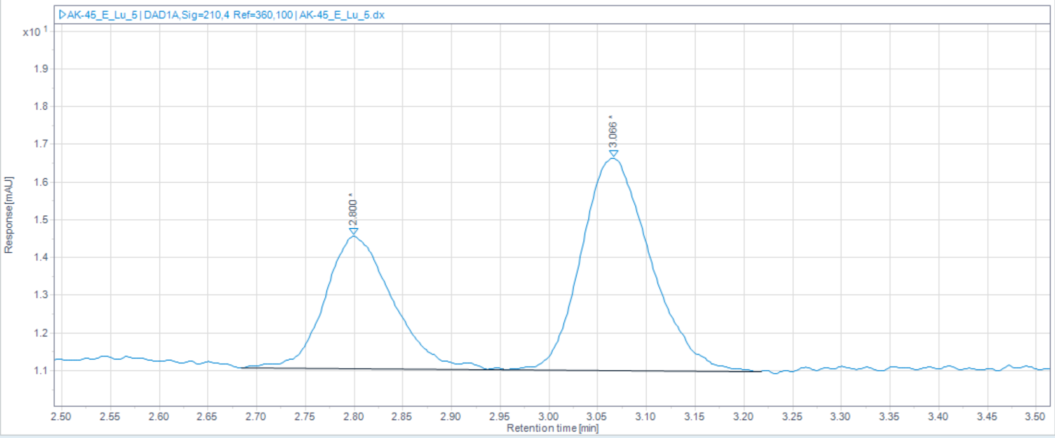

Injection Results

Peaks | Summary

#	Signal description	RT (min)	Area (mAU·s)	Area%	Height (mAU)	Height%	Amount	Concentration	Start time (min)	End time (min)
1	DAD1A,Sig=210,4 Ref=360,100	2.800	16.971	38.063	3.498	38.33			2.684	2.953
2	DAD1A,Sig=210,4 Ref=360,100	3.066	27.615	61.937	5.627	61.67			2.953	3.217

SFC charts of racemic (top) and enantioenriched (bottom) compound **2-Bn**. Chiralpak® IC-3 150 x 4.6 mm ID Analytical column with a column temperature of 40 °C, flow rate 2.75 mL/min, 5% MeOH in CO_2, isocratic.

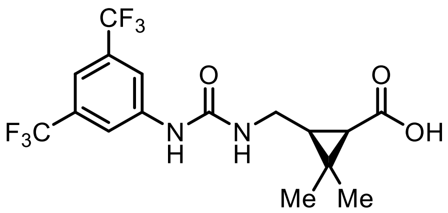

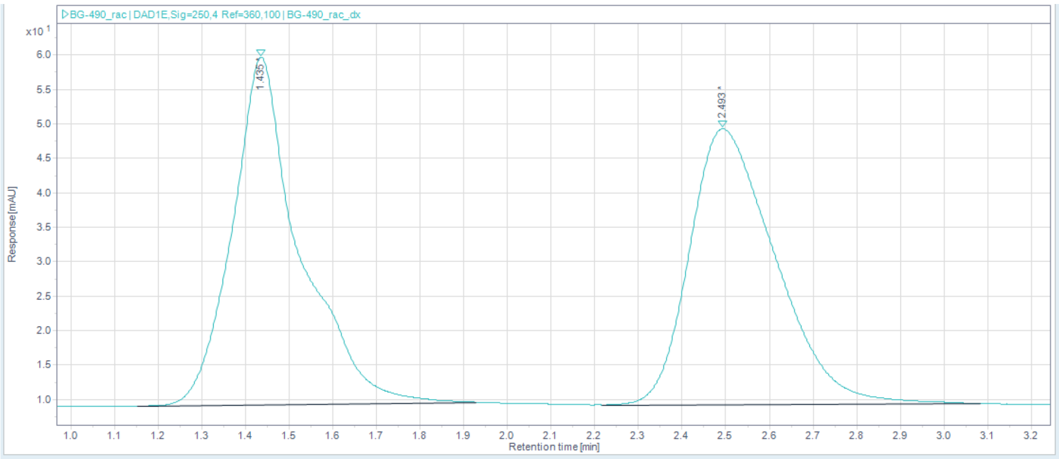

Injection Results

Peaks Summary

#	Signal description	RT (min)	Area (mAU·s)	Area%	Height (mAU)	Height%	Amount	Concentration	Start time (min)	End time (min)
1	DAD1E,Sig=250,4 Ref=360,100	1.435	544.130	49.941	50.454	55.77			1.151	1.929
2	DAD1E,Sig=250,4 Ref=360,100	2.493	545.416	50.059	40.016	44.23			2.215	3.082

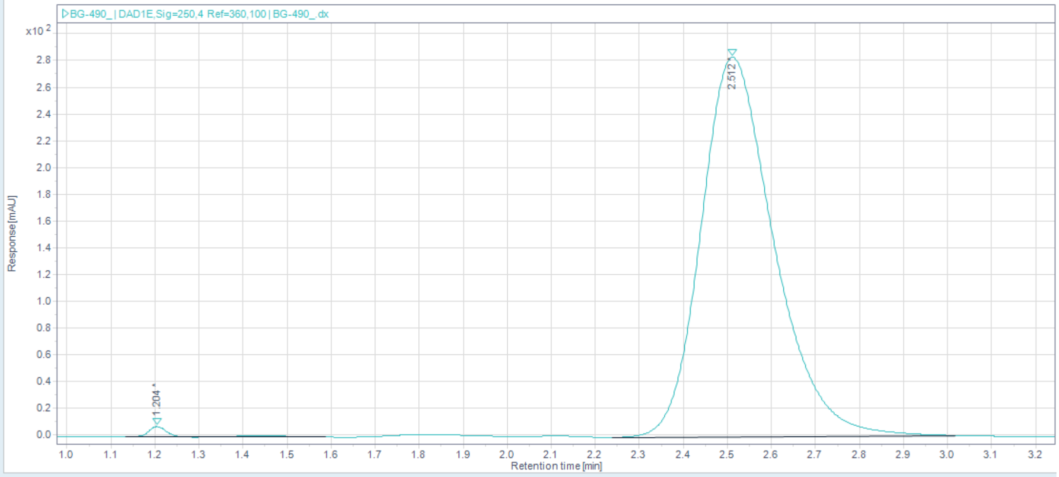

Injection Results

Peaks Summary

#	Signal description	RT (min)	Area (mAU·s)	Area%	Height (mAU)	Height%	Amount	Concentration	Start time (min)	End time (min)
1	DAD1E,Sig=250,4 Ref=360,100	1.204	30.822	0.935	7.810	2.68			1.132	1.586
2	DAD1E,Sig=250,4 Ref=360,100	2.512	3264.006	99.065	283.875	97.32			2.237	3.018

SFC charts of racemic (top) and enantioenriched (bottom) compound **23**. Chiralpak® IB N-3 150 x 4.6 mm ID Analytical column with a column temperature of 40 °C, flow rate 2.75 mL/min, 1% MeOH in CO_2, isocratic.

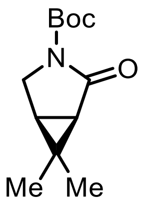

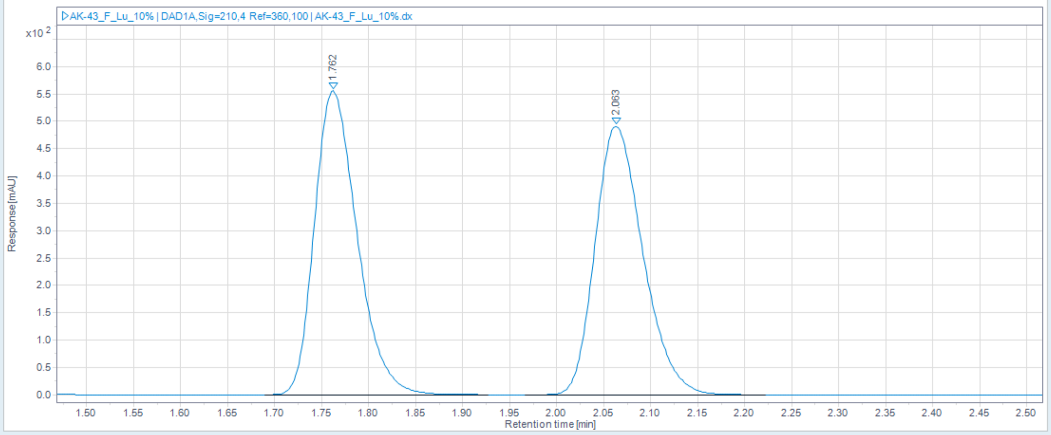

Injection Results

Peaks Summary

#	Signal description	RT (min)	Area (mAU·s)	Area%	Height (mAU)	Height%	Amount	Concentration	Start time (min)	End time (min)
1	DAD1A,Sig=210,4 Ref=360,100	1.762	1754.651	50.233	555.941	53.08			1.689	1.927
2	DAD1A,Sig=210,4 Ref=360,100	2.063	1738.344	49.767	491.338	46.92			1.967	2.222

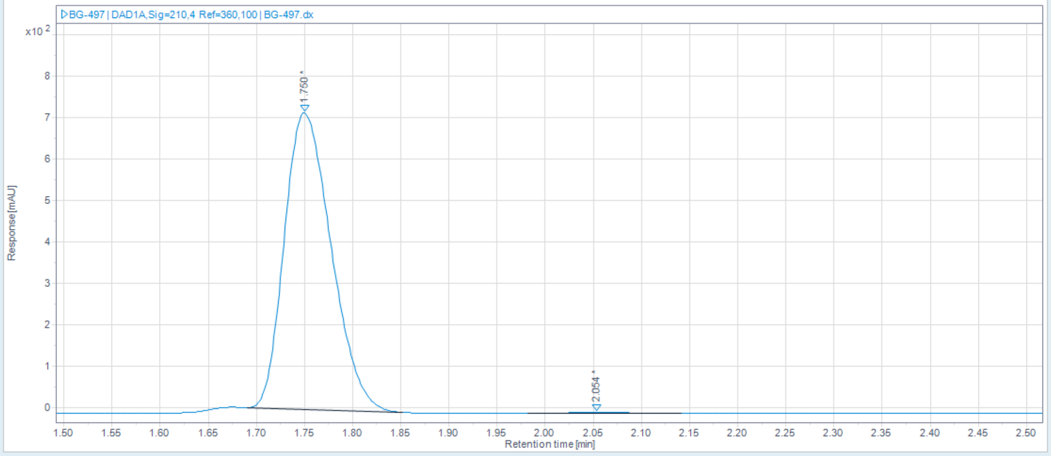

Injection Results

Peaks Summary

#	Signal description	RT (min)	Area (mAU·s)	Area%	Height (mAU)	Height%	Amount	Concentration	Start time (min)	End time (min)
2	DAD1A,Sig=210,4 Ref=360,100	2.054	10.617	0.436	2.792	0.39			1.981	2.141
1	DAD1A,Sig=210,4 Ref=360,100	1.750	2424.706	99.564	715.922	99.61			1.690	1.852

SFC charts of racemic (top) and enantioenriched (bottom) compound **24** from derivatization of crystallized **5**. Chiralpak® IC-3 150 x 4.6 mm ID Analytical column with a column temperature of 40 °C, flow rate 2.75 mL/min, 10% MeOH in CO_2, isocratic.

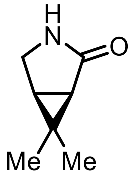

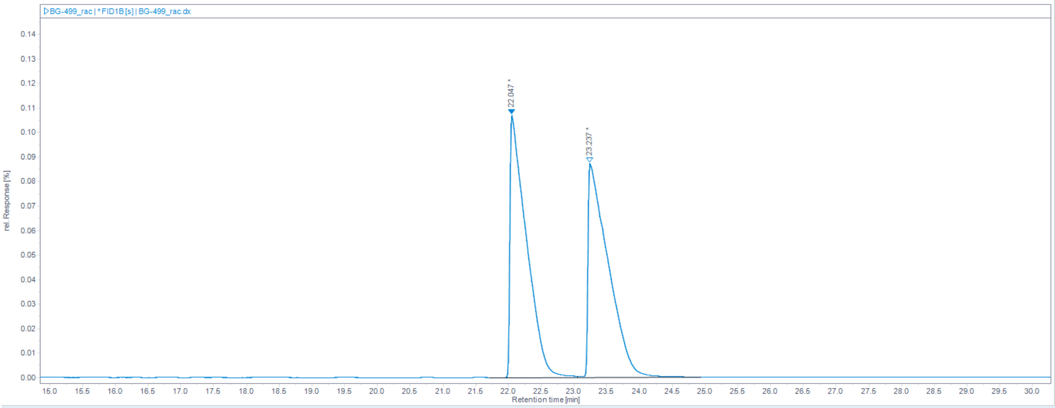

Injection Results

Peaks Summary

#	Name	Signal description	RT (min)	Area (pA·s)	Area%	Height (pA)	Height%	Amount	Concentration	Start time (min)	End time (min)
1		* FID1B [s]	22.047	1173.190	49.943	67.474	55.09			21.713	23.053
2		* FID1B [s]	23.237	1175.846	50.057	55.007	44.91			23.053	24.941

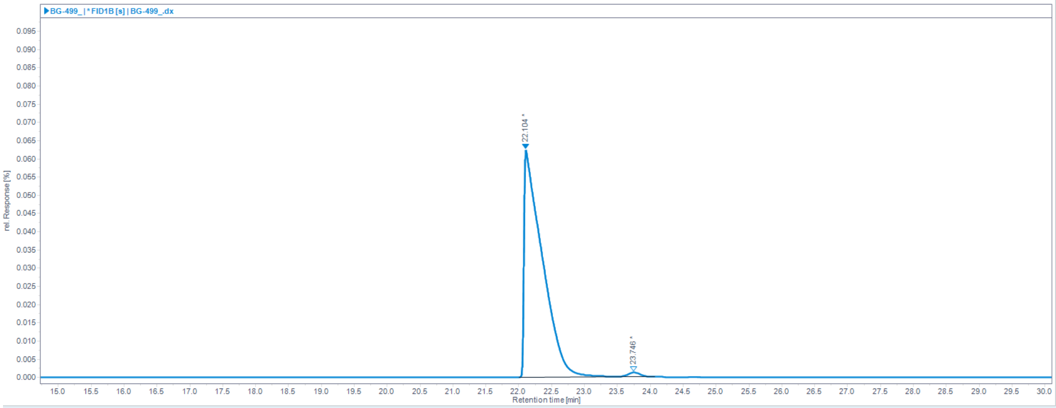

Injection Results

Peaks Summary

#	Name	Signal description	RT (min)	Area (pA·s)	Area%	Height (pA)	Height%	Amount	Concentration	Start time (min)	End time (min)
1		* FID1B [s]	22.104	1345.376	98.880	70.311	98.26			22.001	23.558
2		* FID1B [s]	23.746	15.245	1.120	1.246	1.74			23.558	24.068

GC charts of racemic (top) and enantioenriched (bottom) compound **23**. CycloSil-B 30 m x 250 µm x 0.25 µm, 1.6 mL/min He, 140 °C isocratic.

9. Crystal Structure

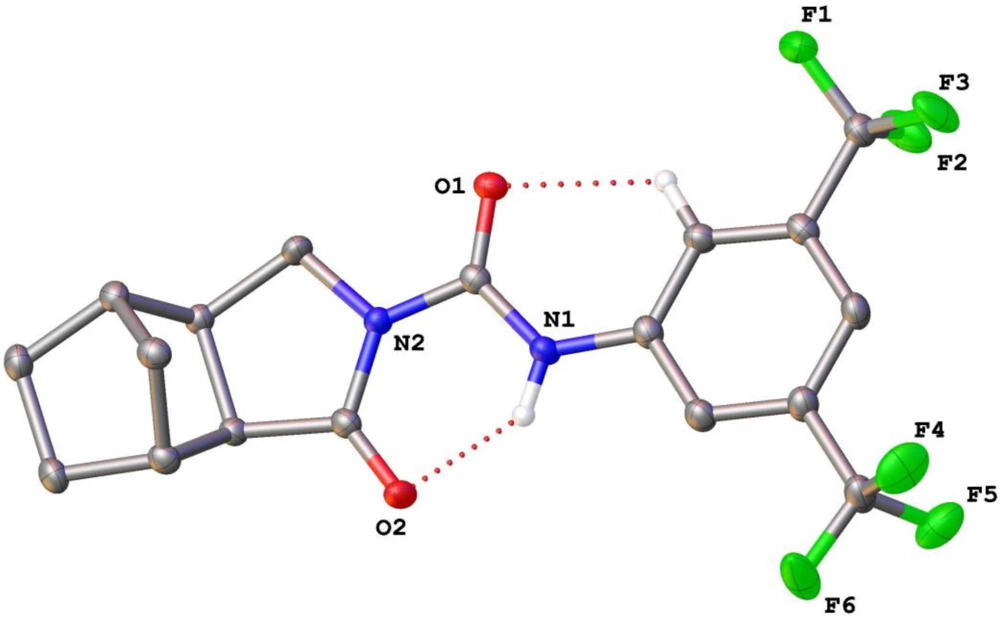

Figure S9. The complete numbering scheme of **15** (007a-21097) with 50% thermal ellipsoid probability levels. The hydrogen atoms are omitted for clarity.

Table S6. Crystal data and structure refinement for **15**.

Identification code	rbg1_abs	
Empirical formula	C18 H16 F6 N2 O2	
Formula weight	406.33	
Temperature	100.00(11) K	
Wavelength	1.54184 Å	
Crystal system	Monoclinic	
Space group	P 1 21 1	
Unit cell dimensions	a = 8.35780(10) Å	a= 90°.
	b = 8.13560(10) Å	b= 91.4190(10)°.
	c = 12.41720(10) Å	g = 90°.
Volume	844.058(16) $Å^3$	
Z	2	
Density (calculated)	1.599 Mg/m^3	
Absorption coefficient	1.320 mm^{-1}	
F(000)	416	
Crystal size	0.276 x 0.187 x 0.111 mm^3	
Theta range for data collection	3.561 to 79.869°.	
Index ranges	-10<=h<=10, -10<=k<=10, -11<=l<=15	
Reflections collected	18536	
Independent reflections	3631 [R(int) = 0.0438]	

Completeness to theta = 67.684°	100.0 %
Absorption correction	Gaussian
Max. and min. transmission	1.000 and 0.349
Refinement method	Full-matrix least-squares on F^2
Data / restraints / parameters	3631 / 2 / 257
Goodness-of-fit on F^2	1.035
Final R indices [I>2sigma(I)]	R1 = 0.0308, wR2 = 0.0760
R indices (all data)	R1 = 0.0326, wR2 = 0.0780
Absolute structure parameter	-0.05(5)
Extinction coefficient	0.0039(6)
Largest diff. peak and hole	0.281 and -0.271 e.Å^{-3}

10. NMR Spectra

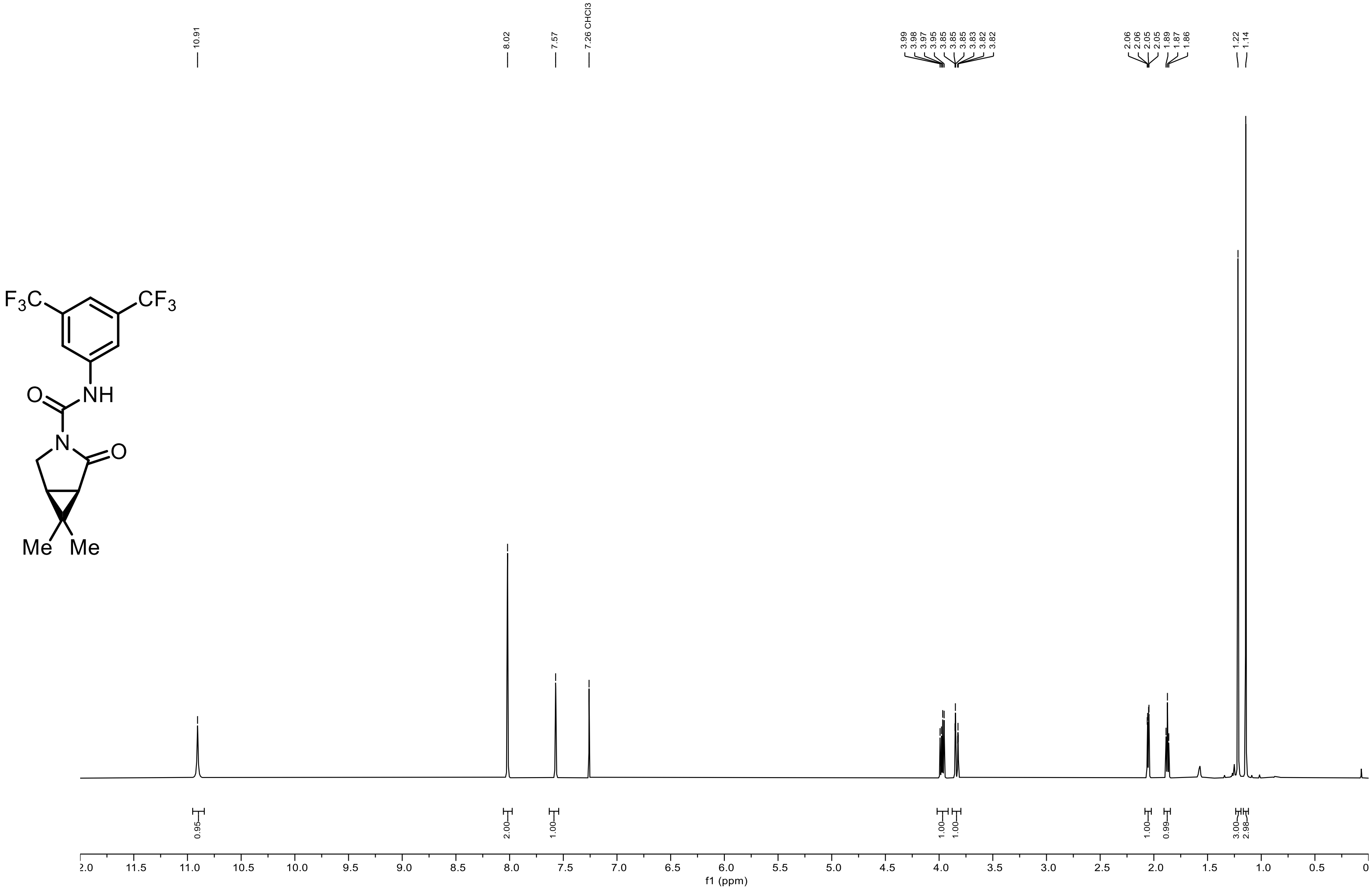

5 ^{1}H NMR (500 MHz, $CDCl_3$).

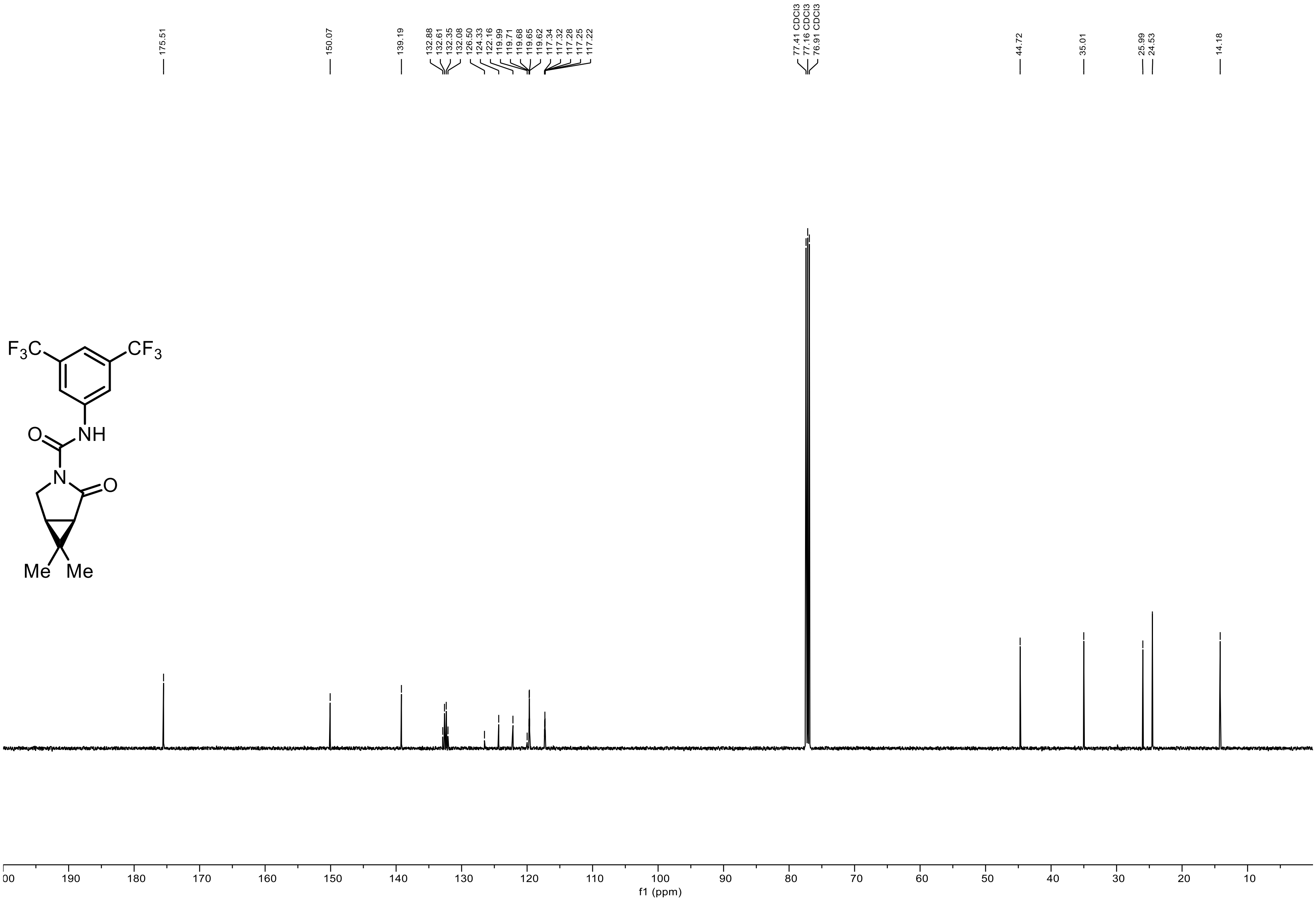

5 ^{13}C NMR (126 MHz, $CDCl_3$).

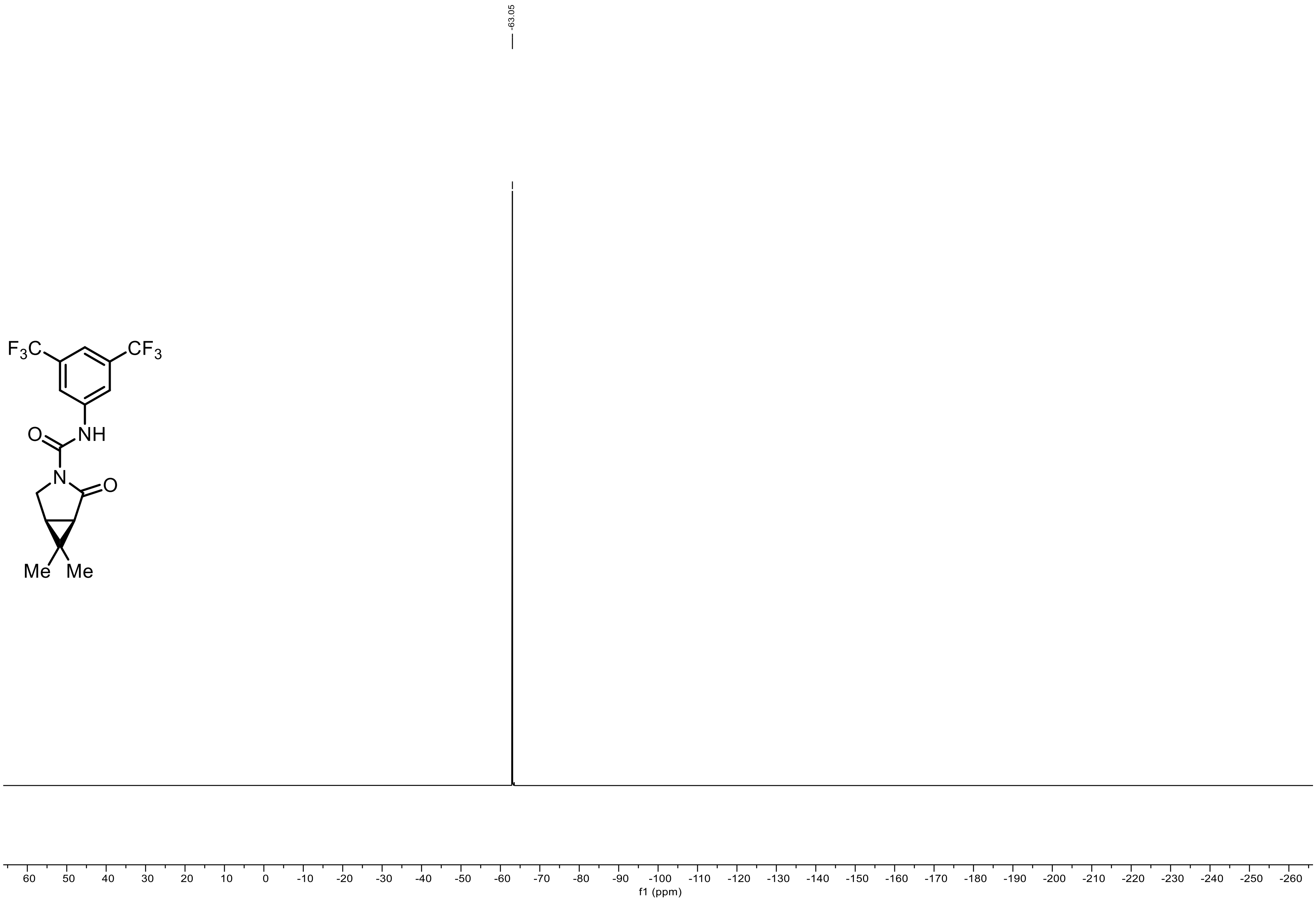

5 ^{19}F NMR (470 MHz, $CDCl_3$).

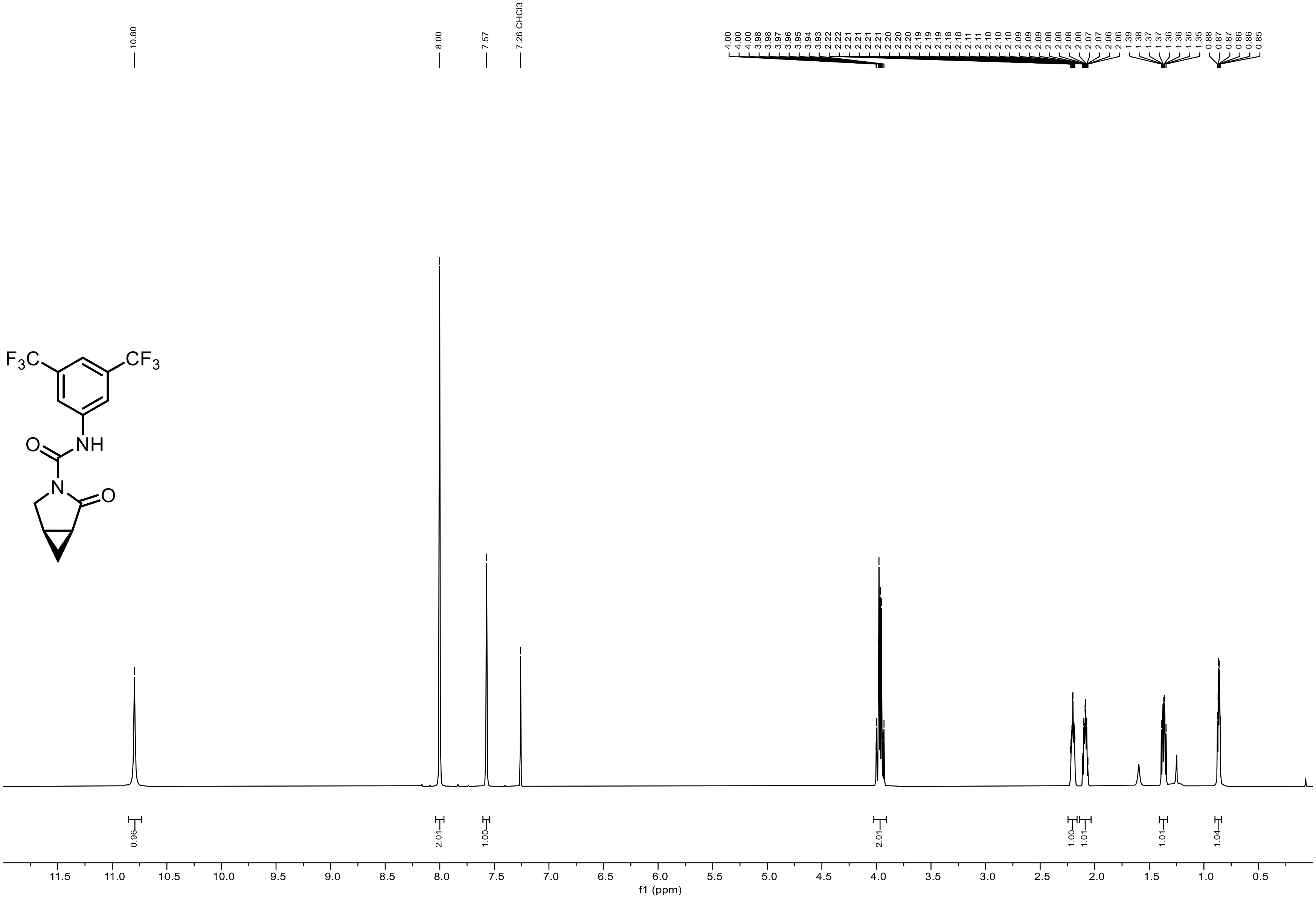

7 ^{1}H NMR (500 MHz, $CDCl_3$).

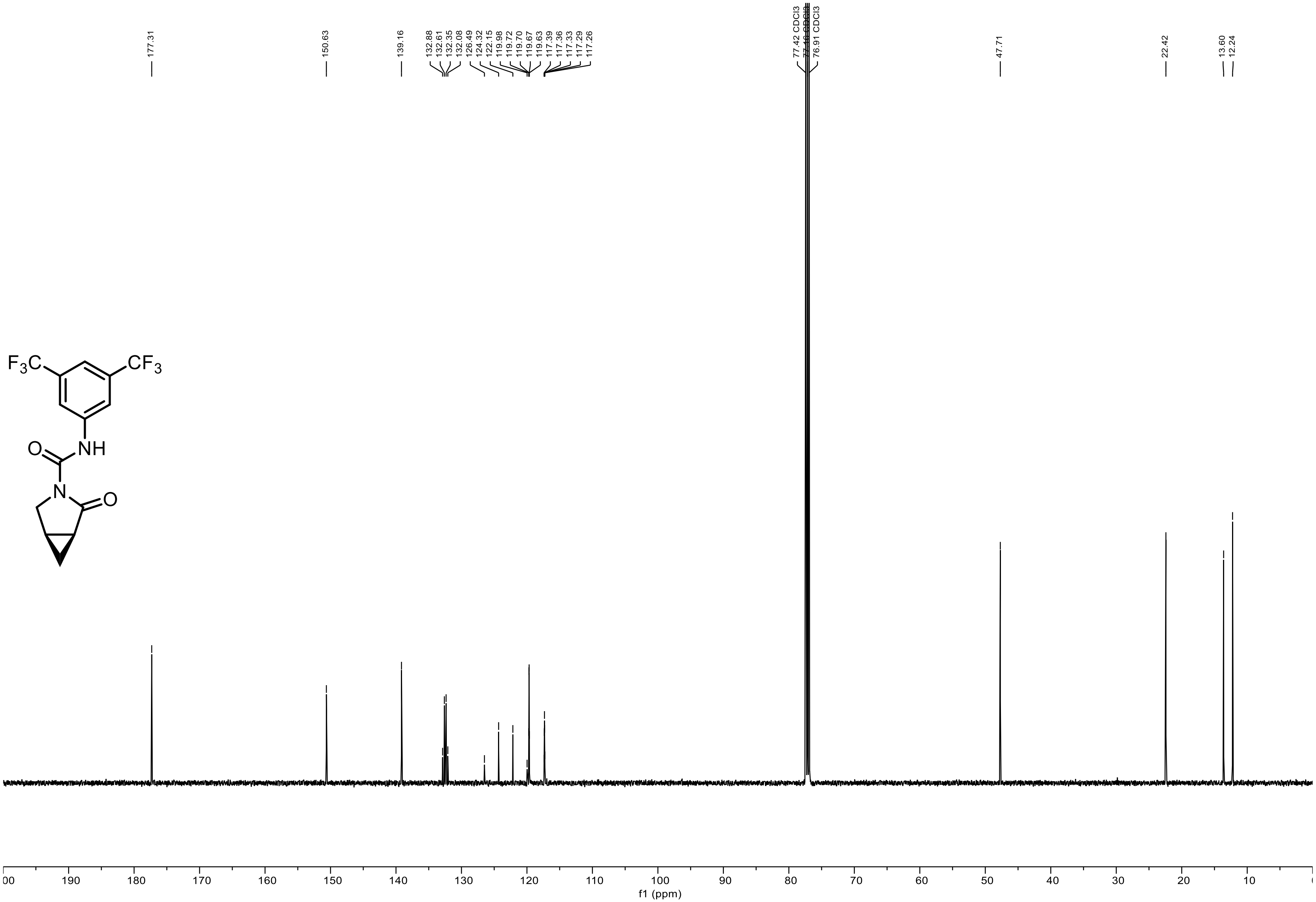

7 ^{13}C NMR (126 MHz, $CDCl_3$).

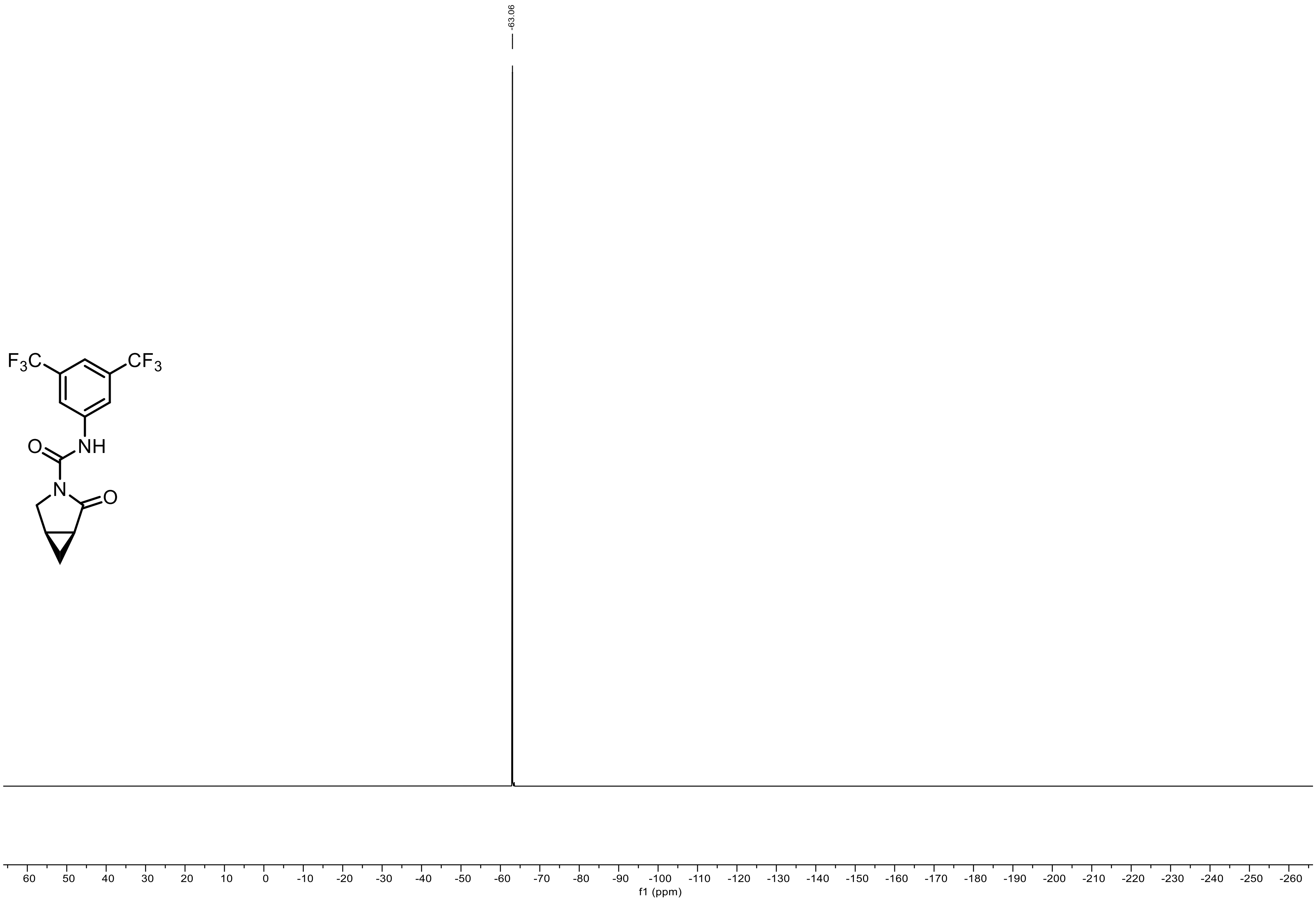

7 ^{19}F NMR (470 MHz, $CDCl_3$).

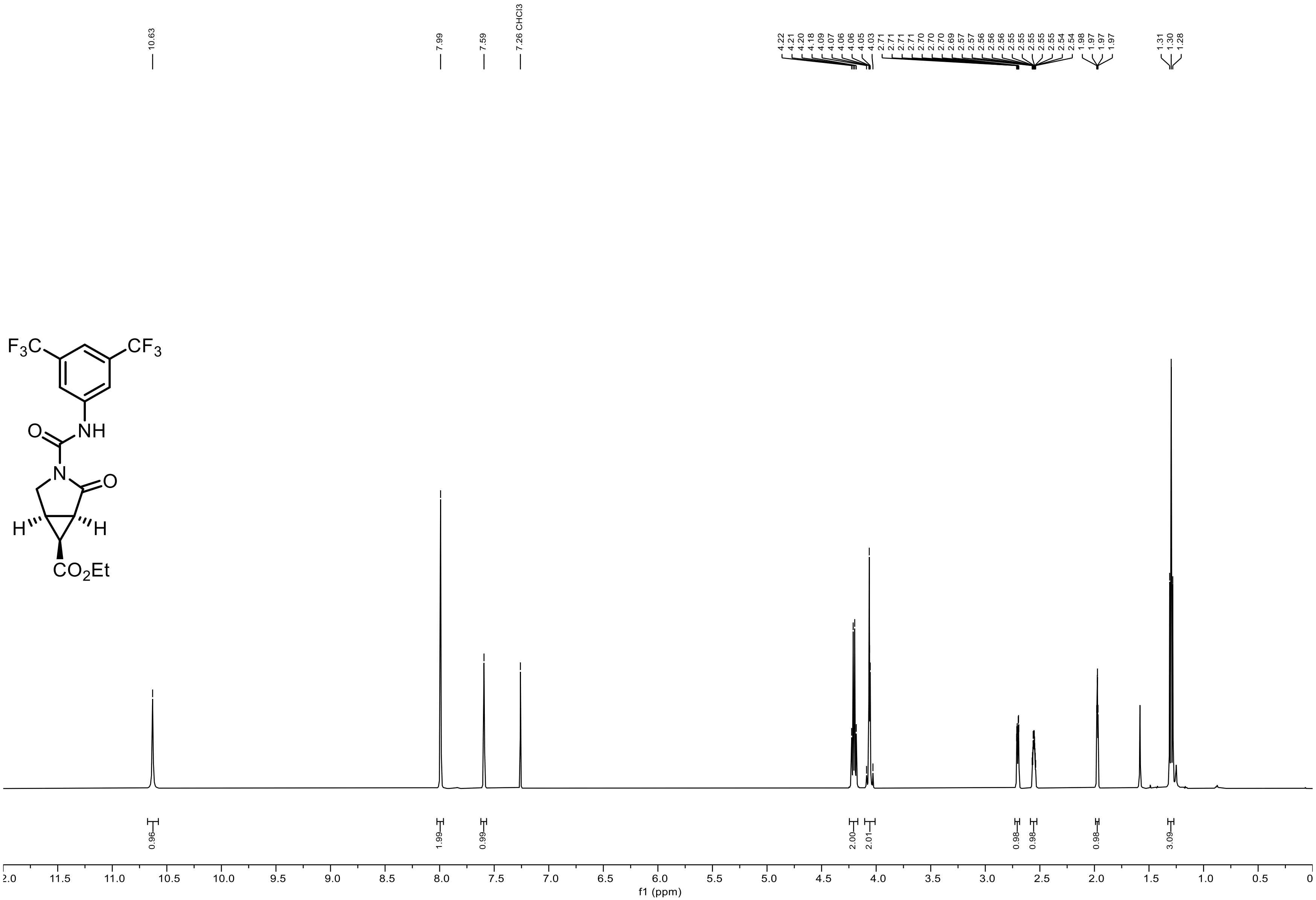

8 ^{1}H NMR (500 MHz, $CDCl_3$).

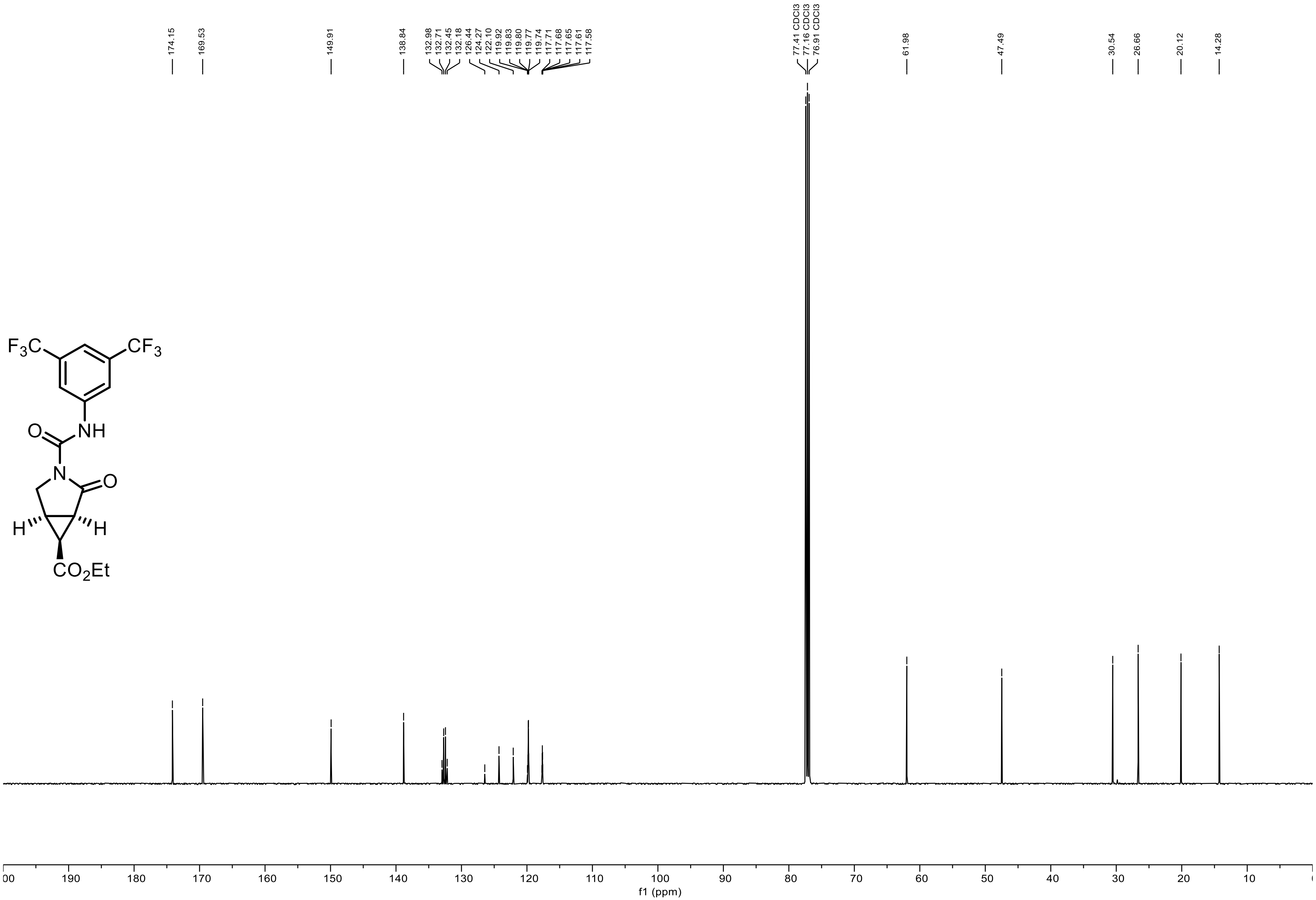

8 ^{13}C NMR (126 MHz, $CDCl_3$).

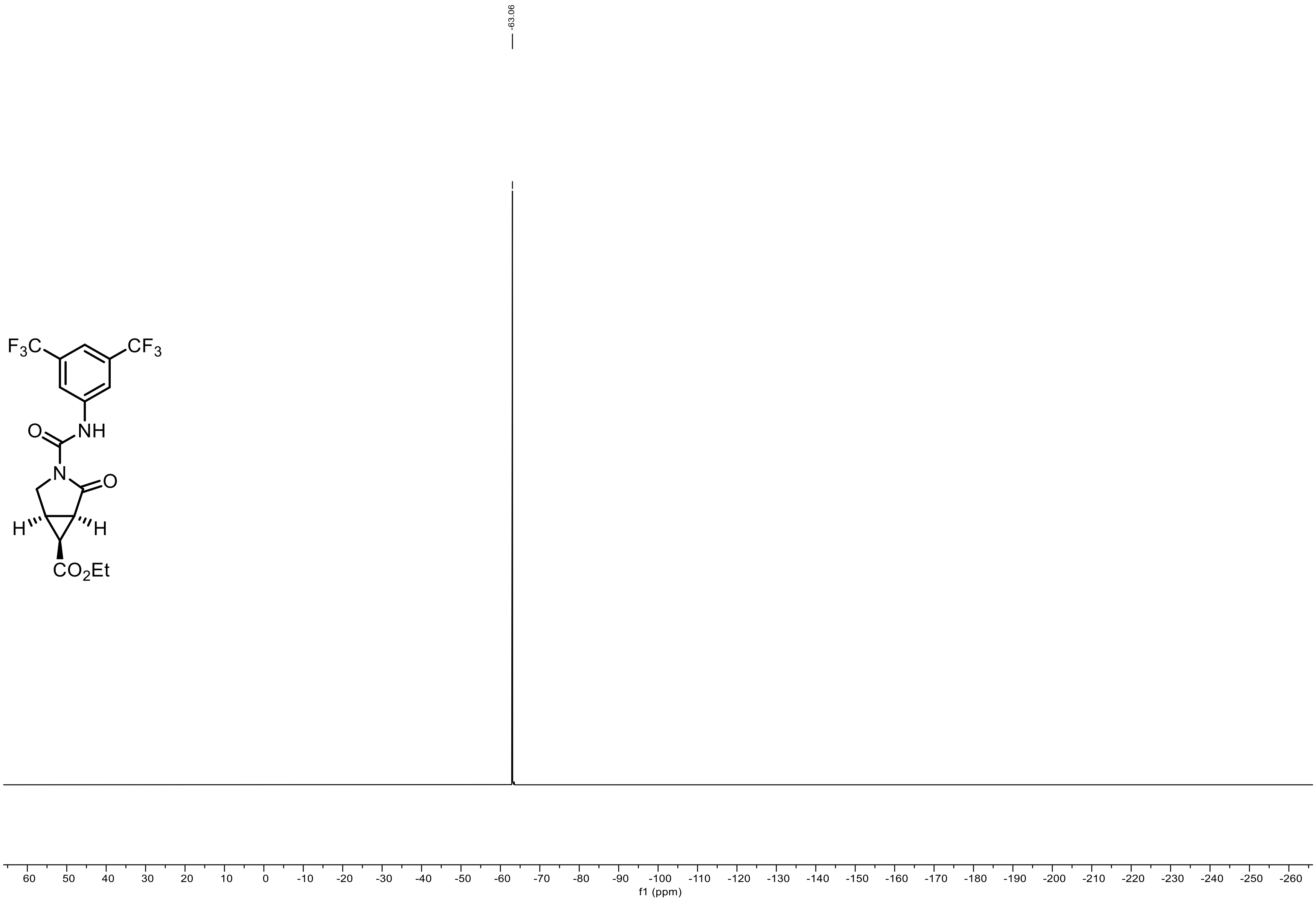

8 ^{19}F NMR (470 MHz, $CDCl_3$).

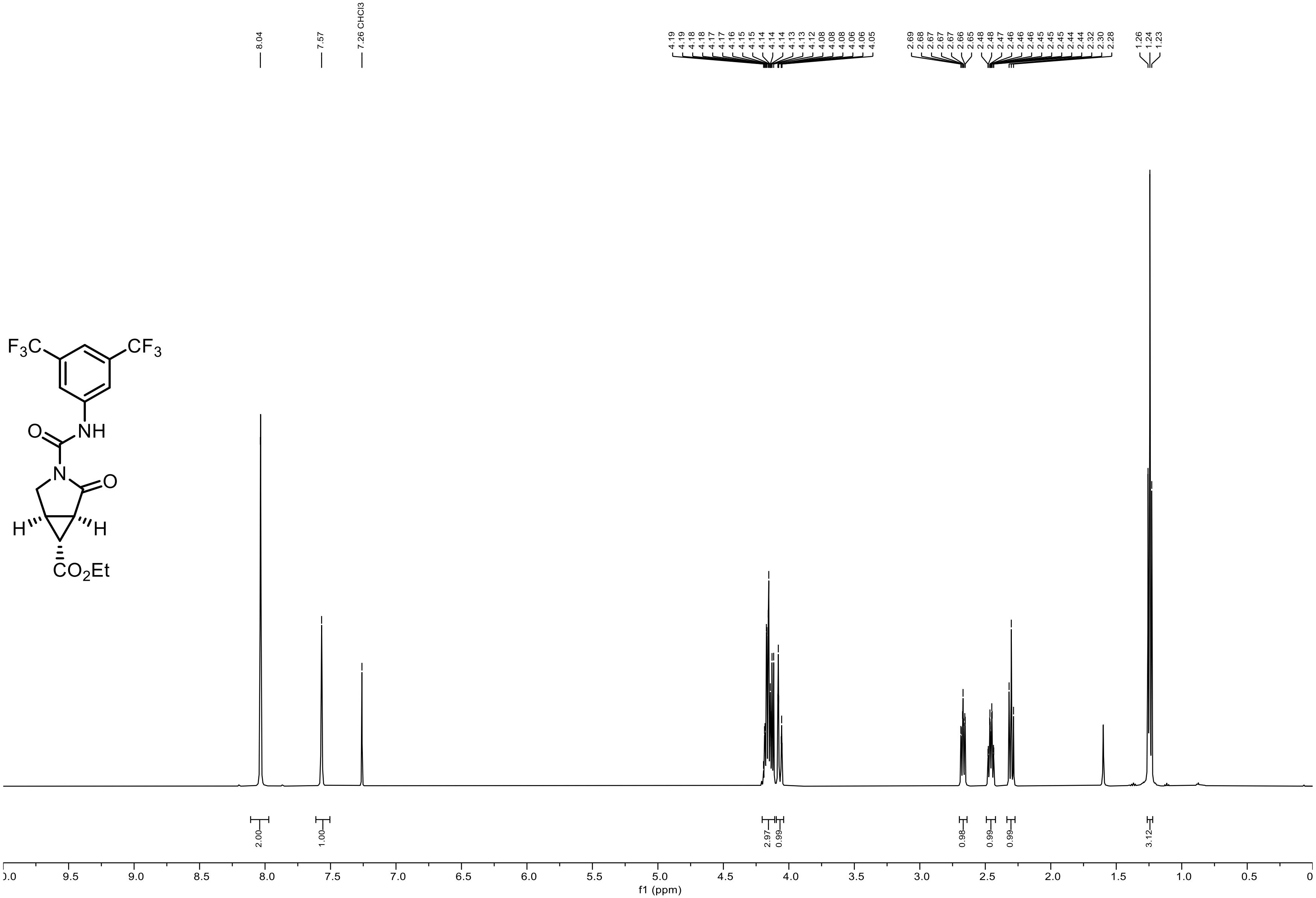

9 ^{1}H NMR (500 MHz, $CDCl_3$).

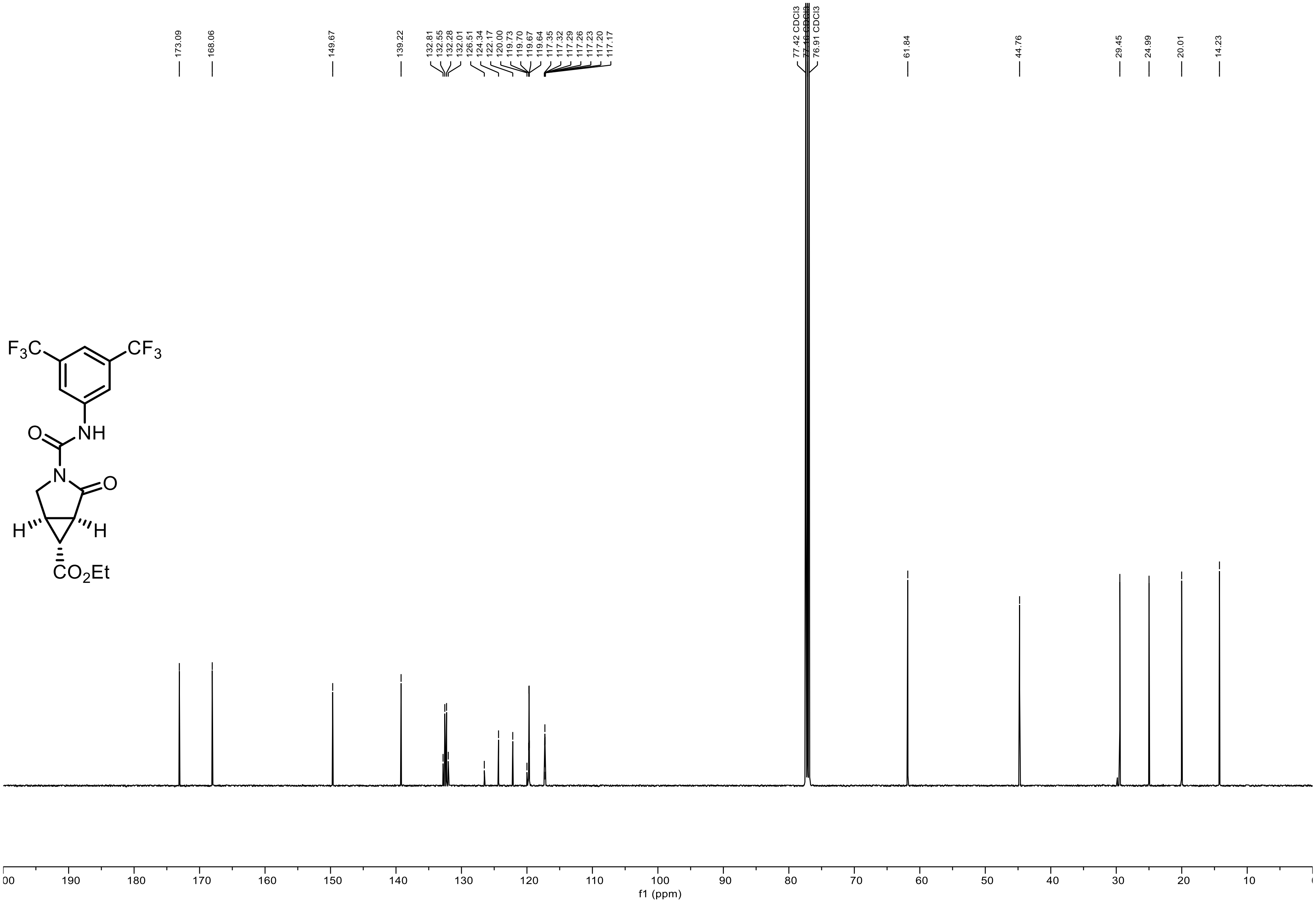

9 ^{13}C NMR (126 MHz, $CDCl_3$).

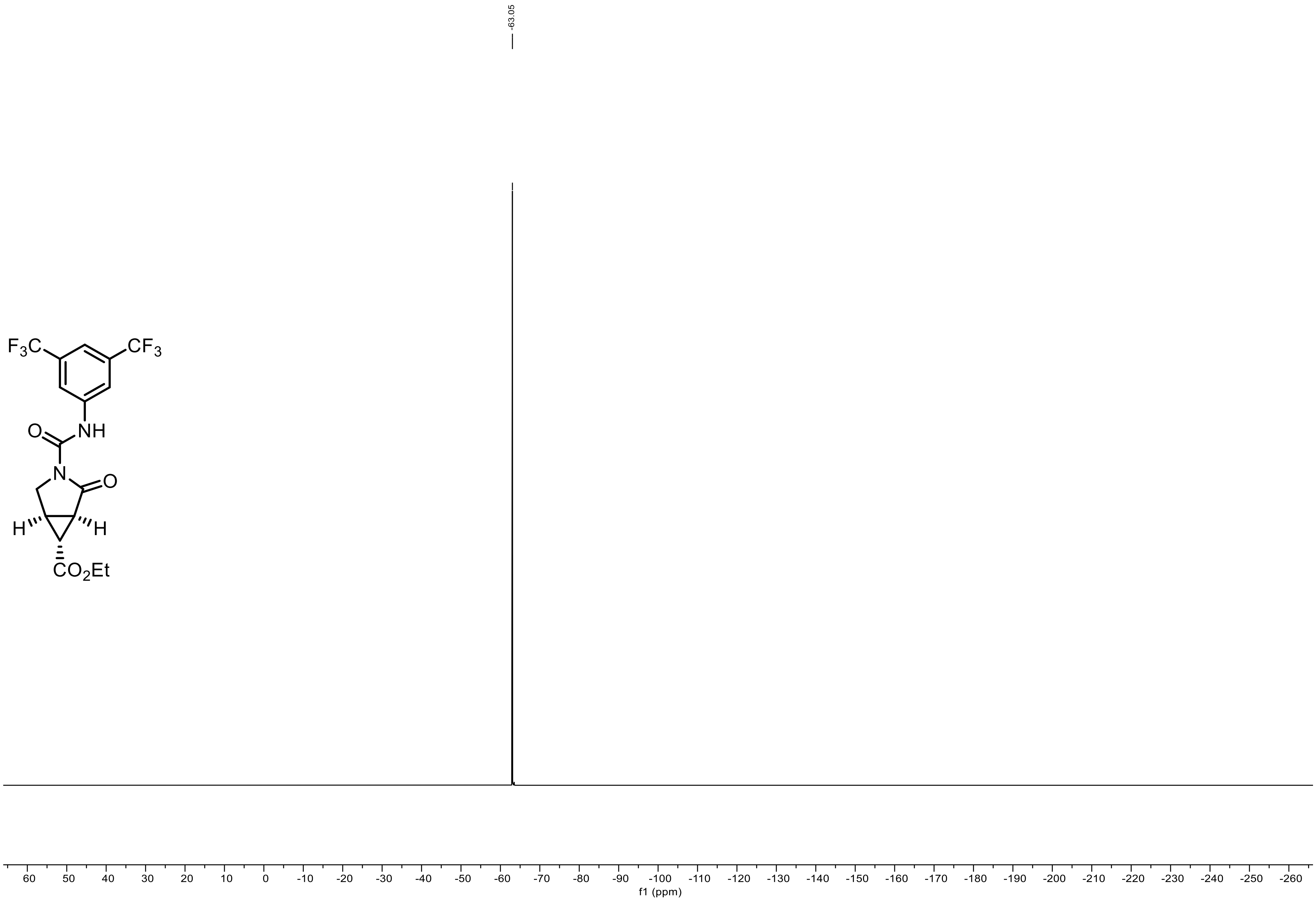

9 ^{19}F NMR (470 MHz, $CDCl_3$).

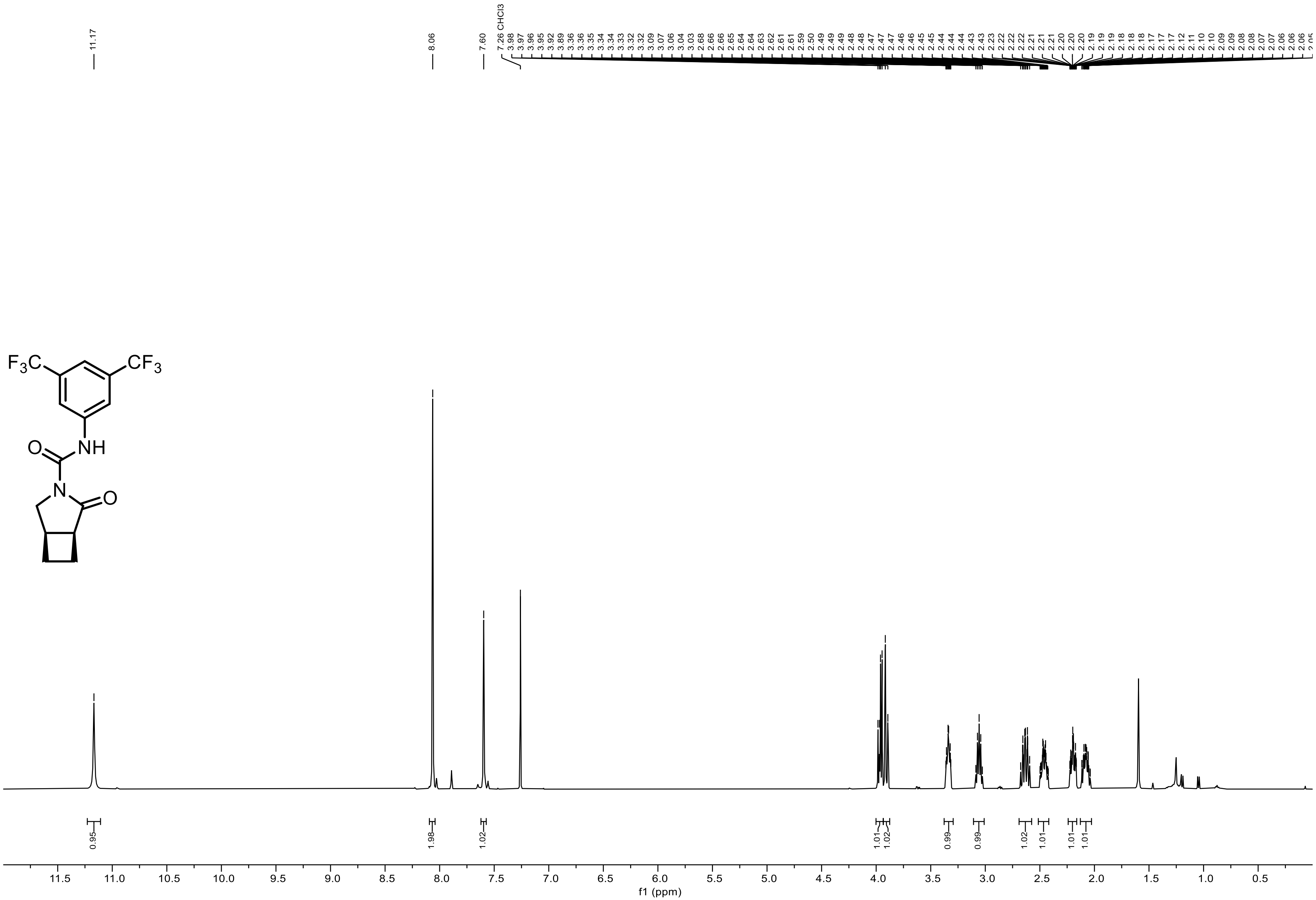

10 ^{1}H NMR (500 MHz, $CDCl_3$).

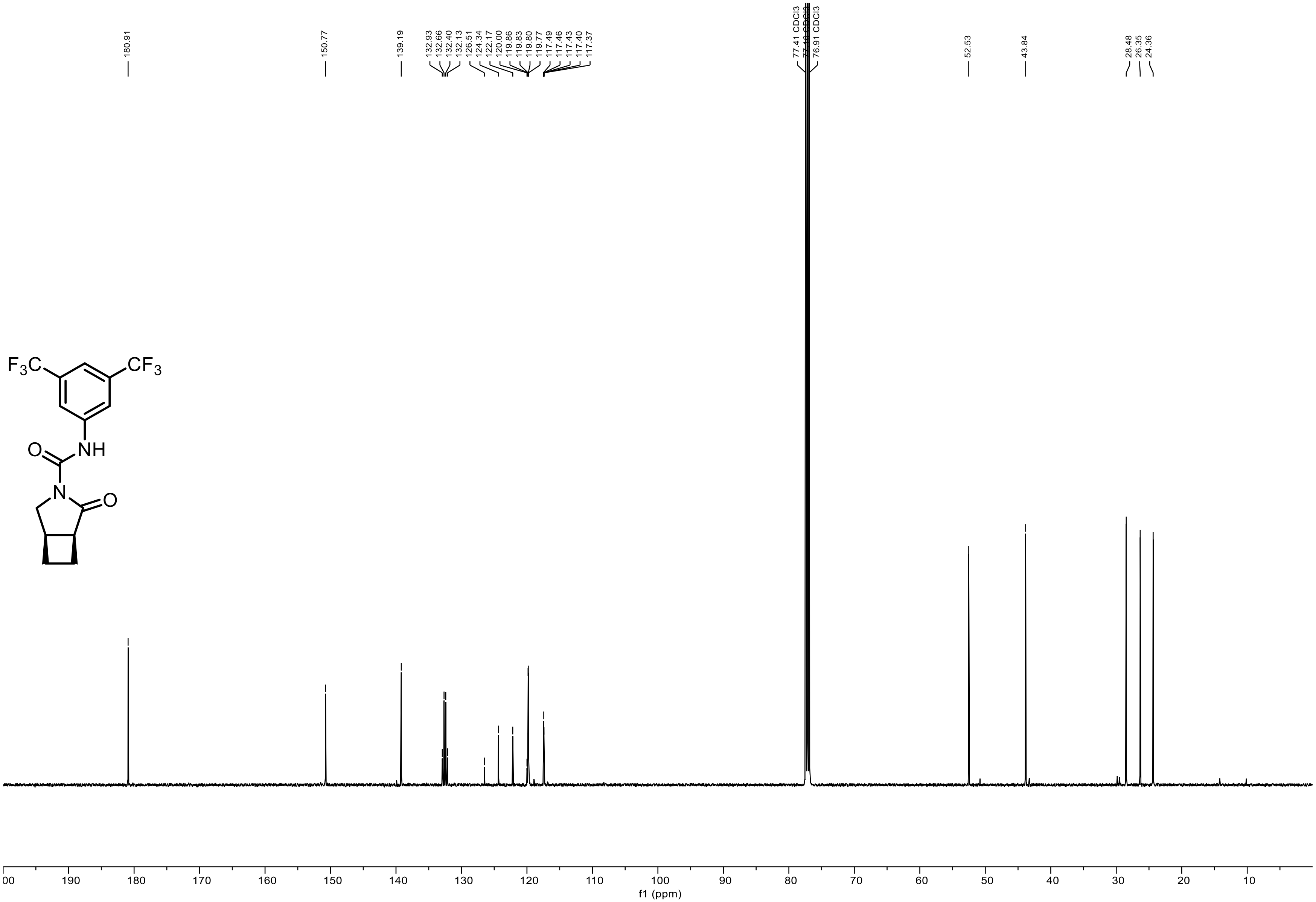

10 ^{13}C NMR (126 MHz, $CDCl_3$).

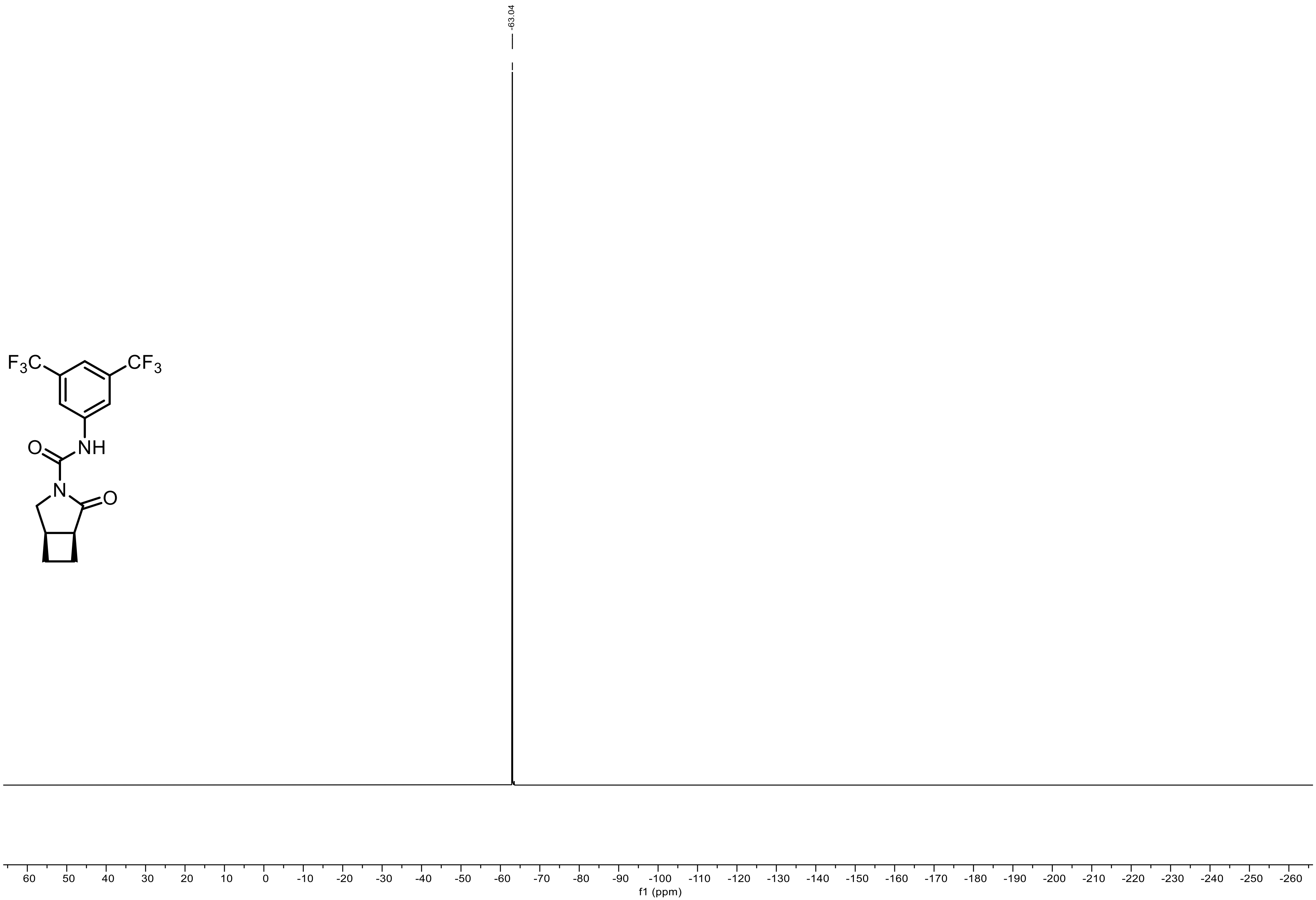

10 ^{19}F NMR (470 MHz, $CDCl_3$).

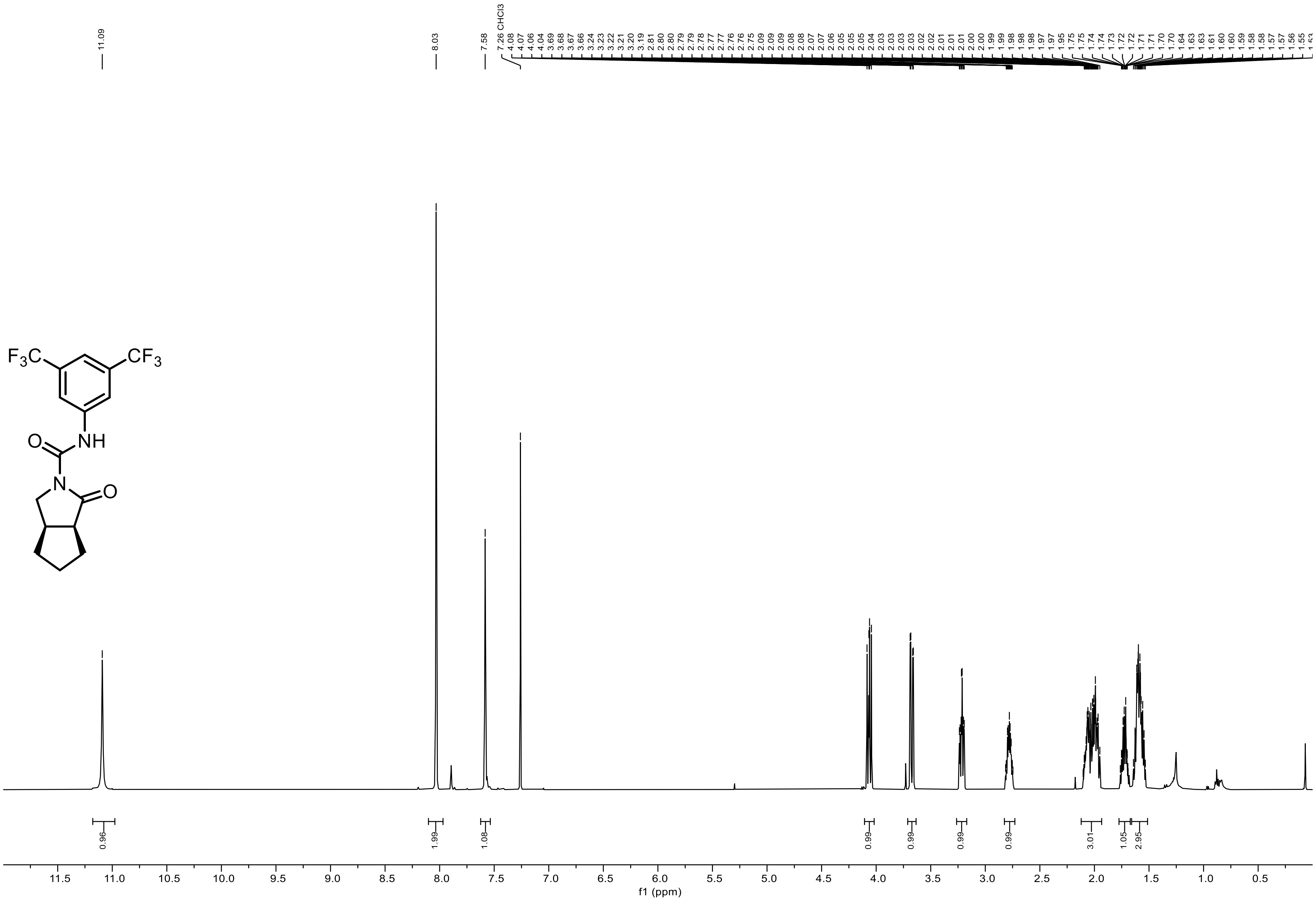

11 ^{1}H NMR (500 MHz, $CDCl_3$).

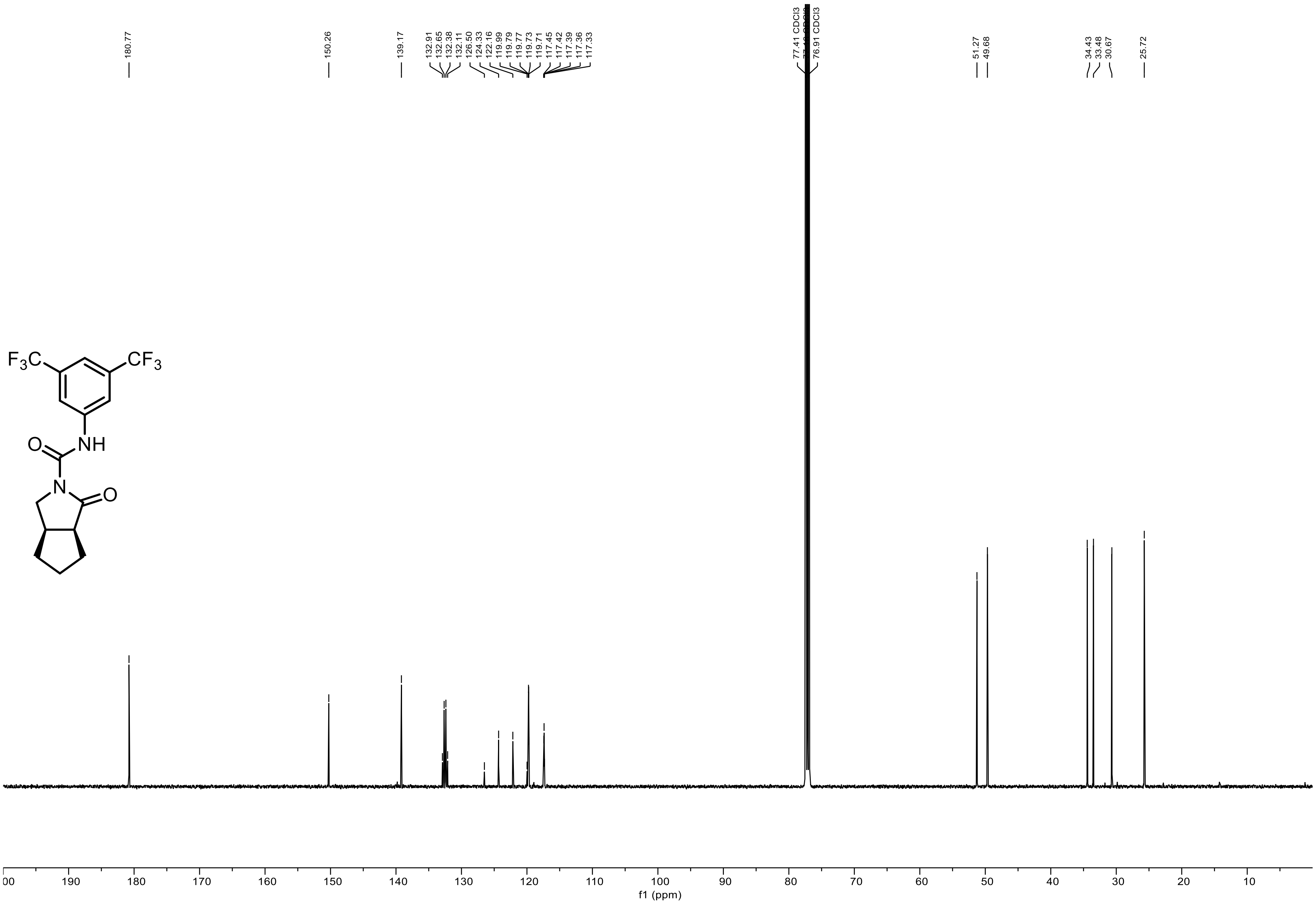

11 ^{13}C NMR (126 MHz, $CDCl_3$).

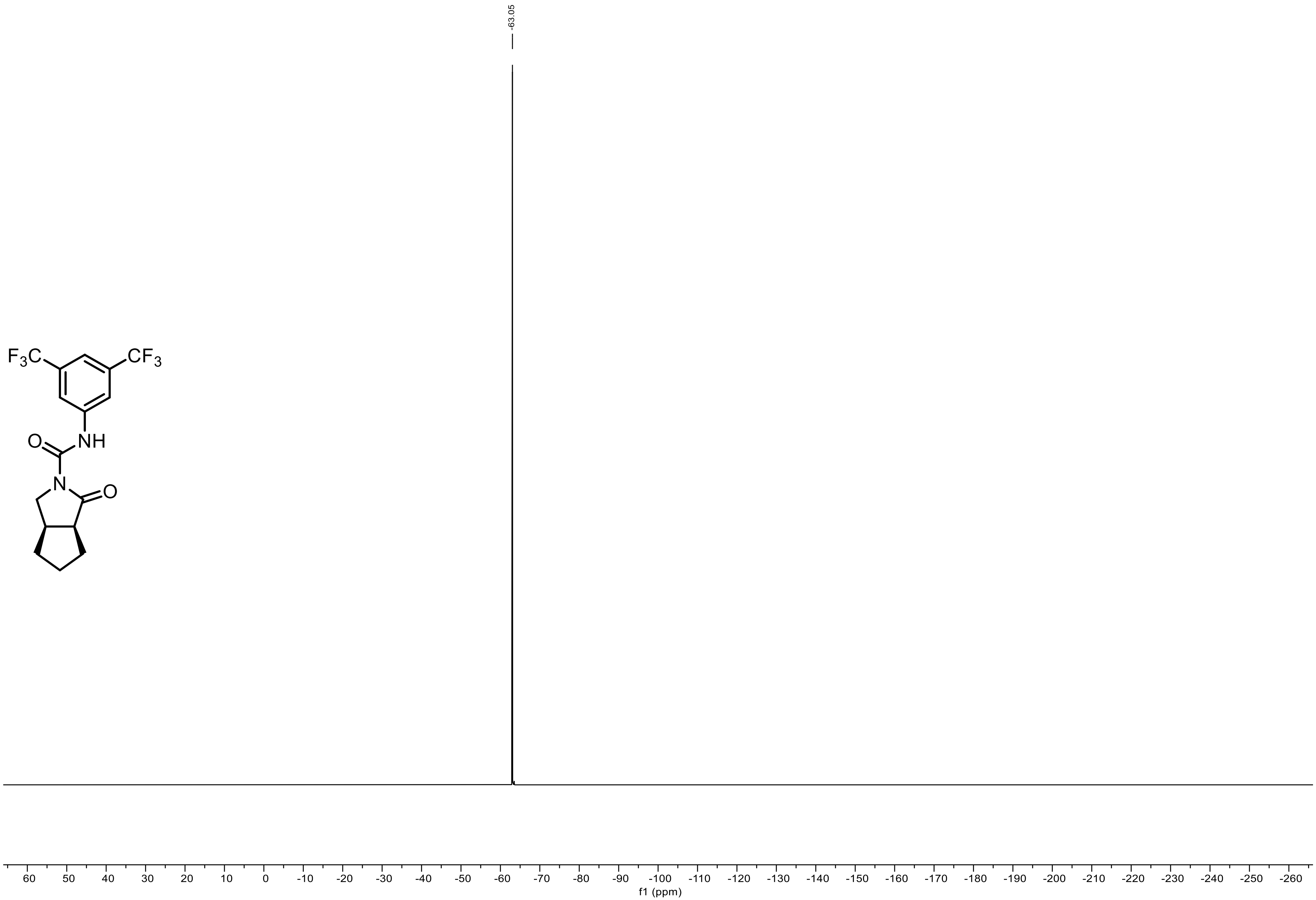

11 ^{19}F NMR (470 MHz, $CDCl_3$).

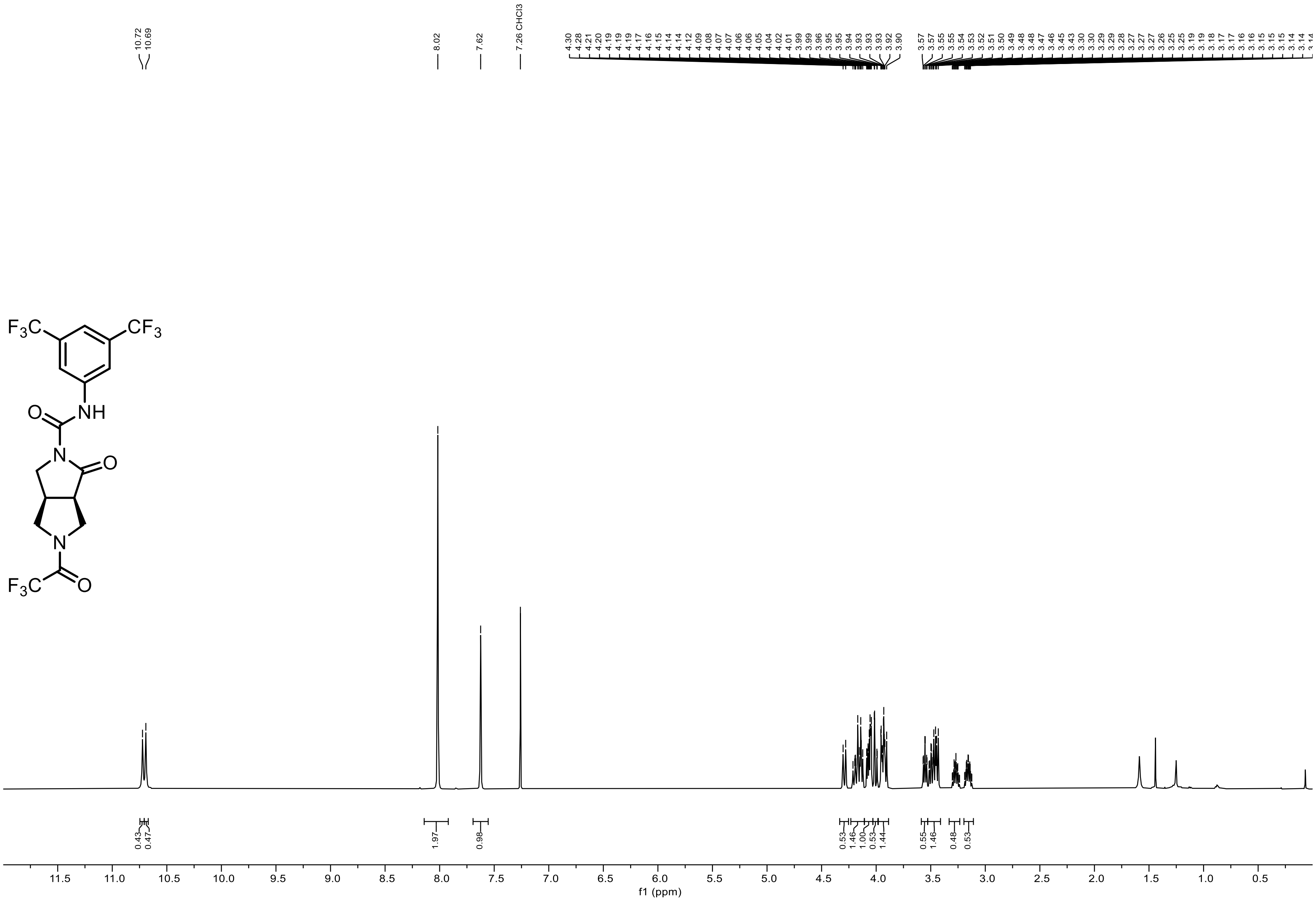

12 ^{1}H NMR (500 MHz, $CDCl_3$, mixture of rotamers).

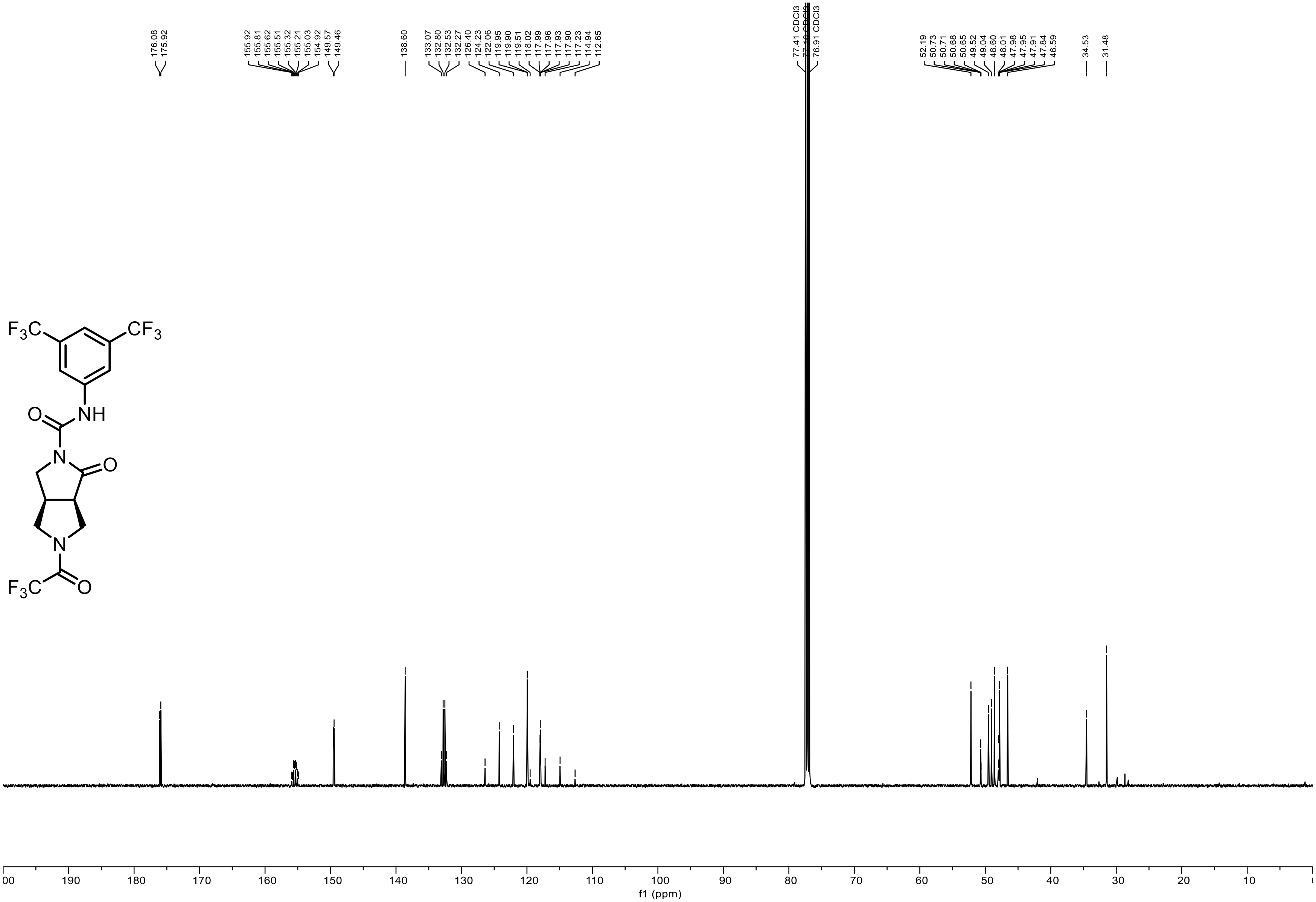

12 ^{13}C NMR (126 MHz, $CDCl_3$, mixture of rotamers).

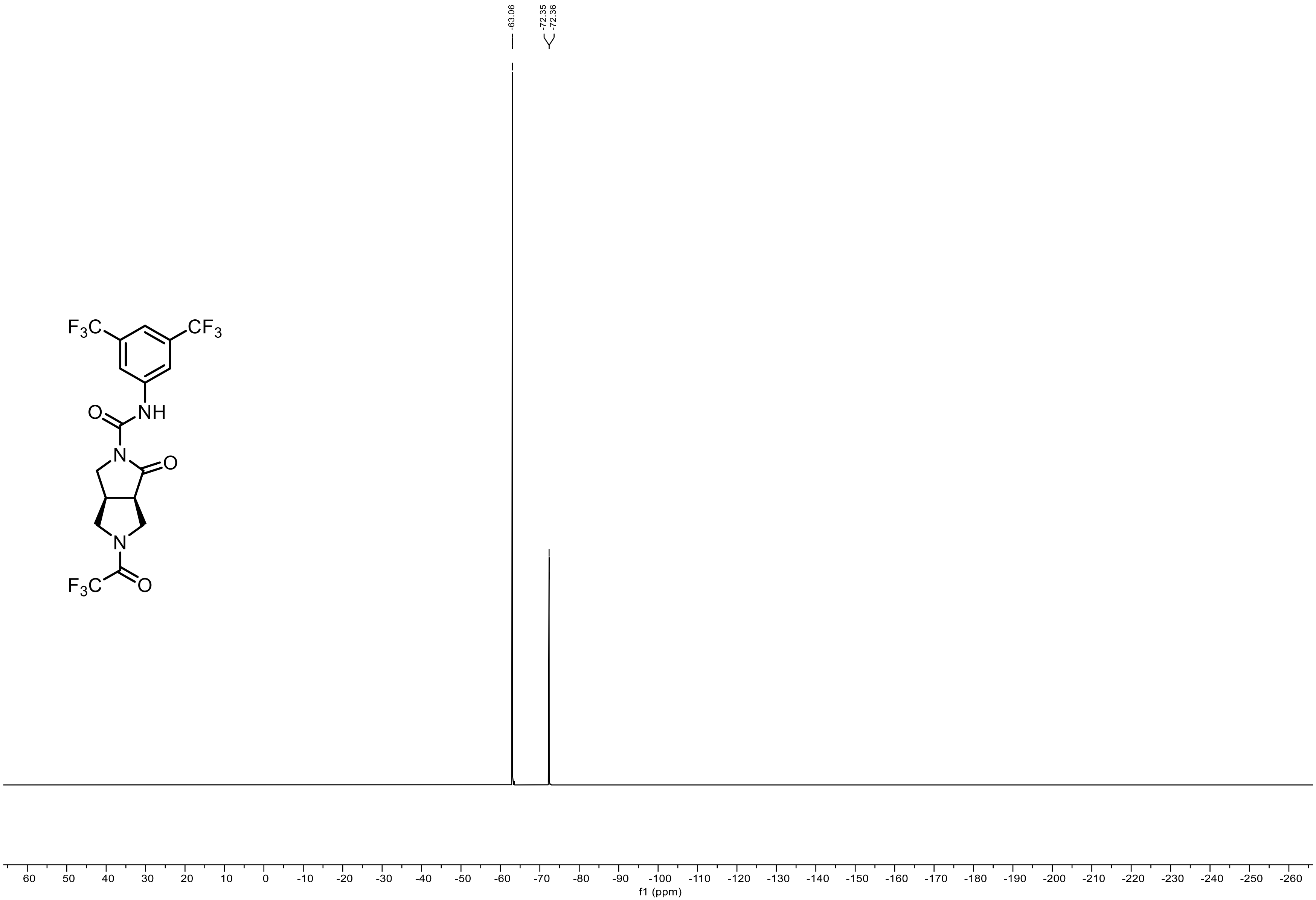

12 ^{19}F NMR (470 MHz, $CDCl_3$, mixture of rotamers).

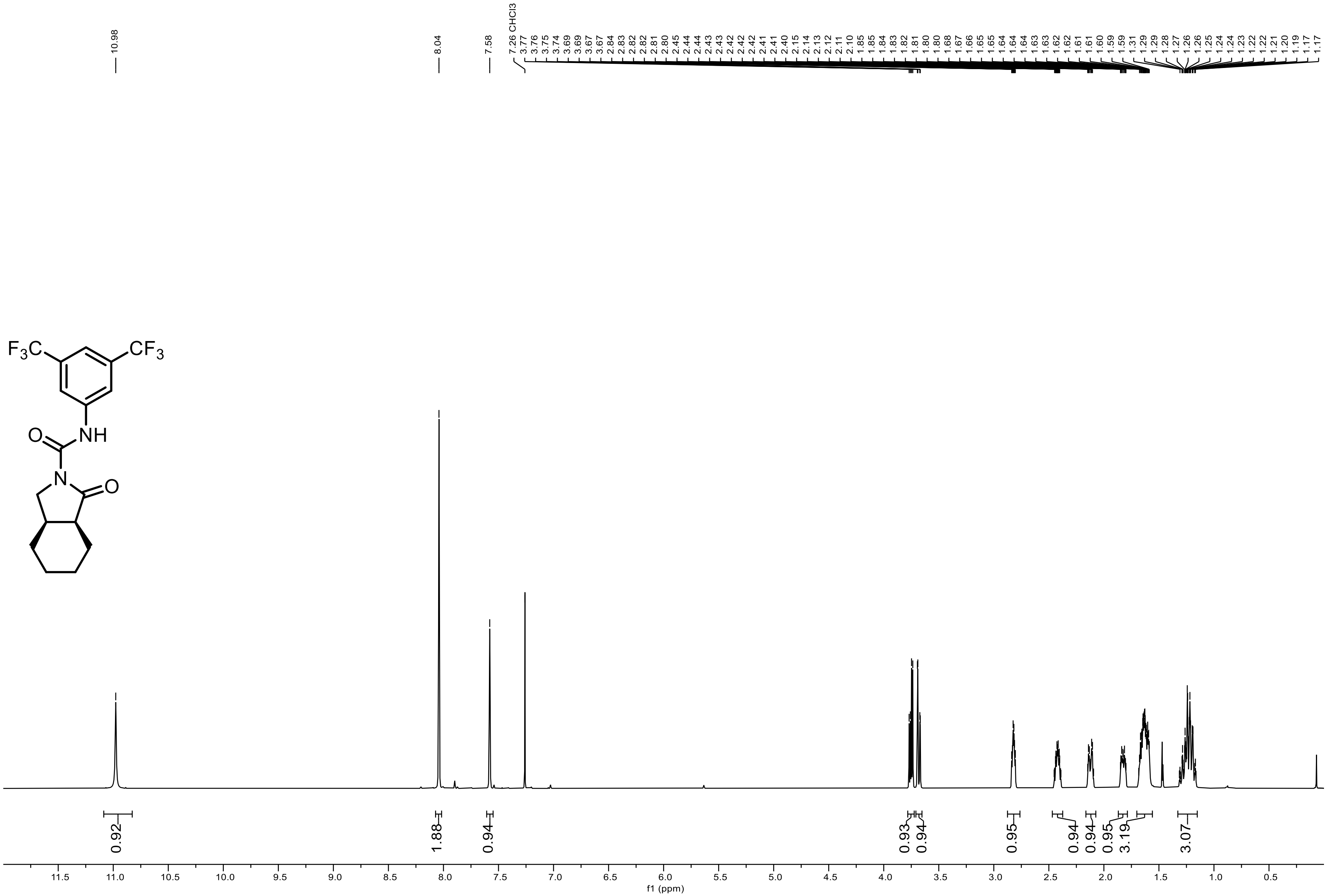

13 ^{1}H NMR (500 MHz, $CDCl_3$).

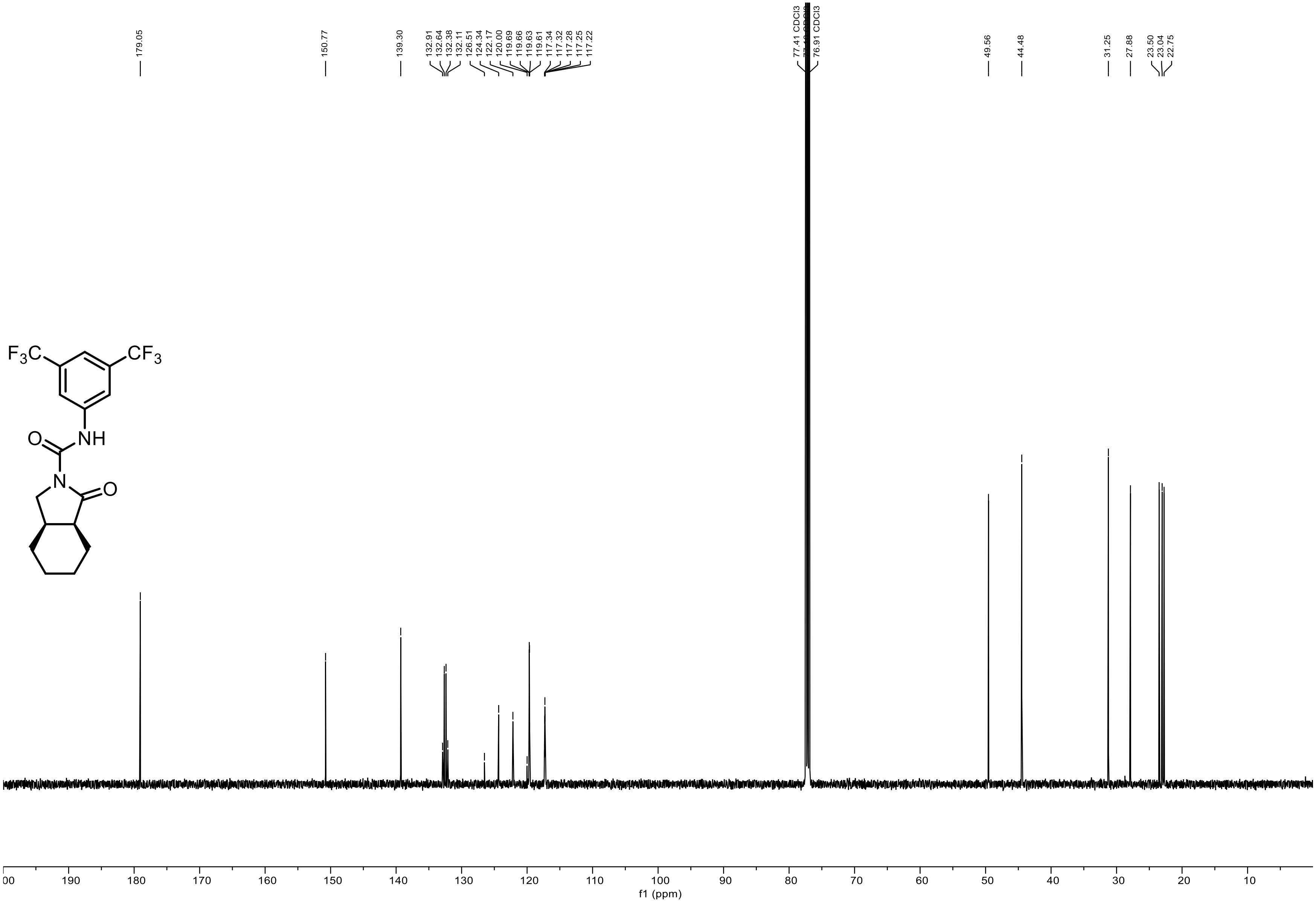

13 ^{13}C NMR (126 MHz, CDCl$_3$).

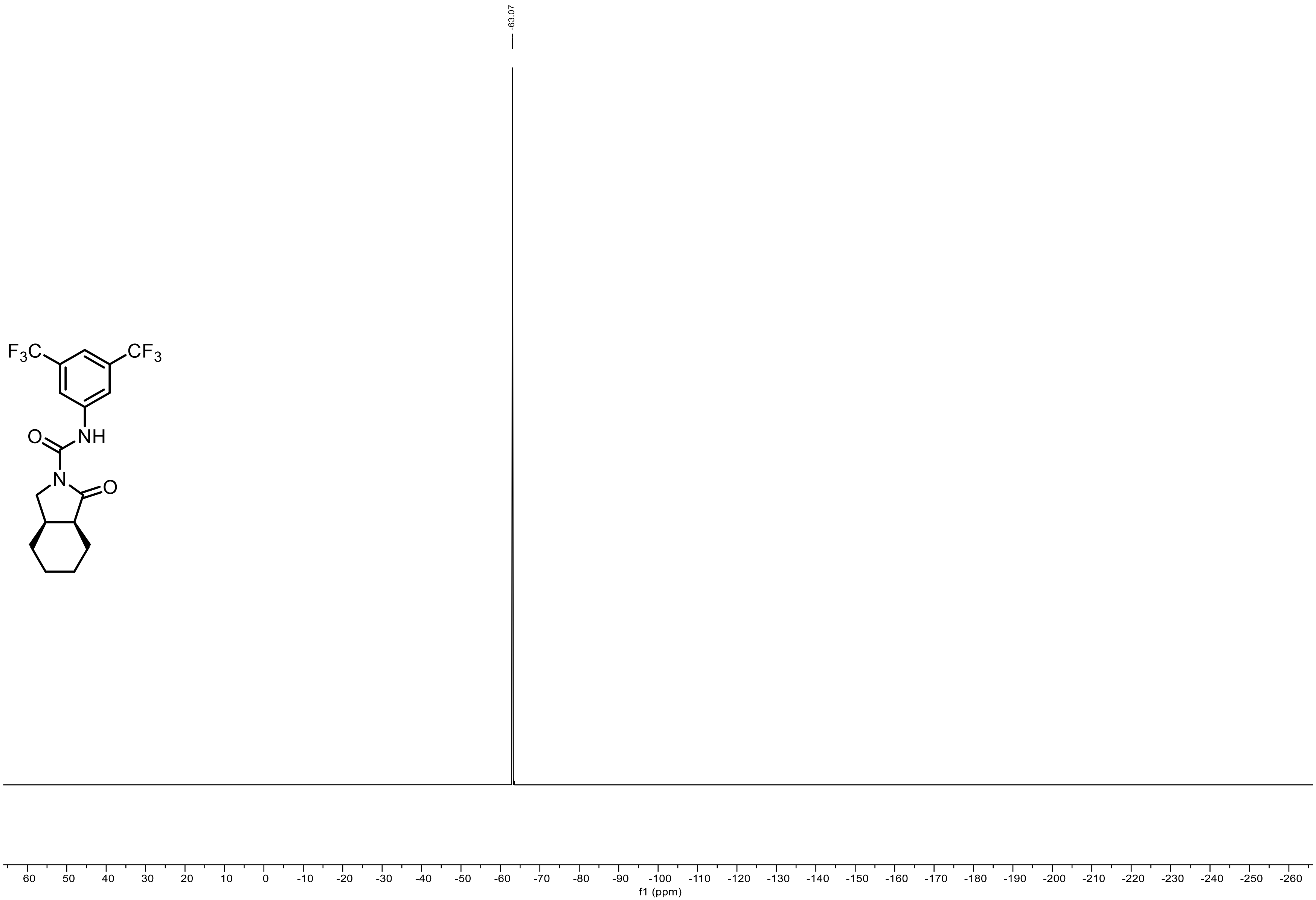

13 ^{19}F NMR (470 MHz, $CDCl_3$).

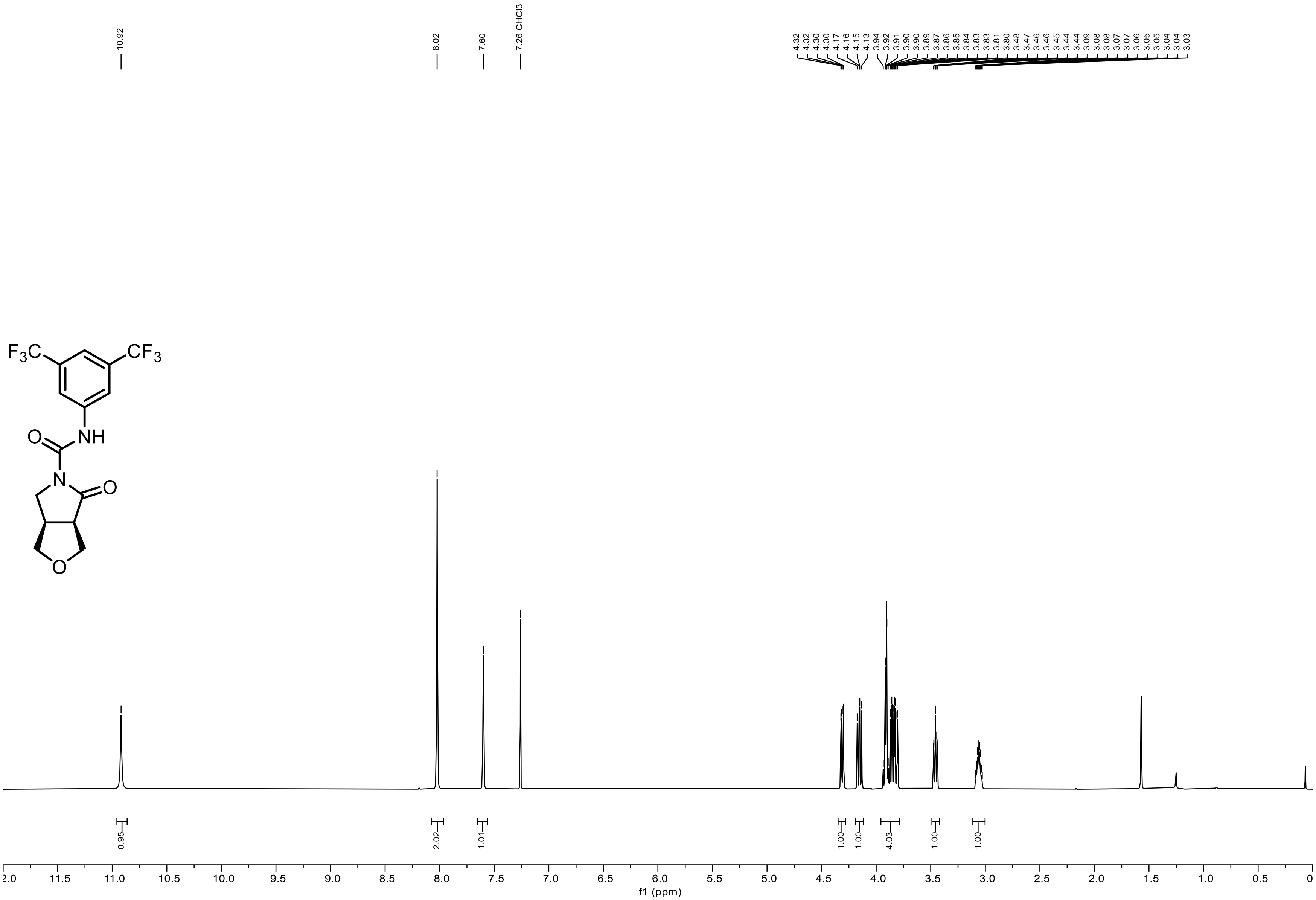

14 ^{1}H NMR (500 MHz, $CDCl_3$).

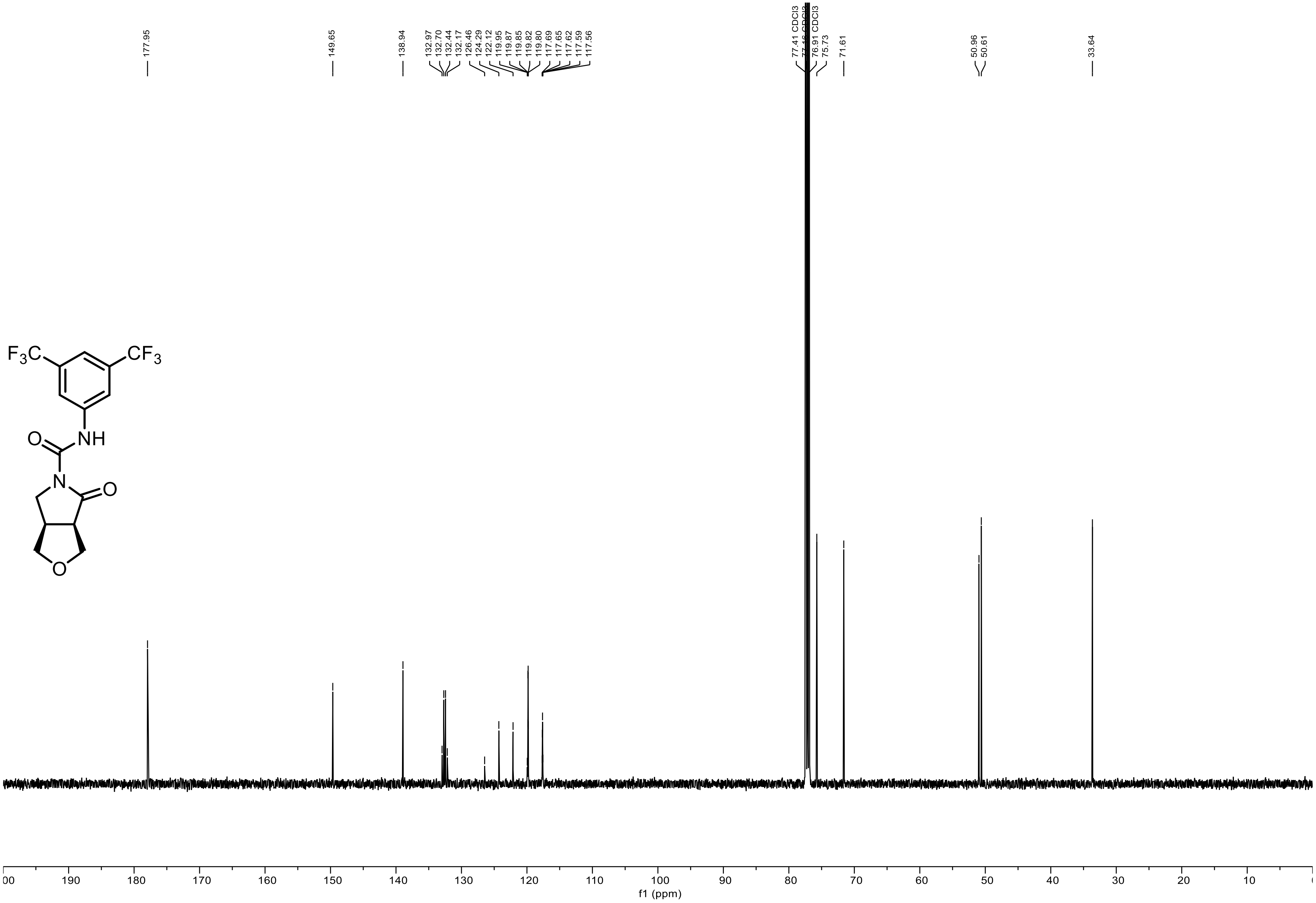

14 ^{13}C NMR (126 MHz, $CDCl_3$).

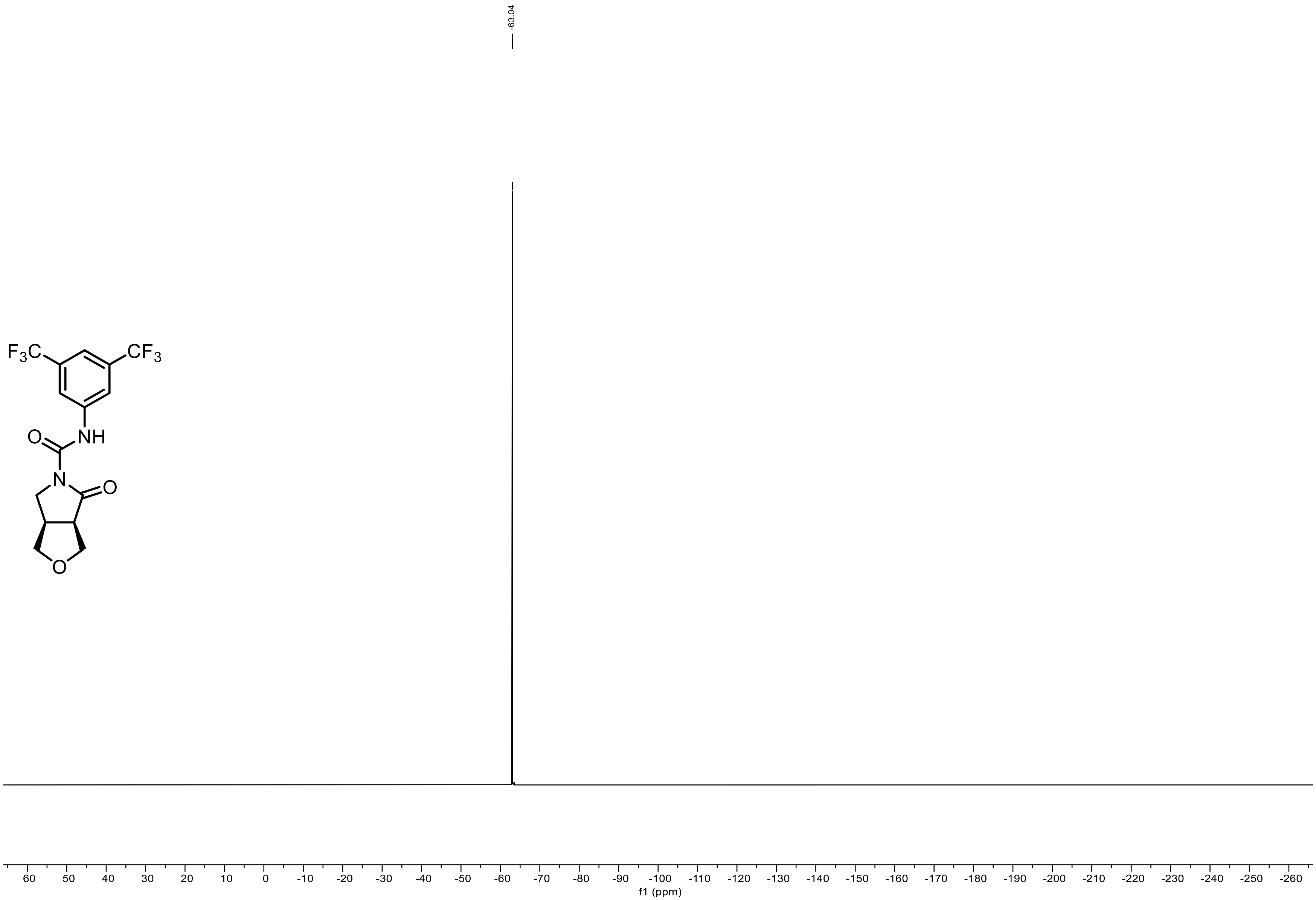

14 ^{19}F NMR (470 MHz, $CDCl_3$).

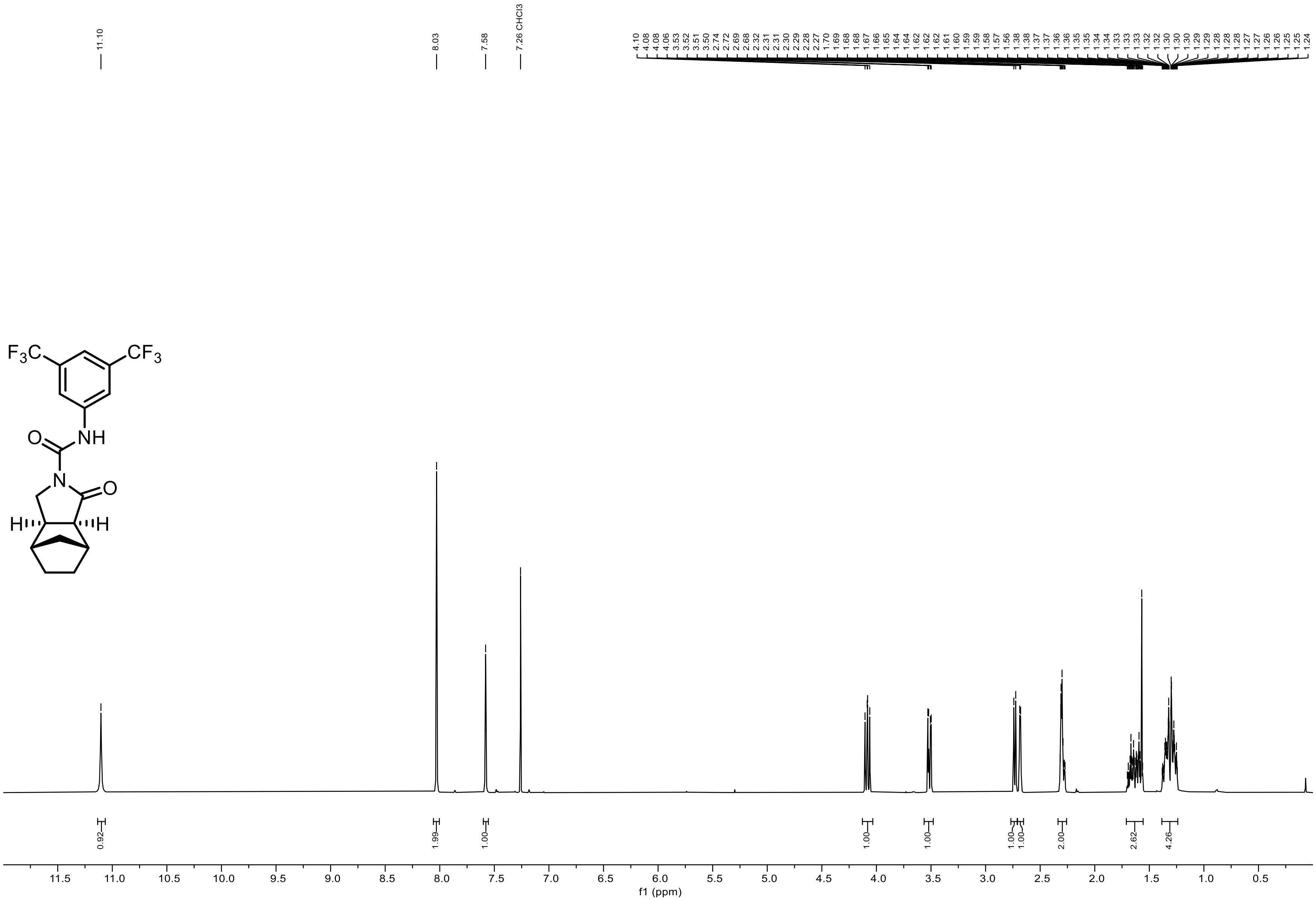

15 ^{1}H NMR (500 MHz, $CDCl_3$).

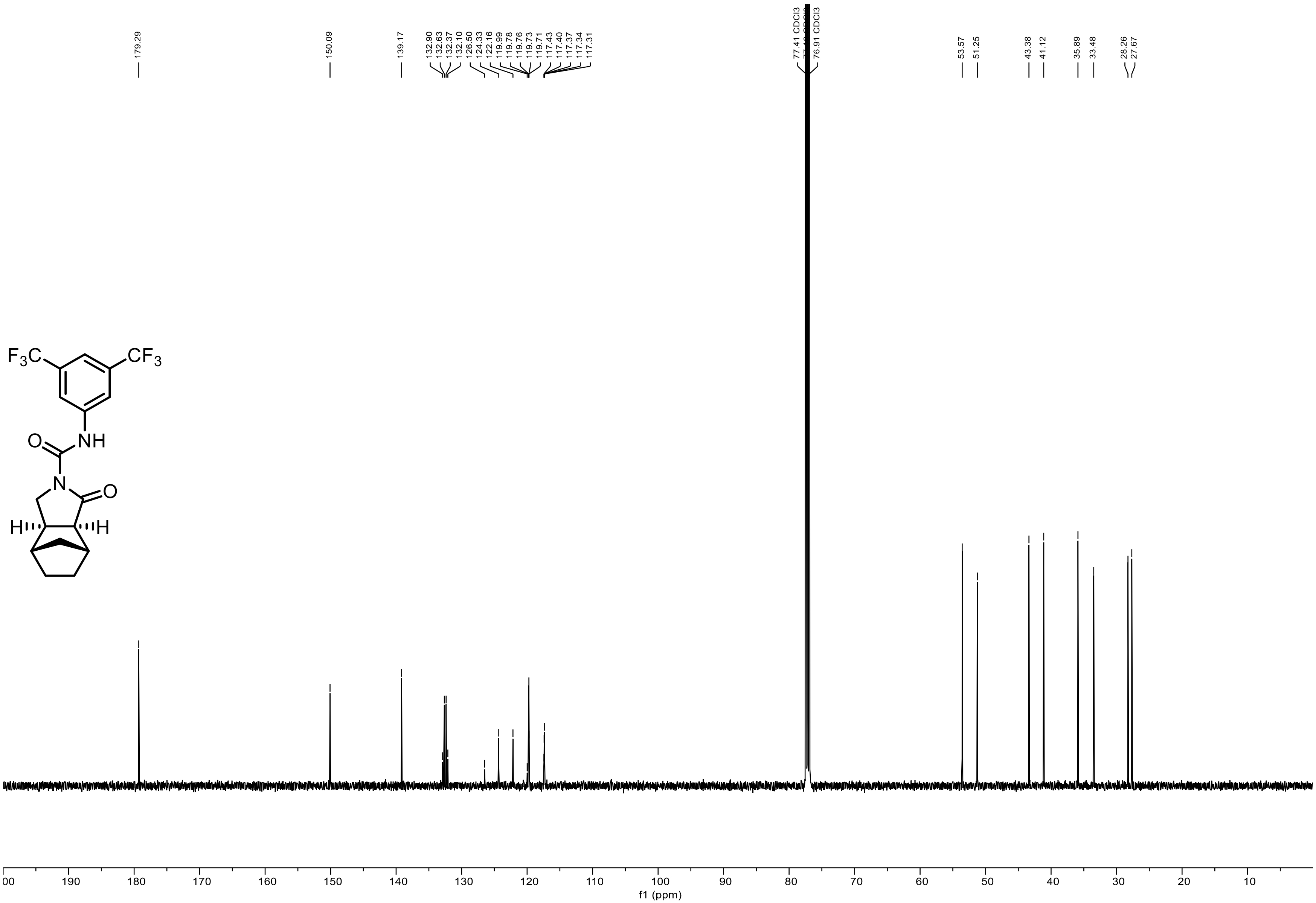

15 ^{13}C NMR (126 MHz, $CDCl_3$).

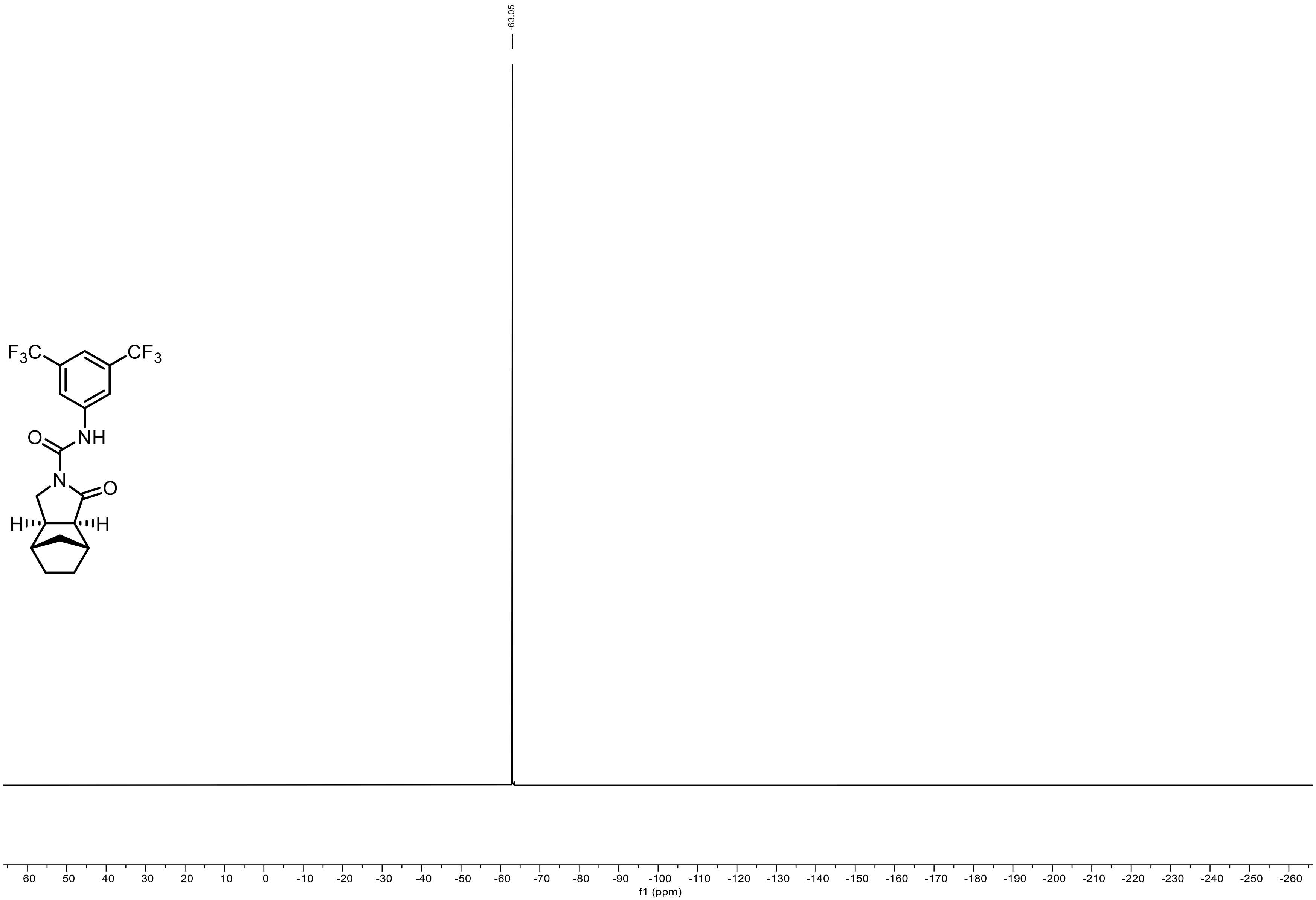

15 ^{19}F NMR (470 MHz, $CDCl_3$).

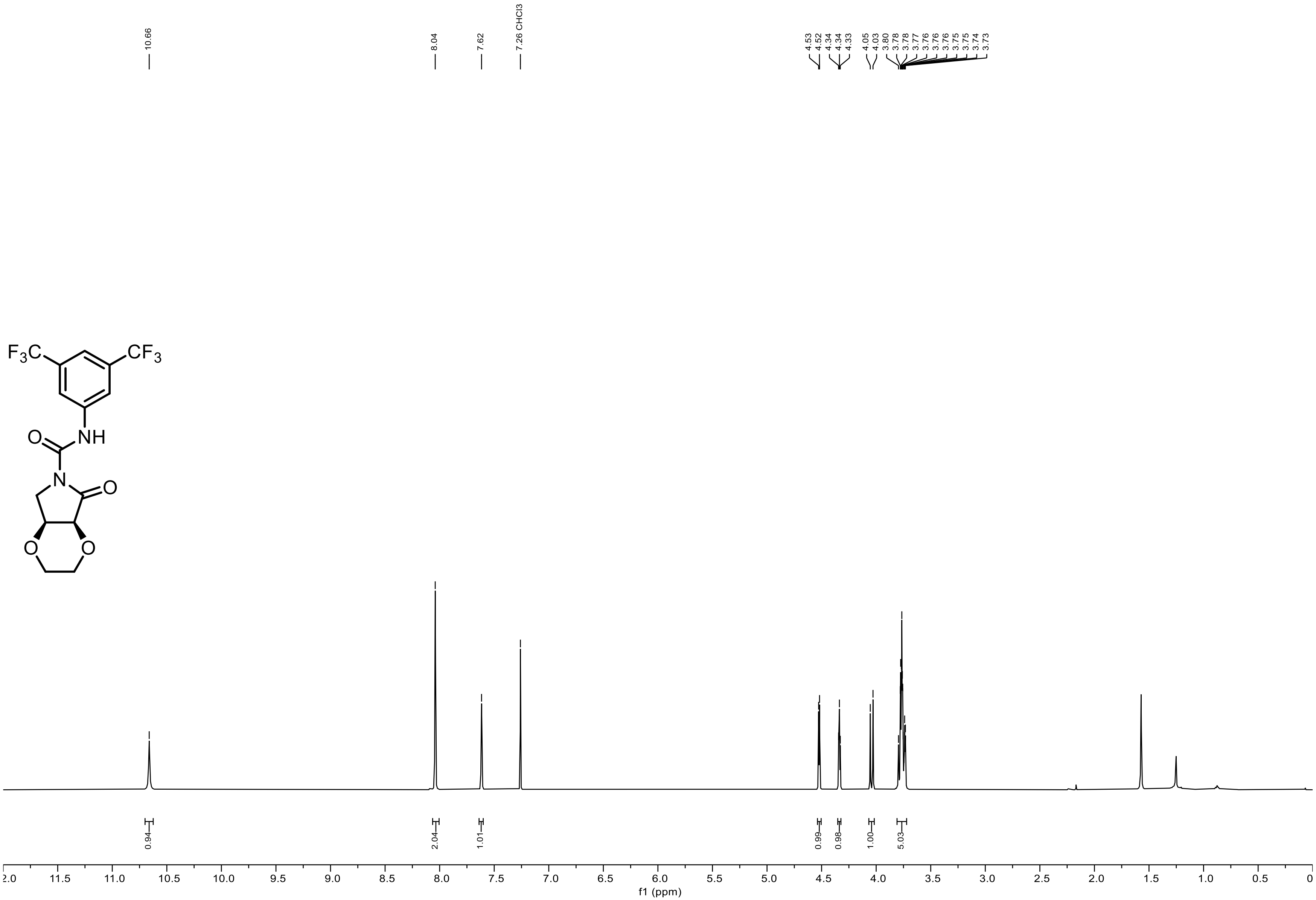

16 ^{1}H NMR (500 MHz, $CDCl_3$).

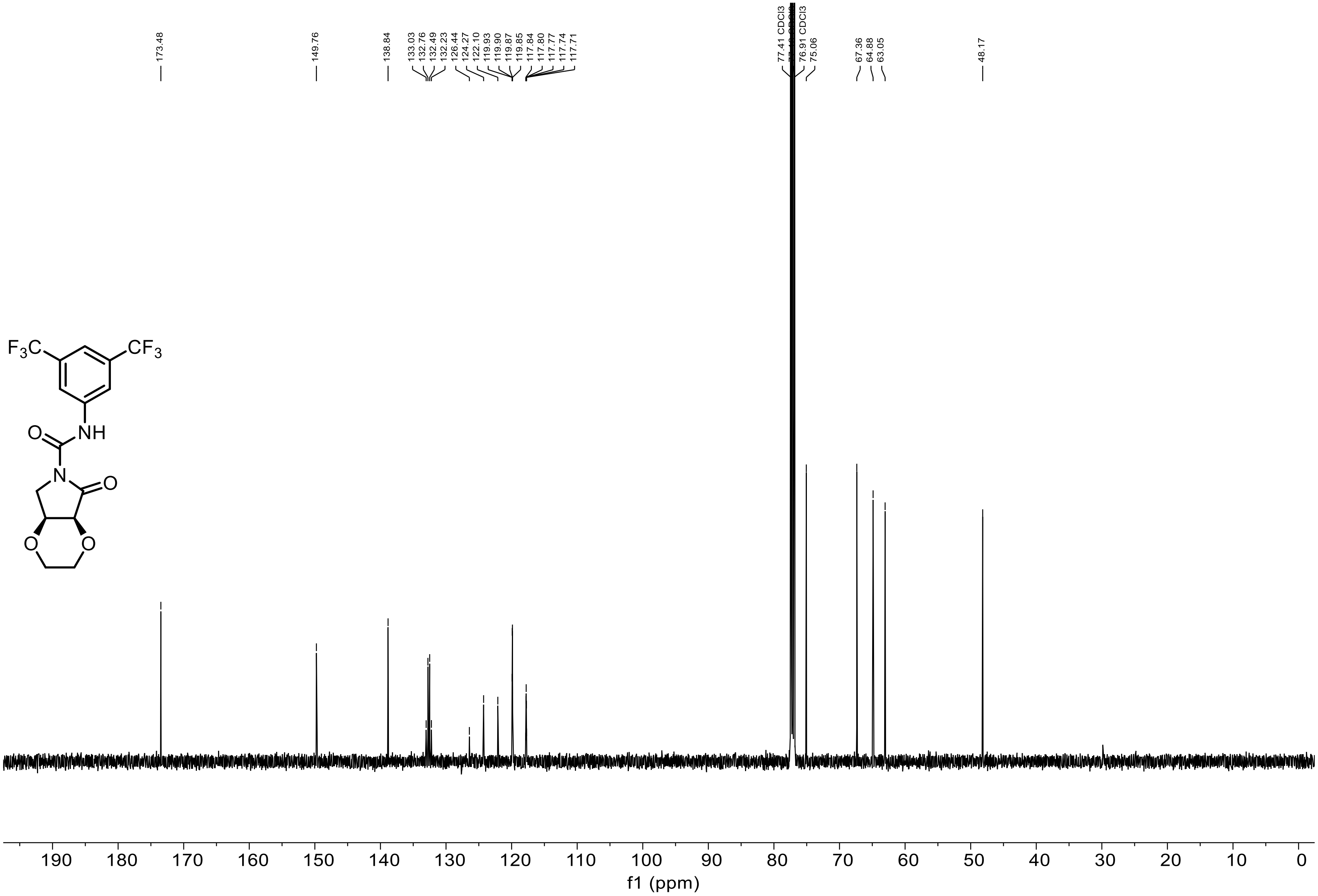

16 ^{13}C NMR (126 MHz, $CDCl_3$).

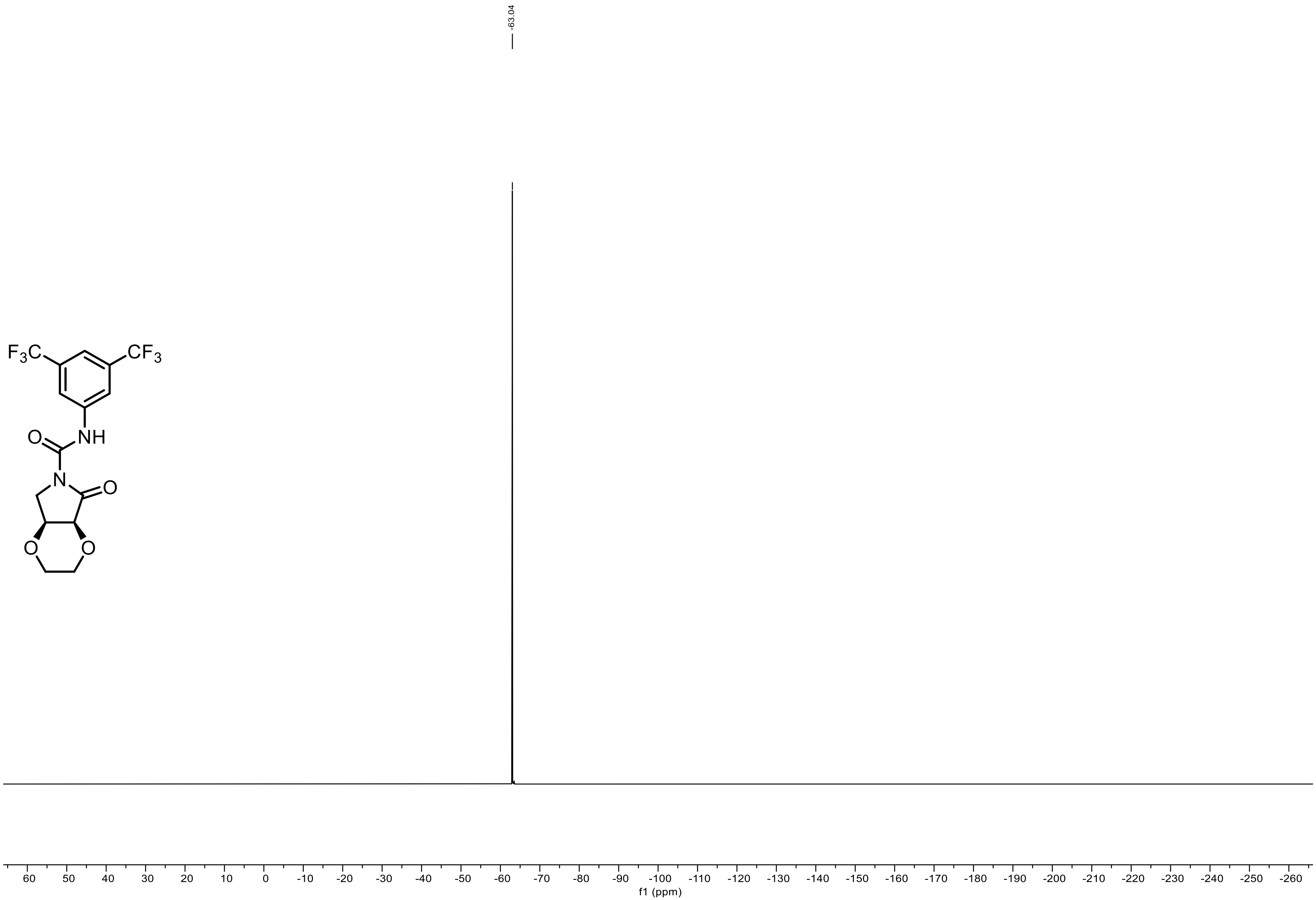

16 ^{19}F NMR (470 MHz, $CDCl_3$).

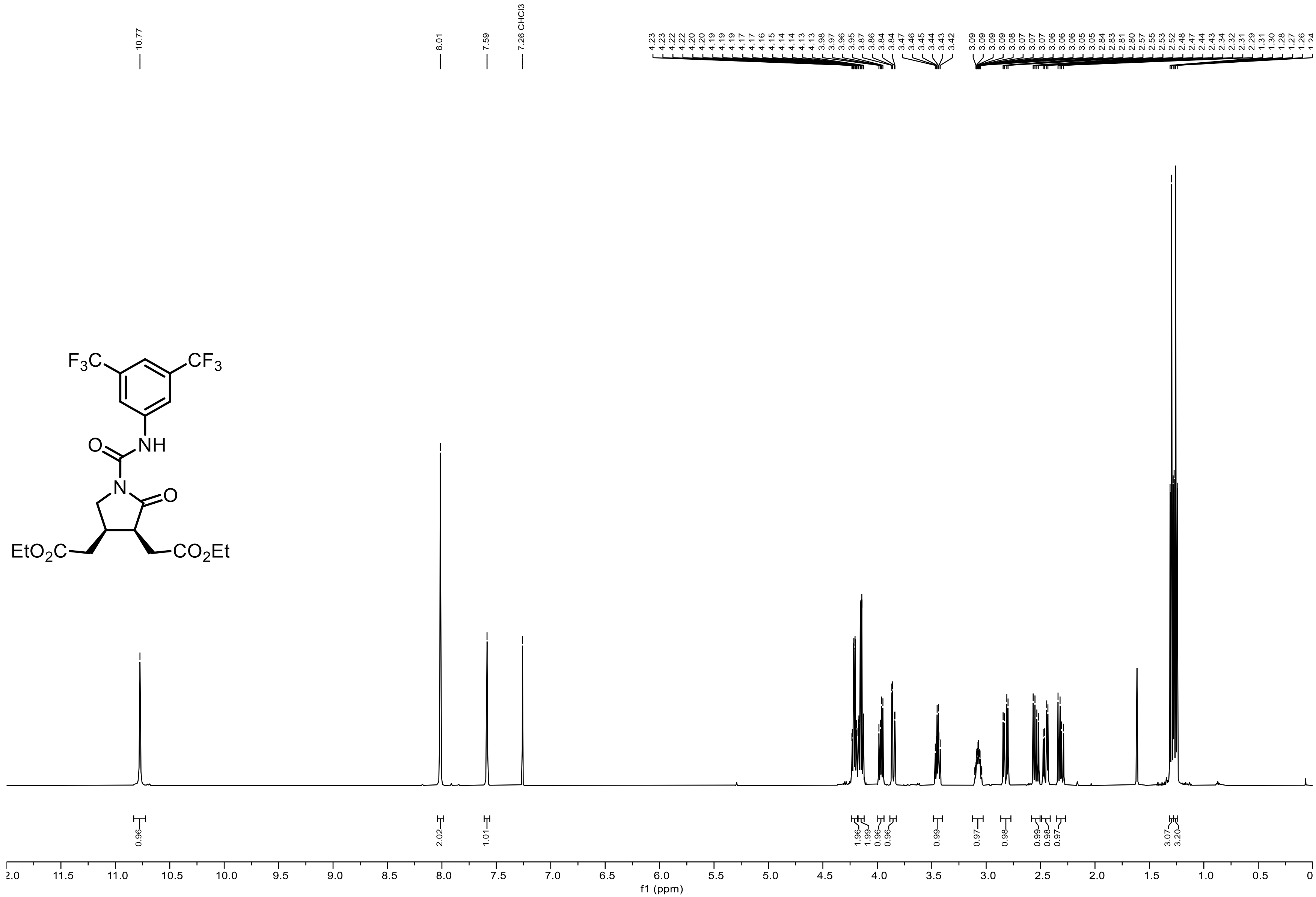

17 ^{1}H NMR (500 MHz, $CDCl_3$).

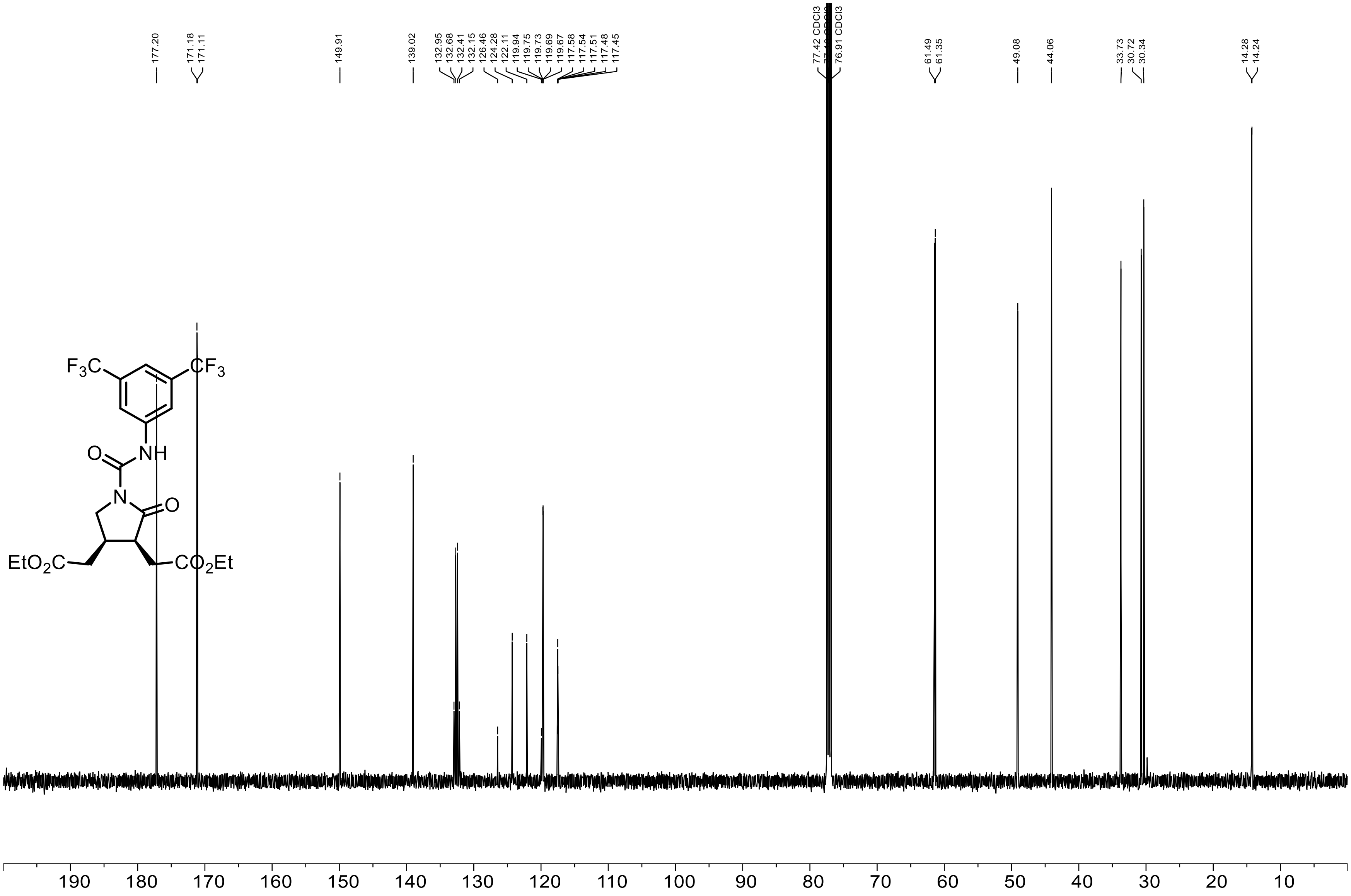

17 ^{13}C NMR (126 MHz, $CDCl_3$).

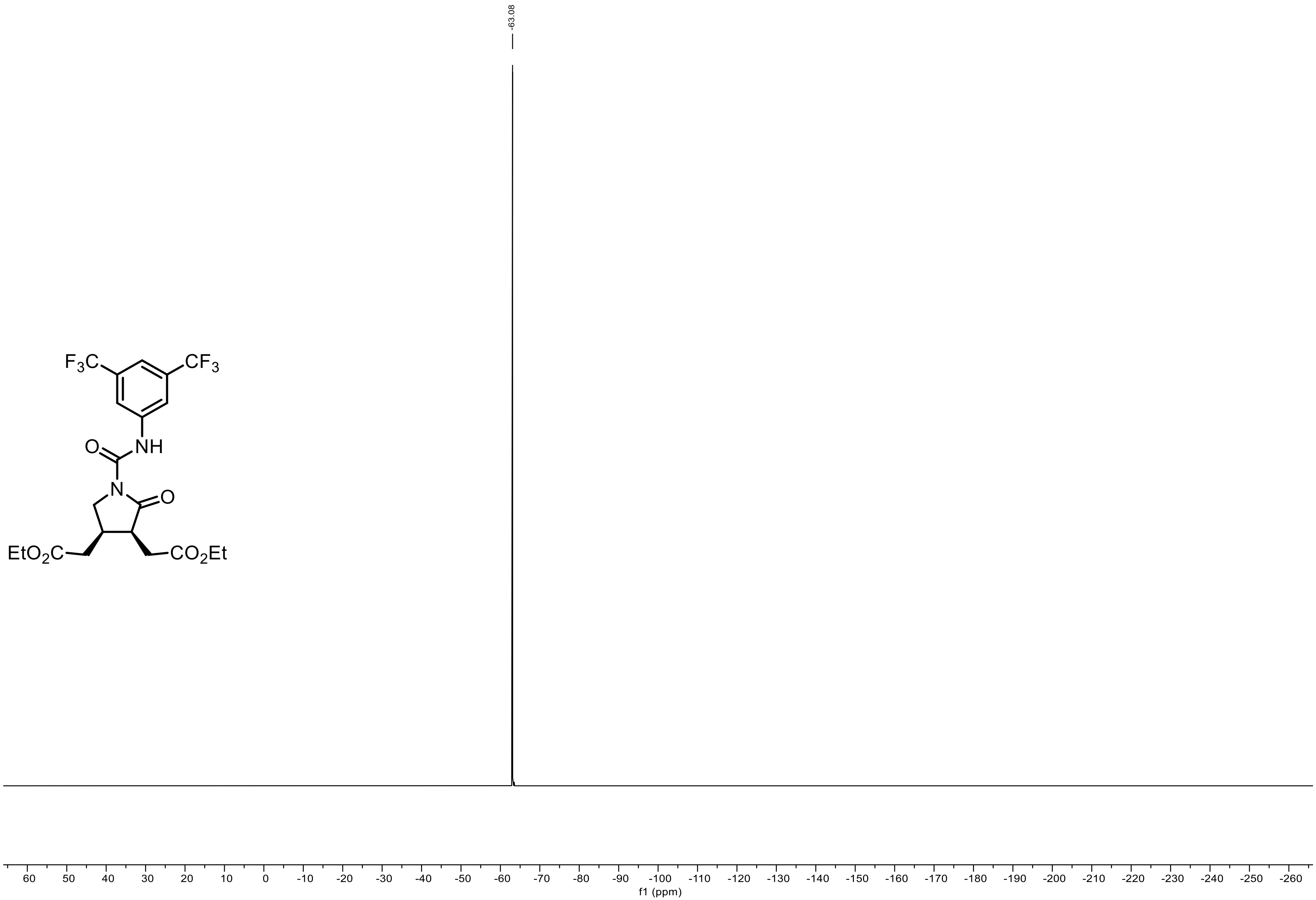

17 ^{19}F NMR (470 MHz, $CDCl_3$).

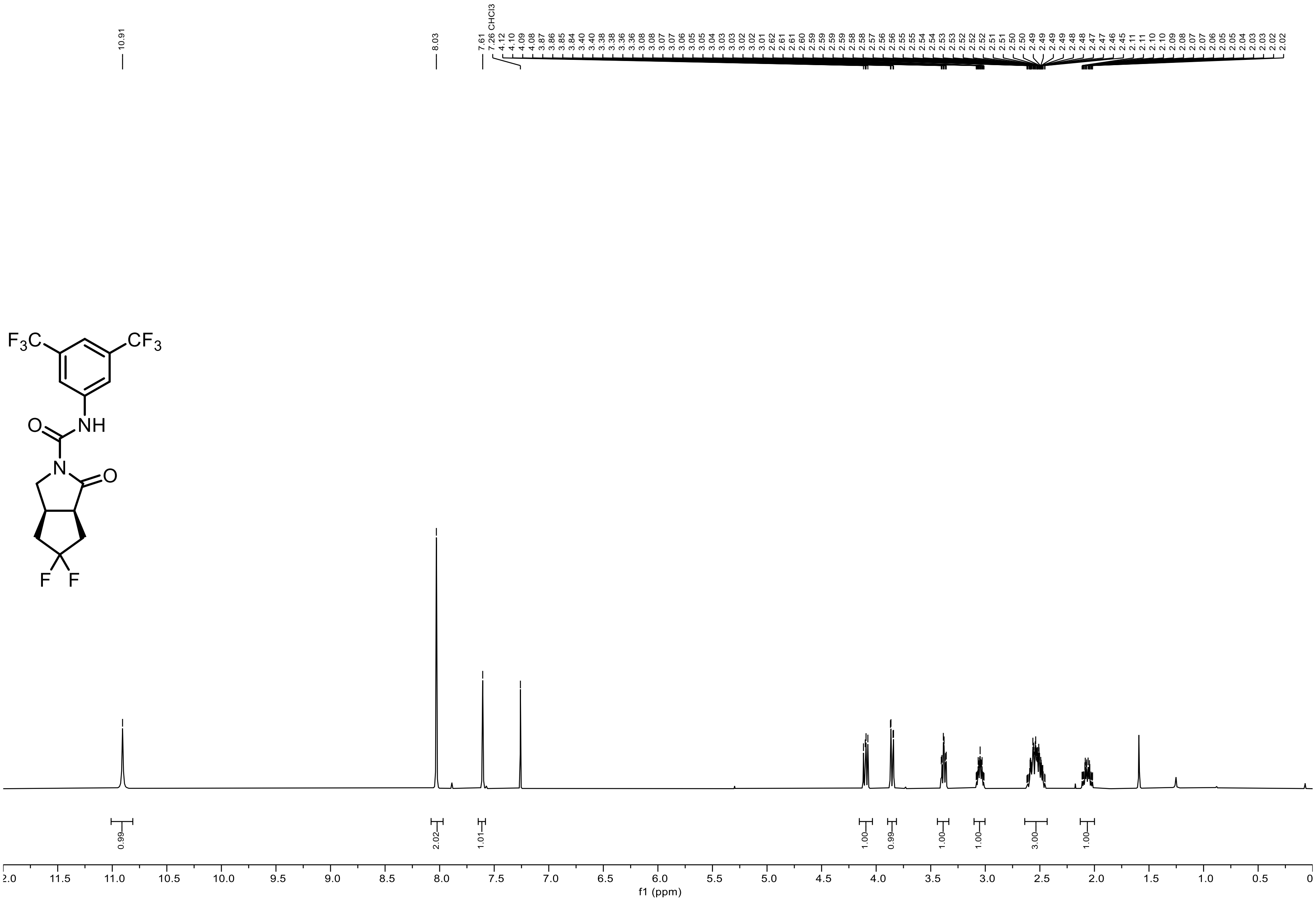

18 ^{1}H NMR (500 MHz, $CDCl_3$).

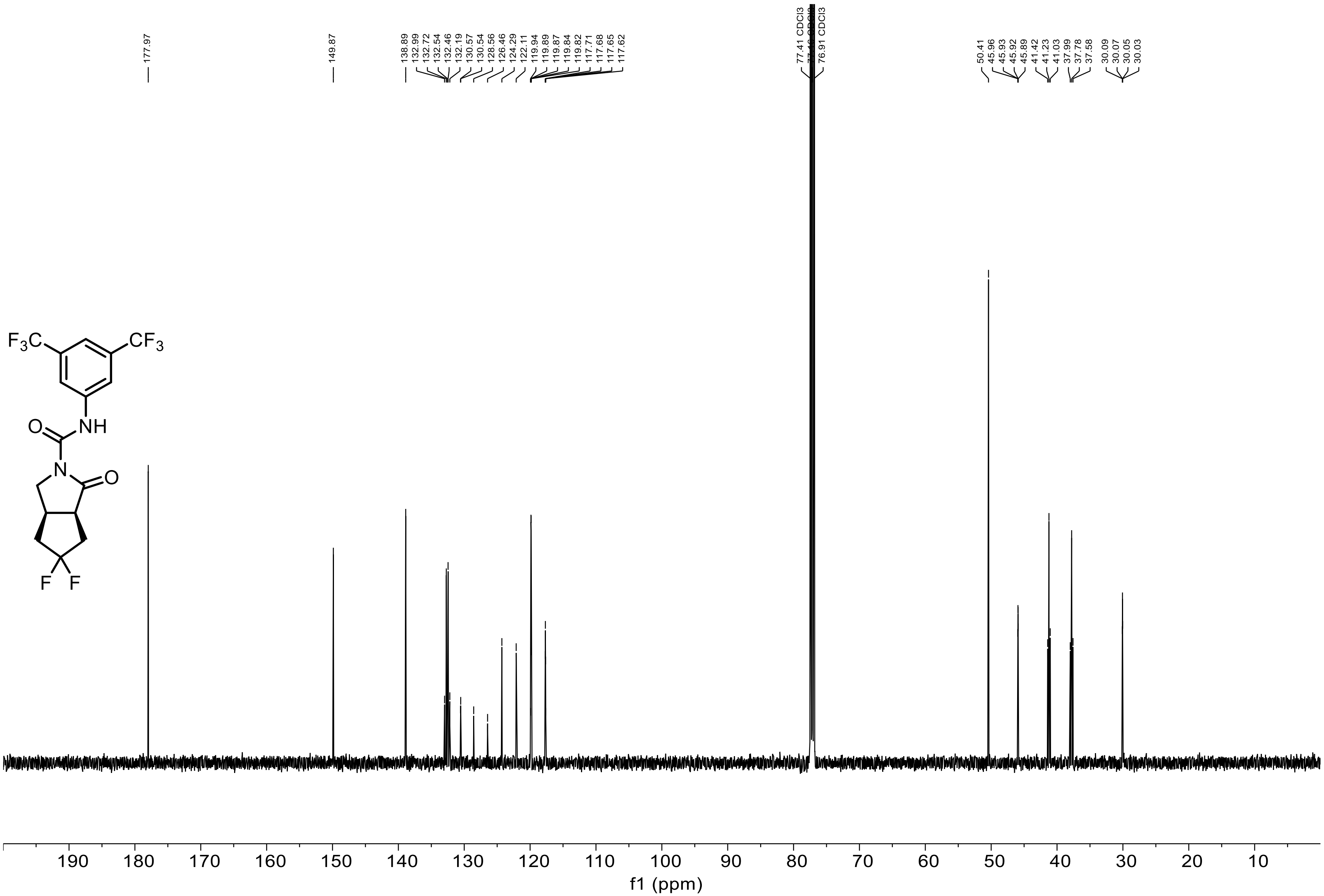

18 ^{13}C NMR (126 MHz, $CDCl_3$).

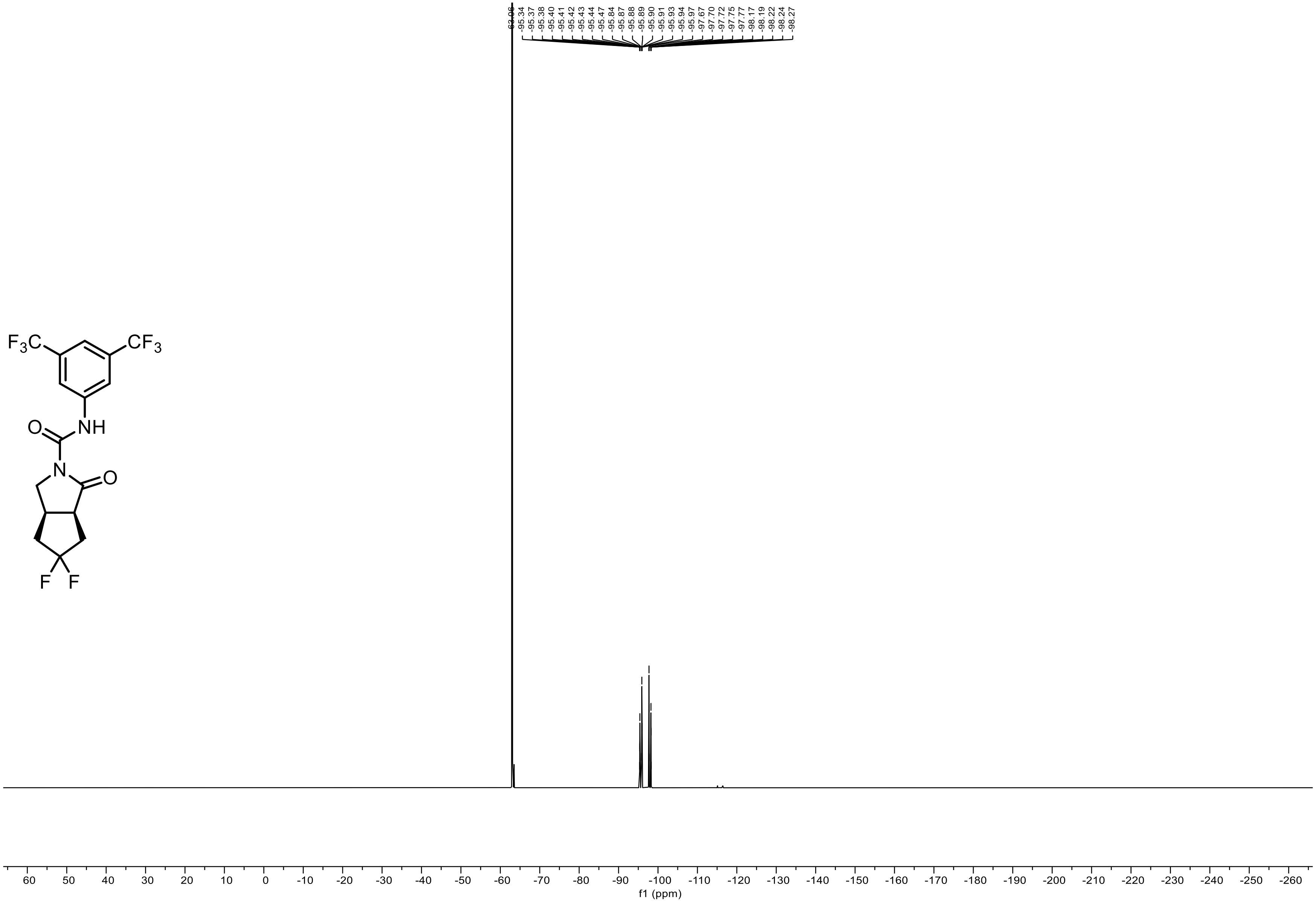

18 ^{19}F NMR (470 MHz, $CDCl_3$).

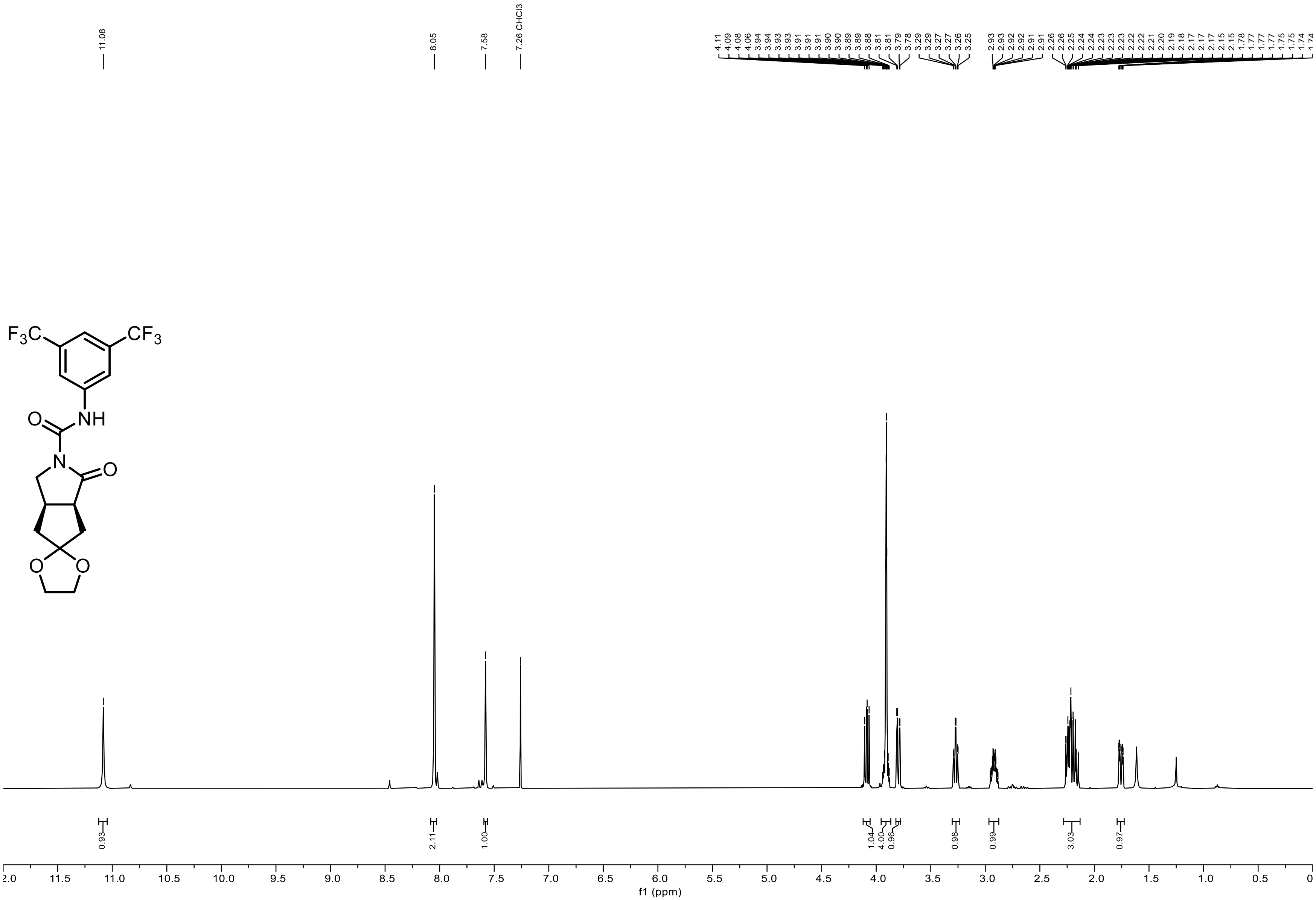

19 ^{1}H NMR (500 MHz, $CDCl_3$).

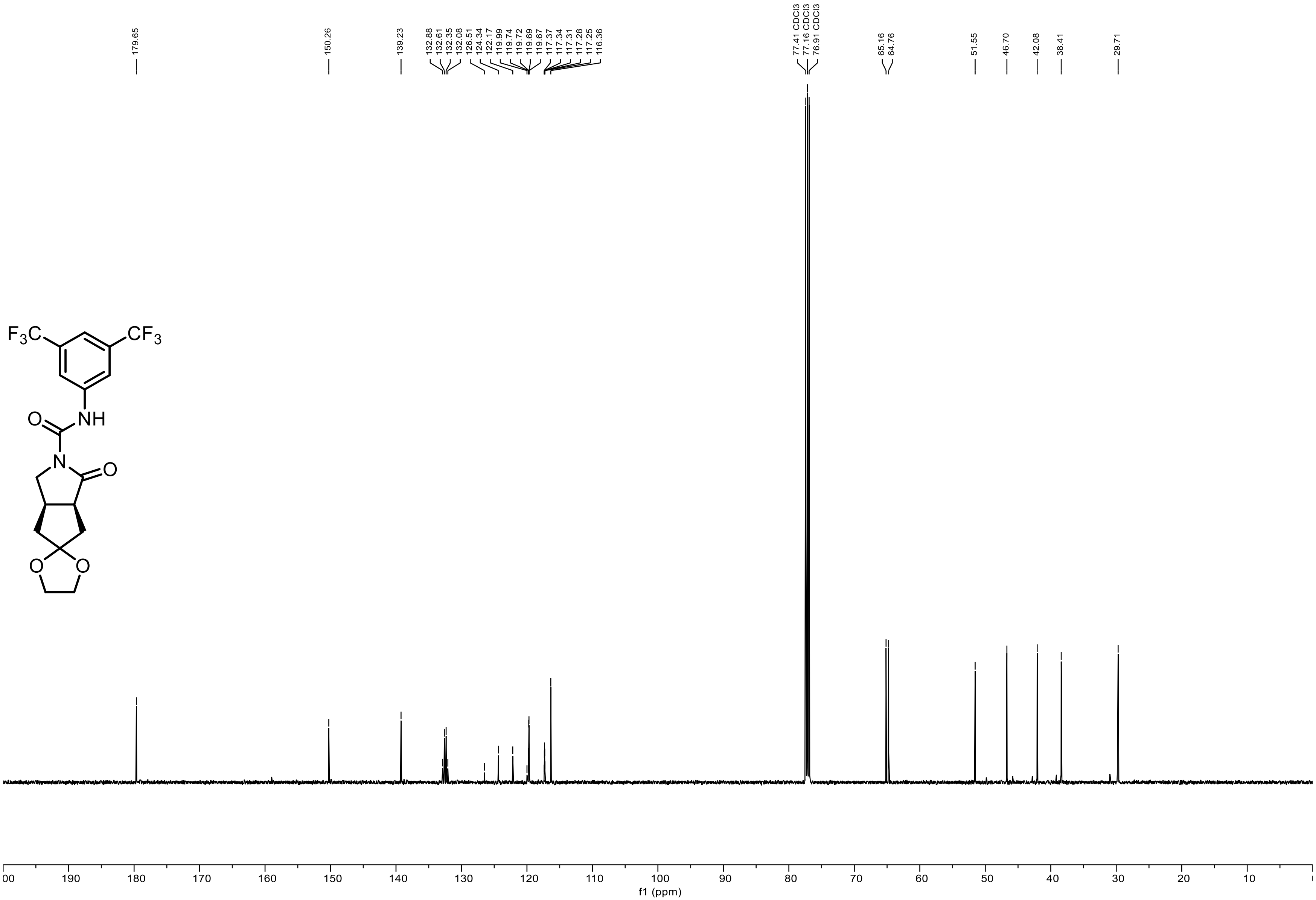

19 ^{13}C NMR (126 MHz, $CDCl_3$).

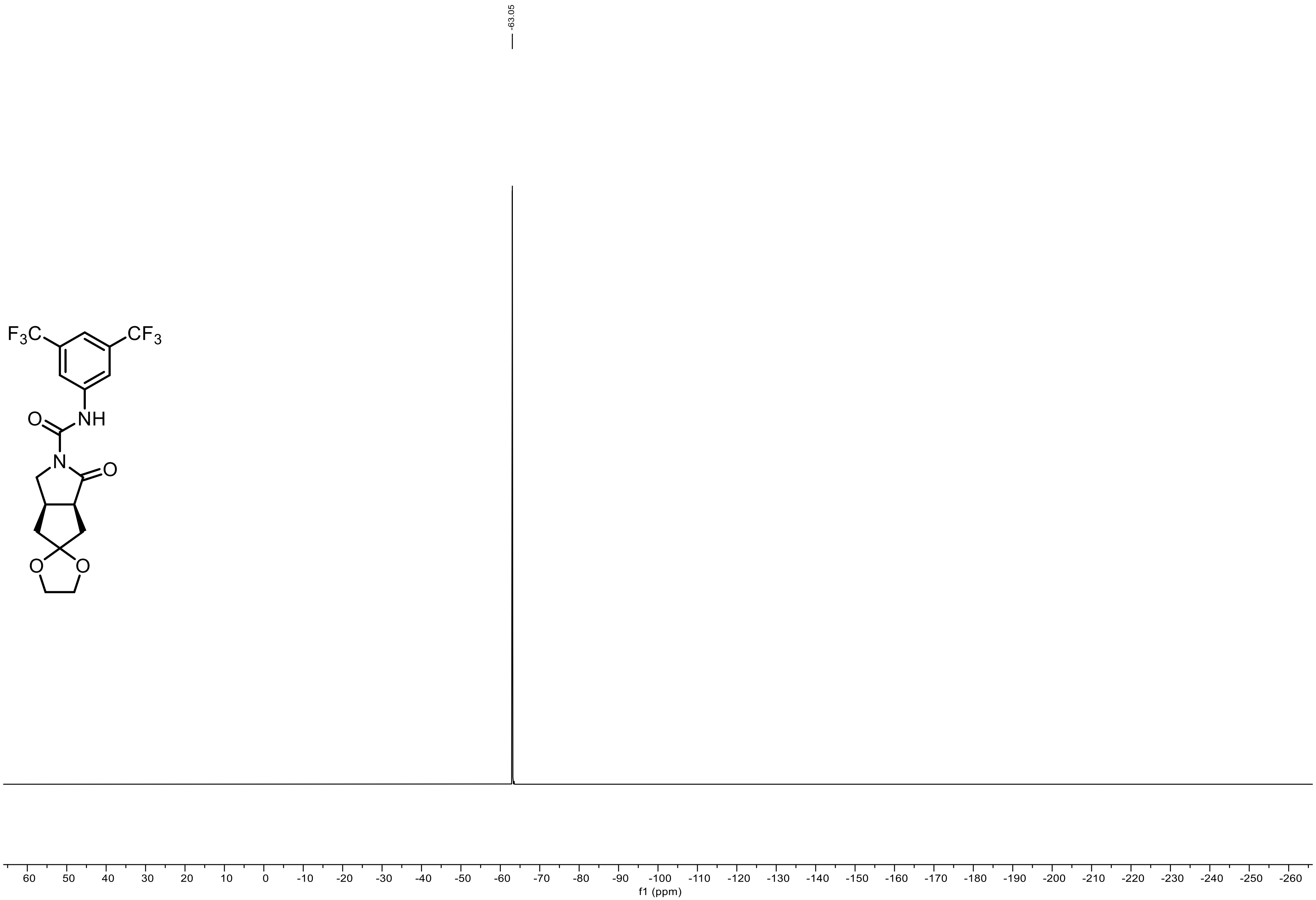

19 ^{19}F NMR (470 MHz, $CDCl_3$).

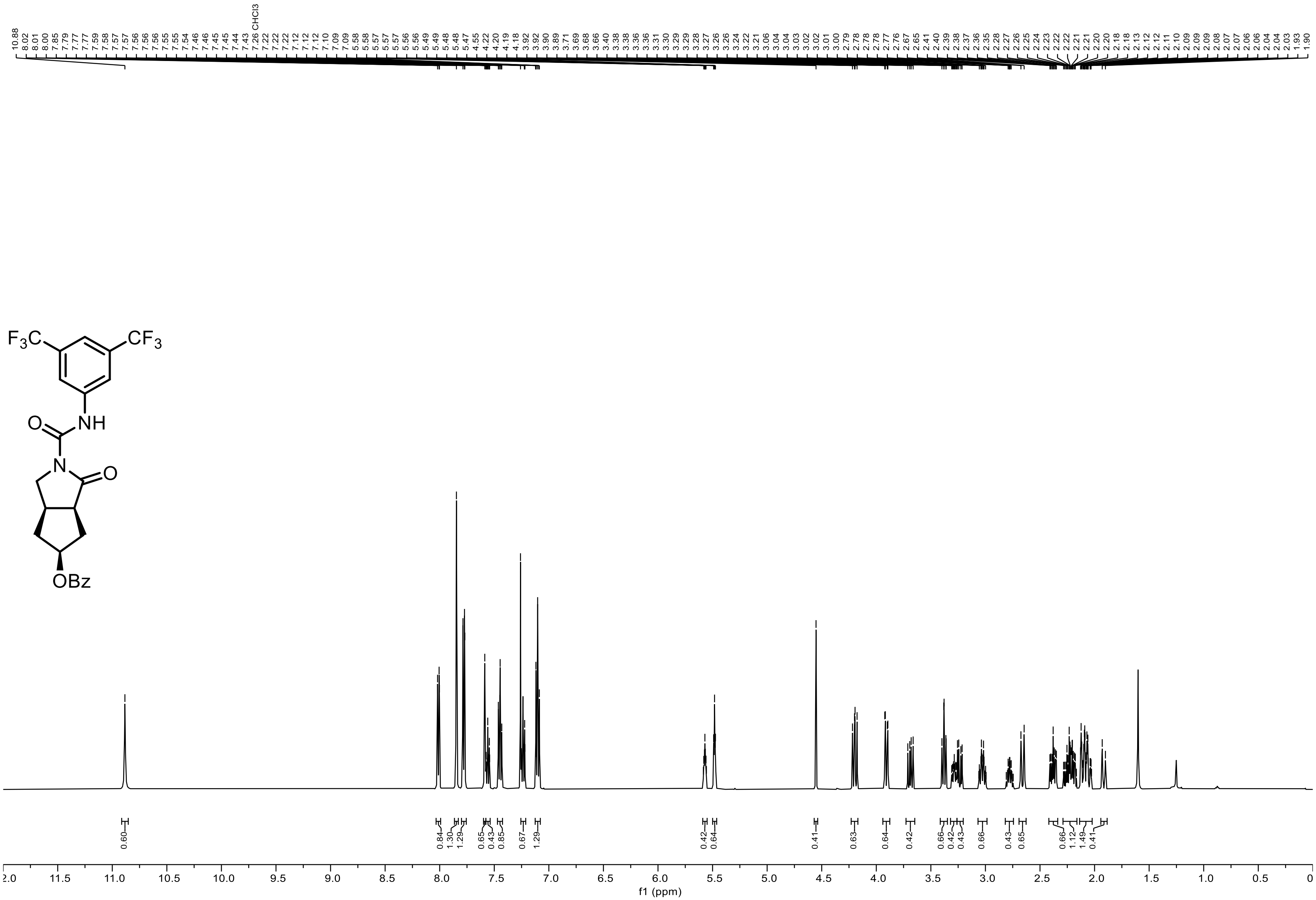

20 ^{1}H NMR (500 MHz, $CDCl_3$, mixture of rotamers).

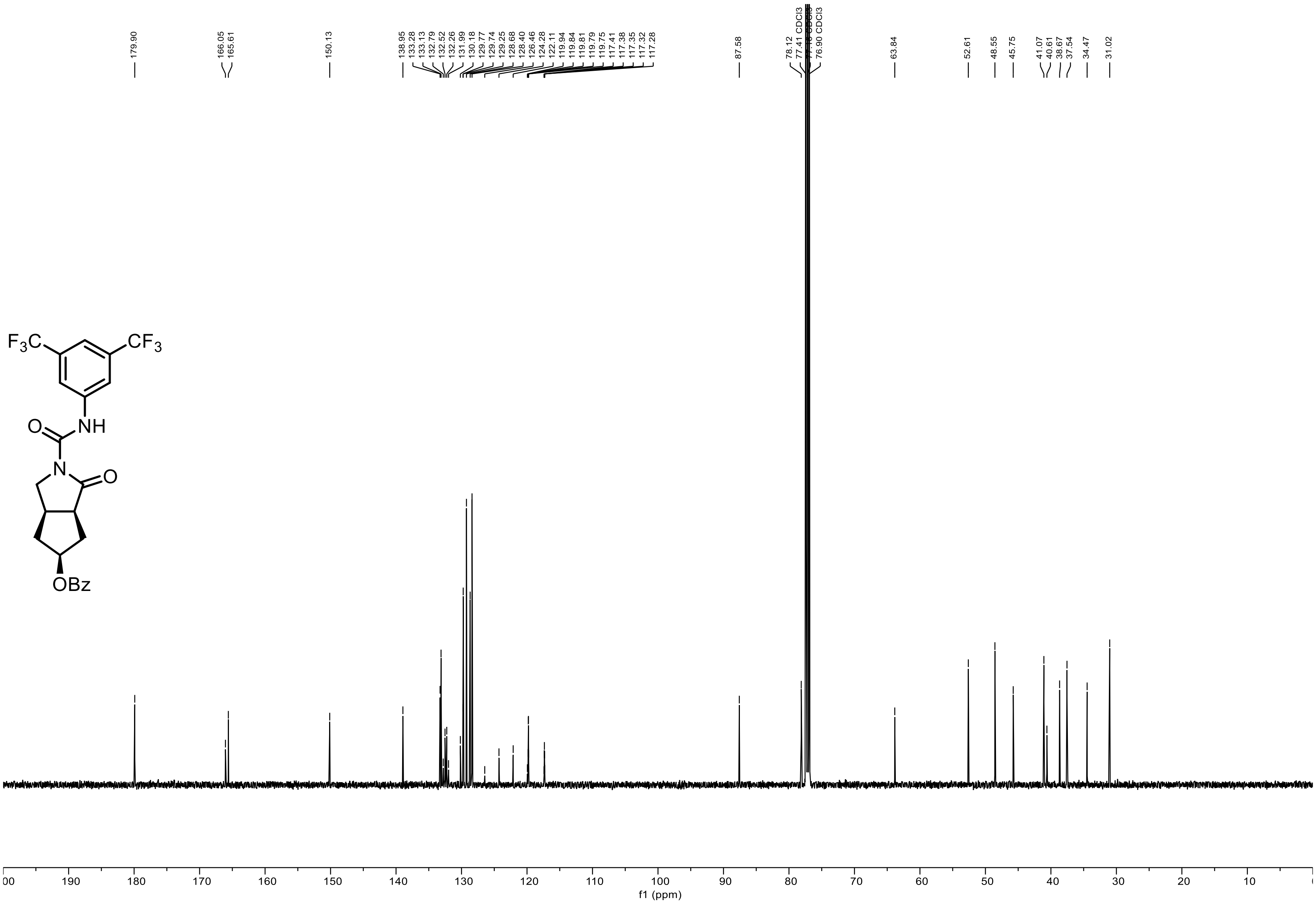

20 ^{13}C NMR (126 MHz, $CDCl_3$, mixture of rotamers).

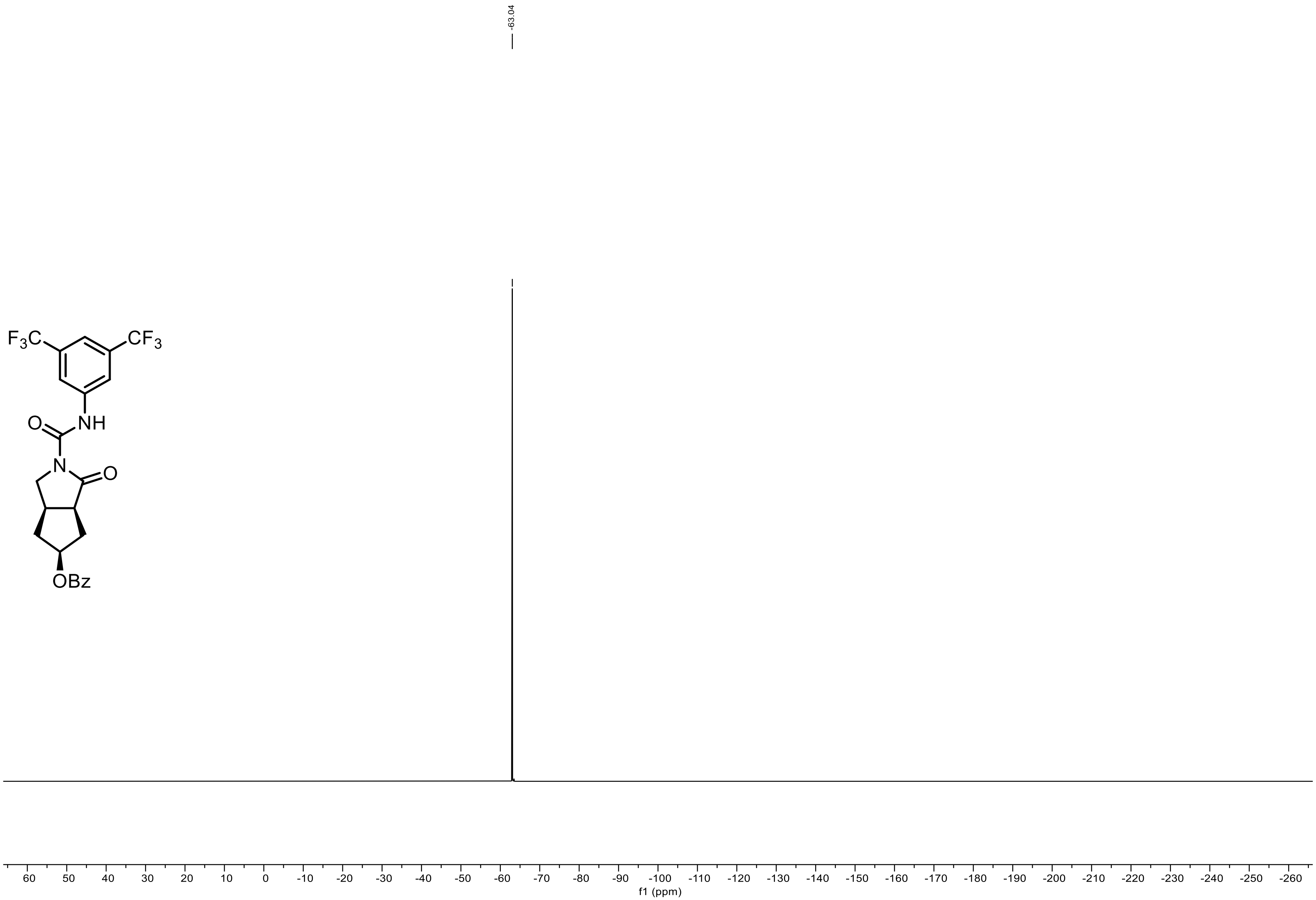

20 ^{19}F NMR (470 MHz, $CDCl_3$).

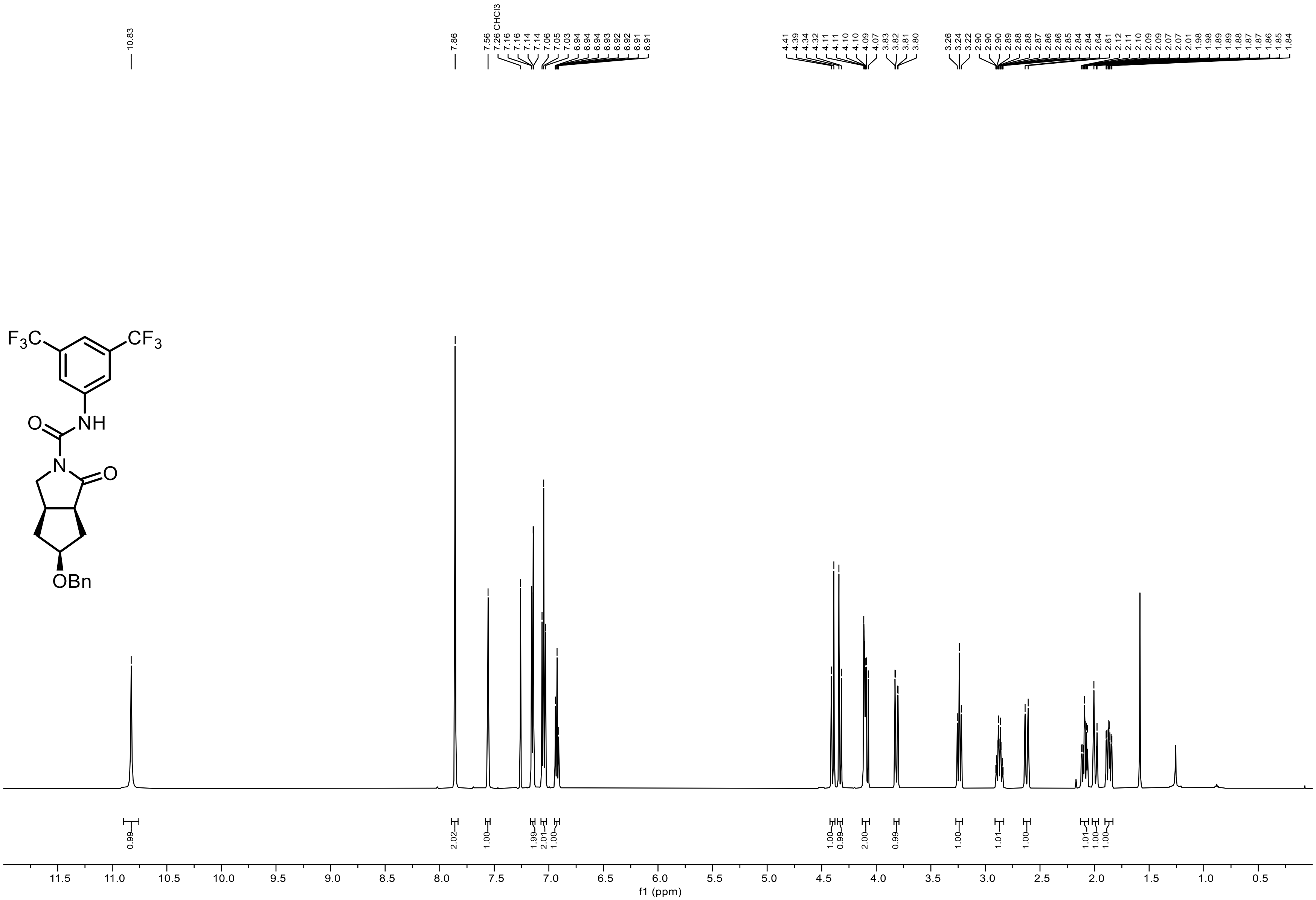

21 ^{1}H NMR (500 MHz, $CDCl_3$).

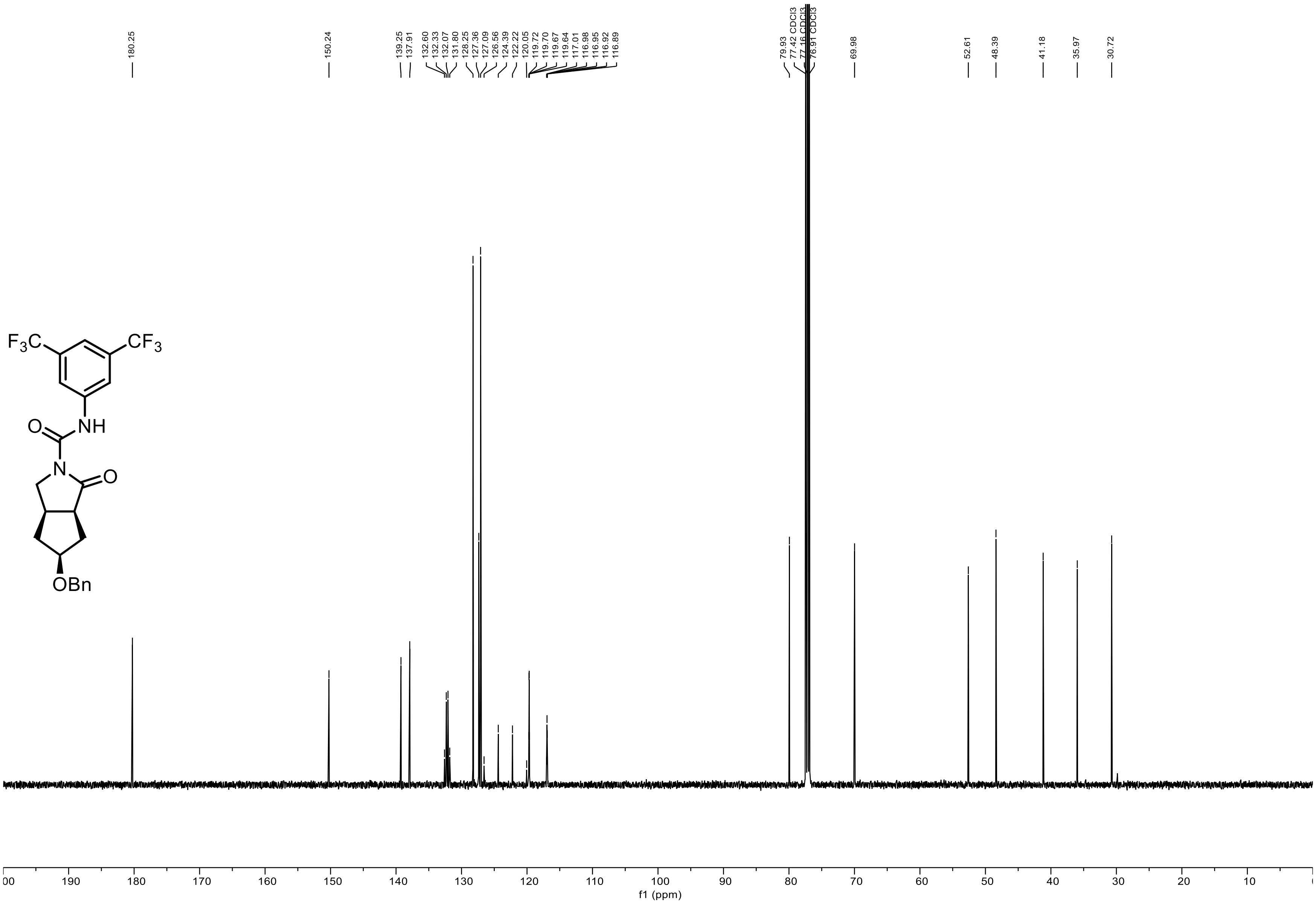

21 ^{13}C NMR (126 MHz, $CDCl_3$).

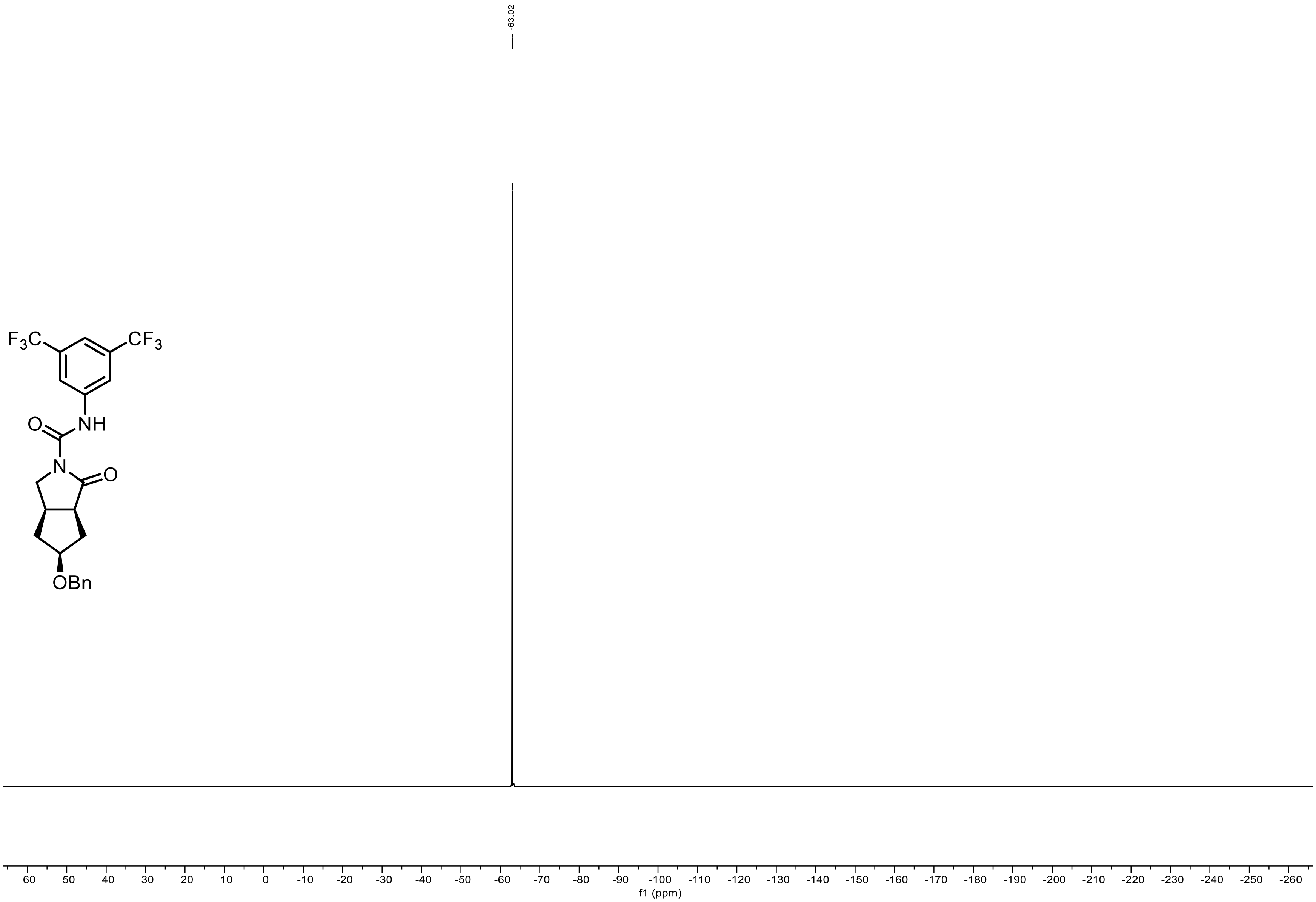

21 ^{19}F NMR (470 MHz, $CDCl_3$).

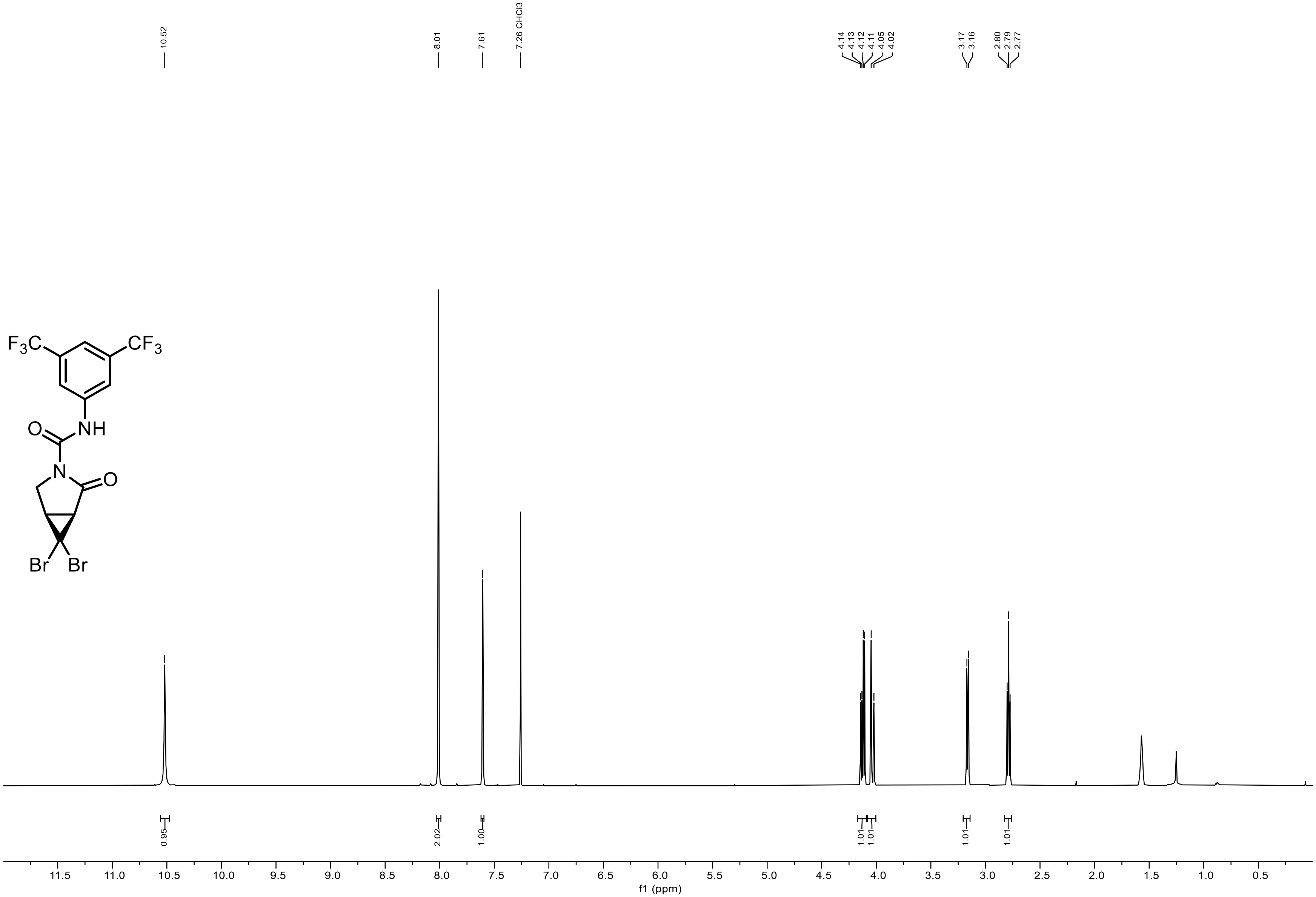

22 ^{1}H NMR (500 MHz, $CDCl_3$).

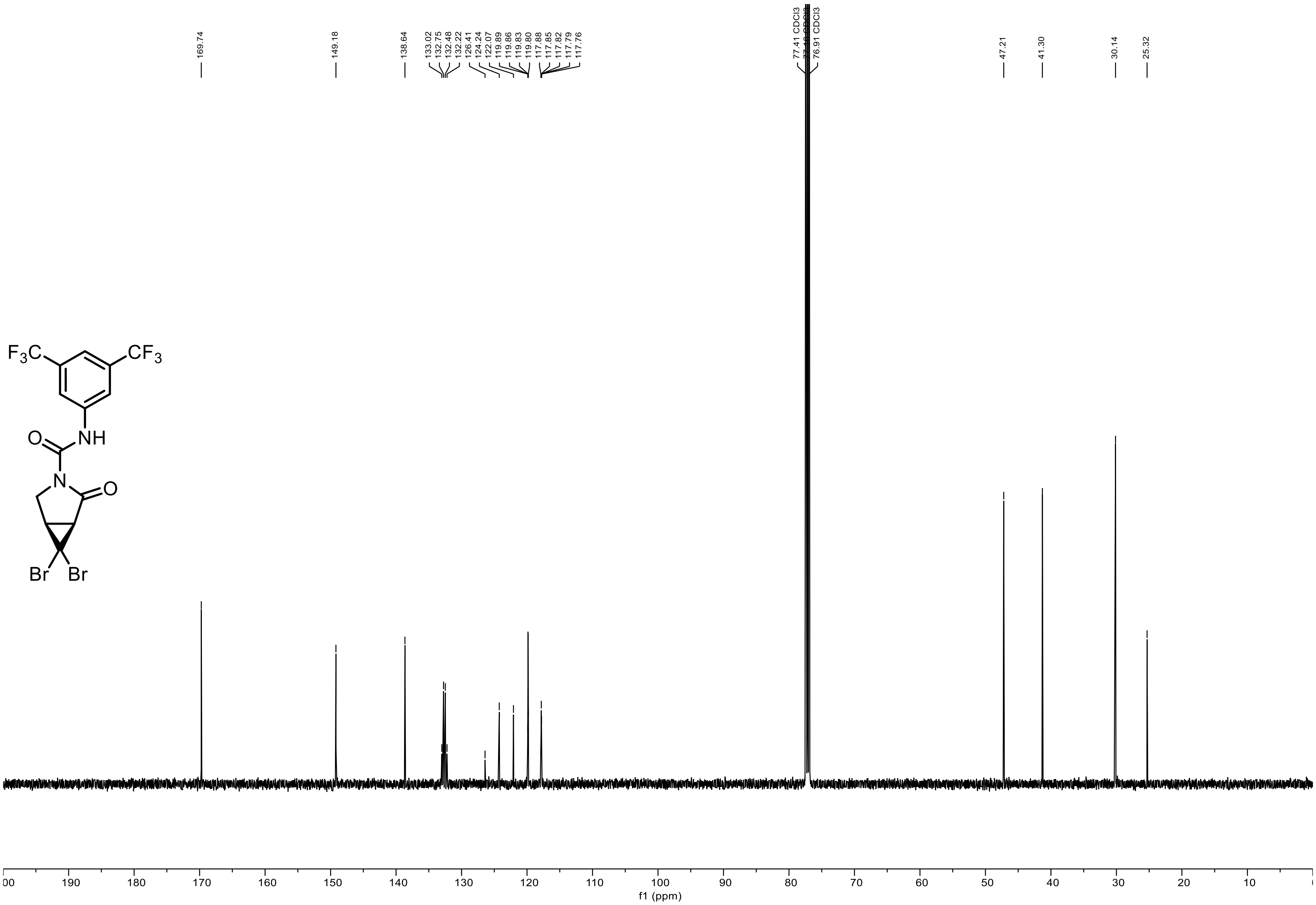

22 ^{13}C NMR (126 MHz, $CDCl_3$).

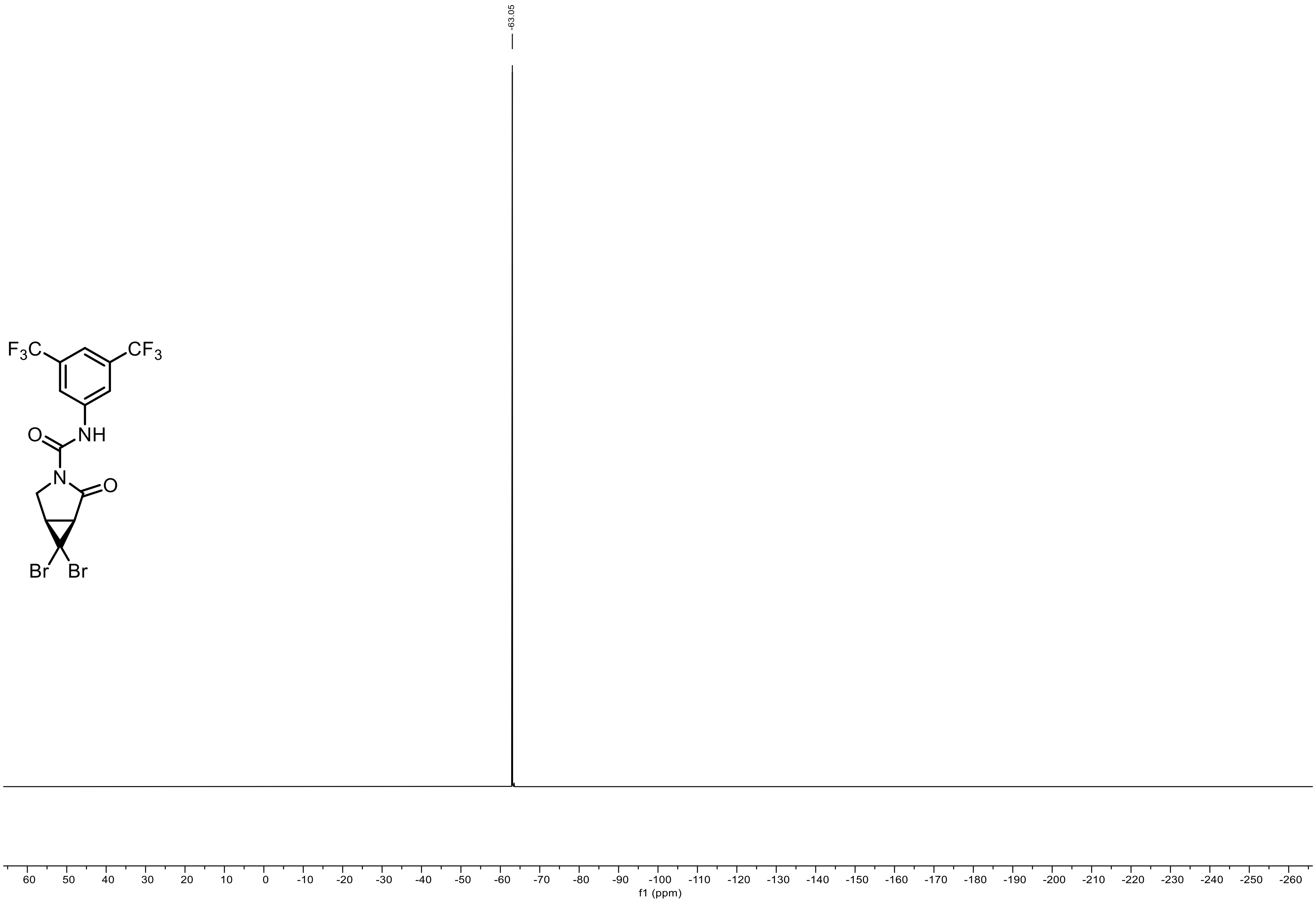

22 ^{19}F NMR (470 MHz, $CDCl_3$).

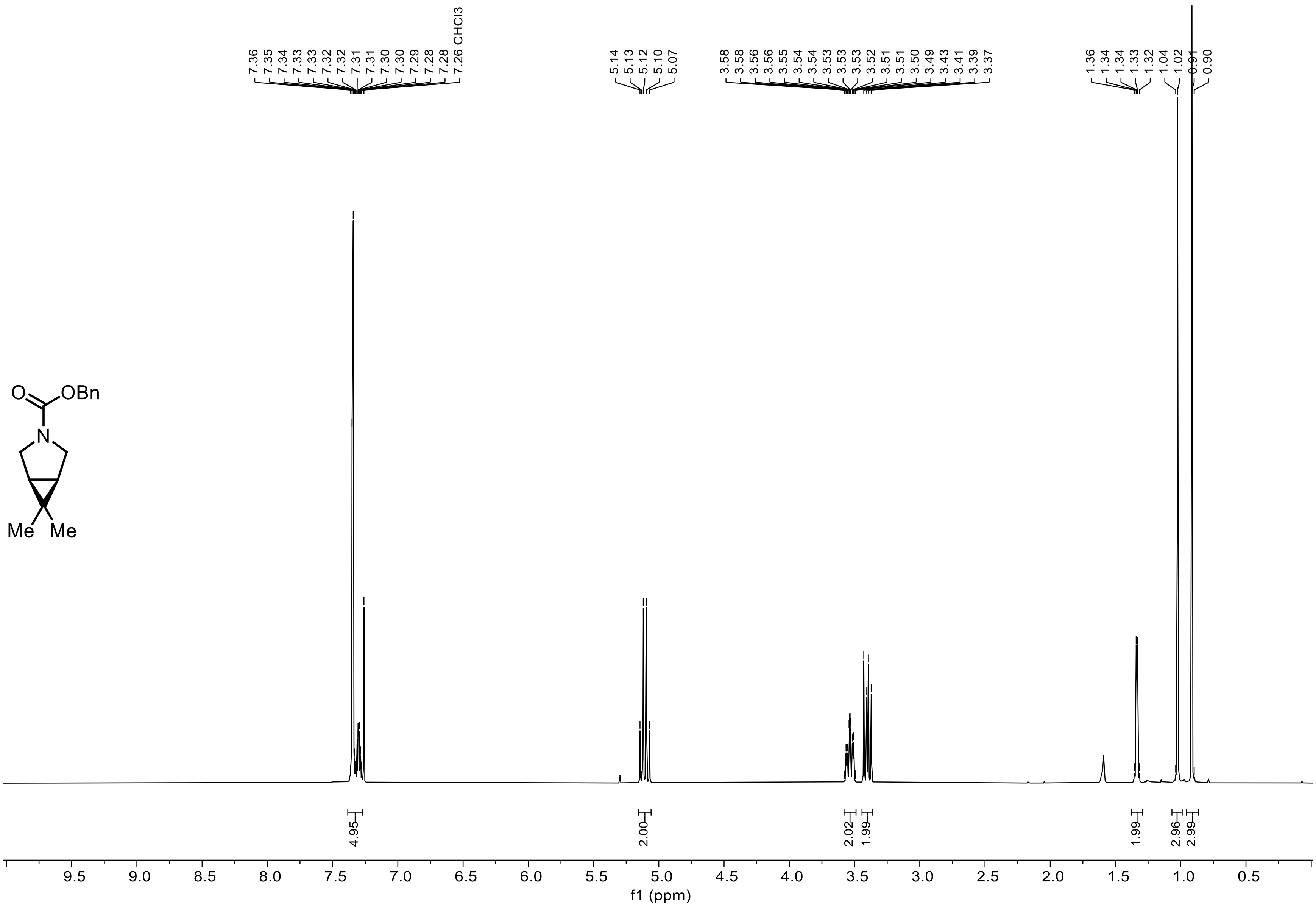

3-OBn ^{1}H NMR (500 MHz, $CDCl_3$).

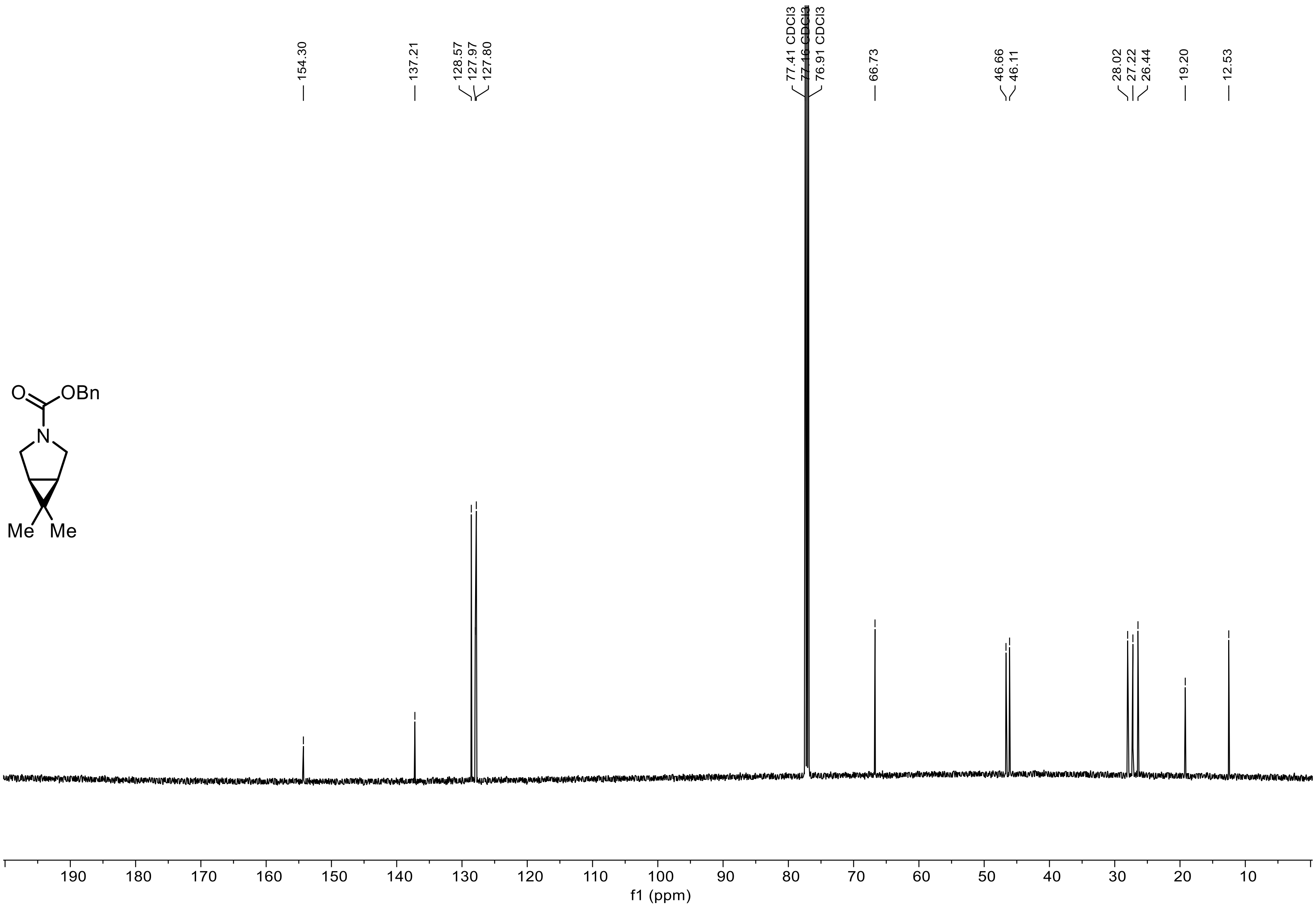

3-OBn ^{13}C NMR (126 MHz, $CDCl_3$).

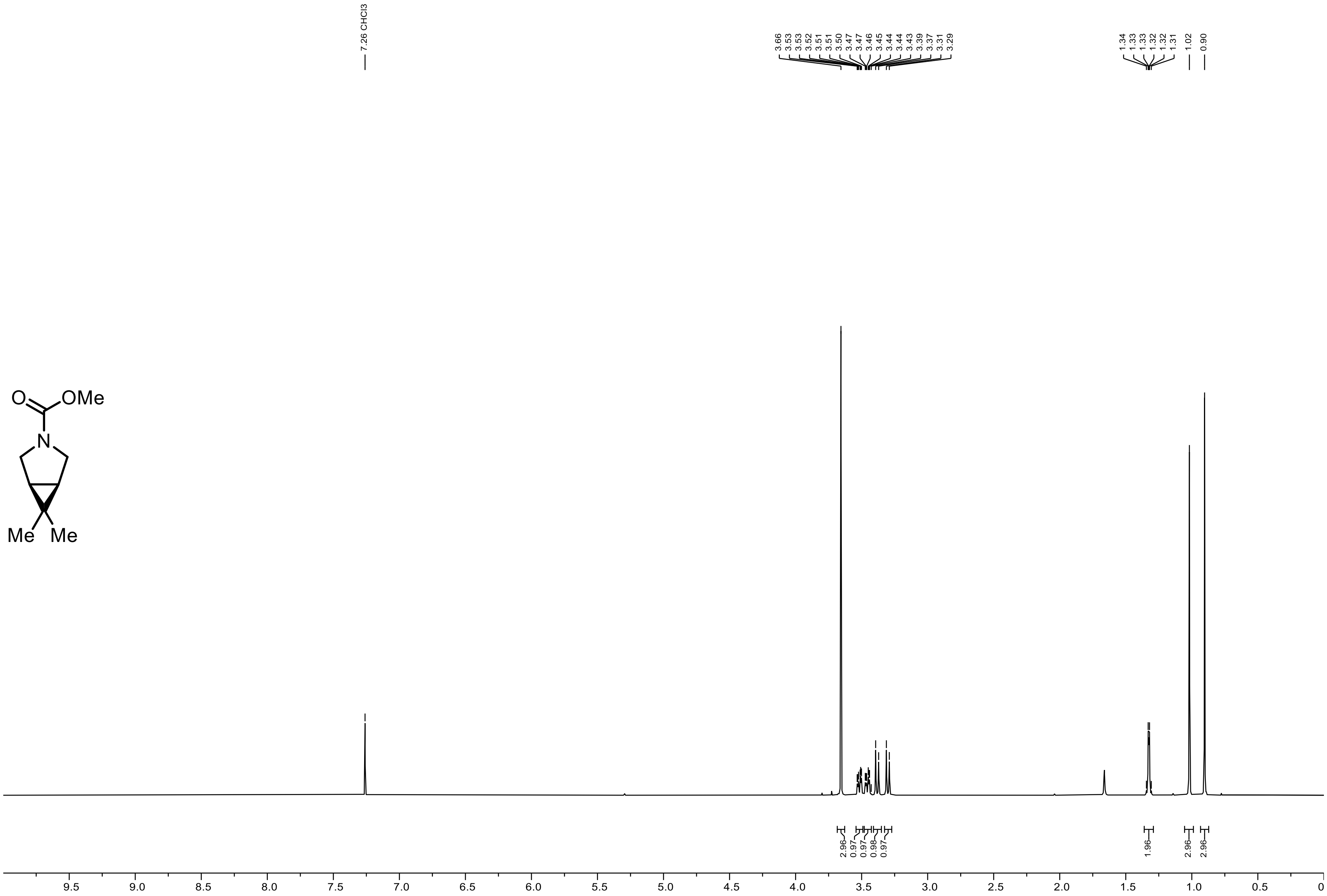

3-OMe ^{1}H NMR (500 MHz, $CDCl_3$).

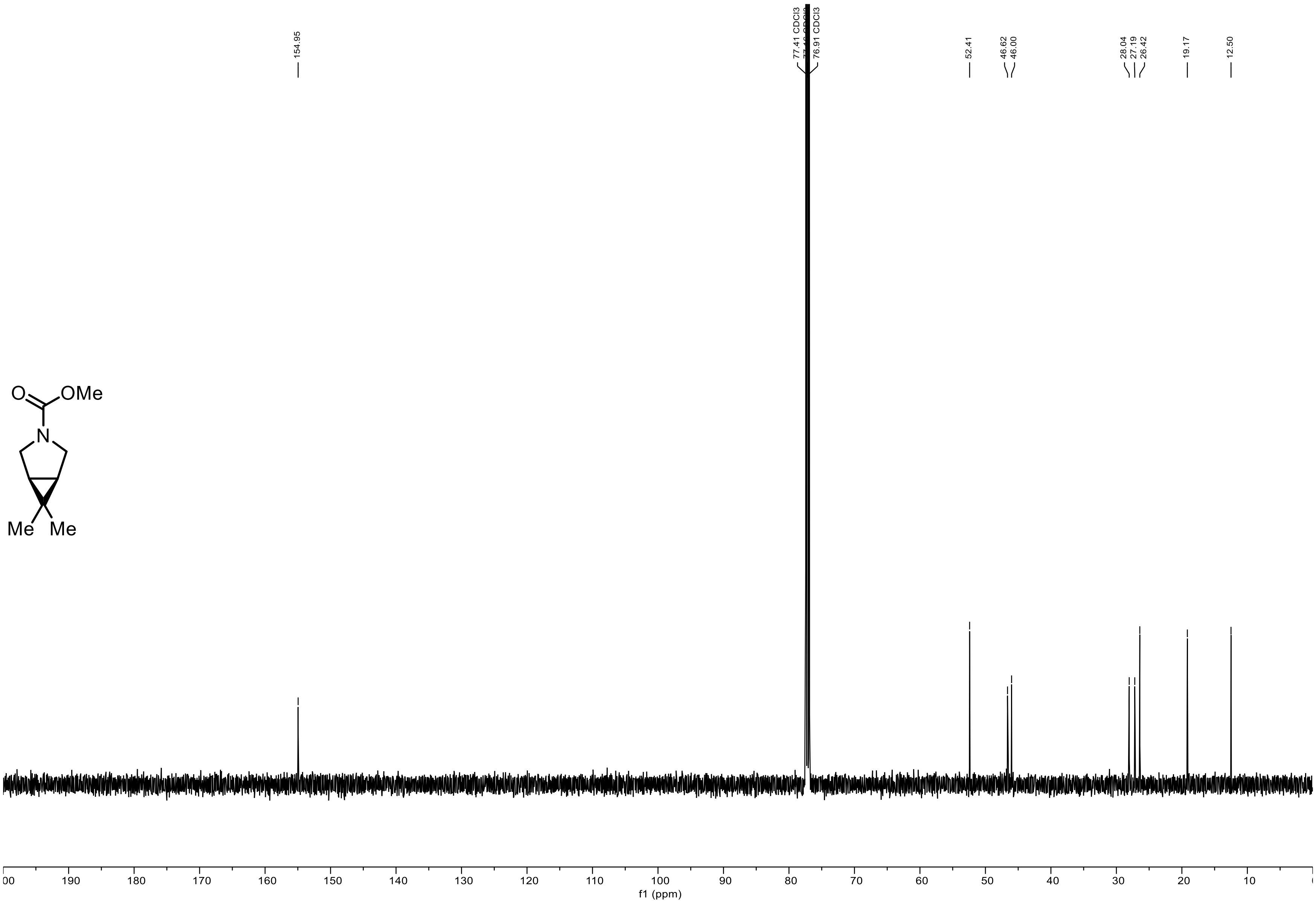

3-OMe ^{13}C NMR (126 MHz, $CDCl_3$).

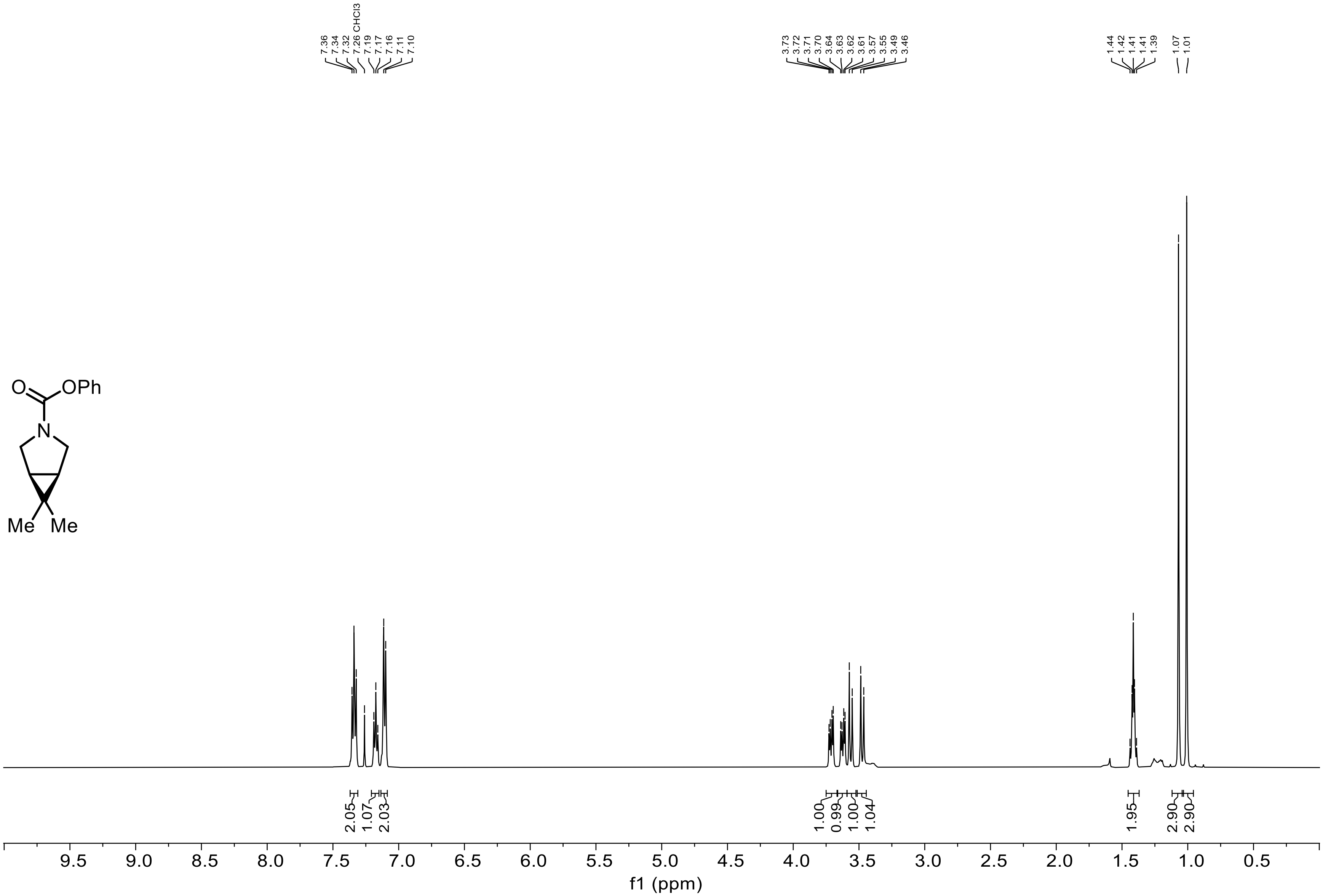

3-OPh ^{1}H NMR (500 MHz, $CDCl_3$).

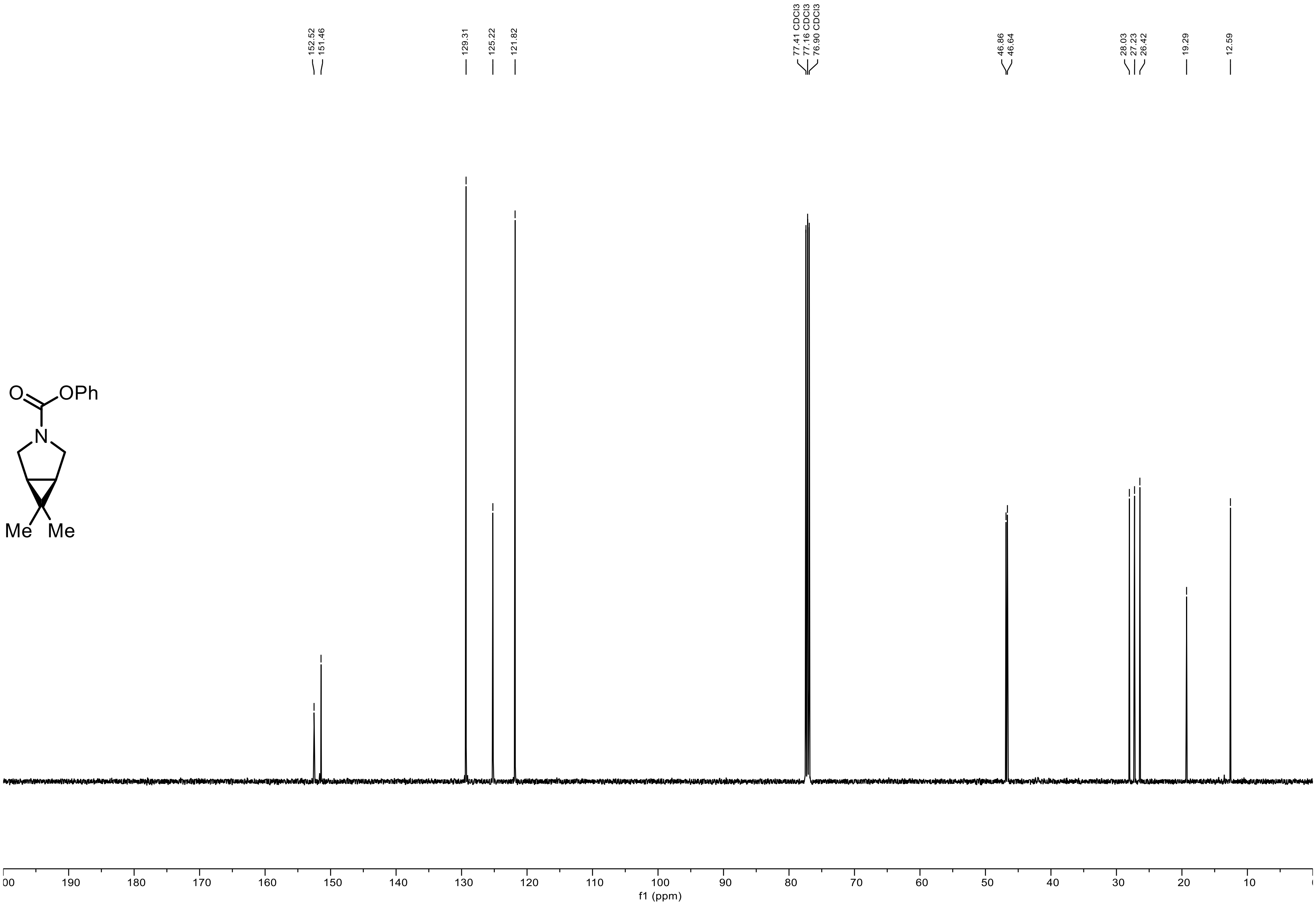

3-OPh ^{13}C NMR (126 MHz, $CDCl_3$).

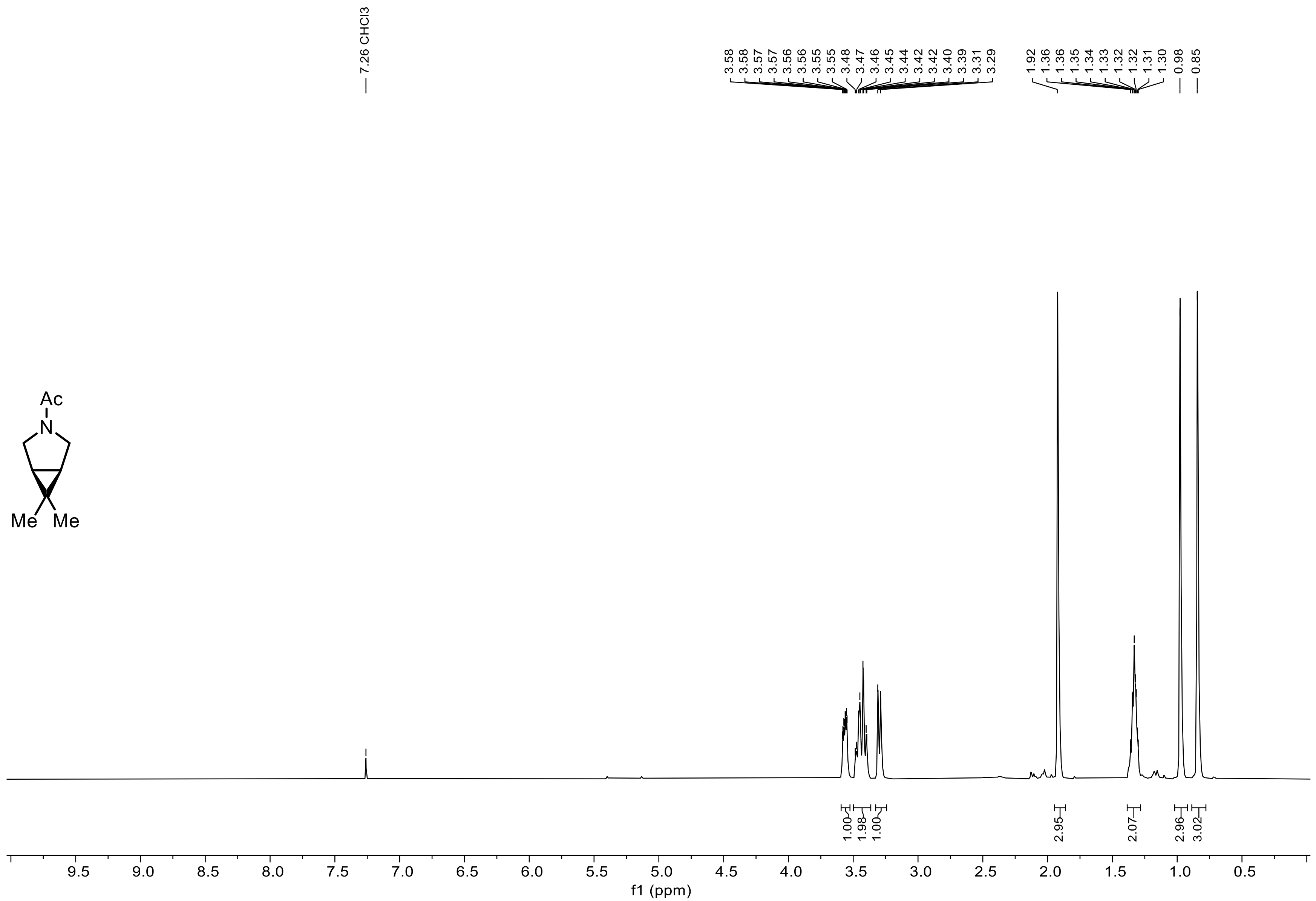

3-Me ^{1}H NMR (500 MHz, $CDCl_3$).

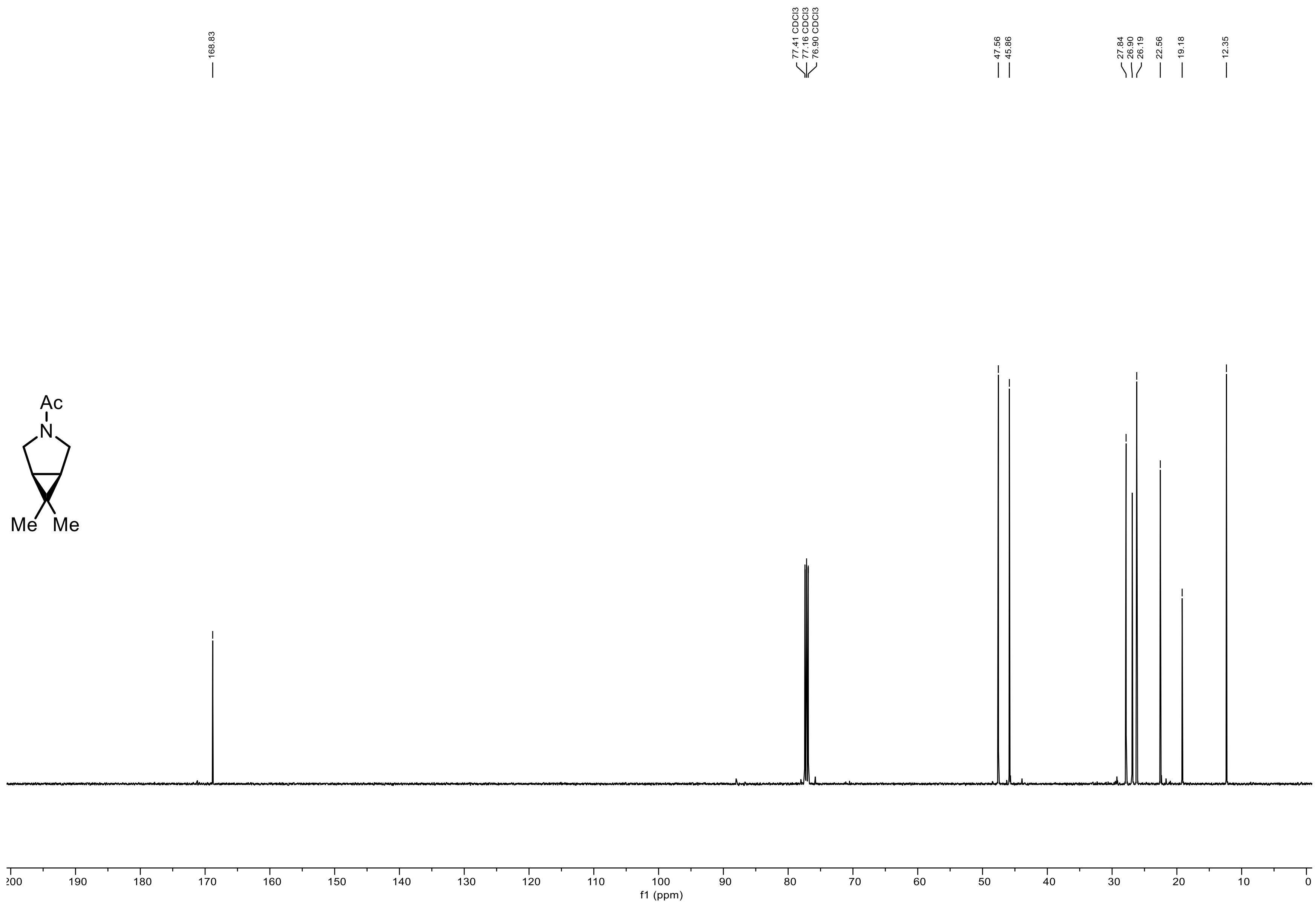

3-Me ^{13}C NMR (126 MHz, $CDCl_3$).

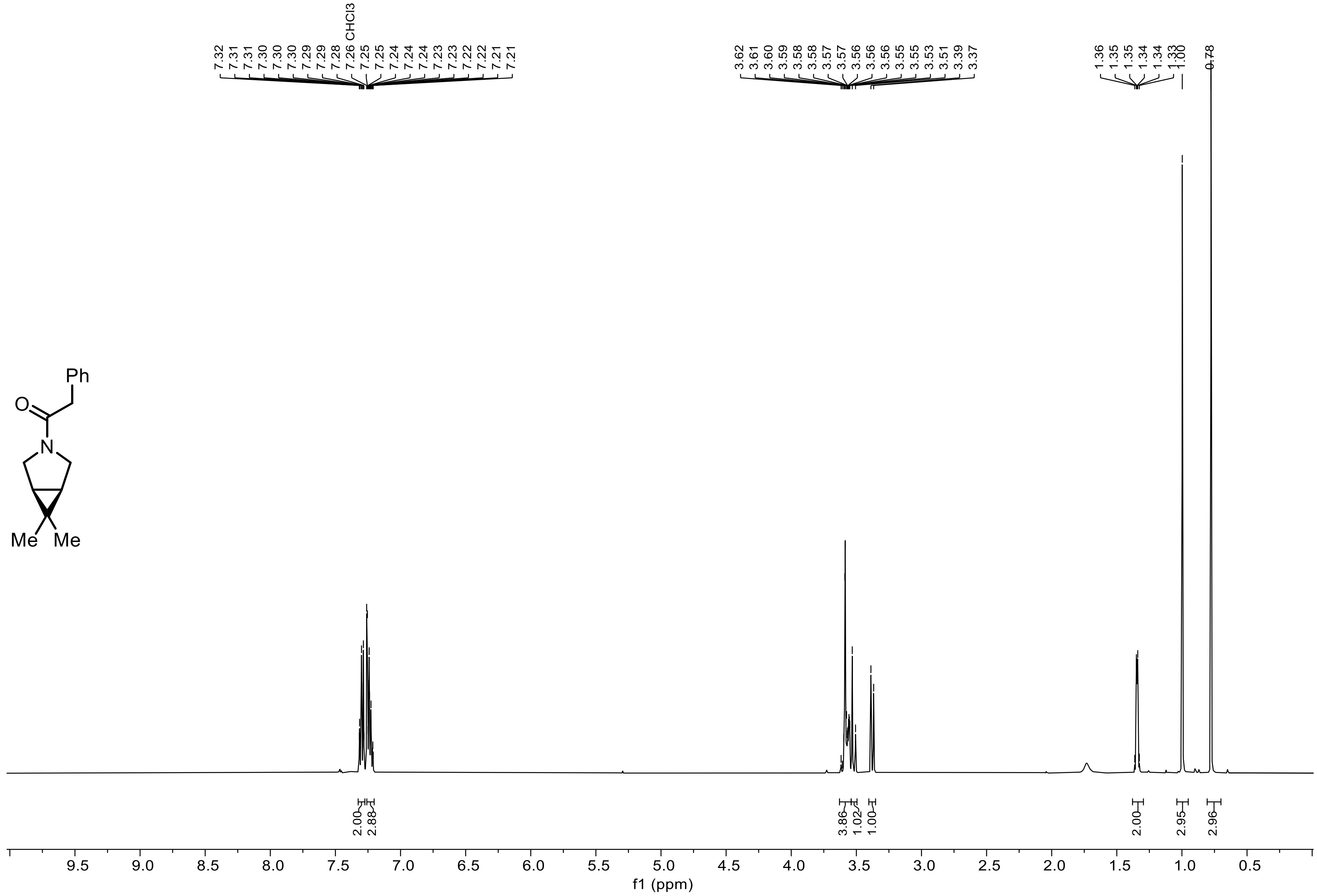

3-Bn ^{1}H NMR (500 MHz, $CDCl_3$).

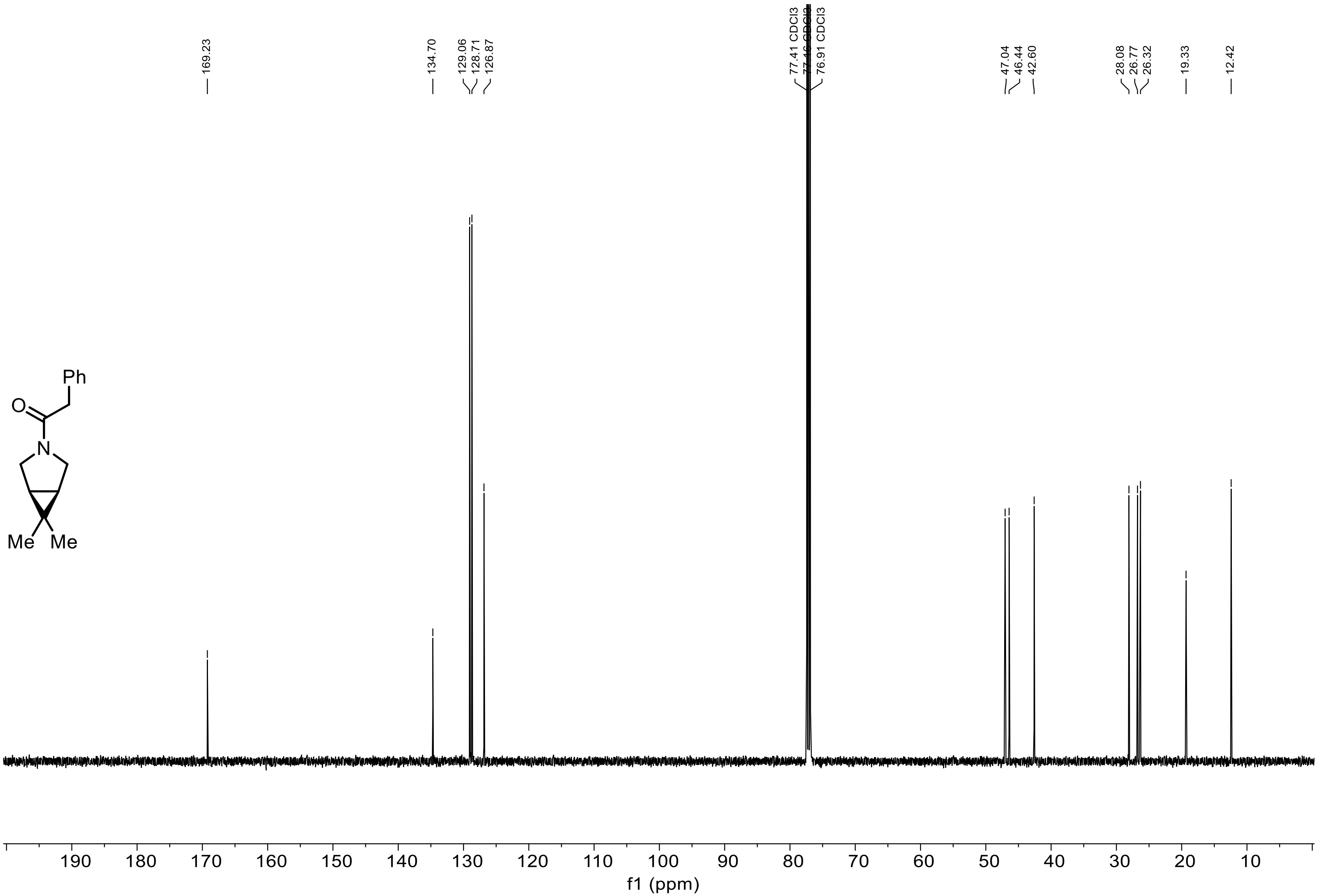

3-Bn ^{13}C NMR (126 MHz, $CDCl_3$).

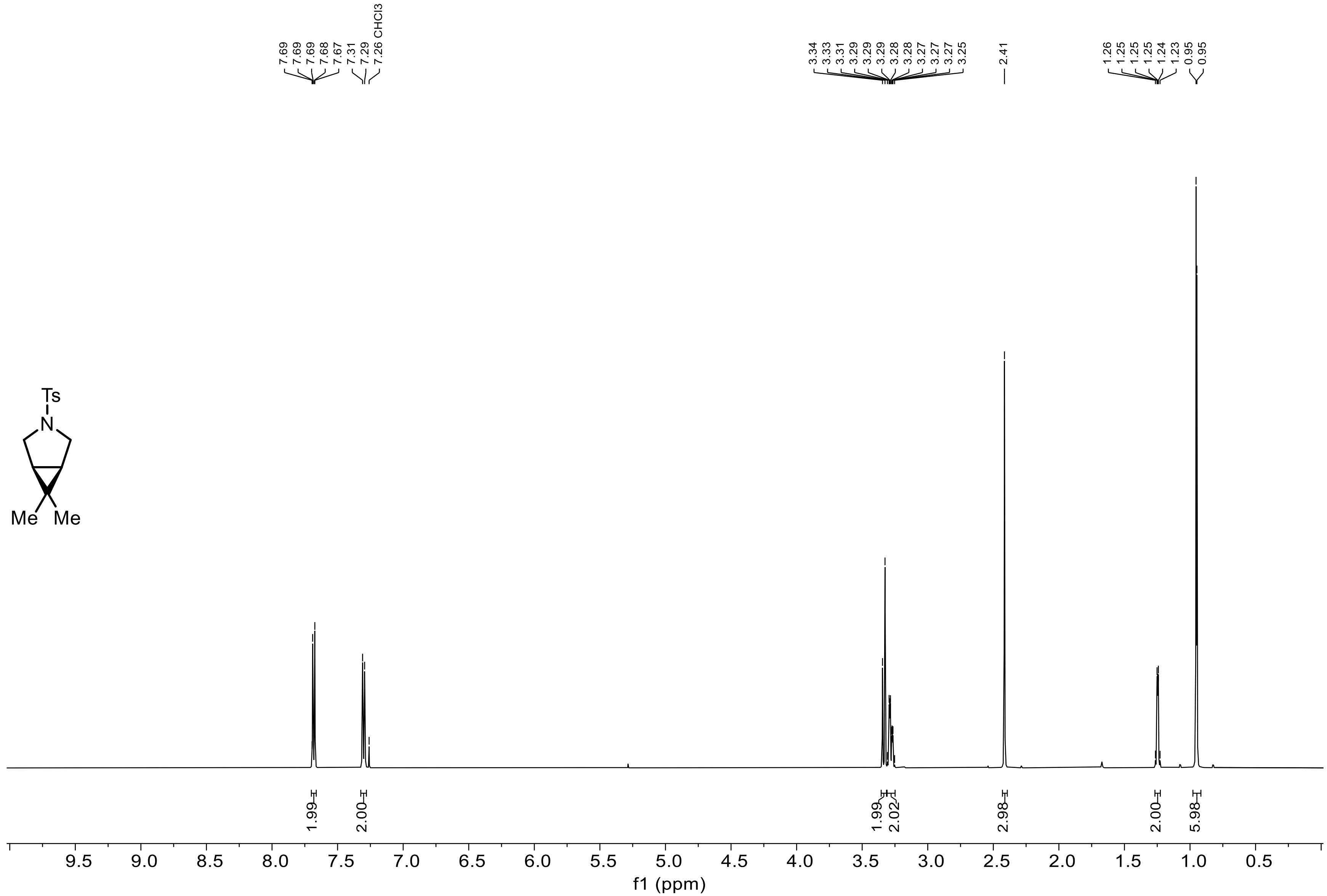

3-Ts ^{1}H NMR (500 MHz, $CDCl_3$).

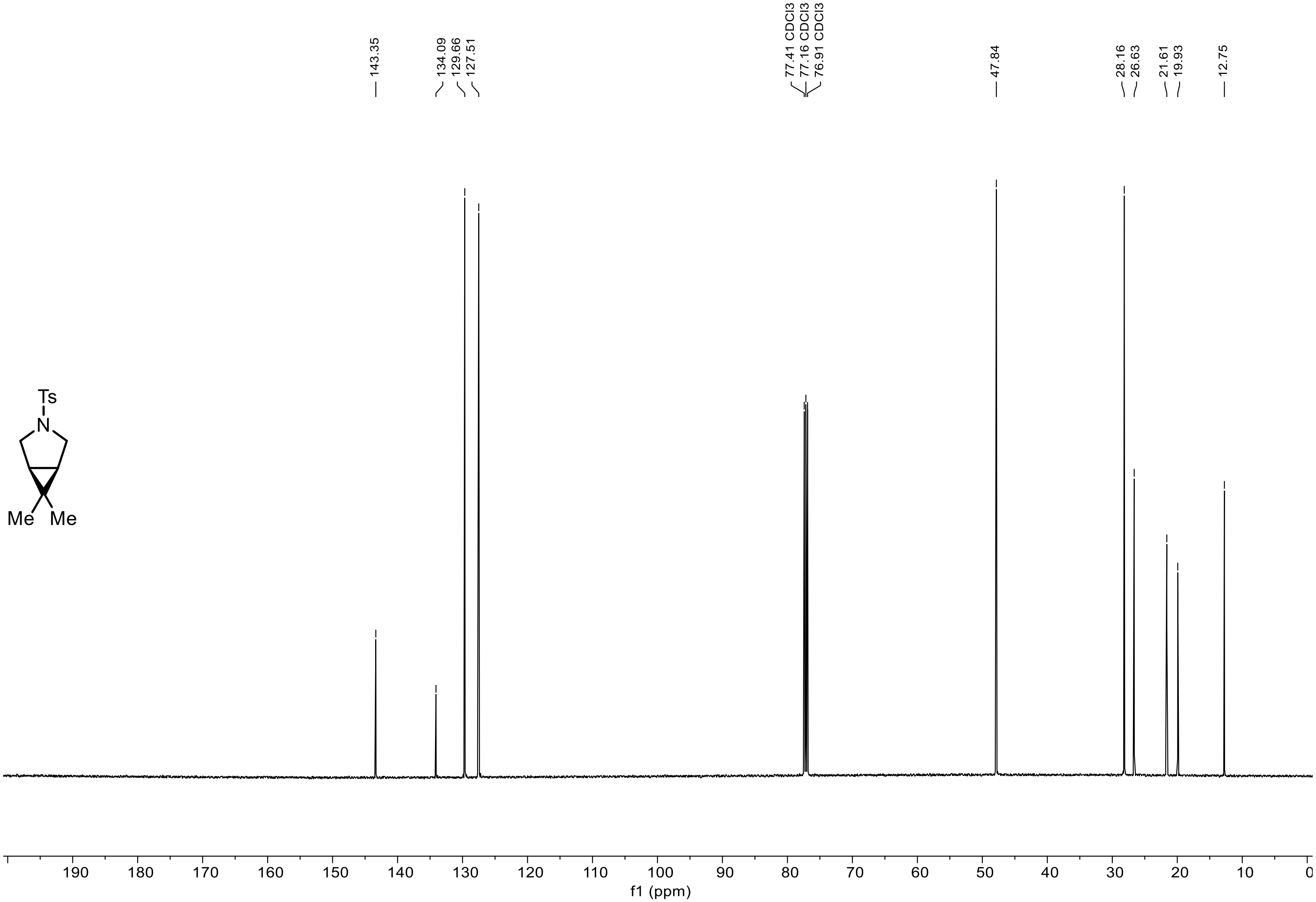

3-Ts ^{13}C NMR (126 MHz, $CDCl_3$).

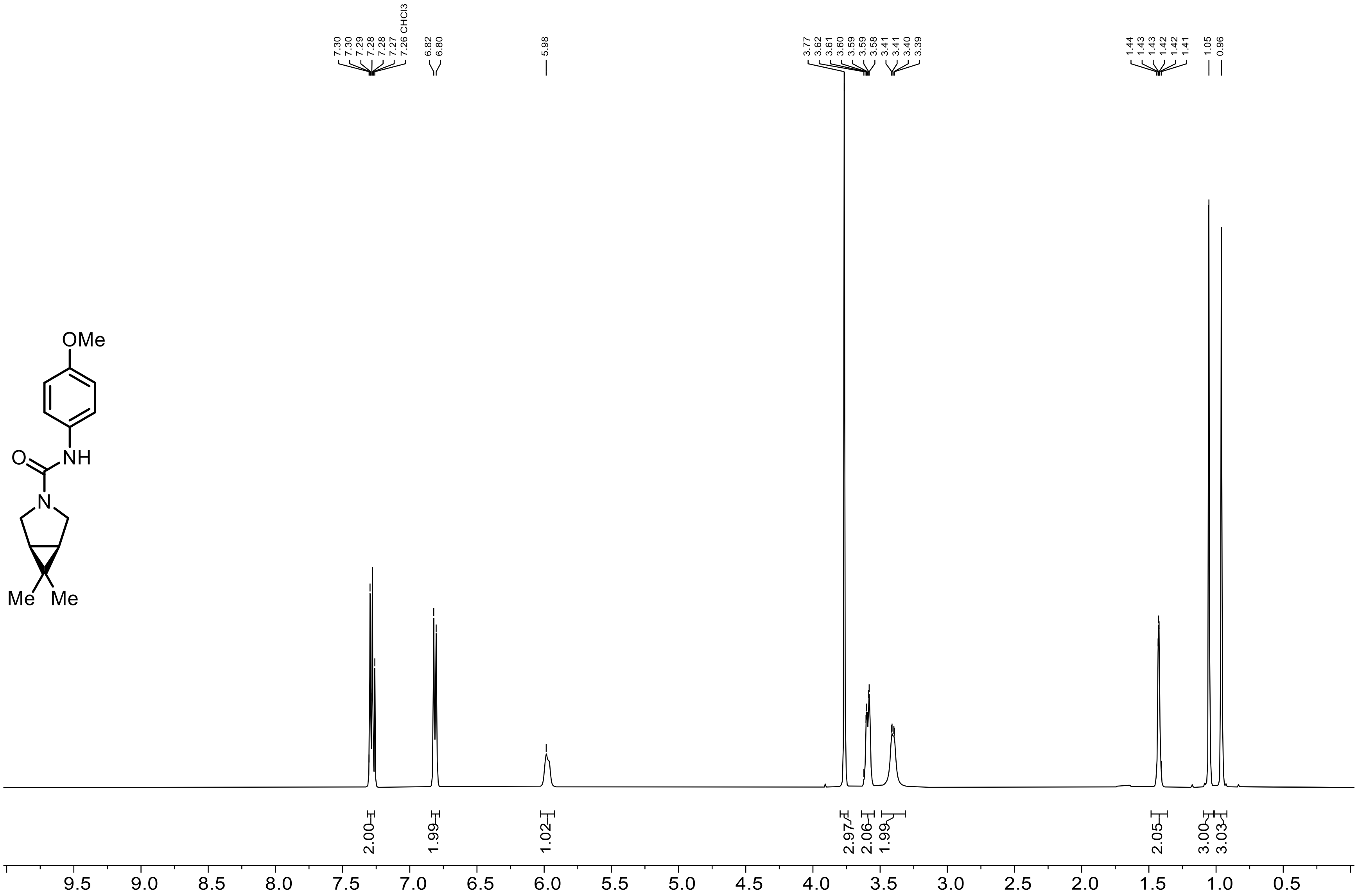

4-OMe ^{1}H NMR (500 MHz, $CDCl_3$).

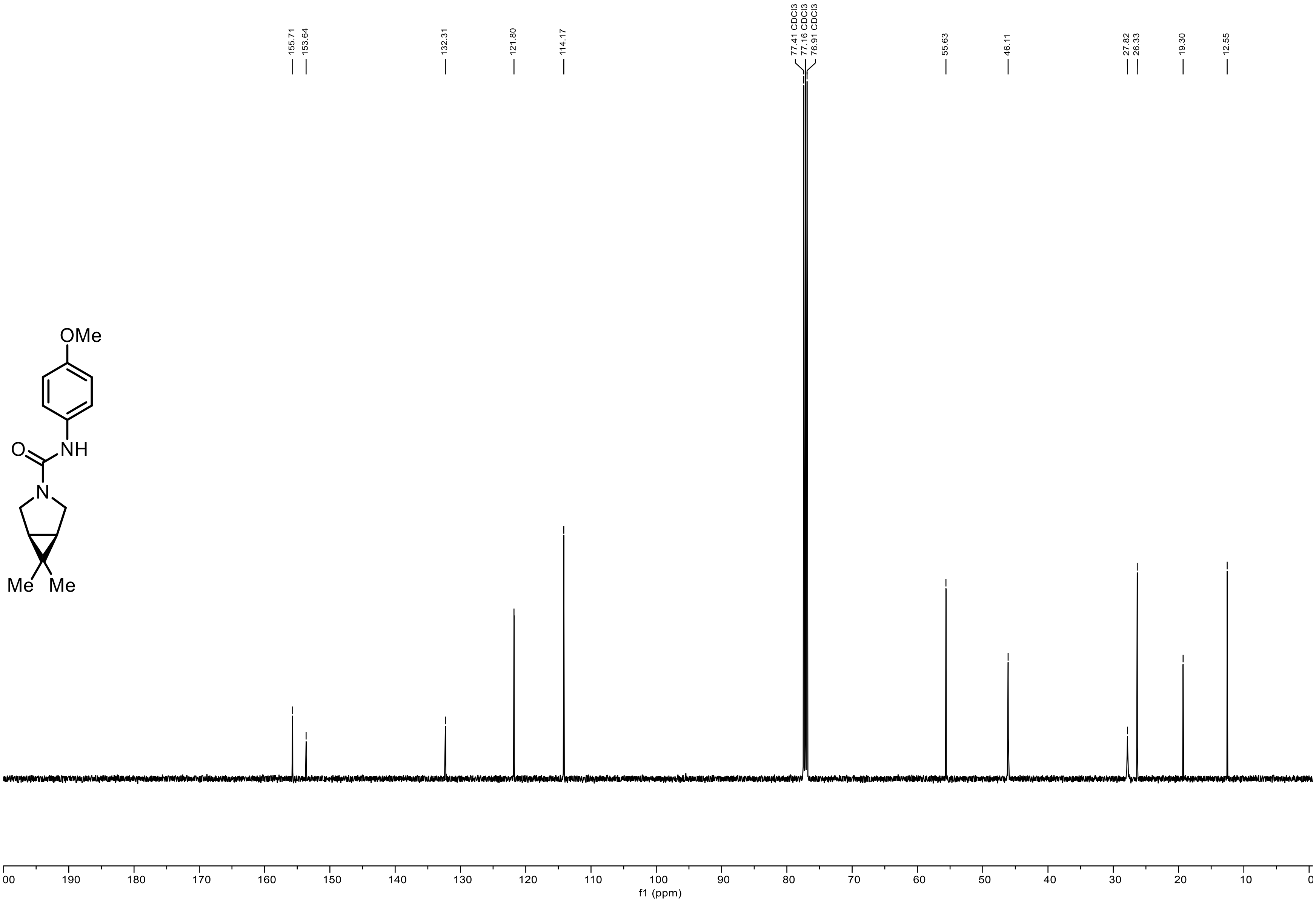

4-OMe ^{13}C NMR (126 MHz, $CDCl_3$).

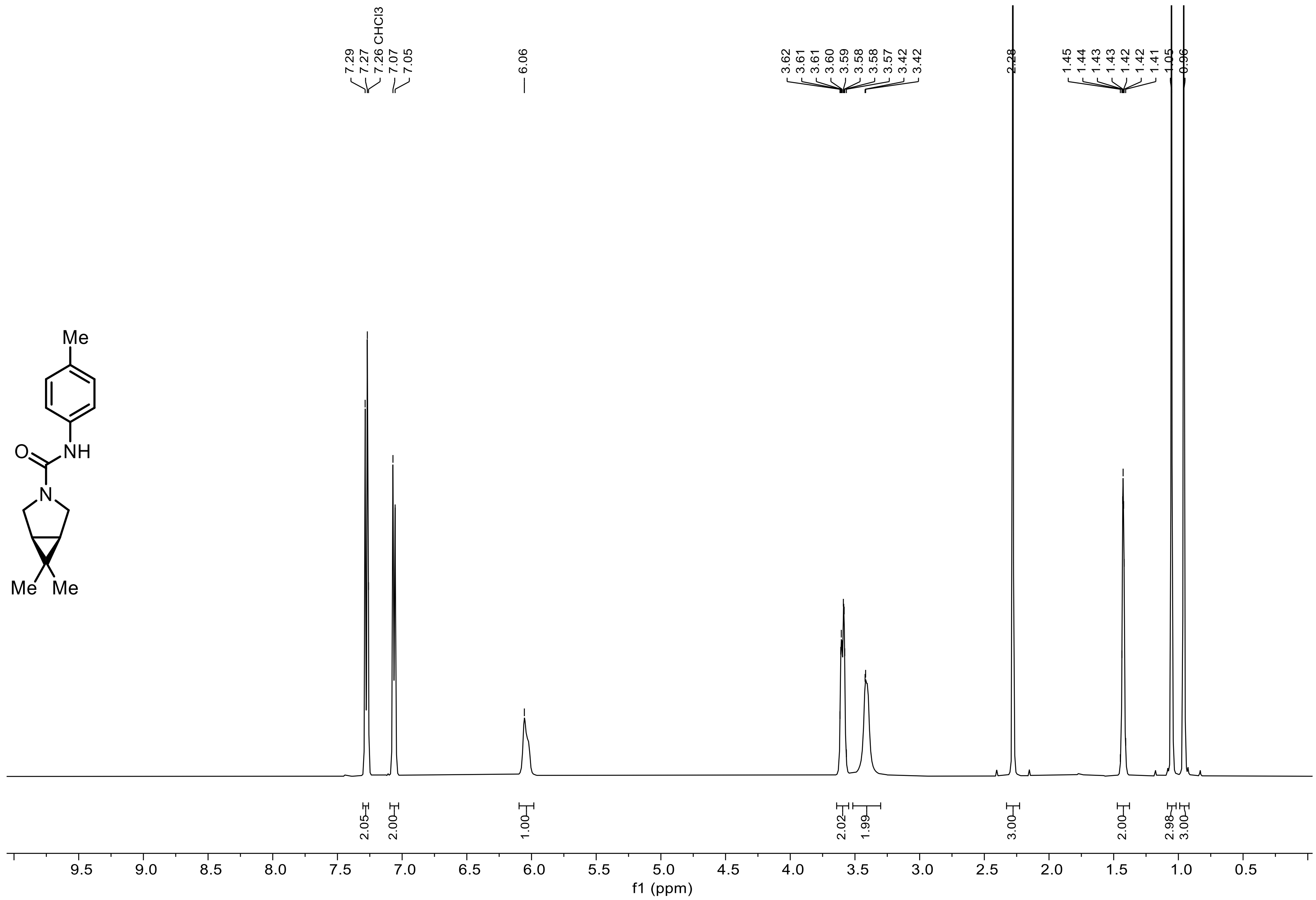

4-Me ^{1}H NMR (500 MHz, $CDCl_3$).

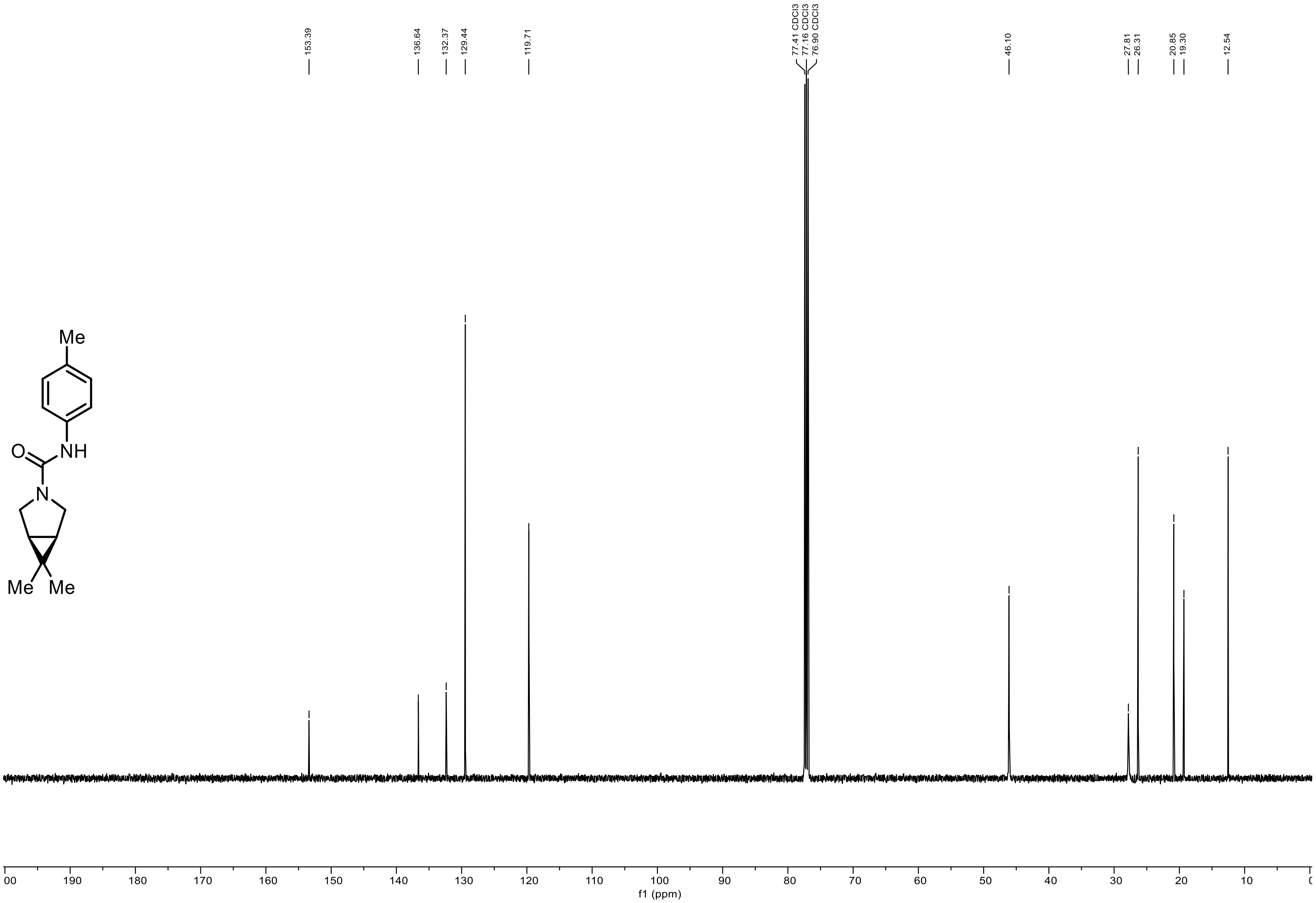

4-Me ^{13}C NMR (126 MHz, $CDCl_3$).

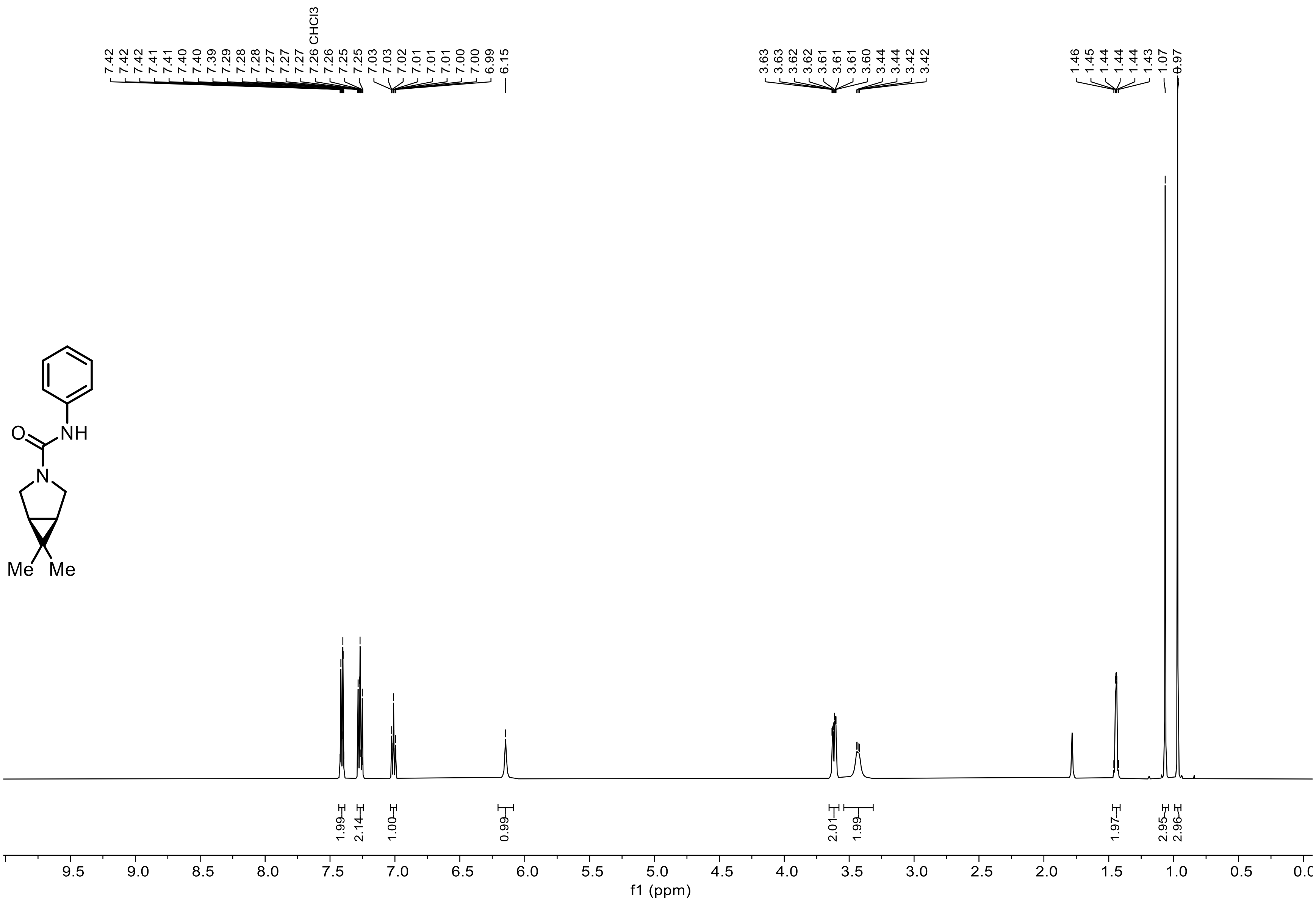

4-H ^{1}H NMR (500 MHz, $CDCl_3$).

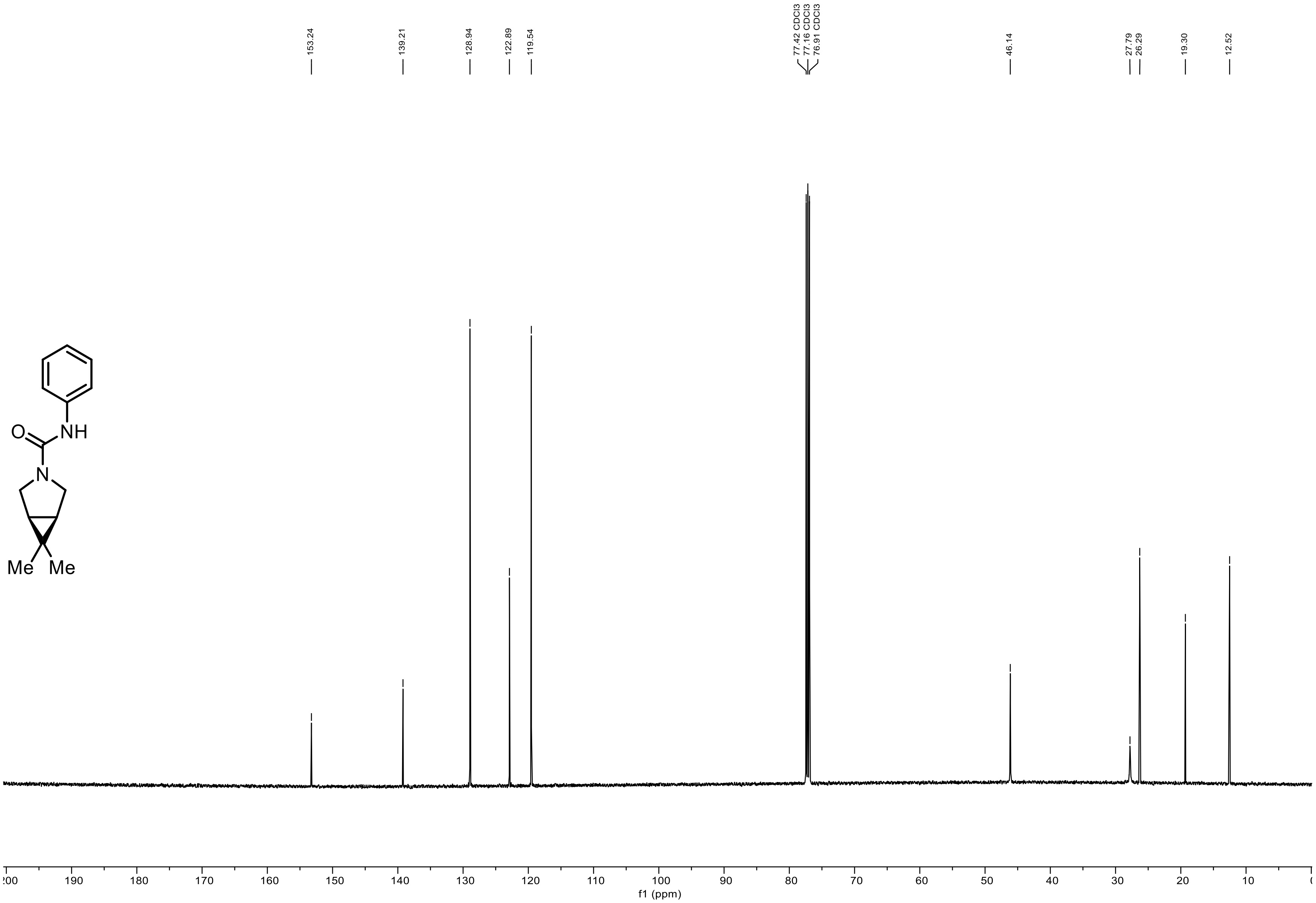

4-H ^{13}C NMR (126 MHz, $CDCl_3$).

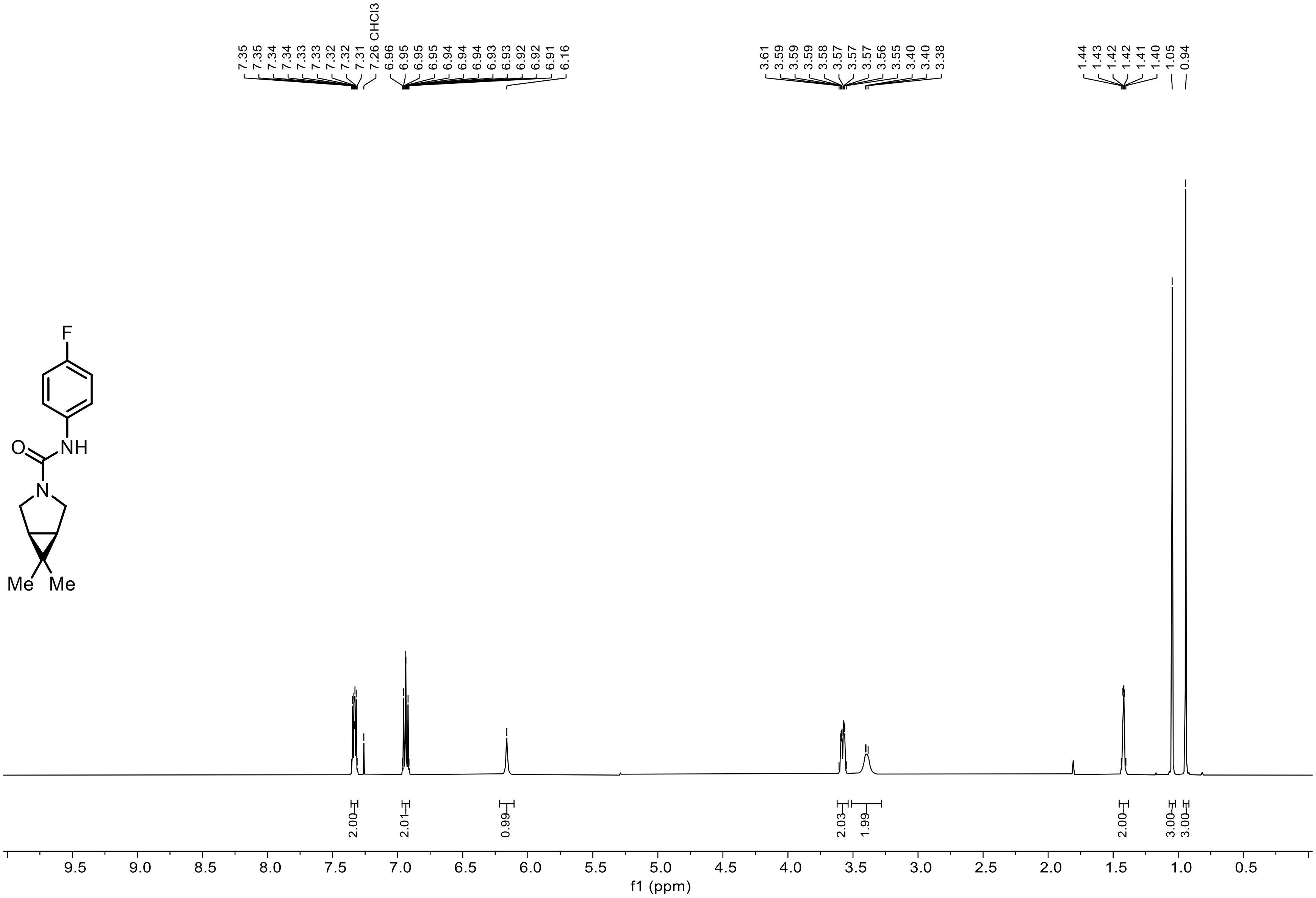

4-F ^{1}H NMR (500 MHz, $CDCl_3$).

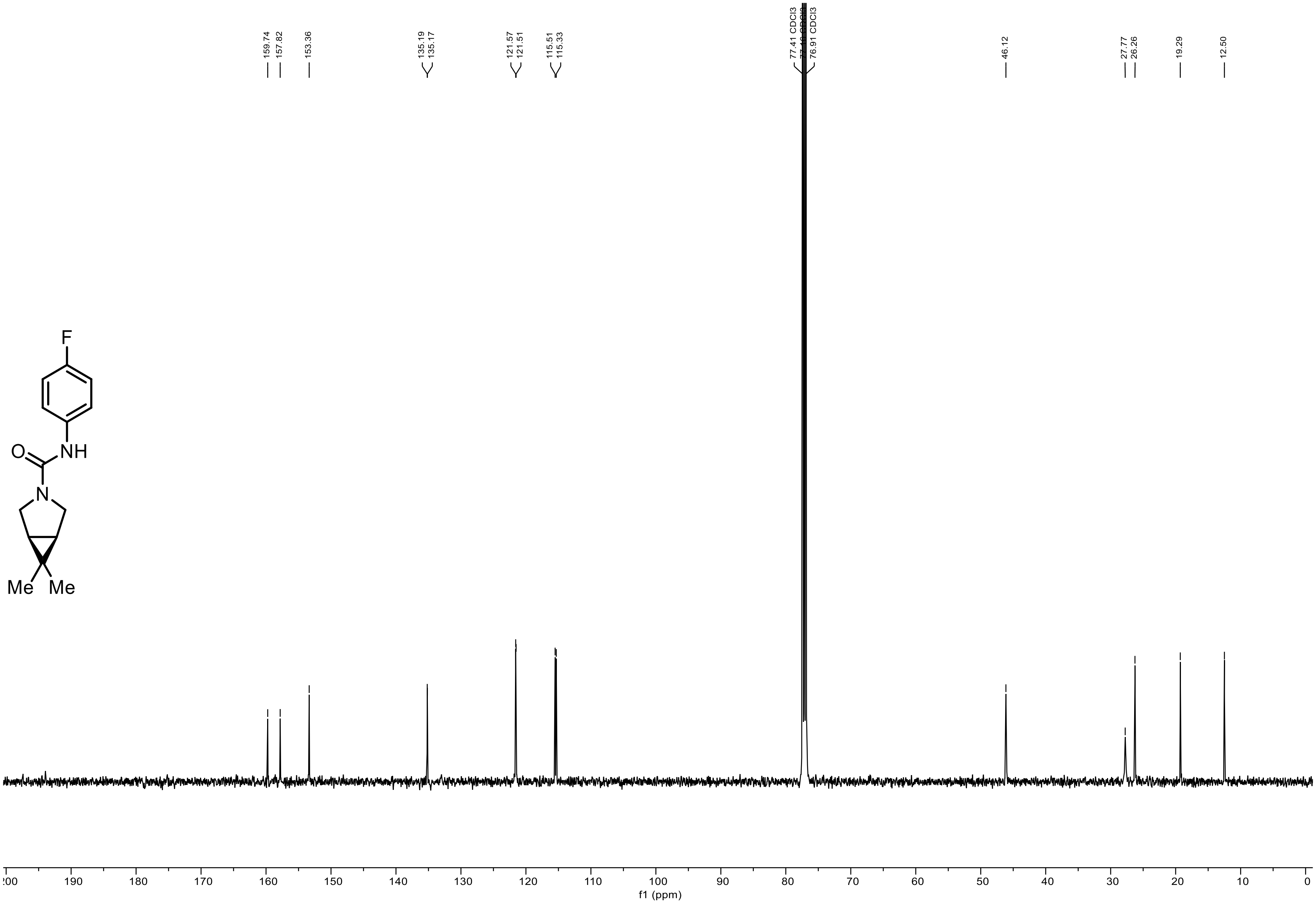

4-F ^{13}C NMR (126 MHz, $CDCl_3$).

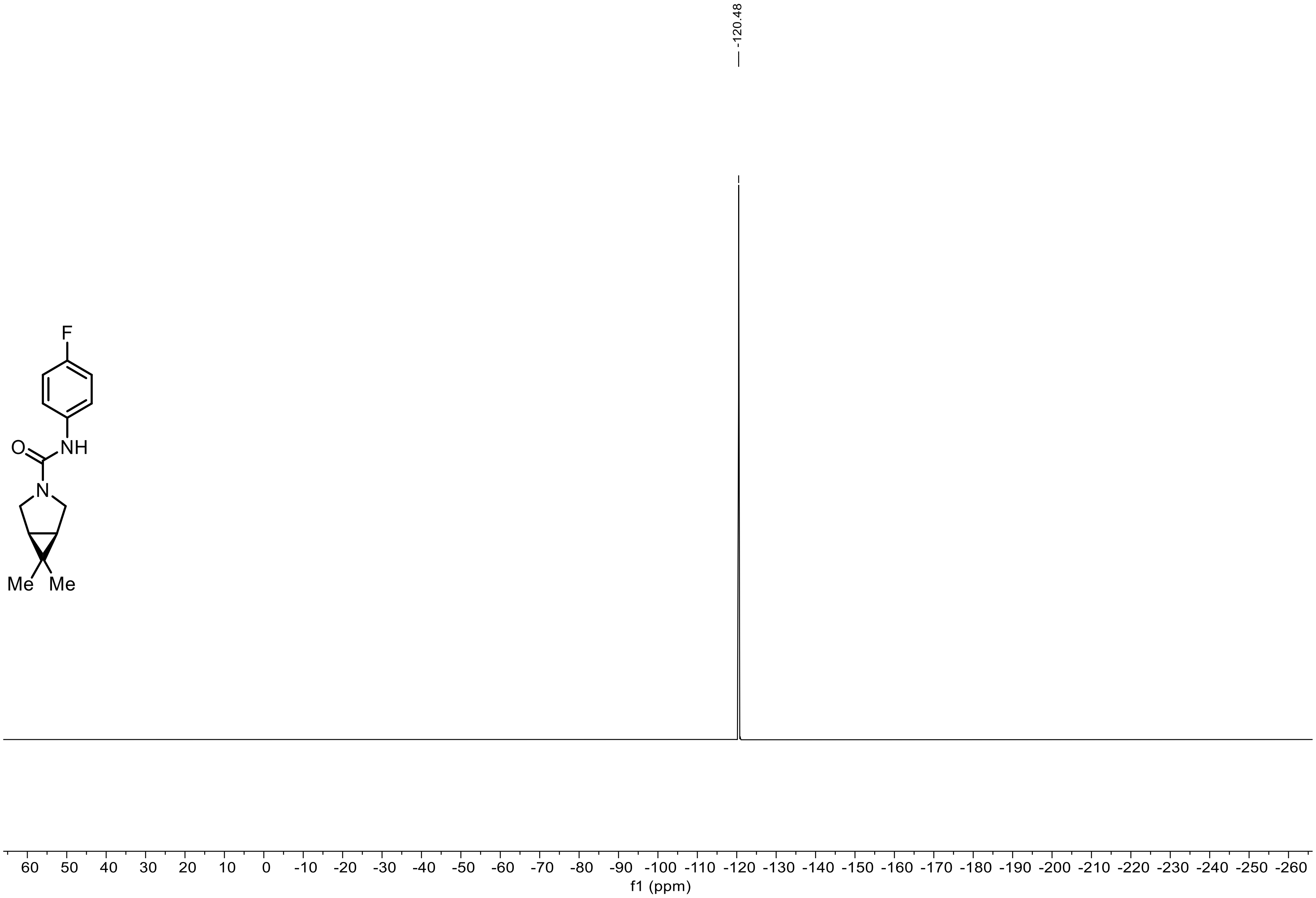

4-F ^{19}F NMR (470 MHz, $CDCl_3$).

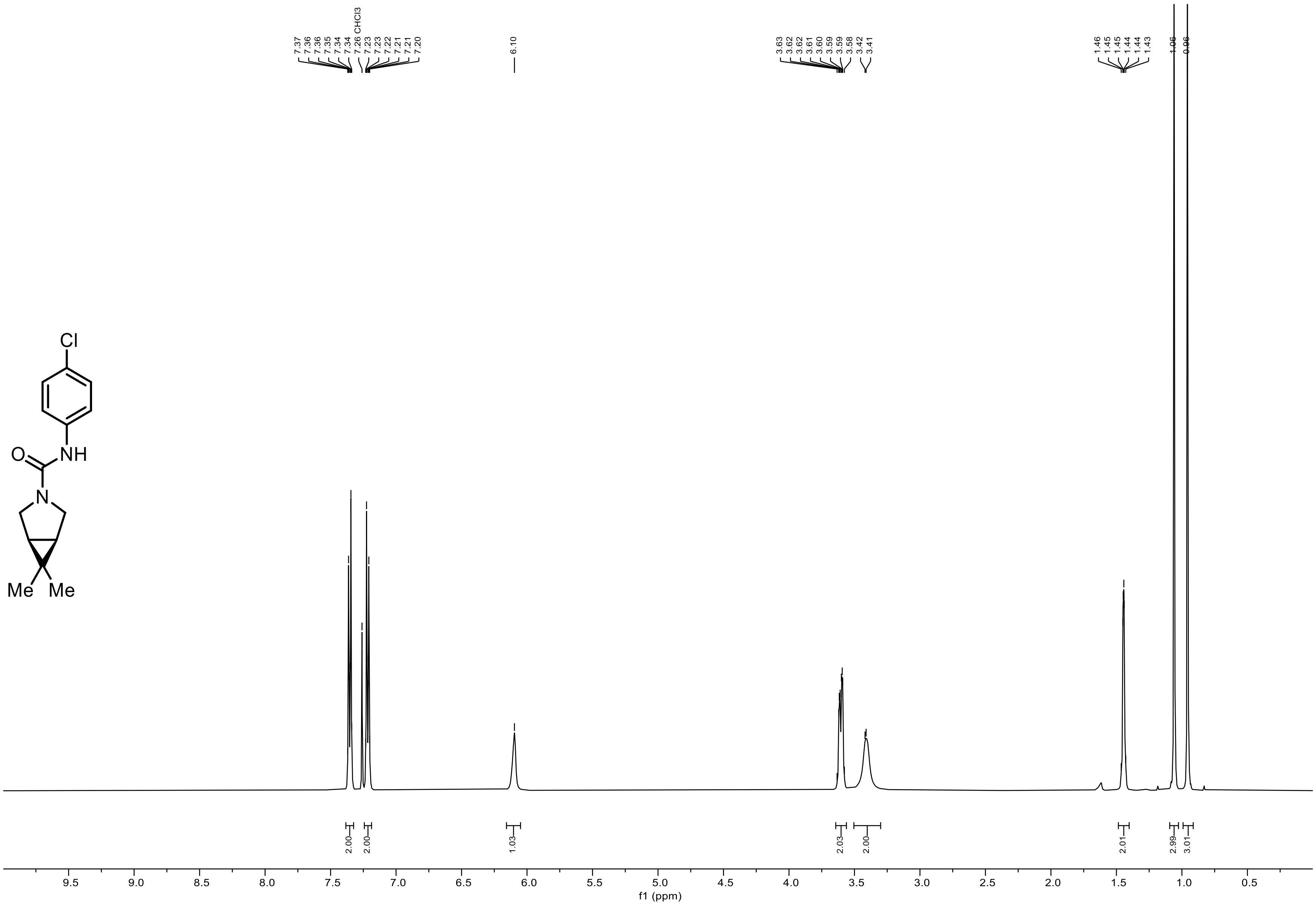

4-Cl ^{1}H NMR (500 MHz, $CDCl_3$).

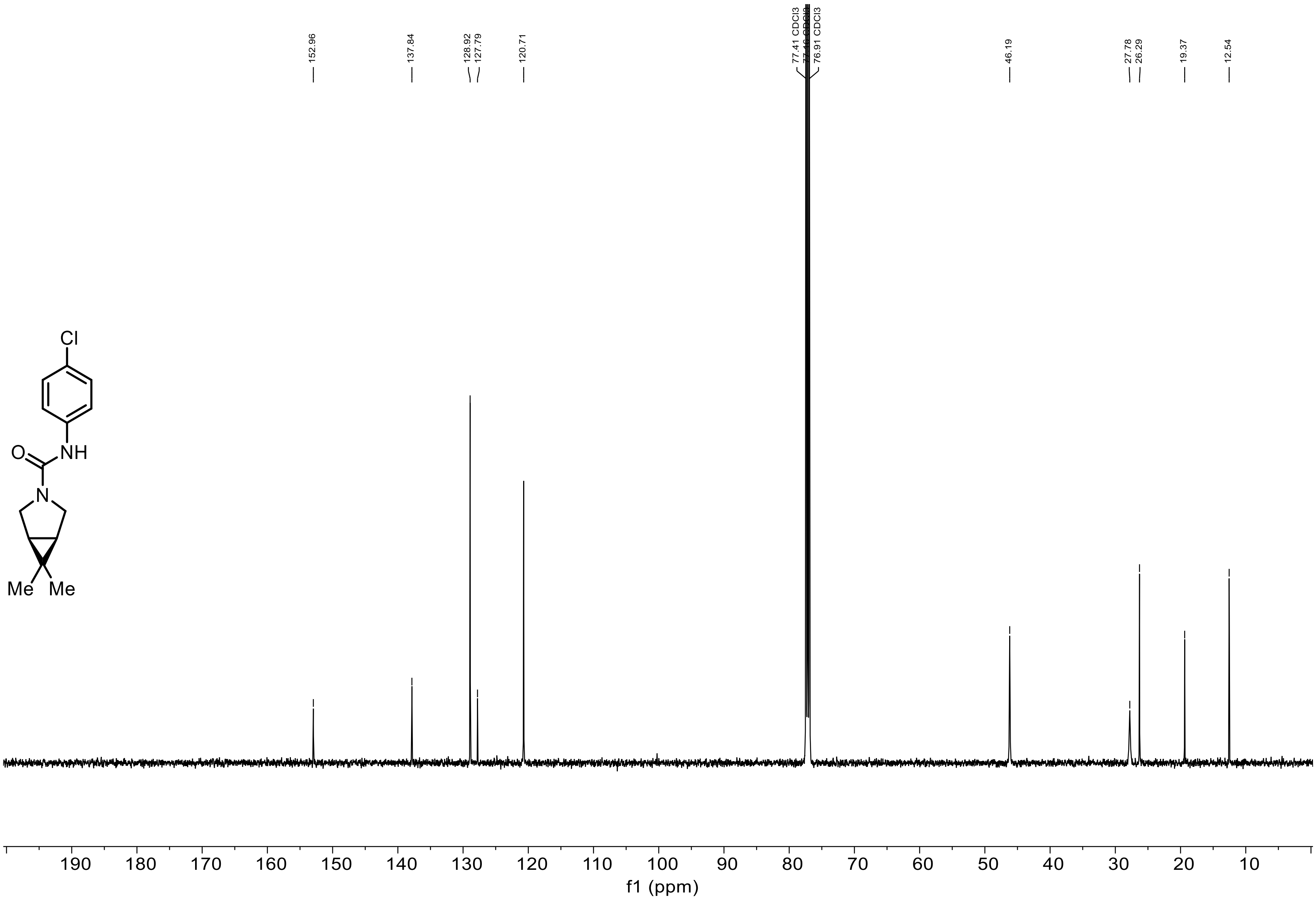

4-Cl ^{13}C NMR (126 MHz, $CDCl_3$).

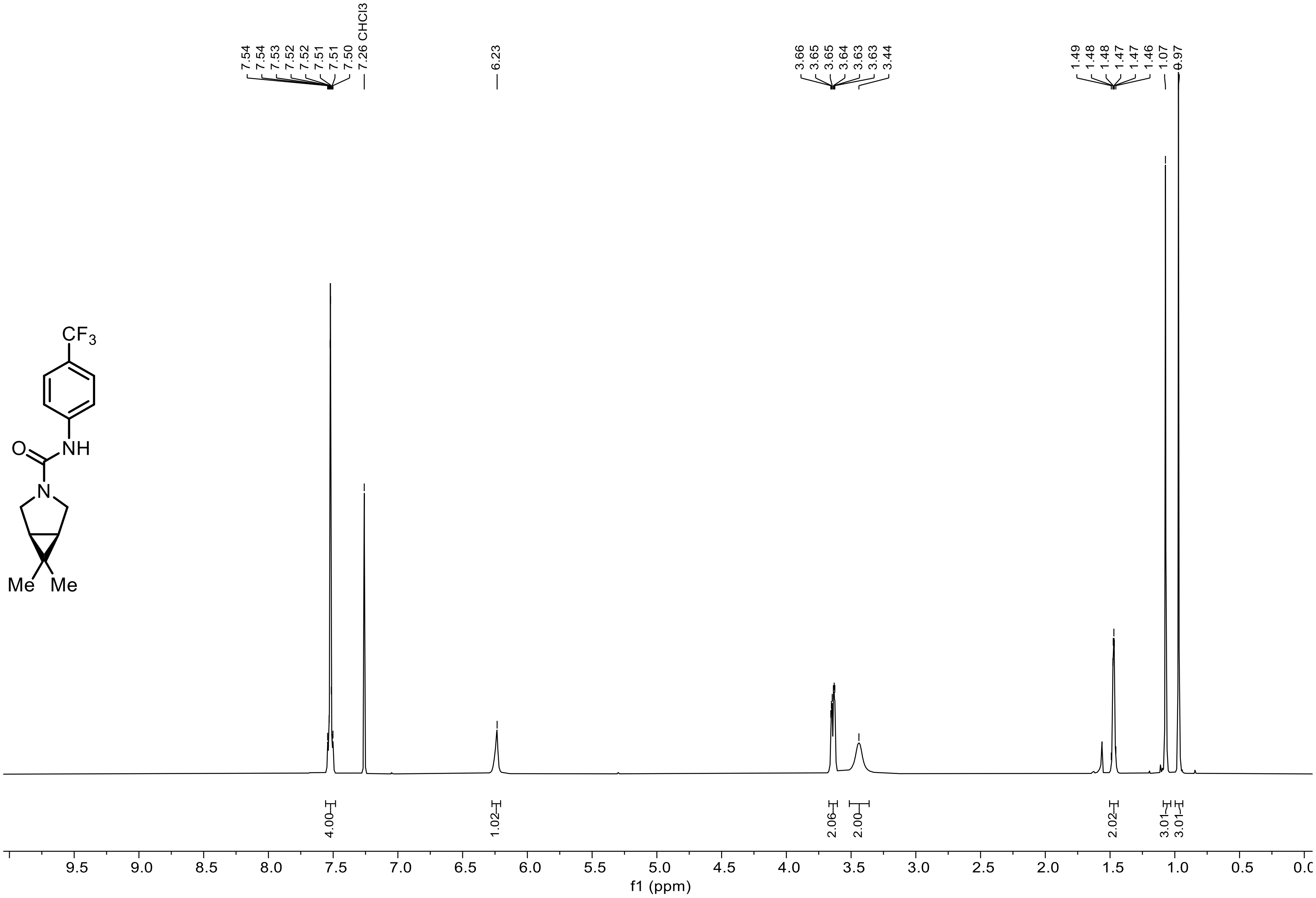

4-CF$_3$ ^{1}H NMR (500 MHz, $CDCl_3$).

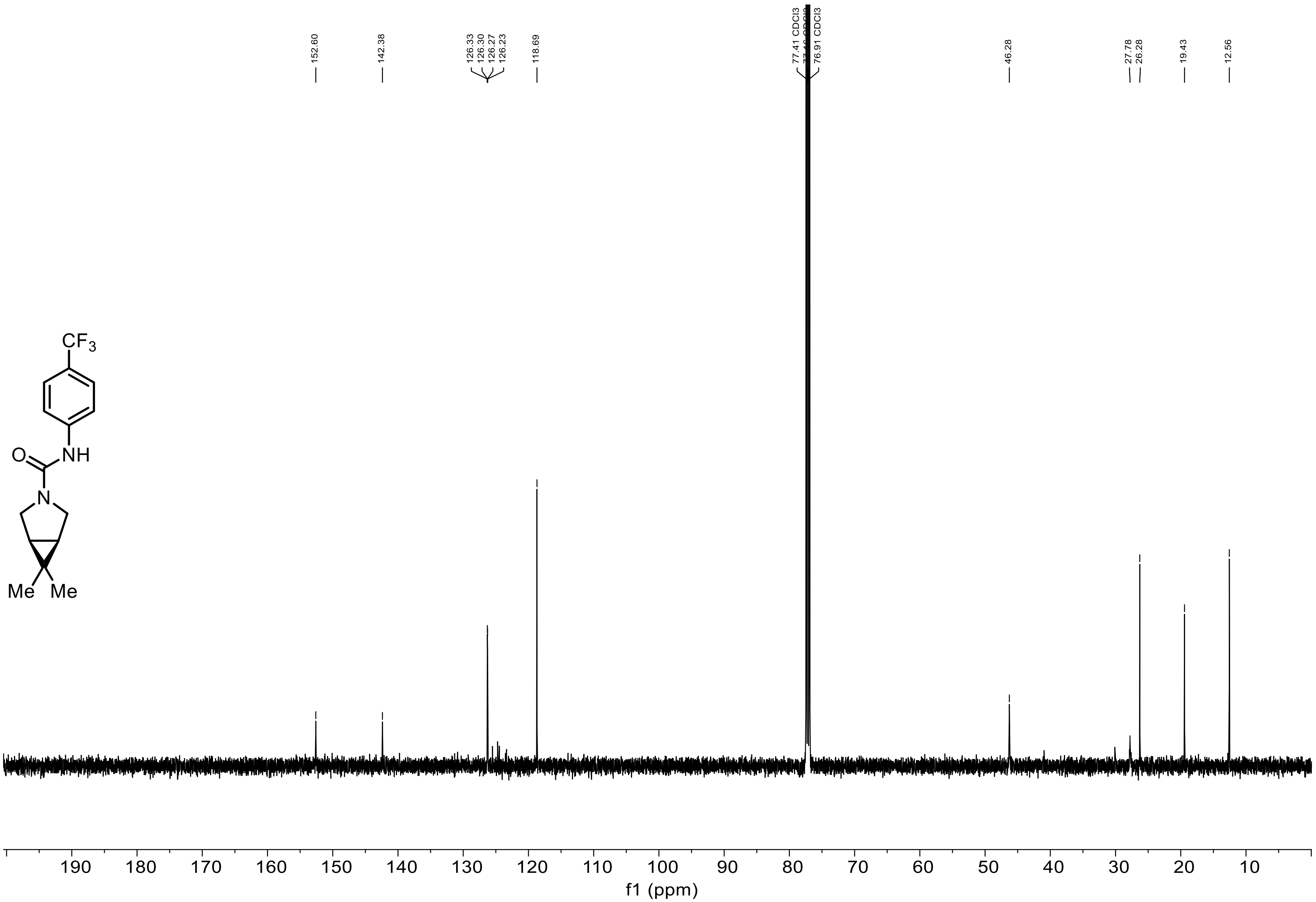

4-CF$_3$ ^{13}C NMR (126 MHz, $CDCl_3$). The ^{13}C peak for the CF_3-group was not observed due to poor signal to noise ratio.

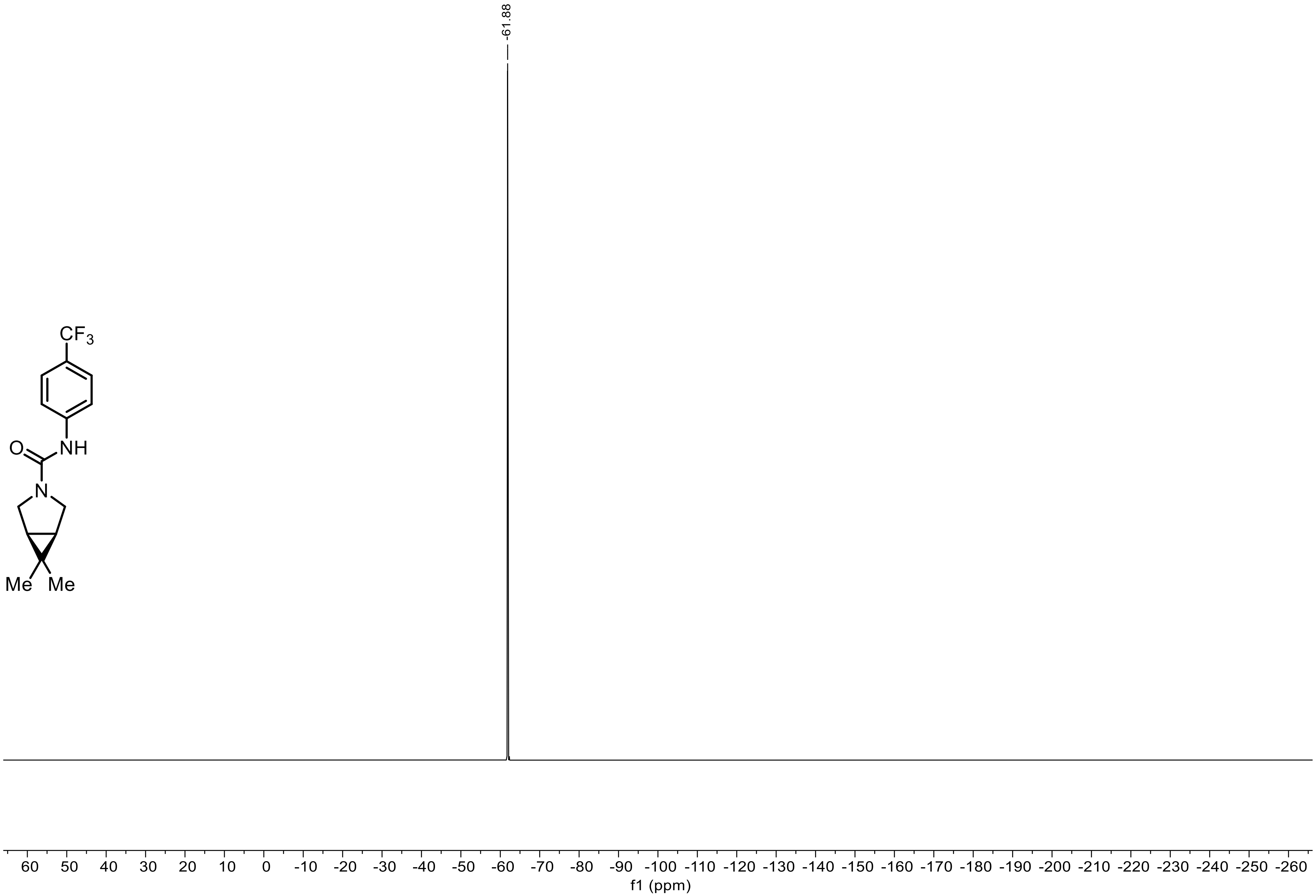

4-CF₃ ^{19}F NMR (470 MHz, $CDCl_3$).

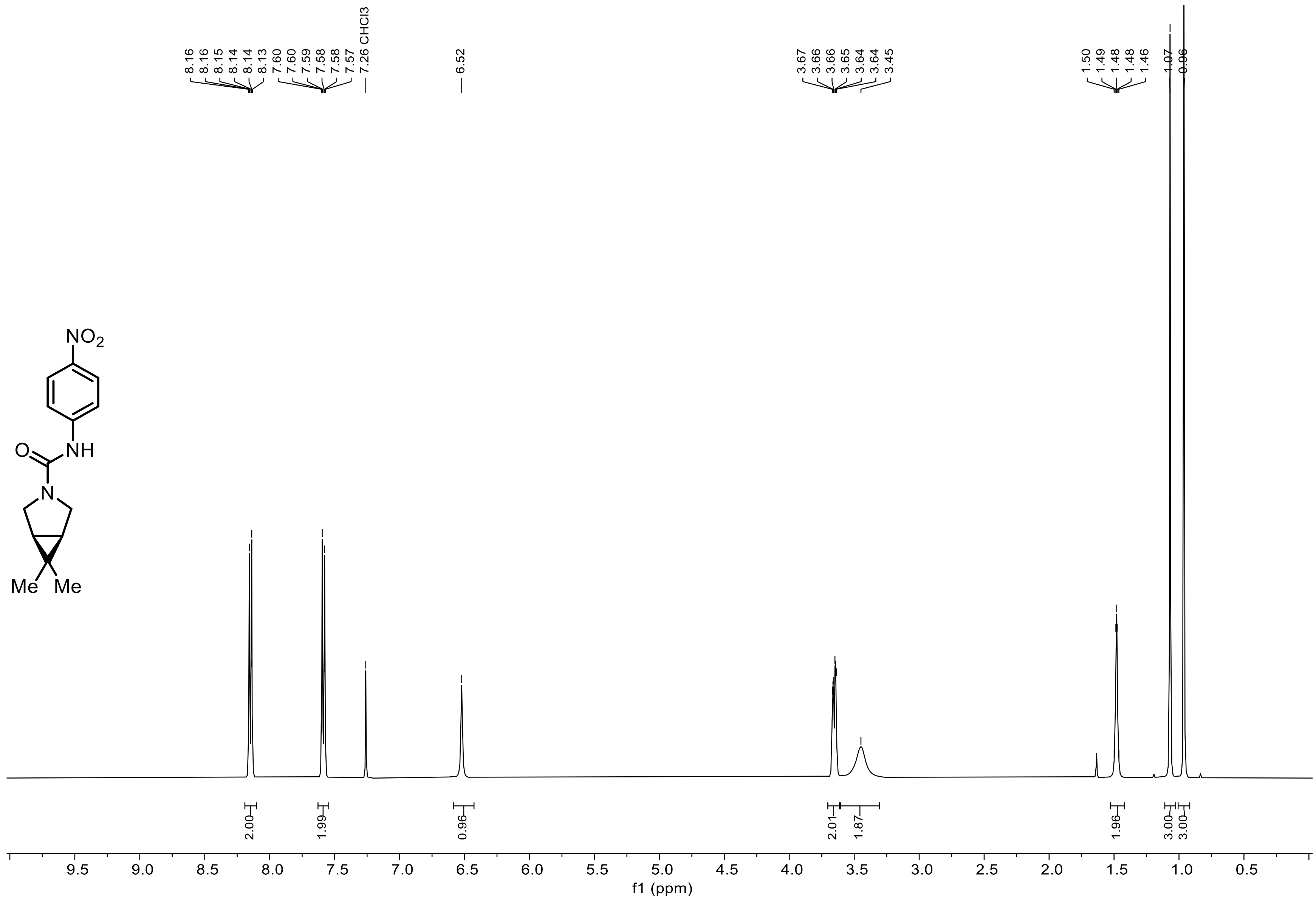

4-NO$_2$ ^{1}H NMR (500 MHz, $CDCl_3$).

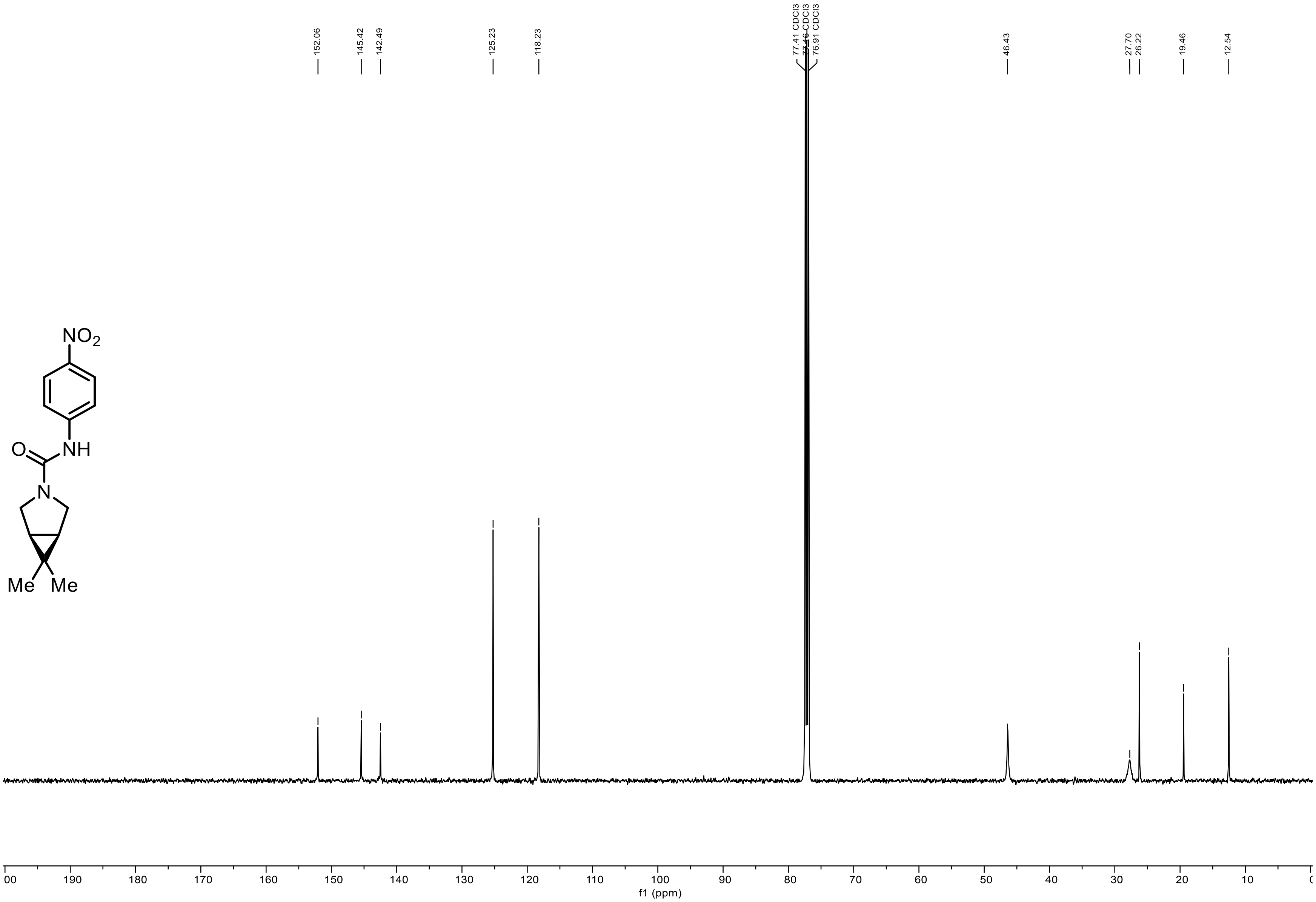

4-NO₂ ^{13}C NMR (126 MHz, CDCl$_3$).

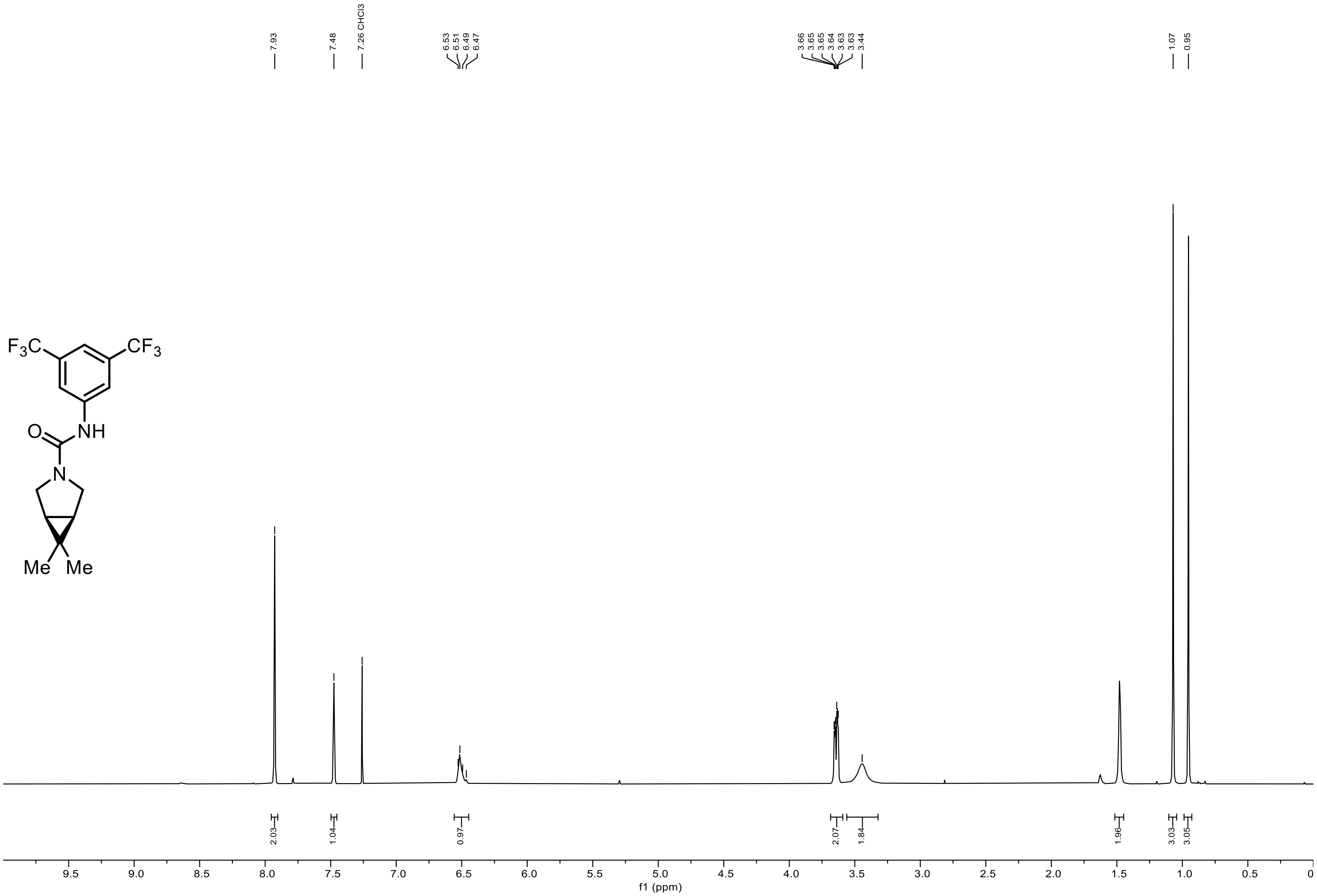

A ^{1}H NMR (500 MHz, $CDCl_3$).

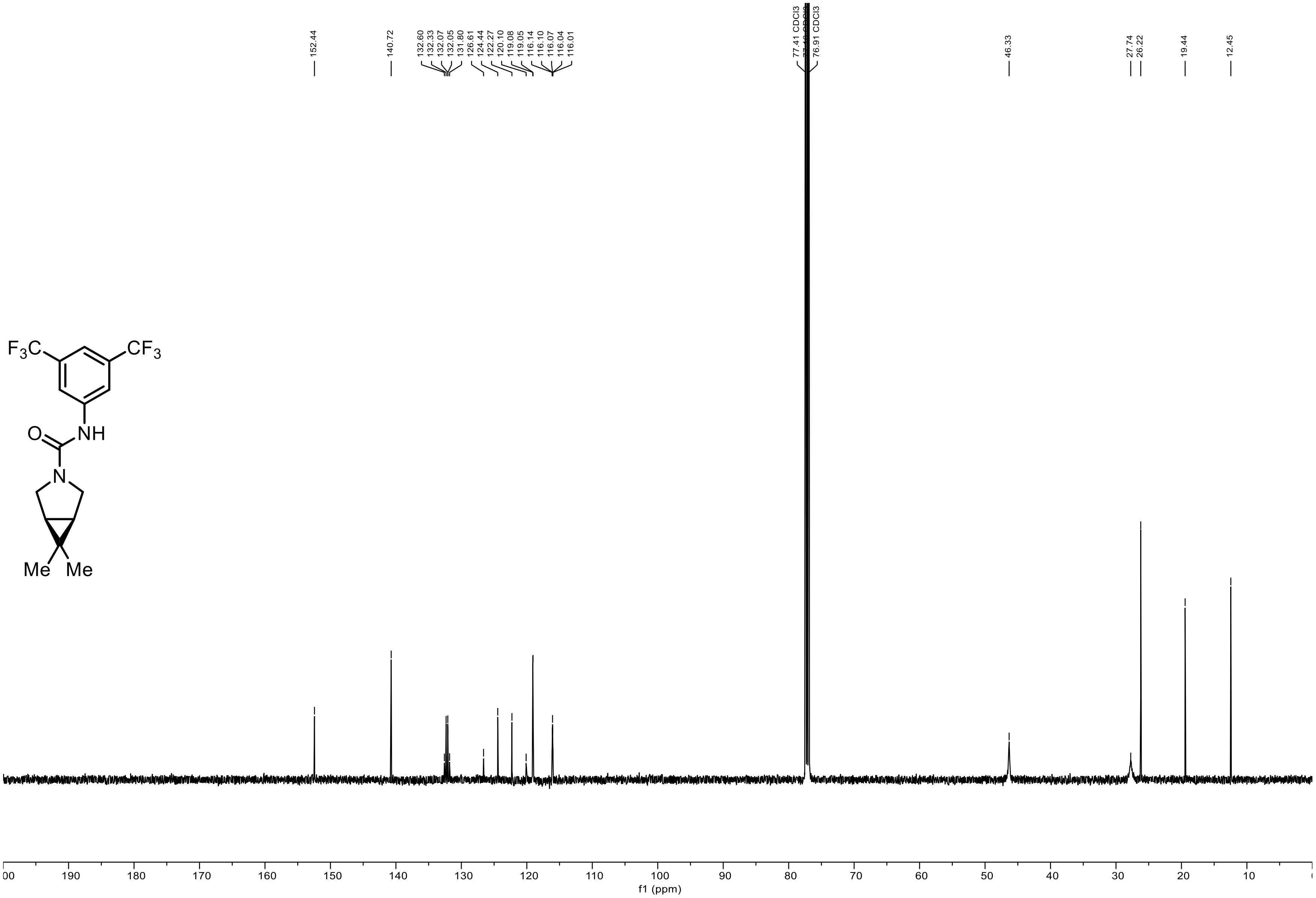

A ^{13}C NMR (126 MHz, $CDCl_3$).

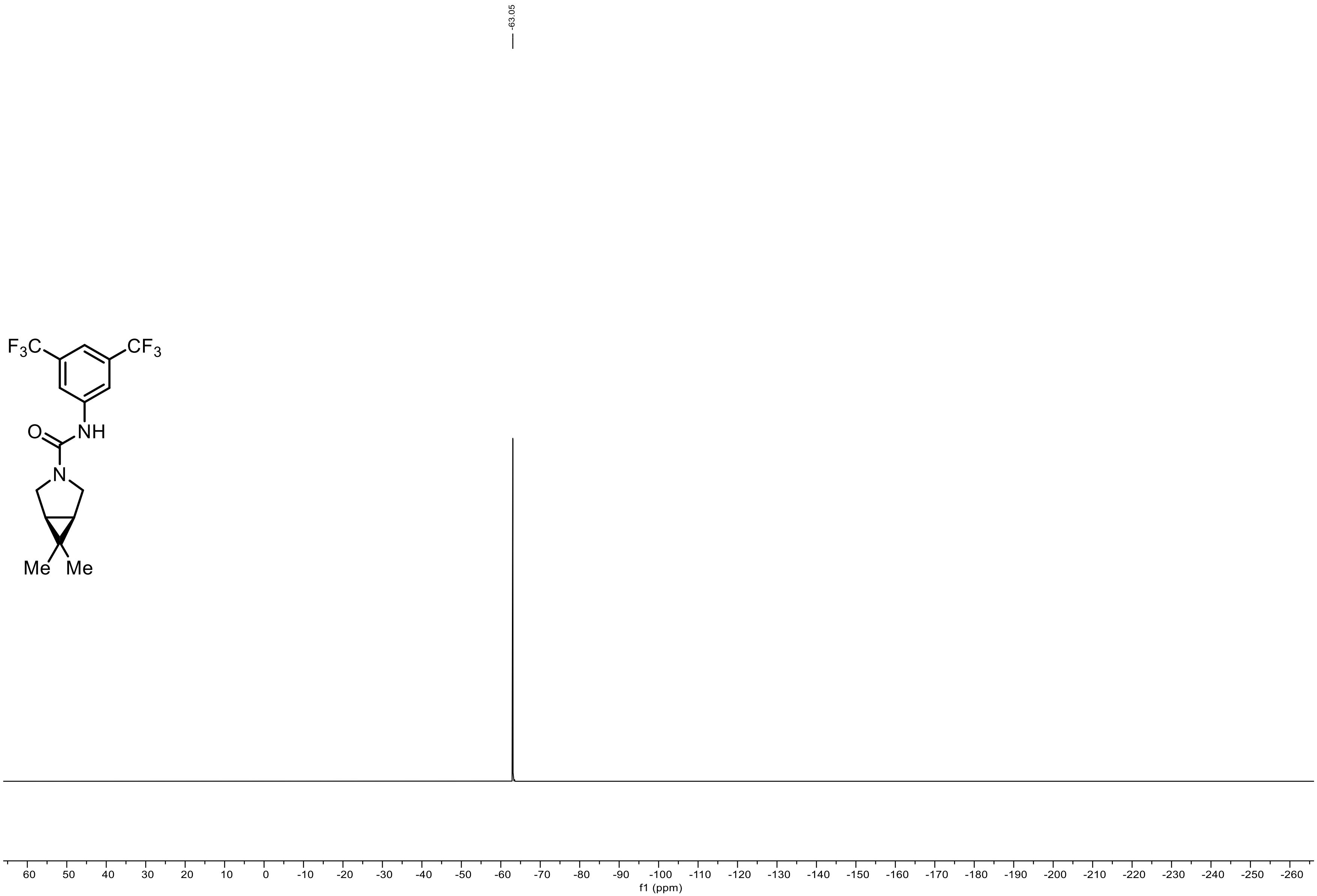

A ^{19}F NMR (470 MHz, $CDCl_3$).

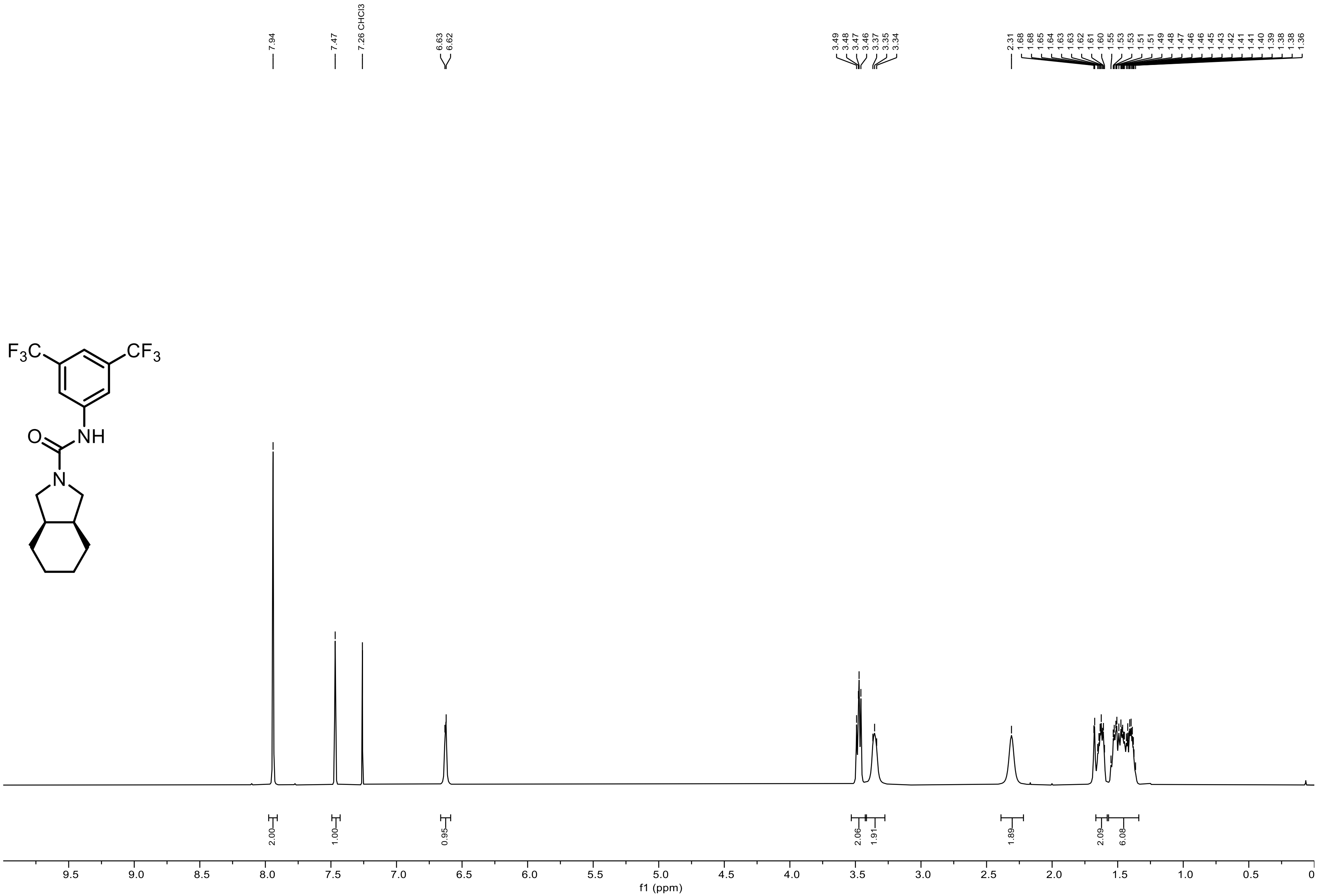

B ^{1}H NMR (500 MHz, $CDCl_3$).

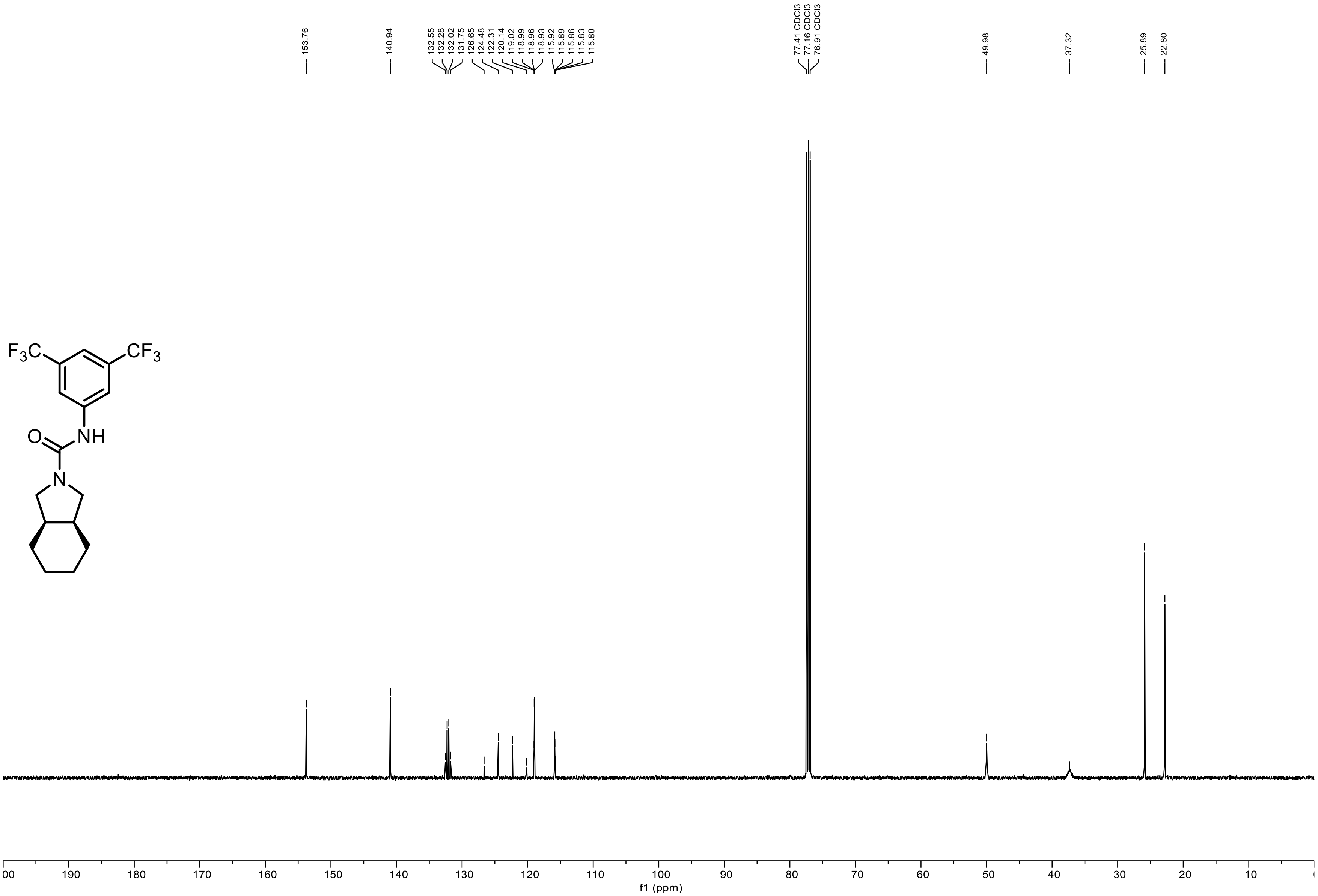

B ^{13}C NMR (126 MHz, $CDCl_3$).

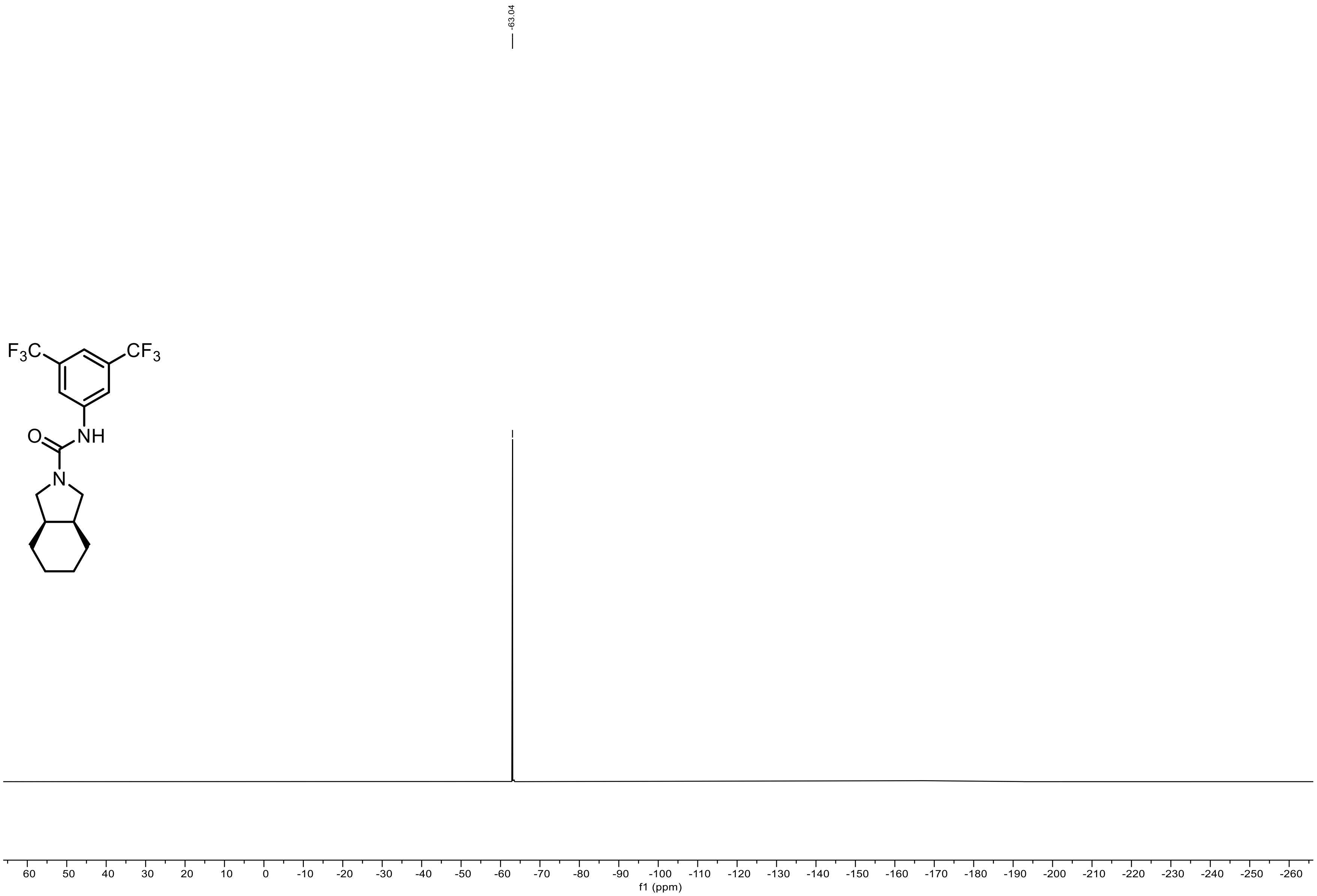

B ^{19}F NMR (470 MHz, $CDCl_3$).

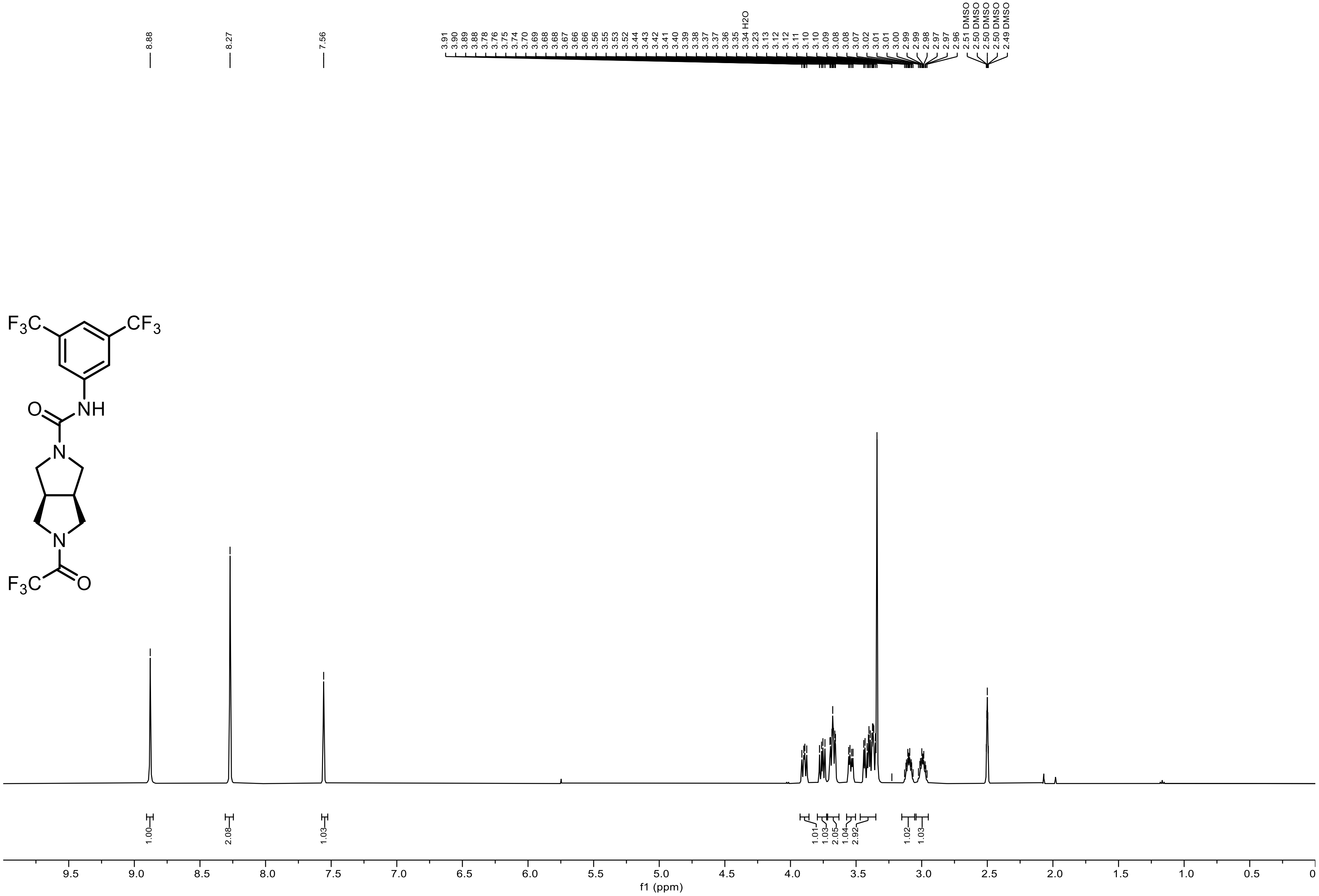

C ^{1}H NMR (500 MHz, DMSO-*d6*).

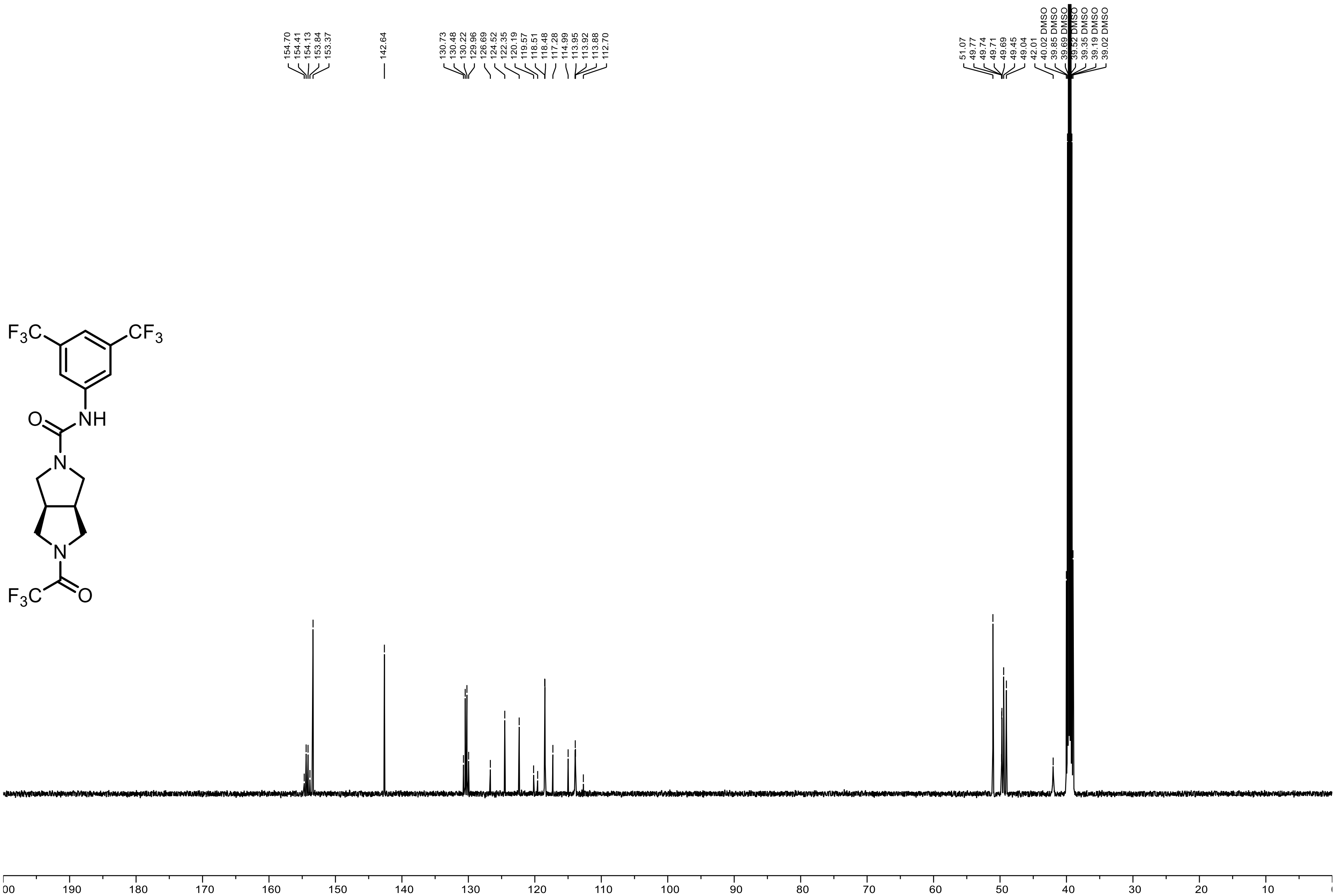

C ^{13}C NMR (126 MHz, DMSO-*d6*).

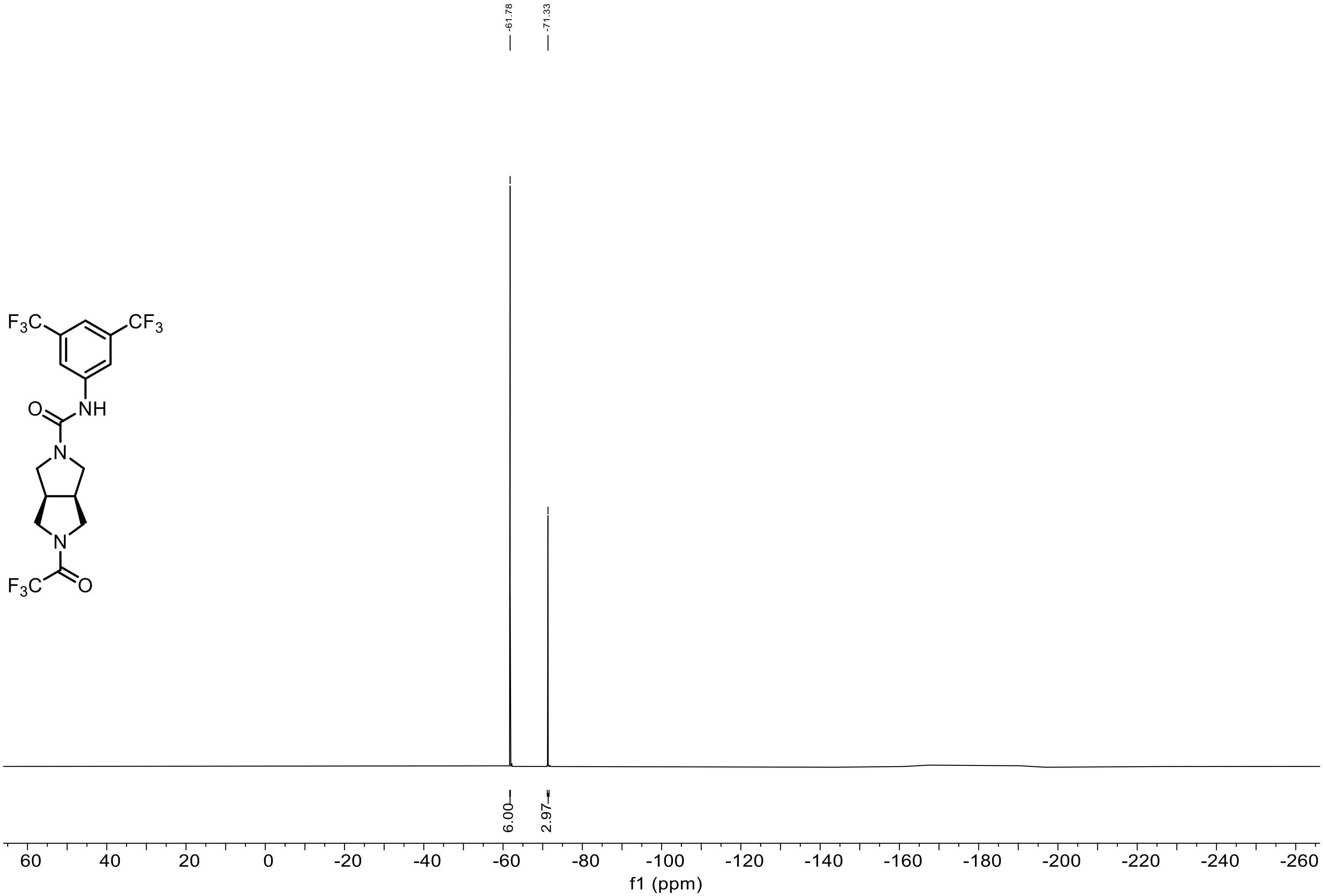

C ^{19}F NMR (470 MHz, DMSO-*d6*).

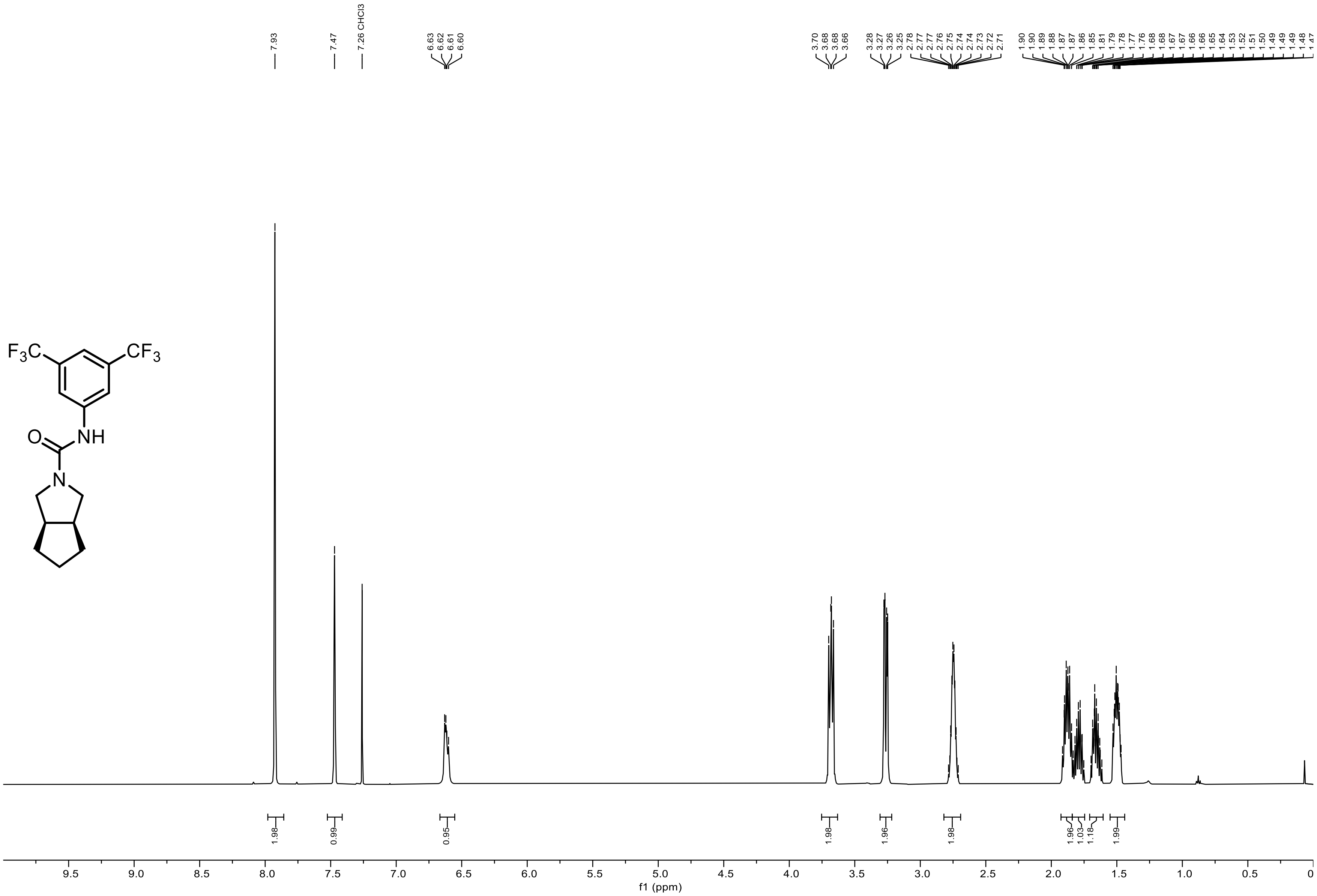

S1 ^{1}H NMR (500 MHz, $CDCl_3$).

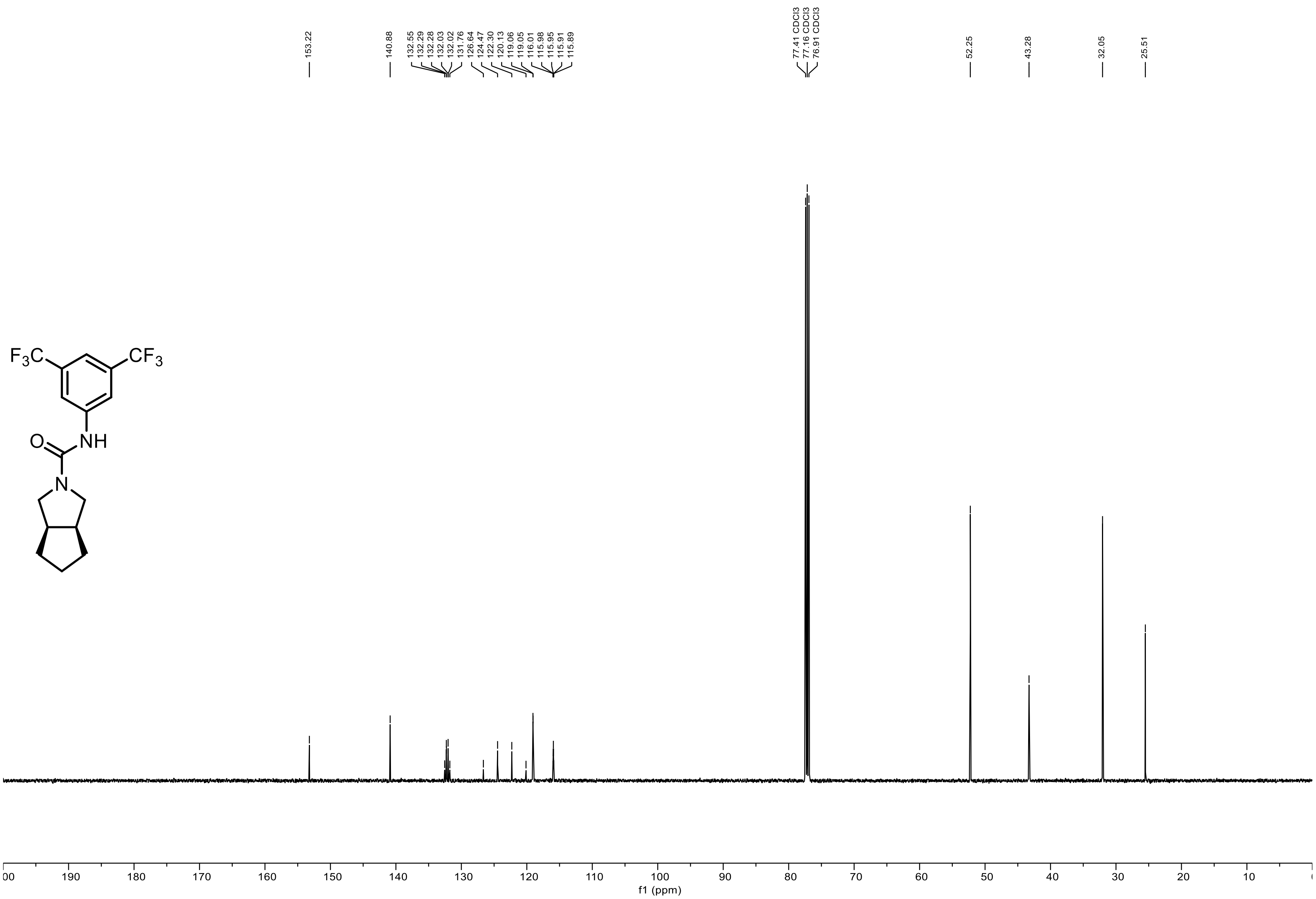

S1 ^{13}C NMR (126 MHz, $CDCl_3$).

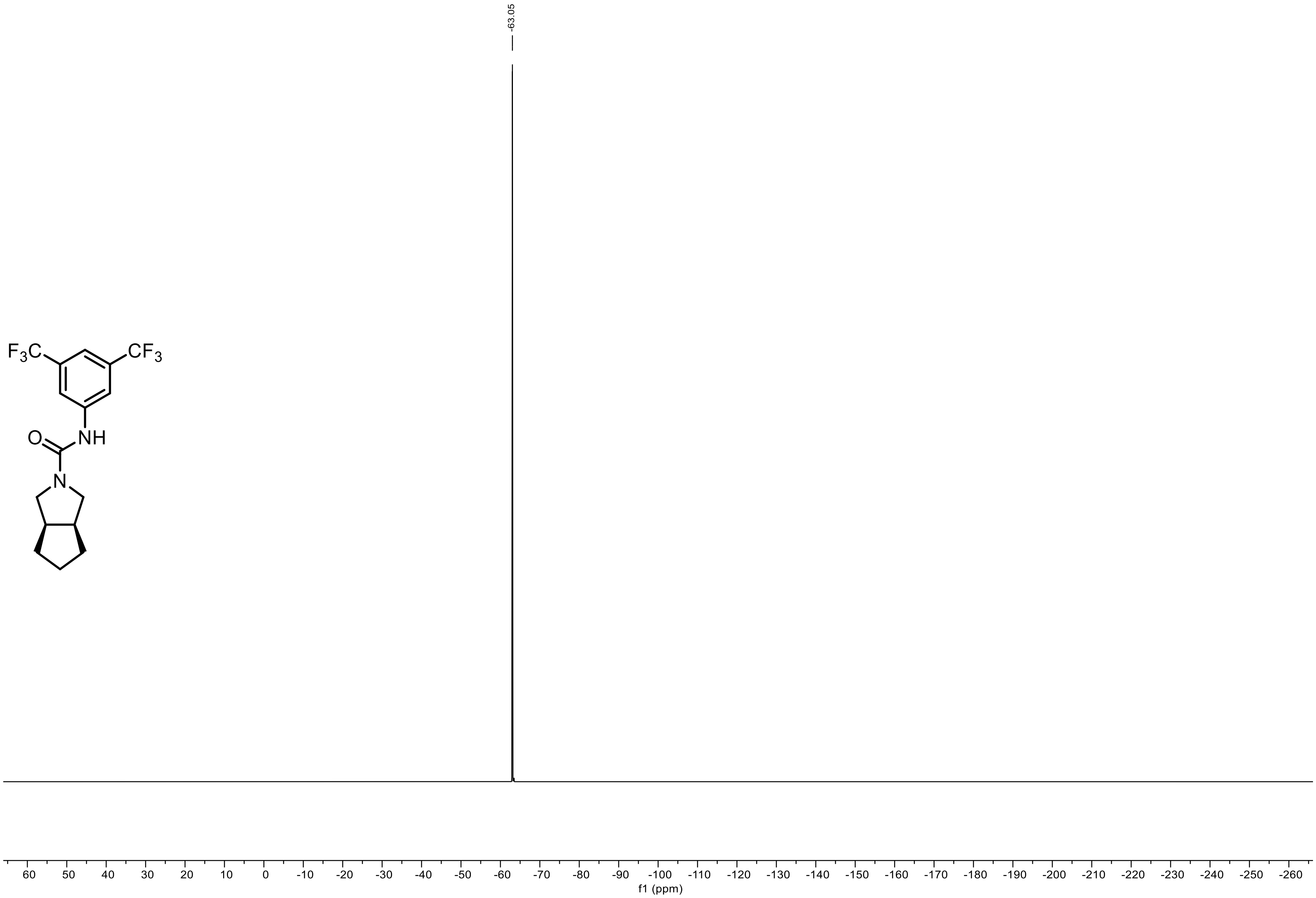

S1 ^{19}F NMR (470 MHz, $CDCl_3$).

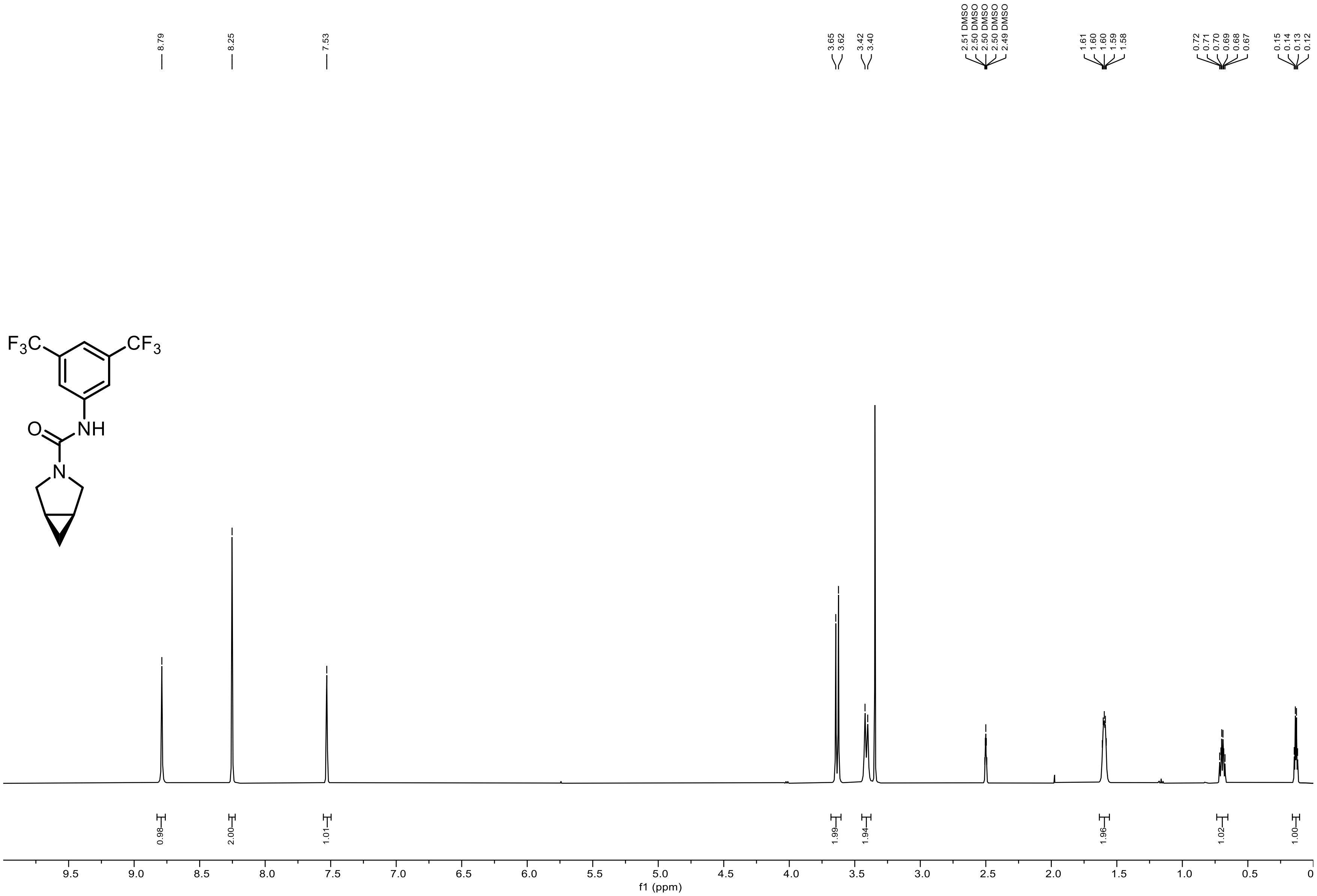

S2 ¹H NMR (500 MHz, DMSO -*d6*).

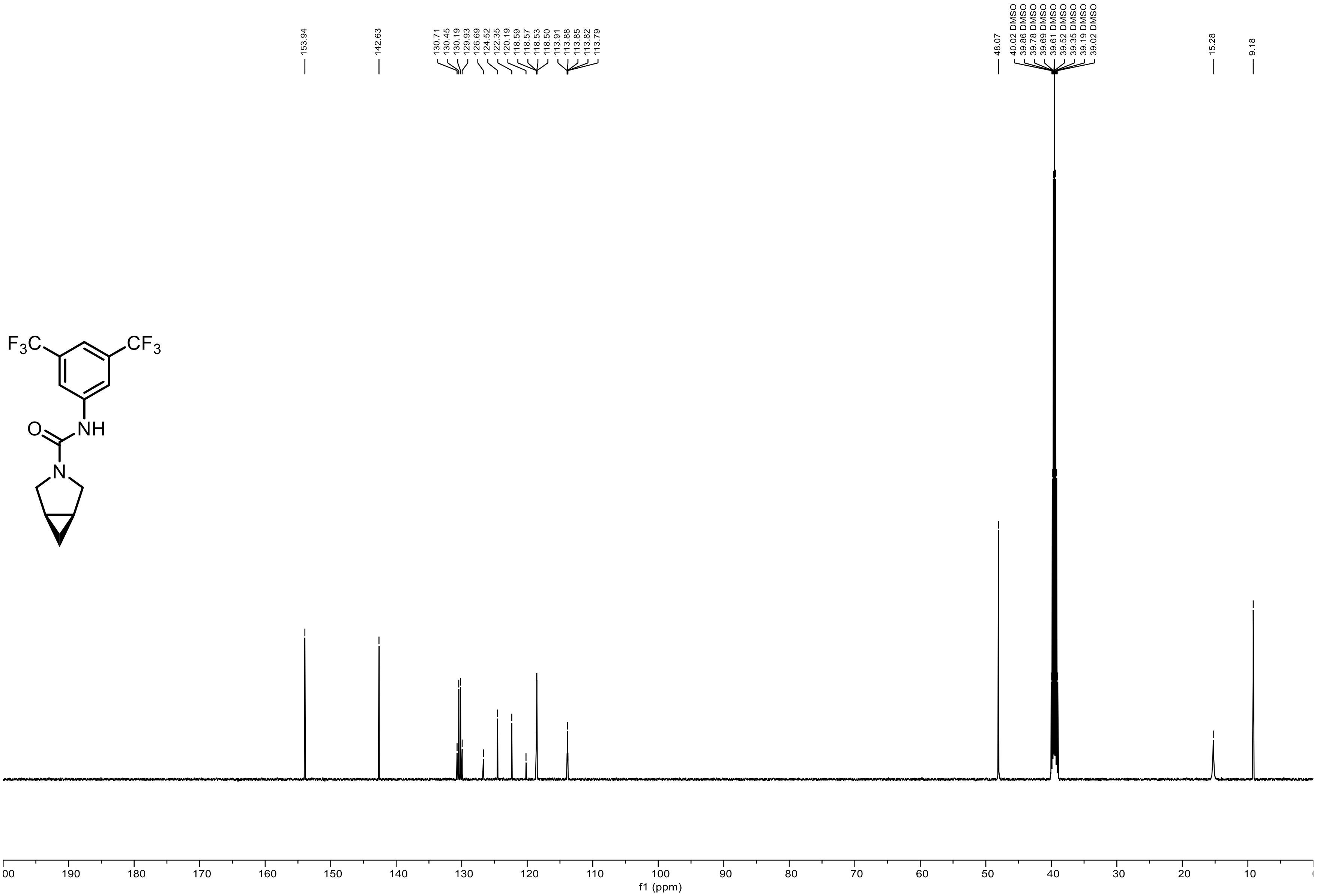

S2 ^{13}C NMR (126 MHz, DMSO-*d6*).

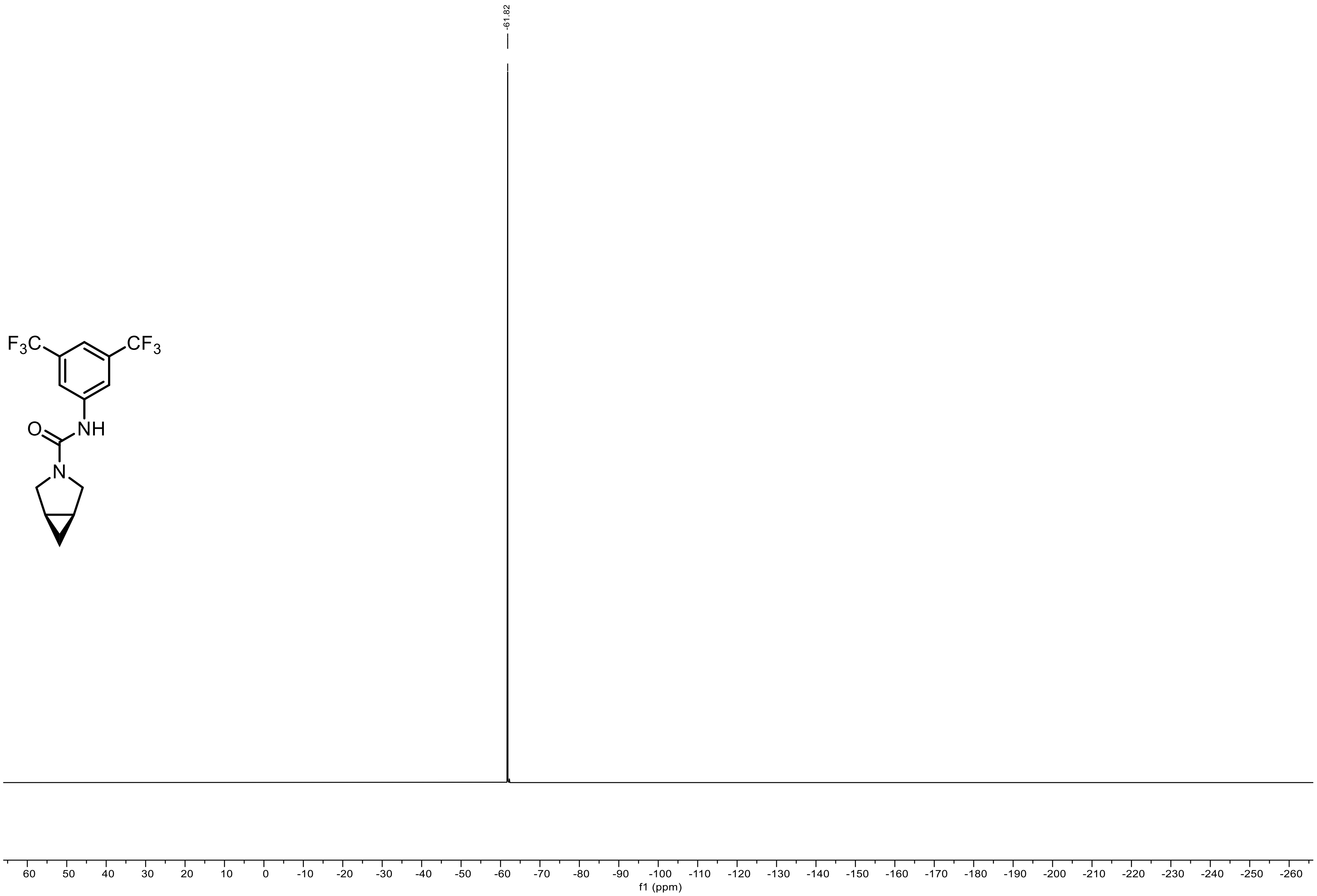

S2 ^{19}F NMR (470 MHz, DMSO-*d6*).

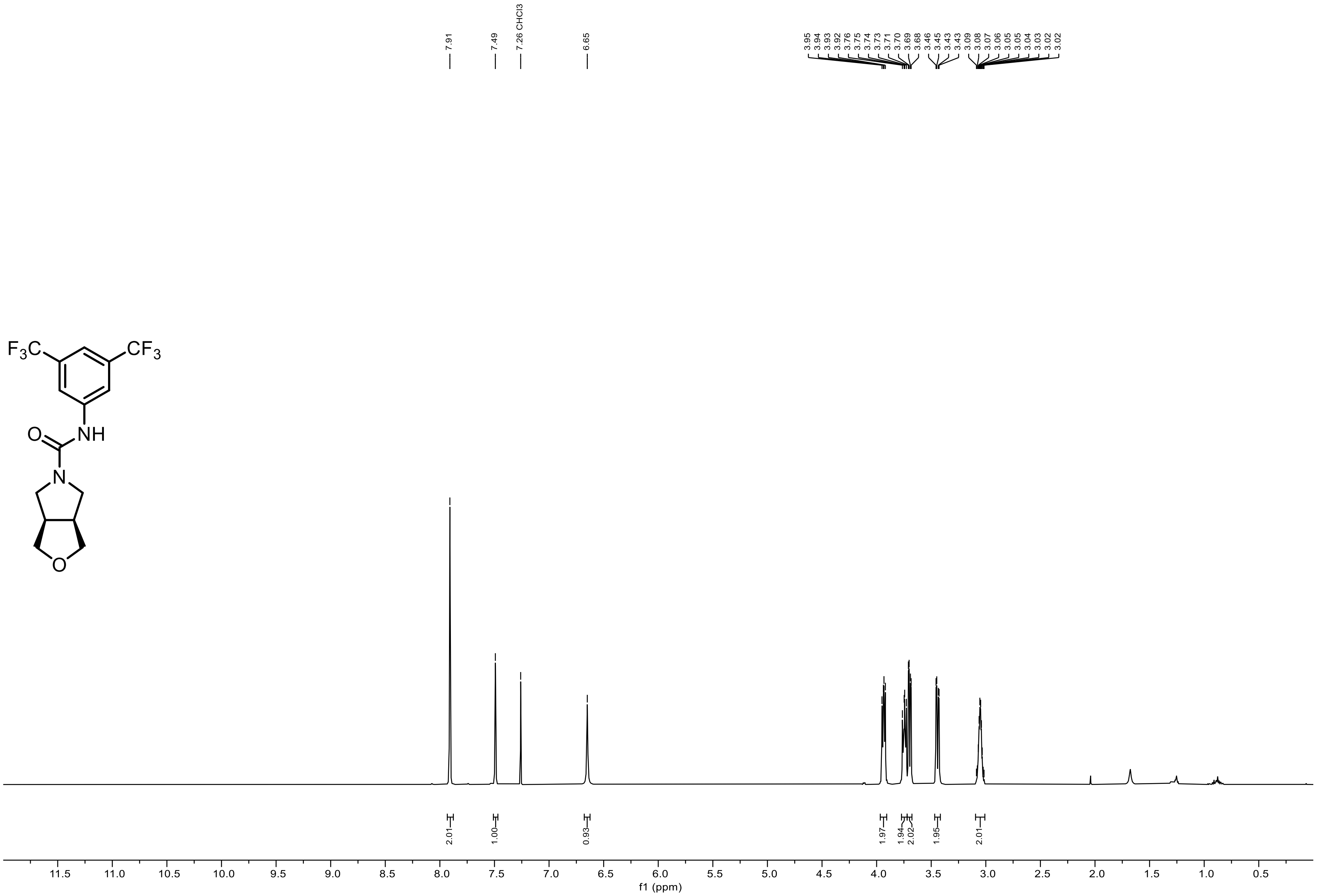

S3 ^{1}H NMR (500 MHz, $CDCl_3$).

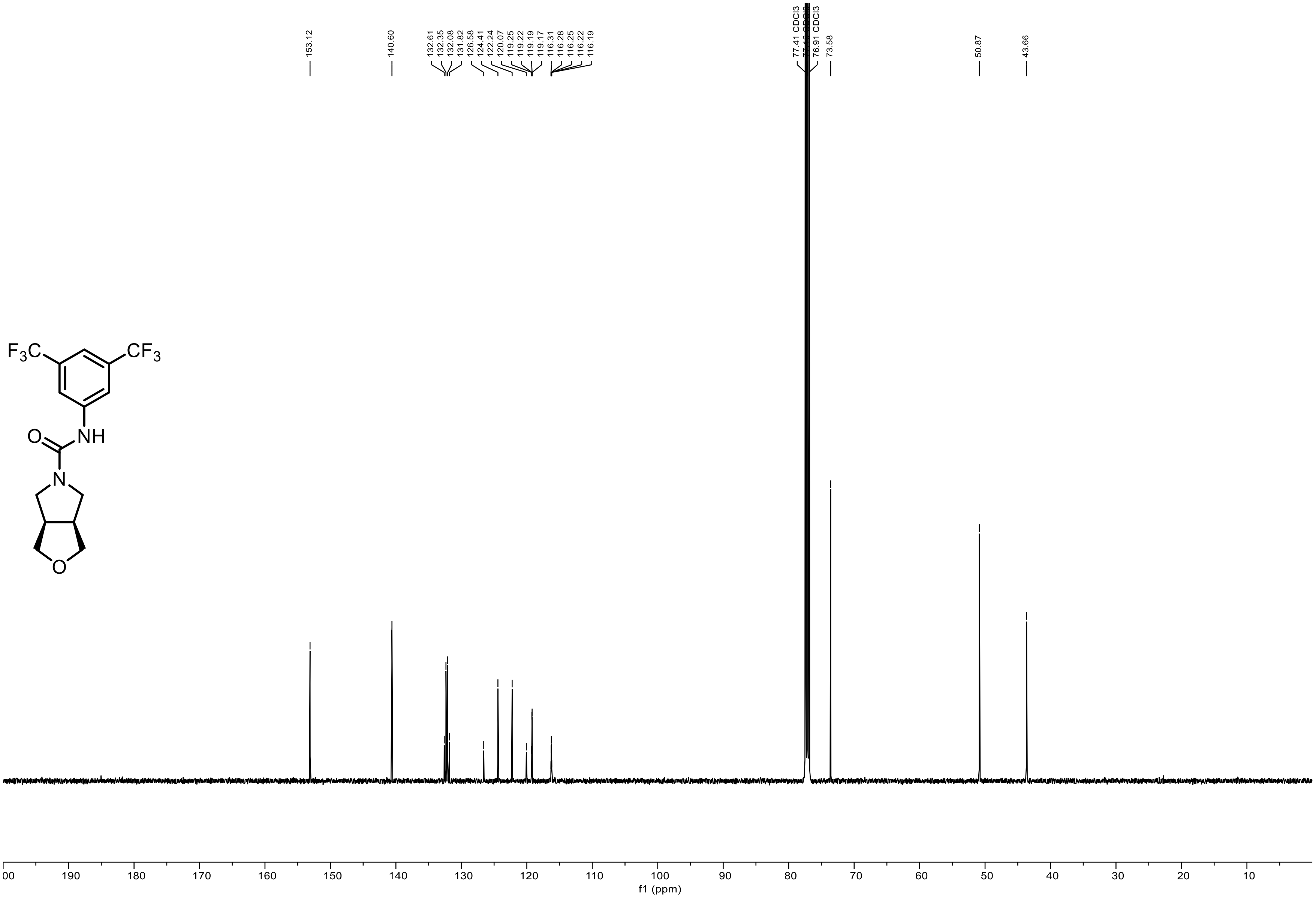

S3 ^{13}C NMR (126 MHz, $CDCl_3$).

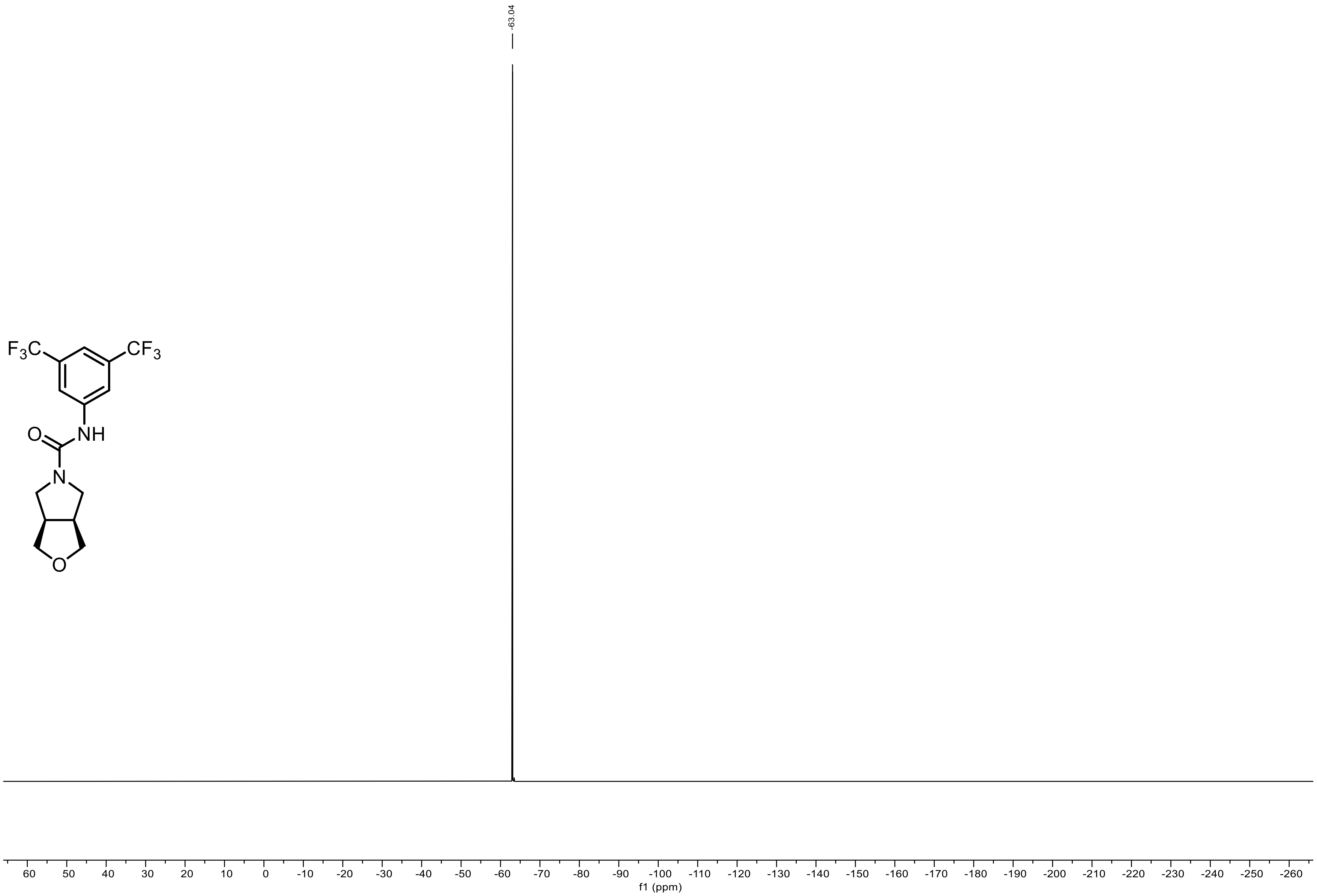

S3 ^{19}F NMR (470 MHz, $CDCl_3$).

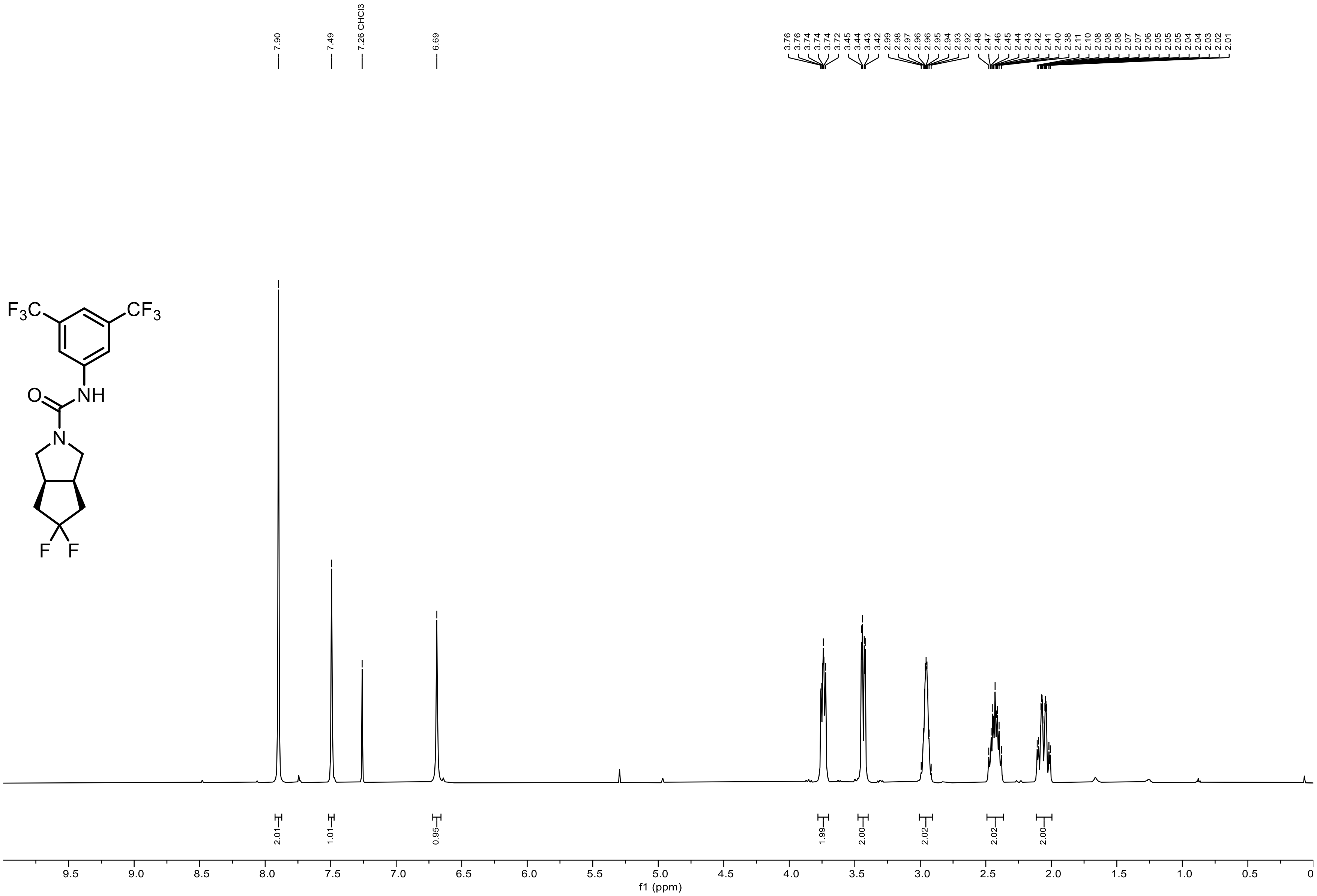

S4 ^{1}H NMR (500 MHz, $CDCl_3$).

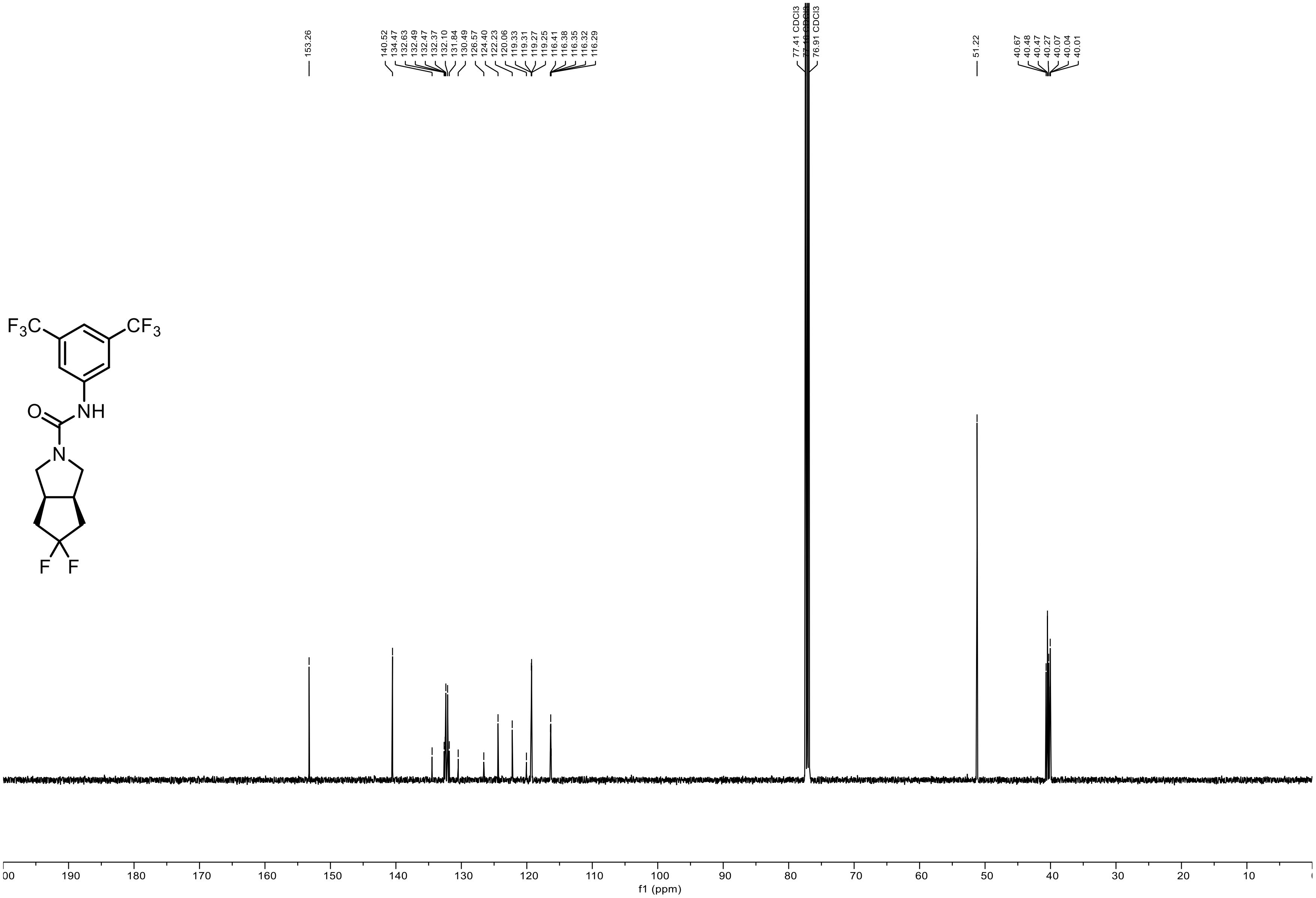

S4 ^{13}C NMR (126 MHz, $CDCl_3$).

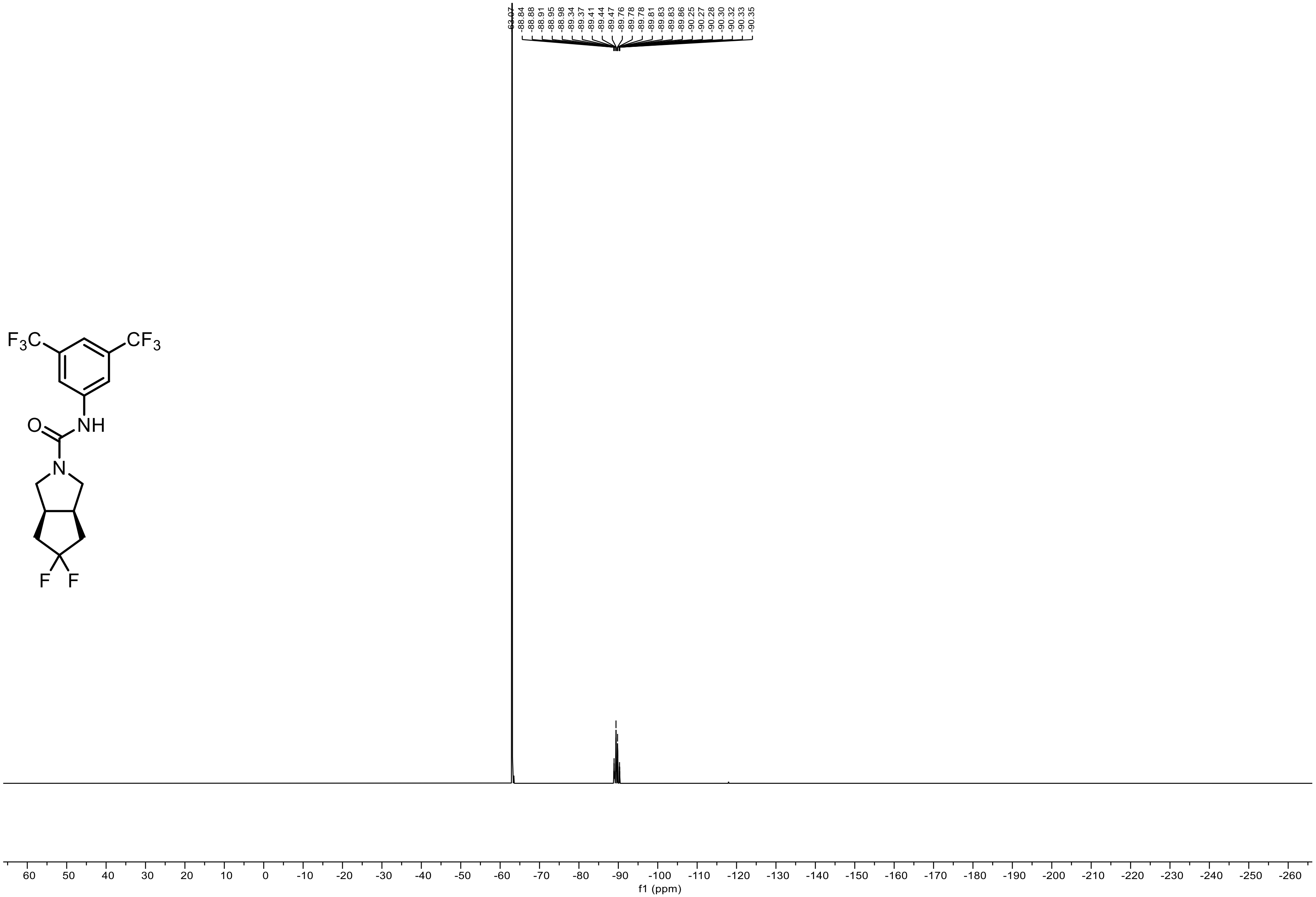

S4 ^{19}F NMR (470 MHz, $CDCl_3$).

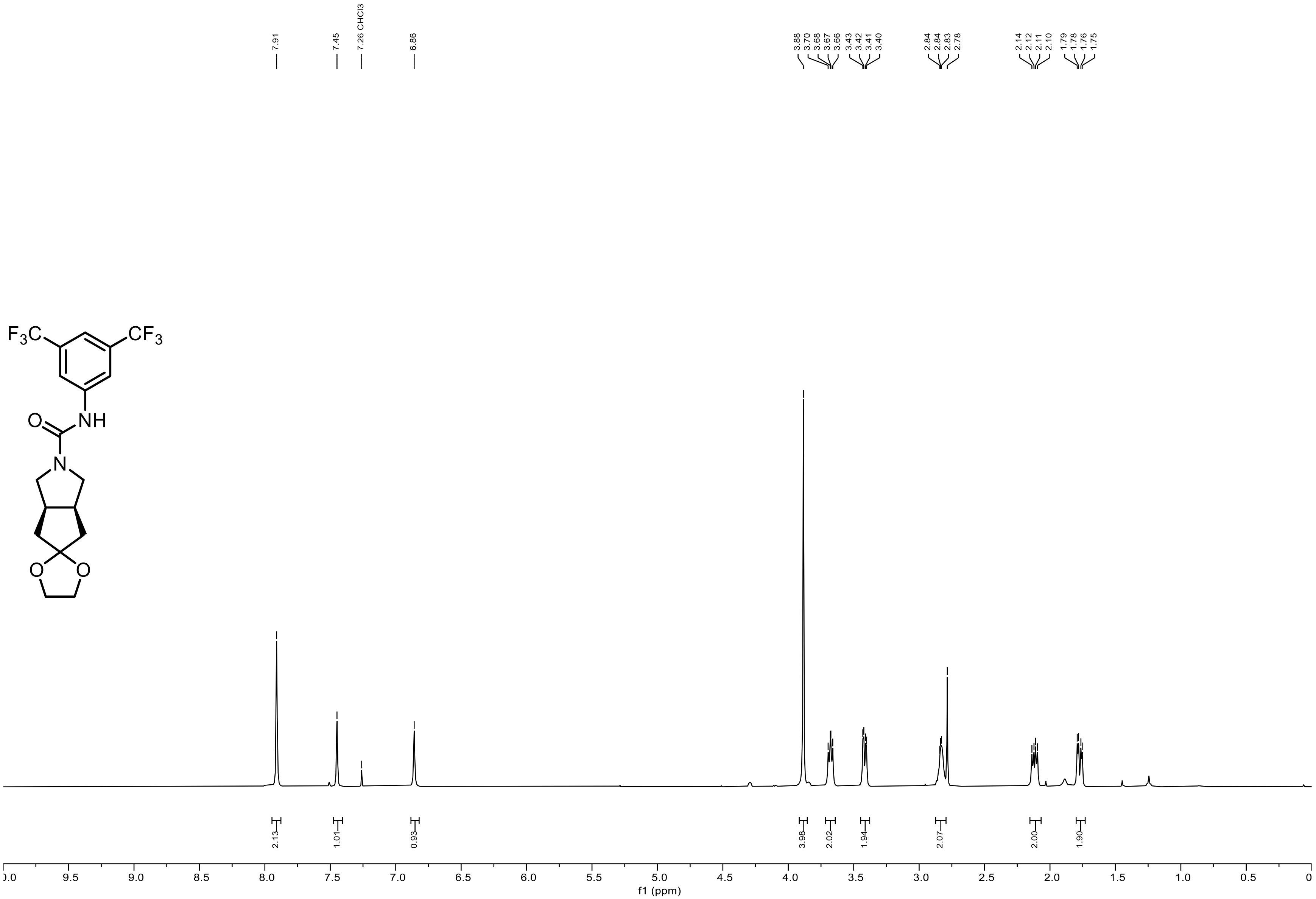

S5 ^{1}H NMR (500 MHz, $CDCl_3$). Unidentified impurity at 2.78 ppm.

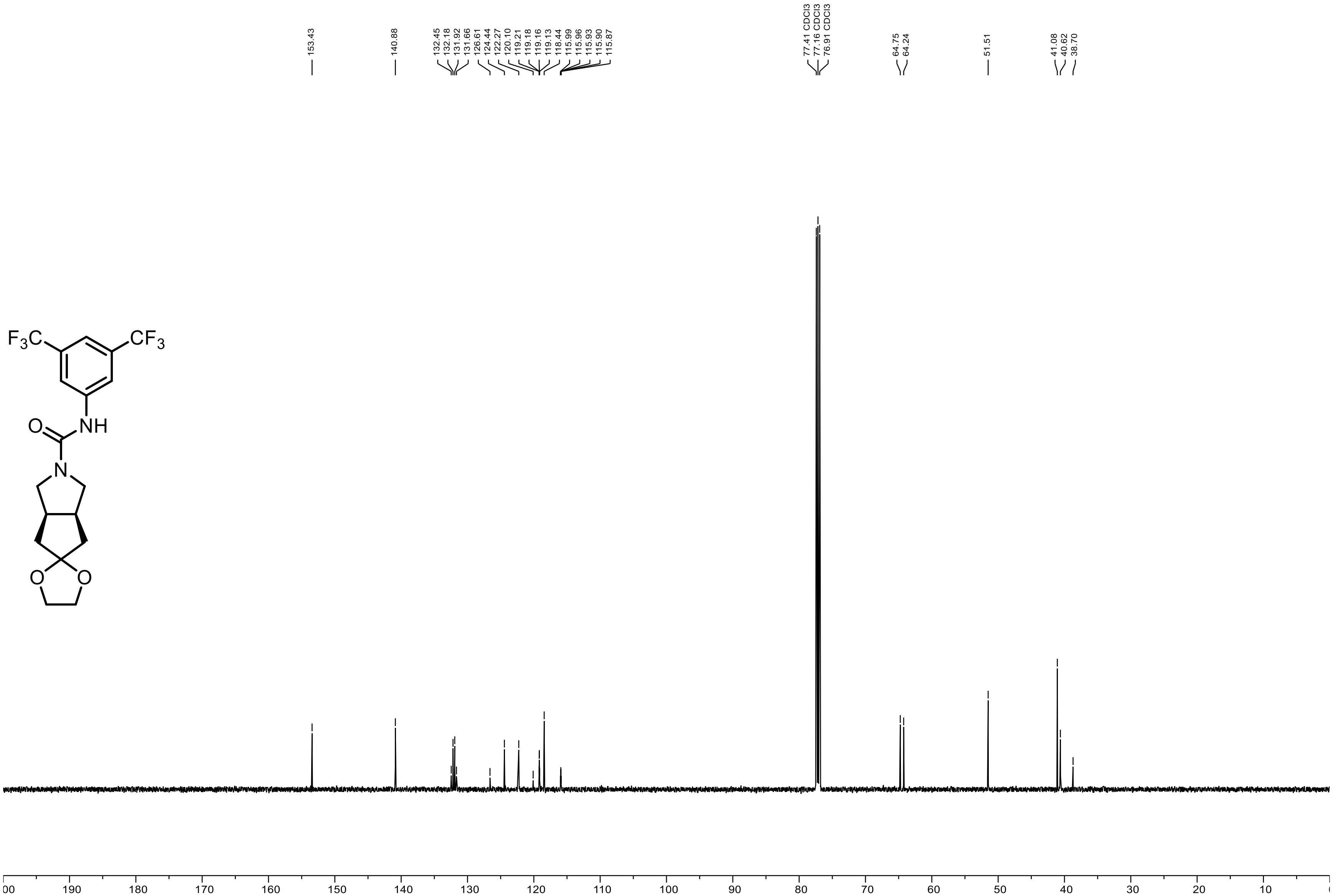

S5 ^{13}C NMR (126 MHz, $CDCl_3$). Unidentified impurity at 38.7 ppm.

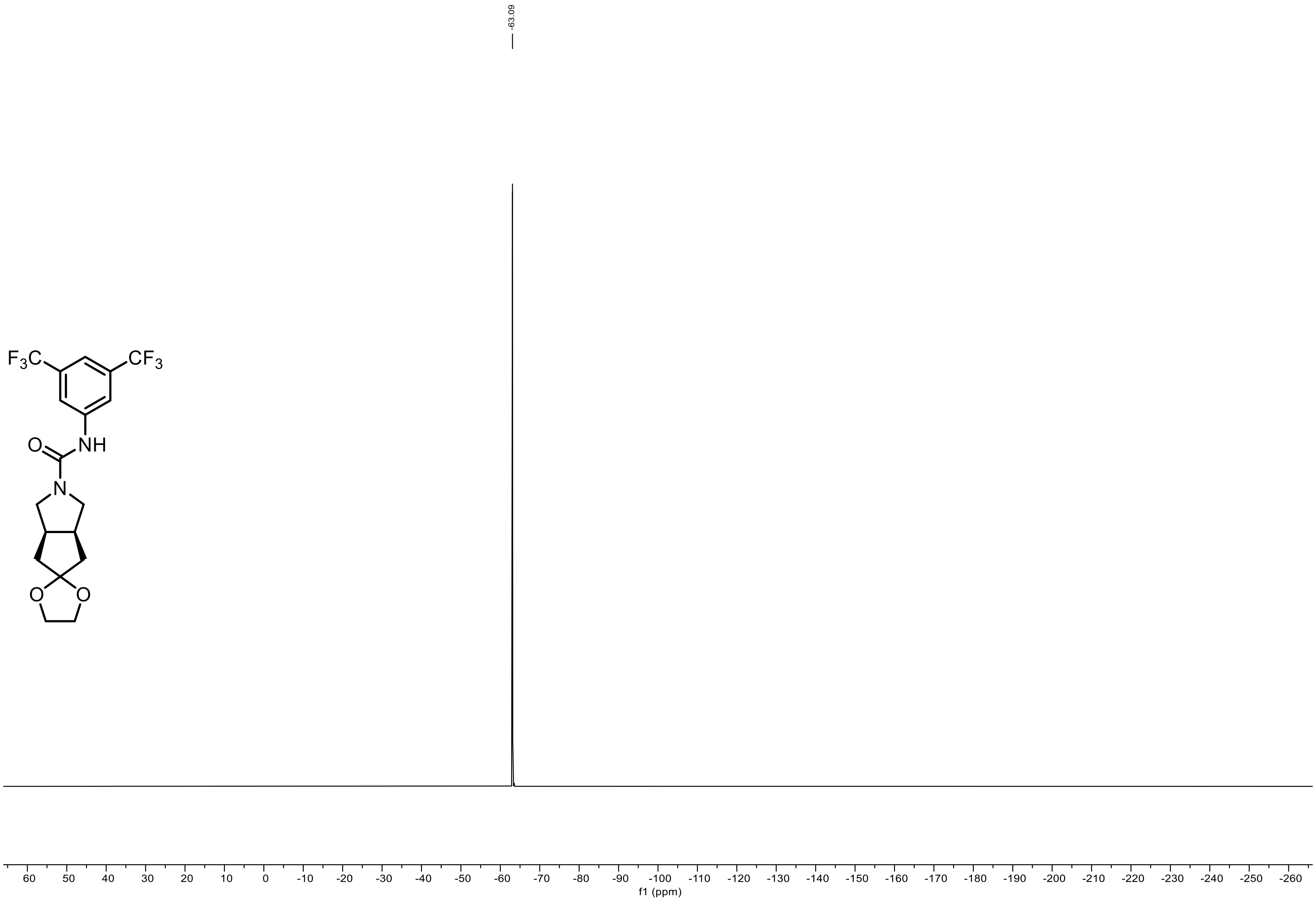

S5 ^{19}F NMR (470 MHz, $CDCl_3$).

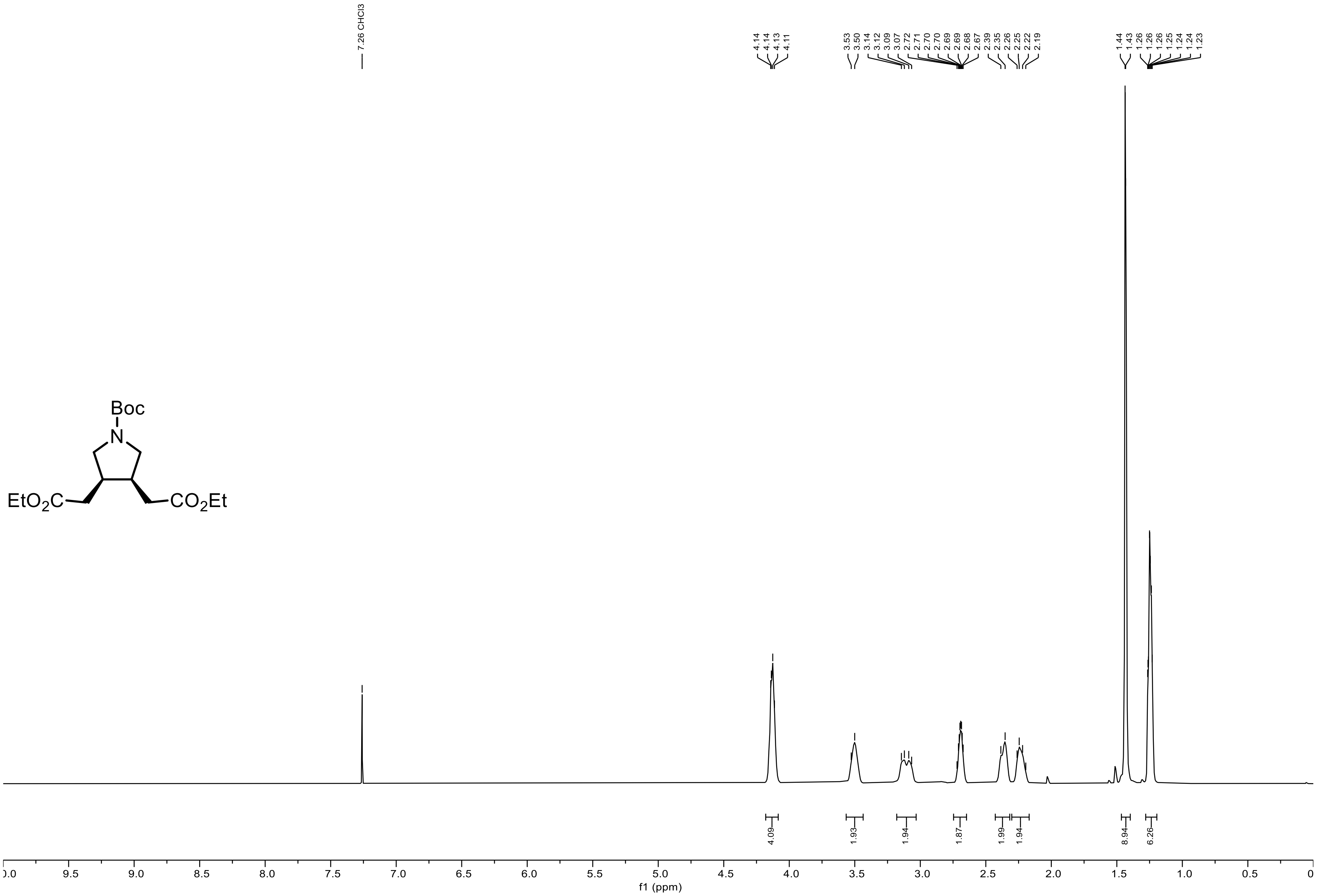

S6 ^{1}H NMR (500 MHz, $CDCl_3$).

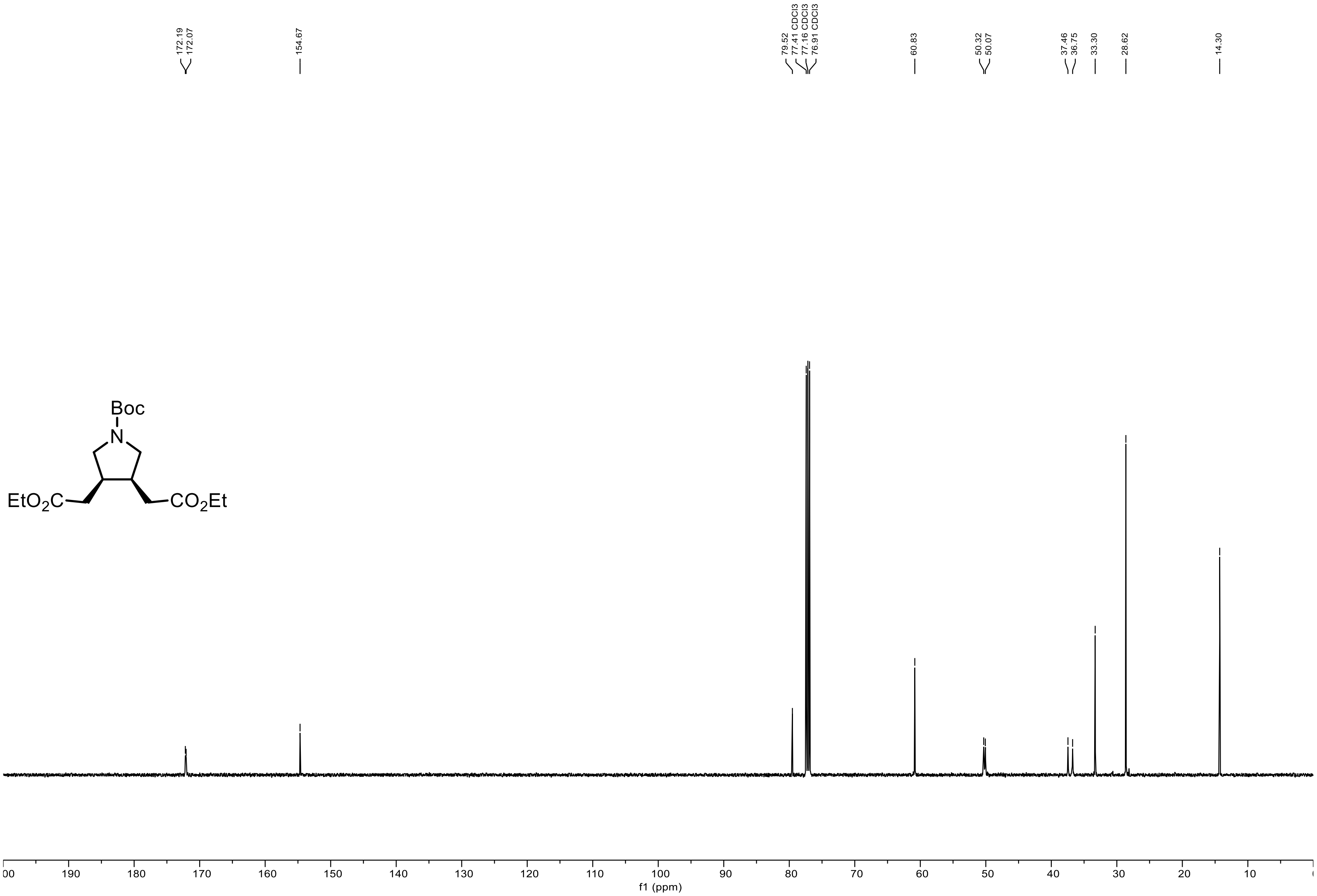

S6 ^{13}C NMR (126 MHz, $CDCl_3$).

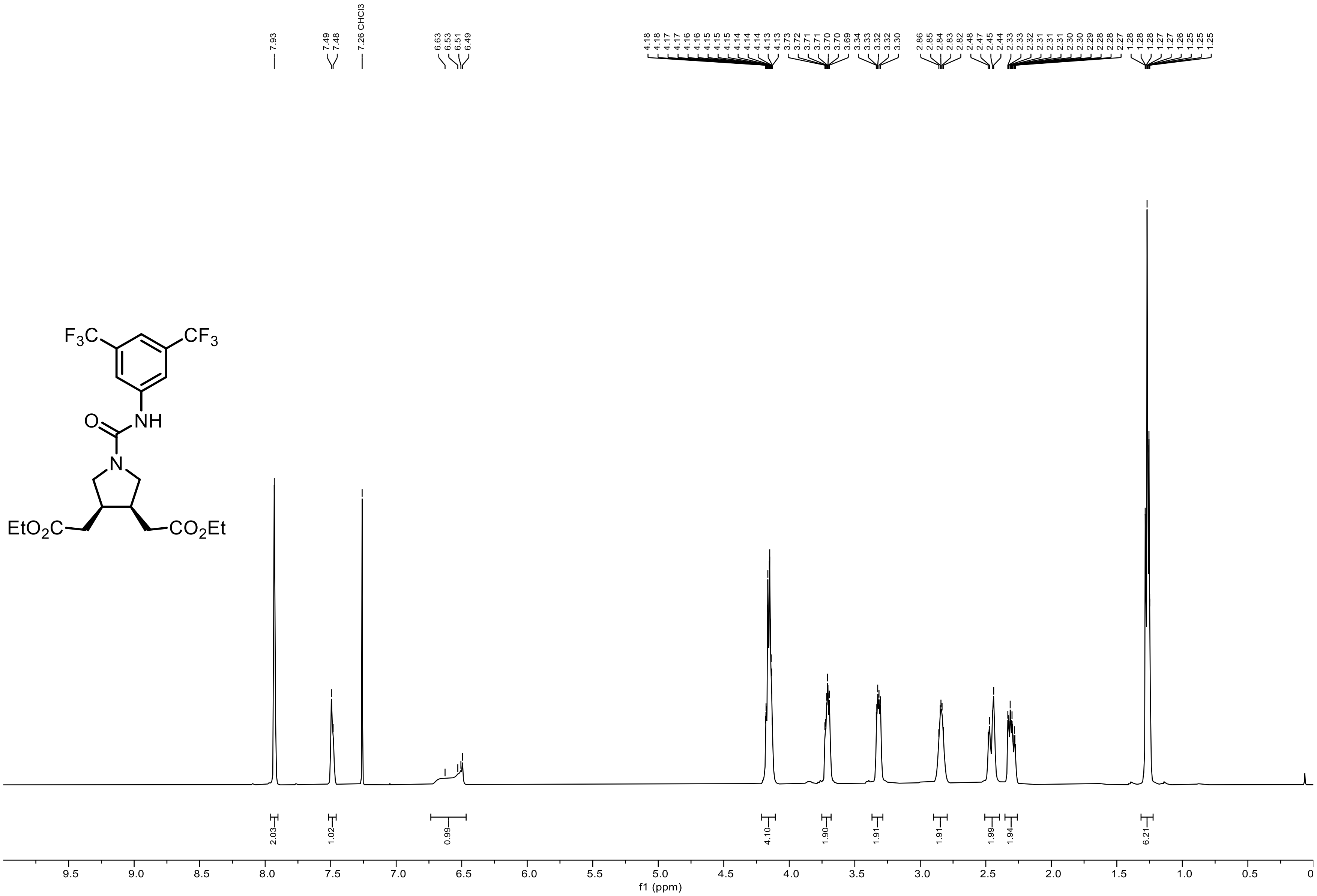

S7 ^{1}H NMR (500 MHz, $CDCl_3$).

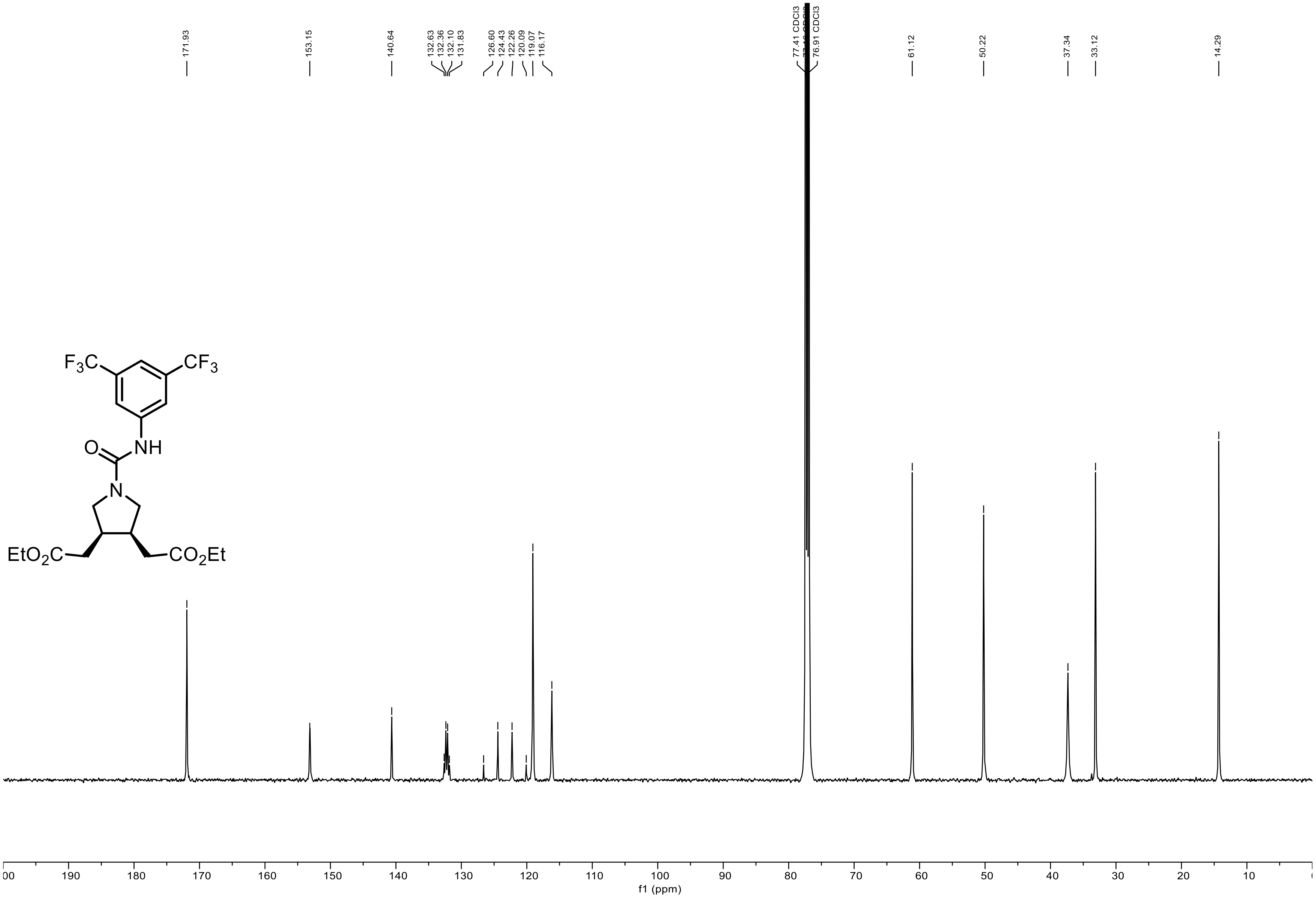

S7 ^{13}C NMR (126 MHz, $CDCl_3$).

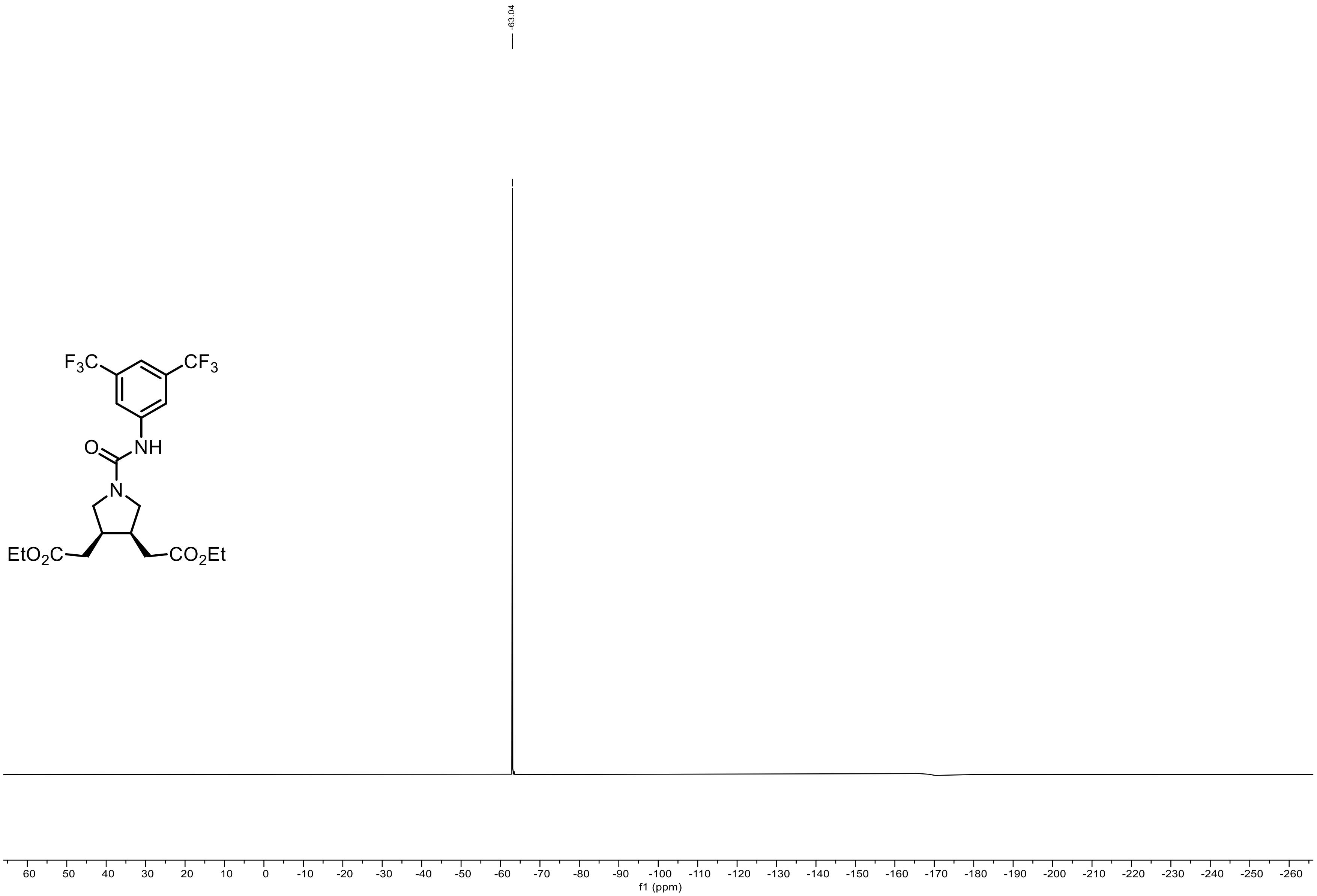

S7 ^{19}F NMR (470 MHz, $CDCl_3$).

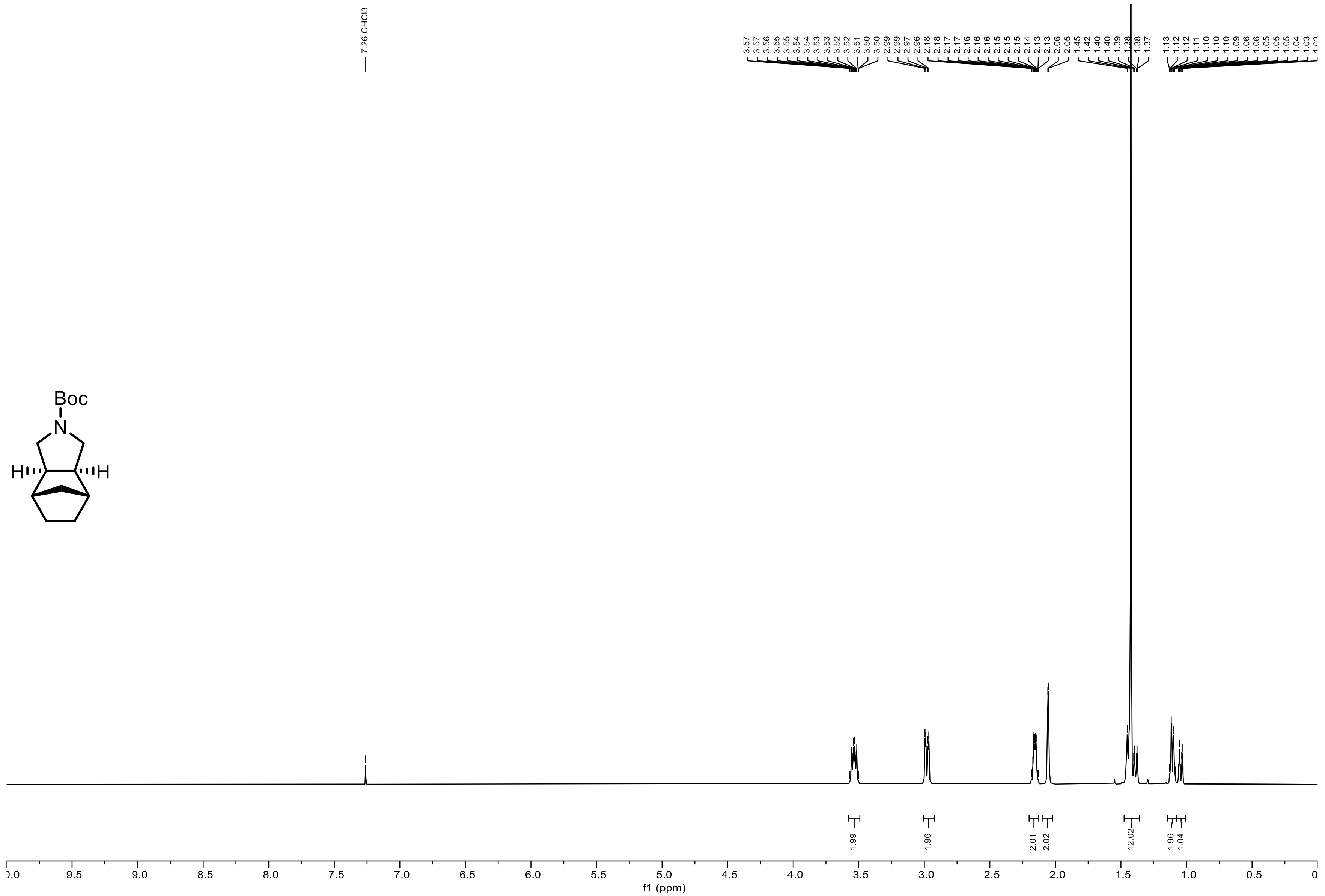

S8 ^{1}H NMR (500 MHz, $CDCl_3$).

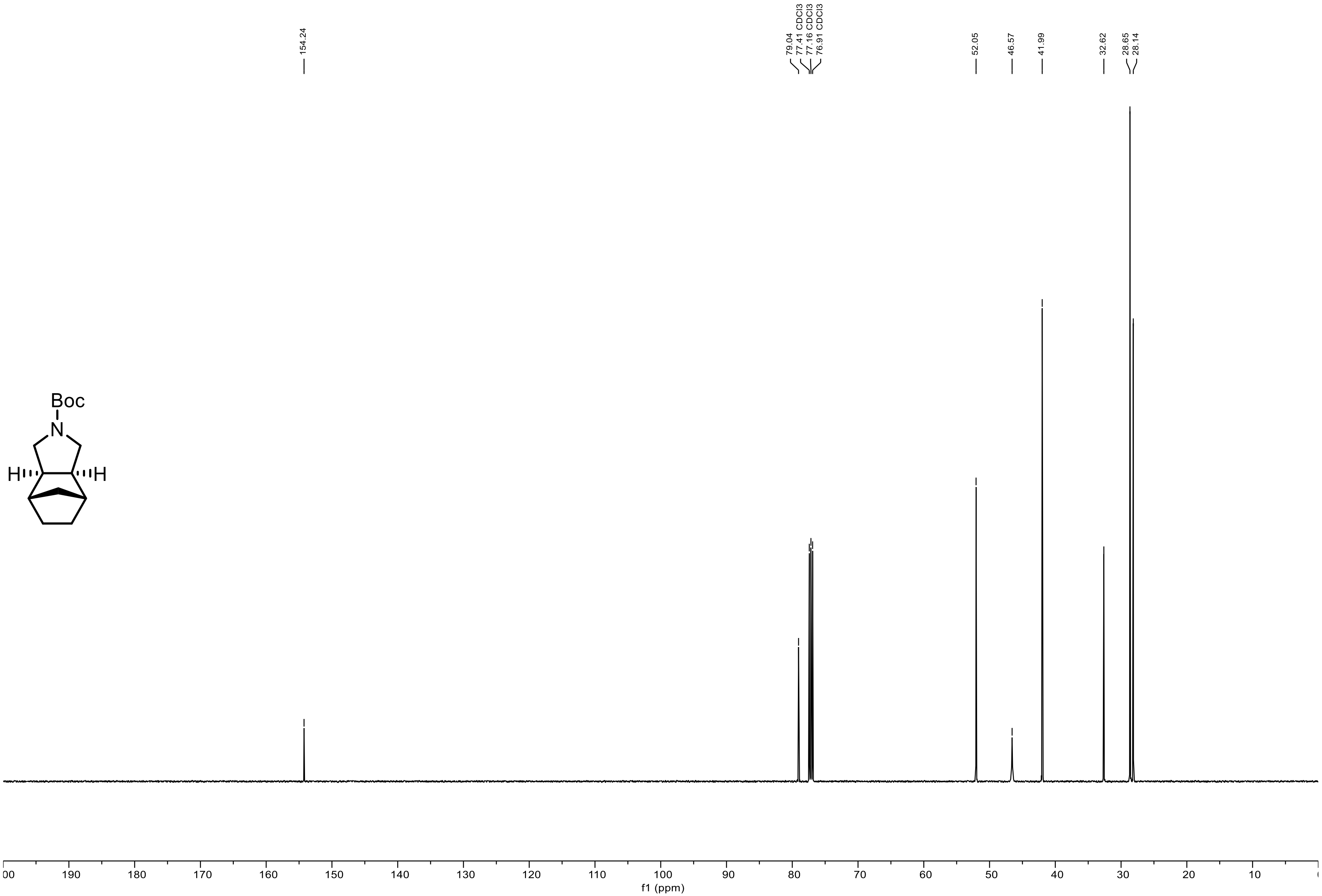

S8 ^{13}C NMR (126 MHz, $CDCl_3$).

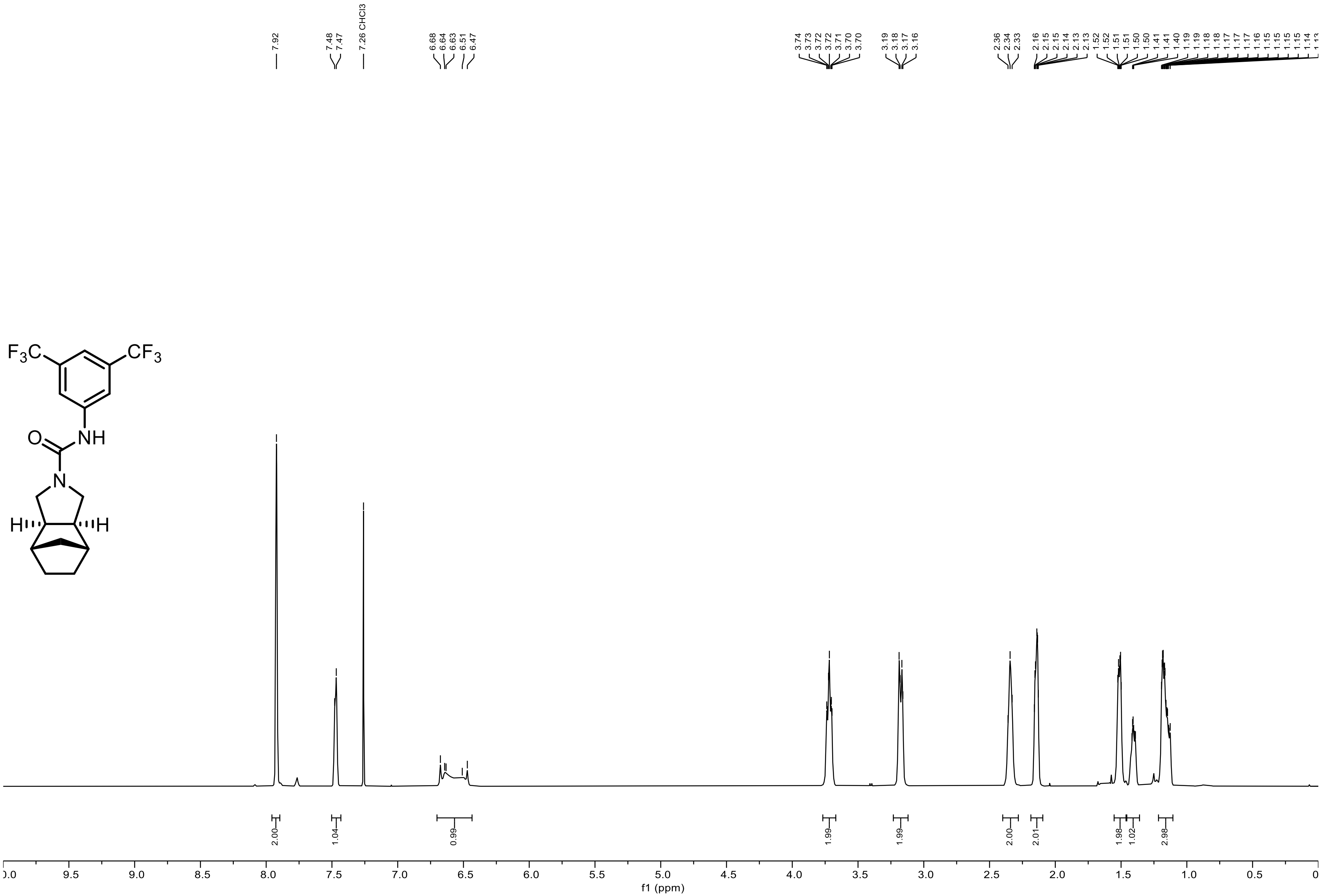

S9 ^{1}H NMR (500 MHz, $CDCl_3$).

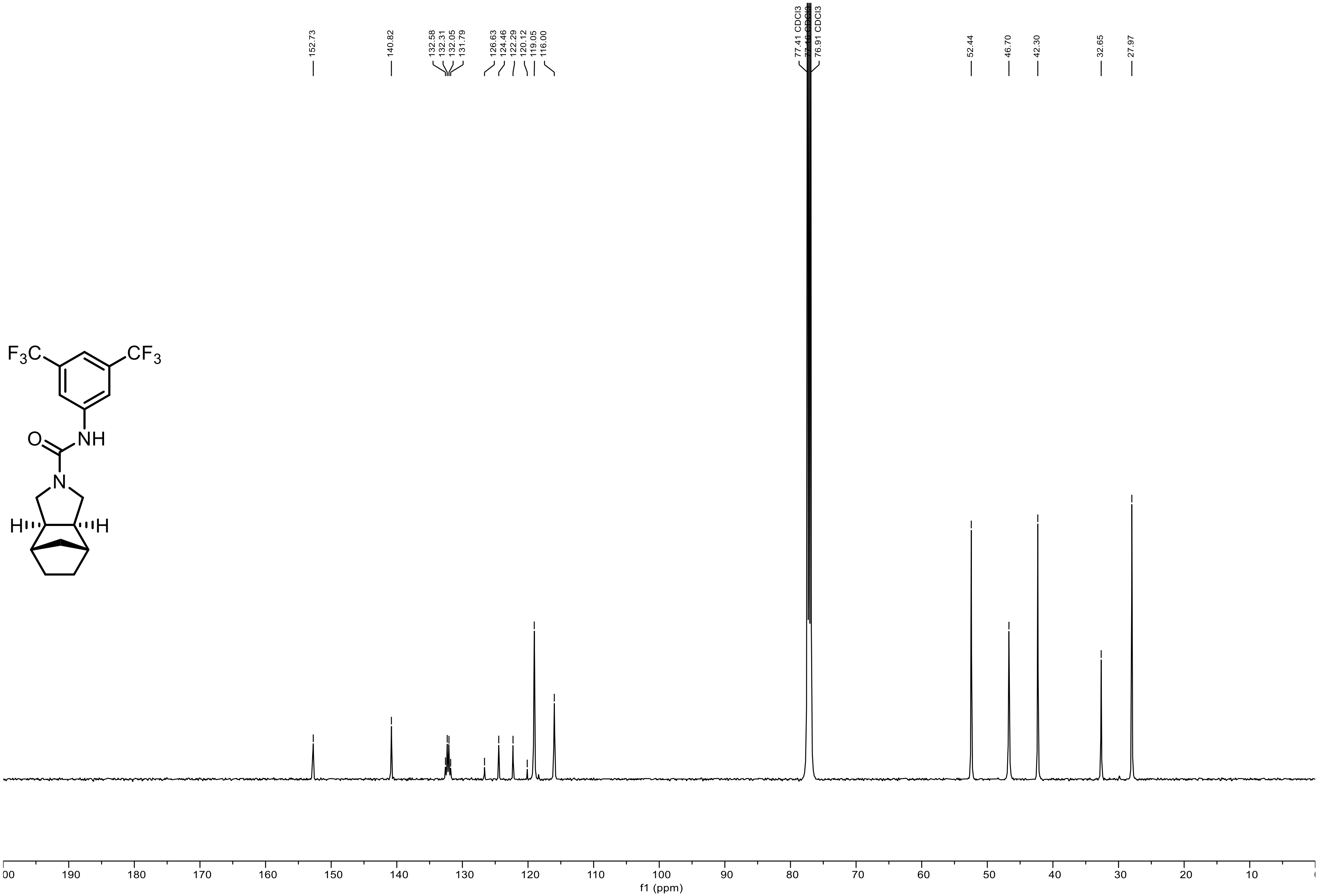

S9 ^{13}C NMR (126 MHz, $CDCl_3$).

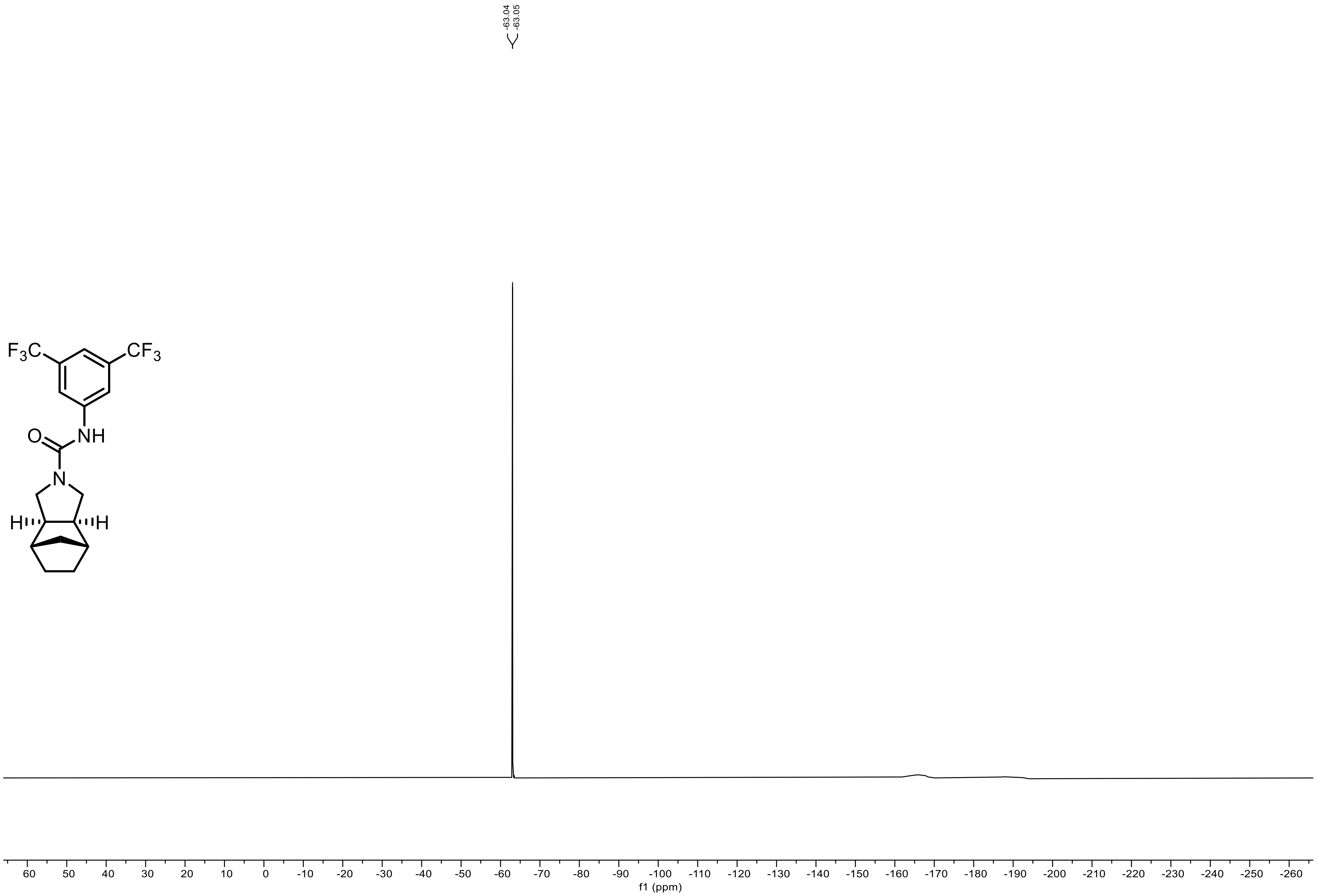

S9 ^{19}F NMR (470 MHz, $CDCl_3$).

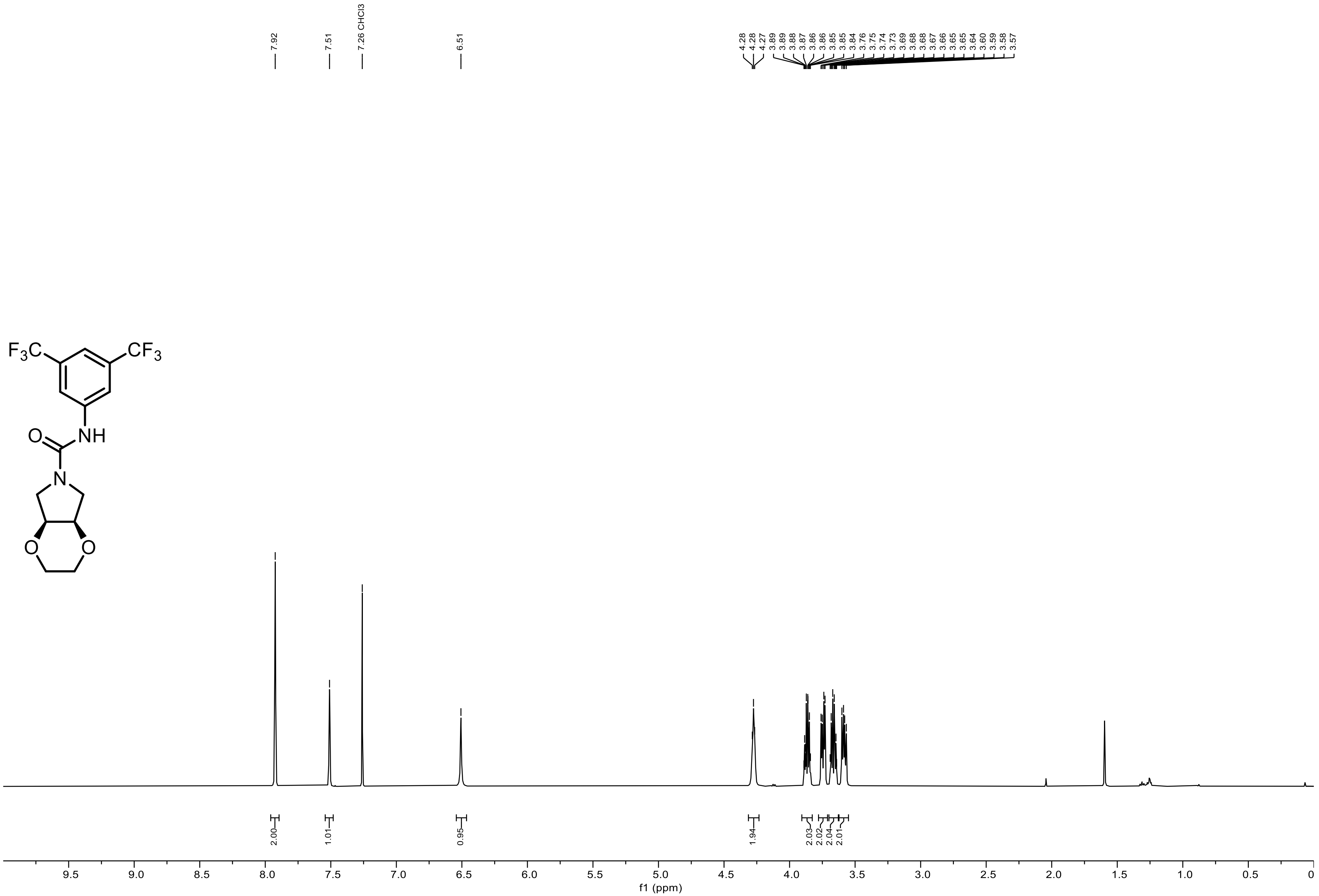

S10 ^{1}H NMR (500 MHz, $CDCl_3$).

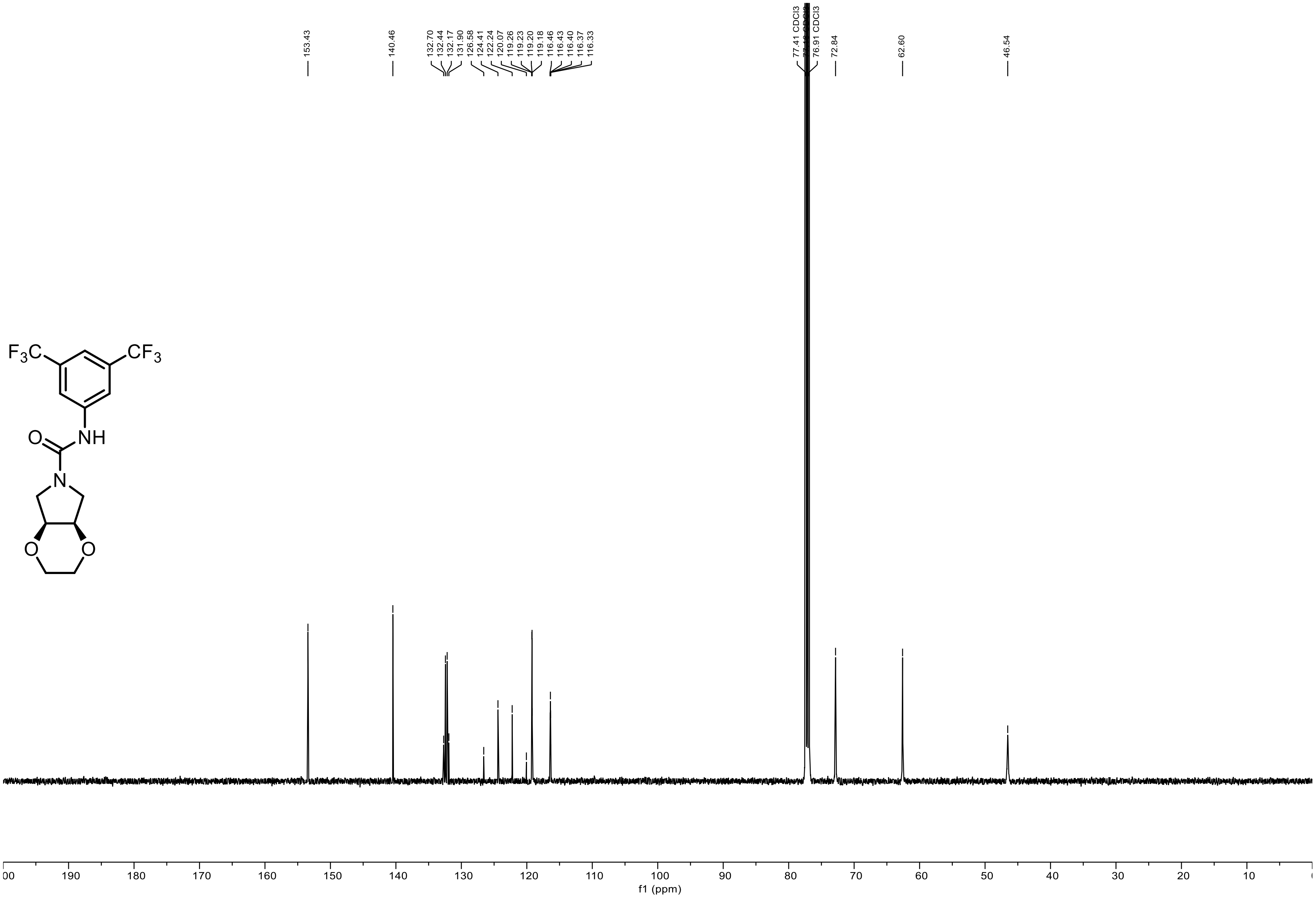

S10 ^{13}C NMR (126 MHz, $CDCl_3$).

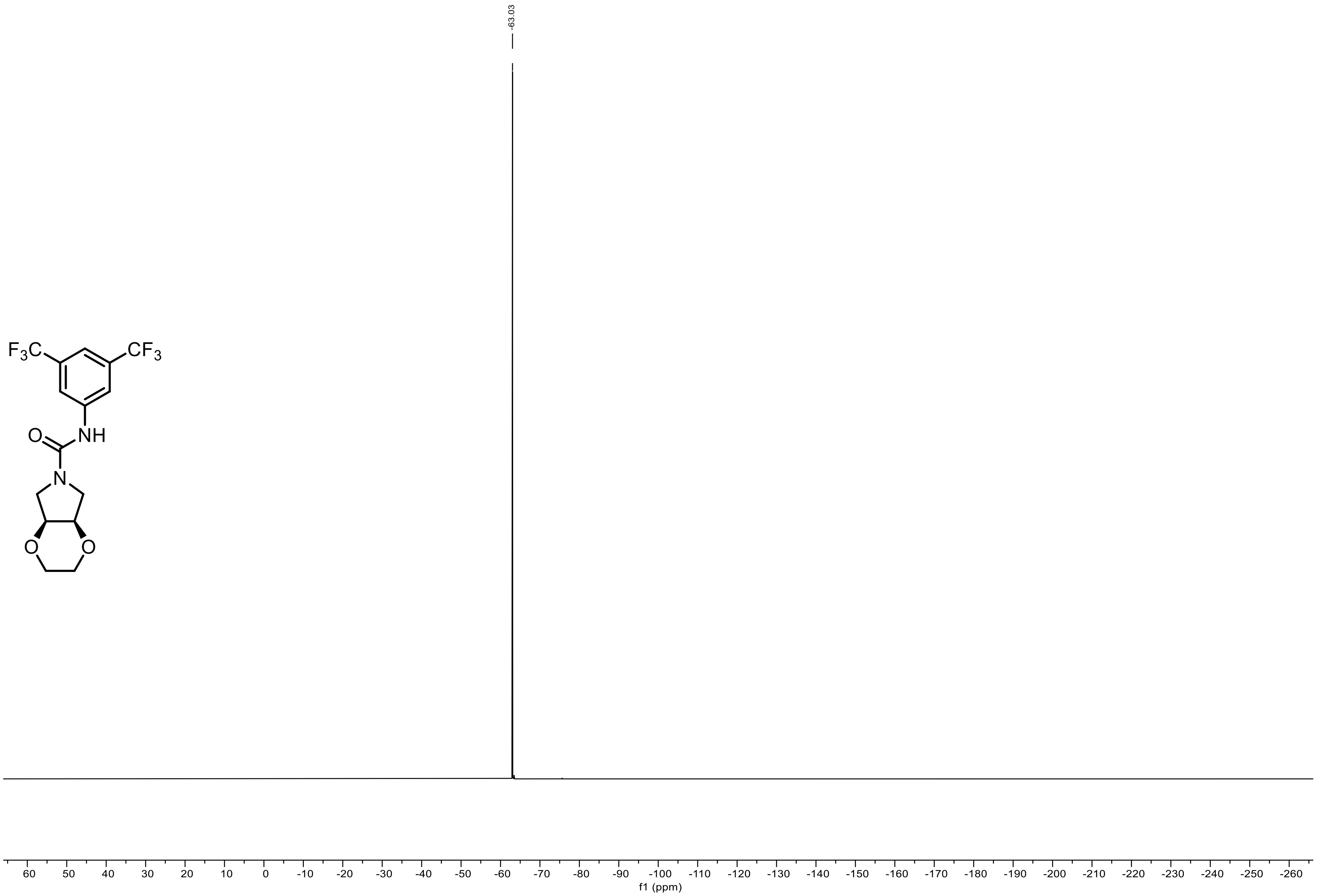

S10 ^{19}F NMR (470 MHz, $CDCl_3$).

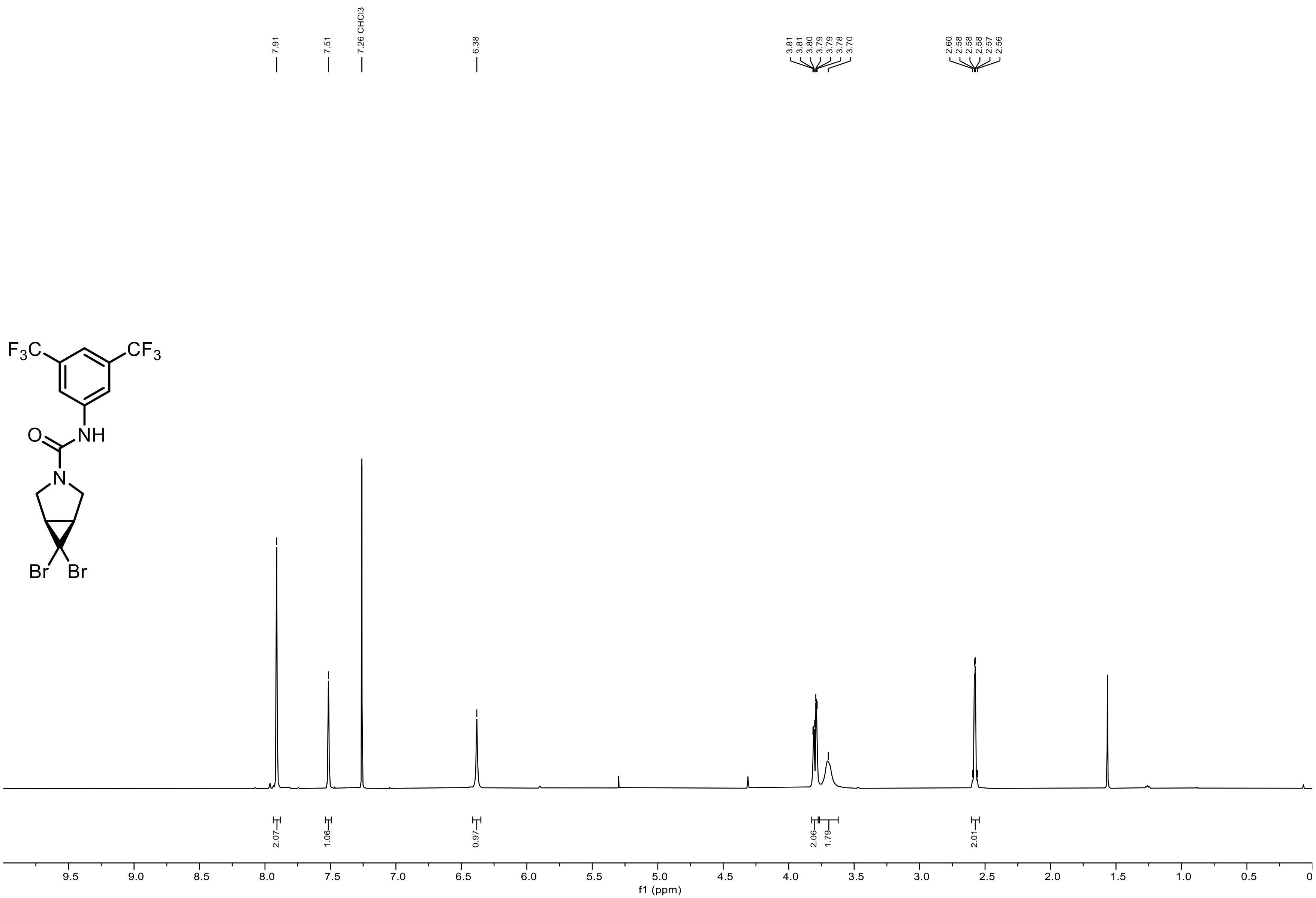

S11 ^{1}H NMR (500 MHz, $CDCl_3$).

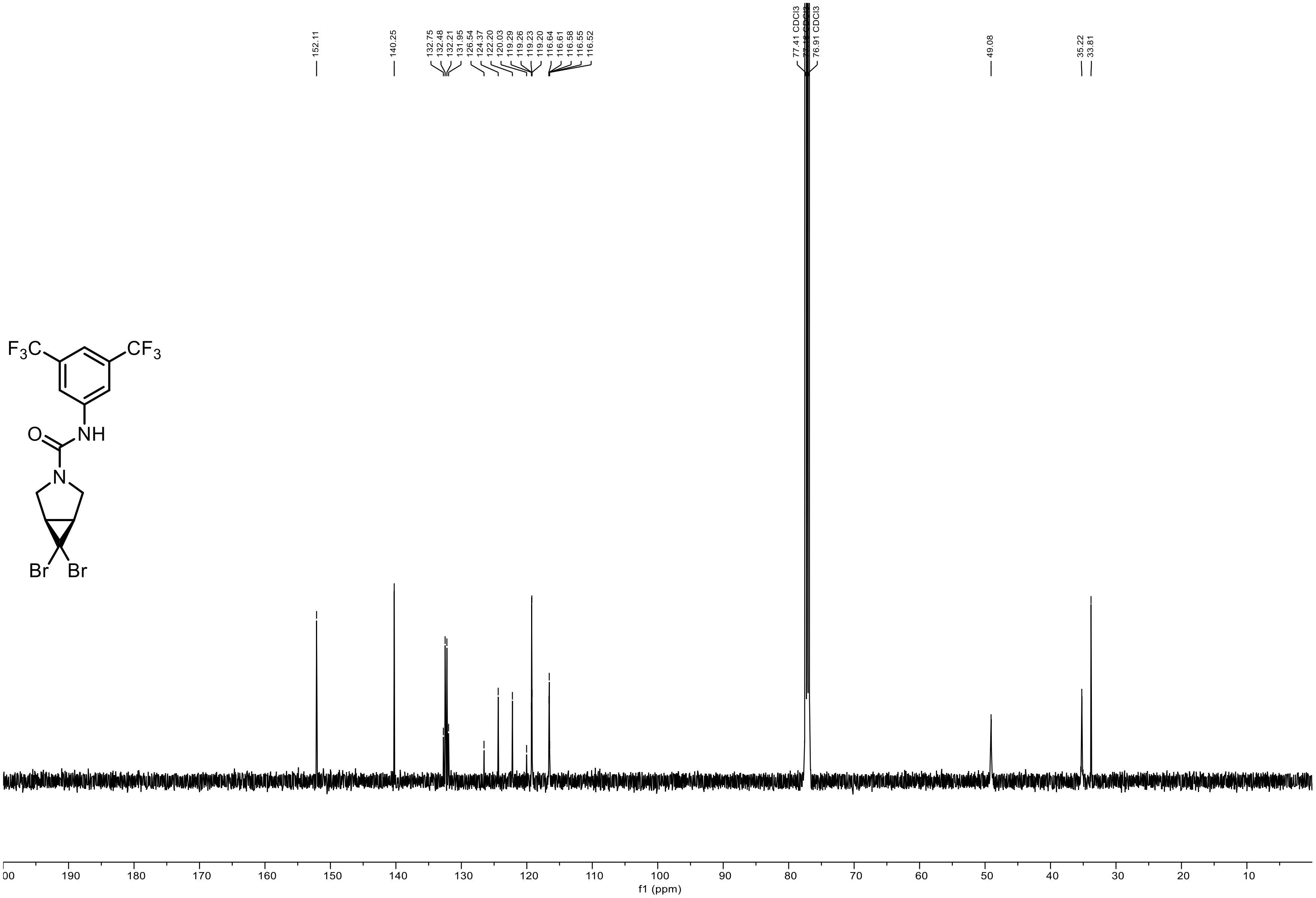

S11 ^{13}C NMR (126 MHz, $CDCl_3$).

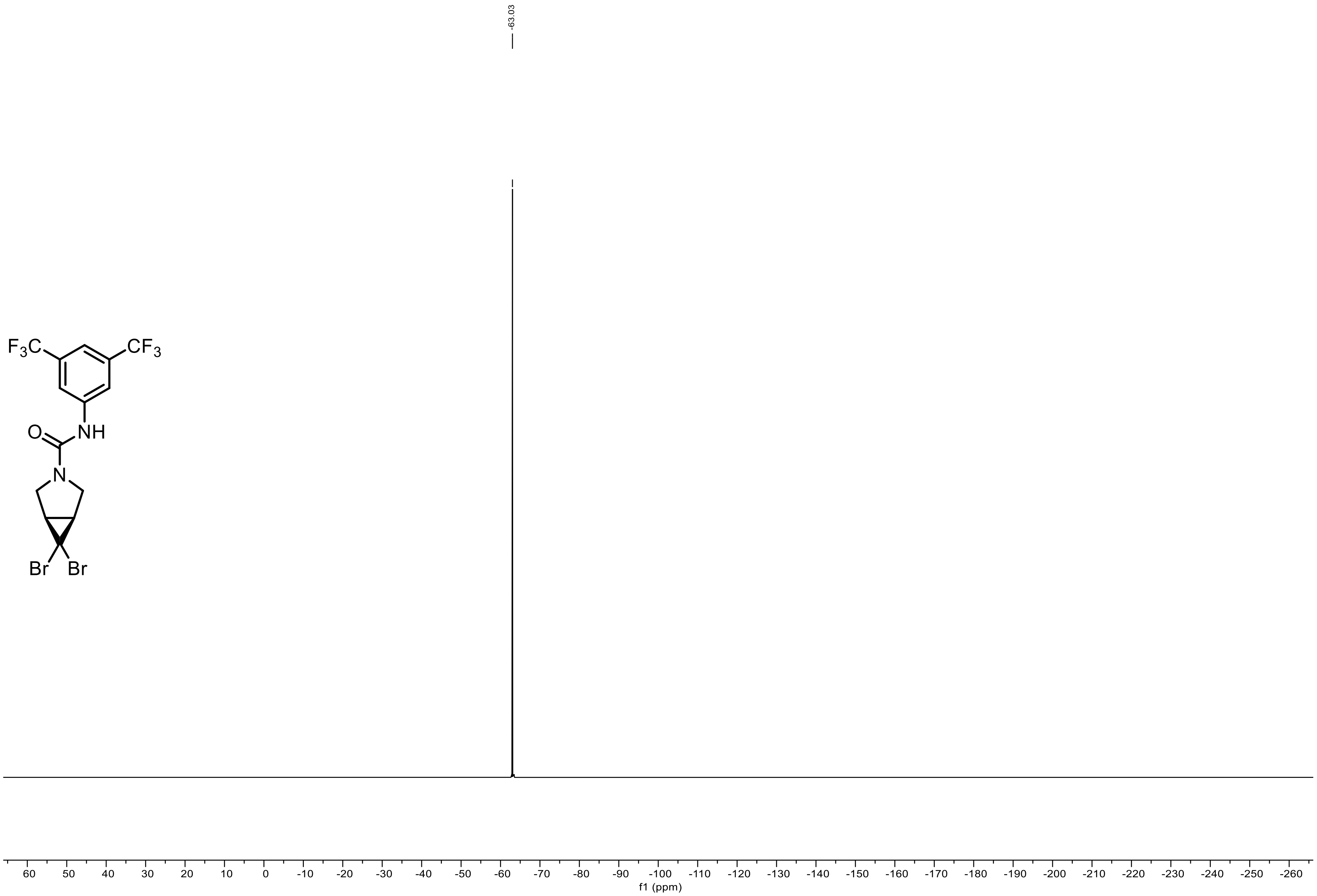

S11 ^{19}F NMR (470 MHz, $CDCl_3$).

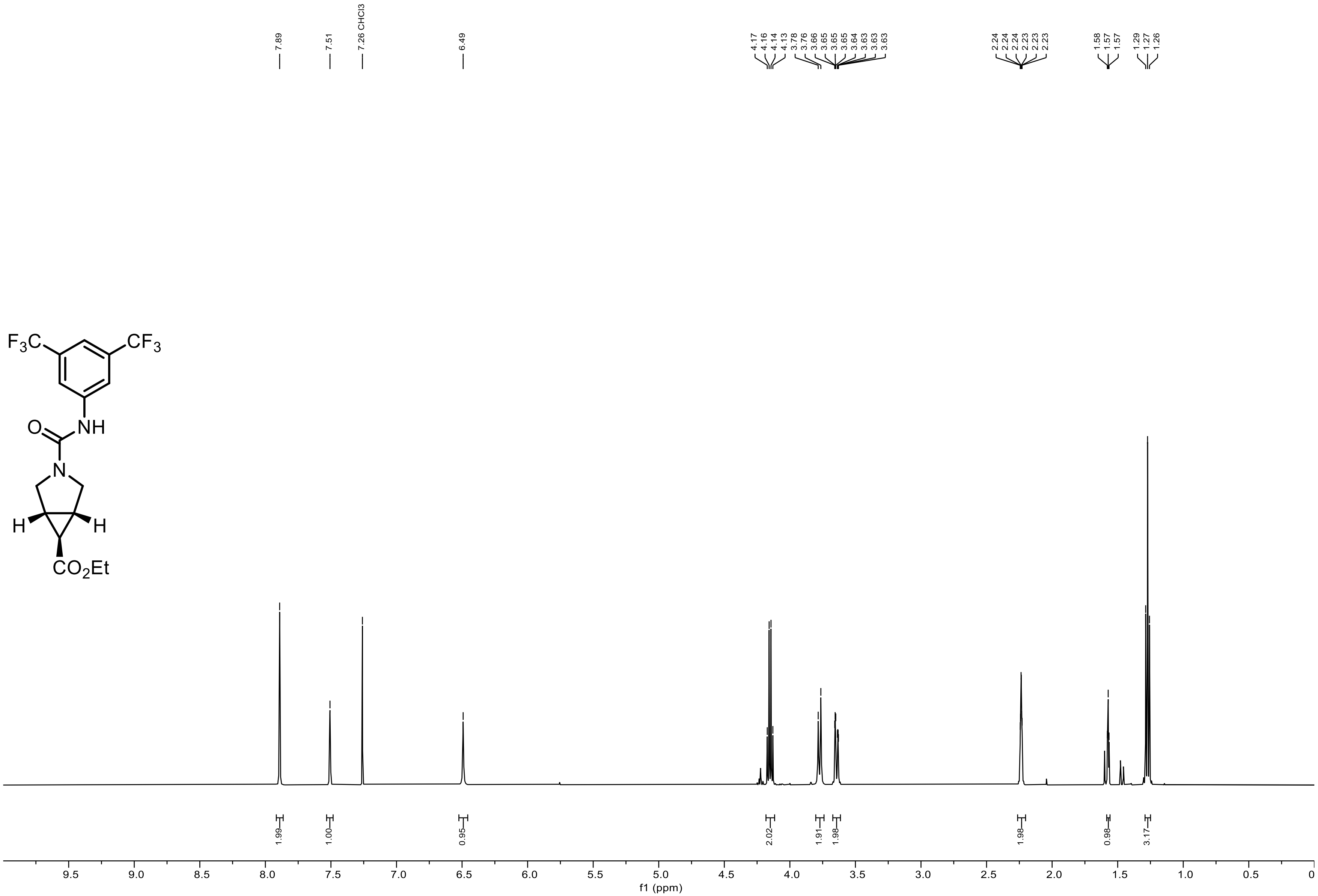

S12 ^{1}H NMR (500 MHz, $CDCl_3$).

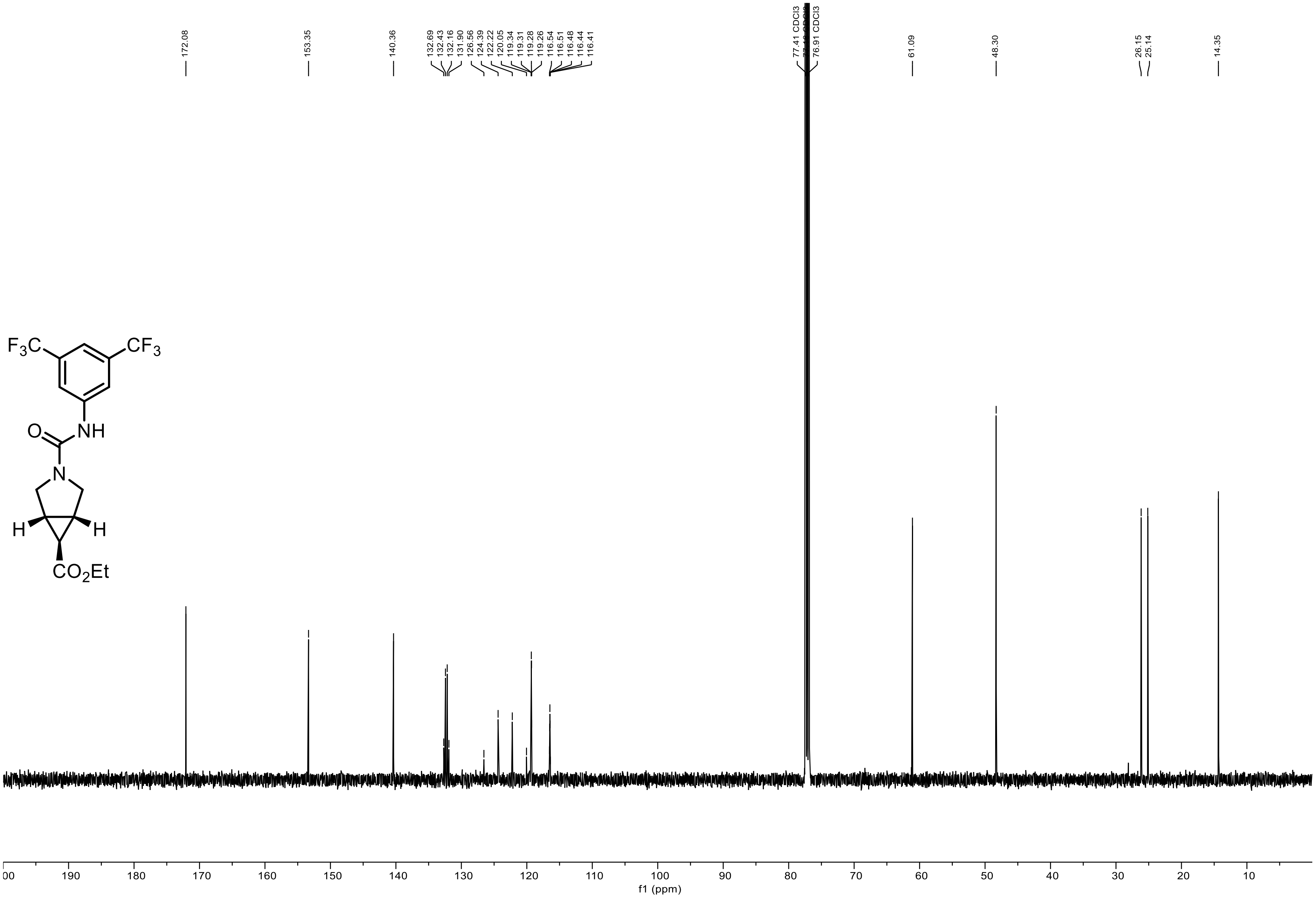

S12 ^{13}C NMR (126 MHz, $CDCl_3$).

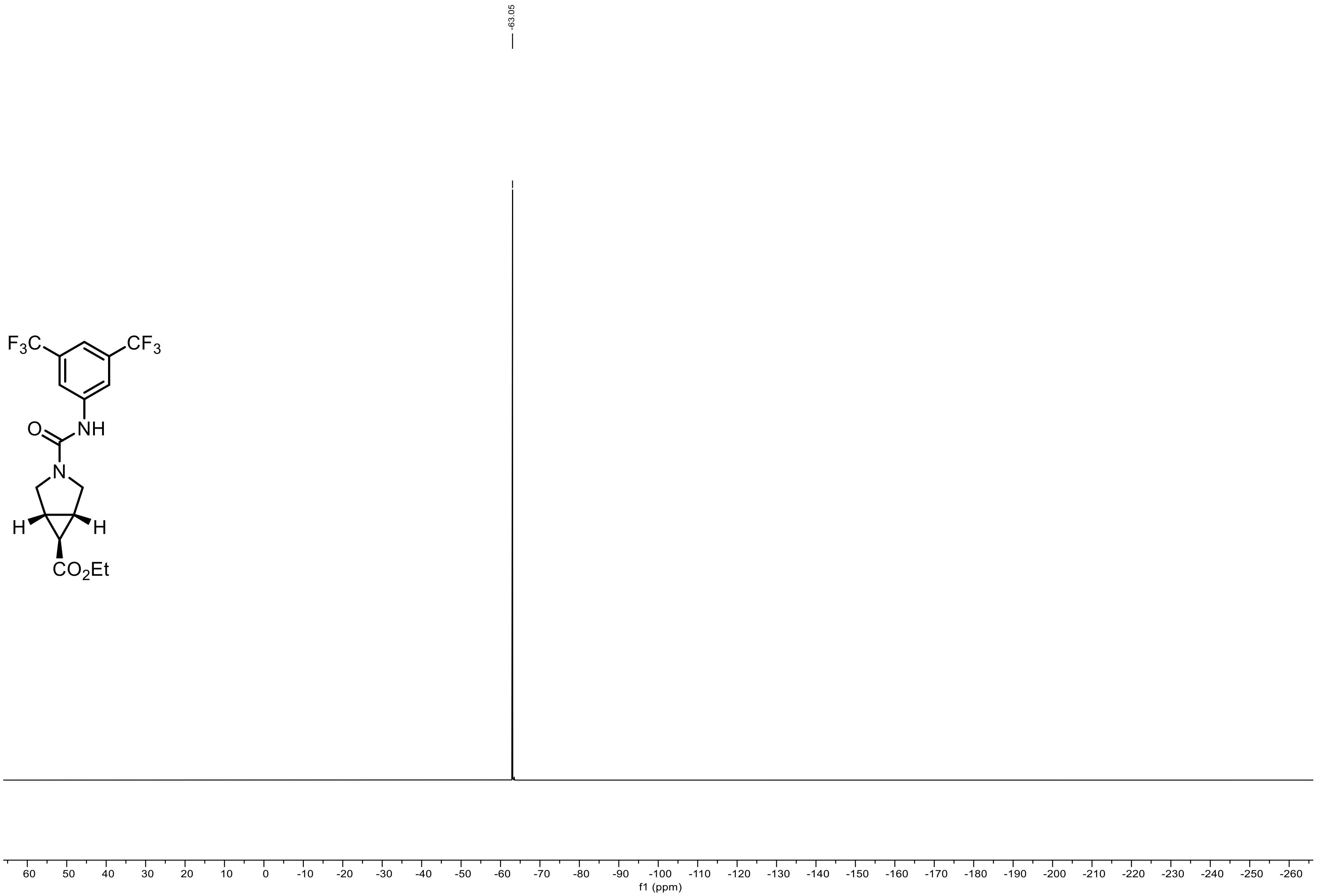

S12 ^{19}F NMR (470 MHz, $CDCl_3$).

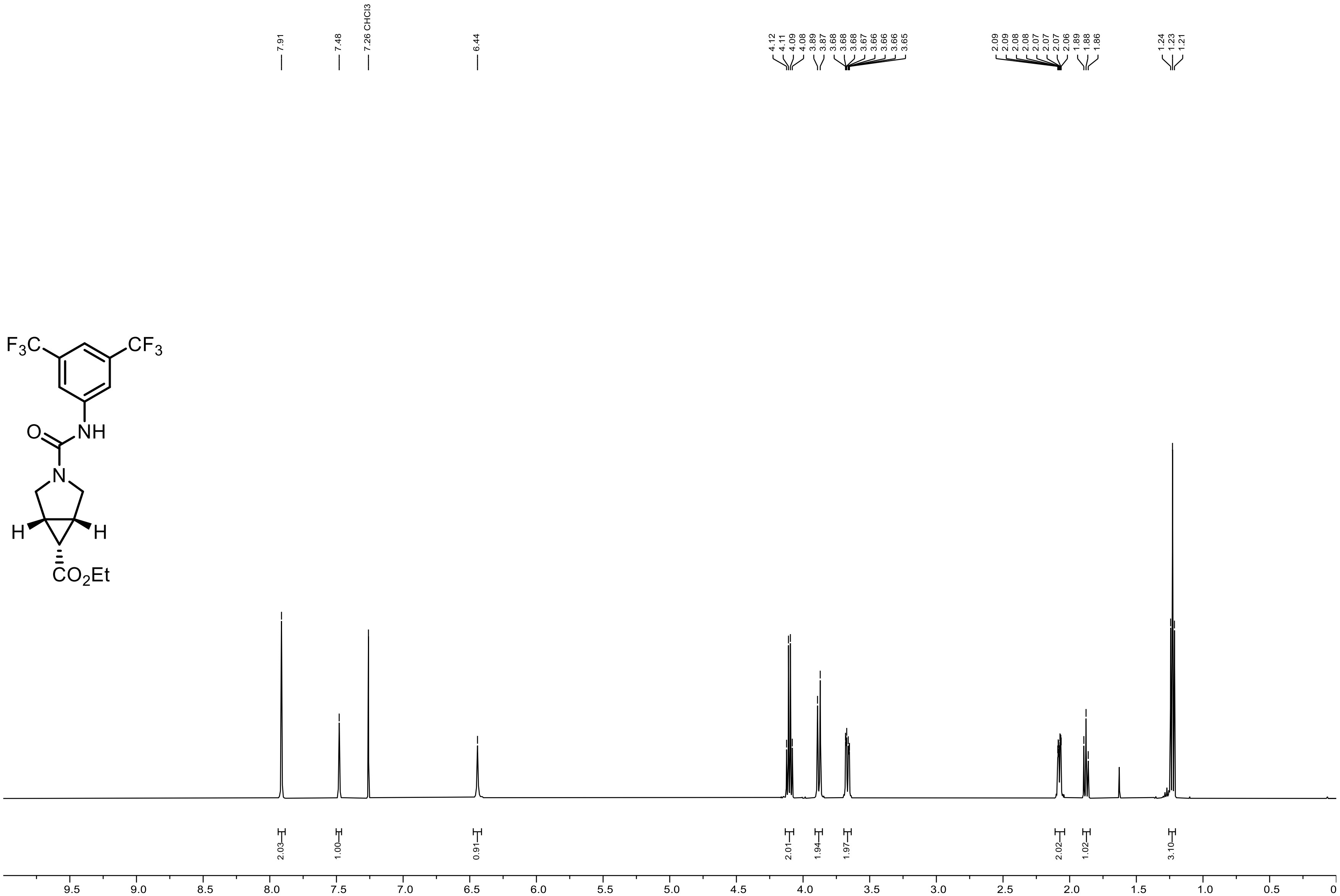

S13 ^{1}H NMR (500 MHz, $CDCl_3$).

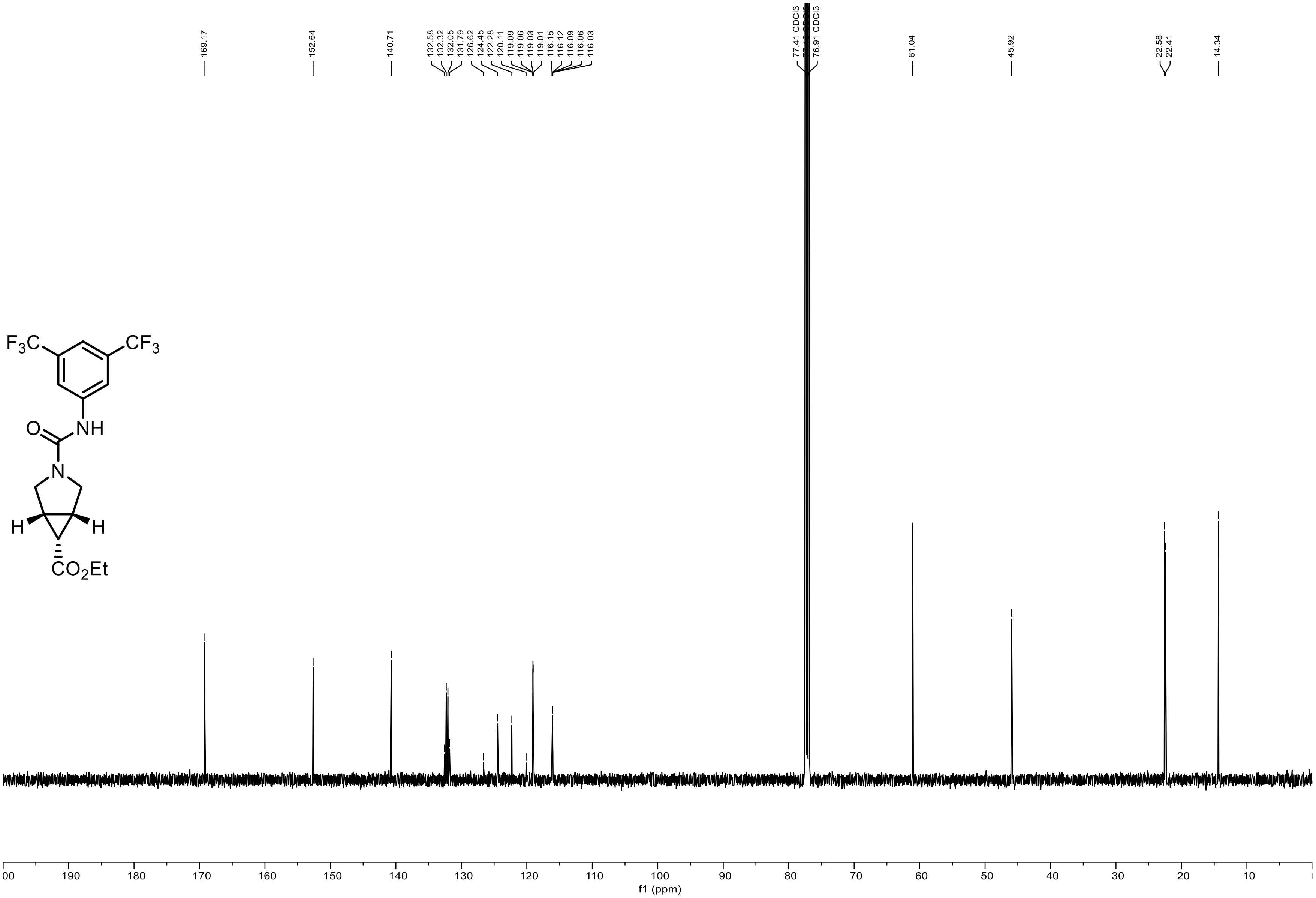

S13 ^{13}C NMR (126 MHz, $CDCl_3$).

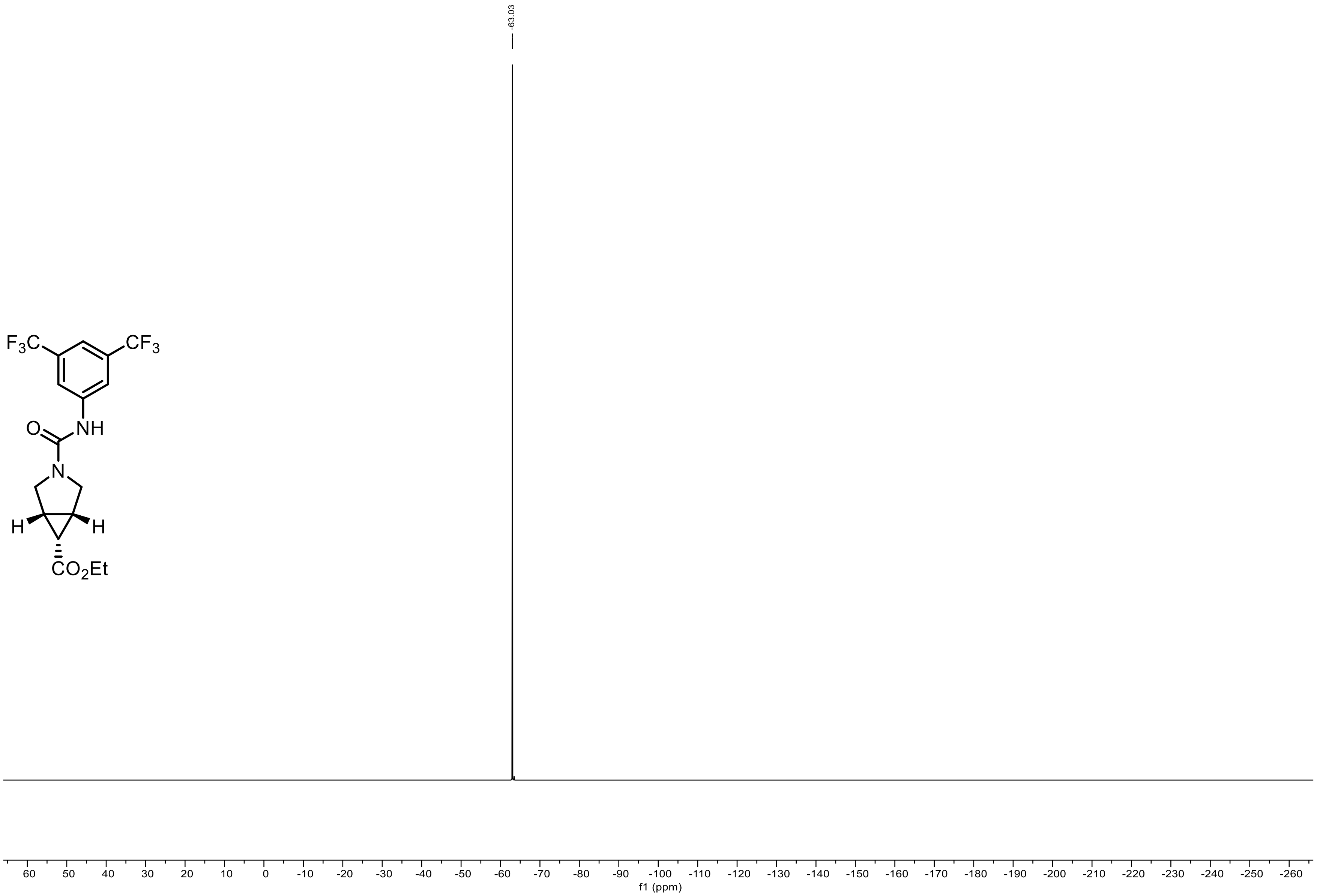

S13 ^{19}F NMR (470 MHz, $CDCl_3$).

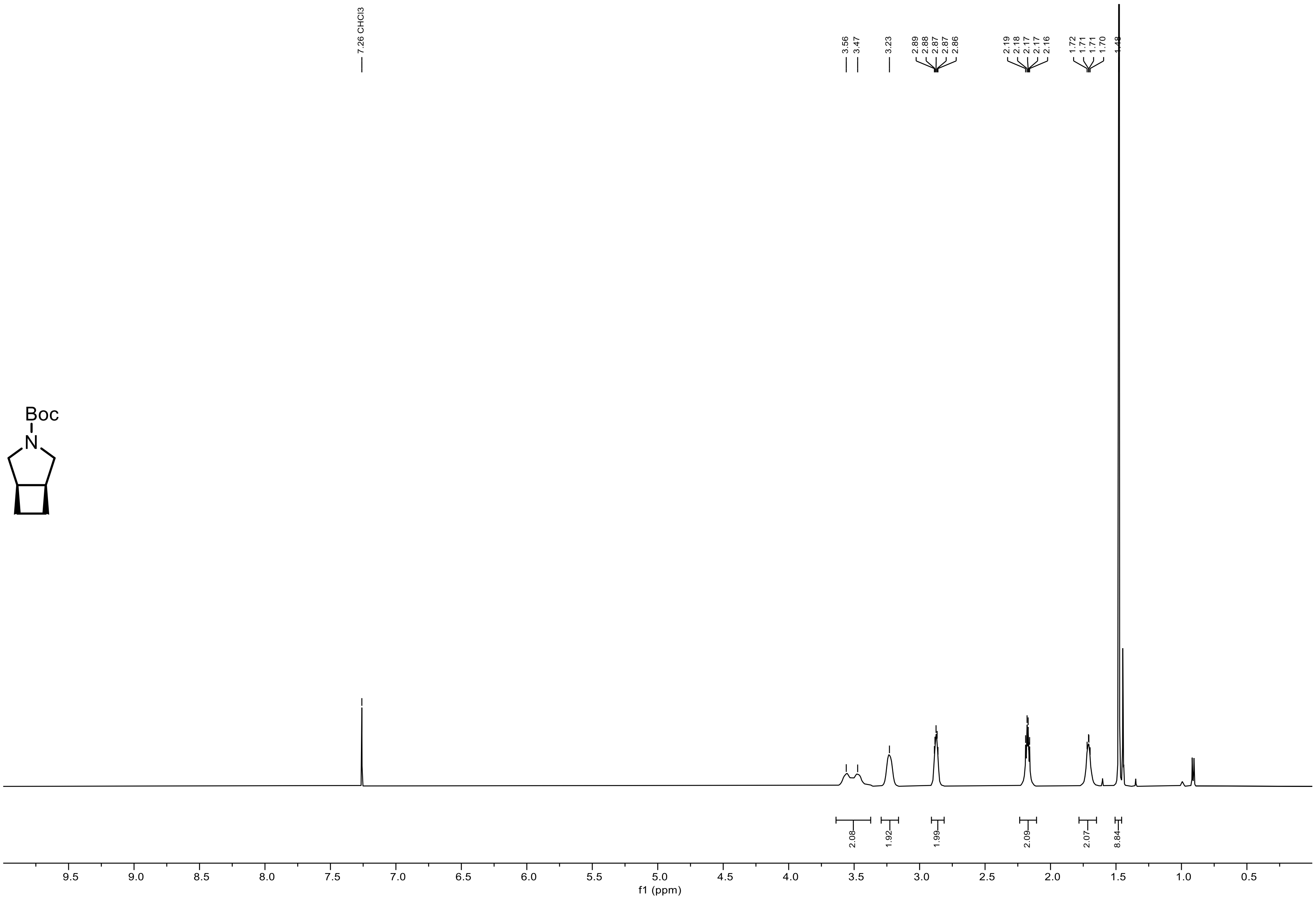

S14 ^{1}H NMR (500 MHz, $CDCl_3$).

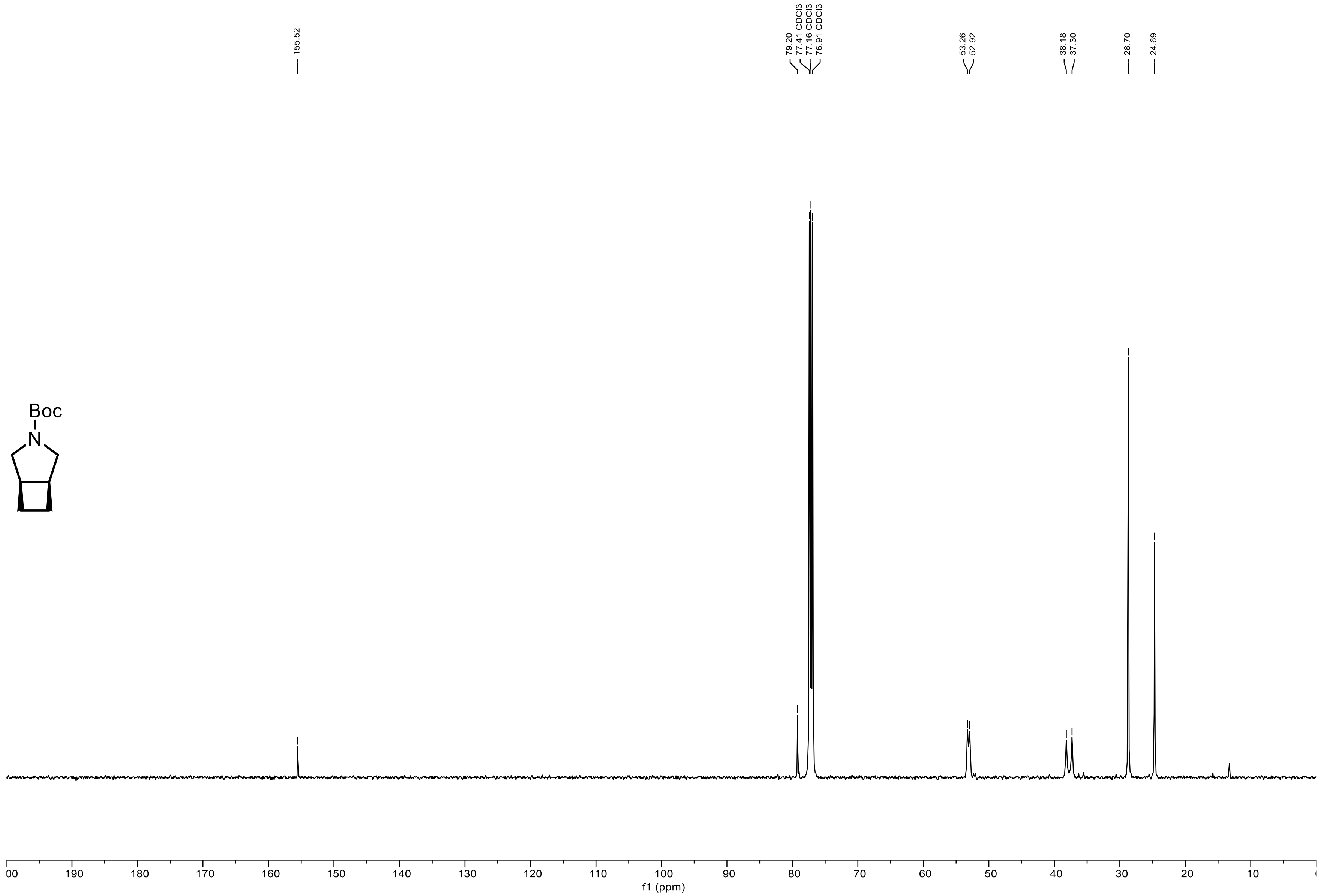

S14 ^{13}C NMR (126 MHz, $CDCl_3$).

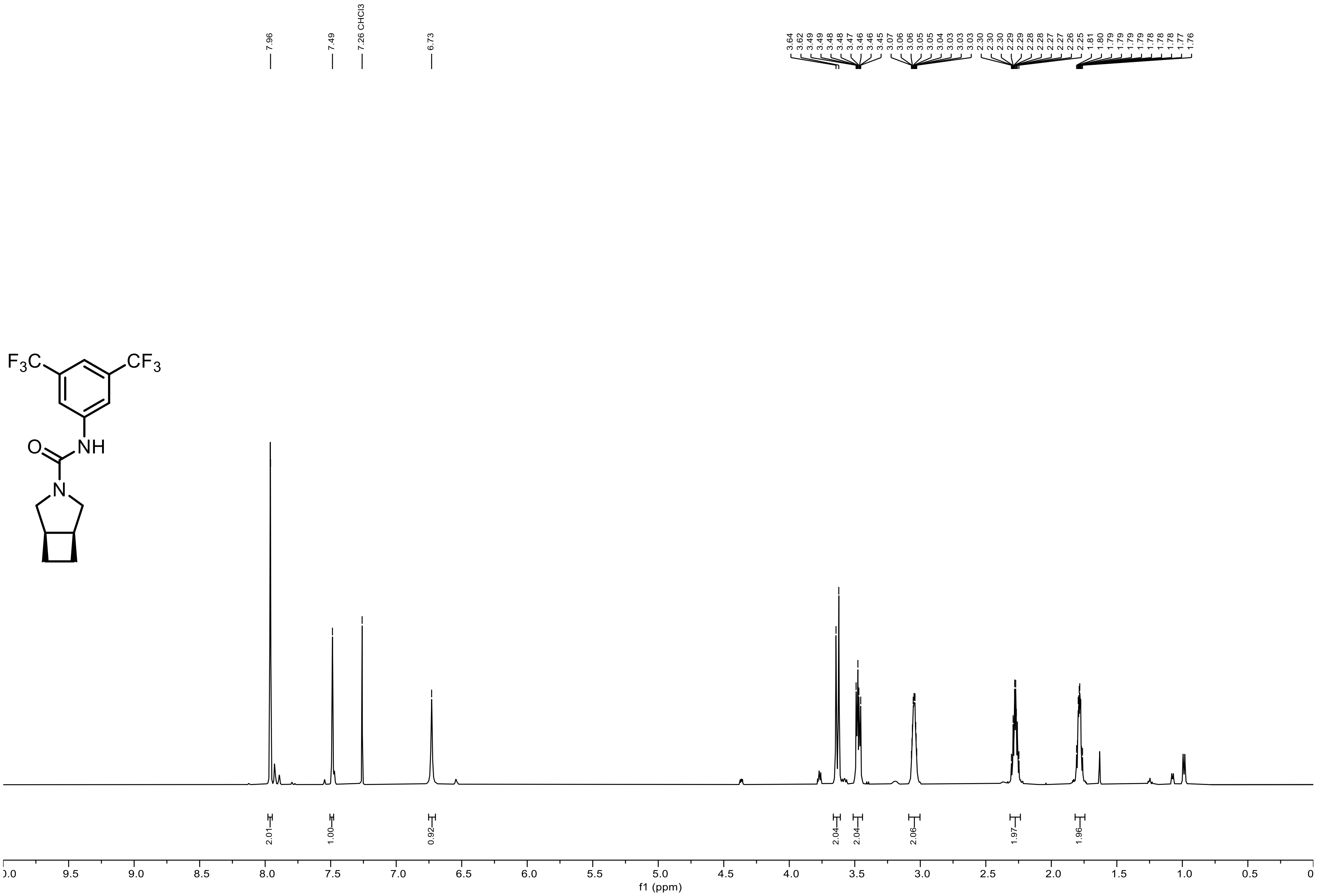

S14 ^{1}H NMR (500 MHz, $CDCl_3$).

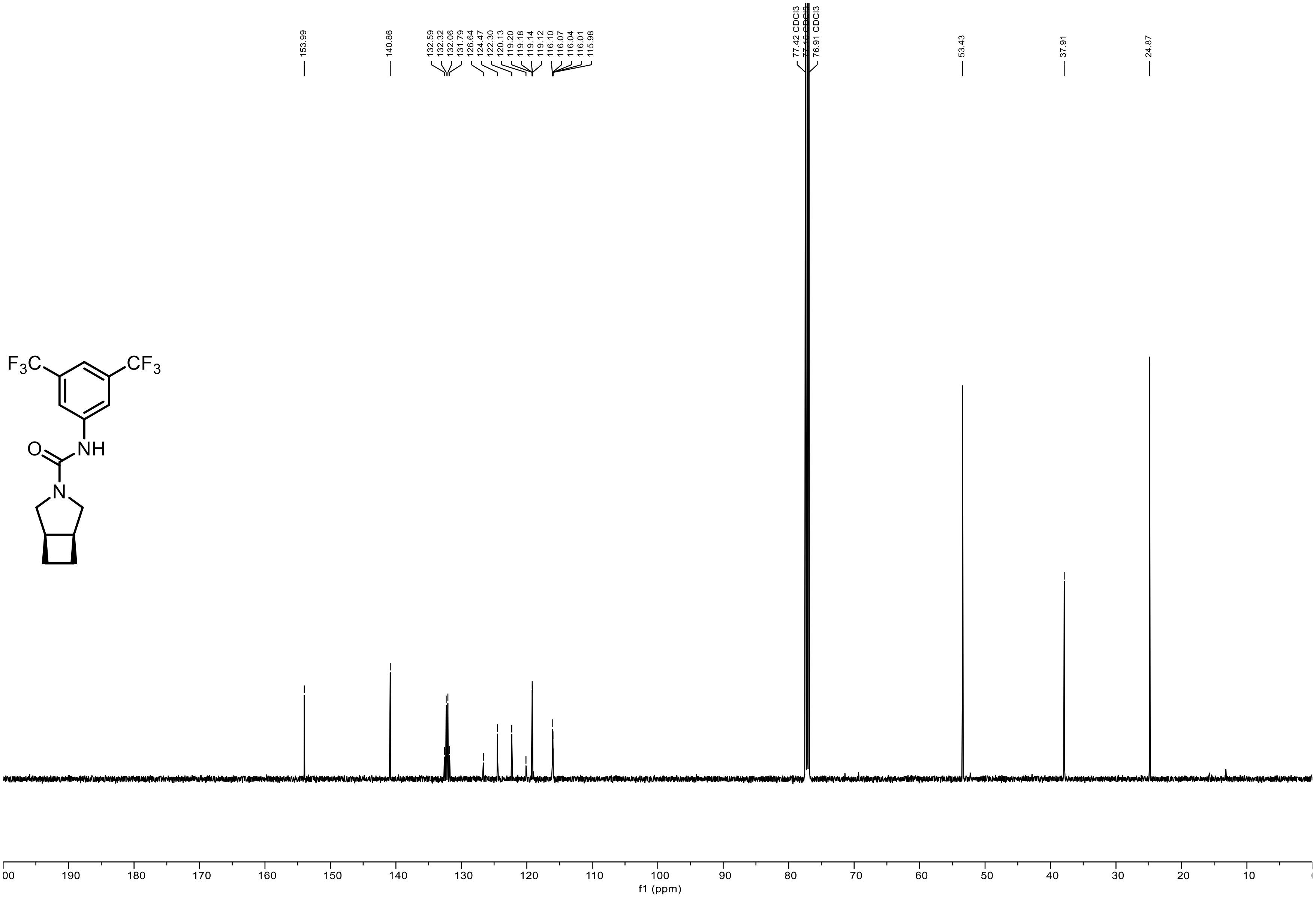

S15 ^{13}C NMR (126 MHz, $CDCl_3$).

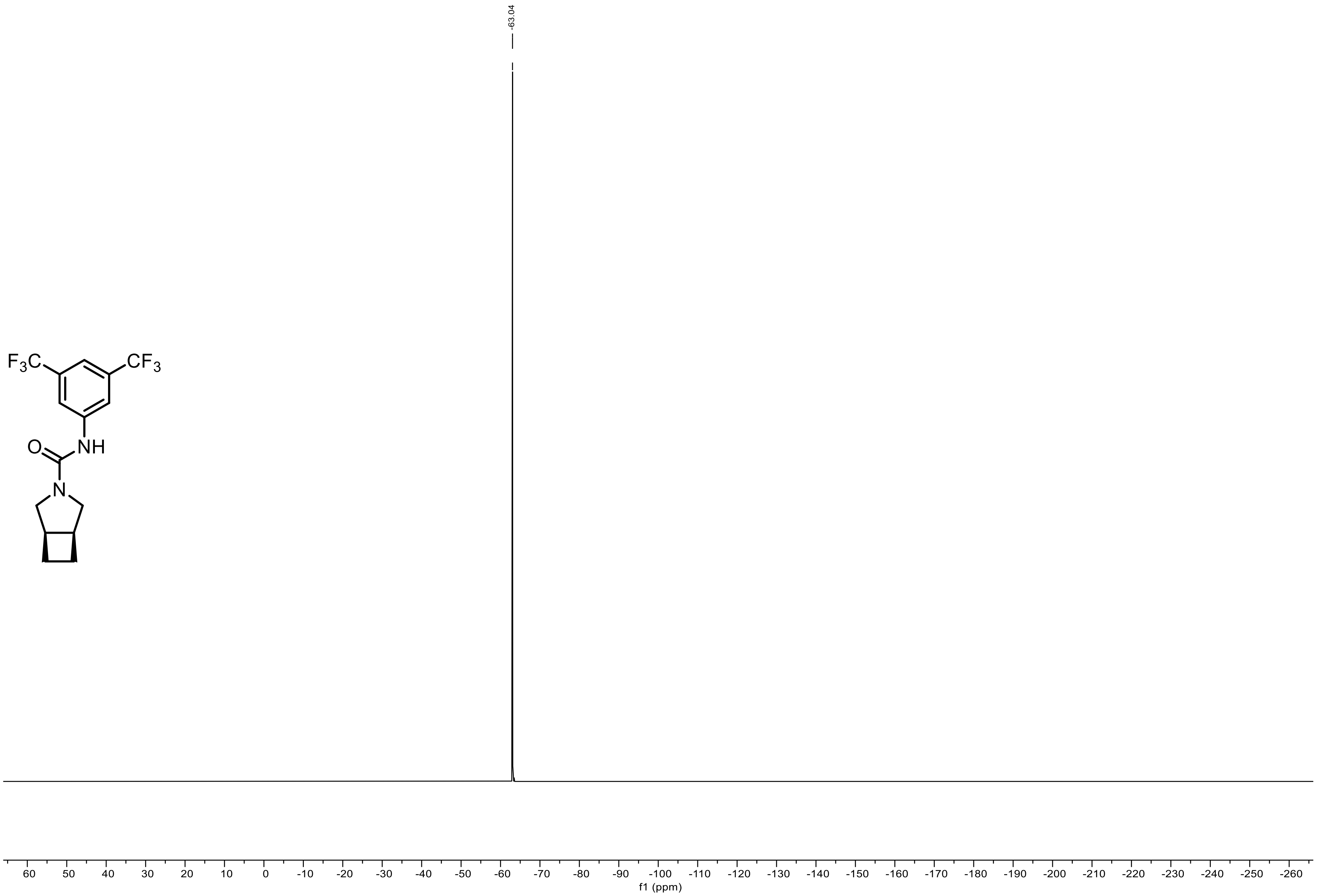

S15 ^{19}F NMR (470 MHz, $CDCl_3$).

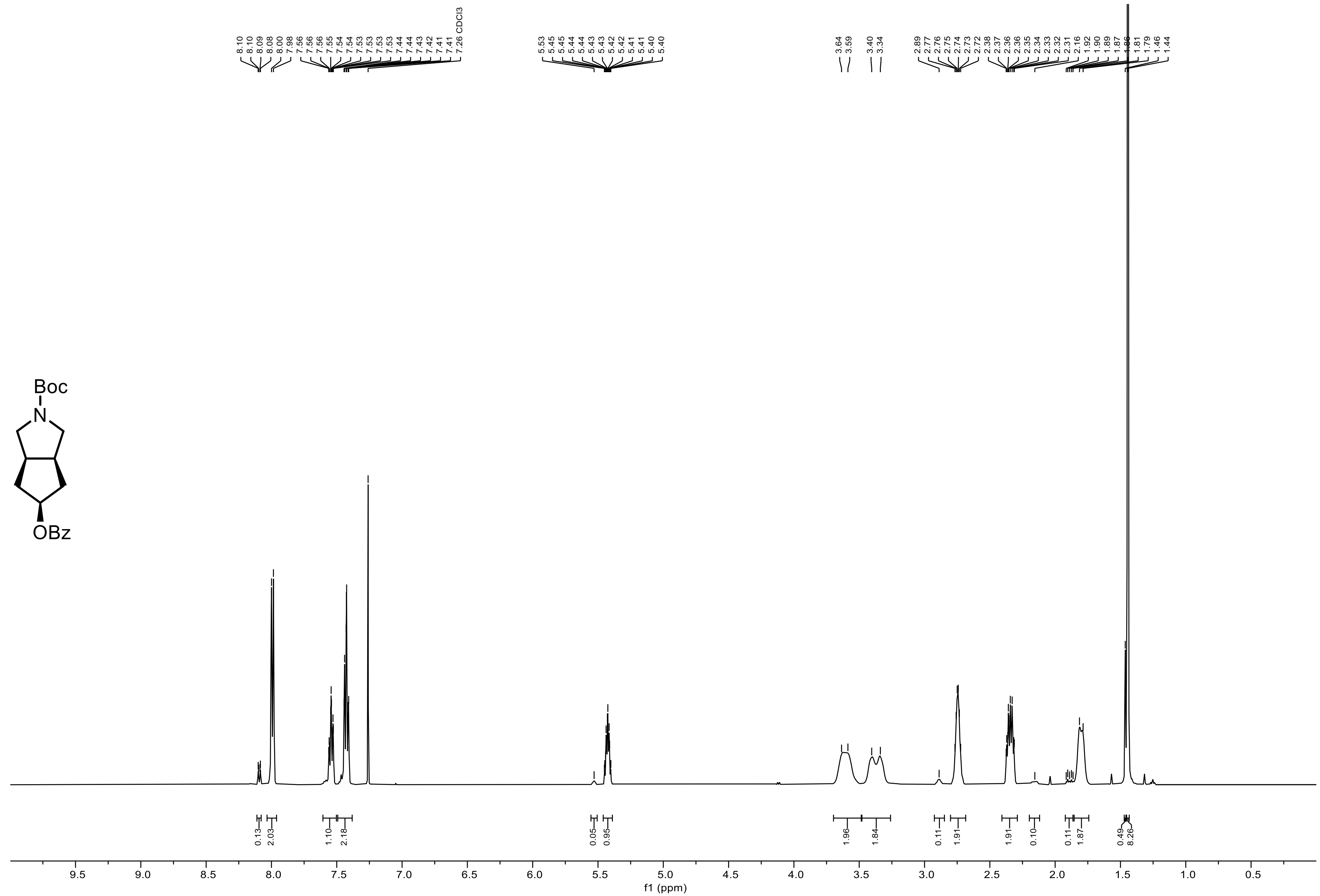

S16 ^{1}H NMR (500 MHz, $CDCl_3$).

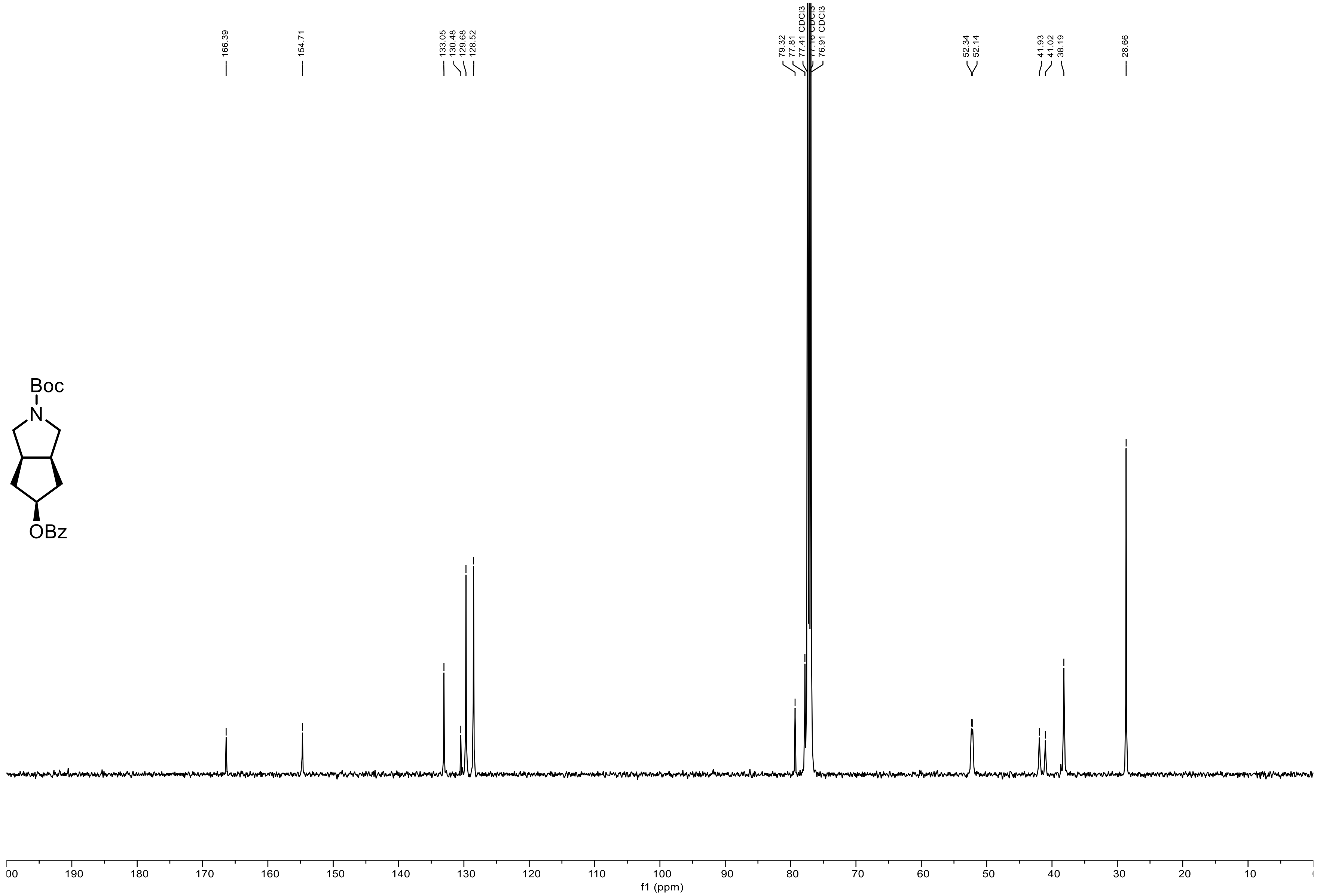

S16 ^{13}C NMR (126 MHz, $CDCl_3$).

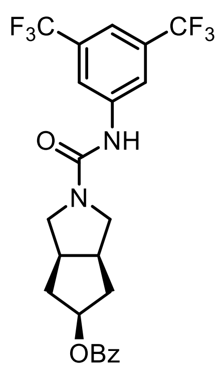

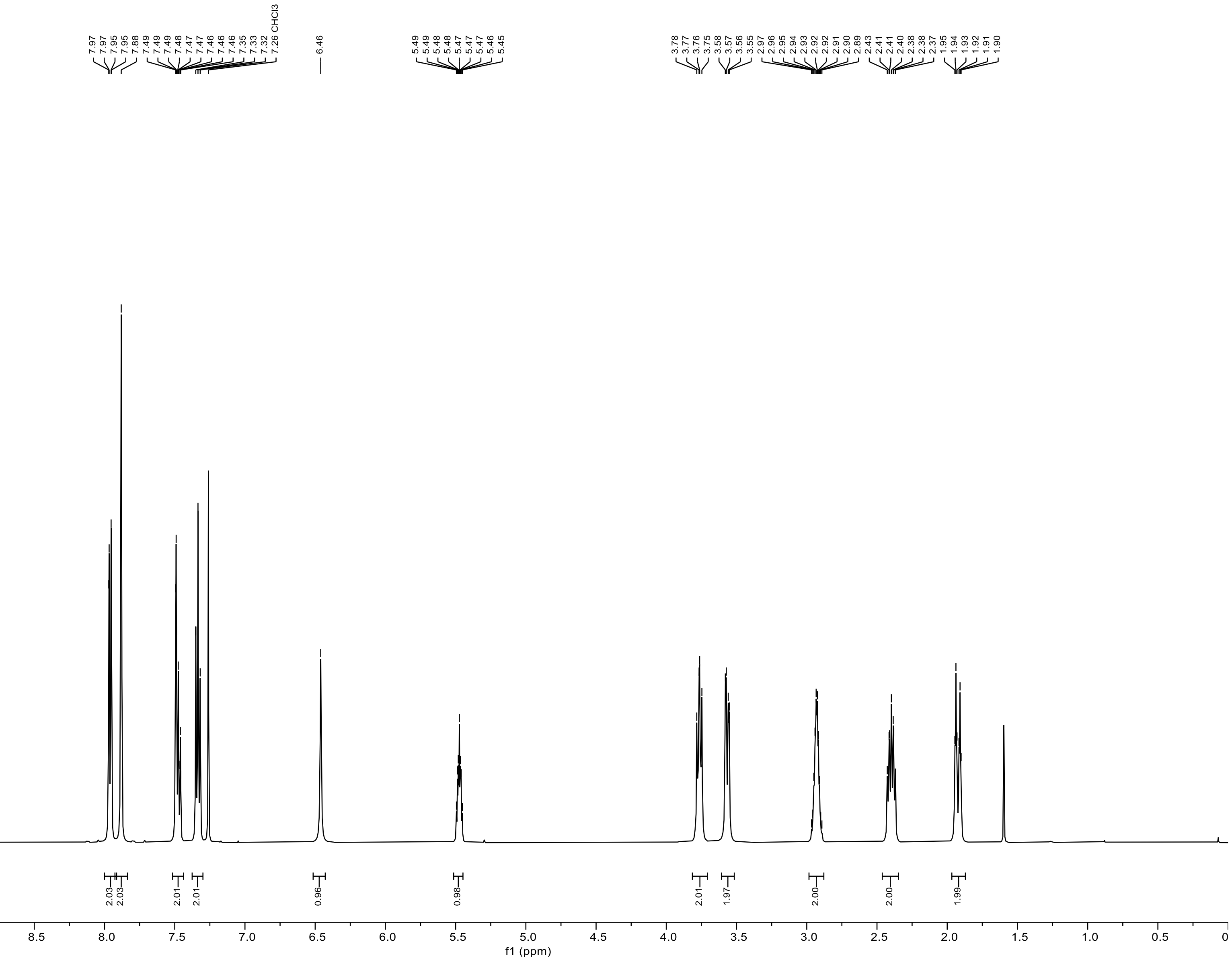

S17 ^{1}H NMR (500 MHz, $CDCl_3$).

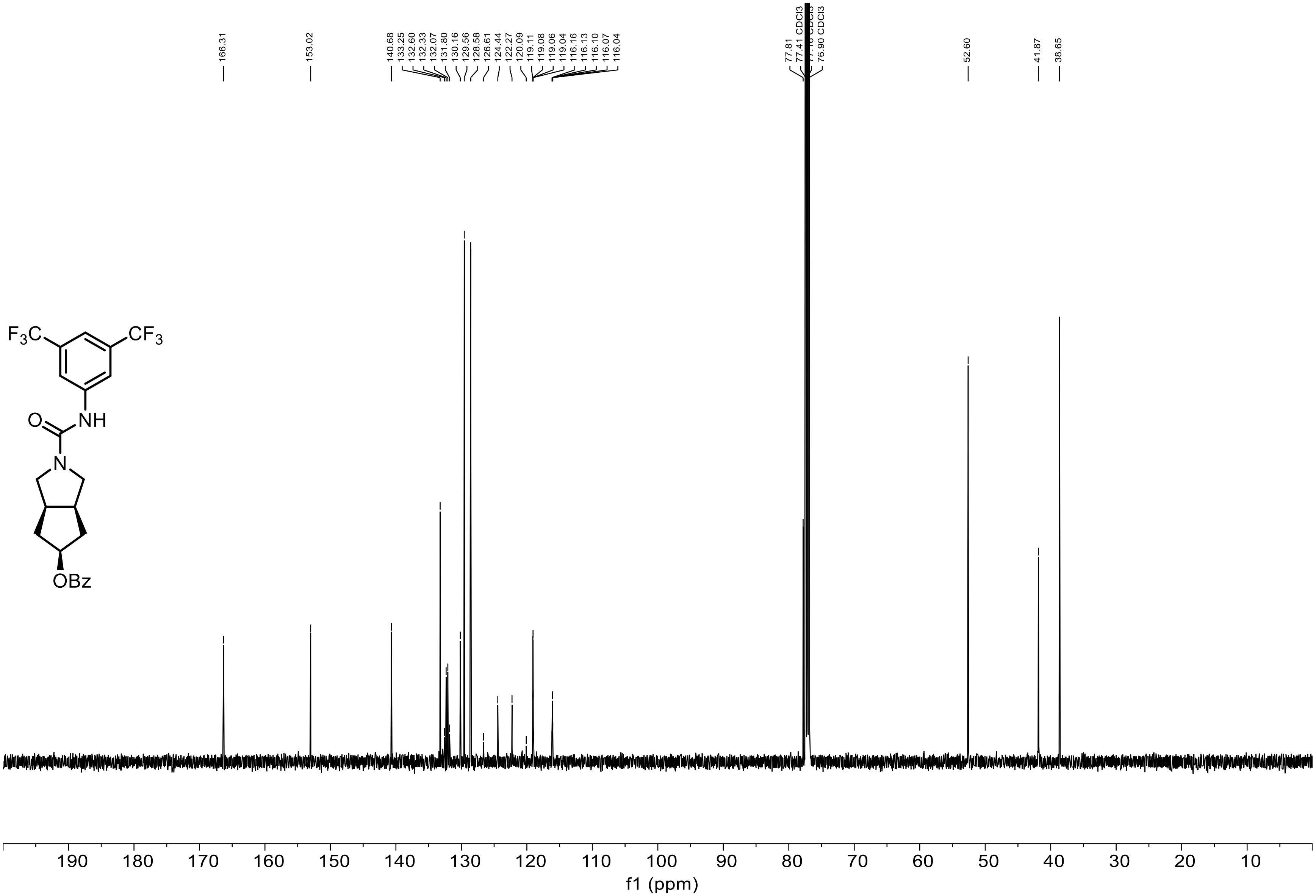

S17 ^{13}C NMR (126 MHz, $CDCl_3$).

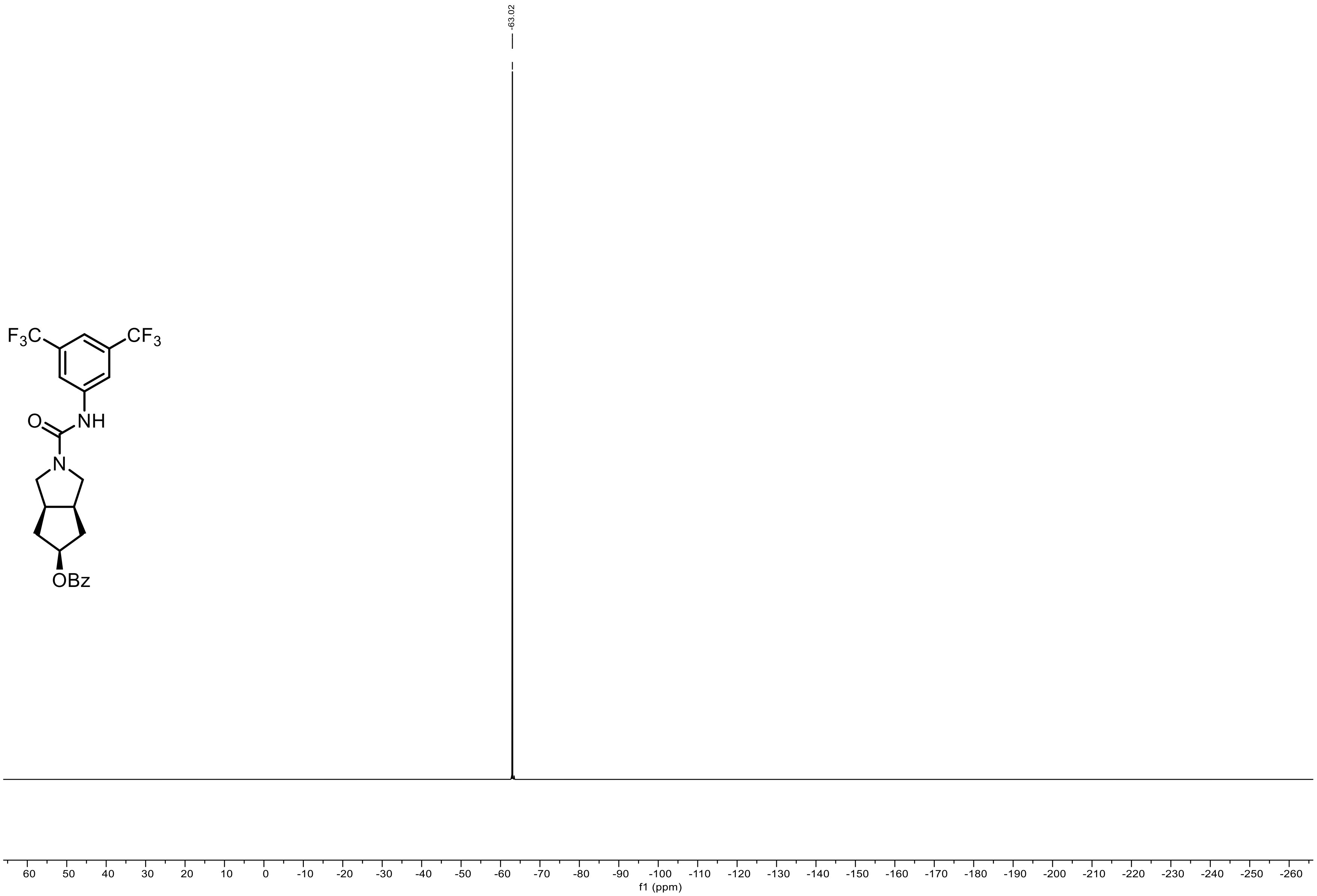

S17 ^{19}F NMR (470 MHz, $CDCl_3$).

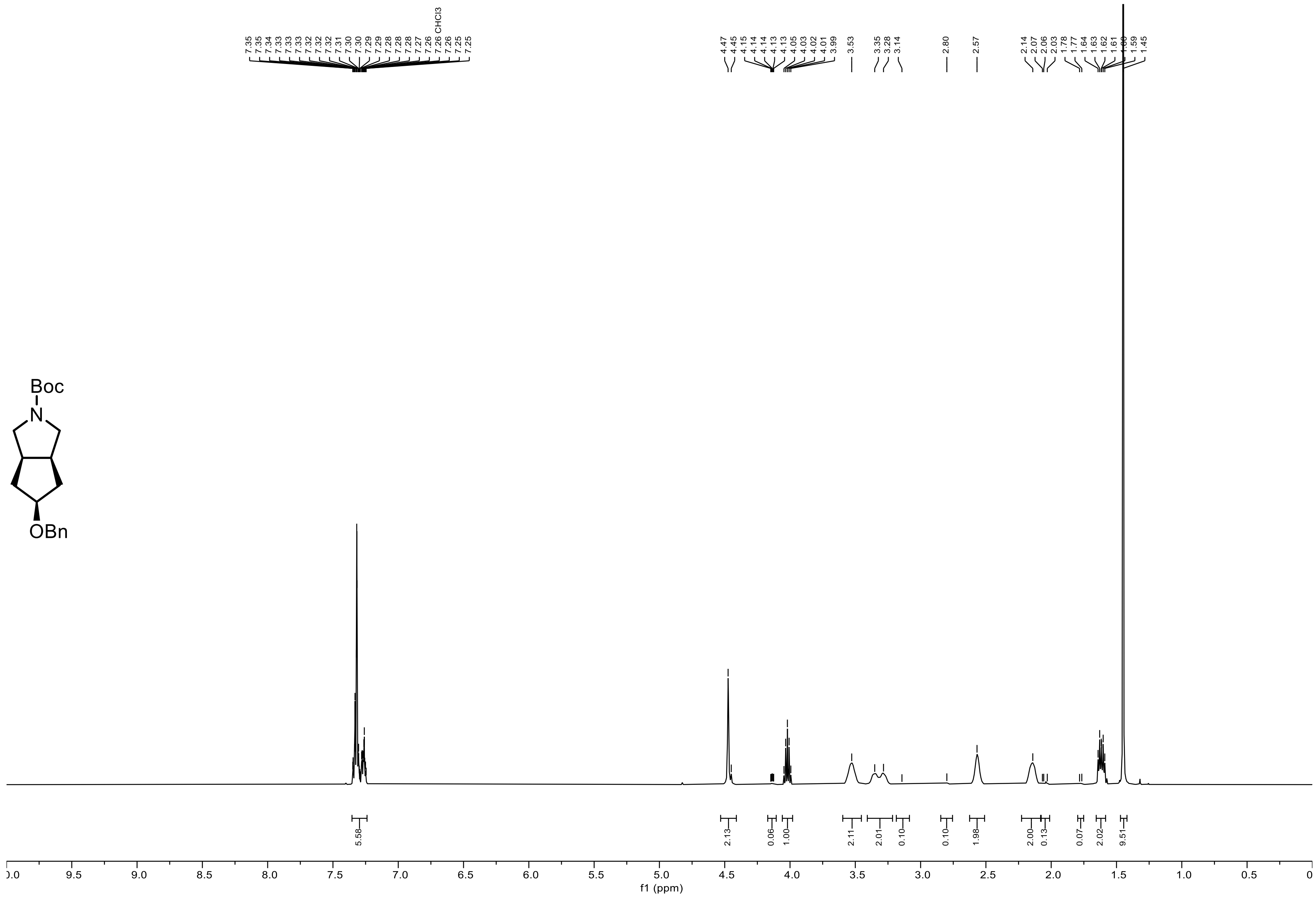

S18 ^{1}H NMR (500 MHz, $CDCl_3$).

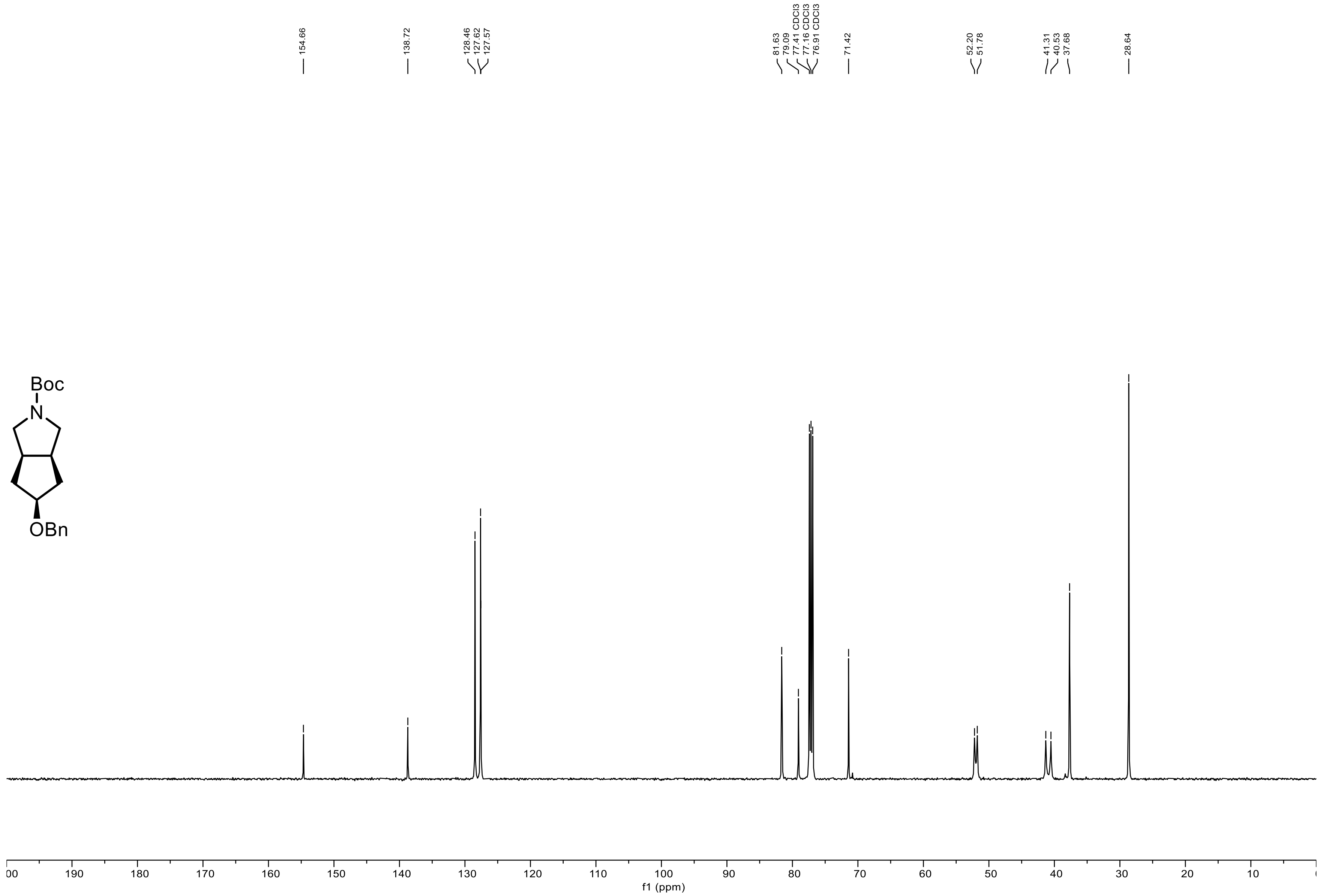

S18 ^{13}C NMR (126 MHz, $CDCl_3$).

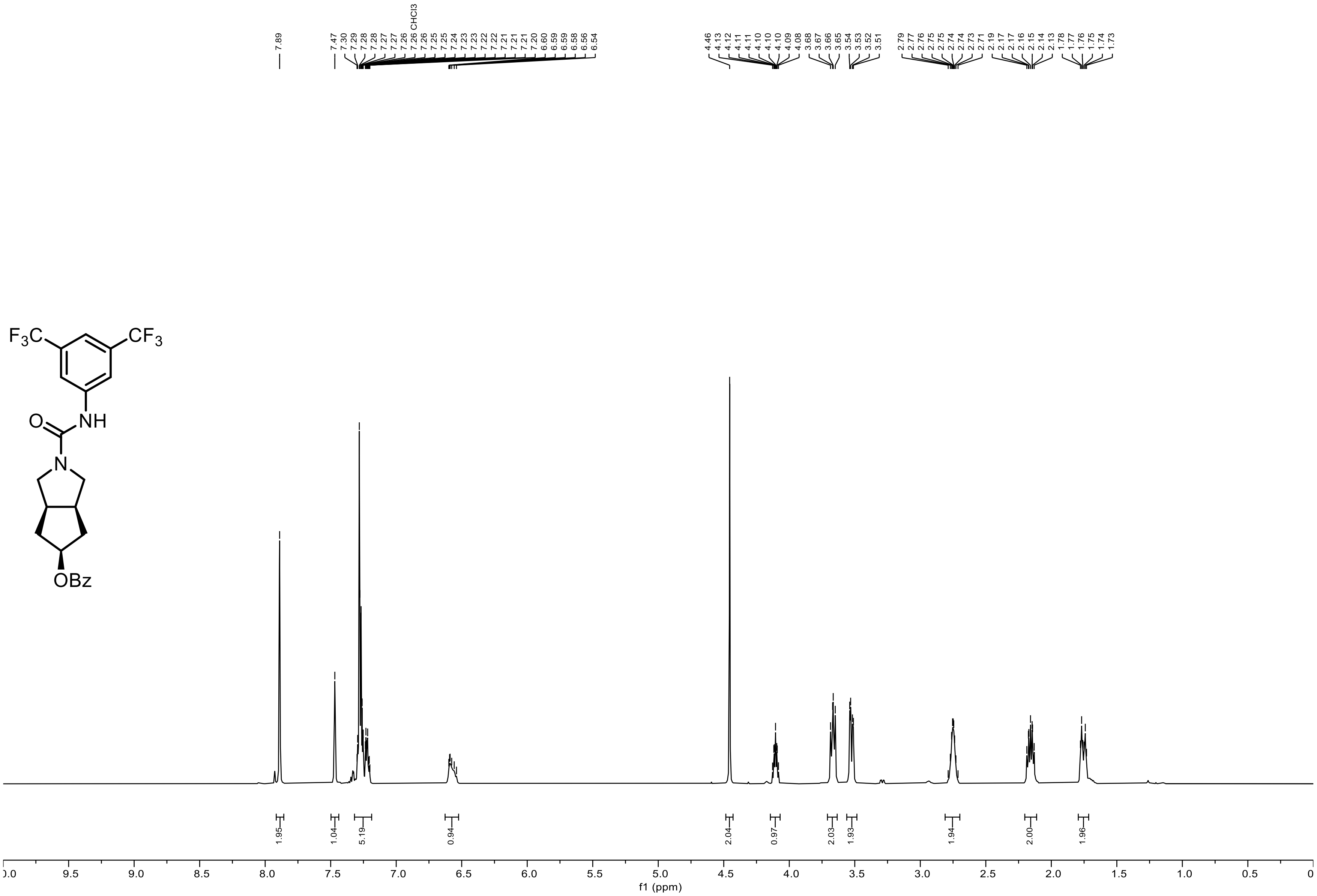

S19 ^{1}H NMR (500 MHz, $CDCl_3$).

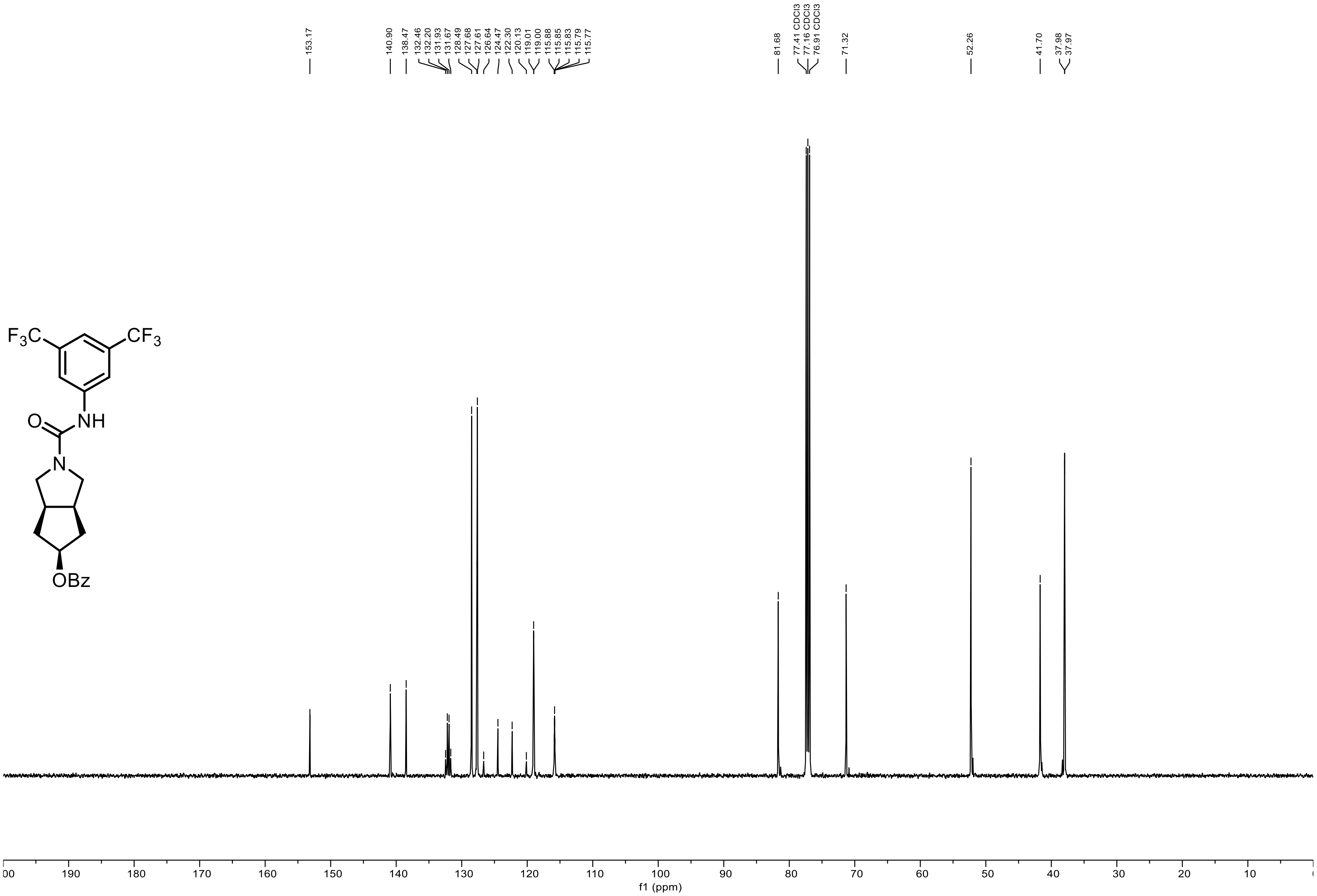

S19 ^{13}C NMR (126 MHz, $CDCl_3$).

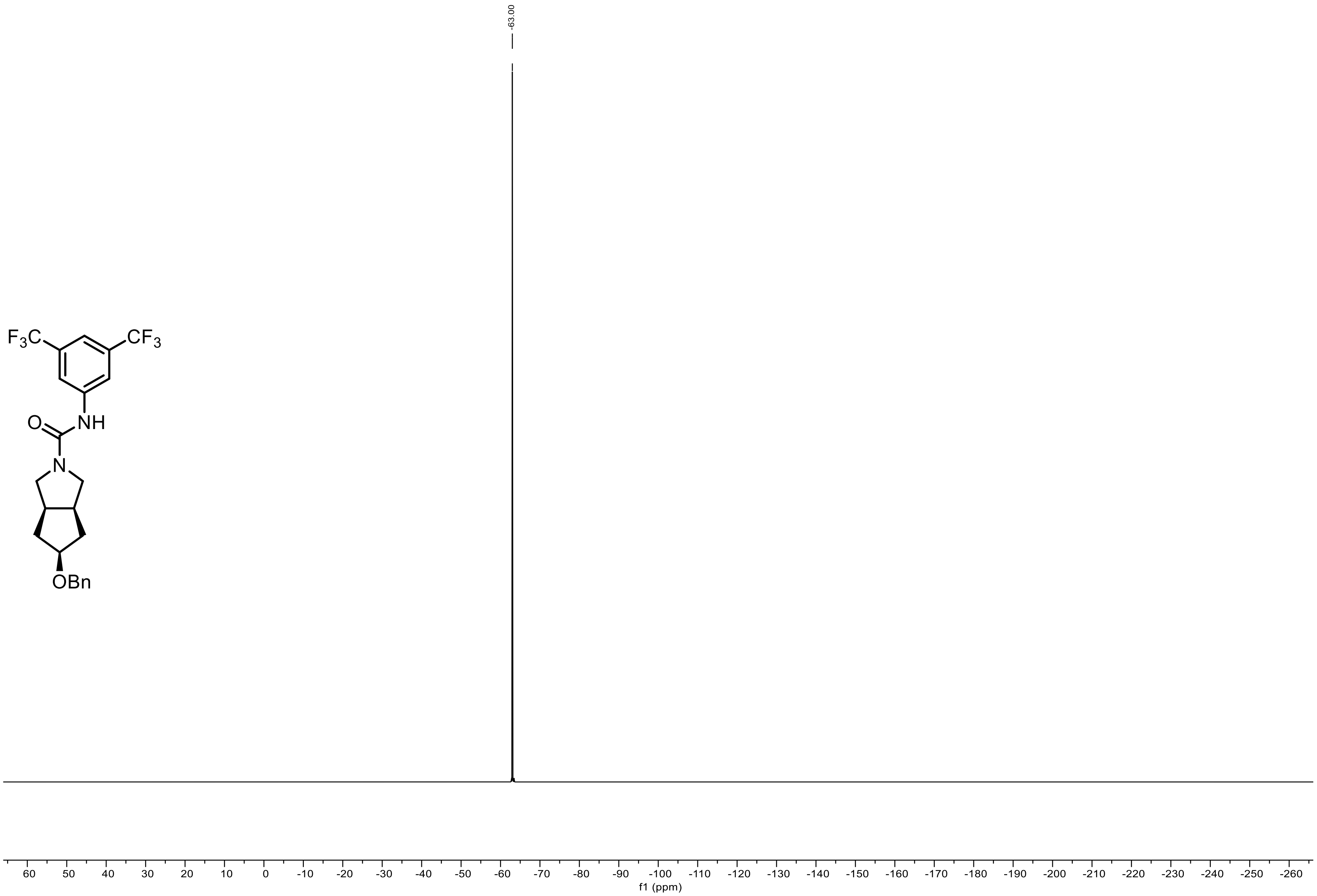

S19 ^{19}F NMR (470 MHz, $CDCl_3$).

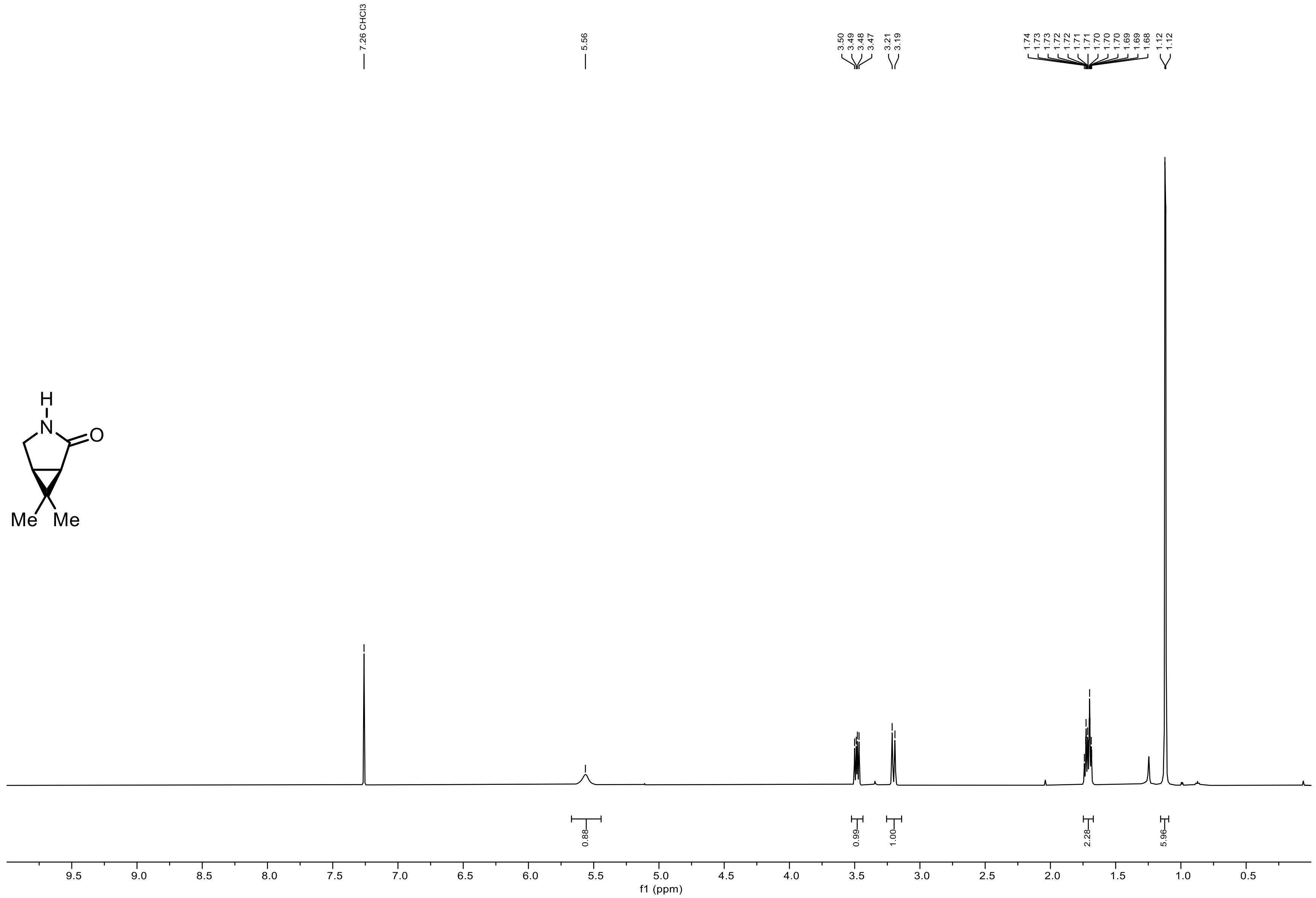

23 ^{1}H NMR (500 MHz, $CDCl_3$).

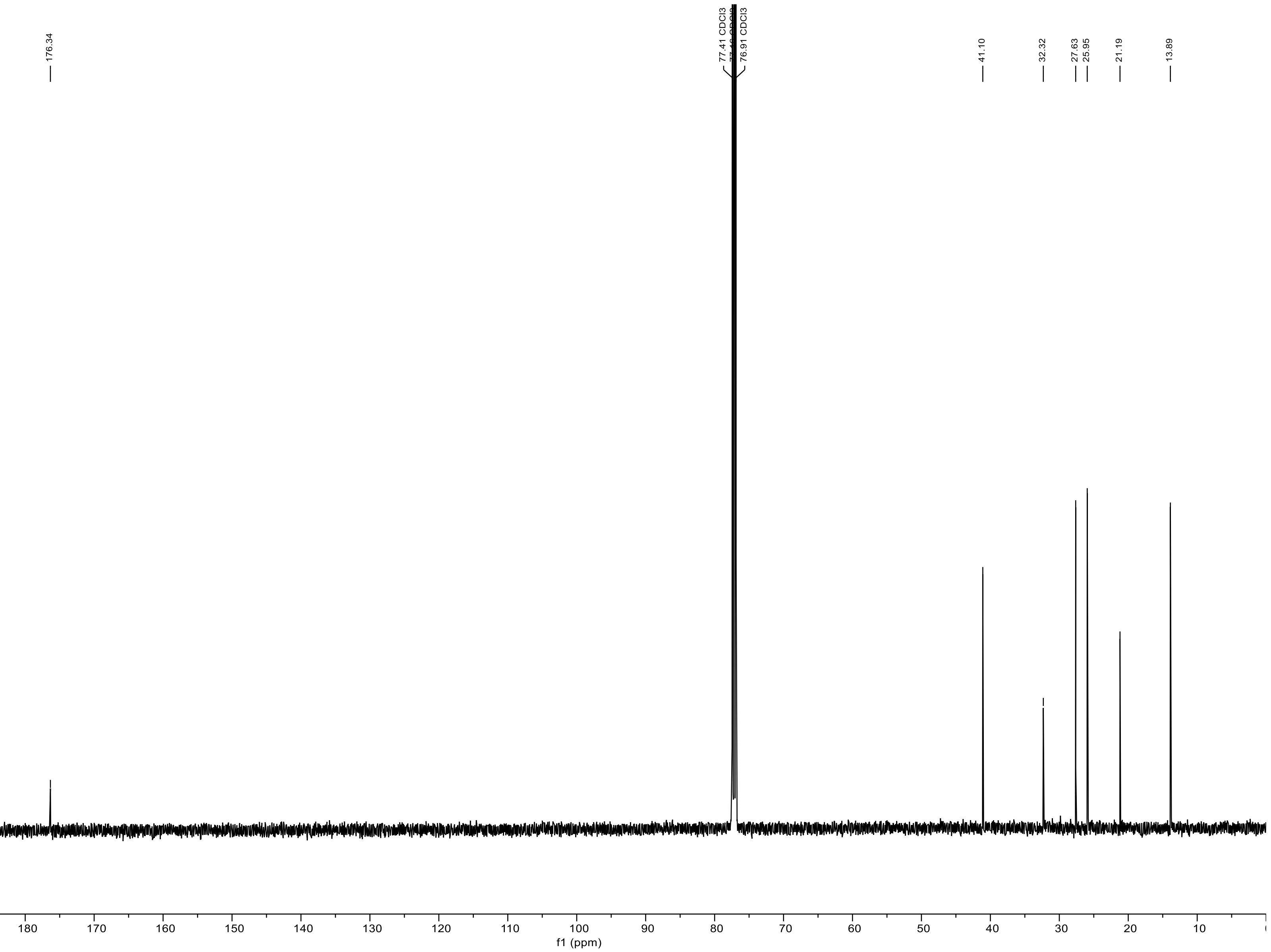

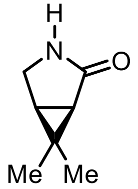

23 ^{13}C NMR (126 MHz, $CDCl_3$).

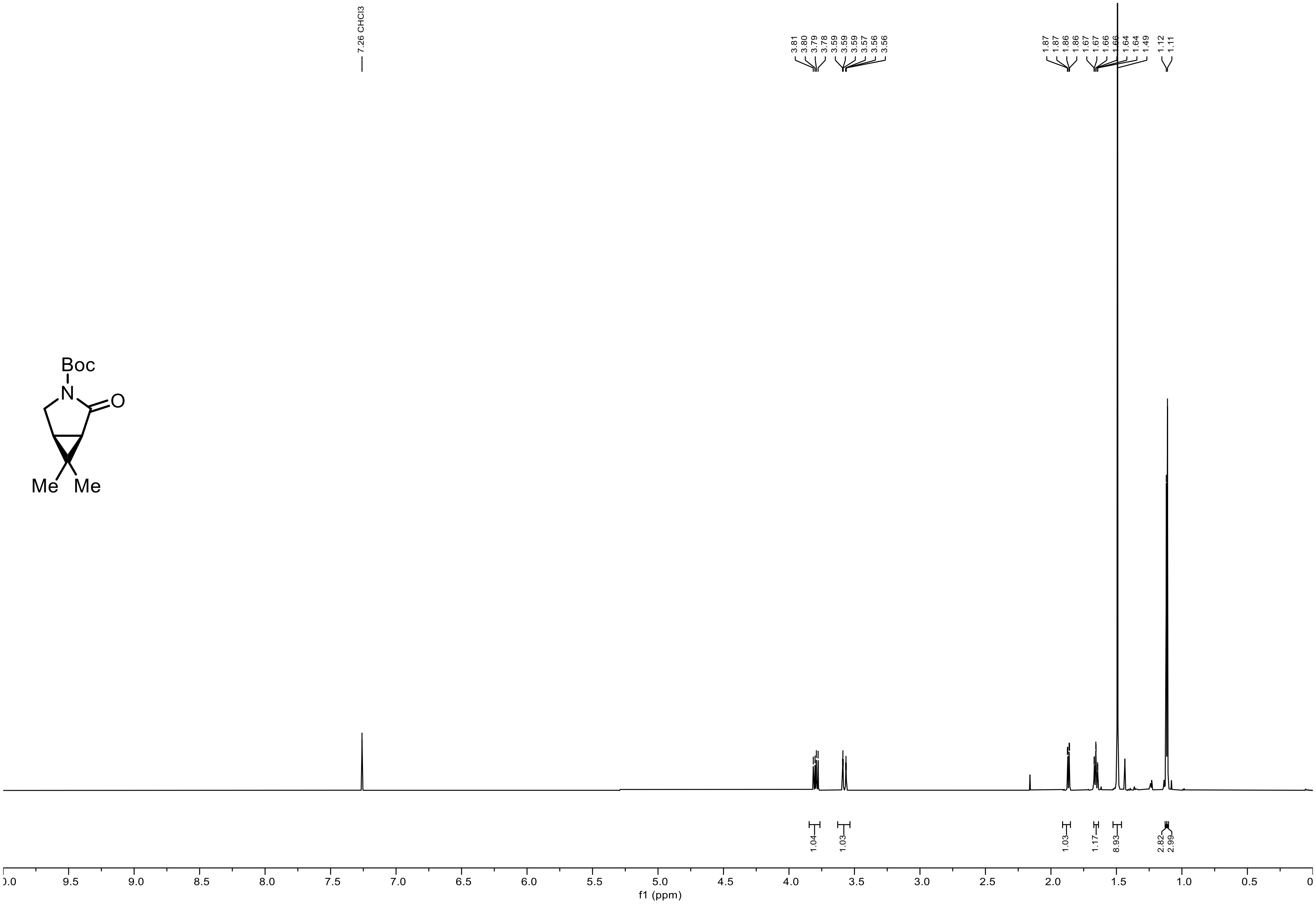

24 ^{1}H NMR (500 MHz, $CDCl_3$).

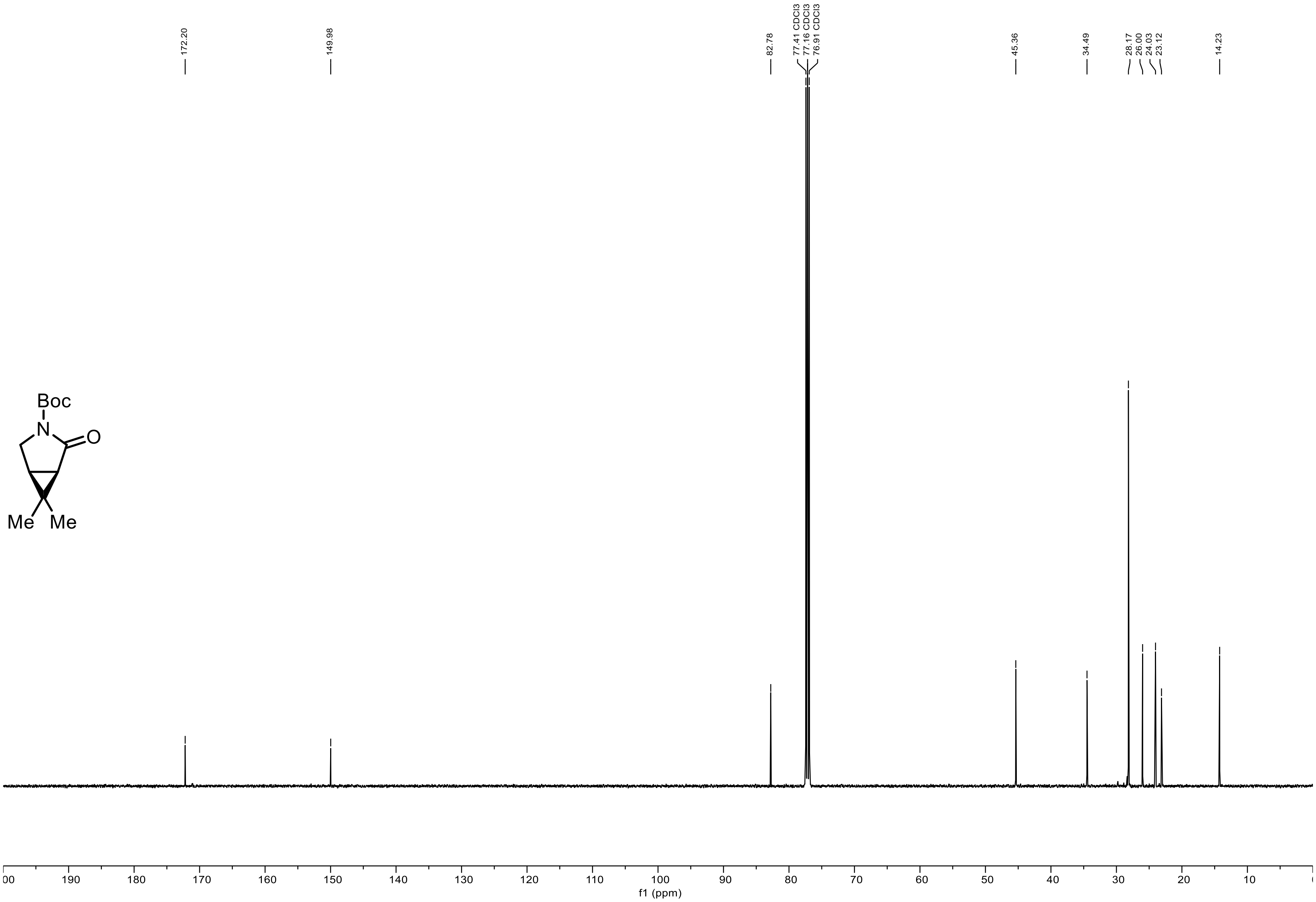

24 ^{13}C NMR (126 MHz, $CDCl_3$).

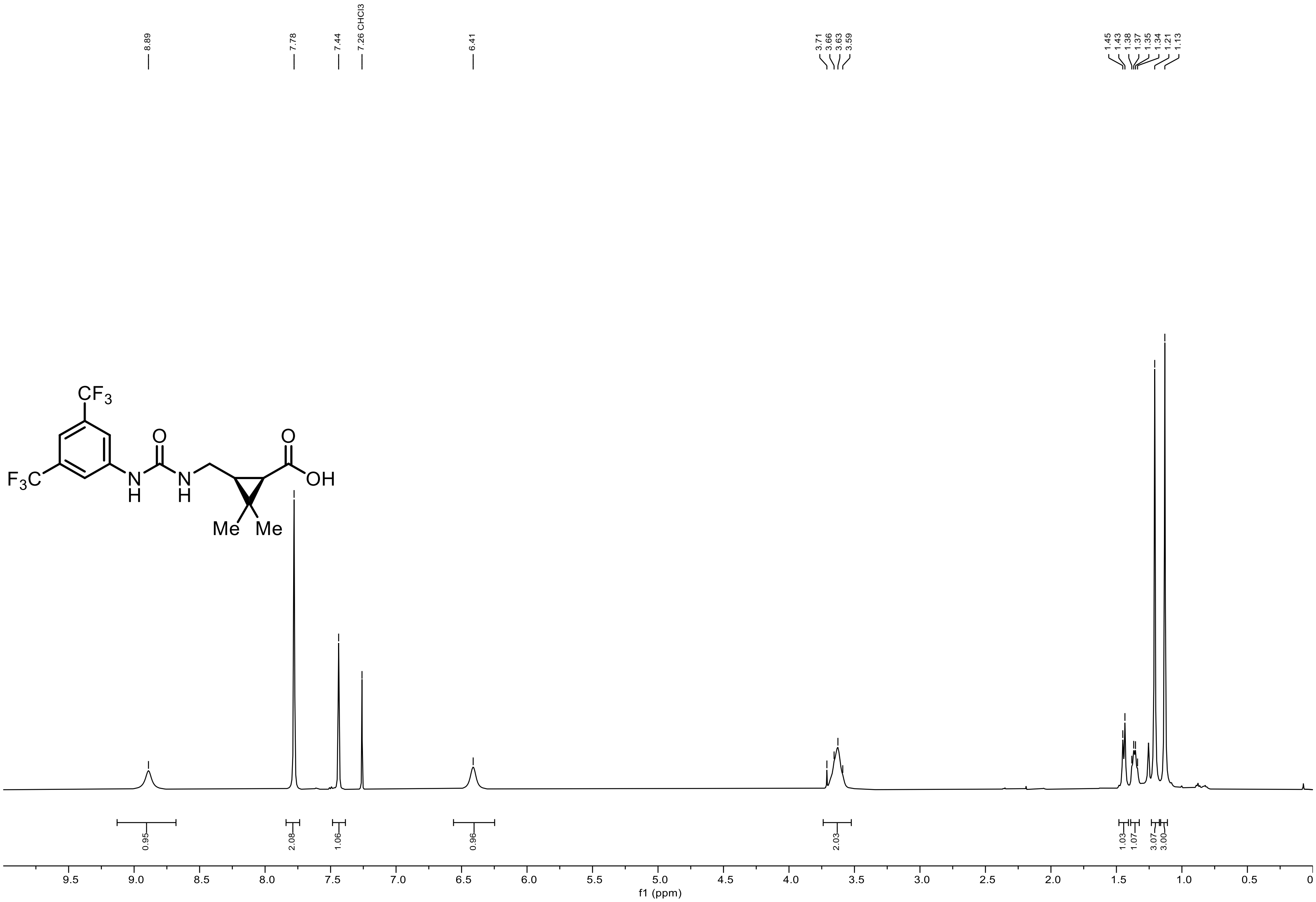

25 ^{1}H NMR (500 MHz, $CDCl_3$).

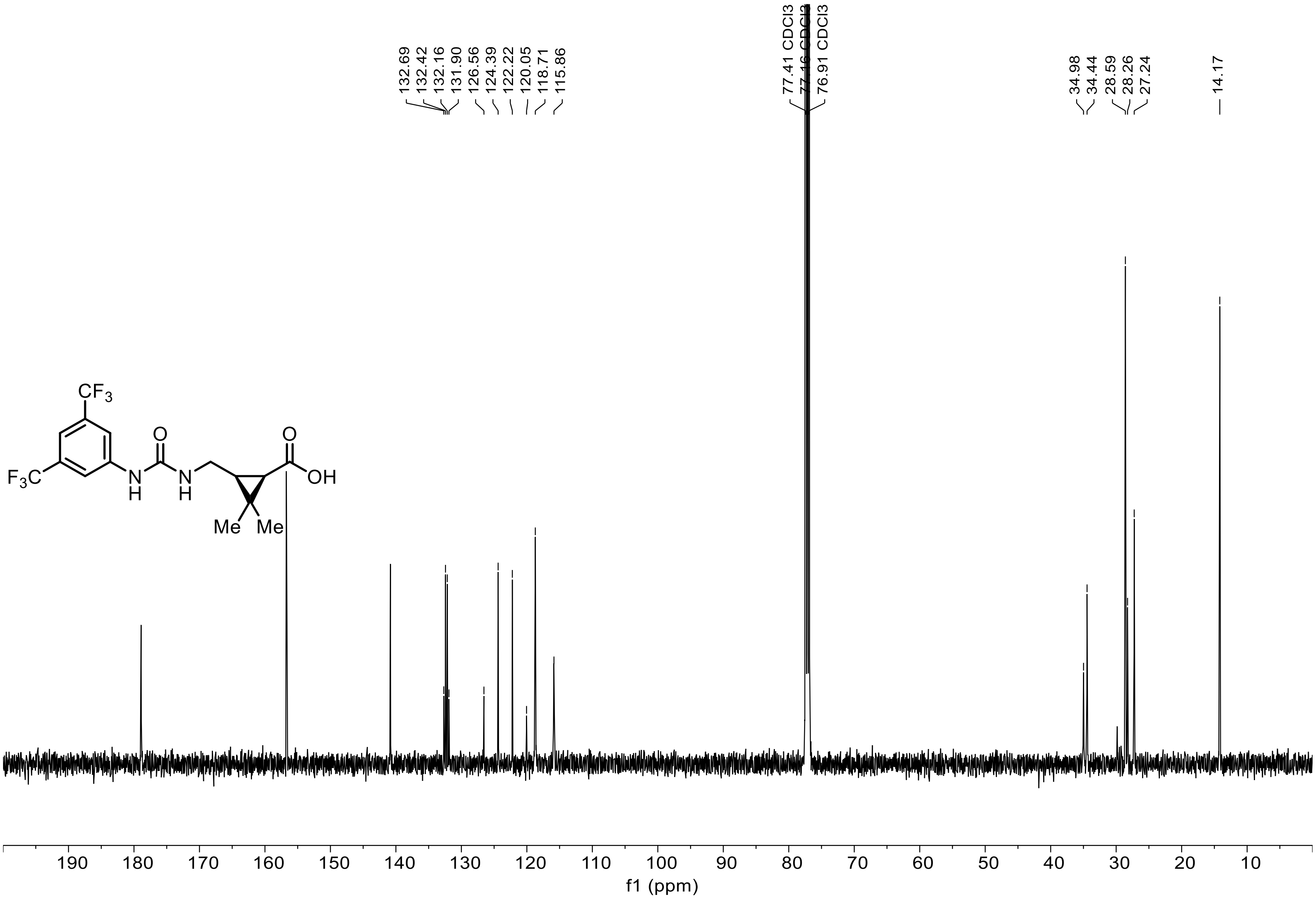

25 ^{13}C NMR (126 MHz, $CDCl_3$).

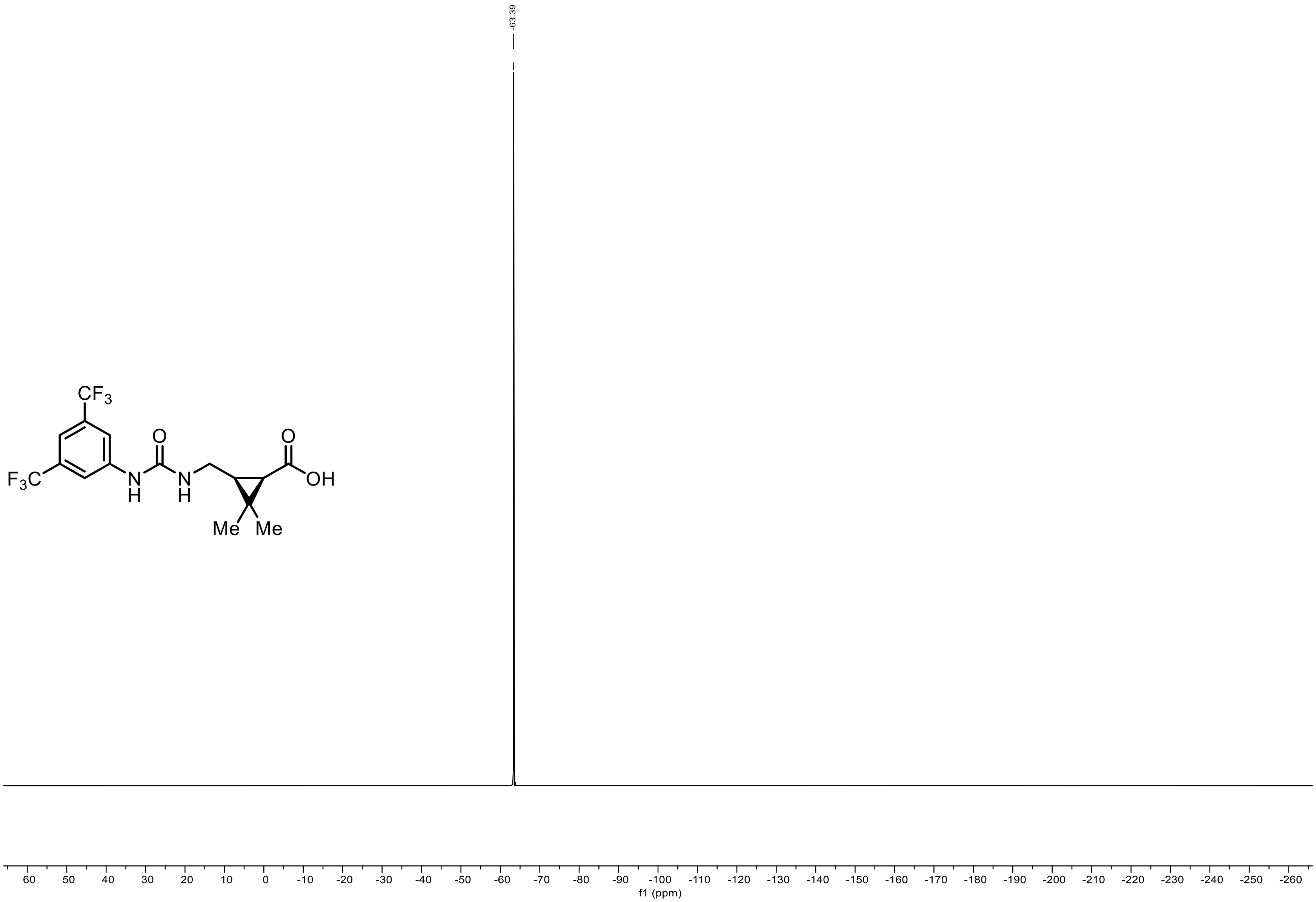

25 ^{19}F NMR (470 MHz, $CDCl_3$).

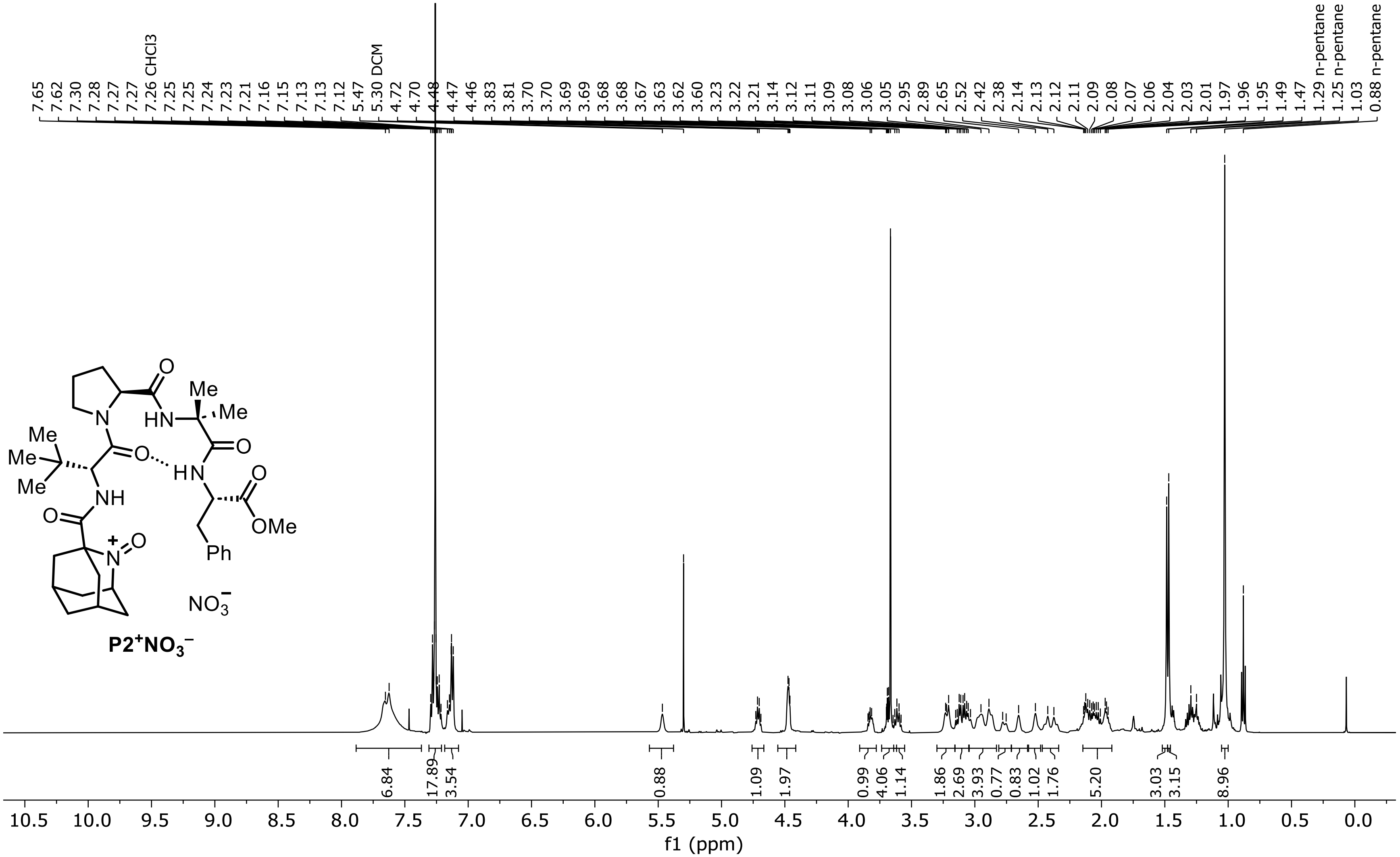

P2⁺NO₃⁻ ^{1}H NMR (600 MHz, $CDCl_3$). This sample was less pure based on integral areas and has lower signal to noise than the sample shown below in CD_2Cl_2. The sample is included for reference as it provides narrow peaks for the AzcH protons: δ 5.47 (s, 1H), 3.22 (m, 2H), 3.05 – 2.27 (m, 10H).

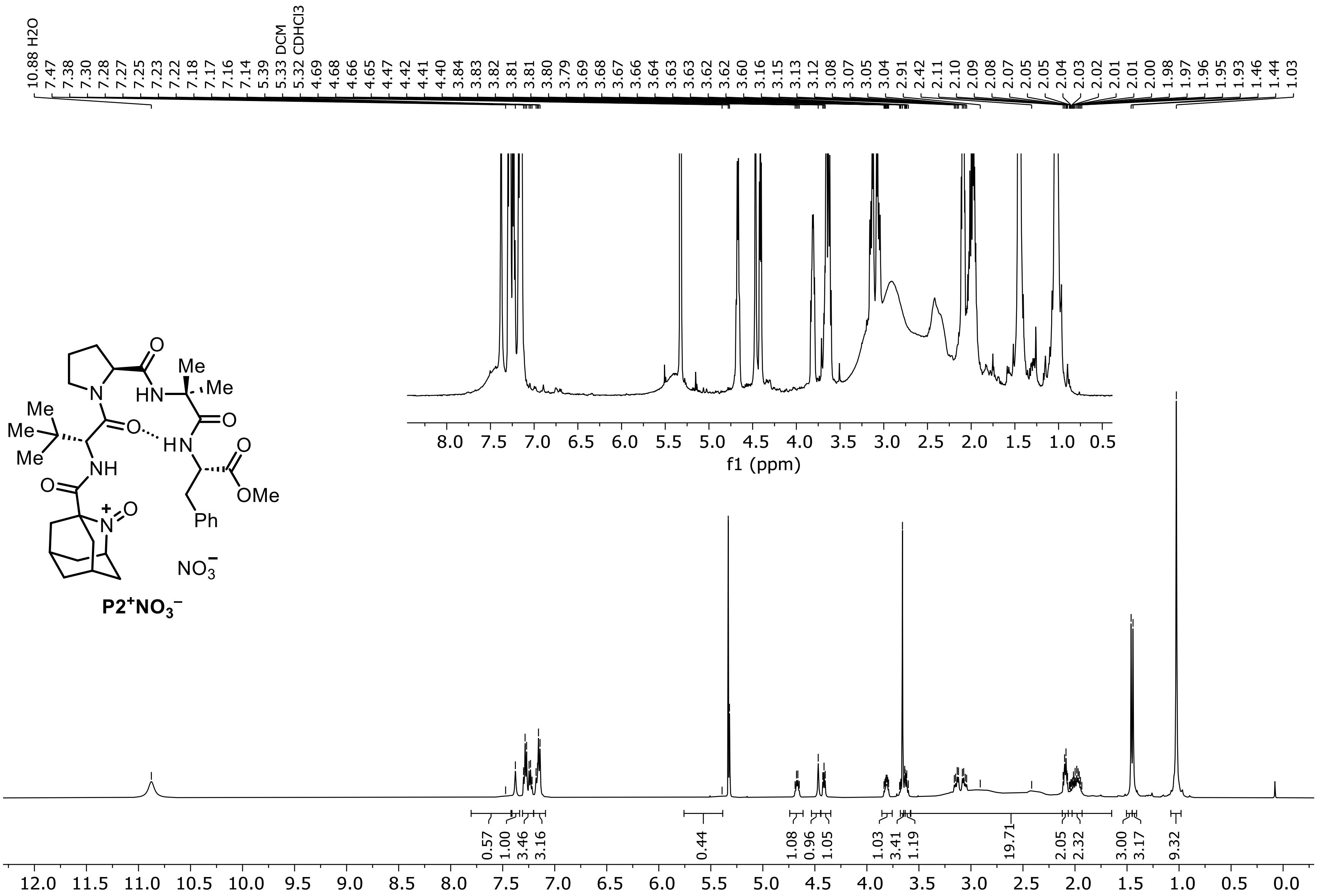

$P2^+NO_3^-$ ^{1}H NMR (500 MHz, CD_2Cl_2). **$P2^+NO_3^-$** *is a Lewis acid causing H_2O to shift downfield.*

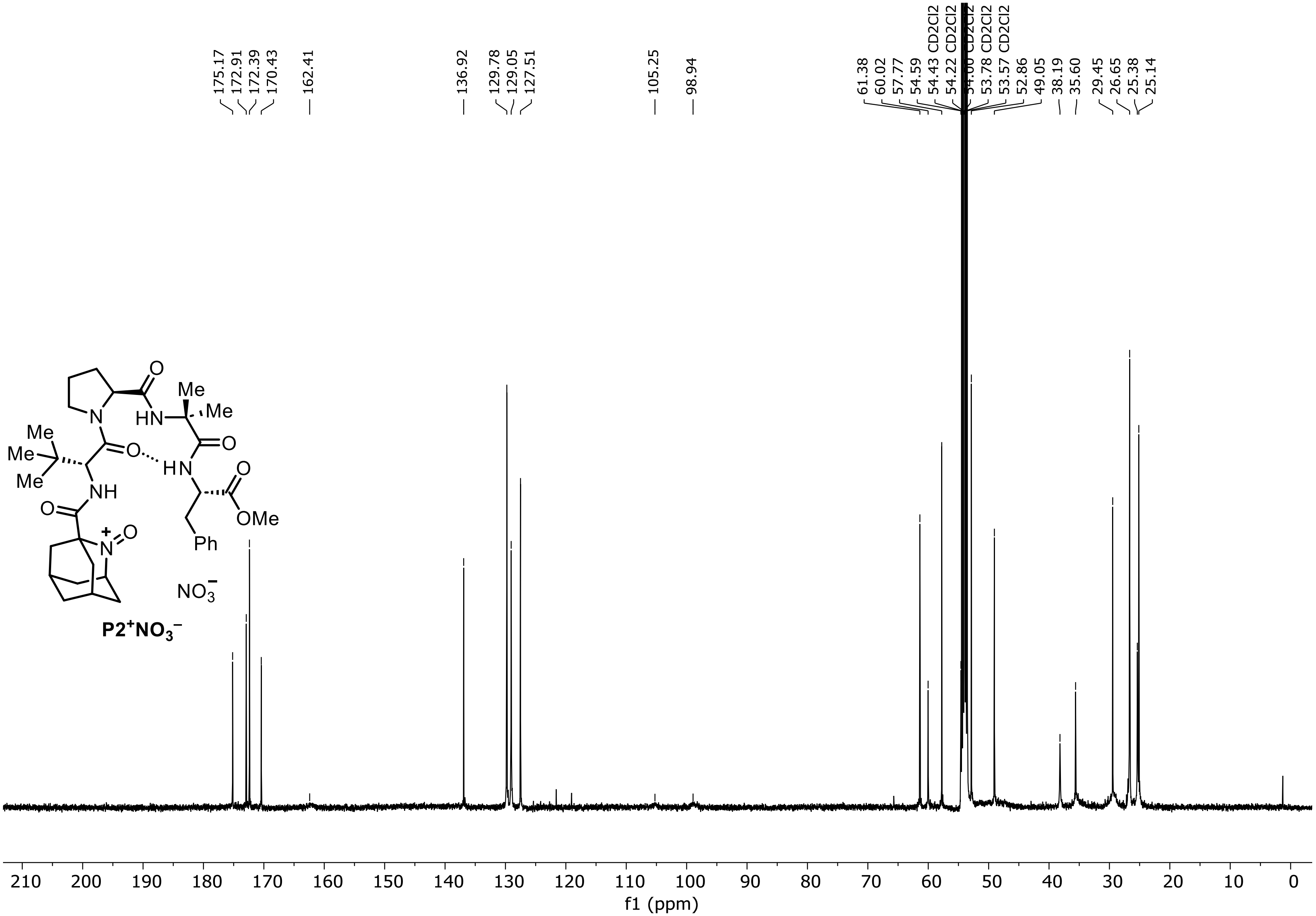

P2$^+$NO$_3^-$ ^{13}C NMR (126 MHz, CD_2Cl_2).

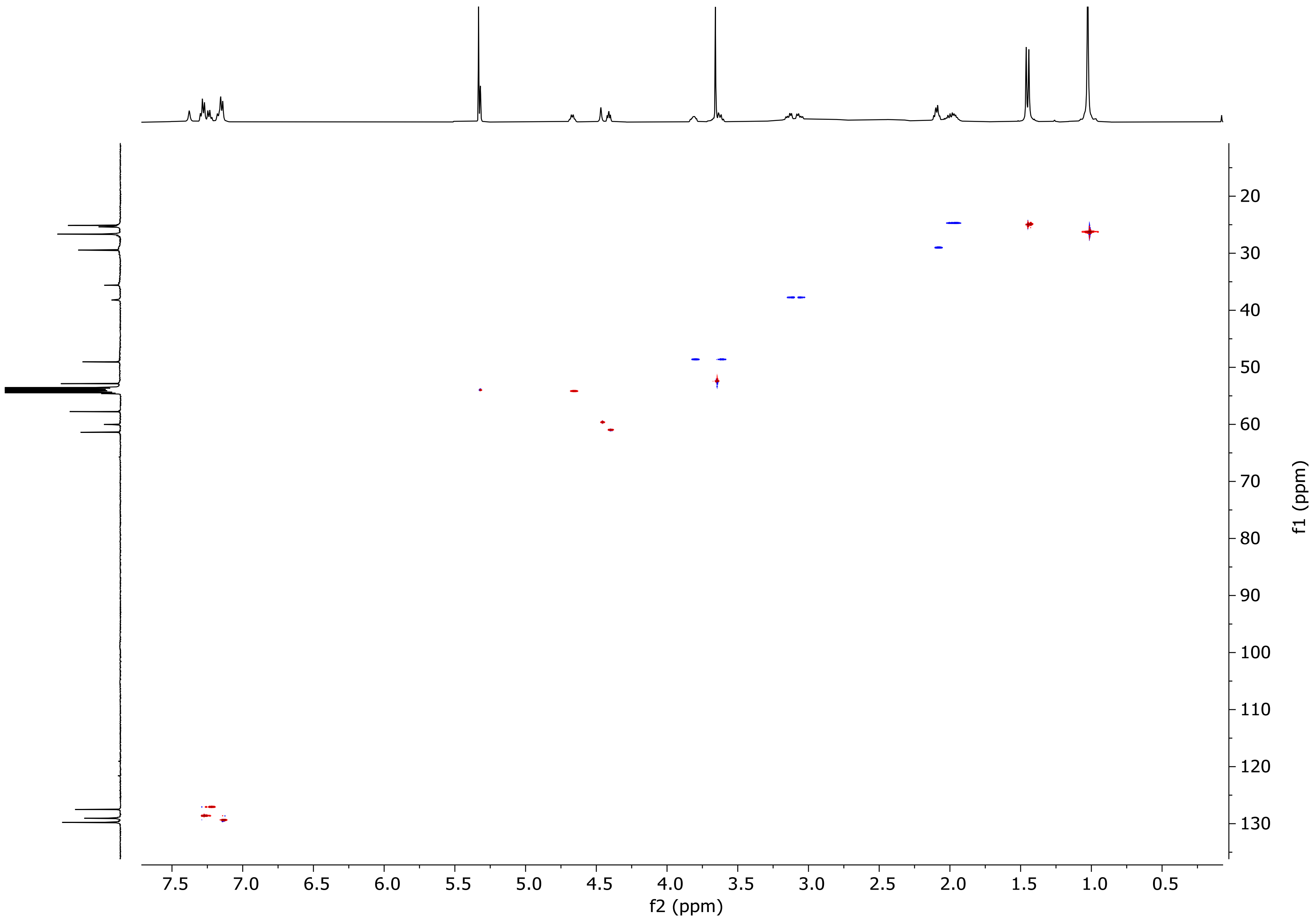

P2^{+}**NO**$_3^{-}$ HSQC (500 MHz; CD_2Cl_2).

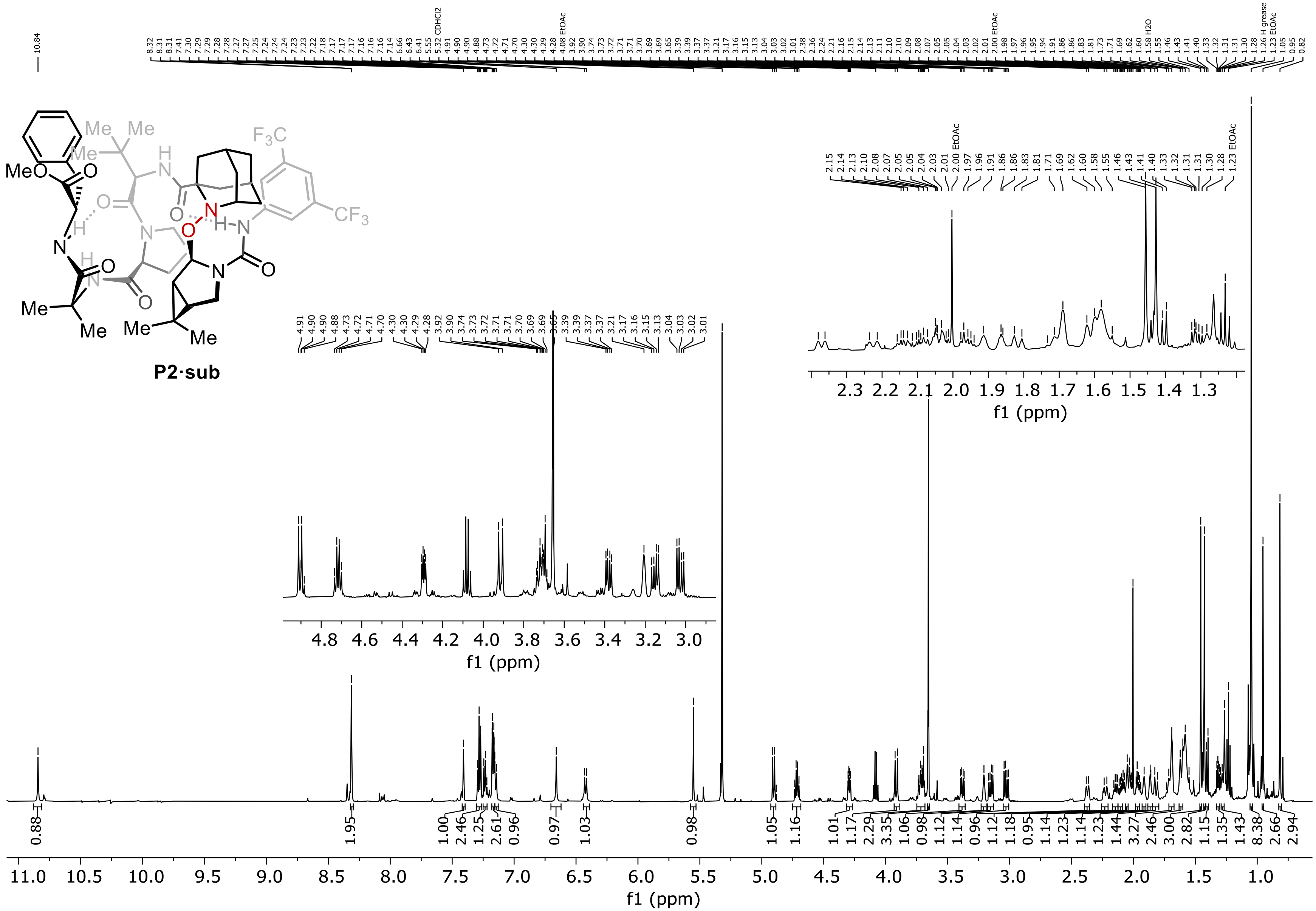

P2·sub ^{1}H NMR (599 MHz, CD_2Cl_2).

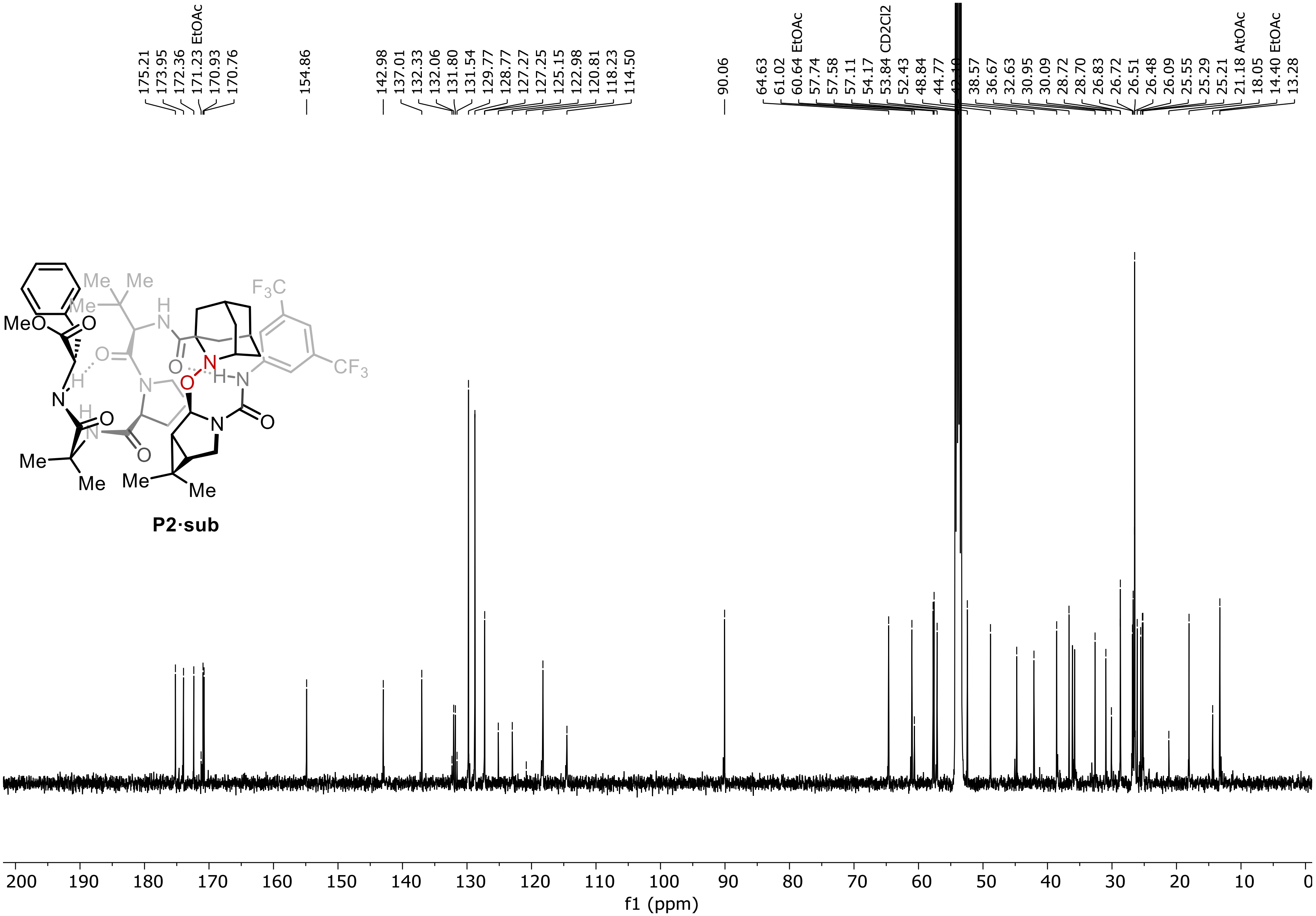

P2·sub ^{13}C NMR (126 MHz, CD_2Cl_2).

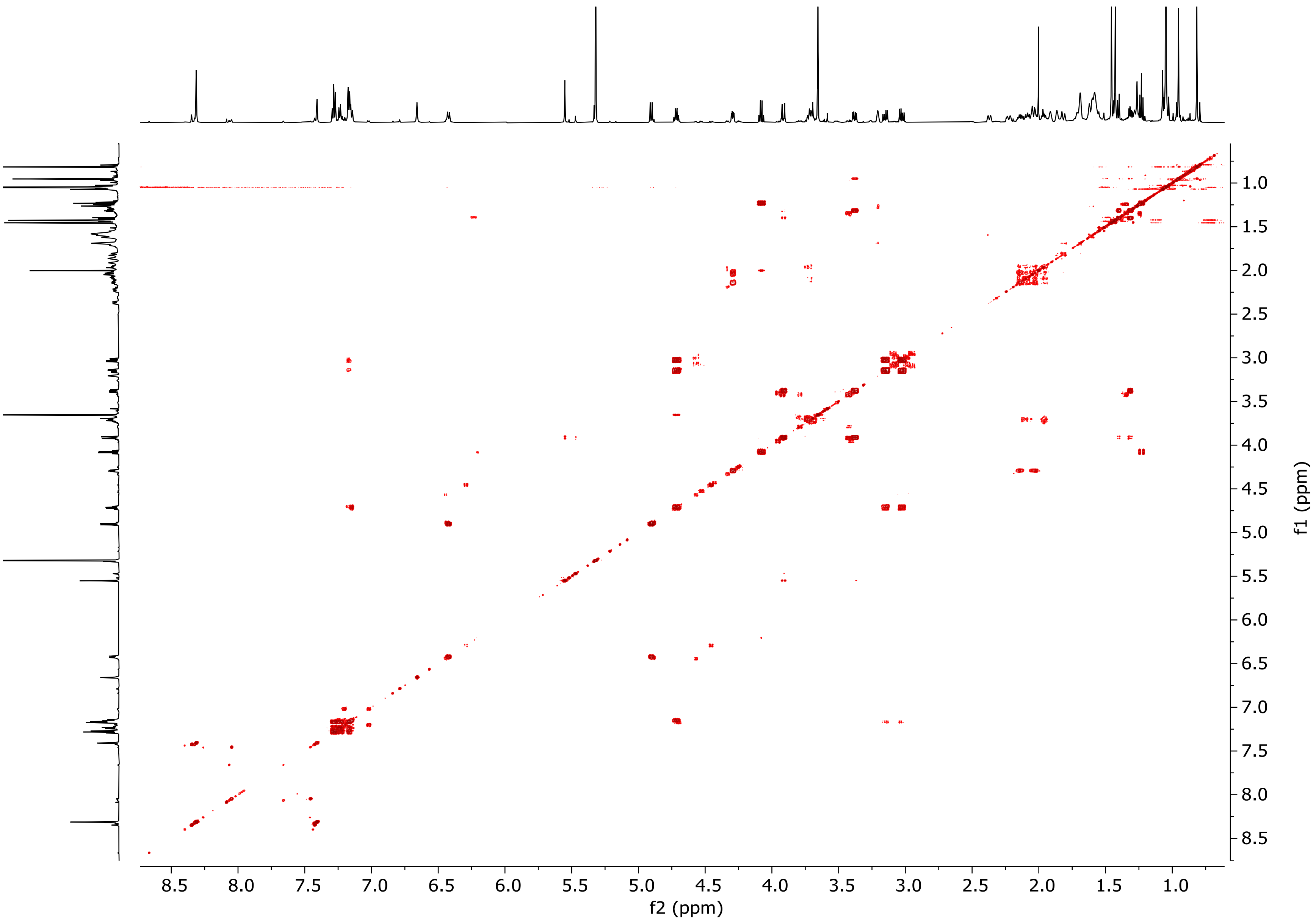

P2·sub gCOSY (500 MHz, CD_2Cl_2).

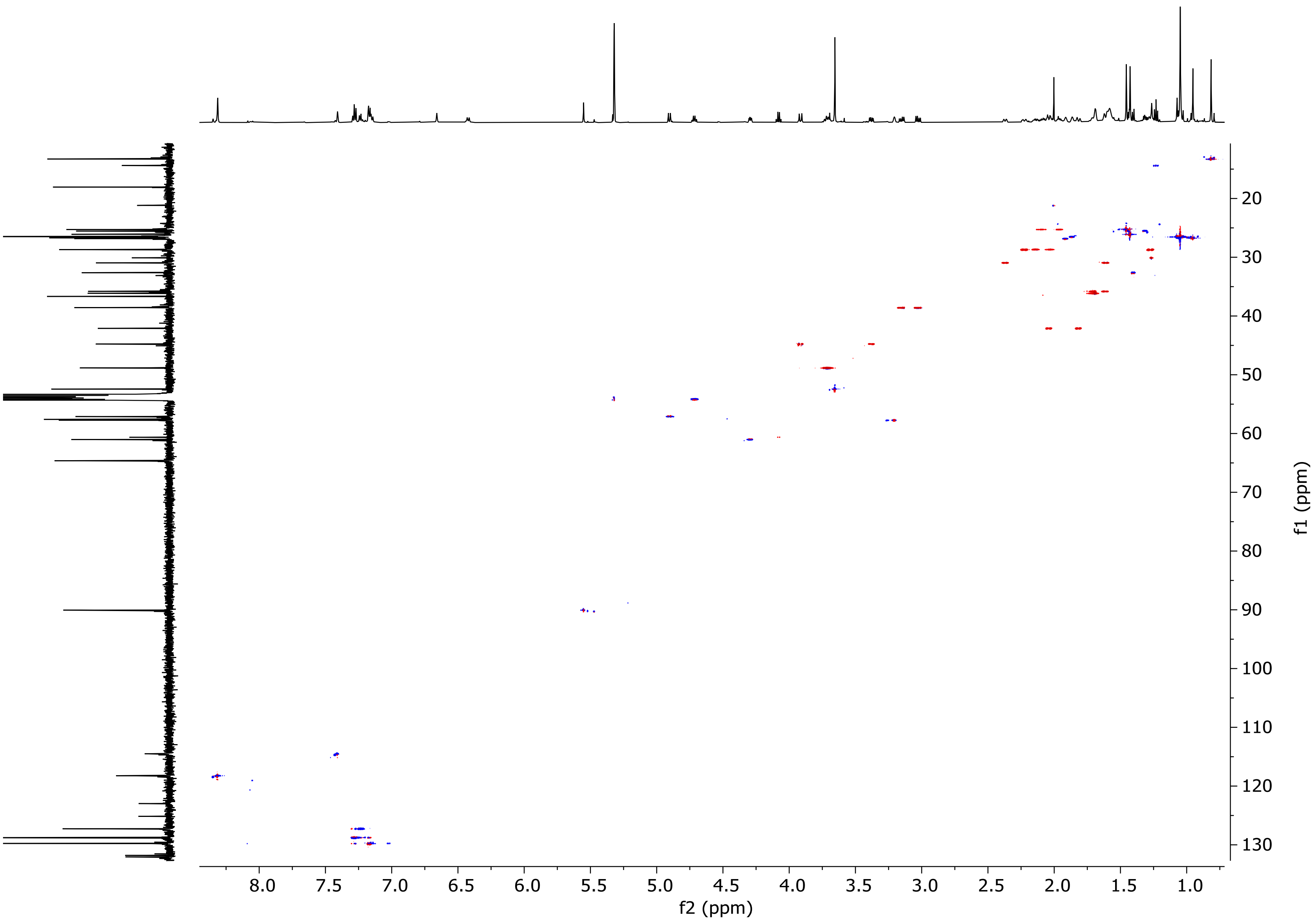

P2·sub HSQC (500 MHz, CD_2Cl_2).

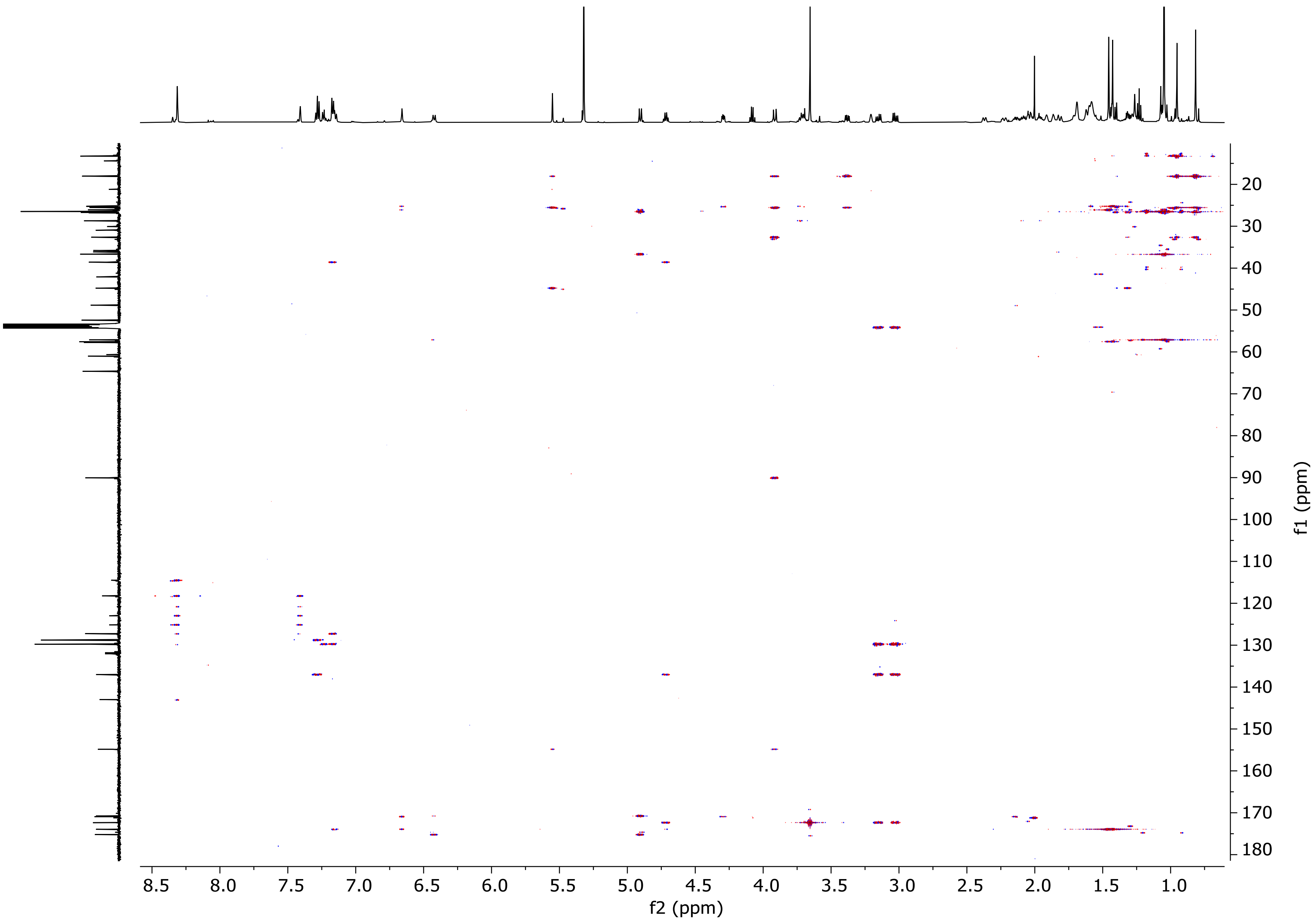

P2·sub HMBC (500 MHz, CD_2Cl_2).

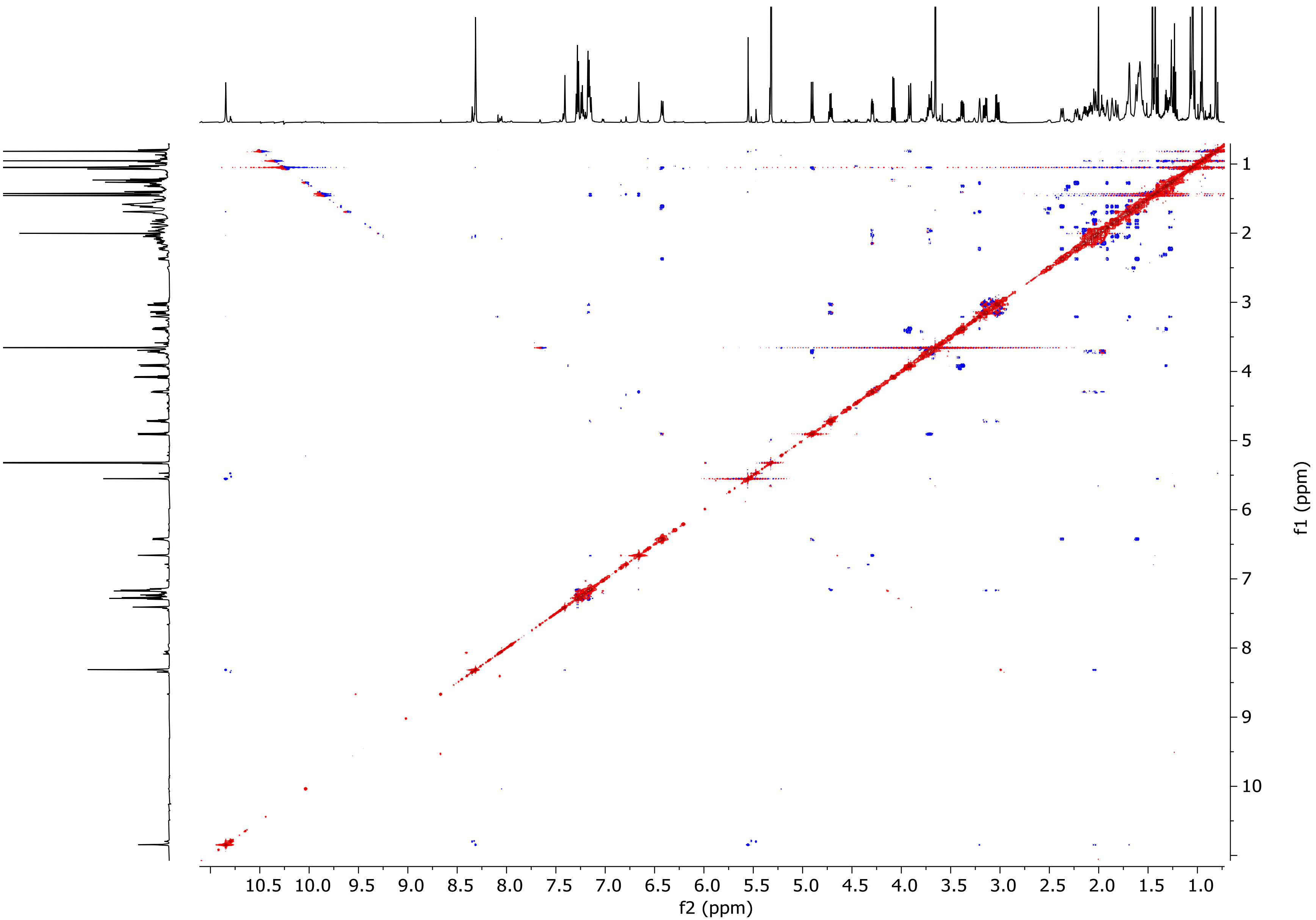

P2·sub ROESY (599 MHz, CD_2Cl_2).

11. References:

[1] Wagen, C. C.; McMinn, S. E.; Kwan, E. E.; Jacobsen, E. N. Screening for Generality in Asymmetric Catalysis. *Nature* **2022**, *610* (7933), 680–686. https://doi.org/10.1038/s41586-022-05263-2.

[2] Mansson, C. M. F.; Burns, N. Z. Aqueous Amine-Tolerant [2+2] Photocycloadditions of Unactivated Olefins. *J. Am. Chem. Soc.* **2022**, *144*, 19689–19694.

[3] Rein, J.; Górski, B.; Cheng, Y.; Lei, Z.; Buono, F.; Lin, S. Oxoammonium-Catalyzed Oxidation of N-Substituted Amines. *J. Am. Chem. Soc.* **2024**, *146*, 31412–31419.

[4] Cheng, Y.; Rein, J.; Le, N.; Lin, S. Oxoammonium-Catalyzed Ether Oxidation via Hydride Abstraction: Methodology Development and Mechanistic Investigation Using Paramagnetic Relaxation Enhancement NMR. *J. Am. Chem. Soc.* **2024**, *146*, 31420–31432.

[5] Bobbitt, J. M.; Eddy, N. A.; Cady, C. X.; Jin, J.; Gascon, J. A.; Gelpí-Dominguez, S.; Zakrzewski, J.; Morton, M. D. Preparation of Some Homologous TEMPO Nitroxides and Oxoammonium Salts; Notes on the NMR Spectroscopy of Nitroxide Free Radicals; Observed Radical Nature of Oxoammonium Salt Solutions Containing Trace Amounts of Corresponding Nitroxides in an Equilibrium Relationship. *J. Org. Chem.* **2017**, *82*, 9279–9290.

[6] Metrano, A. J.; Abascal, N. C.; Mercado, B. Q.; Paulson, E. K.; Hurtley, A. E.; Miller, S. J. Diversity of Secondary Structure in Catalytic Peptides with β-Turn-Biased Sequences. *J. Am. Chem. Soc.* **2017**, *139*, 492–516.

[7] Metrano, A. J.; Abascal, N. C.; Mercado, B. Q.; Paulson, E. K.; Hurtley, A. E.; Miller, S. J. Diversity of Secondary Structure in Catalytic Peptides with β-Turn-Biased Sequences. *J. Am. Chem. Soc.* **2017**, *139*, 492–516.

[8] Pracht, P.; Grimme, S.; Bannwarth, C.; Bohle, F.; Ehlert, S.; Feldmann, G.; Gorges, J.; Müller, M.; Neudecker, T.; Plett, C.; Spicher, S.; Steinbach, P.; Wesołowski, P. A.; Zeller, F. CREST—A Program for the Exploration of Low-Energy Molecular Chemical Space. *The Journal of Chemical Physics* **2024**, *160*, 114110.

[9] Anstine, D. M.; Zubatyuk, R.; Isayev, O. AIMNet2: A Neural Network Potential to Meet Your Neutral, Charged, Organic, and Elemental-Organic Needs. *Chem. Sci.* **2025**, *16* (23), 10228–10244. https://doi.org/10.1039/D4SC08572H.

[10] *Rowan Scientific*. https://www.rowansci.com (accessed 2024-09-20).

[11] CYLview20; Legault, C. Y., Université de Sherbrooke, 2020 (http://www.cylview.org)

[12] Zhao, Y.; Truhlar, D. G. The M06 Suite of Density Functionals for Main Group Thermochemistry, Thermochemical Kinetics, Noncovalent Interactions, Excited States, and

Transition Elements: Two New Functionals and Systematic Testing of Four M06-Class Functionals and 12 Other Functionals. *Theor Chem Account* **2008**, *120* (1), 215–241. https://doi.org/10.1007/s00214-007-0310-x.

[13] Frisch, M. J., Trucks, G. W., Schlegel, H. B., Scuseria, G. E., Robb, M. A., Cheeseman, J. R., Scalmani, G., Barone, V., Petersson, G. A., Nakatsuji, H., Li, X., Caricato, M., Marenich, A. V., Bloino, J., Janesko, B. G., Gomperts, R., Mennucci, B., Hratchian, H. P., Ortiz, J. V., Izmaylov, A. F., Sonnenberg, J. L., Williams-Young, D., Ding, F., Lipparini, F., Egidi, F., Goings, J., Peng, B., Petrone, A., Henderson, T., Ranasinghe, D., Zakrzewski, V. G., Gao, J., Rega, N., Zheng, G., Liang, W., Hada, M., Ehara, M., Toyota, K., Fukuda, R., Hasegawa, J., Ishida, M., Nakajima, T., Honda, Y., Kitao, O., Nakai, H., Vreven, T., Throssell, K., Montgomery, J. A., Jr., Peralta, J. E., Ogliaro, F., Bearpark, M. J., Heyd, J. J., Brothers, E. N., Kudin, K. N., Staroverov, V. N., Keith, T. A., Kobayashi, R., Normand, J., Raghavachari, K., Rendell, A. P., Burant, J. C., Iyengar, S. S., Tomasi, J., Cossi, M., Millam, J. M., Klene, M., Adamo, C., Cammi, R., Ochterski, J. W., Martin, R. L., Morokuma, K., Farkas, O., Foresman, J. B., Fox, D. J. Gaussian 16 Revision C.01.

[14] Frisch, M. J., Trucks, G. W., Schlegel, H. B., Scuseria, G. E., Robb, M. A., Cheeseman, J. R., Scalmani, G., Barone, V., Petersson, G. A., Nakatsuji, H., Li, X., Caricato, M., Marenich, A. V., Bloino, J., Janesko, B. G., Gomperts, R., Mennucci, B., Hratchian, H. P., Ortiz, J. V., Izmaylov, A. F., Sonnenberg, J. L., Williams-Young, D., Ding, F., Lipparini, F., Egidi, F., Goings, J., Peng, B., Petrone, A., Henderson, T., Ranasinghe, D., Zakrzewski, V. G., Gao, J., Rega, N., Zheng, G., Liang, W., Hada, M., Ehara, M., Toyota, K., Fukuda, R., Hasegawa, J., Ishida, M., Nakajima, T., Honda, Y., Kitao, O., Nakai, H., Vreven, T., Throssell, K., Montgomery, J. A., Jr., Peralta, J. E., Ogliaro, F., Bearpark, M. J., Heyd, J. J., Brothers, E. N., Kudin, K. N., Staroverov, V. N., Keith, T. A., Kobayashi, R., Normand, J., Raghavachari, K., Rendell, A. P., Burant, J. C., Iyengar, S. S., Tomasi, J., Cossi, M., Millam, J. M., Klene, M., Adamo, C., Cammi, R., Ochterski, J. W., Martin, R. L., Morokuma, K., Farkas, O., Foresman, J. B., Fox, D. J. Gaussian 16 Revision C.01.

[15] Pritchard, B. P.; Altarawy, D.; Didier, B.; Gibson, T. D.; Windus, T. L. New Basis Set Exchange: An Open, Up-to-Date Resource for the Molecular Sciences Community. *J. Chem. Inf. Model.* **2019**, *59*, 4814–4820.
